# A Vulnerability Assessment of Fish and Invertebrates to Climate Change on the Northeast U.S. Continental Shelf

**DOI:** 10.1371/journal.pone.0146756

**Published:** 2016-02-03

**Authors:** Jonathan A. Hare, Wendy E. Morrison, Mark W. Nelson, Megan M. Stachura, Eric J. Teeters, Roger B. Griffis, Michael A. Alexander, James D. Scott, Larry Alade, Richard J. Bell, Antonie S. Chute, Kiersten L. Curti, Tobey H. Curtis, Daniel Kircheis, John F. Kocik, Sean M. Lucey, Camilla T. McCandless, Lisa M. Milke, David E. Richardson, Eric Robillard, Harvey J. Walsh, M. Conor McManus, Katrin E. Marancik, Carolyn A. Griswold

**Affiliations:** 1 NOAA NMFS Northeast Fisheries Science Center, Narragansett Laboratory, 28 Tarzwell Drive, Narragansett, Rhode Island, 02818, United States of America; 2 Earth Resources Technology, Inc. Under contract for NOAA NMFS, Office of Sustainable Fisheries, 1315 East West Highway, Silver Spring, Maryland 20910, United States of America; 3 NOAA NMFS, Office of Sustainable Fisheries, 1315 East West Highway, Silver Spring, Maryland 20910, United States of America; 4 NOAA NMFS, Office of Science and Technology, 1315 East West Highway, Silver Spring, Maryland 20910, United States of America; 5 NOAA OAR Earth Systems Research Laboratory, 325 Broadway, Boulder, Colorado 80305–3337, United States of America; 6 NOAA NMFS Northeast Fisheries Science Center, Woods Hole Laboratory, 166 Water Street, Woods Hole, Massachusetts 02543, United States of America; 7 NOAA NMFS Greater Atlantic Regional Fisheries Office, 55 Great Republic Drive, Gloucester, Massachusetts, 01930, United States of America; 8 NOAA NMFS Northeast Fisheries Science Center, Maine Field Station, 17 Godfrey Drive-Suite 1, Orono, Maine 04473, United States of America; 9 NOAA NMFS Northeast Fisheries Science Center, Milford Laboratory, 212 Rogers Ave, Milford, Connecticut 06460, United States of America; 10 Integrated Statistics Under contract for NOAA NMFS Northeast Fisheries Science Center, Narragansett Laboratory, 28 Tarzwell Drive, Narragansett, Rhode Island, 02818, United States of America; Bangor University, UNITED KINGDOM

## Abstract

Climate change and decadal variability are impacting marine fish and invertebrate species worldwide and these impacts will continue for the foreseeable future. Quantitative approaches have been developed to examine climate impacts on productivity, abundance, and distribution of various marine fish and invertebrate species. However, it is difficult to apply these approaches to large numbers of species owing to the lack of mechanistic understanding sufficient for quantitative analyses, as well as the lack of scientific infrastructure to support these more detailed studies. Vulnerability assessments provide a framework for evaluating climate impacts over a broad range of species with existing information. These methods combine the exposure of a species to a stressor (climate change and decadal variability) and the sensitivity of species to the stressor. These two components are then combined to estimate an overall vulnerability. Quantitative data are used when available, but qualitative information and expert opinion are used when quantitative data is lacking. Here we conduct a climate vulnerability assessment on 82 fish and invertebrate species in the Northeast U.S. Shelf including exploited, forage, and protected species. We define climate vulnerability as the extent to which abundance or productivity of a species in the region could be impacted by climate change and decadal variability. We find that the overall climate vulnerability is high to very high for approximately half the species assessed; diadromous and benthic invertebrate species exhibit the greatest vulnerability. In addition, the majority of species included in the assessment have a high potential for a change in distribution in response to projected changes in climate. Negative effects of climate change are expected for approximately half of the species assessed, but some species are expected to be positively affected (e.g., increase in productivity or move into the region). These results will inform research and management activities related to understanding and adapting marine fisheries management and conservation to climate change and decadal variability.

## Introduction

Marine fish and invertebrate species are impacted by climate change and decadal variability. A classic example is the historical oscillation between Pacific Sardine and Northern Anchovy populations in the California Current, which occurred before recorded human exploitation began [1]. More recently, changes in marine species distribution and population productivity have been linked to changes in the climate [2–5]. Changes have also been documented in the distribution of fishery landings and potentially the distribution and magnitude of fishing effort [6, 7]. Although fishing remains an important, and in many cases, dominant driver of population abundance, there is now substantial evidence that climate change and decadal variability affect fish and invertebrate populations [8–10].

An increasing number of studies are linking population models to climate models and projecting the effect of future climate change on marine fish and invertebrate species [11–16]. These studies develop either a process-based or empirical relationship between climate variables and population parameters. Projections of the climate factor from climate models are then used to force the population model into the future [17]. In general, these studies show that climate change will continue to impact species and the ecosystem services they provide (e.g., fisheries, forage, [18]) for the foreseeable future (decades to centuries). For many regions, developing a mechanistic model for each species is not possible in the short-term because of the limited personnel and scientific resources, the lack of mechanistic models linking climate to population dynamics, and the large number of managed species. Global and regional species distribution models have been linked to climate projections to project changes in available habitat and resulting distribution shifts [19, 20]. These studies typically do not focus on providing species-specific information that can be used by regional fisheries managers.

Trait-based climate change vulnerability assessments provide another method to evaluate the potential risks to species posed by climate change [21–23]. In general, vulnerability assessments are a formal approach for identifying and prioritizing the vulnerabilities in a system [22, 23]. They often involve expert elicitation to estimate the general sensitivities of species to a stressor. The approach fills the need for broad, transparent, relatively quick evaluation of the vulnerability of multiple species. Vulnerability assessments have been used to evaluate the risk of overfishing for species within given regions [24, 25] and are increasingly being used to evaluate the vulnerabilities of marine species to climate change [26–31].

There are many forms, but in general, trait-based vulnerability assessments identify: i) environmental variables expected to change that could impact species (termed exposure factors) and ii) sensitivity attributes that predict a species intrinsic resilience to change [21, 22, 29, 32]. Some vulnerability assessments separate sensitivity into two components: adaptive capacity and sensitivity [33], but we choose to combine adaptive capacity attributes with sensitivity attributes [34]. Specifically for climate vulnerability, exposure factors include climate variables that have the potential to affect productivity or distribution of a species (or population) in a specific region. For example, temperature is a climate factor that affects species via multiple mechanisms from enzyme reactions to feeding rate to seasonal distribution [35]. Species sensitivity attributes include biological or ecological variables that predict the vulnerability to climate change. For example, a species with an inherently low maximum per capita population growth rate is more sensitive to changes in climate compared to species with an inherently high maximum per capita population growth rate. The exposure factors and sensitivity attributes are scored for each species based on a pre-defined scoring system. These scores are combined across exposure factors and sensitivity attributes to derive a relative species-specific climate vulnerability score. While these methods have limitations, the framework allows a diverse set of species to be assessed in a relatively short period of time, and provides a foundation for further research and management responses [21].

Our objective was to conduct a climate vulnerability assessment for fish and invertebrate species in the Northeast U.S. Continental Shelf Large Marine Ecosystem (hereafter Northeast U.S. Shelf) using the National Marine Fisheries Service (NMFS) Climate Vulnerability Assessment Methodology [34]. We use climate projections between 2005–2055 to evaluate climate change and decadal variability in the 20–40 year time frame. Separating anthropogenic climate change from natural decadal variability is difficult [36] and although we use climate change throughout most of this paper, it is important to recognize that the signals of both change and variability are included in the projections. We define vulnerability as a change in a species’ productivity and or abundance associated with a changing climate, including both climate change and decadal climate variability. We also evaluate the potential for a change in distribution and estimate the directional effect (positive or negative) of a changing climate on species in the Northeast U.S. Shelf. This ecosystem supports valuable commercial ($1.6 billion from landings in 2013 [37]) and recreational ($14.8 billion in total angler expenditures [38]) fisheries. The region is also experiencing relatively rapid climate change [39]. Numerous studies have linked recent climate change to changes in the region’s fish and invertebrate populations, including changes in productivity [4, 8] and distribution [40–43]. Of the numerous fishery species in the region, climate variables have only been directly incorporated into scientific advice and management in a few cases [44, 45]. Although this species-by-species approach is necessary, scientists and managers alike need a broad perspective within which to set research priorities and frame management decisions. Our purpose in conducting this vulnerability assessment is to provide such a system-wide perspective for the Northeast U.S. Shelf.

## Materials and Methods

**Fig 1:**
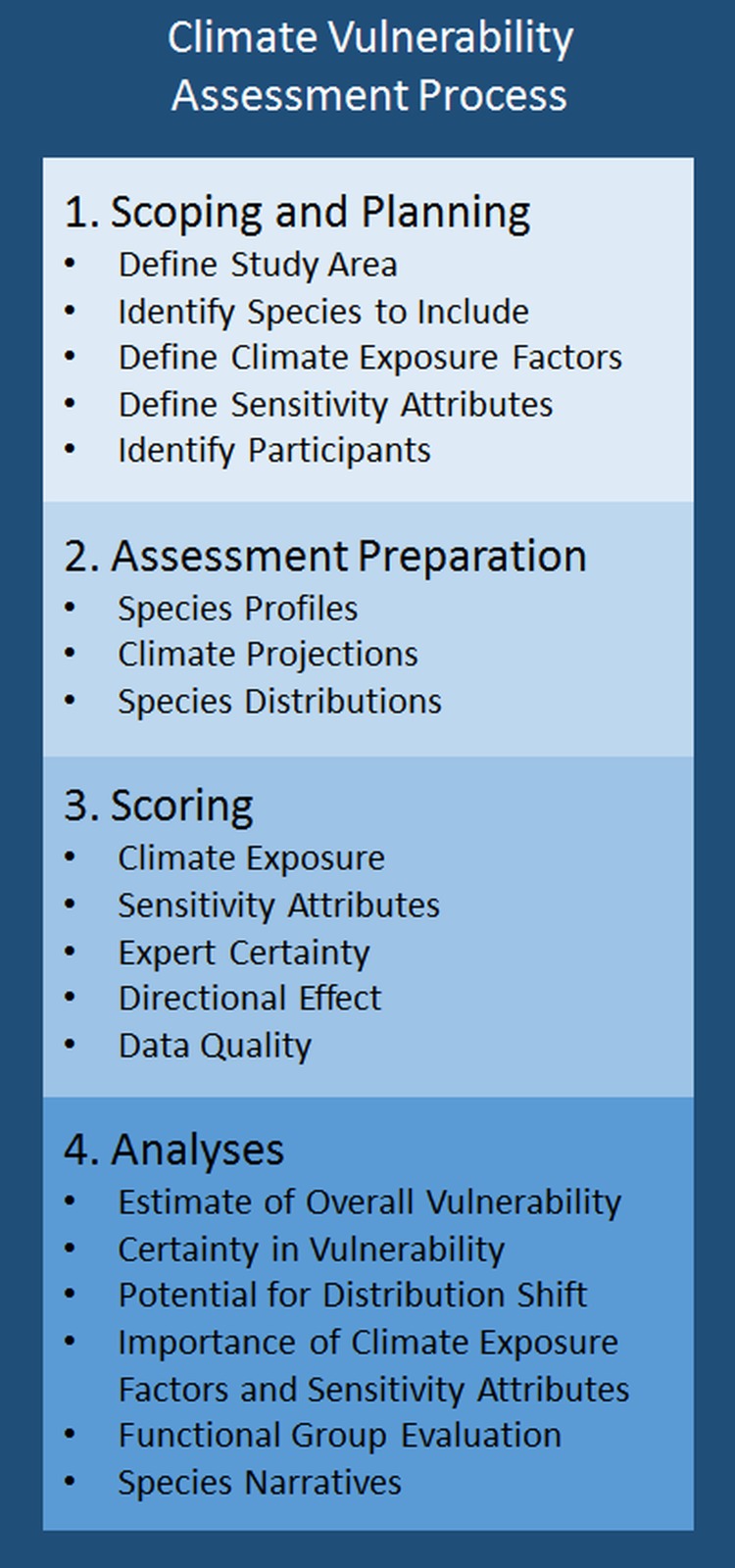
4 Steps used in the Northeast Fisheries Climate Vulnerability Assessment. For more details see the NMFS Climate Vulnerability Assessment Methodology [34].

The methods used here, and the development and rationale behind them, are fully described in the National Marine Fisheries Service (NMFS) Climate Vulnerability Assessment Methodology [34] (see Fig 1). The steps are: 1) scoping and planning the assessment including i) identifying the spatial region, ii) the species to include, iii) the climate variables to include as exposure factors, iv) the biological and ecological traits to include as sensitivity attributes, and v) recruiting experts to participate in the assessment; 2) preparation of materials for the assessment including i) consolidating available information on each species, ii) obtaining information on the future state of exposure factors, and iii) providing the spatial overlap between climate exposure and species distributions in the region; 3) expert scoring of the different components of the assessment including i) climate exposure scoring, ii) sensitivity attribute scoring, iii) quantifying expert certainty in scoring, iv) scoring the directional effect of climate change on a species in the region, and v) scoring the quality of data used in the assessment; and 4) analyses of the scores including i) estimating overall climate vulnerability, ii) estimating the potential for a distribution change using a subset of sensitivity attributes, iii) estimating certainty in overall climate vulnerability, potential for a distribution change, and the directional effect of climate change using bootstrapping; iv) identifying the importance of each exposure factor and sensitivity attribute to the overall climate vulnerability using a leave-one out sensitivity analysis, v) evaluating the results on a functional group basis, and vi) developing species specific narratives that summarize the results for each species.

### Scoping and Planning

#### Study Area

**Fig 2:**
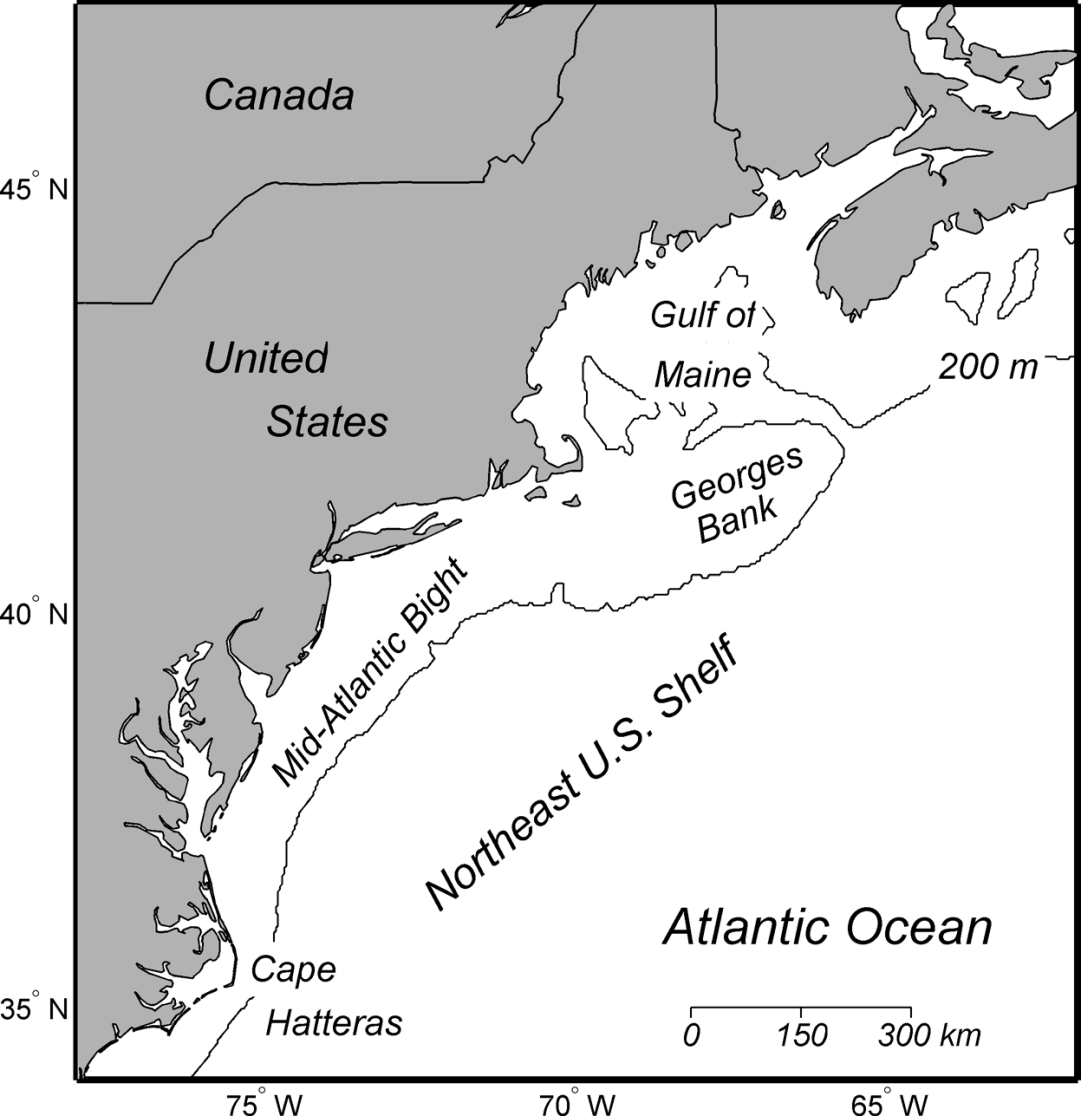
Map of Northeast U.S. Continental Shelf Large Marine Ecosystem.

The study area was the Northeast U.S. Shelf, which ranges from Cape Hatteras, North Carolina through the Gulf of Maine (Fig 2). The focus was on species that occur in marine waters of the Northeast U.S. Shelf, but a number of these species use freshwater, estuaries, and offshore areas during some portion of their life history [46]. Thus the study area included freshwater systems, as well as the shelf and oceanic waters.

#### Species Included

The focus of the vulnerability assessment was on marine fish and invertebrate species that commonly occur in the Northeast U.S. Shelf, including exploited species (e.g., Atlantic Cod, Atlantic Sea Scallop), protected species (e.g., Atlantic Salmon), and ecologically important species (e.g., Sand Lances). The exploited species included federally and state managed species, as well as species that are not currently managed but are harvested in the region. Federal commercial and recreational landings were evaluated in developing the list of species. Protected fish species were also assessed including fish species listed under the Endangered Species Act [47] and species considered as Species of Concern by NMFS [48]. Several ecologically important forage fish species were included based on species identified as forage by the Mid-Atlantic Fishery Management Council [49]. Highly migratory species were generally excluded because much of their life cycle is completed outside the study area. In total, 82 species were included and divided into 6 functional groups: Coastal Fish (n = 14), Diadromous Fish (n = 10), Elasmobranchs (n = 12), Groundfish (n = 19), Benthic Invertebrates (n = 18), and Pelagic Fish and Cephalopods (n = 9) (Table 1). These functional groups were based in part on phylogeny and in part on habitats occupied.

**Table 1:** Species included in the Northeast Fisheries Climate Vulnerability Assessment. Assigned functional group, common name, and scientific name of the 82 fish and invertebrate species included in the Northeast Fisheries Climate Vulnerability Assessment.

Group	Common Name	Scientific Name
Coastal Fish	Atlantic Croaker	*Micropogonias undulates*
Coastal Fish	Atlantic Menhaden	*Brevoortia tyrannus*
Coastal Fish	Black Sea Bass	*Centropristis striata*
Coastal Fish	Northern Kingfish	*Menticirrhus saxatilis*
Coastal Fish	Red Drum	*Sciaenops ocellatus*
Coastal Fish	Scup	*Stenotomus chrysops*
Coastal Fish	Spanish Mackerel	*Scomberomorus maculatus*
Coastal Fish	Spot	*Leiostomus xanthurus*
Coastal Fish	Spotted Seatrout	*Cynoscion nebulosus*
Coastal Fish	Striped Bass	*Morone saxatilis*
Coastal Fish	Summer Flounder	*Paralichthys dentatus*
Coastal Fish	Tautog	*Tautoga onitis*
Coastal Fish	Weakfish	*Cynoscion regalis*
Coastal Fish	Winter Flounder	*Pseudopleuronectes americanus*
Diadromous Fish	Alewife	*Alosa pseudoharengus*
Diadromous Fish	Conger Eel	*Anguilla oceanica*
Diadromous Fish	American Eel	*Anguilla rostrata*
Diadromous Fish	American Shad	*Alosa sapidissima*
Diadromous Fish	Atlantic Salmon	*Salmo salar*
Diadromous Fish	Atlantic Sturgeon	*Acipenser oxyrhynchus*
Diadromous Fish	Blueback Herring	*Alosa aestivalis*
Diadromous Fish	Hickory Shad	*Alosa mediocris*
Diadromous Fish	Rainbow Smelt	*Osmerus mordax*
Diadromous Fish	Shortnose Sturgeon	*Acipenser brevirostrum*
Elasmobranchs	Barndoor Skate	*Dipturus laevis*
Elasmobranchs	Clearnose Skate	*Raja eglanteria*
Elasmobranchs	Dusky Shark	*Carcharhinus obscurus*
Elasmobranchs	Little Skate	*Leucoraja erinacea*
Elasmobranchs	Porbeagle	*Lamna nasus*
Elasmobranchs	Rosette Skate	*Leucoraja garmani*
Elasmobranchs	Sand Tiger	*Carcharias taurus*
Elasmobranchs	Smooth Dogfish	*Mustelus canis*
Elasmobranchs	Smooth Skate	*Malacoraja senta*
Elasmobranchs	Spiny Dogfish	*Squalus acanthias*
Elasmobranchs	Thorny Skate	*Amblyraja radiata*
Elasmobranchs	Winter Skate	*Leucoraja ocellata*
Groundfish	Acadian Redfish	*Sebastes fasciatus*
Groundfish	American Plaice	*Hippoglossoides platessoides*
Groundfish	Atlantic Cod	*Gadus morhua*
Groundfish	Atlantic Hagfish	*Myxine glutinosa*
Groundfish	Atlantic Halibut	*Hippoglossus hippoglossus*
Groundfish	Atlantic Wolffish	*Anarhichas lupus*
Groundfish	Cusk	*Brosme brosme*
Groundfish	Haddock	*Melanogrammus aeglefinus*
Groundfish	Monkfish (Goosefish)	*Lophius americanus*
Groundfish	Ocean Pout	*Zoarces americanus*
Groundfish	Offshore Hake	*Merluccius albidus*
Groundfish	Pollock	*Pollachius virens*
Groundfish	Red Hake	*Urophycis chuss*
Groundfish	Silver Hake	*Merluccius bilinearis*
Groundfish	Tilefish	*Lopholatilus chamaeleonticeps*
Groundfish	White Hake	*Urophycis tenuis*
Groundfish	Windowpane	*Scophthalmus aquosus*
Groundfish	Witch Flounder	*Glyptocephalus cynoglossus*
Groundfish	Yellowtail Flounder	*Limanda ferruginea*
Pelagic Fish and Cephalopods	Anchovies	*Anchoa hepsetus / Anchoa mitchilli*
Pelagic Fish and Cephalopods	Atlantic Herring	*Clupea harengus*
Pelagic Fish and Cephalopods	Atlantic Mackerel	*Scomber scombrus*
Pelagic Fish and Cephalopods	Atlantic Saury	*Scomberesox saurus*
Pelagic Fish and Cephalopods	Bluefish	*Pomatomus saltatrix*
Pelagic Fish and Cephalopods	Butterfish	*Peprilus triacanthus*
Pelagic Fish and Cephalopods	Longfin Inshore Squid	*Doryteuthis pealeii*
Pelagic Fish and Cephalopods	Sand Lances	*Ammodytes americanus & Ammodytes dubius*
Pelagic Fish and Cephalopods	Northern Shortfin Squid	*Illex illecebrosus*
Benthic Invertebrates	American Lobster	*Homarus americanus*
Benthic Invertebrates	Atlantic Sea Scallop	*Placopecten magellanicus*
Benthic Invertebrates	Atlantic Surfclam	*Spisula solidissima*
Benthic Invertebrates	Bay Scallop	*Argopecten irradians*
Benthic Invertebrates	Bloodworm	*Glycera dibranchiata*
Benthic Invertebrates	Blue Crab	*Callinectes sapidus*
Benthic Invertebrates	Blue Mussel	*Mytilus edulis*
Benthic Invertebrates	Cancer Crabs	*Cancer borealis / Cancer irroratus*
Benthic Invertebrates	Channeled Whelk	*Busycotypus canaliculatus*
Benthic Invertebrates	Deep-sea Red Crab	*Chaceon quinquedens*
Benthic Invertebrates	Eastern Oyster	*Crassostrea virginica*
Benthic Invertebrates	Green Sea Urchin	*Strongylocentrotus droebachiensis*
Benthic Invertebrates	Horseshoe Crab	*Limulus polyphemus*
Benthic Invertebrates	Knobbed Whelk	*Busycon carica*
Benthic Invertebrates	Northern Shrimp	*Pandalus borealis*
Benthic Invertebrates	Ocean Quahog	*Arctica islandica*
Benthic Invertebrates	Northern Quahog	*Mercenaria mercenaria*
Benthic Invertebrates	Softshell Clam	*Mya arenaria*

#### Climate Exposure Factors

Exposure is a measure of the projected magnitude of change in the physical environment due to climate [23, 29]. Exposure factors are those climate variables included in the assessment that could impact a species (e.g., temperature, salinity). The exposure score includes information about the magnitude of the expected climate change, but not in relation to each species’ tolerances, which are often unknown. Exposure factors were chosen based on two criteria. First, factors were chosen on the basis that studies have found an effect on fish and invertebrate species in the Northeast U.S. Shelf. Second, factors were chosen that are likely to be well represented in the current class of global climate models (models are described below) [17]. Seven factors were selected: ocean surface temperature (upper 10 m), ocean surface salinity (upper 10 m), surface air temperature, precipitation, surface pH (upper 10 m), currents, and sea-level rise. Bottom estimates were not used owing to the low spatial resolution of current climate models and the fact that most of the models used do not resolve the bathymetry of the Northeast U.S. Shelf. Similarly, primary productivity was not used because of the importance of regional-scale oceanography and estuaries, neither of which are resolved in the current ensemble of current global climate models. All factors were equally weighted owing to the limited knowledge regarding the magnitude of effects and species responses to climate. Changes in the mean and variance of ocean temperature, ocean salinity, air temperature, precipitation, and pH were included, whereas only changes in mean sea level and ocean currents were considered resulting in a total of 12 climate exposure factors (Table 2). Ocean temperature is an important climate factor, which numerous studies have linked to changes in distribution and productivity [4, 41, 42, 50]. Fewer studies have linked changes in ocean salinity to biological responses, but changes in salinity may increase metabolic costs [51], reducing resilience to other changes. Salinity also affects stratification, mixing, and thus the timing and magnitude of primary production [52]. Most climate models are at a scale where water temperatures in estuaries and freshwater areas are not resolved, so air temperature is used as a proxy [14, 53], as it is directly linked to the temperature in shallow water owing to air-water heat exchange [4]. Air temperature and surface ocean temperature are likely correlated because of the large-scale of climate induced warming, but the two factors are important and distinct in terms of their impact on the biology of some species (e.g., Atlantic Salmon are exposed to climate impacts both in freshwater and marine habitats). Streamflow is linked to productivity of a number of diadromous species [54, 55]. Most climate models do not simulate streamflow, rather they have river routing systems that move water among model grid cells. Precipitation is therefore used here as a proxy of the amount of water in streams and rivers. Numerous studies have found an effect of increased dissolved CO_2_ (ocean acidification) on marine organisms from larval survival in molluscs to olfaction in fish [56, 57]. Estimates of pH were derived from Earth Systems Models, which simulate the carbon system to varying degrees of complexity. Ocean currents were also used as a factor since most marine organisms have planktonic early life stages that rely on advection for transport to habitats necessary for the continuation of the life cycle [58]. Small-scale changes in currents cannot be assessed from the current global climate models, but changes in large scale changes can be considered. Sea-level rise threatens a variety of coastal habitats including marshes, seagrass beds, and beaches [59, 60]. This threat is exacerbated by the large degree of coastal development in the Northeast U.S. Shelf [61] and the large reliance of fish and invertebrate species on coastal habitats during portions of their life history [46].

**Table 2:** Climate Exposure Factors and Sensitivity Attributes. List of climate exposure factors and sensitivity attributes used in the climate vulnerability assessment. See NMFS Climate Vulnerability Assessment Methodology for more details [34].

Climate Factor or Biological Attribute		Goal	Low Score	High Score
Climate Factors				
	Mean Ocean Surface Temperature	To determine if there are changes in mean ocean surface temperature comparing the 1956–2005 to 2006–2055 periods	Low magnitude of change	High magnitude of change
	Mean Ocean Surface Salinity	To determine if there are changes in mean ocean surface salinity comparing the 1956–2005 to 2006–2055 periods	Low magnitude of change	High magnitude of change
	Mean Air Temperature	To determine if there are changes in mean air temperature comparing the 1956–2005 to 2006–2055 periods. Air temperature is a proxy for water temperatures in lakes, streams, river, estuaries, and nearshore areas	Low magnitude of change	High magnitude of change
	Mean Precipitation	To determine if there are changes in mean precipitation comparing the 1956–2005 to 2006–2055 periods. Precipitation is a proxy for streamflow.	Low magnitude of change	High magnitude of change
	Mean Ocean pH	To determine if there are changes in mean ocean pH comparing the 1956–2005 to 2006–2055 periods. pH represents ocean acidification.	Low magnitude of change	High magnitude of change
	Variability in Ocean Surface Temperature	To determine if there are changes in variability of ocean surface temperature comparing the 1956–2005 to 2006–2055 periods	Low magnitude of change	High magnitude of change
	Variability in Ocean Surface Salinity	To determine if there are changes in variability of ocean surface salinity comparing the 1956–2005 to 2006–2055 periods	Low magnitude of change	High magnitude of change
	Variability in Air Temperature	To determine if there are changes in variability of air temperature comparing the 1956–2005 to 2006–2055 periods. Air temperature is a proxy for water temperatures in lakes, streams, river, estuaries, and nearshore areas	Low magnitude of change	High magnitude of change
	Variability in Precipitation	To determine if there are changes in variability of precipitation comparing the 1956–2005 to 2006–2055 periods. Precipitation is a proxy for streamflow.	Low magnitude of change	High magnitude of change
	Variability in pH	To determine if there are changes in variability of ocean pH comparing the 1956–2005 to 2006–2055 periods. pH represents ocean acidification.	Low magnitude of change	High magnitude of change
	Sea Level Rise	To evaluate the magnitude of sea level rise relative to the ability of nearshore habitats to change	Low magnitude of change	High magnitude of change
	Ocean Currents	To evaluate changes in large-scale circulation.	Low magnitude of change	High magnitude of change
Biological Attributes				
	Prey Specificity	To determine, on a relative scale, if the stock is a prey generalist or a prey specialist.	Prey generalist	Prey specialist
	Habitat Specificity	To determine, on a relative scale, if the stock is a habitat generalist or a habitat specialist while incorporating information on the type and abundance of key habitats.	Habitat generalist	Habitat specialist
	Sensitivity to Ocean Acidification	To estimate a stock’s sensitivity to ocean acidification based on its relationship with “shelled species.” (followed Kroeker et al. 2012)	Sensitive taxa	Insensitive taxa
	Complexity in Reproductive Strategy	To determine how complex the stock’s reproductive strategy is and how dependent reproductive success is on specific environmental conditions.	Low compleixty, broadcast spawning	High complexity; aggregate spawning
	Sensitivity to Temperature	To use the distribution of the species as a proxy for its sensitivity to temperature. Note: that this attribute uses species (vs. stock) distributions as they better predict thermal requirements.	Broad thermal limits	Narrow thermal limits
	Early Life History Survival and Settlement Requirements	To determine the relative importance of early life history requirements for a stock.	Generalist with few requirements	Specialists with specific requirements
	Stock Size/Status	To estimate stock status to clarify how much stress from fishing the stock is experiencing and to determine if the stock’s resilience or adaptive capacity are compromised due to low abundance.	High abundance	Low abundance
	Other Stressors	To account for conditions that could increase the stress on a stock and thus decrease its ability to respond to changes.	Low level of other stressors	High level of other stressors
	Population Growth Rate	To estimate the relative productivity of the stock.	High population growth	Low population growth
	Dispersal of Early Life Stages	To estimate the ability of the stock to colonize new habitats when/if their current habitat becomes less suitable.	High dispersal	Low dispersal
	Adult Mobility	To estimate the ability of the stock to move to a new location if their current location changes and is no longer favorable for growth and/or survival.	High mobility	Low mobility
	Spawning Cycle	To determine if the duration of the spawning cycle for the stock could limit the ability of the stock to successfully reproduce if necessary conditions are disrupted by climate change.	Year-round spawning	One event per year

#### Sensitivity Attributes

Sensitivity attributes represent biological traits that are indicative of an ability or inability of a species to respond to environmental change. All 12 attributes defined in the NMFS Climate Vulnerability Assessment Methodology [34] were used here (Table 2). As an example, the Adult Mobility of a clam is low and as a result, adults would be unable to move as climate changes making them more vulnerable to climate change. The definition for a few sensitivity attributes varies slightly from those presented in the NMFS Methodology [34] (see S1 Supporting Information) due to minor changes in the definitions in the methodology, which occurred after the implementation described here.

#### Participants

The expert group consisted of the core development team for the methodology [34], as well as regional experts from NOAA NMFS including stock assessment scientists, fisheries scientists, ecologists, and oceanographers. Fourteen experts participated: two experts per functional group (Coastal Fish, Benthic Invertebrates, Pelagic Fish and Cephalopods, Elasmobranchs, and Diadromous Fish), with the exception of 4 experts for Groundfish. Most experts have experience with species in several functional groups. Two climate experts also participated, providing access and advice regarding the climate exposure factors. Representatives of the New England Fisheries Management Council, Mid-Atlantic Fisheries Management Council and Greater Atlantic Regional Fisheries Office provided input on species at a workshop where the experts met to discuss exposure and sensitivity scoring (see expert certainty below).

### Assessment Preparation

#### Species Profiles

Species profiles were prepared to summarize the biological and ecological information needed for experts to score the sensitivity attributes. The consolidation of the information was an important step to ensure that all experts were provided with the same baseline information. In general, the Northeast U.S. Shelf is data rich with regards to fish and invertebrate biology and ecology. However, there are species included in this assessment that are considered data-poor and a Data Quality attribute was included to help identify information gaps. Numerous summary documents were available to complete species profiles including stock assessments [62, 63], Essential Fish Habitat source documents [64], and monographs that describe the biology and ecology of fish and invertebrate species in the region [46, 65, 66]. Peer-reviewed literature was also used to complete the profiles for some species when information was not summarized in the documents described above. General guidelines were developed for completing species profiles and approximately 3–6 hours were spent on each species profile. One scientist then reviewed all the profiles to ensure consistency in content.

#### Climate Projections

For most climate factors included in the assessment, exposure was estimated from an ensemble of global climate models used in the Intergovernmental Panel on Climate Change Assessment Report 5 (IPCC AR5). As described above, the choice of climate factors to use in this assessment was impacted by the resolution of the models used for projections. The ocean resolution of these models is too course (0.5–1.0° latitude, 1.0–1.5° longitude) to allow mesoscale eddies, sub-mesoscale eddies, fronts, and regional scale bathymetry and circulation [67]. The Representative Concentration Pathway 8.5 (RCP 8.5) was used, which represents a “business-as-usual” scenario assuming little to no stabilization of greenhouse gas emissions by 2100 [68, 69]. The 2006–2055 period was chosen for projections because this represents the coming decades, which are of more relevance to living marine resource management than the end of the century. The 50 year average focuses the projections on the forced climate change signal, but also includes the multi-decadal variability signal [17, 36]. The exposures to ocean surface temperature (upper 10 m), surface air temperature, ocean surface salinity (upper 10 m), and precipitation were estimated from an ensemble of 25–35 global climate models. Exposure to ocean acidification was estimated from an ensemble of 11 earth system models. These ensembles were defined as those models available on the Earth System Grid Federation Portal (http://pcmdi9.llnl.gov/esgf-web-fe/) in autumn 2013 that simulated a given parameter over the time period of interest.

Using these model outputs, the change in climate conditions in the future relative to the past were expressed in a standard deviate framework (for the mean factors) and an F-test framework (for the variance factors). Maps of these standard deviates and variance ratios were obtained from NOAA’s Ocean Climate Change Web Portal [70]. Maps of inter-model variability were also obtained to assess the among-model uncertainty. The approach scales projected future change to the past mean state and variability in that state (i.e., the standard deviate of change is used, not the mean change). For example, the exposure resulting from 1°C increase is greater in an ecosystem with a 0.2°C standard deviation in past temperatures compared to a system with a 2°C standard deviation in past temperatures. This construct is based on macroecological principals that indicate niches are broader in areas of more variable environment [71, 72].

Exposure to change in currents and sea-level rise was evaluated by a review of the literature. A summary of likely changes in currents was prepared in consultation with oceanographers and climate scientists (S2 Supporting Information). Exposure to a change in currents was scored based on a species use or dependence of large-scale currents in the region. For example American Eel is dependent on the Gulf Stream to transport larvae from spawning locations in the Sargasso Sea to the oceanic waters off the Northeast U.S. Shelf. Thus American Eel is exposed to changes in the Gulf Stream. Similarly for Sea-Level Rise, a summary was prepared of the expected rate of change of regional sea level compared to rates of sea level rise that coastal habitats can accommodate (S3 Supporting Information). Exposure to sea-level rise was scored based on a species’ reliance on wetland, seagrass, and estuarine habitats.

#### Species distributions

Species distribution information was obtained primarily through the Ocean Biogeographic Information System (OBIS) [73]. OBIS data is point occurrence data only, however, the Northeast U.S. Shelf is well represented in OBIS through the inclusion of data from several ecosystem-wide surveys. For some species, information obtained through OBIS was supplemented with information from other sources including stock assessments [62, 63], Essential Fish Habitat source documents [64], and monographs [46, 65, 66]. Owing to the multiple sources of information used for species distribution, a quantitative match between exposure and distribution was not calculated and experts were allowed to judge spatial overlap individually using the information provided. The uncertainty introduced by expert elicitation is small since the Northeast U.S. Shelf is well sampled and species distributions are relatively well known [41, 42].

### Scoring

#### Climate Exposure

Four scientists scored climate exposure in consultation with a broader group of climate scientists. Prior to scoring, the experts received an overview of the output of the climate model ensembles [70]. Experts then used information on species distribution and the exposure maps of ocean surface temperature, ocean surface salinity, air temperature, and pH to estimate exposure in the Northeast U.S. Shelf ecosystem. The comparisons between species distribution and exposure maps were made visually by each expert independently. This species specific exposure was then converted to a score using pre-defined criteria (S4 Supporting Information). Scores for exposure were: low, moderate, high, and very high. If a species was not exposed to a given factor, it was scored as low exposure (e.g., Acadian Redfish is not exposed to changes in precipitation because they live in deep, offshore waters). For sea-level rise and ocean currents, which were not included in the climate model output, the experts reviewed the exposure summaries (S2 Supporting Information, S3 Supporting Information) and then discussed these documents as a group. Species with no exposure to change in currents were scored as low and species with exposure to currents were scored on the basis of the magnitude of change estimated by expert opinion (clarified in S2 Supporting Information). With regard to Sea-Level Rise, species with no reliance on nearshore habitats were scored low and species that use these habitats were scored based on the magnitude of the impacts of sea-level rise estimated by expert opinion (clarified in S3 Supporting Information).

#### Sensitivity Attributes

Fourteen experts scored sensitivity attributes. Benthic Invertebrate, Coastal Fish, Pelagic Fish and Cephalopods, Elasmobranchs, and Diadromous Fish group experts scored all the species in their assigned group and a random selection of other species. Owing to the number of Groundfish species, assigned experts scored half the Groundfish species and a random selection of other species. Experts used the species profiles and attribute descriptions (S1 Supporting Information) as a common baseline, but were encouraged to bring their expertise and new information to the scoring process.

#### Expert Certainty

Scoring of both climate exposure and biological sensitivity was completed individually using a 5 tally scoring system. Each expert had 5 tallies to score each exposure factor or sensitivity attribute; they could place all five tallies in the same bin (e.g., low) for attributes or factors with high certainty, or they could spread their tallies across all bins for attributes or factors with less certainty (e.g., low, moderate, high, very high). Once the individual scores were recorded, experts met in person and discussed scores as a group. Experts were given the opportunity to independently change their final scores based on the discussion, however, there was no requirement nor expectation that experts reach consensus. The full results of the expert scoring- number of tallies per scoring bin (low, moderate, high, very high) by species and attribute are provided in S5 Supporting Information, S1 Dataset, and S2 Dataset.

#### Directional Effect

Experts were asked to score the directional effect of climate change for each species, giving an overall indication whether impacts are anticipated to be negative, neutral, or positive on the species in the region. For each species, 3 experts scored directional effect. Experts included the functional group experts for each species (n = 2) and the lead author. Each expert was given 4 tallies to score in the 3 bins. The scores were converted to numbers (negative = -1, neutral = 0, positive = 1) and a weighted average was calculated based on the total of 12 tallies. Weighted averages below -0.333 were classified as an overall negative effect, weighted averages between -0.333 and 0.333 were classified as an overall neutral effect, and weighted averages above 0.333 were classified as an overall positive effect. The full results of the expert scoring, number of tallies per scoring bin (Negative, Neutral, Positive) by species are provided in S5 Supporting Information (and S1 Dataset and S2 Dataset).

#### Data Quality

Experts also assessed the quality of information available for scoring (i.e., data quality, S6 Supporting Information). Each expert noted their opinion of data quality for each sensitivity attribute or exposure factor for each species. These data quality scores were then averaged across experts and the proportion of data quality scores <2 for each species was calculated; a score of 2 represents limited data available.

### Analyses

Five analyses were conducted on the expert scores of climate exposure, sensitivity attributes, and directional effect. 1) Overall climate vulnerability was calculated from a combination of climate exposure factors and biological sensitivity attributes. 2) Potential for a change in species distribution was calculated using a subset of sensitivity attributes. 3) Bootstrap analyses were used to evaluate the certainty in the overall vulnerability, potential for a change in distribution, and directional effect of climate change. 4) Leave-one-out sensitivity analyses were used to evaluate the effects exposure factors and sensitivity attributes in determining overall vulnerability. 5) Overall climate vulnerability, potential for distribution change, and directional effect of climate change were analyzed by functional groups to identify larger patterns in climate effects.

#### Estimate of Overall Vulnerability

Overall climate vulnerability was estimated using a four step process. First, the component scores (low, moderate, high and very high) were assigned a numerical value (1, 2, 3, and 4). Second, an average score for each climate exposure factor and sensitivity attribute was calculated as the weighted-mean of the experts’ tallies. Third, an overall exposure and sensitivity score was calculated from the weighted means using a logic rule (Table 3). Fourth, an overall climate vulnerability score was calculated by multiplying the overall exposure and sensitivity scores. The product of the two component numeric scores results in a value between 1 and 16. The overall climate vulnerability rank is then classified as follows: 1–3 low, 4–6 moderate, 8–9 high, and 12–16 very high.

**Table 3:** Logic rule for calculating overall species’ climate exposure and biological sensitivity. The scoring rubric is based on a logic model where a certain number of individual scores above a certain threshold are used to determine the overall climate exposure and overall biological sensitivity.

Overall Sensitivity or Exposure Score	Numeric Score	Logic Rule
Very High	4	3 of more attributes or factors mean ≥ 3.5
High	3	2 of more attributes or factors mean ≥ 3.0
Moderate	2	2 of more attributes or factors mean ≥ 2.5
Low	1	All other scores

#### Potential for Distribution Change

High potential for change in species distribution was defined as highly mobile adults, broadly dispersing early life stages, low habitat specificity, and high temperature sensitivity [34]. These attributes have some skill in predicting species that can change distribution in response to climate change; increased dispersal capacity and ecological generalism promote changes in distribution [74]. The potential for a change in species distribution was estimated using these attributes, reversing the scores for Adult Mobility, Early Life Stage Dispersal, and Habitat Specificity, and applying the same logic rule as in the general vulnerability calculation.

#### Certainty in Vulnerability Scores

Bootstrap analysis was used to calculate the certainty of the climate vulnerability scores, potential for change in distribution scores and directional effect scores. Using the climate vulnerability scores as the examples, the scores across all experts for a given exposure factor (n = 20; 4 experts and 5 tallies) or sensitivity attribute (n = 25; 5 experts and 5 tallies) were drawn randomly with replacement. This was repeated 10,000 times for each of the 12 sensitivity attributes and the 12 exposure factors, and the overall vulnerability score was calculated for each iteration. The outcomes of each iteration was recorded and the proportion of these 10,000 repetitions that scored in each overall vulnerability bin was enumerated. A similar bootstrapping analysis was conducted on the potential for a distribution change (n = 25; 5 experts and 5 tallies) and the directional effect scoring (n = 12; 3 experts and 4 tallies).

#### Importance of Climate Exposure Factors and Sensitivity Attributes

For the sensitivity analysis, the overall vulnerability score for each species was calculated leaving out the scores for each sensitivity attribute or exposure factor. These analyses were then evaluated across species to determine influential factors and attributes in the overall vulnerability rank.

#### Functional Group Evaluation

To evaluate the similarity of vulnerability across functional groups, overall climate vulnerability, potential for distribution change, and directional effect of climate change were pooled by functional group. In addition, sensitivity attribute scores within and among functional groups was evaluated using non-metric multidimensional scaling.

### Species Narratives

In addition to the composite results that show the relative vulnerabilities across species, species specific vulnerability narratives were prepared (S7 Supporting Information). These narratives provide the distribution of tallies and the data quality score for each exposure factor and sensitivity attribute, the overall climate vulnerability, potential for distribution change, directional effect scores, and the certainty in these scores. Additionally, a summary of identified climate effects on the species and a synopsis of the life history is given.

### Ethics Statement

This study was not based on Human Subject Research. The study was not based in Animal Research. No field permits were involved. This was an expert opinion vulnerability assessment and all experts involved are co-authors on the paper.

## Results

### Overall Climate Vulnerability

**Fig 3:**
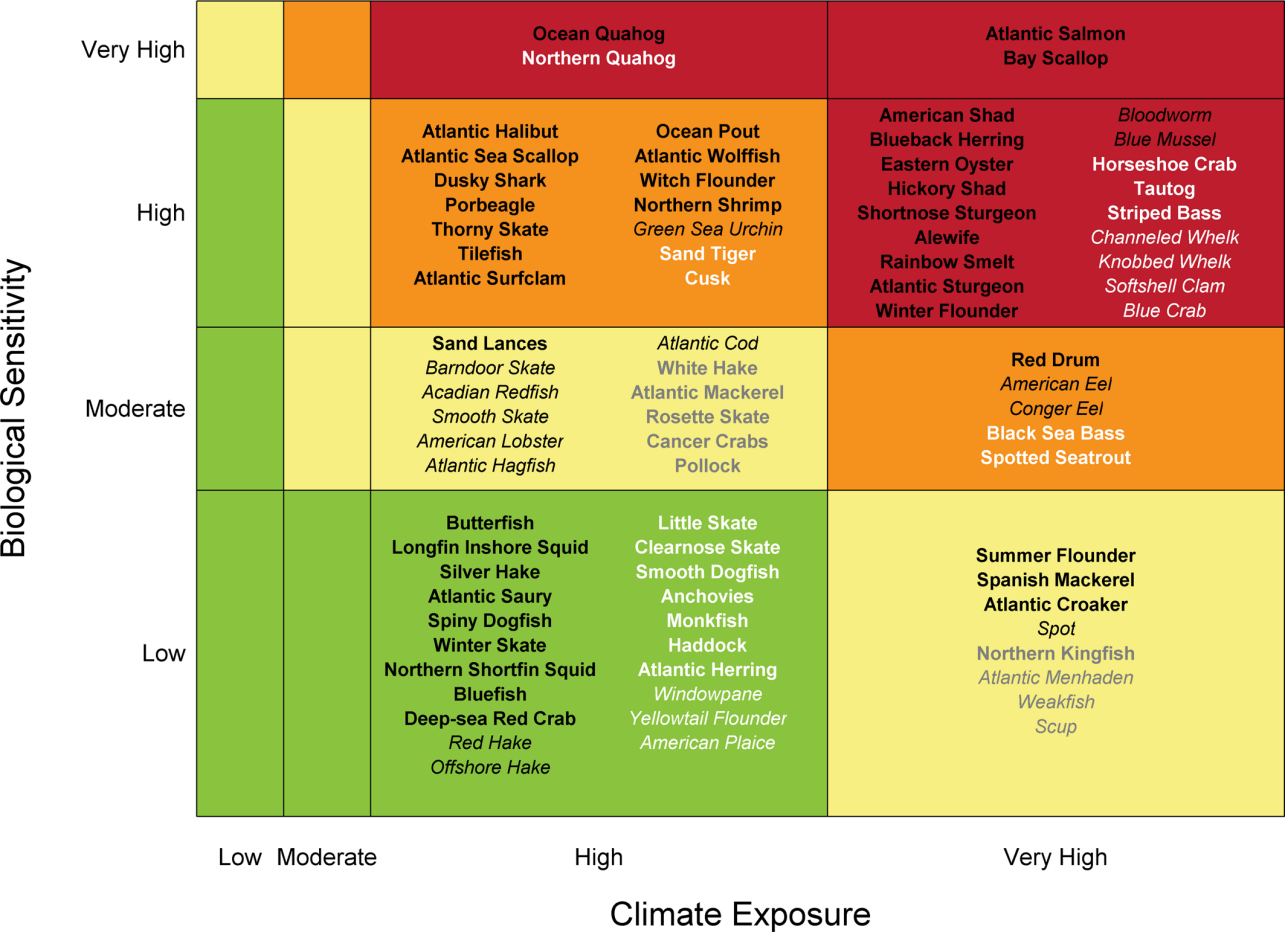
Overall climate vulnerability score. For species names and functional groups see Table 1. Overall climate vulnerability is denoted by color: low (green), moderate (yellow), high (orange), and very high (red). Certainty in score is denoted by text font and text color: very high certainty (>95%, black, bold font), high certainty (90–95%, black, italic font), moderate certainty (66–90%, white or gray, bold font), low certainty (<66%, white or gray, italic font).

The 82 species were nearly equally split among the different climate vulnerability ranks: very high (~27%), high (~23%), moderate (~24%) and low (~26%) (Fig 3). Climate exposure scores for all 82 species were high or very high indicating the magnitude of climate change relative to the variability of past conditions is high. Biological sensitivity ranged from low to very high. The certainty in the score of the majority of species exceeded 90% based on the bootstrap analysis (Fig 3). Approximately 27% of species had certainty scores between 66–90%. Approximately 12% of species had certainty scores <66%. For certainty scores less than 50%, a majority of bootstrapped climate vulnerability scores were different than the actual score. Species narratives provide species-specific summaries of the results (S7 Supporting Information).

### Potential for Distribution Change

**Fig 4:**
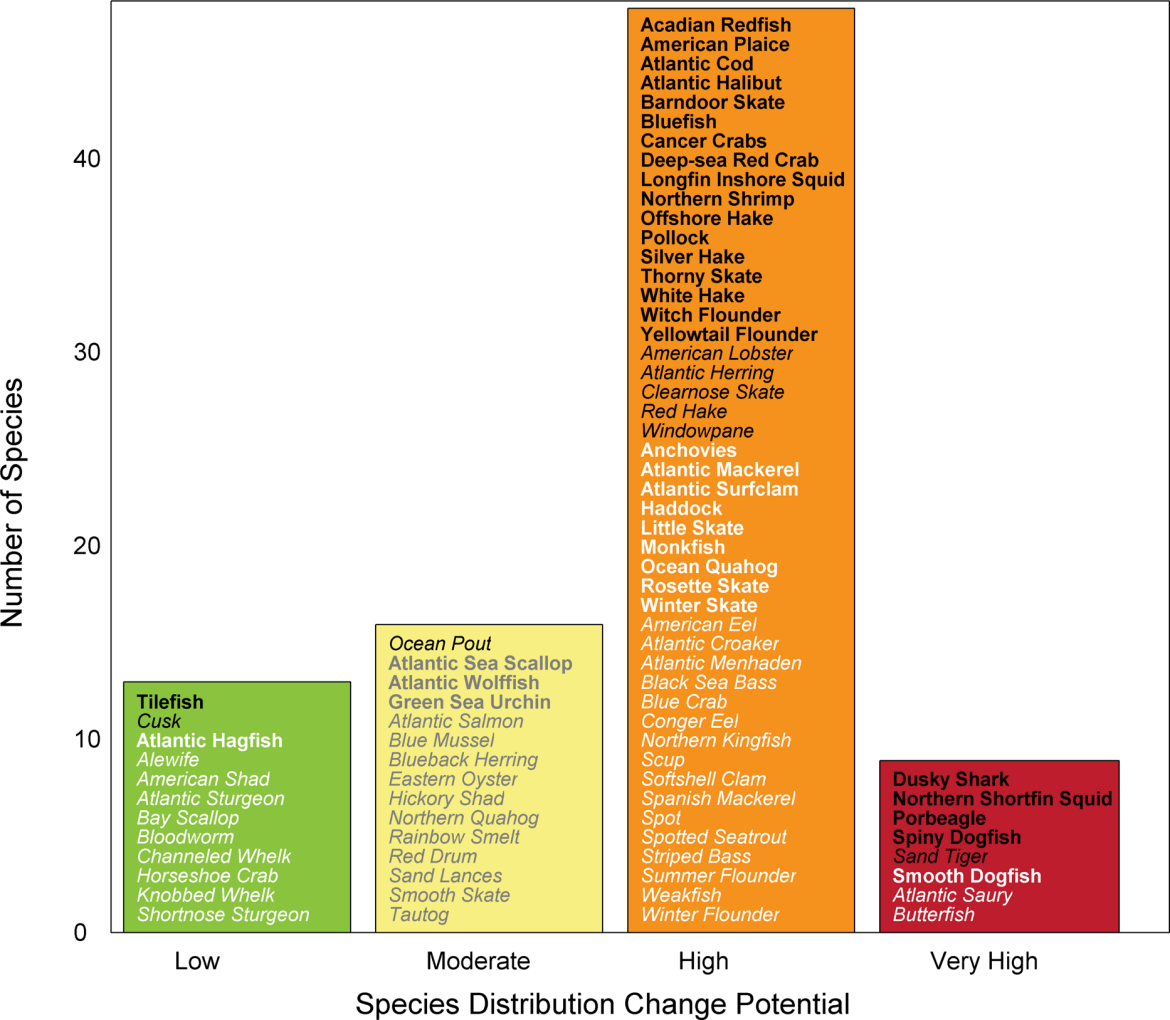
Potential for a change in species distribution. Potential was calculated using a subset of sensitivity attributes. Colors represent low (green), moderate (yellow), high (orange) and very high (red) potential for a change in distribution. Certainty in score is denoted by text font and text color: very high certainty (>95%, black, bold font), high certainty (90–95%, black, italic font), moderate certainty (66–90%, white or gray, bold font), low certainty (<66%, white or gray, italic font).

Many species in the Northeast U.S. Shelf have life history attributes that suggest distribution may change in response to climate change (Fig 4). More than 50% of the species exhibit very high or high potential for a change in species distribution. In general, overall climate vulnerability (changes in population productivity) varies inversely to the potential for a change in species distribution: species highly vulnerable to a change in productivity have a lower potential to change distribution and vice versa (S8 Supporting Information). However, the certainty potential for distribution change score of almost half the species was <66%; this lower value of certainty compared to overall climate vulnerability results from the lower number of attributes included in the potential for distribution change calculation.

### Directional Effect of Climate Change

**Fig 5:**
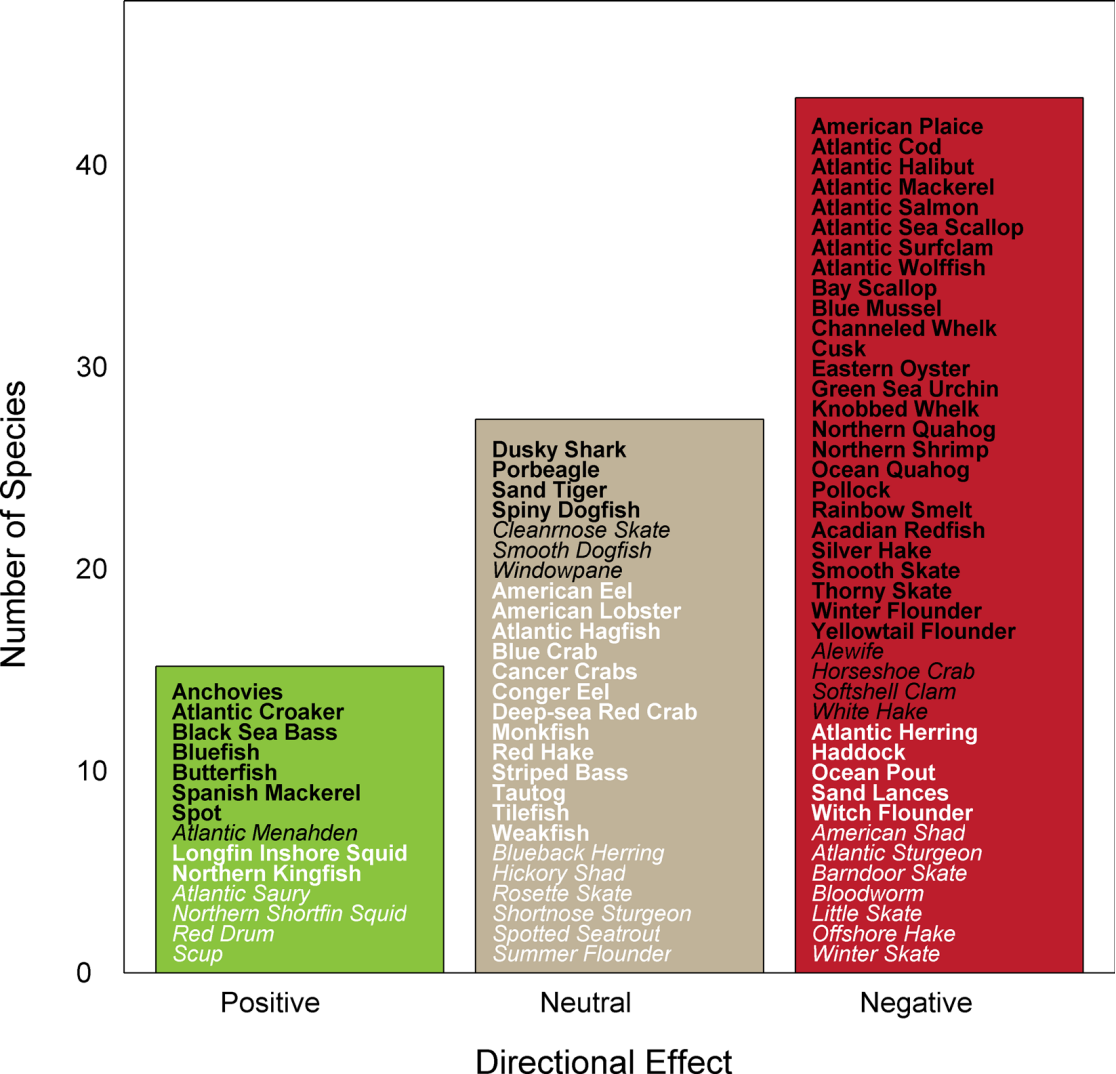
Directional effect of climate change. Colors represent expected negative (red), neutral (tan), and positive (green) effects. Certainty in score is denoted by text font and text color: very high certainty (>95%, black, bold font), high certainty (90–95%, black, italic font), moderate certainty (66–90%, white or gray, bold font), low certainty (<66%, white or gray, italic font).

Based on expert opinion of the directional effect of climate change, approximately half of the species were assessed to be negatively affected by climate change in the Northeast U.S. Shelf (Fig 5). Negative impacts are estimated for many of the iconic species in the ecosystem including Atlantic Sea Scallop, Atlantic Cod, and Atlantic Mackerel. In general, negative effects are anticipated for a number of Benthic Invertebrate and Groundfish species. However, positive effects are anticipated for 17% of species including Inshore Longfin Squid, Butterfish, and Atlantic Croaker. The certainty in directional effect score varied, but was >95% for almost half of the species assessed. However, there were species with low certainty (<66%) in all three directional effect categories. By comparing across the climate vulnerability, potential for distribution change, and directional effect scores, species can be identified that are likely to increase in productivity (e.g., Black Sea Bass) or shift into the region (e.g., Atlantic Croaker) or that are likely to decrease in productivity (e.g., Winter Flounder) or shift out of the region (e.g., Atlantic Mackerel).

### Evaluation of Exposure Factors and Sensitivity Attributes

**Fig 6:**
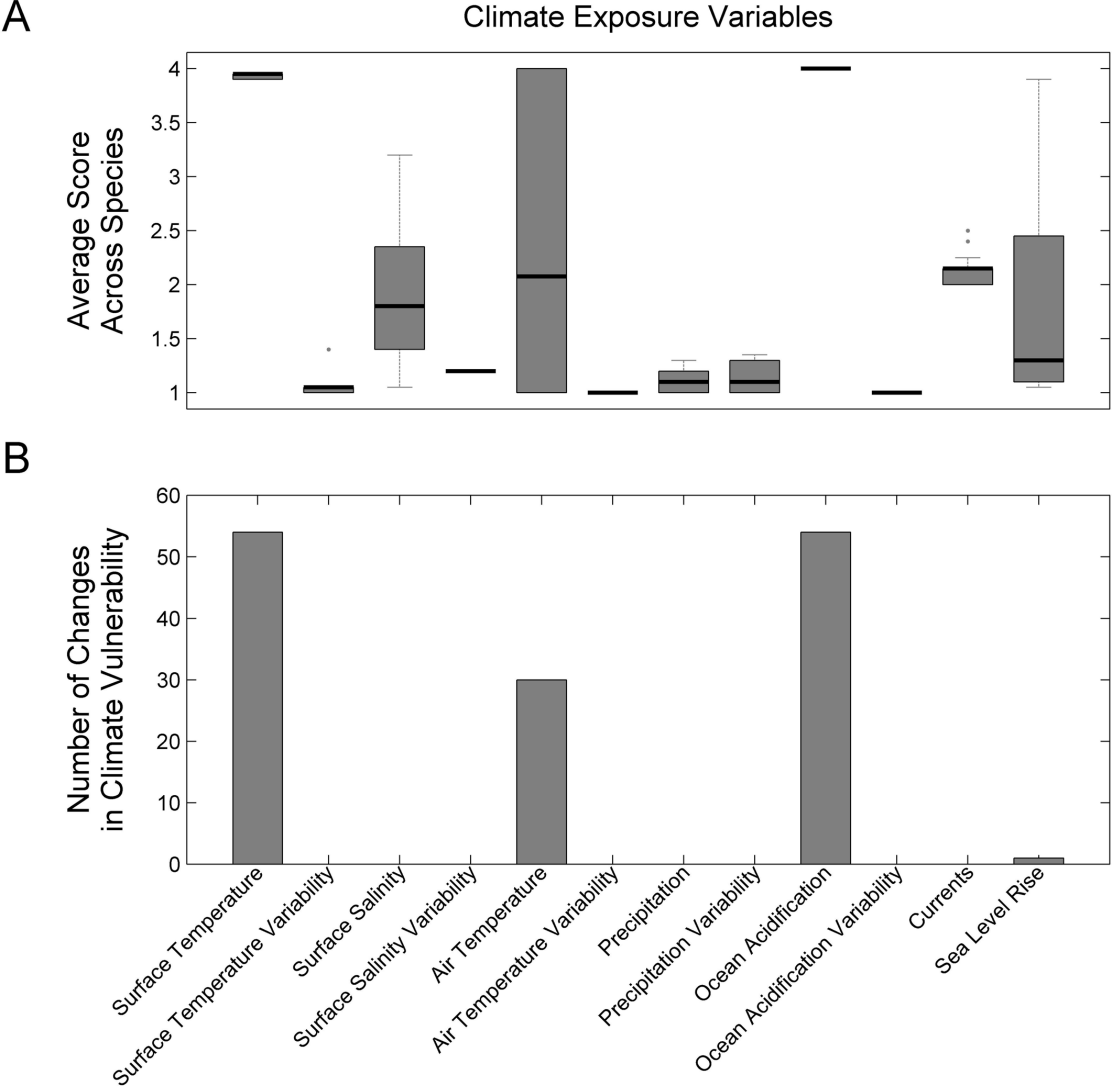
Climate exposure factors. Average climate exposure scores across all species (A) and results of sensitivity analysis for the effect of individual exposure factors on overall climate vulnerability (B).

Mean ocean surface temperature change (upper 10 m) and mean surface pH change were determined to be important factors in the climate vulnerability scores (Fig 6). These factors were scored as very high exposure for all species owing to the magnitude of change projected by 2055 (S9 Supporting Information). Mean surface air temperature change (as a proxy for shallow water temperatures) and to a lesser extent sea-level rise were also important for species that were exposed to these factors (Coastal Fish and Diadromous Fish species). Exposure factors that were not as important in determining vulnerability scores exhibited a lower magnitude of change, particularly the variance of the exposure factors and mean changes in precipitation and ocean surface salinity.

**Fig 7:**
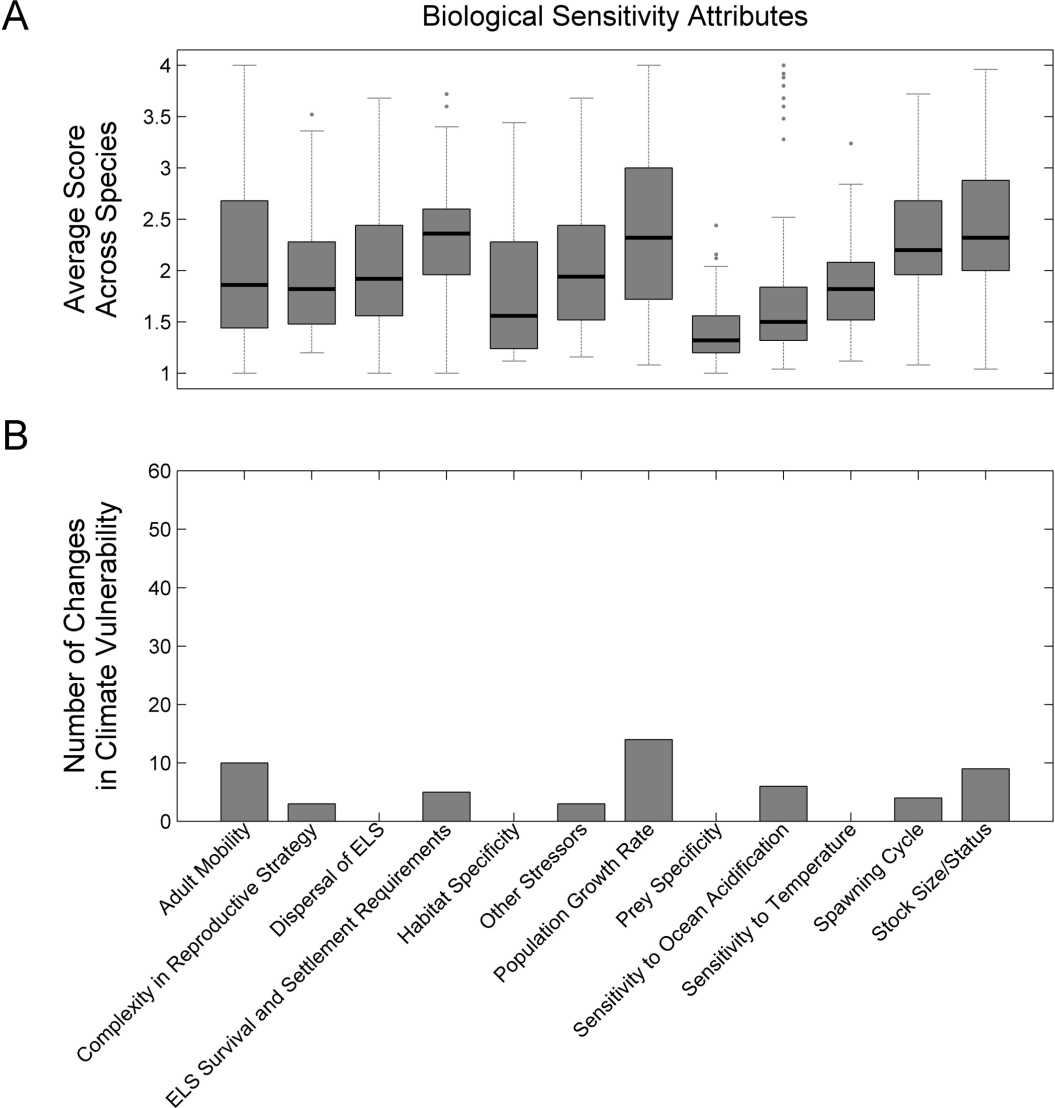
Biological sensitivity attributes. Average sensitivity attribute scores across all species (A) and results of sensitivity analysis for the effect of individual sensitivity attributes on overall climate vulnerability scores (B).

The importance of sensitivity attributes varied across species and there was no subset of dominant attributes (Fig 7). All attributes were scored as very high or high sensitivity for at least one species. There were three attributes that had the strongest influence on climate vulnerability: Population Growth Rate, Adult Mobility, and Stock Status. Removal of the attributes in the sensitivity analysis changed the scores of 14, 10, and 9 species, respectively.

### Functional Group Results

**Fig 8:**
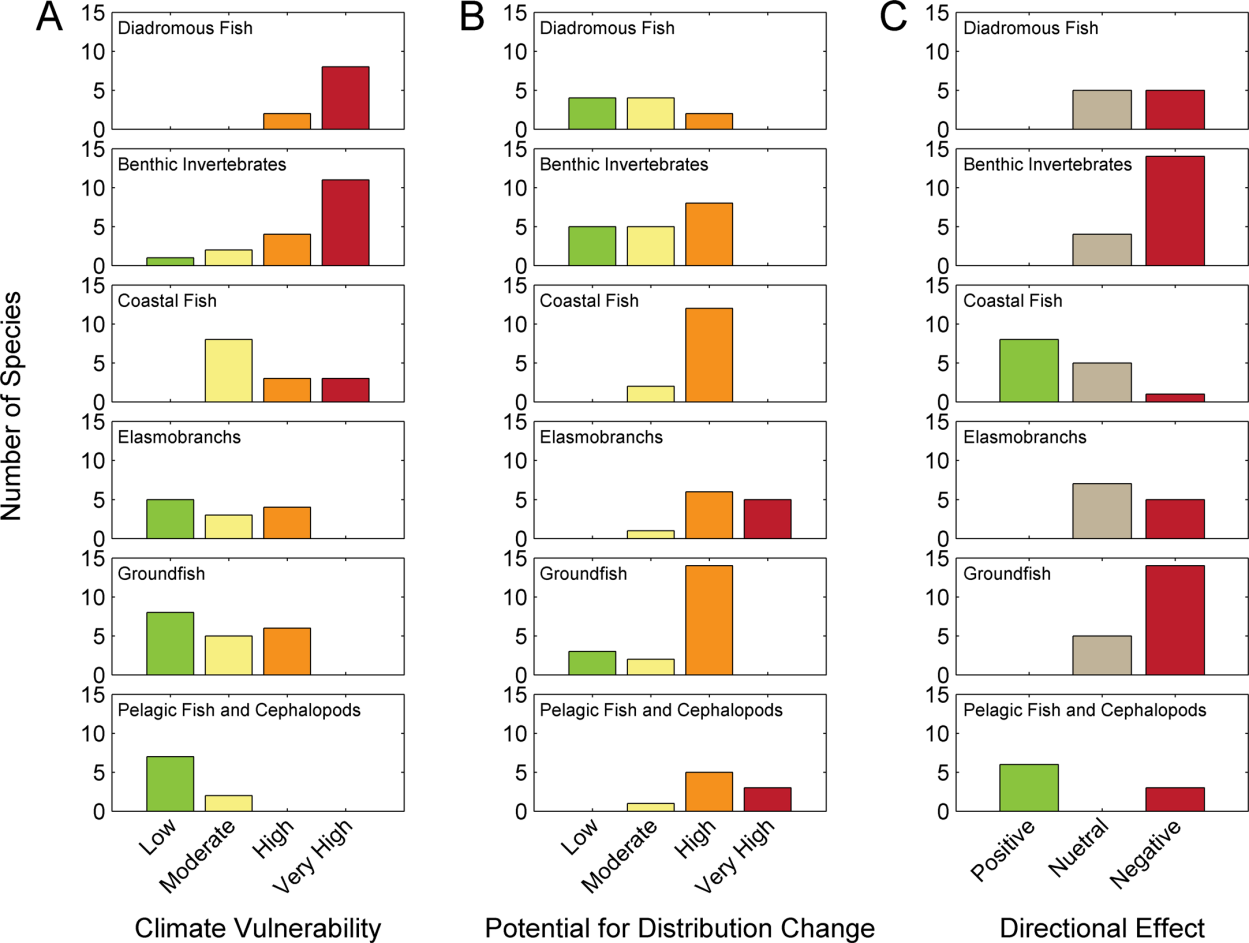
Functional groups and climate vulnerability. Number of species from each functional group by overall climate vulnerability (A), potential for distribution change (B), and directional effect of climate change (C).

The effects of climate change exhibited some consistency across functional groups. In terms of overall climate vulnerability, Diadromous Fish and Benthic Invertebrate species had the highest vulnerabilities (Fig 8). Some Coastal Fish species also scored very highly vulnerable to climate change. Elasmobranchs and Groundfish groups had no species that scored very highly vulnerable, and Pelagic Fish and Cephalopod species had no species that scored very highly or highly vulnerable. Potential for distribution change also varied by functional groups (Fig 8). Only Pelagic Fish and Cephalopods and Elasmobranchs had very high potential for a distribution change. Diadromous Fish, Benthic Invertebrates, and Groundfish had species with a low potential for distribution change. Similarly, the directional effect of climate change varied across functional groups. All groups had species where negative effects of climate change are estimated and Benthic Invertebrates and Groundfish had greatest proportion of species with estimated negative effects. Only Coastal Fish and Pelagic Fish and Cephalopods had species where positive effects of climate change are estimated within the Northeast U.S. Shelf ecosystem. MDS ordination of sensitivity attributes exhibited some consistency within functional groups but also demonstrated that certain species have attribute that are more similar to species in other functional groups (S10 Supporting Information).

## Discussion

The results of this assessment indicate that a number of fish and invertebrate species in the Northeast U.S. Shelf are highly or very highly vulnerable to climate change and decadal scale variability (Fig 3). Climate vulnerability here is defined as the extent to which abundance or productivity of a species could be impacted by climate change. This vulnerability results from the relatively large magnitude of climate change projected for the region over the next 35 years (S9 Supporting Information), as well as attributes of individual species. Changes in population productivity have already been observed in the system [4, 8, 44] and the results presented here suggest that these changes are likely to continue in the future, possibly becoming widespread among species in the ecosystem. The results of this assessment also suggest a large number of species in the ecosystem have a high potential for a change in distribution (Fig 4). Distribution changes have already been observed in the system [41, 42, 75] and have been linked to climate variables and fishing [76]. Finally, this assessment suggests that approximately half of the species included will be affected negatively by climate impacts in the region (e.g., decreased productivity, distribution shifts out of the system). However, positive effects of climate change are expected for some fish and invertebrate species in the ecosystem.

Ocean temperatures, shallow-water temperatures, and ocean acidification were the climate exposure factors with the largest magnitude of change expected by 2055, thereby contributing most to the high and very high climate exposure. There is more known about the impacts of temperature than the impacts of ocean acidification on the species in the NE LME and continued research on the effects of these factors should be a priority. The effects of temperature on species biology and ecology are well documented and there are numerous examples of incorporating temperature into models of population abundance and distribution [11, 14, 77]. These climate induced changes can result in changing reference points for management [11, 14, 78] and changes in stock distribution, which can also influence management [79]. Given the temperature changes experienced in recent decades and projected for the future in the region [39, 80, 81], temperature should be included in regional scientific advice and management. Further, research must continue to understand the mechanisms by which temperature affects species and to parameterize these affects for inclusion in population and ecosystem models [82–84].

The effects of ocean acidification on species biology and ecology are not as well understood [85]. In recent years, a number of studies have started to fill this gap for species in the Northeast U.S. Shelf [57, 86]. However, it is often difficult to place the impacts identified in experimental studies into the context of the magnitude of projected change. In the Northeast U.S. Shelf, pH is projected to decrease by 0.08 to 0.12 units by 2055 (S9 Supporting Information). Many of the studies however use treatments equivalent to ocean acidification conditions expected by 2100 or 2200. In addition, many studies use constant ocean acidification levels, but there is a large magnitude of daily, seasonal, interannual, and regional variability [87]. Research is needed that tests the impacts of ocean acidification at the levels expected over the next 20–40 years to support shorter term projections that can be used in management. One example is a recent study with Atlantic Sea Scallop where fishery yields are projected to decrease in the coming decades owing to the effects of ocean acidification [16]. Because a meta-analysis shows consistent negative impacts of ocean acidification on mollusc larvae [85] this methodology scored molluscs as very sensitive to ocean acidification (S1 Supporting Information). As these species also exhibit low adult mobility, molluscs were ranked with a high or very high vulnerability to climate change. This should be interpreted cautiously, and highlights the need for more research on species specific impacts of ocean acidification. It is imperative to experimentally examine the effect of ocean acidification on exploited species, incorporate this information into future climate vulnerability assessments, and include these effects in assessment models that evaluate the impact of acidification in the coming decades.

In contrast to the high influence of a limited number of exposure factors, the importance of biological sensitivity attributes was more variable; indicating the diversities in life history strategies among fish and invertebrates species in the Northeast U.S. Shelf. In general, Stock Status, Population Growth Rate, and Adult Mobility were the most influential attributes. Improved understanding of these attributes is identified as priority research areas. There are a number of other species specific needs that are identified in the species narratives (S7 Supporting Information). There is also a need to understand the relative role of climate change in concert with multiple other stressors in affecting population abundance. Here we focused on changing climate but included fishery status and other stressors as sensitivity attributes; stress from climate change will act in concert with other stressors. Including multiple stressors as exposure factors is possible in the vulnerability assessment framework [21, 23]; for management to result in long-term sustainability, the important stressors need to be identified and included in management considerations [88]. Fishing is still a dominant factor, but as fishing mortality decreases, the relative importance of other stressors (e.g., climate change, habitat alterations) will increase [8, 76]. New contaminant and disease concerns are being identified and require more study to understand their effect on fish and shellfish populations [89, 90]. In addition, although barriers to diadromous fish passage are decreasing, a number of barriers remain [91].

For this study, adaptive capacity was incorporated into the sensitivity attributes. Adaptive capacity includes a species ability to move when conditions change (adult dispersal), ability to acclimate to changes (plasticity in response, generalist versus specialist), and the ability to evolve as a population or species [33, 92]. This methodology incorporated aspects of the first two, but not the third; the genetic ability to adapt to climate change remains under-known [33, 92]. Understanding genotypic and phenotypic adaptive capacity in marine fish and invertebrates should be a priority for future research.

The results from this assessment can be compared with detailed studies examining past changes in fish and invertebrate species and projecting future changes. In some cases, these more detailed studies seemingly contradict the results presented here. Population productivity of both the Southern New England Yellowtail Flounder and Winter Flounder stocks has decreased and these decreases have been attributed to changes in the environment [8, 44]. In this climate vulnerability assessment, Yellowtail Flounder was ranked as having a low vulnerability to change in population productivity and Winter Flounder was ranked as having a very high vulnerability to changes in productivity. Similarly, Alewife and American Shad have exhibited some of the greatest shifts in distribution in the ecosystem [41], but the potential for a change in species distribution was low. The results of an expert-based assessment are never going to completely agree with the results of more detailed, empirical and process-oriented studies or assessments. However, expert opinion summarizes current knowledge in a defined framework and can guide future monitoring, research, and modeling studies. Further, the studies described above are not directly comparable with the results of this assessment. The changes in productivity were demonstrated for specific stocks of Yellowtail Flounder and Winter Flounder, those at the southern extent of the range, while this assessment was conducted at the species level encompassing two or three stocks. The directional effect measure estimates negative effects on both Yellowtail Flounder and Winter Flounder. Similarly, the distribution changes in Alewife and American Shad have been observed during the marine phase only. Natal homing is an important part of the life history of these species [93], so changes in spawning distribution are less likely to occur as predicted by this methodology. The important accomplishment of this assessment is to frame climate vulnerability for a majority of managed fish and invertebrate species in the ecosystem and for a number of unmanaged, but ecologically or commercially important species in the ecosystem.

The assessment was done at the species level. There are pros and cons associated with running a vulnerability assessment at finer (i.e. stock) and coarser (i.e. functional group) levels. A previous assessment in the region was performed at the functional group level [30] and management in many cases is at the stock level (sub-species) [94]. The analysis by functional group (Fig 8, S10 Supporting Information) suggests that while “indicator” species or general functional groups can be used, similar species can have different biological attributes and different overall climate vulnerabilities. A stock specific assessment would be more appropriate to fisheries management, but only in cases where regional species vulnerability is different from stock-specific vulnerability. The choice of number of species (or stocks or functional groups) is dependent on the resources available and the objectives of the assessment.

Our objective here was to provide a broad examination of climate vulnerability for fish and invertebrates on the Northeast U.S. shelf. There are other approaches to estimating species’ vulnerability to climate change. For example, thermal habitat modeling was completed to estimate available future thermal habitat on the Scotian Shelf [15, 95]. While objectives were similar, the methodologies are different enough that the results provide answers to different questions (projected available habitat vs trait based vulnerabilities). Mechanistic climate-population models are another way of investigating climate vulnerability. Models have been developed for a few species (<10 species) but to complete such detailed analyses for the 82 species included here will take several years at least [11, 14, 77]. Finally, Bioclimatic Envelop Modeling has been conducted at the global [19, 20] and regional scale [96]. All of these approaches have value and can contribute to informing managers of the challenges and opportunities that result from climate change. Together, these studies indicate that climate change is going to impact fish and invertebrate species for the foreseeable future.

In addition to addressing scientific questions and identifying important research needs, the results of this assessment can be used by managers in at least four ways. First, the information resulting from the assessment can be used to inform management and regulatory documents, including fishery management plans developed under the Magnuson-Stevens Fishery Conservation and Management Act, Biological Opinions, and Listing Decisions under the Endangered Species Act, and Environmental Impact Statements under the National Environmental Policy Act [97]. Second, the results can be used to guide management actions. The climate vulnerability of 8 species decreases if Stock Status is removed from the analysis: Atlantic Halibut, Winter Flounder, Witch Flounder, Barndoor Skate, Dusky Shark, Porbeagle, Rossette Skate, Sand Tiger, and Thorny Skate. Efforts to decrease fishing mortality on these species might result in an increase in stock size and thus lower overall climate vulnerability. Third, the assessment can contribute to the development of regional Ecosystem-Based Fisheries Management [98], inform spatial management (e.g., management areas for future refuges of vulnerable species), and guide the inclusion of climate variables in single-, multi-species and ecosystem models [99, 100]. Fourth, the vulnerability narratives are useful for identification of species specific research and management needs as they provide in-depth species specific scores as well as a discussion of earlier climate related studies on that species.

We advocate that the Fisheries Climate Vulnerability Assessment should be conducted iteratively. Most fishery stock assessments are conducted iteratively [101] and we recommend conducting the climate vulnerability assessments following the same interval as the IPCC Assessment Reports. Similarly, Integrated Ecosystem Assessments are planned to be iterative [102]. The implementation of the Climate Vulnerability Assessment Methodology was successful (S11 Supporting Information). However, there are improvements that should be considered for the next iteration. First, bottom temperature and improved ocean acidification projections should be included; both of these would require some form of regional downscaling [77] or higher resolution climate models [103]. Second, the ensemble uncertainty in climate change could also be included formally in the assessment (S12 Supporting Information), along with different emission scenarios and potentially time periods. Third, the next iteration should include an update of the species profiles and rescoring of both the climate exposure and sensitivity attributes. If information on species specific plasticity or evolutionary adaptability exist by the next iteration, this information should be included to improve the evaluation of adaptive capacity [33, 104, 105]. Fourth, the next assessment should include experts from a broader background than used here. This assessment included only NMFS employees with some outside observers. One of the main objectives of this effort was to provide the first implementation of NMFS Methodology [34]. This will not be an objective of the next iteration and efforts should be made to include a broad array of fisheries, protected species, and ecosystem stakeholders. Fifth, there are potential links that could be made between Overfishing Vulnerability Assessments [24, 25], Habitat Vulnerability Assessments [106] and Social Vulnerability Assessments [107]. The vulnerability assessment framework provides a powerful tool that can complement other assessment techniques and support the broader implementation of Ecosystem-Based Management and climate change adaptation strategies.

## Supporting Information

S1 Dataset Vulnerability Results. (CSV)

S2 Dataset Directional Effect Results. (CSV)

S1 Supporting Information Sensitivity Attributes. (PDF)

S2 Supporting Information Ocean Currents Exposure. (PDF)

S3 Supporting Information Sea-level Rise Exposure. (PDF)

S4 Supporting Information Climate exposure scoring. (PDF)

S5 Supporting Information Assessment Results. (PDF)

S6 Supporting Information Data Quality. (PDF)

S7 Supporting Information Species Narratives. (PDF)

S8 Supporting Information Climate Vulnerability and Distribution Change Potential. (PDF)

S9 Supporting Information Climate Exposure Maps. (PDF)

S10 Supporting Information MDS Ordination of Sensitivity Attributes. (PDF)

S11 Supporting Information Methodology Evaluation. (PDF)

S12 Supporting Information Climate Model Uncertainty. (PDF)
