# Supplementary material for: A Vulnerability Assessment of Fish and Invertebrates to Climate Change on the Northeast U.S. Continental Shelf
Source: PLoS One. 2016 Feb 3;11(2):e0146756. doi: 10.1371/journal.pone.0146756 (PMC4739546; doi:10.1371/journal.pone.0146756)
Supplement: S1 Supporting Information — (PDF) [file pone.0146756.s003.pdf]

S1 Supporting Information. Sensitivity Attributes

Table of Contents

BACKGROUND 2

SENSITIVITY ATTRIBUTES 2

Habitat Specificity 3

Prey Specificity 5

Sensitivity to Ocean Acidification 6

Complexity in Reproductive Strategy 6

Sensitivity to Temperature 10

Early Life History Survival and Settlement Requirements 12

Stock Size/Status 14

Other Stressors 16

Population Growth Rate 17

Dispersal of Early Life Stages 19

Adult Mobility 21

Spawning Cycle 22

REFERENCES 23

See Morrison et al. (2015) for more details.

Morrison W, Nelson M, Howard J, Teeters E, Hare JA, Griffis R. et al. Methodology for assessing the vulnerability of fish stocks to a changing climate. NOAA Technical Memorandum. 2015; NMFS-OSF-3: 1-48. http://www.st.nmfs.noaa.gov/Assets/ecosystems/climate/documents/TM%20OSF3.pdf

Background

The goal of this project is to provide regional fisheries managers with a practical tool to efficiently assess the vulnerability of fish stocks to climate change. *Vulnerability* is defined here as the extent to which the abundance and productivity of a stock could be impacted by climate change. The potential for a change in distribution and the directional effect (positive or negative) of a changing climate are also assessed. This project considers the overall vulnerability of fish stocks to climate change to be a function of two main components: **exposure** and **sensitivity**.

Exposure is a measure of the predicted environmental change that the stock may experience within its range. It is the overlap between the stock's distribution and the magnitude of and spatial distribution of the expected environmental change. The factors accounted for in exposure may include increases in temperature, changes to freshwater input, rise in sea level, changes to ocean circulation, etc.

The *sensitivity* component is composed of biological attributes which are believed to be indicative of the response that a stock will have to potential changes in climate. Sensitivity includes the attributes that describe the stock's resilience (the ability of a stock to survive and recover from a perturbation) and its adaptive capacity (the ability of a stock to adapt, reduce or mitigate the consequences through evolutionary changes and plastic ecological responses) (Williams et al. 2008).

This document provides definitions, justifications, links to climate change and scoring bins for each of the sensitivity attributes. This vulnerability assessment can be completed at either the species level or the stock level, depending on the goals of the assessment. We have used the term "stock" throughout this document; however, where appropriate, it can be replaced by "species" if that is the level of analysis being implemented.
This is the first version of this methodology. As more information becomes available on which stocks are more likely to be impacted by changes in climate, the following attribute definitions will need to be updated.

This methodology leans heavily on expert opinions. Experts should use their expert knowledge when using and interpreting these attributes and attribute bins. For example, experts may encounter a situation where the scoring bins suggest a specific attribute score, but their expert knowledge of the species or data makes them think the score should be higher, lower, or more uncertain. We are counting on the experts to make these calls. However, experts should be prepared to provide justification for their scores.

SENSITIVITY ATTRIBUTES

Habitat Specificity

Goal: To determine, on a relative scale, if the stock is a habitat generalist or a habitat specialist while incorporating information on the type and abundance of key habitats.

Relationship to climate change: Stocks that are reliant on specific habitat types may be more vulnerable to climate change because they are dependent on not only their own response to climate change, but also the impact on their habitat (EPA 2009). Note: the type (biotic vs. abiotic) and distribution of these habitats should be considered for this attribute.

Background: Changes in climate are expected to alter marine and coastal habitats that fish stocks depend upon. Species that are habitat generalists (can utilize several different habitat types) are expected to be more likely to succeed in a changing environment. The more a species specializes on a specific habitat, the more likely the species will be impacted by an environmental change. However, not all habitats are expected to be impacted equally. Stocks that depend on habitats that are abundant and wide ranging are less likely to be impacted by changes than species that depend on habitats that are limited in scope. We expect habitats that are created by disturbances (e.g. coral rubble or edge habitats) to increase with climate change. In addition, biological habitats (i.e., live coral reefs, mangroves, salt marshes, sea grass beds) are more likely to be impacted by the changes than physical habitats (sand, mud, rocky bottom). When considered together, these three criteria (habitat specialist or generalist; whether or not the stock depends on biological habitats; and habitat availability) are indicative of how a stock will be impacted by climate-induced changes on habitat.

How to use expert opinion: This attribute will be scored using a combination of the three criteria described above: habitat specialist or generalist; whether or not the stock depends on biological habitats (i.e., live coral reefs, mangroves, salt marshes, sea grass beds); and habitat availability (limited vs. abundant). It is understood that these criteria are not dichotomous but are a continuum. Stocks that are dependent on "disturbed" habitats should do fine or increase with climate change, so put these species in the "low" bin. If you think that a stock fits in multiple scoring bins, weight your 5 tallies between the appropriate bins. Using your expert opinion, account for any lifespan or ontogenetic shifts in diet; however, limit your response to the juvenile and adult life stages as larvae are considered under the attribute "early life history survival and settlement requirements."

Habitat Specificity Bins:

1. **Low: The stock is a habitat generalist and/or utilizes very common physical habitats.** Occurrences of the stock have been documented in diverse habitats. Also, included in this bin are stocks that are restricted to one physical habitat which is widespread and common (e.g. vast stretches of sandy bottom, or pelagic waters over a large range).

2. **Moderate: The stock strongly prefers a particular habitat.** The stock prefers a particular habitat, but can survive in other habitats (with possible impacts to their fitness).
3. **High: The stock is a specialist on an abundant biological habitat.** The stock is a specialist that is restricted to a specific, but common biological habitat.
4. **Very High: The stock is a specialist on a restricted biological habitat.** The stock is a specialist that is restricted to a specific and uncommon biological habitat.

Prey Specificity

Goal: To determine, on a relative scale, if the stock is a prey generalist or a prey specialist.

Relationship to climate change: Understanding how reliant a stock is on specific prey species could predict its ability to persist as the climate changes. Generalists (who feed across a wide spectrum of prey types) should have a better chance to persist in response to a changing environment. Alternatively, specialists (who have specific prey requirements) are likely to be more vulnerable to climate change because their persistence is dependent on not only their own response to climate change, but also the response of their prey.

Background: Climate change impacts extend beyond the stock in question to include species within its food web (e.g., prey, predators and competitors).

How to use expert opinion: The scoring bins below estimate the stocks' relative distribution along a continuum that runs between prey specialists and prey generalists. Using your expert opinion, account for any lifespan or ontogenetic shifts in diet; however, limit your response to the juvenile and adult life stages as larvae are considered under the attribute "early life history survival and settlement requirements." For this attribute, prey type refers to groups of similar species; copepods, krill, forage fish, etc., for example, are each categorized as a prey type.

Prey Specificity Bins:

1. **Low: The stock eats a large variety of prey.** The stock can eat a variety of prey types depending on what is available. Include detritivores, herbivores, and omnivores in this bin.
2. **Moderate: The stock eats a limited number of prey types.** The stock can feed on a wide variety of prey, but are restricted to a limited number (~3) of prey types (copepods, krill, forage fish, etc).
3. **High: The stock is partial to a single prey type.** The stock's diet is composed of one main prey type. The stock is able to switch to a different prey type if the preferred food is unavailable, but this may negatively impact fitness.
4. **Very High: The stock is a specialist.** The stock is dependent on one prey type and is unable to switch to alternatives if the preferred prey is unavailable.

Sensitivity to Ocean Acidification (OA)

Goal: To estimate a stock's sensitivity to ocean acidification based on its relationship with "sensitive taxa."

Relationship to climate change: Impacts of OA on marine organisms can be highly variable, with considerable variability between taxa and species (Kroeker et al. 2013). Therefore, we are estimating impact of OA by examining the dependence of the stock on sensitive taxa. For example, current research shows a consistent negative impact of OA on mollusks and corals, so species in either of these classes or dependent on species in these classes should be considered more sensitive to changes in ocean pH. We expect the volume of research into ocean acidification to increase in the near future, so this attribute will be updated as new information becomes available.

Background: Ocean acidification is often called "the other carbon dioxide problem," and is the term given to the chemical changes in the ocean as a result of carbon dioxide emissions (Wicks and Roberts 2012). While initial research suggested that the majority of species that have calcium carbonate or chitin shells or those that lay down calcium carbonate skeletons (corals) will be negatively impacted by ocean acidification (Arnold et al. 2009; Hoegh-Guldberg et al. 2007;Honisch et al. 2012; Kawaguchi et al. 2011; Orr et al. 2005), recent studies have highlighted a high variablilty in response between different shelled organisms and suggest that not all shelled species will be impacted to the same degree and not all impacts will be negative. (i.e.,Ries et al. 2009, Kroeker et al. 2013). For example, Kroeker et al. (2013) in a meta-analysis of 228 studies found significant and consistent negative impacts of OA on the larval stages of mollusks and corals (see Figure 4 from Kroeker et al. below). In contrast, high variability in the responses of crustaceans suggests impacts may be species specific within this group, with brachyuran crustaceans showing a higher resistance (Kroeker et al. 2013).

The direct effect of ocean acidification on finfish is not well understood. Recent research suggests impacts on finfish stocks will be most prevalent at the egg and early larval stages (Baumann et al. 2011; Franke and Clemmenssen 2011; Frommel et al. 2011),but juvenile and adult olfaction may also be affected (Mundy et al. 2009).Despite these studies, not enough is known to be able to predict which finfish stocks will be more sensitive. This attribute will be updated when more information is available on which finfish stocks are more likely to be directly impacted by ocean acidification.

How to use expert opinion: Use the results presented in Figure 4 from Kroeker et al. 2013 (or other relevant information) to bin species. When scoring, base your score on the most sensitive life stage, if appropriate. In cases where research has shown that the effects of OA may be positive or mitigated by biological processes (e.g. reduced OA by plant absorption of CO_2), use your expert judgment to inform the score.

Sensitivity to Ocean Acidification Bins:

1. **Low: Stock not reliant on sensitive taxa.** The stock does not utilize sensitive taxa for food or habitat. Species expected to respond positively to ocean acidification should be scored as low.
2. **Moderate**: **Stock is somewhat reliant on sensitive taxa.** The stock utilizes sensitive taxa as either food or habitat. This can include omnivores and species that prefer coral habitats but can utilize any rigid structure.
3. **High: Stock is reliant on sensitive taxa.** The stock is highly dependent on sensitive taxa for either food or habitat (i.e., cannot switch to a non-sensitive alternative).
4. **Very High: Stock is a sensitive taxa.** The stock is a sensitive taxa (such as corals or mollusks) that have been shown to have a consistent negative impact of OA on survival, growth or abundance.

Complexity in Reproductive Strategy

Goal: To determine how complex the stock's reproductive strategy is and how dependent reproductive success is on specific environmental conditions.

Relationship to climate change: Species that have complex reproductive strategies (that require a series of events or special conditions) are more likely have these conditions disrupted by changes in the environment.

Background: There is great diversity in reproductive strategies in marine fishes. The more complex the reproductive strategy, the more precise the conditions may need to be, and thus the more vulnerable the stock may be to environmental change. For our purposes, complexity in reproductive strategy is defined as reproductive behaviors, characteristics or cues that create specific requirements that must be met in order for reproduction to be successful.

How to use expert opinion: A list of common reproductive characteristics that may affect the reproductive capacity of a stock in a changing climate is provided below. To score, determine if any of these examples apply to the stock. Note: this is not intended to be an exhaustive list. If other characteristics exist that may affect a stock's reproduction capacity in a changing climate, incorporate that information and adjust your score appropriately.

Example reproductive characteristics that create "complexity":

- The stock has known temperature effects on reproduction. Examples include temperature-dependent sex changes, and temperature cues that impact spawning, gonad development, etc.
- The stock uses large spawning aggregations. Large spawning aggregations can contribute to a high sensitivity because a large number of individuals must get to the spawning area simultaneously (i.e., migration or cues to migrate may be impeded by a change in the environment), the spawning area has to retain the environmental conditions that made it successful in the past, and the reproductive success for that year is dependent on the conditions present at one time period.
- The stock experiences decreased recruitment at low stock sizes due to depensation/allee effects. If this is not known, does the stock share life history characteristics that would predict strong alee effects (e.g., at low densities, urchins can experience decreased fertilization and thus reduced recruitment)?
- The reproductive success of the stock requires the use of vulnerable habitats (freshwater, estuaries, mangroves, salt marshes, coral reefs) for spawning or rearing of young. Vulnerable habitats are likely to experience larger climate change impacts (such as changes in salinity, dissolved oxygen, pollution, sedimentation, or water depth), and stocks that require these habitats for successful reproduction will likely be impacted.

Complexity in Reproductive Strategy Scoring Bins:

1. **Low: Simple reproductive strategy.** The stock contains no more than one characteristic that suggest complexity in reproductive strategy.

2. **Moderate: Slight complexity.** The stock has two characteristics that suggest complexity in reproductive strategy.
3. **High: Complex reproductive strategy.** The stock has three characteristics that suggest complexity in reproductive strategy.
4. **Very High: Very complex reproductive strategy.** The stock has four or more characteristics that suggest complexity in reproductive strategy.

Sensitivity to Temperature

Goal: To use information regarding temperature of occurrence or the distribution of the species as a proxy for its sensitivity to temperature. Note: that this attribute uses species (vs. stock) distributions as they better predict thermal requirements.

Relationship to climate change: Species that experience a wide range of temperature regimes are more likely to persist in a warming ocean (Chin et al. 2010).

Background: The distribution of a species within or across provinces provides an estimate of its temperature requirements. Spalding et al. (2007) divides coastal waters of the world into 62 provinces and 232 ecoregions. Even though Spalding's provinces are not specifically based on temperature (they also consider upwelling, currents, salinity, nutrients, etc.), they can be used to delineate areas with similar thermal conditions.

In addition, a species' distribution in the water column and seasonal movements can indicate its sensitivity to temperature. Species that make large diurnal migrations across the thermocline have lower sensitivities to changing temperatures than species that have limited depth distributions. Additionally, species that make large seasonal migrations and track seasonally changing water temperatures may have more sensitivity to temperature than indicated by range alone.

How to use expert opinion: Use known temperature requirements to score this attribute when available. When temperature information is not known, use the species distribution, along with Spalding et al. (2007) to determine if a species is found across >1 province. Also use knowledge of seasonal and diurnal movements to adjust the tallies. Keep in mind that you can adjust your tallies depending on the distribution of the species relative to the area of interest (i.e. if the area of interest is at the edge of the distribution of the species, consider if the species is expected to move out of or expand into the area of interest). Spalding et al. (2007) only characterize coastal environments; therefore, use your expert opinion for open ocean species. If information about temperature requirements or depth distributions is available, use this to modify your response. For example, if a species is found across 2 provinces, but it has a limited depth distribution, the expert could distribute the 5 tallies between bins 2 and 3. If a species' sensitivity changes with ontogeny, consider the most limited stage when determining the most appropriate bin(s).

Temperature Sensitivity Bins:

1. **Low: Large temperature range.** Species occurs in a wide range of temperatures (>15°C), or is found across 3 or more provinces.
2. **Moderate: Moderate temperature range.** Species occurs in a moderately wide range of temperatures (10-15°C), or is found across 2 provinces.
3. **High: Somewhat limited temperature range.** Species occurs in a moderately narrow range of temperatures (5-10°C), or is found within one province but has a variable depth distribution.

4. **Very High: Very limited temperature range.** Species occurs in a narrow range of temperatures (<5°C), or is found within one province and has a limited depth distribution (i.e., depth range is <100 m).

Early Life History Survival and Settlement Requirements

Goal: To determine the relative importance of early life history requirements for a stock.

Relationship to climate change: In general, the early life stages (eggs and larvae) of marine fish are characterized by high mortality rates, via predation, starvation, advection, or unsuitable conditions. Small changes in the environment can lead to large changes in early life survival, which can affect recruitment and year-class strength.

Background: Close to 100 years ago, fisheries scientists recognized the importance of recruitment variability in fish populations (Hjort 1914). Since then, multiple hypotheses have been developed to explain this variability, but scientists now understand that multiple processes are important during the egg and larval stages (Houde 2008). Conditions that can lead to decreased or negligible recruitment include:

- Larvae that are dependent on specific biological conditions in the water column during their larval stage. For example, if the larvae are dependent on the presence of food at a specific point in development, different emergence of the larvae and the food (due to dependence on different cues) could result in a mismatch in availability. Alternatively, if the larvae have evolved to survive in low predator (and low food) conditions, a change in predation pressure could impact survival (Bakun 2010).
- Larvae that are dependent on specific physical conditions to survive (e.g., temporary gyres that provide food and retention, calm conditions that allow for concentration of prey, specific transport pathways to nursery habitats, etc.).
- Larvae that are dependent on a settlement habitat or cue that could be impacted by a changing climate.

For the purpose of this assessment, early life history requirements include the environmental conditions necessary for larval survival, and encompass the eggs, pelagic larvae stages, and settlement. The more specific the early life history requirements, the more precise the environmental conditions may need to be, and thus the more vulnerable the stock may be in a changing environment. Note: some fish species, namely elasmobranchs, have evolved life history traits which minimize or eliminate early life stages either by birthing well-developed young or by laying egg cases that allows embryos to fully develop before hatching. Therefore, elasmobranchs should ranked as "Low."

How to use expert opinion: Marine species are largely dependent on both physical and biological conditions during their larval stage. However, the specificity of these conditions varies between stocks. If no citable reference is available, the score may be based on expert opinion.

Early Life History Survival and Settlement Bins:

1. **Low: Larval requirements are minimal.** Stock has general requirements for the larval stage that are relatively resilient to environmental change. Elasmobranchs should be ranked as "Low."

2. **Moderate: Larval requirements are minimal or unknown.** Stock requirements are not well understood and recruitment is relatively constant, suggesting limited environmental influence.
3. **High: Larvae have some specific requirements.** Stock requirements are not well understood, but recruitment is highly variable and appears to have a strong dependence on environmental conditions.
4. **Very High: Larvae have multiple specific requirements.** Stock has specific known biological and physical requirements for larval survival.

Stock Size/Status

Goal: To estimate stock status to clarify how much stress from fishing the stock is experiencing and to determine if the stock's resilience or adaptive capacity are compromised due to low abundance.

Relationship to climate change: It is assumed that a stock that has a large biomass is more resilient to changes in climate. Conversely, stocks with very low biomass are likely to be in a compromised ecological position and therefore may have a diminished capability to respond to climate change (Rose 2004). The genetic diversity, as well as the abundance, of a stock can impact its susceptibility. The assumption is that species with a limited genetic diversity could be more negatively impacted by climate change as their offspring would be less variable and thus less likely to have the combination of genes needed to adapt to changes in the environment. Note: stocks that are at historical high biomass levels may be an indication of a net positive effect to an environmental change.

Background: Fish stocks that are already being affected by other stressors are likely to have faster and more acute reactions to climate change. Fishing is the largest stressor currently impacting fish stocks (Jackson et al. 2001), and the magnitude of the stress can be estimated through the status of the stock. Stock size/status can be measured as a ratio of the current stock size (B) over the biomass at maximum sustainable yield (B_{MSY}) and is a commonly used biological reference point for federally managed stocks. Use the following link for information on current estimates of B/B_{MSY}: http://www.nmfs.noaa.gov/sfa/statusoffisheries/SOSmain.htm.

Low genetic variation can decrease a species' ability to adapt to climate change. Large variation in reproductive success between individuals, large fluctuations in population size, and frequent local extinctions can all decrease genetic diversity (Grosberg and Cunningham 2001). Presence of these characteristics could suggest a decreased ability to adapt to changes in the environment.

Beyond stock status and genetic diversity, there are additional concerns for stocks that are particularly rare. The IUCN (Musick 1999) set a level of <10,000 individuals as the criteria for a stock being considered vulnerable to the risk of extinction. Therefore, for the purposes of this attribute, stocks with population sizes less than 10,000 individuals are considered to have significantly reduced ability to adapt to climate change and should be scored as "High."

How to use expert opinion: If a direct measure of biomass is not available, biomass proxies (such as survey indices or spawning stock biomass) may be used. For data-poor stocks with an unknown status, or stocks that are analyzed as part of a species group, use your expert opinion to estimate the stock size and rate the data quality accordingly. Also, if a stock has known low genetic diversity, adjust your ranks accordingly.

Stock Size/Status Bins:

1. **Low:** $B/B_{MSY} \geq 1.5$ (or proxy)
2. **Moderate:** $B/B_{MSY} \geq 0.8$ but < 1.5 (or proxy)

3. **High:** $B/B_{MSY} \geq 0.5$ but < 0.8 (or proxy)
4. **Very High:** $B/B_{MSY} < 0.5$ (or any stock below <10,000 individuals)

Other Stressors

Goal: To account for conditions that could increase the stress on a stock and thus decrease its ability to respond to changes.

Relationship to climate change: In most cases but not all, climate change is predicted to exacerbate the effects of other stressors. Fish stocks that are already being affected by other stressors are likely to have faster and more acute reactions to climate change.

Background: A stress is an activity that induces an adverse effect and therefore degrades the condition and viability of a natural system (Groves et al. 2000; EPA 2008). This attribute attempts to take into account interactions between climate change and other stressors already impacting fish stocks. Some examples of other stressors include: habitat degradation, invasive species, disease, pollution, and hypoxia. Although climate change is not currently the biggest threat to many natural systems, its effects are projected to be an increasingly important source of stress in the future (Mooney et al. 2009). Consideration of observed and projected impacts of climate change in the context of other environmental stressors is essential for effective planning and management.

How to use expert opinion: For the purpose of this assessment, we are looking for detrimental impacts from other stressors. We have provided examples of other stressors that may be impacting stocks, but the list is not exhaustive. If the stock being scored is suffering from a known or suspected stressor that is not listed below, adjust the score appropriately. It is expected that in some cases, impacts of climate change could create positive impacts (e.g., reduction in predators). If you suspect positive impacts, adjust tallies toward the lower bins as appropriate. We are not including fishing pressure as a stressor here as it is covered under the "stock size/status" attribute.

Example stressors the stock may be experiencing:

- The habitat on which the stock depends is degraded. Examples include anthropocentric effects or changes to freshwater input, stratification, storm intensity, and hypoxia.
- The stock is currently exposed to detrimental levels of pollution (chemical and/or nutrient).
- The stock has experienced a known increase in parasites, disease, or harmful algal bloom exposure.
- The stock has experienced a detrimental impact due to a change in the food web. Examples include increases in the abundance of predators or competitors, or the introduction of an invasive species that negatively impacts the stock. Do not include changes to prey here as they are covered under the "prey specificity" attribute.

Other Stressors Bins:

1. **Low: Stock is experiencing no known stress other than fishing.** Stock is experiencing no more than one known stressor.

2. **Moderate: Stock is experiencing limited stress other than fishing.** Stock is experiencing no more than two known stressors.
3. **High: Stock is experiencing moderate stress other than fishing.** Stock is experiencing no more than three known stressors.
4. **Very High: Stock is experiencing high stress other than fishing.** Stock is experiencing four or more known stressors.

Population Growth Rate

Goal: To estimate the relative productivity of the stock.

Relationship to climate change: More productive stocks are, in general, better suited to rebound after the population is stressed by changes in the environment, such as climate change.

Background: Population growth rate is defined as the maximum population growth that would be expected to occur under natural conditions (e.g., no fishing). The amount the population changes over time can be attributed to births, deaths, emigration, or immigration of individuals between separate populations (EPA 2009). If direct measurements of population growth rate (r) are unavailable, other biological reference points that are correlated with population growth rate can be used: von Bertalanffy growth rate (k), age at maturity, maximum age and natural mortality. Scoring bins for these proxies were modified from Musick (1999) by an analysis of 141 marine fish species that were considered to be representative of U.S. fisheries (Patrick et al. 2009).

How to use expert opinion: Multiple proxies may be used to inform the final score, but the accuracy and precision of the different proxies should be considered. For example, a stock with a "good" estimate of age at maturity is in the range for a "High" score, and a "fair" estimate of maximum age is in the range for the "High" scoring bin. In that case, the scorer should use their expert opinion to weight their response according to their confidence in the estimates. If no estimates are available, estimate a relative score for the stock across a continuum of r-selected (low) vs. k-selected (high) species.

Population Growth Rate Bins:

Parameter	Low	Moderate	High	Very High
Intrinsic rate of increase (r)	> 0.50	0.16 - 0.50	0.05 - 0.15	< 0.05
von Bertalanffy K	> 0.25	0.16 - 0.25	0.11 - 0.15	<= 0.10
Age at maturity	< 2 yrs	2 - 3 yrs	4 - 5 yrs	> 5 yrs
Maximum age	< 10 yrs	11 - 15 yrs	15 - 25 yrs	> 25 yrs
Natural mortality (M)	> 0.50	0.31 - 0.50	0.21 - 0.30	< 0.2

Dispersal of Early Life Stages

Goal: To estimate the ability of the stock to colonize new habitats when/if their current habitat becomes less suitable.

Relationship to climate change: In general, the greater the dispersal of larvae, the better its ability to respond to climate change. Wide distribution of eggs and larvae can lead to greater ability to colonize new habitats in areas that are suitable for survival. Conversely, if a stock has limited larval distribution and the habitat in the localized area becomes unsuitable, then the stock is more likely to be negatively affected.

Background: For marine species, extended larval dispersal is an important strategy for colonizing new areas. Duration of the larval stage may impact dispersal distance and stock persistence. Jablonski and Lutz (1983) found that marine invertebrates with relatively long planktonic larval stages were more persistent in the fossil record than those species with non-planktonic larvae and had lower extinction rates. Early life stage dispersal is affected by a number of factors including spawning, advection, diffusion, larval behavior, planktonic duration, planktonic survival, and settlement habitat (Pineda et al. 2008, Hare and Richardson in press). In general, studies have found that spawning time and place and planktonic duration are key factors, but the other factors can be important in specific situations.

How to use expert opinion: The main point of this attribute is to estimate dispersal ability. If a stock has a relatively short larval duration, but is known to disperse large distances, or if the larvae are able to influence dispersal through selective tidal stream transport, adjust your tallies accordingly. Keep in mind that long-distance dispersal of only a small fraction of the larvae could still be adequate for colonization of new areas in a changing climate. For elasmobranchs that have evolved life history strategies that produce a smaller number of well-developed offspring, the impact of this attribute will be reduced. For elasmobranchs with live birth, dispersal will occur while in utero and should be scored as low to moderate. For elasmobranchs with egg cases, egg dispersal will be more limited, but juveniles will have the ability to disperse if needed so these stocks should be scored as moderate to high. Bins were modified from Pecl et al. (2014).

Dispersal of Early Life Stages Bins:

1. **Low: Highly dispersed eggs and larvae.** Duration of planktonic eggs and larvae greater than 8 weeks and/or larvae are dispersed >100 km from spawning locations.
2. **Moderate: Moderately dispersed eggs and larvae.** Duration of planktonic eggs and larvae less than 8 but greater than 2 weeks and/or larvae are dispersed 10-100 km from spawning locations.
3. **High: Low larval dispersal.** Duration of planktonic eggs and larvae less than 2 weeks and/or larvae typically found over the same location as parents.
4. **Very High: Minimal larval dispersal.** Benthic eggs and larvae or little to no planktonic early life stages.

Adult Mobility

Goal: To estimate the ability of the stock to move to a new location if their current location changes and is no longer favorable for growth and/or survival.

Relationship to climate change: Site-dependent species that are unable to move to better habitat when a location becomes unfavorable are less able to adapt to environmental change than highly mobile species.

Background: As climate change occurs, habitats that were once suitable may change and no longer be able to sustain a given stock of fish. Similarly, what was once unsuitable habitat may become suitable. A stock can survive changes in habitat as long as they have the ability to disperse from unsuitable habitat and find new, suitable habitat. This can occur through larval dispersal and settlement (covered under the “Dispersal of Early Life Stages” attribute) or through adult mobility. Species can be limited in their mobility by physical or behavioral (e.g., won’t swim across open ocean) barriers.

How to use expert opinion: This attribute represents a continuum from sessile to highly migratory organisms. Use your expert opinion to place the stock in question in the appropriate bin according to its physical and behavioral ability to move. Homing behavior for spawning should not be considered here as it is accounted for in the “Complexity in Reproductive Strategy” attribute. For this attribute, we define site-dependent stocks as those whose adults are site-attached (i.e. spend their entire adult phase in one limited location).

Adult Mobility Bins:

1. **Low: Non-site dependent**. The stock is highly mobile and non-site dependent.
2. **Moderate: Site dependent but highly mobile.** The stock has site-dependent adults capable of moving from one site to another if necessary.
3. **High: Site dependent with limited mobility.** The stock has site-dependent adults that are restricted in their movement by environmental or behavioral barriers.
4. **Very High: Non-mobile.** The stock has sessile adults.

Spawning Cycle

Goal: To determine if the duration of the spawning cycle for the stock could limit the ability of the stock to successfully reproduce if necessary conditions are disrupted by climate change.

Relationship to climate change: It is assumed that stocks that spawn over an extended period of time will be more likely to be successful in a changing environment. Conversely, stocks that spawn all at once in major events are more likely to experience recruitment failure with potential changes in environmental conditions.

Background: Spawning characteristics describe the spawning activity of a stock (in aggregate, not individually) over a particular time frame. If a stock spawns several times per year across a variety of seasons, then they will likely be less susceptible to climate change because their reproductive events are not dependent on just one set of very specific conditions (e.g., phenological events). Increased spawning events, a type of bet hedging, also help to protect against vulnerabilities associated with single spawning aggregations (see the "Complexity in Reproductive Strategy" attribute). Similarly, stocks that reproduce seasonally are also less likely to adapt to climate change as they are dependent on environmental conditions historically present during a given season that may not persist through time. For example, spring-like conditions and related activities have occurred progressively earlier since the 1960s (Walther et al. 2002) and changes in spawning season and location have already been observed and predicted to continue (Shoji et al. 2011; Rijnsdorp et al. 2009). Note: We are describing the spawning activity of the entire stock, not the individual. In other words, we are interested in the time from when spawning commences until when it ends, not how long a single individual spawns.

How to use expert opinion: It is impossible to distill every potential spawning cycle into 4 scoring bins. The below bins are rough breaks in a continuum of possibilities. If a species does not fit the below bins, use your expert judgment to best score the species based on the above discussion. For stocks (such as elasmobranchs) that are born as fully developed juveniles capable of long distance movements, there is less concern over a short hatching/mating period, and these stocks should be ranked low to moderate.

Spawning Characteristics Bins:

1. **Low: Consistent throughout the year.** Stocks that spawn continuously throughout the year without a defined "spawning season" are considered to be at the lowest risk of suffering from adverse affects of climate change. Example: a stock that spawns daily or monthly.
2. **Moderate: Several spawning events throughout the year.** Stocks that spawn several times per year and spawn across more than one season have a moderate risk. Example: a stock that spawns in both the spring and summer.
3. **High: Several spawning events per year within a confined time frame.** Stocks that may spawn several times per year but all spawning events in that year take place in one season have a high risk of being effected by climate change. Example: the spawning season occurs once a year and lasts over a period of less than 3 months.

4. **Very High: One spawning event per year.** Stocks that require very specific environmental/social queues to initiate spawning and that only spawn once per year are at the highest risk level for being affected by climate change. Example: the spawning season occurs once a year over a brief period of time.

References

Arnold KE, Findlay HS, Spicer JI, Daniels CL, and Boothroyd D. (2009) Effect of CO_2 related acidification on aspects of the larval development of the European lobster, *Homarus gammarus*. Biogeosciences 2009; 6(8): 1747-1754. doi:10.5194/bg-6-1747-2009

Bakun A. Linking climate to population variability in marine ecosystems characterized by non-simple dynamics: Conceptual templates and schematic constructs. J Mar Syst. 2009; 79: 361-373. doi:10.1016/j.jmarsys.2008.12.008

Baumann H, Talmage S, Gobler C. Reduced early life growth and survival in a fish in direct response to increased carbon dioxide. Nat Clim Change. 2011; 2: 38-41. doi:10.1038/nclimate1291

Chin A, Kyne PM, Walker TI, McAuley RO. An integrated risk assessment for climate change: analysing the vulnerability of sharks and rays on Australia's Great Barrier Reef. Global Change Biology. 2012 16(7), 1936-1953.

U.S. Environmental Protection Agency (EPA). A framework for categorizing the relative vulnerability of threatened and endangered species to climate change. National Center for Environmental Assessment, Washington, DC; 2009. EPA/600/R-09/011. Available: http://ofmpub.epa.gov/eims/eimscomm.getfile?p_download_id=492883

U.S. Environmental Protection Agency (EPA). U.S. EPA's 2008 Report on the Environment (Final Report). Washington, DC; EPA/600/R-07/045F (NTIS PB2008-112484). Available: http://cfpub.epa.gov/si/si_public_record_report.cfm?dirEntryId=190806&subject=Air%20Research&showCriteria=0&searchAll=Air%20and%20Monitoring&actType=Product&TIMSType=PUBLISHED+REPORT&sortBy=revisionDate

Franke A, Clemmesen C. Effect of ocean acidification on early life stages of Atlantic herring (*Clupea harengus L.*). Biogeosciences. 2011; 8(12): 3697-3707. doi:10.5194/bg-8-3697-2011

Frommel A, Maneja R, Lowe D, Malzahn A, Geffen A, Folkvord A, et al. Severe tissue damage in Atlantic cod larvae under increasing ocean acidification. Nat Clim Change. 2011; 2: 42-46. doi:10.1038/nclimate1324

Grosberg R, Cunningham C. Genetic structure in the sea: from populations to communities. In: Bertness M, Gaines S, and Hay M, editors. Marine Community Ecology. Sunderland, MA: Sinauer Associates, Inc.; 2001. pp. 61-84.

Groves C, L Valutis L, Vosick D, Neely B, Wheaton K, Touval J, et al. Designing a geography of hope: a practitioner’s hand book for ecoregional conservation planning 2nd edition. Arlington: The Nature Conservancy; 2000. Available: http://www.denix.osd.mil/nr/upload/Geography-of-hope-handbook-Vol-I-02-136.pdf

Hare, J. and D. Richardson. The use of early life stages in stock identification studies. In: Kerr L, Cardin S, editors. Stock Identification Methods. Waltham: Academic Press. 2014

Hjort, J. Fluctuations in the great fisheries of northern Europe viewed in the light of biological research. Rapports et Proces-verbaux des Reunions. Conseil International pour l'Exploration de la Mer. 1914; 20: 1-228. Available: http://hdl.handle.net/11250/109177

Hoegh-Guldberg O, Mumby P, Hooten A, Steneck R , Greenfield P, Gomez E, et al. Coral reefs under rapid climate change and ocean acidification. Science. 2007; 318(5857): 1737-1742. DOI: 10.1126/science.1152509

Hönisch B, Ridgwell A, Schmidt D, Williams B. The geological record of ocean acidification. Science. 2012; 335: 1058-1063. doi: 10.1126/science.1208277

Houde E. Emerging from Hjort's shadow. J NW Atl Fish Sci. 2008; 41: 53-70. doi:10.2960/J.v41.m634

Jablonski D, Lutz R. Larval ecology of marine benthic invertebrates: Paleobiological implications. Biological Reviews, 1983; 58: 21–89. doi: 10.1111/j.1469-185X.1983.tb00380.x

Jackson J, Kirby M, Berger W, Bjorndal K. Historical overfishing and the recent collapse of coastal ecosystems. Science. 2001; 293(5530): 629-637. DOI:10.1126/science.1059199

Kawaguchi, S., Kurihara H, King R, Hale L, Berli T, Robinson J, et al. Will krill fare well under southern ocean acidification? Biol Lett. 2011; 7(2): 288-291. DOI: 10.1098/rsbl.2010.0777

Kroeker KJ, Kordas RL, Crim R, Hendriks IE, Ramajo L, Singh GS, et al. Impacts of ocean acidification on marine organisms: quantifying sensitivities and interaction with warming. Glob Change Biol. 2013; 19: 1884-1896. doi: 10.1111/gcb.12179

Mooney H, Larigauderie A, Cesario M, Elmquist T, Hoegh-Guldberg O, Lavorel S, et al. Biodiversity, climate change, and ecosystem services. Curr Opin Environ Sustain. 2009; 1: 46-54. doi:10.1016/j.cosust.2009.07.006

Musick J. Criteria to define extinction risk in marine fishes: The American Fisheries Society Initiative. Fisheries. 1999; 24: 6-14. doi: 10.1577/1548-8446(1999)024<0006:CTDERI>2.0.CO;2

Munday P, Dixson D, Donelson J, Jones G, Pratchett M, Devitsina G, et al. Ocean acidification impairs olfactory discrimination and homing ability of a marine fish. Proc Natl Acad Sci. 2009; 106: 1848-1852. doi: 10.1073/pnas.0809996106

Orr J, Fabry V, Aumont O, Bopp L, Doney S, Feely R, et al. Anthropogenic ocean acidification over the twenty-first century and its impact on calcifying organisms. Nature, 2005; 437(7059): 681-686. doi:10.1038/nature04095

Patrick W, Spencer P, Ormseth O, Cope J, Field J, Kobayashi D, et al. Use of productivity and susceptibility indices to determine stock vulnerability, with example applications to six U.S. fisheries. 2009. NOAA Technical Memorandum NMFS-F/SPO-101. 90p. Available: http://spo.nmfs.noaa.gov/tm/TM101.pdf

Pecl GT, Ward TM, Doubleday ZA, Clarke S, Day J, Dixon C, et al. Rapid assessment of fisheries species sensitivity to climate change. Climatic Change, 2014; 127(3-4), 505-520. doi: 10.1007/s10584-014-1284-z

Pineda J, Hare J, and Sponaugle S. Larval transport and dispersal in the coastal ocean and consequences for population connectivity. Oceanography. 2007; 20: 24-41. doi: 10.5670/oceanog.2007.27

Ries J, Cohen A, McCorkle D. Marine calcifiers exhibit mixed responses to CO_2-induced ocean acidification. Geology. 2009; 37(12): 1131-1134. doi: 10.1130/G30210A.1

Rijnsdorp A, Peck M, Engelhard G, Mollmann C, Pinnegar J. Resolving the effect of climate change on fish populations. ICES J Mar Sci. 2009; 66: 1570-1583. doi: 10.1093/icesjms/fsp056

Rose G. Reconciling overfishing and climate change with stock dynamics of Atlantic cod (*Gadus morh*ua) over 500 years. Can J Fish Aquat Sci. 2004; 61(9): 1553-1557. doi: 10.1139/f04-173

Shoji J, Toshito S, Mizuno K, Kamimura Y, Hori M, Hirakawa K. Possible effects of global warming on fish recruitment: shifts in spawning season and latitudinal distribution can alter growth of fish early life stages through changes in daylength. ICES J Mar Sci. 2011; 68(6): 1165-1169. doi: 10.1093/icesjms/fsr059

Spalding M, Fox H, Allen G, Davidson N, Ferdana Z, Finlayson M, et al. Marine ecoregions of the world: a bioregionalization of coastal and shelf areas. Bioscience, 2007; 57(7): 573-583. doi: 10.1641/B570707

Walther G, Post E, Convery P, Menzel A, Parmesan C, Beebee T, et al. Ecological responses to recent climate change. Nature, 2002; 416: 389-95. doi:10.1038/416389a

Wicks L, Roberts J. Benthic invertebrates in a high-CO_2 world. Oceanography and Marine Biology: An Annual Review, 2012; 50: 127-188. doi: 10.1201/b12157-4

Williams S, Shoo L, Isaac J, Hoffman A, Langham G. Towards an integrated framework for assessing the vulnerability of species to climate change. PLoS Biol, 2008; 6: 2621-2626. doi: 10.1371/journal.pbio.0060325
