# Supplementary material for: A Vulnerability Assessment of Fish and Invertebrates to Climate Change on the Northeast U.S. Continental Shelf
Source: PLoS One. 2016 Feb 3;11(2):e0146756. doi: 10.1371/journal.pone.0146756 (PMC4739546; doi:10.1371/journal.pone.0146756)
Supplement: S2 Supporting Information — (PDF) [file pone.0146756.s004.pdf]

S2 Supporting Information. Ocean Currents Exposure

Ocean circulation can be described across multiple spatial scales, from the large thermohaline circulation that moves large water masses between ocean basins, to gyres that circulate around ocean basins, down to regional currents that respond to local wind and density distributions. Most analysis of how climate change will impact ocean circulation centers on impacts to thermohaline circulation. The planet-wide thermohaline circulation is called the global meridional overturning circulation (MOC). Typically, cold, high salinity dense waters sink at high latitudes in the North Atlantic and near Antarctica (Bindoff et al. 2007). This deep water flows south at depth toward the equator where it warms and upwells to the surface and slowly moves back toward high latitudes to replace the cold sinking water. The MOC is often described as a “conveyor belt” that moves large masses of water in consistent patters, but recent analyses suggest this may not be a good descriptor (Lozier 2010). Most climate models suggest that global warming and changes in freshwater input (changes to high latitude rainfall and melting of the Greenland ice sheet) could impact the density distribution of these large water masses and could potentially decrease the MOC (Collins et al. 2013). So far, evidence for changes to the MOC is limited (Bryden et al. 2005, Bindoff et al. 2007).

At the ocean basin scale, the dominant type of flow in the ocean’s mixed layer is gyres. Gyres are a roughly circular collection of upper ocean currents that circulate across large ocean basins (Moran 2011). Gyres (especially tropical gyres) typically have four linked currents: two currents that flow roughly north or south at the western and eastern edges of the basin, and two currents that flow west and east at the top and bottom of the gyres. The western boundary currents are deepest and fastest non-tidal ocean currents on earth and include the Gulf Stream, Kuroshio and Agulhas currents. The currents that make up the gyres are driven by a combination of the Coriolis Effect and wind forcing, and thus rotate clockwise in the northern hemisphere and counterclockwise in the southern hemisphere. Climate change may impact the winds that drive these gyres; changing the speed and/or the extent of the currents. A high-resolution model developed by the Geophysical Fluid Dynamics Laboratory indicates that the Gulf Stream will shift northward and the water mass entering the Northeast U.S. Shelf will switch from cold Labrador Slope water to warm Atlantic slope water (Saba et al. 2015)

Changes to the boundary currents and gyres can also affect the frequency or direction of eddies. Eddies are rings of rotating water that break off from large currents, creating self-contained marine environments that are a different composition from the surrounding waters. Eddies can last for up to eighteen months before finally dissipating and mixing with the surrounding environment (Moran 2011). Increases in wind speed could increase current strength and lead to increases in eddy activity, as has been hypothesized for the Antarctic

circumpolar current (Meridith and Hogg 2006). Characteristics of the water within eddies can vary depending on where they leave the current. For example, eddies that pinch off to the North of the Gulf Stream rotate clockwise and contain warm water with low biological production. Conversely, eddies that pinch off to the South of the Gulf Stream rotate counterclockwise and have high biological production (Moran 2011). An analysis of kinetic energy between years 1993 and 2003 suggests subpolar north Atlantic has had increased eddy activity while the western Pacific Ocean has decreased (Stammer et al. 2006). Increases in eddy variability have been documented for Kuroshio and Gulf Streams (Stammer et al. 2006). Changes to eddies could impact species that depend on eddies for creating the right larval conditions for survival, growth, and transport to appropriate settlement habitats.

Climate change can also impact currents at smaller, more local, spatial scales. Local currents can be divided into three main types: tides, wind stress, and density gradients (Epifiano and Garvine, 2001). Climate change will not impact all of these processes evenly. 1) Tidal currents should experience minimal impact. 2) Currents based on wind stress could be impacted when wind and storm tracks are modified. For example, increases in the westerlies (strong winds between 30-60° N) "alter the flow from oceans to continents and are a major cause of observed changes in winter storm tracks" (Trenberth et al. 2007). 3) Currents based on density gradients may or may not be impacted by climate change, depending on their underlying mechanisms (Epifiano and Garvine, 2001). One density mechanism is the interplay between large water masses at the edge of the continental shelf. Since experts expect minor changes to the boundary currents in the near future (Stouffer et al. 2006), the interplay between water masses at the edge of the continental shelf may change minimally. A second density mechanism is surface heat flux, which should be impacted by the expected increases in sea surface temperature. The final density mechanism is freshwater flow, mostly associated with the output of major rivers. Freshwater flow is tied to precipitation events, and climate models vary in their predictions regarding precipitation. Precipitation is expected to increase in some areas and decrease in others, but all areas are expected to experience an increase in extreme events (Trenberth et al., 2007).

The relative importance of tides, wind stress, or density gradients in influencing local currents varies by region. Tidal processes can be important for some areas, like estuaries and Georges Bank (Epifiano and Garvine, 2001). However, multiple studies from the United States have determined that wind and buoyancy driven flow have the largest impact on marine recruitment, with species that spawn on the mid or outer shelf during fall and winter having an increased importance wind driven flow, while species that spawn on the inner shelf during spring and summer typically dependent on buoyancy driven flow (Epifiano and Garvine, 2001). In general, estuarine species that are dependent on local currents for transport could have

recruitment impacted by changes in storm frequency. However, all changes will not be negative; Atlantic croaker shows a positive correlation between recruitment and hurricane activity (Kerr et al. 2009).

<u>Scoring Exposure to Currents:</u>
Ideally, exposure is scored as the overlap between a species' distribution and the magnitude of the expected climate change. Unfortunately, due to high uncertainty associated with how climate change will impact ocean currents, we do not have maps showing the magnitude of the expected change to ocean currents. We will update this factor as more information becomes available. However, the description provided above suggests a low magnitude of change to tidal currents and large boundary currents, with a higher magnitude of change expected for nearshore currents. Use your expert knowledge of a species' distribution to determine where overlap with currents occurs. Species that have a high overlap with nearshore currents should have a higher exposure score than species that have a high overlap with large boundary currents.

Descriptions of low (1) and high (4) exposure are provided as bookends of a continuum. Use your tallies across all four bins to represent your expert opinion of how much the species will be exposed to changes in currents. For example, a stock is distributed partially within an area with nearshore currents and partially outside this area should be scored between a 1 and 4.

1 Low: Score stocks as low if their distributions overlap almost exclusively with large boundary currents or tidal currents.
4 High: Score stocks as high if their distributions overlap almost exclusively with currents that are expected to have a high magnitude of change such as estuarine circulation, nearshore density and wind driven currents, and/or eddies.

REFERENCES:

Bindoff NL, Willebrand J, Artale V, Cazenave A, Gregory J, Gulev S, et al. 2007: Observations: Oceanic Climate Change and Sea Level. In: Climate Change 2007: The Physical Science Basis. Contribution of Working Group I to the Fourth Assessment Report of the Intergovernmental Panel on Climate Change [Solomon S, Qin D, Manning M, Chen Z, M. Marquis, Averyt KB, et al. editors]. Cambridge: Cambridge University Press. https://www.ipcc.ch/publications_and_data/ar4/wg1/en/ch5.html

Bryden HL, Longworth HR, Cunningham SA. Slowing of the Atlantic Meridional Overturning Circulation at 25oN. Nature 2005; 438: 655-657. doi:10.1038/nature04385

Collins M, Knutti R, Arblaster J, Dufresne JL, Fichefet T, Friedlingstein P, et al. 2013: Long-term Climate Change: Projections, Commitments and Irreversibility. In: Climate Change 2013: The Physical Science Basis. Contribution of Working Group I to the Fifth Assessment Report of the Intergovernmental Panel on Climate Change [Stocker TF, Qin D, Plattner GK, Tignor M, Allen SK, Boschung J, et al. editors]. Cambridge University Press, Cambridge, United Kingdom and New York, NY, USA. http://www.ipcc.ch/pdf/assessment-report/ar5/wg1/WG1AR5_Chapter12_FINAL.pdf

Epifanio CE, Garvine RW. Larval Transport on the Atlantic Continental Shelf of North America: a Review. Estuar Coast Shelf Sci. 2001; 52: 51-77. doi:10.1006/ecss.2000.0727

Kerr LA, Connelly WJ, Martino EJ, Peer AC, Woodland RJ, Secor DH. Climate Change in the U.S. Atlantic Affecting Recreational Fisheries. Rev Fish Sci. 2009; 17: 267-289. doi: 10.1080/10641260802667067

Lozier SM. Deconstructing the Conveyor Belt. Science 2010; 328: 1507-1511. doi: 10.1126/science.1189250

Meredith MP, Hogg AM. Circumpolar Response of Southern Ocean Eddy Activity to a Change in the Southern Annular Mode. Geophys Res Lett. 2006; 33: L16608. doi: 10.1029/2006GL026499

Moran JM. Ocean Studies: Introduction to Oceanography, Third Edition. Boston: American Meteorological Society; 2011.

Saba VS, Griffies SM, Anderson WG, Winton M, Alexander MA, et al. Enhanced warming of the northwest Atlantic Ocean under climate change. J Geophys Res Oceans. 2015; doi:10.1002/2015JC011346

Stammer D, Wunsch C, Ueyoshi K. Temporal changes in ocean eddy transport. J Phys Oceanogr. 2006; 36:543-550. doi: http://dx.doi.org/10.1175/JPO2858.1

Trenberth KE, Jones PD, Ambenje P, Bojariu R, Easterling D, Tank AK, et al. 2007: Observations: Surface and Atmospheric Climate Change. In: Climate Change 2007: The Physical Science Basis. Contribution of Working Group I to the Fourth Assessment Report of the Intergovernmental Panel on Climate Change [Solomon, S., D. Qin, M. Manning, Z. Chen, M. Marquis, K.B. Averyt, et al. editors]. Cambridge: Cambridge University Press. https://www.ipcc.ch/publications_and_data/ar4/wg1/en/ch3.html
