# Supplementary material for: A Vulnerability Assessment of Fish and Invertebrates to Climate Change on the Northeast U.S. Continental Shelf
Source: PLoS One. 2016 Feb 3;11(2):e0146756. doi: 10.1371/journal.pone.0146756 (PMC4739546; doi:10.1371/journal.pone.0146756)
Supplement: S3 Supporting Information — (PDF) [file pone.0146756.s005.pdf]

S3 Supporting Information. Sea-level Rise Exposure

The effects of rising sea levels have implications for the productivity of coastal ecosystems and marine fisheries through coastal erosion, inundation of wetlands, and alterations of salinity regimes (Nicholls et al. 2007). The rate of global sea level rise has increased since the early 20th century and is expected to increase further through the 21st century. The rate of global mean sea level rise was 1.7 mm yr-1 between 1901 and 2010, for a total sea level rise of 19 cm. The rate of global sea level rise increased between 1993 and 2010 to 3.2 mm yr-1 (Church et al. 2013). Across all radiative forcing scenarios considered in the 2013 Intergovernmental Panel on Climate Change Fifth Assessment Report (AR5), by 2081–2100 the rate of global mean sea level rise is expected to be between 2.0 and 15.7 mm/year, with a median rate of 11.2 mm/year under the RCP8.5 scenario (Church et al. 2013). Under the RCP8.5 scenario the 2046-2065 global mean sea level is projected to be above the 1986–2005 level by between 22 and 38 cm (Church et al. 2013).

Recent sea level rise is primarily due the expansion of the warming ocean and the transfer of water currently stored on land to the ocean, particularly from glaciers and ice sheets (Church et al. 2011, Church et al. 2013). Regional sea levels are affected by geographic variability in thermal expansion, vertical land movements (from postglacial rebound, plate tectonics, subsidence, and sedimentation), changes in gravity due to water mass redistribution, changes in wind and air pressure, and other factors (Cazenave and Nerem 2004, Church et al. 2013). For example, the rate of sea level rise along the North American Atlantic coast from Cape Hatteras to Boston was approximately 3 to 4 times higher than the global average between 1950-1979 and 1980-2009 (Sallenger et al. 2012). High rates of sea level rise in the Mid-Atlantic and the Carolinas is primarily due to subsistence, possibly caused by sediment compaction, postglacial rebound, groundwater extraction, and plate tectonics (Parris et al. 2012). In the future, these higher than average rates of sea-level rise are projected to continue along the Northeast U.S. Shelf (Church et al. 2013, their Figure 13.20).

Wetlands are highly sensitive to sea level rise because their location is intimately linked to sea level (Nicholls et al. 2007). If wetlands build vertically at a slower rate than the sea rises, they cannot maintain their elevation relative to sea level and will become submerged during the tide cycle for progressively longer periods (Nicholls et al. 1999). Wetlands experiencing a small tidal range, like those along the Atlantic coast of North America, may be more vulnerable to relative sea level rise than those experiencing a large tidal range (Nicholls et al. 1999, Nicholls 2004). There is some evidence that the phenomenon known as “salt marsh dieback” observed along the east and gulf coasts of the U.S. over the past decade may be associated with sea level rise and changes in precipitation rates (Alber et al. 2008, Kearney and Alexis Riter 2011). McFadden

et al. (2007) estimated that by 2050 22% of global wetlands could be lost with 0.5 m of sea level rise and 32% of global wetlands could be lost with 1 m of sea level rise.

However, coasts are dynamic systems that respond to perturbations, such as storm events (Nicholls et al. 2007). Increased vertical accretion rates through increased sediment and organic matter input may allow coastal wetlands to keep up with rising sea level, but this is a dynamic and non-linear process (Nicholls 2004, Kirwan et al. 2010). There is mixed evidence regarding the changes in wetland soil carbon accretion under elevated CO_2 and warming, suggesting these conditions could increase organic matter productivity but also increase organic decomposition rates (Langley et al. 2009, Kirwan et al. 2010, Kirwan and Blum 2011). For the mid-Atlantic region, an expert assessment of wetland response to sea level rise concluded with a moderate level of confidence that those wetlands keeping pace with 20th century sea level rise would survive a 2 mm/yr acceleration in sea level rise (under optimal hydrology and sediment supply conditions), but would not survive a 7 mm/yr acceleration of sea level rise, which is within the likely range by 2081–2100 under the AR5 scenarios (Cahoon et al. 2009, Church et al. 2013).

The effects of sea level rise on seagrass are also uncertain due to the relative short time scales of seagrass growth dynamics compared to the rates of sea level rise. However, Short and Neckles (1999) estimate that a 0.5 meter increase in water depth from sea level rise could cause a 30% to 40% reduction in seagrass growth due to decreased photosynthesis. Sea level rise may also impact seagrass through increased saltwater intrusion, erosion, sedimentation, increased turbidity with decreases in light transmittance, and changes in tidal variation (Short and Neckles 1999).

Wetlands and seagrass may be able to move inland with sea level rise, but in many areas this cannot occur due to fixed structures on the shoreline. For example, shore protection built to minimize the impact of floods and shore erosion on coastal communities, such as bulkheads, dikes and beachfill, prevents the inland migration of coastal ecosystems (Nicholls 2004, Nicholls et al. 2007, Titus et al. 2009). Titus et al. (2009) estimated that of the land less than one meter above sea level along the U.S. Atlantic coast, almost 60% is developed or likely to be developed, making it unavailable for inland wetland migration, and less than 10% has been set aside for conservation. However, there are varying levels of coastal development along the U.S. Atlantic coast. For example, more than 80% of the land less than one meter above sea level in Florida and from Delaware to Massachusetts is developed or intermediately developed, while only 45% of the land from Georgia to Delaware is developed or intermediately developed (Titus et al. 2009). Also, conservation lands account for most of the Virginia ocean coast, and large parts of the Massachusetts, North Carolina, and Georgia ocean coast potentially mitigating the effect of

sea level rise in these areas because the ecosystem can respond without manmade barriers (Titus et al. 2009).

Scoring Exposure to Sea Level:

Ideally, exposure is scored as the overlap between a species' distribution and the magnitude of the expected climate change. Unfortunately, there is still a large amount of uncertainty regarding future sea level rise and how this will impact coastal habitats. Research suggests that sea level rise with impact wetland, seagrass, and estuary habitats, and biological habitats such as wetland and seagrass will be more impacted than estuarine water habitats. Use your tallies across all four bins to represent your expert knowledge of how reliant the species is on habitats that are expected to be affected by sea level rise. Expert opinion has indicated wetlands would survive a 2 mm/yr acceleration in sea level rise, but would not survive a 7 mm/yr acceleration of sea level rise, so an acceleration of sea level rise at or above this level would indicate a high score (Cahoon et al. 2009). There are regional differences in sea level rise and fixed shoreline structures, as discussed above, so take these factors into consideration when scoring. If no regional estimate are available for expected sea level rise, use the expected global mean sea level rise under the RCP8.5 scenario of approximately 5 to 9.5 mm yr-1 in 2050 (Church et al. 2013).

1. Low: Score stocks as low if they do not rely on wetland, seagrass, or estuary habitat.
2. Moderate: Score stocks as moderate if they do not rely on wetland or seagrass habitat but do rely on estuary habitat.
3. High: Score stocks as high if they rely on wetland or seagrass habitat, and the change in regional sea level within their range is expected to increase less than 7 mm yr-1 by 2050.
4. Very High: Score stocks as very high if they rely on wetland or seagrass habitat for one or more life stage and regional sea level within their range is expected to increase more than 7 mm yr-1 by 2050.

REFERENCES:

Alber M, Swenson EM, Adamowicz SC, Mendelssohn IA. Salt marsh dieback: An overview of recent events in the US. Estuar Coast Shelf Sci. 2008; 80: 1-11. doi:10.1016/j.ecss.2008.08.009

Cahoon DR, Reed DJ, Kolker AS, Brinson MM, Stevenson, J.C., Riggs, S., et al. Coastal wetland sustainability. In Coastal Sensitivity to Sea-Level Rise: A Focus on the Mid-Atlantic Region. Edited by J.G. Titus, K.E. Anderson, D.R. Cahoon, D.B. Gesch, S.K. Gill, B.T. Gutierrez, E.R.

Thieler and S.J. Williams. U.S. Environmental Protection Agency, Washington DC. 2009: pp. 57-72. Available: http://downloads.globalchange.gov/sap/sap4-1/sap4-1-final-report-all.pdf

Cazenave, A, Nerem, RS. Present-day sea level change: Observations and causes. Rev Geophys. 2004; 42(3): RG3001. 10.1029/2003RG000139

Church J, Gregory J, White N, Platten S, and Mitrovica J. 2011. Understanding and Projecting Sea Level Change. Oceanography 2011; 4(2): 130-143. doi: 10.5670/oceanog.2011.33

Church JA, Clark PU, Cazenave A, Gregory JM, Jevrejeva S, Levermann A, et al. Sea Level Change. In Climate Change 2013: The Physical Science Basis. Contribution of Working Group I to the Fifth Assessment Report of the Intergovernmental Panel on Climate Change. Stocker TF, Qin D, Plattner GK, Tignor M, Allen SK, Boschung J, et al. editors. Cambridge: Cambridge University Press; 2013. Available: https://www.ipcc.ch/pdf/assessment-report/ar5/wg1/WG1AR5_Chapter13_FINAL.pdf

Kearney MS, Alexis Riter JC. Inter-annual variability in Delaware Bay brackish marsh vegetation, USA. Wetlands Ecol Manage. 2011; 19(4): 373-388. doi: 10.1007/s11273-011-9222-6

Kirwan ML, Blum LK. Enhanced decomposition offsets enhanced productivity and soil carbon accumulation in coastal wetlands responding to climate change. Biogeosciences 2011; 8(4): 987-993. doi:10.5194/bg-8-987-2011

Kirwan ML, Guntenspergen GR, D'Alpaos A, Morris JT, Mudd SM, Temmerman S. Limits on the adaptability of coastal marshes to rising sea level. Geophys Res Lett. 2010; 37: L23401. doi: 10.1029/2010GL045489.

Langley JA, McKee KL, Cahoon DR, Cherry JA, Megonigal JP. Elevated CO2 stimulates marsh elevation gain, counterbalancing sea-level rise. Proc Natl Acad Sci U S A. 2009; 106(15): 6182-6186. doi: 10.1073/pnas.0807695106

McFadden L, Spencer T, and Nicholls, R.J. Broad-scale modelling of coastal wetlands: what is required? Hydrobiologia 2007; 577(1): 5-15. doi: 10.1007/s10750-006-0413-8

Nicholls RJ. Coastal flooding and wetland loss in the 21st century: changes under the SRES climate and socio-economic scenarios. Glob Environ Change 2004; 14(1): 69-86. doi:10.1016/j.gloenvcha.2003.10.007

Nicholls RJ, Hoozemans FMJ, Marchand M. 1999. Increasing flood risk and wetland losses due to global sea-level rise: regional and global analyses. Glob Environ Change 1999; 9, Supplement 1: S69-S87. doi:10.1016/S0959-3780(99)00019-9

Nicholls RJ, Wong PP, Burkett VR, Codignotto JO, Hay JE, McLean RF, et al. Coastal systems and low-lying areas. In Climate Change 2007: Impacts, Adaptation and Vulnerability. Contribution of Working Group II to the Fourth Assessment Report of the Intergovernmental Panel on Climate Change. Parry ML, Canziani OF, Palutikof JP, van der Linden PJ, Hanson CE, editors. 2007: Cambridge; Cambridge University Press. pp. 315-356. Available: https://www.ipcc.ch/publications_and_data/ar4/wg2/en/ch6.html

Parris A, Bromirski P, Burkett V, Cayan D, Culver M, Hall J, et al. Global Sea Level Rise Scenarios for the United States National Climate Assessment. NOAA Tech Memo OAR CPO-1. 2012. 37pp. available: http://cpo.noaa.gov/sites/cpo/Reports/2012/NOAA_SLR_r3.pdf

Sallenger AH, Doran KS, Howd PA. Hotspot of accelerated sea-level rise on the Atlantic coast of North America. Nat Clim Chang. 2012; 2(12): 884-888. doi:10.1038/nclimate1597

Short FT, Neckles HA. The effects of global climate change on seagrasses. Aquat Bot. 1999; 63(3–4): 169-196. doi:10.1016/S0304-3770(98)00117-X

Titus JG, Hudgens DE, Trescott DL, Craghan M, Nuckols WH, Hershner CH, et al. State and local governments plan for development of most land vulnerable to rising sea level along the US Atlantic coast. Environ Res Lett. 2009; 4(4): 044008. doi:10.1088/1748-9326/4/4/044008
