# Supplementary material for: A Vulnerability Assessment of Fish and Invertebrates to Climate Change on the Northeast U.S. Continental Shelf
Source: PLoS One. 2016 Feb 3;11(2):e0146756. doi: 10.1371/journal.pone.0146756 (PMC4739546; doi:10.1371/journal.pone.0146756)
Supplement: S4 Supporting Information — (PDF) [file pone.0146756.s006.pdf]

S4 Supporting Information. Climate exposure scoring

Scoring of change in mean climate conditions was based on a standard deviate of the modeled future relative to the past. The mean of the past was subtracted from the mean of the future and this difference was divided by the standard deviation of the past. This calculation was mapped by the NOAA Climate Change Web Portal (ESRL 2014) using the Standard Anom (avg historical) statistic. A Very High score was given if the future mean climate was more than 2 standard deviations different than the historical climate.

Scoring of change in the variability in climate conditions was based on an F-test, which divided the variance of the future period by the variance of the historical period. The F-test thresholds were set-up to match the thresholds of the comparison of mean conditions. A Very High score for variance was at the 95% level comparable to the 2 standard deviation threshold used to compare the means.

The historical period was 1956-2005 and the future period was 2006-2055. Conditions were averaged over 5 decades to minimize the influence of natural variability and emphasize the climate change signal. However, the comparison still includes an element of decadal variability.

	Exposure	Approximate Probabilities	$\lvert E_E \rvert$	Exposure Score
**Mean**	Very High	>95.4%	>2	4
	High	86.6-95.4%	1.5-2	3
	Moderate	38.3-86.6%	0.5-1.5	2
	Low	<38.3%	<0.5	1
			**|vES|**	
**Variance**	Very High	>95.4%	>1.78	4
	High	86.6-95.4%	1.54-1.78	3
	Moderate	38.3-86.6%	1.15-1.54	2
	Low	<38.3%	<1.15	1

Earth Systems Research Laboratory. NOAA's Ocean Climate Change Web Portal. 2014. Available: http://www.esrl.noaa.gov/psd/ipcc/ocn/
