# Supplementary material for: A Vulnerability Assessment of Fish and Invertebrates to Climate Change on the Northeast U.S. Continental Shelf
Source: PLoS One. 2016 Feb 3;11(2):e0146756. doi: 10.1371/journal.pone.0146756 (PMC4739546; doi:10.1371/journal.pone.0146756)
Supplement: S5 Supporting Information — (PDF) [file pone.0146756.s007.pdf]

S5 Supporting Information. Assessment Results

Two comma-delimited ascii files are included: S1 Dataset (Vulnerability Results) and S2 Dataset (Directional Effects Results).

S1 Dataset (Vulnerability Results)
1969 rows
3 columns
Delimiter: ,
Header Rows: 1
Rows are:

Results for each species and attribute (82 x 24 = 1968 rows + 1 header row)

Columns are:

Species name - Common Name as listed in Table 1
Species functional group - as listed in Table 1
Attribute name - as listed in Table 2
Attribute category - as listed in Table 2: Climate Exposure or Biological Sensitivity
Low - total number of expert tallies received for that species and that attribute
Moderate - total number of expert tallies received for that species and that attribute
High - total number of expert tallies received for that species and that attribute
Very - total number of expert tallies received for that species and that attribute

Note: Sensitivity Attributes have a total of 25 tallies (5 experts, 5 tallies each), Climate Exposure Attributes has a total of 20 tallies (4 experts, 5 tallies)

S2 Dataset (Directional Effects Results)

83 rows

5 columns

Delimiter: ,

Header Rows: 1

Rows are:

results for each species (82 rows + 1 header row

Columns are

Species name - Common Name as listed in Table 1

Species functional group - as listed in Table 1

Negative - total number of expert tallies received for that species

Neutral - total number of expert tallies received for that species

Positive - total number of expert tallies received for that species

Note: Directional Effect scores have a total of 12 tallies (3 experts, 4 tallies each)
