# Supplementary material for: A Vulnerability Assessment of Fish and Invertebrates to Climate Change on the Northeast U.S. Continental Shelf
Source: PLoS One. 2016 Feb 3;11(2):e0146756. doi: 10.1371/journal.pone.0146756 (PMC4739546; doi:10.1371/journal.pone.0146756)
Supplement: S6 Supporting Information — (PDF) [file pone.0146756.s008.pdf]

S6 Supporting Information. Data Quality

Definitions used by experts to score data quality for each biological sensitivity attribute and climate exposure factor. Each expert scored data quality independently resulting in 5 data quality scores for each sensitivity attribute and 4 data quality scores for each climate exposure factor.

Data Quality Score	Description
3	**Adequate Data**. The score is based on data which have been observed, modeled or empirically measured for the species in question and comes from a reputable source.
2	**Limited Data**. The score is based on data which has a higher degree of uncertainty. The data used to score the attribute may be based on related or similar species, come from outside the study area or the reliability of the source may be limited.
1	**Expert Judgement**. The attribute score refelects the expert judgement of the reviewer and is based on their general knowledge of the species, or related species, and their relative role on the ecosystem.
0	**No Data**. No information to base an attribute score on. Very little is known about the species or related species and there is no basis for forming an expert opinion.
