# Supplementary material for: A Vulnerability Assessment of Fish and Invertebrates to Climate Change on the Northeast U.S. Continental Shelf
Source: PLoS One. 2016 Feb 3;11(2):e0146756. doi: 10.1371/journal.pone.0146756 (PMC4739546; doi:10.1371/journal.pone.0146756)
Supplement: S7 Supporting Information — (PDF) [file pone.0146756.s009.pdf]

S7 Supporing Information. Species Narratives

Table of Contents

Acadian Redfish 1
Alewife 4
American Eel 9
American Lobster 13
American Plaice 17
American Shad 20
Anchovies 24
Atlantic Cod 27
Atlantic Croaker 31
Atlantic Hagfish 35
Atlantic Halibut 38
Atlantic Herring 41
Atlantic Mackerel 45
Atlantic Menhaden 48
Atlantic Salmon 52
Atlantic Saury 56
Atlantic Sea Scallop 59
Atlantic Sturgeon 62
Atlantic Surfclam 66
Atlantic Wolfish 69
Barndoor Skate 73
Bay Scallop 76
Black Sea Bass 79
Bloodworm 82
Blue Crab 85
Blue Mussel 89
Blueback Herring 93
Bluefish 97
Butterfish 101

Cancer Crabs 104
Channeled Whelk 107
Clearnose Skate 110
Conger Eel 113
Cusk 116
Deep-sea Red Crab 119
Dusky Shark 122
Eastern Oyster 126
Green Sea Urchin 130
Haddock 133
Hickory Shad 136
Horseshoe Crab 139
Knobbed Whelk 142
Little Skate 145
Longfin Inshore Squid 148
Monkfish (Goosefish) 152
Northern Kingfish 155
Northern Quahog 158
Northern Shortfin Squid 161
Northern Shrimp 164
Ocean Pout 168
Ocean Quahog 171
Offshore Hake 174
Pollock 177
Porbeagle 181
Rainbow Smelt 184
Red Drum 188
Red Hake 191
Rosette Skate 194
Sand Lances 197
Sand Tiger 200
Scup 203
Shortnose Sturgeon 206

Return to Table of Contents

Silver Hake 210
Smooth Dogfish 213
Smooth Skate 217
Softshell Clam 220
Spanish Mackerel 224
Spiny Dogfish 227
Spot 230
Spotted Seatrout 233
Striped Bass 236
Summer Flounder 240
Tautog 244
Thorny Skate 248
Tilefish 251
Weakfish 255
White Hake 258
Windowpane 261
Winter Flounder 264
Winter Skate 268
Witch Flounder 271
Yellowtail Flounder 274

Acadian Redfish – *Sebastes fasciatus*

Overall Vulnerability Rank = Moderate

Biological Sensitivity = Moderate
Climate Exposure = High
Data Quality = 83% of scores ≥ 2

Sebastes fasciatus	Expert Scores	Data Quality	Expert Scores Plots (Portion by Category)
Stock Status	2.0	2.8	
Other Stressors	1.9	2.2	
Population Growth Rate	3.5	2.4	
Spawning Cycle	2.6	2.8	
Complexity in Reproduction	1.9	1.2	
Early Life History Requirements	2.2	2.0	
Sensitivity to Ocean Acidification	1.1	0.8	
Prey Specialization	2.0	3.0	
Habitat Specialization	1.5	3.0	
Sensitivity to Temperature	2.2	3.0	
Adult Mobility	2.8	2.0	
Dispersal & Early Life History	1.8	2.8	
Sensitivity Score	Moderate		
Sea Surface Temperature	3.9	3.0	
Variability in Sea Surface Temperature	1.0	3.0	
Salinity	1.1	3.0	
Variability Salinity	1.2	3.0	
Air Temperature	1.0	3.0	
Variability Air Temperature	1.0	3.0	
Precipitation	1.0	3.0	
Variability in Precipitation	1.0	3.0	
Ocean Acidification	4.0	2.0	
Variability in Ocean Acidification	1.0	2.2	
Currents	2.1	1.0	
Sea Level Rise	1.1	1.5	
Exposure Score	High		
Overall Vulnerability Rank	Moderate		

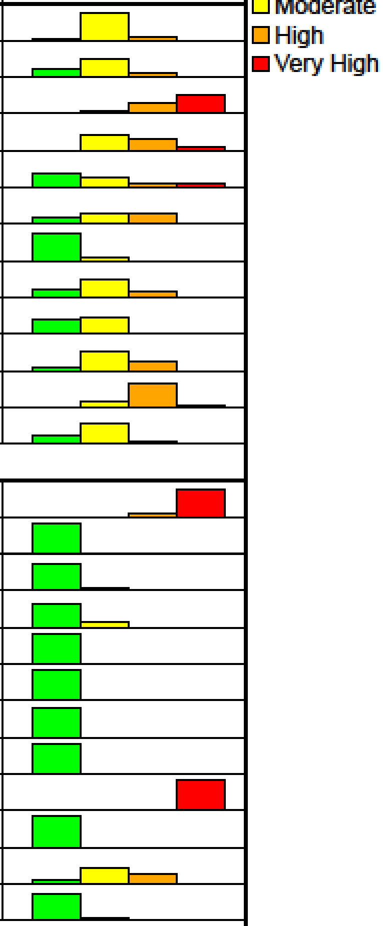

Acadian Redfish (*Sebastes fasciatus*)

Overall Climate Vulnerability Rank: **Moderate** (94% certainty from bootstrap analysis).

Climate Exposure: **High**. Two exposure factors contributed to this score: Ocean Surface Temperature (3.9) and Ocean Acidification (4.0). All life stages of Acadian Redfish use marine habitats.

Biological Sensitivity: **Moderate**. Three sensitivity attributes scored above 2.5: Population Growth Rate (3.5), Spawning Cycle (2.6), Adult Mobility (2.8). Acadian Redfish are long-lived and slow growing and adults are relatively sedentary and site-attached. Fertilization occurs internally during the late spring (Klein-MacPhee and Collette, 2002).

Distributional Vulnerability Rank: **High** (97% certainty from bootstrap analysis).

Directional Effect in the Northeast U.S. Shelf: The effect of climate change on Acadian Redfish on the Northeast U.S. Shelf is very likely to be negative (>95% certainty in expert scores). Warmer temperatures are associated with a decrease in productivity. In addition, Acadian Redfish is a cold water species and continued warming will likely limit habitat.

Data Quality: 88% of the data quality scores were 2 or greater indicate that data quality is moderate.

Climate Effects on Abundance and Distribution: Working across the Northwest Atlantic, Devine and Haedrich (2011) reported that a mix of exploitation and environmental variability accounted for observed trends in abundance. They also found that the relative importance of exploitation and environment differed among stocks. Population productivity in the Northeast U.S. Shelf is related to climate conditions; recruitment anomalies increase with increasing NAO (Brodziak and O'Brien, 2005). A positive NAO is associated with colder temperatures in the Gulf of Maine (Hare and Kane, 2005). Murawski (1993) and Nye et al. (2009) reported that Acadian Redfish distributions have not changed in response to temperature but have changed in response to population abundance.

Life History Synopsis**:** Acadian Redfish is a long-lived, slow-growing, benthic, marine species occurring from Iceland to Virginia (Klein-MacPhee and Collette, 2002). Acadian Redfish is difficult to separate morphologically from the Deepwater Redfish (*S. mentella*), and so are often treated together (Klein-MacPhee and Collette, 2002). Species of the *Sebastes* genus, including Acadian Redfish, are mature between 5 and 9 years and are fairly unique in being ovoviviparous with a long-duration pelagic phase (Pikanowski et al., 1999; Klein-MacPhee and Collette, 2002). Copulation likely occurs from October to January, fertilization is internal and delayed until February to April, and larvae are released near the end of the yolk-sac stage from April-August (Pikanowski et al., 1999). Larvae and early juveniles of the genus are pelagic (above the thermocline till 25 mm, below the thermocline from 25-50 mm) for 4-5 months (Pikanowski et al., 1999). While pelagic, Acadian Redfish feed during the day on copepods, euphausiids, and fish and invertebrate eggs (Pikanowski et al., 1999). Larger Acadian Redfish and Atlantic Halibut are the main predators of young Acadian Redfish (Pikanowski et al., 1999). Larvae metamorphose by 19 mm standard length and settle to the bottom (25-400 m) at approximately 50 mm standard length (Pikanowski et al., 1999; Klein-MacPhee and Collette, 2002). As adults, Acadian Redfish prefer cool, deepwater habitats (< 13 ^{o}C, 128-592 m), but are also found in Gulf of Maine shoal waters (Pikanowski et al., 1999; Klein-MacPhee and Collette, 2002). While Acadian Redfish probably have no preference for bottom type, they are rarely collected from sandy bottom, may rely on boulders and anemones for cover, and are nearly sessile (Pikanowski et al., 1999). Juveniles and adults of the genus feed on

euphausiids, mysids, and bathypelagic fish at night, rising off the bottom to follow prey (Pikanowski et al., 1999). Predators of redfish include any larger piscivorous fish including Monkfish (Goosefish), Atlantic Cod, Pollock, and Atlantic Wolfish (Pikanowski et al., 1999). Acadian Redfish are managed under the New England Fisheries Management Council's Northeast Multispecies Management Plan and are considered rebuilt, neither overfished nor experiencing overfishing (NEFMC, 2014).

Literature Cited:

Brodziak J, O'Brien L. Do environmental factors affect recruits per spawner anomalies of New England groundfish?. ICES J Mar Sci. 2005; 62(7): 1394-1407. DOI:10.1016/j.icesjms.2005.04.019

Devine JA, Haedrich RL. The role of environmental conditions and exploitation in determining dynamics of redfish (*Sebastes* species) in the Northwest Atlantic. Fisher Oceanogr. 2011; 20(1): 66-81. DOI: 10.1111/j.1365-2419.2010.00566.x

Hare JA, Kane J. Zooplankton of the Gulf of Maine—a changing perspective. Amer Fish Soc Symp. 2012; 79: 115-137.

Klein-MacPhee G, Collette BB. Acadian Redfish/*Sebastes fasciatus* Storer 1854. In: Collette, B. B. and G. Klein-MacPhee eds. Bigelow and Schroeder's fishes of the Gulf of Maine. 3rd Edition. Washington, DC: Smithsonian Institution Press. 2002; 334-338.

Murawski SA. Climate change and marine fish distributions: forecasting from historical analogy. Trans Amer Fish Soc. 1993; 122(5): 647-658. DOI: 0.1577/1548-8659(1993)122<0647:CCAMFD>2.3.CO;2

New England Fishery Management Council (NEFMC). Status, Assessment and Management Information for NEFMC Managed Fisheries. 2014. Accessed online (August 2015): http://www.nefmc.org/press/council_discussion_docs/Publish/April%202014/Status%20Assessment%20and%20Management%20Information%20APR%202014.pdf

Nye JA, Link JS, Hare JA, Overholtz WJ. Changing spatial distribution of fish stocks in relation to climate and population size on the Northeast United States continental shelf. Mar Ecol Prog Ser. 2009; 393: 111-129. DOI: 0.3354/meps08220

Pikanowski RA, Morse WW, Berrien PL, Johnson DL, McMillan DG. Essential fish habitat source document: Redfish, *Sebastes* spp., life history and habitat characteristics. NOAA Tech Memo NMFS NE 1999; 132: 19 p. Accessed Online (August 2015): http://www.nefsc.noaa.gov/publications/tm/tm132/tm132.pdf

Return to Table of Contents

Alewife – *Alosa pseudoharengus*

Overall Vulnerability Rank = Very High

Biological Sensitivity = High
Climate Exposure = Very High
Data Quality = 79% of scores ≥ 2

Alosa pseudoharengus	Expert Scores	Data Quality	Expert Scores Plots (Portion by Category)
Stock Status	2.5	1.4	
Other Stressors	3.3	2.2	
Population Growth Rate	2.2	1.4	
Spawning Cycle	3.2	2.9	
Complexity in Reproduction	3.2	3.0	
Early Life History Requirements	3.3	2.4	
Sensitivity to Ocean Acidification	1.5	1.8	
Prey Specialization	1.5	3.0	
Habitat Specialization	2.6	3.0	
Sensitivity to Temperature	2.0	3.0	
Adult Mobility	1.6	2.8	
Dispersal & Early Life History	2.8	2.6	
Sensitivity Score	High		
Sea Surface Temperature	4.0	3.0	
Variability in Sea Surface Temperature	1.0	3.0	
Salinity	1.7	3.0	
Variability Salinity	1.2	3.0	
Air Temperature	4.0	3.0	
Variability Air Temperature	1.0	3.0	
Precipitation	1.3	3.0	
Variability in Precipitation	1.4	3.0	
Ocean Acidification	4.0	2.0	
Variability in Ocean Acidification	1.0	2.2	
Currents	2.0	1.0	
Sea Level Rise	2.8	1.5	
Exposure Score	Very High		
Overall Vulnerability Rank	Very High		

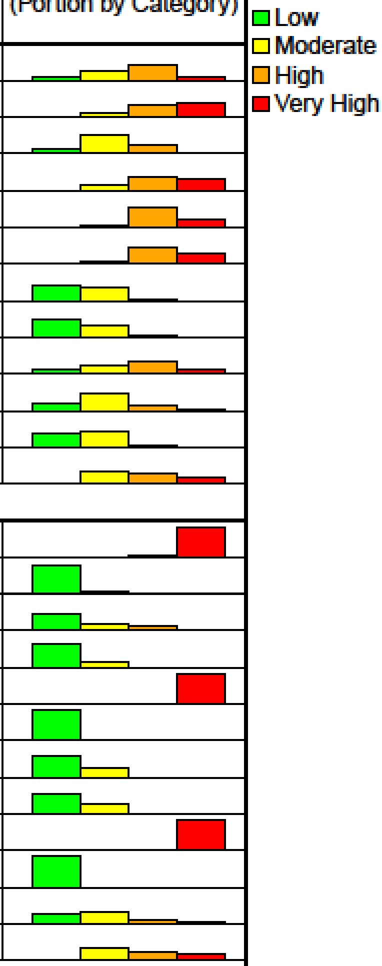

Alewife (*Alosa pseudoharengus*)

Overall Climate Vulnerability Rank: **Very High** (100% certainty from bootstrap analysis).

Climate Exposure: **Very High**. Three exposure factors contributed to this score: Ocean Surface Temperature (4.0), Ocean Acidification (4.0) and Air Temperature (4.0). Alewife are anadromous, spawning in freshwater, developing in freshwater and estuarine habitats, feeding as adults in marine habitats.

Biological Sensitivity: **High**. Four sensitivity attributes scored above 3.0: Other Stressors (3.3), Early Life History Requirements (3.3), Spawning Cycle (3.2), Complexity in Reproduction (3.2). Alewife are anadromous and exposed to a number of other stressors including habitat destruction, blockage to spawning habitats, and contaminants (Limburg and Waldman, 2009). Spawning time varies latitudinally and is linked to spring warming (Monroe, 2002). Eggs and larvae inhabit freshwaters and then juveniles move to estuarine and ocean waters.

Distributional Vulnerability Rank: **Low** (62% certainty from bootstrap analysis). Alewife have a relatively high degree of spawning site fidelity, limiting the ability of the species to shift distribution.

Directional Effect in the Northeast U.S. Shelf: The effect of climate change on Alewife is likely to be negative (90-95% certainty in expert scores). Climate change will probably cause marine distributions to continue to shift, thereby causing longer migrations to natal rivers. Changes in rivers from increased precipitation and warming may cause decreases in productivity particularly in the southern portion of the Northeast U.S. shelf. The effect of ocean acidification over the next 30 years is likely to be minimal.

Data Quality: 79% of the data quality scores were 2 or greater indicate that data quality is moderate.

Climate Effects on Abundance and Distribution: Alewife productivity and distribution are susceptible to climate change. Tommasi et al. (2015) indicated that recruitment was affected by stream temperatures and river flow, both of which will be impacted by climate change. A number of other components of Alewife physiology and ecology are affected by temperature (Kellogg, 1982; Ellis and Vokoun, 2009) and other climate factors such as changes in streamflow and sea-level rise may also affect Alewife (NMFS, 2012). Distribution in the marine stage is also affected by temperature. Nye et al. (2009) found large shifts in the distribution of Alewife at sea and Lynch et al. (2014) developed projections of the change in the distribution of thermal habitat. However, natal homing is an important element in Alewife life history, thus the marine distribution may be changing faster than the spawning distribution.

Life History Synopsis**:** Alewife is an anadromous species, meaning adults migrate from estuarine and marine feeding habitat to freshwater spawning and nursery habitat. Alewife are iteroparious; adults can return to rivers in multiple years to spawn. Alewife occurs from Newfoundland to Florida, but individuals are predominantly found from Nova Scotia to Virginia (Munroe, 2002). The stock is contiguous range wide, but three regional genetic groupings have been documented: Northern New England, Southern New England, and Mid Atlantic (Palkovacs et al., 2013; NMFS, 2013). Alewife reach sexual maturity after 3-5 years for males and 4-6 years for females, and some portion of the population are repeat spawners (Loesch, 1987; Munroe, 2002). The spawning migration begins in early spring through August, varying by latitude and affected by water temperature (Monroe, 2002). Spawning occurs in slow moving freshwater and lasts a few days before spent adults move downstream (Munroe, 2002). Migration distances vary by river, but movement upstream is influenced by light intensity, water

flow, and temperature (Munroe, 2002). Eggs are demersal for the first several hours, but become less adhesive during hardening and are eventually pelagic (Munroe, 2002). Eggs and larvae can survive a range of salinities, but prefer freshwater (Loesch, 1987; Klauda et al., 1991; Wang and Kernehan, 1979). The egg stage lasts less than a week; during which time, the egg and yolk-sac-stage larvae drift downstream to slower-moving water with cool to mild temperatures (Wang and Kernehan, 1979; Munroe, 2002). Feeding begins within a week of hatching (Munroe, 2002). Larvae are selective feeders on cladocerans and copepods adding larger specimens as they grow (Nigro and Ney, 1982; Stone and Jessop, 1992; Munroe, 2002). Transformation to the juvenile stage occurs a little over a month after hatching, but juveniles remain in slow-moving, freshwater and estuarine nursery habitat until the summer or autumn migration to sea (Bigelow and Shroeder, 1953; Jones et al., 1978; Wang and Kernehan, 1979; Munroe, 2002). Juveniles are opportunistic feeders on seasonally available zooplanktonic and benthic organisms such as insects, amphipods, ostracods, and oligochaete worms (Watt and Duerden, 1974; Gregory et al., 1983; Grabe, 1996). Alewife adults can occur in landlocked freshwater systems, but are most often found from nearshore estuarine to offshore marine habitats (Munroe, 2002; Nye et al., 2013). This pelagic schooling species uses diel vertical migrations to follow zooplankton such as copepods, amphipods, and chaetognaths, and small fishes (Bowman et al., 2000, Monroe, 2002). Adults are most common in areas with cool bottom temperatures in a wide range of salinities (Munroe, 2002; Nye et al., 2013). Alewife at all stages is important prey for fish, birds, amphibians, reptiles and mammals (Klauda et al., 1991, Monroe, 2002). Alewife abundance has been in decline throughout the species' range for decades likely caused by heavy fishing and habitat destruction (Haas-Castro, 2006). As a result, NMFS has declared Alewife a Species of Concern, several states have declared fishing moratoriums, and efforts are being made to restore access to spawning and nursery habitat throughout the range (ASMFC, 2012).

Literature Cited:

Atlantic States Marine Fisheries Commission (ASMFC). 2012. River Herring Benchmark Stock Assessment Volume II. Stock Assessment Report No. 12-02 of the Atlantic States Marine Fisheries Commission. Available: http://www.asmfc.org/uploads/file/riverHerringBenchmarkStockAssessmentVolumeIIR_May2012.pdf

Bigelow HB, Schroeder WC. 1953. Fishes of the Gulf of Maine. U.S. Fish and Wildlife Service. Available: http://www.gma.org/fogm/

Bowman RE, Stilwell CE, Michaels WL, Grosslein MD. 2000. Food of northwest Atlantic fishes and two common species of squid. NOAA Technical Memorandum. NMFS-NE-155. Available: http://www.nefsc.noaa.gov/publications/tm/tm155/tm155.pdf

Ellis D, Vokoun JC. Earlier spring warming of coastal streams and implications for alewife migration timing. N Am J Fish Manage. 2009; 29(6): 1584-1589. doi: 10.1577/M08-181.1

Grabe SA. Feeding chronology and habits of Alosa spp. (Clupeidae) juveniles from the lower Hudson River estuary, New York. Environ Biol Fishes. 1996; 47: 321-326. doi: 10.1007/BF00000504

Gregory RS,Brown GS, Danborn GR. Food habits of young anadromous alewife, Alosa pseudoharengus, in Lake Ainslie, Nova Scotia. Canadian Field Naturalist. 1983; 97: 423-426. http://www.biodiversitylibrary.org/item/89042#page/7/mode/1up

Jones PW, Martin FD, Hardy Jr. JD. Development of fishes of the Mid-Atlantic Bight: an atlas of the egg, larval and juvenile stages, Volume I. U.S. Fish and Wildlife Service Program. FWS/OBS-78/12. 1978; 366 pp . http://docs.lib.noaa.gov/rescue/NOAA_E_DOCS/Coastal_Services_Center_docs/Development%20of%20Fishes%20of%20the%20Mid-Atlantic%20Bight%20-%20An%20Atlat%20of%20Eg/

Kellogg RL. Temperature requirements for the survival and early development of the anadromous alewife. The Progressive Fish-Culturist. 1982; 44(2): 63-73. doi: 10.1577/1548-8659(1982)44[63:TRFTSA]2.0.CO;2

Klauda RJ, Fischer SA, Hall Jr. LW, Sullivan JA. Alewife and blueback herring Alosa pseudoharengus and Alosa aestivalis. In: Funderburk SL, Mihursky JA, Jordan SJ, Riley D, editors. Habitat requirements for Chesapeake Bay living resources, 2nd ed. Annapolis: Living Resources Subcommittee, Chesapeake Bay Program; 1991. pp. 10.1-10.29.

Limburg KE, Waldman JR. Dramatic declines in North Atlantic diadromous fishes. BioScience. 2009; 59(11): 955-965. doi: 10.1525/bio.2009.59.11.7

Loesch JG. Overview of life history aspects of anadromous alewife and blueback herring in freshwater habitats. In: Dadswell MJ, Klauda RJ, Moffitt CM, Saunders RL, editors. Common strategies of anadromous and catadromous fishes. Bethesda: American Fisheries Society, Symposium 1; 1987. p. 89-103.

Lynch PD, Nye JA, Hare JA, Stock CA, Alexander MA, Scott JD, et al. Projected ocean warming creates a conservation challenge for river herring populations. ICES J Mar Sci. 2015; 72(2): 374-387. doi: 10.1093/icesjms/fsu134

Munroe T. Herring and herring-like fishes: Order Clupeiformes. In: B.B. Collette BB, Klein-MacPhee G, editors, Fishes of the Gulf of Maine, 3rd ed. Washington: Smithsonian Institution Press; 2002. p. 104-158.

National Marine Fisheries Service (NMFS). 2012. River Herring Climate Change Workshop Report. Greater Atlantic Fishery Regional Office, Gloucester, Massachusetts, 60p. Available: http://www.greateratlantic.fisheries.noaa.gov/prot_res/CandidateSpeciesProgram/sswpdocs/RIVER%20HERRING%20CLIMATE%20CHANGE%20WORKSHOP%20REPORT_122712.pdf

National Marine Fisheries Service (NMFS). 2013.Endangered and Threatened Wildlife and Plants; Endangered Species Act Listing Determination for Alewife and Blueback Herring. Federal Register Notice. 78: 48944. 52pp. Available: http://www.nero.noaa.gov/regs/2013/August/13riverherringlistingesanoticefr.pdf

Nigro AA, Ney JJ. Reproduction and early life accommodations of landlocked alewife to a southern range extension. Trans Am Fish Soc. 1982; 96: 387-393. doi: 10.1577/1548-8659(1982)111<559:RAEAOL>2.0.CO;2

Nye JA, Lynch P, Alexander M, Stock C, Hare J. 2012. Results of Preliminary Analysis of the Effect of Climate Change on River Herring. Fisheries and the Environment (FATE) Annual Report. 17pp. Available: http://www.nefsc.noaa.gov/epd/ocean/MainPage/Climate_and_river_herring_summary.pdf

Palkovacs EP, Hasselman DJ, Argo EE, Gephard SR, Limburg KE, Post DM, et al. Combining genetic and demographic information to prioritize conservation efforts for anadromous alewife and blueback herring. Evol Appl. 2013; 7(2): 212–226. doi: 10.1111/eva.12111

Stone HH, Jessop BM. Seasonal distribution of river herring Alosa pseudoharengus and A. aestivalis off the Atlantic coast of Nova Scotia. Fish Bull. 1992; 90: 376-389. Available: http://fishbull.noaa.gov/902/stone.pdf

Tommasi D, Nye J, Stock C, Hare JA, Alexander M, Drew K. Effect of Environmental Conditions on Juvenile Recruitment of Alewife (Alosa pseudoharengus) and Blueback Herring (A. aestivalis) in fresh water: a coastwide Perspective. Can J Fish Aquat Sci. 2015; 72(7): 1037-1047. doi: 10.1139/cjfas-2014-0259

Wang JCS, Kernehan RJ. Fishes of Delaware estuaries: A guide to early life histories. Towson, MD: Ecological Analysts, Inc.; 1979. 307 p.

Watt WD, Duerden FC. Aquatic ecology of the Saint John River. Fredericton, New Brunswick: The Saint John River Basin Board Volume 2, Report No. 15g. 1974.

Return to Table of Contents

American Eel – *Anguilla rostrata*

Overall Vulnerability Rank = High

Biological Sensitivity = Moderate
Climate Exposure = Very High
Data Quality = 71% of scores ≥ 2

Anguilla rostrata	Expert Scores	Data Quality	Expert Scores Plots (Portion by Category)
Stock Status	2.7	1.0	
Other Stressors	2.8	1.7	
Population Growth Rate	2.8	1.8	
Spawning Cycle	2.5	2.2	
Complexity in Reproduction	2.7	1.9	
Early Life History Requirements	2.6	1.2	
Sensitivity to Ocean Acidification	1.1	2.0	
Prey Specialization	1.1	3.0	
Habitat Specialization	2.6	3.0	
Sensitivity to Temperature	1.3	3.0	
Adult Mobility	1.2	3.0	
Dispersal & Early Life History	1.1	2.6	
Sensitivity Score	Moderate		
Sea Surface Temperature	4.0	3.0	
Variability in Sea Surface Temperature	1.0	3.0	
Salinity	1.6	3.0	
Variability Salinity	1.2	3.0	
Air Temperature	4.0	3.0	
Variability Air Temperature	1.0	3.0	
Precipitation	1.3	3.0	
Variability in Precipitation	1.4	3.0	
Ocean Acidification	4.0	2.0	
Variability in Ocean Acidification	1.0	2.2	
Currents	2.4	1.0	
Sea Level Rise	2.6	1.5	
Exposure Score	Very High		
Overall Vulnerability Rank	High		

Low
Moderate
High
Very High

American Eel (*Anguilla rostrata*)

Overall Climate Vulnerability Rank: **High** (95% certainty from bootstrap analysis).

Climate Exposure: **Very High**. Three exposure factors contributed to this score: Ocean Surface Temperature (4.0), Ocean Acidification (4.0) and Air Temperature (4.0). American Eel are semelparous and catadromous: spawning in the ocean, developing in marine, estuarine, and freshwater water habitats, then feeding growing, and maturing in freshwater.

Biological Sensitivity: **Moderate**. Seven sensitivity attributes scored above 2.5, but none above a 3.0: Other Stressors (2.8), Population Growth Rate (2.8), Complexity in Reproduction (2.7), Stock Status (2.7), Early Life History Requirements (2.6), Habitat Specialization (2.6), and Spawning Cycle (2.5). American Eel are catadromous and exposed to a number of other stressors including habitat destruction, blockage to spawning habitats, and contaminants (Limburg and Waldman, 2009). In Northeast U.S. Shelf Ecosystem, the species is at or near historically low levels due to a combination of historical overfishing, habitat loss, food web alterations, predation, turbine mortality, environmental changes, toxins and contaminants, and disease (ASMFC, 2012). American Eel are relatively late maturing, migrate to specific areas in the Sargasso Sea to spawn, specialized larval stages – leptocephalia – disperse and enter river systems coastwide while undergoing metamorphosis.

Distributional Vulnerability Rank: **High** (100% certainty from bootstrap analysis). Two attributes indicated vulnerability to distribution shift. American Eel larvae are widely dispersed over much of the North Atlantic and adults are highly mobile returning to the Sargasso Sea from river systems along most of the North American coast. However, the ability for individual American Eel to shift distribution once in freshwater habitats is limited.

Directional Effect in the Northeast U.S. Shelf: The effect of climate change on American Eel is estimated to be neutral, but this estimate has uncertainty (66-90% certainty in expert scores). American Eel is found in a range of habitats and it is unclear the effect of climate change on population productivity and distribution. However, American Eel spawn in the Sargasso Sea, so shifts in the Gulf Stream have the potential to effect larval dispersal, survival, and potentially recruitment, but the direction and magnitude of these effects are unclear. The effect of ocean acidification over the next 30 years is likely to be minimal.

Data Quality: 71% of the data quality scores were 2 or greater indicate that data quality is moderate.

Climate Effects on Abundance and Distribution: Correlations have been found between American Eel recruitment and climate factors including the North Atlantic Oscillation, ocean temperatures, frontal locations, and ocean currents (see Friedland et al., 2007; Miller and Casselman, 2014). Castonguay et al. (1994) identified synchronous declines in American and European Eel recruitment and indicated basin-scale processes were the likely cause, specifically identifying climate-driven changes in ocean circulation. Bonhommeau et al. (2008) proposed that decreases in oceanic primary production through climate-driven processes has caused a reduction in recruitment of Anguillid species worldwide, including the American Eel. Sullivan et al. (2006) found glass eel (a young stage before pigmentation forms) abundance entering estuaries was related to winter precipitation and hypothesized that increased freshwater flow into the coastal ocean enhanced detection by returning glass eels. Thus, climate factors operating on multiple scales will likely effect the productivity of American Eel. The American Eel occurs throughout the eastern United States stretching south through Central America and the Caribbean and

north through Atlantic Canada and Greenland (Miller Casselman, 2014), thus changes in distribution, if they occur, will have little impact in the Northeast U.S. Shelf Ecosystem, which is in the north central part of their range.

Life History Synopsis**:** The life history of the American Eel differs from almost all other fish found in the United States. Eels are among the few catadromous species, meaning they live in freshwater but reproduce in salt water (McCleave, 2001; Cairns et al., 2005). They range from Venezuela to Greenland and all come from the same genetic stock (panmictic; Avise, 2003). They aggregate in the Sargasso Sea to spawn, and the spawning site varies depending on environmental conditions (McCleave, 2001; Cairns et al., 2005). After spawning, eggs hatch into leptocephali – long, flat, leaf-like larvae – that float like plankton. They drift with the currents for about a year feeding off detritus and marine snow before metamorphosing into glass eels and moving into coastal areas (McCleave, 2001; Cairns et al., 2005). Glass eels are transparent, around 2-3 inches long, and gain yellow/green pigmentation as they migrate up river and grow (McCleave, 2001; Cairns et al., 2005). Most of the eel's life is spent in fresh or brackish waters where it feeds on an assortment of foods including crustaceans, fish, insect larvae, and plankton (McCleave, 2001; Cairns et al., 2005). When eels reach and appropriate size and have enough stores of fat (3-24 years old) they metamorphose into silver eels and begin the long migration back to the Sargasso Sea to complete their life cycle (McCleave, 2001; Cairns et al., 2005). Eels have a very plastic life history strategy that allows individuals to adapt to a broad diversity of habitats. Due to the panmictic nature of the species, there is currently no estimate of total abundance or population status. In 2010, the U.S. Fish and Wildlife Service received a petition to list the American Eel as threatened under the Endangered Species Act (CESAR, 2010). The The U.S. Fish and Wildlife Service found information indicates that the petition "may be warranted by a causal link between oceanic changes … and decreasing glass eel recruitment (USFWS, 2011)."

Literature Cited:

Atlantic States Marine Fisheries Commission (ASMFC). 2012. American Eel Benchmark Stock Assessment. Stock Assessment Report No. 12-01. Available: http://www.asmfc.org/uploads/file/americanEelBenchmarkStockAssessmentReport_May2012.pdf

Bonhommeau S, Chassot E, Planque B, Rivot E, Knap A, Le Pape O. Impact of climate on eel populations of the Northern Hemisphere. Mar Ecol Prog Ser. 2008; 373: 71-80. doi: 10.3354/meps07696

Cairns, D., V. Tremblay, J. Casselman, F. Caron, G. Verreault, Y. Mailhot, P. Dumont, R. Bradford, K. Clarke, Y. de Fontaine, B. Jessop, and M. Feigenbaum. 2005. Conservation status and population trends of the American eel in Canada. Canadian Science Advisory Secretariat, Fisheries and Oceans Canada. Draft October 9, 2005. Section 3. Biology and Distribution

Castonguay M, Hodson PV, Moriarty C, Drinkwater KF, Jessop BM. Is there a role of ocean environment in American and European eel decline? Fish Oceanogr. 1994; 3(3): 197-203. doi: 10.1111/j.1365-2419.1994.tb00097.x

Council for Endangered Species Act Reliability (CESAR). 2010. Petition to list the American Eel (Anguilla rostrata) as a threatened species under the Endangered Species Act. Accessed online (May 2014): http://www.fws.gov/northeast/newsroom/pdf/American_eel_petition_100430.pdf

Friedland KD, Miller MJ, Knights B. Oceanic changes in the Sargasso Sea and declines in recruitment of the European eel. ICES J Mar Sci. 2007; 64(3): 519-530. doi: 10.1093/icesjms/fsm022

McCleave JD. Fish: Eels. In J. Steele J, McNeil C, editors. Encyclopedia of Ocean Sciences. London: Academic Press; 2001. pp. 800-809.

Miller MJ, Casselman JM. The American eel: a fish of mystery and sustenance for humans. In Eels and Humans. Japan: Springer; 2014. pp. 155-169.

Sullivan MC, Able KW, Hare JA, Walsh HJ. Anguilla rostrata glass eel ingress into two, US east coast estuaries: patterns, processes and implications for adult abundance. J Fish Biol. 2006; 69(4): 1081-1101. doi: 10.1111/j.1095-8649.2006.01182.x

U.S. Fish and Wildlife Service (FWS). 2011. Endangered and Threatened Wildlife and Plants; 90-Day finding on a petition to list the American Eel as threatened. 50CFR Part 17. Federal Register 76: 60431-60444. Available: https://www.federalregister.gov/articles/2011/09/29/2011-25084/endangered-and-threatened-wildlife-and-plants-90-day-finding-on-a-petition-to-list-the-american-eel

[Return to Table of Contents](#)

American Lobster – *Homarus americanus*

Overall Vulnerability Rank = Moderate

Biological Sensitivity = Moderate
Climate Exposure = High
Data Quality = 88% of scores ≥ 2

Homarus americanus	Expert Scores	Data Quality	Expert Scores Plots (Portion by Category)
Stock Status	2.3	1.7	
Other Stressors	2.6	2.4	
Population Growth Rate	3.3	2.7	
Spawning Cycle	2.7	3.0	
Complexity in Reproduction	1.4	2.4	
Early Life History Requirements	2.4	2.6	
Sensitivity to Ocean Acidification	2.1	2.6	
Prey Specialization	1.1	3.0	
Habitat Specialization	1.6	3.0	
Sensitivity to Temperature	2.3	2.6	
Adult Mobility	2.1	2.4	
Dispersal & Early Life History	1.9	2.8	
Sensitivity Score	Moderate		
Sea Surface Temperature	4.0	3.0	
Variability in Sea Surface Temperature	1.0	3.0	
Salinity	1.4	3.0	
Variability Salinity	1.2	3.0	
Air Temperature	2.4	3.0	
Variability Air Temperature	1.0	3.0	
Precipitation	1.2	3.0	
Variability in Precipitation	1.2	3.0	
Ocean Acidification	4.0	2.0	
Variability in Ocean Acidification	1.0	2.2	
Currents	2.1	1.0	
Sea Level Rise	1.3	1.5	
Exposure Score	High		
Overall Vulnerability Rank	Moderate		

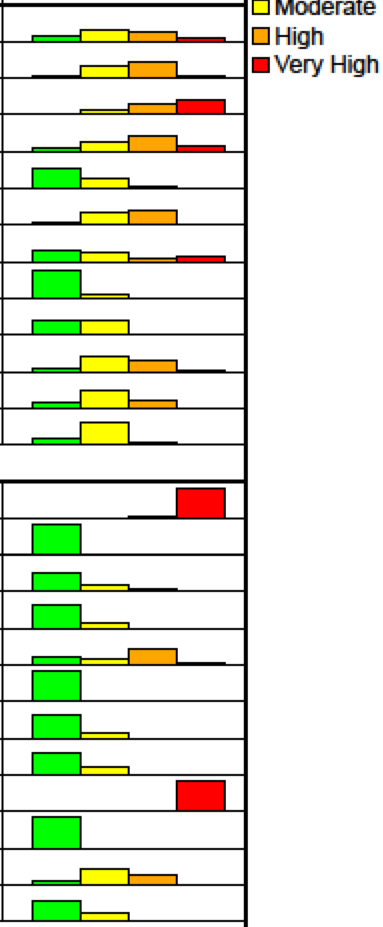

American Lobster

Overall Climate Vulnerability Rank: **Moderate** (93% certainty from bootstrap analysis).

Climate Exposure: **High**. Two exposure factors contributed to this score: Ocean Surface Temperature (3.9) and Ocean Acidification (4.0). All life stages of American Lobster use marine habitats.

Biological Sensitivity: **Moderate**. Three sensitivity attributes scored above 2.5: Population Growth Rate (3.3), Spawning Cycle (2.7), and Other Stressors (2.6). American Lobster are relatively slow-growing and can reach ages >50 years. Fertilization occurs after molting in the summer. Eggs are carried for almost a year before release as planktonic larvae. Other stressors are effecting lobster including a shell disease and decreased water quality in once highly productive bays and sounds in the region (e.g., Long Island Sound).

Distributional Vulnerability Rank: **High** (97% certainty from bootstrap analysis).

Directional Effect in the Northeast U.S. Shelf: The effect of climate change on American Lobster on the Northeast U.S. Shelf is estimated to be neutral, but with a moderate degree of uncertainty (66-90% certainty in expert scores). Research suggests that crustaceans are not negatively impacted by ocean acidification. American Lobster has changed distribution and abundances in the southern part of the region have decreased, but abundances in the northern part have increased dramatically. Thus, across the whole region the effects of climate change are estimated to be neutral.

Data Quality: 88% of the data quality scores were 2 or greater indicate that data quality is moderate.

Climate Effects on Abundance and Distribution: Recent warming has been linked to population decreases in the southern portion of the Northeast U.S. Shelf (Wahle et al., 2015) and population increases in the northern portion (Mills et al., 2012). Similar regional patterns were observed during a system-wide warming event in the 1950s (Taylor et al., 1957). Experimental work indicates negative physiological effects at summer temperatures now common in the southern part of the range (Dove et al., 2005). Juvenile shell growth increased under lower aragonite saturation state suggesting positive effects of ocean acidification (Ries et al., 2009). However, larval growth decreased and development times increased under lower pH conditions (Keppel et al., 2012).

Life History Synopsis**:** American Lobster is a benthic marine species ranging from Maine to New Jersey in inshore waters, and from Maine to North Carolina in offshore waters (ASMFC, 2014). Females may take 10 years to mature (Acheson and Steneck, 1997). Spawning occurs after the female molts from Late May through September (Factor, 1995). Sperm can be held internally by the female for up to two years before being extruded to fertilize eggs (ASMFC, 2014). Eggs are attached to the underside of the female and carried for 9-11 months, hatching in mid-May to mid-June (ASMFC, 2014). Associated with the surface layer, larvae are dispersed by a combination of surface drift, vertical migrations, and directed swimming, allowing lobster spawned offshore to settle in inshore habitats (Harding et al., 1982; Katz et al., 1994; Factor, 1995). Larvae are pelagic for up to two months (Factor, 1995). The timing and location of settlement are determined by temperature and the presence or absence of the thermocline (Templeman, 1936; Boudreau et al., 1992; Factor, 1995). Larvae and postlarvae eat phytoplankton and zooplankton (copepods and other invertebrate larvae) and are preyed upon by Cunner and other fish (Factor, 1995). As juveniles, American Lobster are restricted to sheltered benthic habitat such as rocks, cobble, and vegetation, sometimes burrowing in mud, and are more active at night (Wahle and Steneck,

1991). Adult American Lobsters also use shelter such as boulders and kelp, but are not as restricted (Wahle and Steneck, 1991; Acheson and Steneck, 1997). Juveniles and adults undergo an ontogenetic shift in diet from softer, more easily acquired prey, such as scavenged flesh, Blue Mussels, and macroalgae to increased reliance on hard-shelled taxa such as Rock Crab in addition to molluscs, echinoderms, and polychaetes as they grow (Elner and Campbell, 1987; Sainte-Marie and Chabot, 2002). American Lobster is managed as three stock units based on life history differences: Gulf of Maine, Georges Bank, and southern New England. All three stocks are managed by the Atlantic States Marine Fisheries Commission to increase female egg production particularly for the depleted southern New England stock (ASMFC, 2014).

Literature Cited:

Acheson JM, Steneck RS. Bust and then boom in the Maine lobster industry: perspectives of fishers and biologists. N Amer J of Fisher Man. 1997; 17(4): 826-847. DOI:10.1577/1548-8675(1997)017<0826:BATBIT>2.3.CO;2

Atlantic States Marine Fisheries Commission (ASMFC). American Lobster. 2014. Accessed online (August 2014): http://www.asmfc.org/species/american-lobster

Boudreau B, Simard Y, Bourget E. Influence of thermocline on vertical distribution and settlement of post-larvae of the American lobster *Homarus americanus* Milne-Edwards. J Exp Mar Biol Ecol. 1992; 162: 35-49. DOI: 10.1016/0022-0981(92)90123-R

Dove AD, Allam B, Powers JJ, Sokolowski MS. A prolonged thermal stress experiment on the American lobster, *Homarus americanus*. J Shellf Res. 2005; 24(3): 761-765. Accessed Online (August 2015): http://www.somas.stonybrook.edu/~madl/pubspdf/Dove-thermalstress.pdf

Elner RW, Campbell A. Natural diets of lobster Homarus americanus from barren ground and macroalgal habitats off southwestern Nova Scotia, Canada. Mar Ecol Prog Ser. 1987; 37: 131–140. Accessed Online (August2015): http://www.int-res.com/articles/meps/37/m037p131.pdf

Factor JR. Biology of Lobster: Homarus americanus. Academic Press, Inc. 1995; 516 pp.

Harding GC, Vass WP, Drinkwater KP. Aspects of larval American lobster (Homarus americanus) ecology in St. Georges Bay, Nova Scotia. Can J Fish Aquat Sci. 1982; 39: 1117-1129. DOI: 10.1139/f82-149

Katz CH, Cobb JS, Spaulding M. Larval behavior, hydrodynamic transport, and potential offshore-to-inshore recruitment in the American lobster *Homarus americanus*. Mar. Ecol. Prog. Ser. 1994; 103: 265-273. DOI: 10.3354/meps103265

Keppel EA, Scrosati RA, Courtenay SC. Ocean acidification decreases growth and development in American lobster (*Homarus americanus*) larvae. J Northw Atl Fish Sci. 2012; 44: 61-66. DOI: 10.2960/J.v44.m683

Mills KE, Pershing AJ, Brown CJ, Chen Y, Chiang FS, Holland DS, et al. Fisheries management in a changing climate lessons from the 2012 ocean heat wave in the Northwest Atlantic. Oceanography, 2013; 26(2): 191-195. DOI: http://dx.doi.org/10.5670/oceanog.2013.27

Ries JB, Cohen AL, McCorkle DC. Marine calcifiers exhibit mixed responses to CO2-induced ocean acidification. Geol. 2009; 37(12), 1131-1134. doi: 10.1130/G30210A.1

Sainte-Marie B, Chabot D. Ontogenetic shifts in natural diet during benthic stages of American lobsters (*Homarus americanus*), off the Magdalen Islands. Fish Bull. 2002; 100: 106-116. Accessed Online (August 2015); http://fishbull.noaa.gov/1001/sai.pdf

Taylor CC, Bigelow HB, Graham HW. Climatic trends and the distribution of marine animals in New England. Fish Bull. 1957; 293-345. Accessed Online (August 2015): http://fishbull.noaa.gov/57-1/taylor.pdf

Templeman W. The influence of temperature, salinity, light and food conditions on the survival and growth of the larvae of the lobster (*Homarus americanus*). J Biol Board Can. 1936; 2: 485-497. DOI: 10.1139/f36-029

Wahle RA, Steneck RS. Recruitment habitats and nursery grounds of the American lobster *Homarus americanus*: a demographic bottleneck? Mar Ecol Prog Ser. 1991; 69: 231-243. Accessed Online (August 2015): http://www.int-res.com/articles/meps/69/m069p231.pdf

Wahle RA, Dellinger L, Olszewski S, Jekielek P. American lobster nurseries of southern New England receding in the face of climate change. ICES J Mar Sci. 2015; fsv093. DOI: 10.1093/icesjms/fsv093

Return to Table of Contents

American Plaice – *Hippoglossoides platessoides*

Overall Vulnerability Rank = Low

Biological Sensitivity = Low
Climate Exposure = High
Data Quality = 88% of scores ≥ 2

Hippoglossoides platessoides	Expert Scores	Data Quality	Expert Scores Plots (Portion by Category)
Stock Status	3.0	3.0	
Other Stressors	1.2	1.6	
Population Growth Rate	2.5	2.6	
Spawning Cycle	2.1	2.9	
Complexity in Reproduction	1.6	2.7	
Early Life History Requirements	2.2	2.5	
Sensitivity to Ocean Acidification	2.1	2.5	
Prey Specialization	1.9	2.8	
Habitat Specialization	1.1	2.4	
Sensitivity to Temperature	1.8	2.8	
Adult Mobility	2.4	2.6	
Dispersal & Early Life History	1.4	2.7	
Sensitivity Score	Low		
Sea Surface Temperature	3.9	3.0	
Variability in Sea Surface Temperature	1.0	3.0	
Salinity	1.1	3.0	
Variability Salinity	1.2	3.0	
Air Temperature	1.0	3.0	
Variability Air Temperature	1.0	3.0	
Precipitation	1.0	3.0	
Variability in Precipitation	1.0	3.0	
Ocean Acidification	4.0	2.0	
Variability in Ocean Acidification	1.0	2.2	
Currents	2.1	1.0	
Sea Level Rise	1.1	1.5	
Exposure Score	High		
Overall Vulnerability Rank	Low		

Low
Moderate
High
Very High

Return to Table of Contents

American Plaice (*Hippoglossoides platessoides*)

Overall Climate Vulnerability Rank: **Low** (36% certainty from bootstrap analysis).

Climate Exposure: **High**. Two exposure factors contributed to this score: Ocean Surface Temperature (3.9) and Ocean Acidification (4.0). All life stages of American Plaice use marine habitats.

Biological Sensitivity: **Low**. One sensitivity attributes scored above 2.5: Stock Status (3.0). The Stock is overfished, but not currently undergoing overfishing (O'Brien, 2006).

Distributional Vulnerability Rank: **High** (100% certainty from bootstrap analysis).

Directional Effect in the Northeast U.S. Shelf: The effect of climate change on American Plaice on the Northeast U.S. Shelf is very likely to be negative (>95% certainty in expert scores). Warming may decrease productivity and may cause reductions in available habitat.

Data Quality: 88% of the data quality scores were 2 or greater indicate that data quality is moderate.

Climate Effects on Abundance and Distribution: There is relatively little information regarding the effect of climate factors on American Plaice. Brodziak and O'Brien (2005) found a link between recruitment and NAO but the relationship was curvilinear. In terms of distribution, American Plaice have not shifted latitudinally, but have increased in depth (Nye et al., 2009).

Life History Synopsis**:** American Plaice is a cold-water (-1.5-13 ^{o}C), benthic, marine species occurring on both sides of the North Atlantic Ocean, ranging from Labrador to New York in the western Atlantic (Johnson et al., 1999; Johnson, 2004). Temperature can have a significant effect on the growth and maturity of the species, but plaice are generally mature by 4 years (Johnson et al., 1999; Johnson, 2004; O'Brien, 2006). Mature fish spawn on the sea floor in 3-6°C water at depths <90 m, and make short migrations from offshore if necessary (Johnson et al., 1999; Klein-MacPhee, 2002; Johnson, 2004). Plaice are batch spawners over a roughly 26-day period between January and August, peaking in spring (Johnson et al., 1999; Klein-MacPhee, 2002; Johnson, 2004). The resultant eggs are pelagic and generally retained within the 100m isobath (Klein-MacPhee, 2002; Johnson, 2004). Incubation lasts approximately 2 weeks (Johnson et al., 1999; Klein-MacPhee, 2002; Johnson, 2004). Larvae drift near the surface for 3-4 months (varying by temperature) eating diatoms and copepods (Johnson et al., 1999; Klein-MacPhee, 2002; Johnson, 2004). Metamorphosis is complete by 30 - 40 mm, and is temperature dependent (Johnson et al., 1999; Johnson, 2004). Juveniles settle to the bottom and eat small shrimps, crustaceans, polychaetes, and by 41-45 cm concentrate more on echinoderms (Johnson et al., 1999; Klein-MacPhee, 2002; Johnson, 2004). Juvenile and adult American Plaice are mostly sedentary on soft bottom substrate, but make short migrations for food and spawning (Johnson et al., 1999; Klein-MacPhee, 2002; Johnson, 2004). Adult American Plaice are opportunistic predators on small bottom-dwelling organisms such as echinoderms (brittle stars), bivalves, polychaetes, and small crustaceans (Johnson et al., 1999; Klein-MacPhee, 2002; Johnson, 2004). Atlantic Cod are the dominant predator of American Plaice <35 cm (Johnson et al., 1999; Johnson, 2004). The primary predators of adult American Plaice include larger predatory fish and mammals such as Greenland Shark, Monkfish (Goosefish), Spiny Dogfish, and grey seals (Johnson et al., 1999; Klein-MacPhee, 2002; Johnson, 2004). American Plaice are managed under the Greater Atlantic Regional Fisheries Office, Northeast Multispecies Fishery Management Plan to rebuild stocks to target biomass levels (Johnson et al., 1999; O'Brien, 2006). The Stock is overfished, but not currently undergoing overfishing (NEFSC 2012).

Literature Cited:

Brodziak J, O'Brien L. Do environmental factors affect recruits per spawner anomalies of New England groundfish?. ICES J Mar Sci. 2005; 62(7): 1394-1407. DOI:10.1016/j.icesjms.2005.04.019

Johnson DL. American plaice, *Hippoglossoides platessoides*, life history and habitat characteristics. NOAA Tech. Memo. 2004; NMFS-NE-187: 72 p. Accessed Online (August 2015): http://www.nefsc.noaa.gov/nefsc/publications/tm/tm187/tm187.pdf

Johnson DL; Berrien PL, Morse WW, Vitaliano JJ. American plaice, *Hippoglossoides platessoides*, life history and habitat characteristics. NOAA Tech. Memo. 1999; NMFS-NE-123: 31 p. Accessed Online (August 2015): http://www.nefsc.noaa.gov/nefsc/publications/tm/tm123/tm123.pdf

Klein-MacPhee G. American plaice/*Hippoglossoides platessoides* (Fabricius 1780). In: Collette BB, Klein-MacPhee G, editors. Bigelow and Schroeder's fishes of the Gulf of Maine. 3rd Edition. Washington, DC: Smithsonian Institution Press. 2012; 564-569.

Northeast Fisheries Science Center. Assessment or Data Updates of 13 Northeast Groundfish Stocks through 2010. US Dept Commer, Northeast Fish Sci Cent Ref Doc. 2012; 12-06: 789 p. Accessed Online (August 2015): http://nefsc.noaa.gov/publications/crd/crd1206/

Nye JA, Link JS, Hare JA, Overholtz WJ. Changing spatial distribution of fish stocks in relation to climate and population size on the Northeast United States continental shelf. Mar Ecol Prog Ser. 2009; 393: 111-129. DOI: 0.3354/meps08220

Return to Table of Contents

American Shad – *Alosa sapidissima*

Overall Vulnerability Rank = Very High

Biological Sensitivity = High
Climate Exposure = Very High
Data Quality = 88% of scores ≥ 2

Alosa sapidissima	Expert Scores	Data Quality	Expert Scores Plots (Portion by Category)
Stock Status	2.9	1.4	
Other Stressors	3.7	2.0	
Population Growth Rate	2.3	2.0	
Spawning Cycle	3.7	2.8	
Complexity in Reproduction	3.3	2.8	
Early Life History Requirements	3.3	2.8	
Sensitivity to Ocean Acidification	1.4	2.0	
Prey Specialization	1.6	2.5	
Habitat Specialization	2.9	2.6	
Sensitivity to Temperature	1.6	3.0	
Adult Mobility	1.4	2.8	
Dispersal & Early Life History	3.2	3.0	
Sensitivity Score	High		
Sea Surface Temperature	4.0	3.0	
Variability in Sea Surface Temperature	1.0	3.0	
Salinity	1.6	3.0	
Variability Salinity	1.2	3.0	
Air Temperature	4.0	3.0	
Variability Air Temperature	1.0	3.0	
Precipitation	1.3	3.0	
Variability in Precipitation	1.4	3.0	
Ocean Acidification	4.0	2.0	
Variability in Ocean Acidification	1.0	2.2	
Currents	2.0	1.0	
Sea Level Rise	2.8	1.5	
Exposure Score	Very High		
Overall Vulnerability Rank	Very High		

Low
Moderate
High
Very High

Return to Table of Contents

American Shad (*Alosa sapidissima*)

Overall Climate Vulnerability Rank: **Very High** (100% certainty from bootstrap analysis).

Climate Exposure: **Very High**. Three exposure factors contributed to this score: Ocean Surface Temperature (4.0), Ocean Acidification (4.0) and Air Temperature (4.0). American Shad are anadromous, spawning in freshwater, developing in freshwater and estuarine habitats, feeding as adults in marine habitats.

Biological Sensitivity: **High**. Five sensitivity attributes scored above 3.0: Other Stressors (3.7), Spawning Cycle (3.7), Early Life History Requirements (3.3), Complexity in Reproduction (3.3), and Dispersal and Early Life History (3.2). American Shad are diadromous and exposed to a number of other stressors including habitat destruction, blockage to spawning habitats, and contaminants (Limburg and Waldman, 2009). Spawning time varies latitudinally and is linked to spring warming (Munroe, 2002). Eggs and larvae inhabit freshwaters and then juveniles move to estuarine and ocean waters. Dispersal of eggs and larvae is limited.

Distributional Vulnerability Rank: **Low** (97% certainty from bootstrap analysis). American Shad have a relatively high degree of spawning fidelity, limiting the ability of the species to shift distribution. Fidelity to the river system is high; fidelity to specific tributaries is lower (Walther et al., 2008).

Directional Effect in the Northeast U.S. Shelf: The effect of climate change on American Shad is estimated to be negative, but this estimate has a high degree of uncertainty (<66% certainty in expert scores). American Shad is distributed to Florida, so northward range shifts will likely move American Shad into the Northeast U.S. Shelf. However, changes in rivers from increased precipitation and warming will likely cause decreases in productivity particularly in the southern portion of the Northeast U.S. shelf. The effect of ocean acidification over the next 30 years is likely to be minimal.

Data Quality: 88% of the data quality scores were 2 or greater indicate that data quality is moderate.

Climate Effects on Abundance and Distribution: A number of studies indicate climate effects on American Shad productivity. Crecco et al. (1986) found that spring river flows affected subsequent recruitment. Castro-Santos and Letcher (2010) developed a simulation model of up-stream migration and spawning and concluded that behavior, physiology, and energetics strongly affected both the distribution of spawning effort and survival to the marine environment. Leach and Houde (1998) found that larval growth and survival is related to temperature. Leggett and Carscadden (1978) concluded that the thermal regime of the home river was the principal factor influencing reproductive strategy in American Shad. These studies indicate that climate effects (e.g., streamflow, temperature) will impact the ecology and productivity of American Shad along the East Coast. Distribution in the marine stage has also changed substantially over the past several decades and a link to climate has been suggested (Nye et al., 2009). The impact of the interaction between distribution changes in the marine phase and strong natal homing is not clear.

Life History Synopsis**:** American Shad is an anadromous, migratory, pelagic, schooling species ranging from southern Labrador to northern Florida with some introductions along the United States Pacific coast (Munroe, 2002; Able and Fahay, 2010). The center of abundance is in the highly urbanized and disturbed section of North America from Connecticut to North Carolina (Able and Fahay, 2010). American Shad take 3-6 years to reach sexual maturity, most of which is spent in the ocean (Able and

Fahay, 2010; ASMFC, 2007). American Shad are semelparous south of Cape Hatteras, but increasing rates of iteroparity occur at increasing latitude (Munroe, 2002). American Shad return to natal rivers in dense schools to batch spawn, a process determined by river temperature and usually occurring in spring (Munroe, 2002; Able and Fahay, 2010). The beginning and cessation of spawning is cued by temperature (12-20 ^{o}C; Able and Fahay, 2010) and usually occurs well upstream when not impeded by dams (Munroe, 2002). Eggs sink within several meters of the spawning site and often get lodged in bottom substrate (Munroe, 2002). Larvae are planktonic for approximately one month before metamorphosing and feed opportunistically on zooplankton such as copepods, euphausiids, and insect larvae (Munroe, 2002; Able and Fahay, 2010). Juveniles mature in the estuary, feeding on zooplankton (copepods, cladocerans, and insect larvae; Munroe, 2002; Able and Fahay, 2010). Migration to the marine environment is size (75-125 mm) and temperature dependent, with young American Shad emerging from the estuary from late fall to spring (Munroe, 2002; Able and Fahay, 2010). Overwintering usually occurs near the mouth of the parent river (Munroe, 2002). Adult American Shad occur across the continental shelf with seasonal patterns in along-shelf, cross-shelf, and depth distributions (north and deep in fall, south and deep in winter, widely distributed in spring and summer; Munroe, 2002; Able and Fahay, 2010). Adults are opportunistic feeders on zooplankton (copepods, mysids), but consume much less during the spawning run (Munroe, 2002; Able and Fahay, 2010). Many predators consume American Shad, such as Bluefish and Sea Lamprey in rivers and Spiny Dogfish and seals at sea (Munroe, 2002; Able and Fahay, 2010). American Shad are considered fully exploited due to low population sizes caused by fishing and habitat destruction (ASMFC, 2007). Major efforts to improve access to and to restore spawning grounds are helping (Munroe, 2002), but the stocks in general remain depleted (ASMFC, 2014). American Shad are managed by the Atlantic States Marine Fisheries Commission to reduce overfishing and habitat destruction (ASMFC, 2014).

Literature Cited:

Able KW, Fahay MP. Ecology of estuarine fishes: temperate waters of the western North Atlantic. Baltimore: The Johns Hopkins University Press; 2010. 566p.

Atlantic States Marine Fisheries Commission (ASMFC). 2007. American shad stock assessment report for peer review Vol. 1. Stock Assessment Report No. 07-01 (Supplement). 224p. Accessed online (May 2014): http://www.asmfc.org/uploads/file/2007ShadStockAssmtReportVolumeI.pdf

Atlantic States Marine Fisheries Commission (ASMFC). 2014. Shad and River Herring. Accessed online (May 2014): http://www.asmfc.org/species/shad-river-herring

Castro-Santos T, Letcher BH. Modeling migratory energetics of Connecticut River American shad (Alosa sapidissima): implications for the conservation of an iteroparous anadromous fish. Can J Fish Aquat Sci. 2010; 67(5): 806-830. doi: 10.1139/F10-026

Crecco V, Savoy T, Whitworth W. Effects of density-dependent and climatic factors on American shad, Alosa sapidissima, recruitment: a predictive approach. Can J Fish Aquat Sci. 1986; 43(2): 457-463. doi: 10.1139/f86-056

Leach SD, Houde ED. Effects of environmental factors on survival, growth, and production of American shad larvae. J Fish Biol. 1999; 54(4): 767-786. doi: 10.1111/j.1095-8649.1999.tb02032.x

Leggett WC, Carscadden JE. Latitudinal variation in reproductive characteristics of American shad (Alosa sapidissima): evidence for population specific life history strategies in fish. J Fish Board Can. 1978; 35(11): 1469-1478. doi: 10.1139/f78-230

Limburg KE, Waldman JR. Dramatic declines in North Atlantic diadromous fishes. BioScience, 2009; 59(11): 955-965. doi: 10.1525/bio.2009.59.11.7

Munroe TA. American shad/ *Alosa sapidissima* (Wilson 1811). In: B.B. Collette BB, Klein-MacPhee G, editors, Fishes of the Gulf of Maine, 3rd ed. Washington: Smithsonian Institution Press; 2002. pp. 125-132.

Nye, JA, Lynch P, Alexander M, Stock C, Hare J. 2012. Results of Preliminary Analysis of the Effect of Climate Change on River Herring. Fisheries and the Environment (FATE) Annual Report. 17pp. Available: http://www.nefsc.noaa.gov/epd/ocean/MainPage/Climate_and_river_herring_summary.pdf

Return to Table of Contents

Anchovies – *Anchoa spp.*

Overall Vulnerability Rank = Low

Biological Sensitivity = Low
Climate Exposure = High
Data Quality = 88% of scores ≥ 2

Anchoa spp.	Expert Scores	Data Quality	Expert Scores Plots (Portion by Category)
Stock Status	1.7	1.4	
Other Stressors	2.1	2.2	
Population Growth Rate	1.2	2.8	
Spawning Cycle	1.6	3.0	
Complexity in Reproduction	1.3	2.2	
Early Life History Requirements	2.5	2.2	
Sensitivity to Ocean Acidification	1.4	2.4	
Prey Specialization	1.2	2.4	
Habitat Specialization	1.5	3.0	
Sensitivity to Temperature	1.4	2.6	
Adult Mobility	2.2	2.4	
Dispersal & Early Life History	1.9	2.6	
Sensitivity Score	Low		
Sea Surface Temperature	4.0	3.0	
Variability in Sea Surface Temperature	1.0	3.0	
Salinity	2.6	3.0	
Variability Salinity	1.2	3.0	
Air Temperature	3.4	3.0	
Variability Air Temperature	1.0	3.0	
Precipitation	1.1	3.0	
Variability in Precipitation	1.1	3.0	
Ocean Acidification	4.0	2.0	
Variability in Ocean Acidification	1.0	2.2	
Currents	2.2	1.0	
Sea Level Rise	2.3	1.5	
Exposure Score	High		
Overall Vulnerability Rank	Low		

Low
Moderate
High
Very High

Anchovies (*Anchoa hepsetus / Anchoa mitchilli*)

Overall Climate Vulnerability Rank: **Low** (80% certainty from bootstrap analysis).

Climate Exposure: **High**. Two exposure factors contributed to this score: Ocean Surface Temperature (4.0) and Ocean Acidification (4.0). Exposure to ocean surface temperature and ocean acidification occurs during all life stages.

Biological Sensitivity: **Low**. Only one sensitivity attribute was scored at a 2.5 or higher: Early Life History Requirements (2.5). Anchovies spawn in estuarine and nearshore habitats.

Distributional Vulnerability Rank: **High** (89% certainty from bootstrap analysis). Two attributes contributed to the high vulnerability for a distribution shift. Anchovies are habitat generalists and occur in estuarine and coastal waters throughout the southern portion of the Northeast U.S. Shelf. Anchovies also have early life stages that disperse in the coastal zone; whether there is exchange among estuarine systems is unknown.

Directional Effect in the Northeast U.S. Shelf: The effect of climate change on anchovies on the Northeast U.S. Shelf is very likely to be positive (>95% certainty in expert scores). As warming continues more habitat in the Northeast U.S. is expected to become available. Based on research in other regions, population productivity is also likely to increase with continued warming. The effect of ocean acidification over the next 30 years is likely to be minimal.

Data Quality: 88% of the data quality scores were 2 or greater.

Climate Effects on Abundance and Distribution: There have been surprisingly few studies of the effect of climate change on *Anchoa* spp., especially in the Northeast U.S. Shelf ecosystem. Lou and Brandt (1993) developed a bioenergetics model for *Anchoa mitchilli* in the Chesapeake Bay; their work indicated that Bay Anchovy consumption of zooplankton will increase with warming waters. In the Black Sea using an ecosystem bioenergetics model, Güraslan et al. (2014) indicated that population productivity of anchovies would increase as temperature rises.

Life History Synopsis**:** Anchovies are small, coastal, pelagic, schooling species found along most of the western North Atlantic coast including the Gulf of Mexico (Able and Fahay, 2010). These highly fecund species mature early (after their first winter or approximately 10 months), have a long spawning season (spring to early fall in temperate waters, possibly year-round in subtropical zones), and batch spawn often (every 1-4 days for a total of approximately 55 batches in a season; Munroe, 2002). The energy for this large spawning effort comes from daily consumption, not stored energy, and peak spawning corresponds to peak microzooplankton abundance (Munroe, 2002). Eggs are pelagic and hatch in approximately 24 hours (Hildebrand, 1963). Larvae absorb yolk within 2 days (Hildebrand, 1963; Munroe, 2002). Eggs and larvae are planktonic, both in estuaries and on the inner shelf, and are usually found in surface waters, but may go deeper at night (Munroe, 2002; Able and Fahay, 2010). Anchovy larvae are very common and are often the most abundant ichthyoplankton in collections (Munroe, 2002). Juvenile anchovies occur in estuaries during summer, but some species or regional populations may make winter migrations to deeper water within the estuary or to the inner-shelf (Munroe, 2002). Able to tolerate a wide range of salinities, adult anchovy occur from as far offshore as the Gulf Stream all the way into freshwater, but are most common on the coastal inner-shelf and in estuaries (Munroe, 2002; Able and Fahay, 2010). Cross-shelf and along-shelf seasonal migrations to avoid sharp drops in

temperature may occur (Munroe, 2002; Able and Fahay, 2010). Juvenile and adult anchovies are planktivorous, consuming zooplankton such as copepods, mysids, and occasionally (when >100 mm) fish larvae and other small fish (Munroe, 2002; Able and Fahay, 2010). Weakfish, Bluefish, Atlantic Mackerel, and many other predatory fish, sharks, and birds prey on anchovies (Hildebrand, 1963; Munroe, 2002; Able and Fahay, 2010). Anchovy are not managed in the western North Atlantic and are generally one of the most abundant species in bays, estuaries, and coastal systems (Munroe, 2002). However, natural mortality is very high (approximately 95%) for this well-utilized prey species that links secondary production directly to fisheries (Munroe, 2002).

Literature Cited:

Able KW, Fahay MP. Ecology of estuarine fishes: temperate waters of the western North Atlantic. Baltimore: The Johns Hopkins University Press; 2010. 566p.

Güraslan C, Fach BA, Oguz T. Modeling the impact of climate variability on Black Sea anchovy recruitment and production. Fish Oceanogr. 2014; 23(5): 436-457. doi: 10.1111/fog.12080

Hildebrand SF. Fishes of the Western North Atlantic, Part 3, Number I. New Haven: Sears Foundation for Marine Research; 1963. 630p.

Lou J, Brandt SB. Bay anchovy production and consumption in mid-Chesapeake Bay based upon a bioenergetics model and acoustic measurements of fish abundance. Mar Ecol Prog Ser. 1993; 98: 223-236.

Munroe TA. Anchovies. Family Engraulidae. In: Collete BB, Klein-MacPhee G, editors, Fishes of the Gulf of Maine, 3rd edition. Washington: Smithsonian Institution Press; 2002. pp. 105-111.

Atlantic Cod – *Gadus morhua*

Overall Vulnerability Rank = Moderate

Biological Sensitivity = Moderate

Climate Exposure = High

Data Quality = 92% of scores ≥ 2

Gadus morhua	Expert Scores	Data Quality	Expert Scores Plots (Portion by Category)
Stock Status	3.9	3.0	
Other Stressors	2.0	2.5	
Population Growth Rate	2.8	2.8	
Spawning Cycle	2.0	3.0	
Complexity in Reproduction	2.4	2.9	
Early Life History Requirements	2.8	2.8	
Sensitivity to Ocean Acidification	1.3	2.0	
Prey Specialization	1.2	3.0	
Habitat Specialization	1.8	3.0	
Sensitivity to Temperature	2.0	3.0	
Adult Mobility	2.2	2.8	
Dispersal & Early Life History	1.4	2.8	
Sensitivity Score	Moderate		
Sea Surface Temperature	3.9	3.0	
Variability in Sea Surface Temperature	1.0	3.0	
Salinity	1.4	3.0	
Variability Salinity	1.2	3.0	
Air Temperature	1.0	3.0	
Variability Air Temperature	1.0	3.0	
Precipitation	1.0	3.0	
Variability in Precipitation	1.0	3.0	
Ocean Acidification	4.0	2.0	
Variability in Ocean Acidification	1.0	2.2	
Currents	2.1	1.0	
Sea Level Rise	1.6	1.5	
Exposure Score	High		
Overall Vulnerability Rank	Moderate		

Low
Moderate
High
Very High

Return to Table of Contents

Atlantic Cod (*Gadus morhua*)

Overall Climate Vulnerability Rank: **Moderate.** (90% certainty from bootstrap analysis).

Climate Exposure: **High**. Two exposure factors contributed to this score: Ocean Surface Temperature (3.9) and Ocean Acidification (4.0). All life stages of Atlantic Cod use marine habitats.

Biological Sensitivity: **Moderate**. Three sensitivity attributes scored above 2.5: Stock Status (3.9), Population Growth Rate (2.8), and Early Life History Requirements (2.8). Atlantic Cod is overfished and overfishing is occurring (NEFSC, 2014). Atlantic Cod are relatively slow growing and long-lived (17 years, Lough, 2004; NEFSC, 2013). Finally, the early life stages may be dependent on circulation patterns on Georges Bank for larval retention and specific habitats on the Bank or in coastal regions for juvenile nursery habitats (Fahay, 1999; Lough, 2004).

Distributional Vulnerability Rank: **High** (98% certainty from bootstrap analysis). Atlantic Cod are habitat generalists that are mobile, and have dispersive early life stages (Lough, 2004).

Directional Effect in the Northeast U.S. Shelf: The effect of climate change on Atlantic Cod on the Northeast U.S. Shelf is very likely to be negative (>95% certainty in expert scores). Decreases in recruitment have been linked to increases in temperature and climate-related changes in prey in the Gulf of Maine. Further the species is at the southern end of its range and continued warming will likely result in further reductions in available habitat.

Data Quality: 88% of the data quality scores were 2 or greater indicate that data quality is moderate.

Climate Effects on Abundance and Distribution: Atlantic Cod productivity and distribution are both likely negatively affected by climate factors in the Northeast U.S. Shelf. Drinkwater (2005) noted that Atlantic Cod in the Northeast U.S. Shelf is at the southern end of the range and that warming waters will have negative impacts. Fogarty et al. (2007) developed a stock-recruitment relationship that included temperature; as temperatures increased, recruitment decreased.Pershing et al. (2015) conducted similar moldeing with updated data and the results were similar, providing further evidence of a negative effect of temperature on recruitment. Fogarty et al. (2007) also modeled Atlantic Cod distributions a function of temperature; as temperatures increased, Atlantic Cod distributions shifted northwards.

Life History Synopsis**:** Atlantic Cod are a typical marine groundfish that occurs on both coasts of the North Atlantic, but from Greenland to Cape Hatteras, North Carolina, in the western north Atlantic (Fahay et al., 1999; Lough, 2004). Over the last half century, age at maturity has fluctuated (1.7 - 5 years) reaching a low in the 1990s due to declines in stock abundance and increased temperature (Klein-MacPhee, 2002). Age at maturity has increased slightly (2-3 years) in the last decade (Lough, 2004). Spawning occurs at or near the bottom at night with a peak during winter and early spring (5-7 °C water) (Fahay et al., 1999; Lough, 2004). Peak spawning shifts with latitude and is correlated to temperature (Fahay et al., 1999; Lough, 2004). Fecundity is particularly high for Atlantic Cod, especially for larger females who produce millions of eggs over the course of a spawning season (Klein-MacPhee, 2002). Fertilized eggs are pelagic and hatch after 8-60 days varying with temperature, but average 2-3 weeks in typical spring conditions (Fahay et al., 1999; Lough, 2004). Larvae are pelagic, occurring from near surface down to 75 m, inhabiting deeper water with age (Fahay et al., 1999; Lough, 2004). Larvae consume phytoplankton and abundant zooplankton, such as copepods and nauplii (Fahay et al., 1999;

Klein-MacPhee, 2002; Lough, 2004). Planktivorous fish such as Atlantic Herring and Atlantic Mackerel are the main predators of larval Atlantic Cod (Lough, 2004). The pelagic stage lasts approximately 3 months after hatching (Fahay et al., 1999; Lough, 2004). Metamorphosis occurs at around 20 mm, but juveniles remain in the water column until they reach sizes of 40-60 mm, usually in early summer, then settle to the benthos (Fahay et al., 1999; Lough, 2004). Juvenile Atlantic Cod consume molluscs and crustaceans, selecting mostly pelagic prey when young, but increasingly benthic prey items as they grow and settle (Fahay et al., 1999; Lough, 2004). Gravel substrate provides better refuge from predators, which include many fishes, primarily: Spiny Dogfish, Sea Raven, Fourspot Flounder, and adult Atlantic Cod (Fahay et al., 1999; Klein-MacPhee, 2002; Lough, 2004). Adults occur from nearshore to offshore in cool water, with seasonal shifts (coastal northern waters in summer and fall; deeper southern waters in winter and spring) and age (near spawning grounds when young, more widely distributed and deeper, colder, and more saline with age (Fahay et al., 1999; Klein-MacPhee, 2002; Lough, 2004). These migrations are longer in the northern and southern extent of Atlantic Cod range and shorter in the middle part of the range (Fahay et al., 1999; Lough, 2004). Adults prefer rocky substrate usually occurring within 2 m of the bottom, but have been known to follow prey fish into the water column (Klein-MacPhee, 2002). Atlantic Cod travel as a mass with largest fish in the lead and smallest fish at the end (Fahay et al., 1999; Lough, 2004). Adult Atlantic Cod consume a variety of prey including pelagic and benthic invertebrates and smaller fish (Fahay et al., 1999; Lough, 2004). Large sharks and seals are among the only predators of large Atlantic Cod (Fahay et al., 1999; Klein-MacPhee, 2002; Lough, 2004). Cod in United States' waters have been exploited since at least the 1500s (Klein-MacPhee, 2002) and are currently managed as two stocks: Gulf of Maine and Georges Bank and southward (Fahay et al., 1999) under the New England Fishery Management Council's Northeast Multispecies Fishery Management Plan (Klein-MacPhee, 2002). Atlantic Cod are overfished and overfishing is occurring in both stocks (NEFSC 2013).

Literature Cited:

Drinkwater KF. The response of Atlantic cod (*Gadus morhua*) to future climate change. ICES J Mar Sci. 2005; 62(7): 1327-1337. DOI: 10.1016/j.icesjms.2005.05.015

Fahay MP, Berrien PL, Johnson DL, Morse WW. Essential fish habitat source document: Atlantic cod, *Gadus morhua*, Life history and habitat characteristics. NOAA Tech Memo. 1999; NMFS-NE-124: 41p. Accessed online (August 2015): http://www.nefsc.noaa.gov/nefsc/publications/tm/tm124/tm124.pdf

Fogarty M, Incze L, Hayhoe K, Mountain D, Manning J. Potential climate change impacts on Atlantic cod (Gadus morhua) off the northeastern USA. Mitigation and Adaptation Strategies for Global Change, 2008; 13(5-6): 453-466. DOI: 10.1007/s11027-007-9131-4

Klein-MacPhee G. Atlantic cod/*Gadus morhua* Linnaeus 1758. Pages 228–235 In: BB Collette, G Klein-MacPhee (editors), Fishes of the Gulf of Maine, 3rd edition. Smithsonian Institution Press, Washington D.C. 2012; 882 p.

Lough GR. Essential fish habitat source document: Atlantic cod, Gadus morhua, life history and habitat characteristics, Second Edition. NOAA Tech. Memo. 2004; NMFS-NE-190: 94p. Accessed Online (August 2014): http://www.nefsc.noaa.gov/nefsc/publications/tm/tm190/tm190.pdf

Northeast Fisheries Science Center (NEFSC). 55th Northeast Regional Stock Assessment Workshop (55th SAW) Assessment Summary Report. Northeast Fish Sci Cent Ref Doc. 2013; 13-01: 41 p. Accessed Online (August 2015): http://www.nefsc.noaa.gov/saw/saw55/crd1301.pdf

Northeast Fisheries Science Center (NEFSC). Gulf of Maine Atlantic Cod 2014 Assessment Update Report. Northeast Fisheries Science Center. 2014. Accessed Online (August 2014): http://www.nefsc.noaa.gov/saw/cod/pdfs/GoM_cod_2014_update_20140822.pdf

Pershing AJ, Alexander MA, Hernandez CM, Kerr LA, Le Bris A, Mills KE, et al. Slow adaptation in the face of rapid warming leads to collapse of the Gulf of Maine cod fishery. Science. 2015; 350(6262):809-12.

Return to Table of Contents

Atlantic Croaker – *Micropogonias undulatus*

Overall Vulnerability Rank = Moderate

Biological Sensitivity = Low
Climate Exposure = Very High
Data Quality = 88% of scores ≥ 2

Micropogonias undulatus	Expert Scores	Data Quality	Expert Scores Plots (Portion by Category)
Stock Status	1.9	1.6	
Other Stressors	2.2	2.4	
Population Growth Rate	1.7	2.4	
Spawning Cycle	1.8	3.0	
Complexity in Reproduction	2.1	2.6	
Early Life History Requirements	3.0	2.7	
Sensitivity to Ocean Acidification	1.6	2.0	
Prey Specialization	1.3	3.0	
Habitat Specialization	2.0	3.0	
Sensitivity to Temperature	1.4	3.0	
Adult Mobility	1.5	2.6	
Dispersal & Early Life History	1.8	2.6	
Sensitivity Score	Low		
Sea Surface Temperature	4.0	3.0	
Variability in Sea Surface Temperature	1.0	3.0	
Salinity	2.8	3.0	
Variability Salinity	1.2	3.0	
Air Temperature	4.0	3.0	
Variability Air Temperature	1.0	3.0	
Precipitation	1.2	3.0	
Variability in Precipitation	1.3	3.0	
Ocean Acidification	4.0	2.0	
Variability in Ocean Acidification	1.0	2.2	
Currents	2.0	1.0	
Sea Level Rise	2.5	1.5	
Exposure Score	Very High		
Overall Vulnerability Rank	Moderate		

Low
Moderate
High
Very High

Return to Table of Contents

Atlantic Croaker (*Micropogonias undulatus*)

Overall Climate Vulnerability Rank: **Moderate** (98% certainty from bootstrap analysis).

Climate Exposure: **Very High**. Three exposure factors contributed to this score: Ocean Surface Temperature (4.0), Ocean Acidification (4.0) and Air Temperature (4.0). Exposure to all three factors occur during all life stages. Atlantic Croaker spawn on the shelf, juveniles use estuarine nurseries, and adults make seasonal migrations to feed in estuarine and coastal areas.

Biological Sensitivity: **Low**. Only one sensitivity attribute scored above 2.5: Early Life History Requirements (3.0). Atlantic Croaker exhibit an obligate estuarine-dependent life cycle, using estuaries during the late larval and juvenile stages (Able 2005).

Distributional Vulnerability Rank: **High** (98% certainty from bootstrap analysis). Two attributes indicated vulnerability to distribution shift. Atlantic Croaker larvae are widely dispersed after spawning and adults make north-south seasonal migrations along the Northeast U.S. Shelf.

Directional Effect in the Northeast U.S. Shelf: The effect of climate change on Atlantic Croaker on the Northeast U.S. Shelf is very likely to be positive (>95% certainty in expert scores). Recruitment will likely increase as winters become warmer and the adult distribution will likely extend northwards. The effect of ocean acidification over the next 30 years is likely to be minimal.

Data Quality: 88% of the data quality scores were 2 or greater.

Climate Effects on Abundance and Distribution: Climate effects have been identified on both productivity and distribution of Atlantic Croaker. Hare and Able (2007) proposed that over-winter mortality of young-of-the-year in estuarine nursery habitats was a major contributor to recruitment variability. Hare et al. (2010) projected that as climate warmed, recruitment increased leading to higher population abundances. Hare et al. (2010) also projected northward expansion of the population, largely owing to increase in population abundance, but also directly related to warming temperatures. Diamond et al. (2013) indicated that warming temperatures would have a large positive effect on Atlantic Croaker in the region, but that increased variability in salinity, increased offshore transport, and sea-level rise would have negative effects. Munyandorero (2014) used a biomass dynamic model and found equivocal support for climate effects on Atlantic Croaker population dynamics and recommended continue monitoring and modeling of the population and the environment.

Life History Synopsis**:** Atlantic Croaker is an estuarine-dependent, coastal species occurring from Cape Cod, Massachusetts, through the Gulf of Mexico (Able and Fahay, 2010). There is no genetic differentiation among populations along the Atlantic coast, but the Atlantic and Gulf of Mexico populations are discrete (Able and Fahay, 2010). Maturity may be reached at the end of the first year and most fish are mature by the third year of life (Barbieri et al., 1994). Adult Atlantic Croaker migrate from coastal areas to the continental shelf to spawn in early fall through winter (Able and Fahay, 1998, 2010). Eggs and larvae are pelagic and rely on fall winds for transportation across the shelf (Able and Fahay, 2010). Larvae are selective feeders consuming zooplankton such as tintinnids, pteropods, and pelecypods (Govoni et al., 1986; Able and Fahay, 2010). A few weeks after spawning, late stage larvae ingress into estuarine and freshwater nursery habitat along the mid Atlantic coast during early fall – winter (Able and Fahay, 2010). Juvenile Atlantic Croaker overwinter in deep areas estuaries and tributaries typically near grassbeds and mud flats (Able and Fahay, 2010). The northern extent of

suitable nursery habitat is limited by winter temperature < 3 °C (Able and Fahay, 2010). While in estuaries, juveniles consume demersal prey such as polychaetes, copepods, mysids and detritus (Able and Fahay, 2010). Striped Bass can be a major predator of juvenile Atlantic Croaker (Able and Fahay, 2010). Juveniles egress from the estuaries the next fall, and follow adults offshore (Able and Fahay, 2010). Adults are common inshore demersal fish over mud and sandy mud in spring and summer, but move offshore and south along the continental shelf to spawn and overwinter in late fall through winter (Able and Fahay, 2010). Benthic macroinvertebrates such as crustaceans, polychaetes, molluscs, and fish are common prey for adult Atlantic Croaker (Able and Fahay, 2010). Atlantic Croaker is managed under the Atlantic States Marine Fisheries Commission Interstate Fishery Management Plan for Atlantic Croaker (2005) and Addendum I (2011; ASMFC, 2011). Atlantic roaker is not experiencing overfishing and biomass estimates are increasing for the Atlantic stock (ASMFC, 2010).

<u>Literature Cited</u>:

Able KW. A re-examination of fish estuarine dependence: evidence for connectivity between estuarine and ocean habitats. Estuar Coast Shelf Sci. 2005; 64(1), 5-17. doi:10.1016/j.ecss.2005.02.002

Able KW, Fahay MP. The first year in the life of estuarine fishes in the middle Atlantic bight. Brunswick: Rutgers University Press; 1998. 342 p.

Able KW, Fahay MP. Ecology of estuarine fishes: temperate waters of the western North Atlantic. Baltimore: The Johns Hopkins University Press; 2010. 566p.

Atlantic States Marine Fisheries Commission (ASMFC). 2010. Atlantic croaker 2010 benchmark stock assessment. Washington (DC): ASMFC. 366p. Accessed online (May 2014): http://www.asmfc.org/uploads/file//5282798aatlanticCroaker2010BenchmarkStockAssessment.pdf

Atlantic States Marine Fisheries Commission (ASMFC). 2011. Addendum I to amendment 1 to the Atlantic croaker fishery management plan. Washington (DC): ASMFC. 7p. Accessed online (May 2014): http://www.asmfc.org/uploads/file/croakerAddendumI.pdf

Barbieri LR; Chittenden, Jr. ME; Lowerre-Barbieri SK. Maturity, spawning, and ovarian cycle of Atlantic croaker, *Micropogonias undulatus*, in the Chesapeake Bay and adjacent coastal waters. Fish Bull. 1994; 92: 671-685. Available: http://fishbull.noaa.gov/924/barbieri.pdf

Diamond SL, Murphy CA, Rose KA. Simulating the effects of global climate change on Atlantic croaker population dynamics in the mid-Atlantic Region. Ecol Modell. 2013; 264, 98-114. doi:10.1016/j.ecolmodel.2013.05.001

Govoni, J. J.; P. B. Ortner; F. Al-Yamani; L. C. Hill. 1986. Selective feeding of spot, *Leiostomus xanthurus*, and Atlantic croaker, *Micropogonias undulatus*, larvae in the northern Gulf of Mexico. Mar. Ecol. Prog. Ser. 28: 175-183.

Hare JA, Able KW. Mechanistic links between climate and fisheries along the east coast of the United States: explaining population outbursts of Atlantic croaker (*Micropogonias undulatus*). Fish Oceanogr, 2007; 16(1), 31-45. doi: 10.1111/j.1365-2419.2006.00407.x

Return to Table of Contents

Hare JA, Alexander MA, Fogarty MJ, Williams EH, Scott JD. (2010). Forecasting the dynamics of a coastal fishery species using a coupled climate-population model. Ecol Appl. 2010; 20(2), 452-464. doi: 10.1890/08-1863.1

Munyandorero J. In search of climate effects on Atlantic Croaker (*Micropogonias undulatus*) stock off the U.S. Atlantic coast with Bayesian state-space biomass dynamic models. Fish Bull. 2014; 112:49-70. doi: 10.7755/FB.112.1.4

Return to Table of Contents

Atlantic Hagfish – *Myxine glutinosa*

Overall Vulnerability Rank = Moderate

Biological Sensitivity = Moderate
Climate Exposure = High
Data Quality = 67% of scores ≥ 2

Myxine glutinosa	Expert Scores	Data Quality	Expert Scores Plots (Portion by Category)
Stock Status	2.4	0.2	
Other Stressors	1.6	1.1	
Population Growth Rate	2.6	0.6	
Spawning Cycle	1.1	2.6	
Complexity in Reproduction	1.9	1.0	
Early Life History Requirements	1.1	2.1	
Sensitivity to Ocean Acidification	1.5	1.9	
Prey Specialization	1.0	2.6	
Habitat Specialization	1.2	3.0	
Sensitivity to Temperature	1.6	2.7	
Adult Mobility	3.0	2.2	
Dispersal & Early Life History	2.7	1.4	
Sensitivity Score	Moderate		
Sea Surface Temperature	3.9	3.0	
Variability in Sea Surface Temperature	1.0	3.0	
Salinity	1.4	3.0	
Variability Salinity	1.2	3.0	
Air Temperature	1.0	3.0	
Variability Air Temperature	1.0	3.0	
Precipitation	1.0	3.0	
Variability in Precipitation	1.0	3.0	
Ocean Acidification	4.0	2.0	
Variability in Ocean Acidification	1.0	2.2	
Currents	2.1	1.0	
Sea Level Rise	1.1	1.5	
Exposure Score	High		
Overall Vulnerability Rank	Moderate		

Low
Moderate
High
Very High

Atlantic Hagfish (*Myxine glutinosa*)

Overall Climate Vulnerability Rank: **Moderate** (92% certainty from bootstrap analysis).

Climate Exposure: **High**. Two exposure factors contributed to this score: Ocean Surface Temperature (3.9) and Ocean Acidification (4.0). All life stages of Atlantic Hagfish use marine habitats.

Biological Sensitivity: **Moderate**. Two sensitivity attributes scored above 2.5: Adult Mobility (3.0) and Dispersal and Early Life History (2.7). The life cycle is uncertain, but it is likely that there is no larval stage and adults hatch from eggs (Martini and Flescher, 2002). Hagfish burrow in soft sediment and likely have limited mobility (Martini and Flescher, 2002).

Distributional Vulnerability Rank: **Low** (82% certainty from bootstrap analysis). Adults are relatively immobile and there is likely no dispersive larval stage limiting the potential of a change in distribution.

Directional Effect in the Northeast U.S. Shelf: The effect of climate change on Atlantic Hagfish on the Northeast U.S. Shelf is estimated to be neutral, but with a moderate degree of uncertainty (66-90% certainty in expert scores). There is very little information available for hagfish making the estimate of the directional effect of climate change difficult.

Data Quality: 67% of the data quality scores were 2 or greater indicate that data quality is moderate.

Climate Effects on Abundance and Distribution: There is very little information regarding the dynamics of Atlantic Hagfish as well as little information regarding environmental effects on abundance and distribution.

Life History Synopsis**:** Atlantic Hagfish are an ancient, benthic, marine species found throughout the Atlantic Ocean from Kola Peninsula, Russia, to the Shetland Islands off the Antarctic Peninsula (Martini and Flescher, 2002). Very little is known about the spawning and early development of Atlantic Hagfish. Individuals >400 mm are sexually mature and can have both male and female reproductive organs, but only one is developed at a time (Martini and Flescher, 2002). Most individuals are female, with developed ovary and rudimentary testis, and about a quarter of the population are sterile (Martini and Flescher, 2002). Spawning can occur year round and is believed to occur in deep water (Martini and Flescher, 2002). Eggs are rare in samples and few are fertilized, leading to the assumption of low fecundity among Atlantic Hagfish (Martini and Flescher, 2002). There is likely no larval stage; small immature adults hatch out of eggs (Martini and Flescher, 2002). Hagfish live within horizontal burrows in soft muddy sediment on the sea floor (Martini and Flescher, 2002). Hagfish need high salinity water; sharp changes in salinity or temperature can have severe effects on survival (Martini and Flescher, 2002). Hagfish are opportunistic feeders on Northern Shrimp, dead or dying fish, and benthic and burrowing invertebrates, often interfering with commercial trap, gillnet, and longline catches (Martini and Flescher, 2002). Atlantic Cod, Spiny Dogfish, harbor seal, and harbor porpoise eat Atlantic Hagfish, and groundfish may prey on their eggs (Martini and Flescher, 2002). There is a small fishery for Atlantic Hagfish in the United States to sell to the Asian leather industry, but the species is not actively managed (Martini and Flescher, 2002).

Return to Table of Contents

Literature Cited:

Martini FH, Flescher D. Atlantic Hagfish/ Myxine glutinosa Linnaeus 1758. Pages 10-16 In: BB Collette, G Klein-MacPhee (editors), Fishes of the Gulf of Maine, 3rd edition. Smithsonian Institution Press, Washington D.C. 2002; 882 p.

Return to Table of Contents

Atlantic Halibut – *Hippoglossus hippoglossus*

Overall Vulnerability Rank = High

Biological Sensitivity = High
Climate Exposure = High
Data Quality = 83% of scores ≥ 2

Hippoglossus hippoglossus	Expert Scores	Data Quality	Expert Scores Plots (Portion by Category)
Stock Status	4.0	3.0	
Other Stressors	1.9	1.5	
Population Growth Rate	3.9	2.5	
Spawning Cycle	2.0	2.5	
Complexity in Reproduction	1.4	2.4	
Early Life History Requirements	2.1	1.6	
Sensitivity to Ocean Acidification	1.5	2.0	
Prey Specialization	1.0	3.0	
Habitat Specialization	1.2	2.8	
Sensitivity to Temperature	2.1	3.0	
Adult Mobility	1.9	2.3	
Dispersal & Early Life History	1.3	2.2	
Sensitivity Score	High		
Sea Surface Temperature	3.9	3.0	
Variability in Sea Surface Temperature	1.0	3.0	
Salinity	1.1	3.0	
Variability Salinity	1.2	3.0	
Air Temperature	1.0	3.0	
Variability Air Temperature	1.0	3.0	
Precipitation	1.0	3.0	
Variability in Precipitation	1.0	3.0	
Ocean Acidification	4.0	2.0	
Variability in Ocean Acidification	1.0	2.2	
Currents	2.1	1.0	
Sea Level Rise	1.1	1.5	
Exposure Score	High		
Overall Vulnerability Rank	High		

Low
Moderate
High
Very High

Return to Table of Contents

Atlantic Halibut (*Hippoglossus hippoglossus*)

Overall Climate Vulnerability Rank: **High** (100% certainty from bootstrap analysis).

Climate Exposure: **High**. Two exposure factors contributed to this score: Ocean Surface Temperature (3.9) and Ocean Acidification (4.0). All life stages of Atlantic Halibut use marine habitats.

Biological Sensitivity: **High**. Two sensitivity attributes scored above 3.0: Stock Status (4.0) and Population Growth Rate (3.9). Atlantic Halibut are overfished but overfishing is not occurring (Blaylock and Legault, 2012). Atlantic Halibut are slow-growing, late maturing (6-8 years) and long-lived (50 years, Cargnelli et al., 1999).

Distributional Vulnerability Rank: **High** (100% certainty from bootstrap analysis). Atlantic Halibut are habitat generalists, highly mobile and have dispersive early life stages.

Directional Effect in the Northeast U.S. Shelf: The effect of climate change on Atlantic Halibut on the Northeast U.S. Shelf is very likely to be negative (>95% certainty in expert scores). Atlantic Halibut is a cold water species and warming could cause distribution shifts out of the region. Productivity may also be negatively affected by warming and ocean acidification.

Data Quality: 83% of the data quality scores were 2 or greater indicate that data quality is moderate.

Climate Effects on Abundance and Distribution: Atlantic Halibut growth is sensitive to temperature and optimal temperature for growth decreased with increasing fish size (Björnsson and Tryggvadóttir, 1996). Long-term exposure to CO2-acidified seawater also reduced growth. Thus, with changing climatic conditions, Atlantic Halibut growth and population productivity would be expected to decrease in the Northeast U.S. Shelf Ecosystem. Nye et al. (2009) found Atlantic Halibut distributions shifting northward in recent years.

Life History Synopsis**:** Atlantic Halibut is a long-lived, slow-growing, benthic, marine, flatfish species found on both sides of the Atlantic Ocean, and ranges from Labrador to Long Island, New York, in the western Atlantic (Cargnelli et al., 1999; Klein-MacPhee, 2002). Sexual dimorphism in the species results in larger, older females (Cargnelli et al., 1999). Males mature at approximately 6 years old and females at approximately 7-8 years old (Sigourney et al., 2006). Spawning occurs on offshore banks and the shelf slope in late winter – spring (although some individuals spawn into summer; Cargnelli et al., 1999; Klein-MacPhee, 2002). Atlantic Halibut are annual, group-synchronous, batch spawners over hard sand, clay, or gravel bottom in 4-7 ^{o}C water (Cargnelli et al., 1999; Klein-MacPhee, 2002). Eggs are large and, most likely, bathy-pelagic (50-200 m), but sink with development to near the sea floor (Klein-MacPhee, 2002). Eggs incubate for 2 to 3 weeks, but the timing is temperature dependent (Cargnelli et al., 1999). Larvae are pelagic and prefer salinities of 30-34 (Cargnelli et al., 1999). Larvae are rare, resulting in limited information about larval duration, but development is slow. Atlantic Halibut larvae may stay in the water column for up to a year before fully metamorphosing and settling to the bottom (Cargnelli et al., 1999; Klein-MacPhee, 2002). Juvenile Atlantic Halibut mature in two phases. Early juveniles settle to nursery grounds with sandy substrate at 20-60 m depth (Cargnelli et al., 1999; Klein-MacPhee, 2002). At 3-4 years old, Atlantic Halibut emigrates out of the nursery and begins a dispersive phase, migrating longer distances than adults and occasionally moving among regions (Cargnelli et al., 1999; Klein-MacPhee, 2002). Adult Atlantic Halibut are widely dispersed on the shelf (25-700 m) in sand, gravel or clay substrates (Cargnelli et al., 1999; Klein-MacPhee, 2002). Atlantic Halibut move inshore from deep

winter habitat in the spring, probably in response to food availability and regulated by the presence of suitably cool temperatures (Klein-MacPhee, 2002). Diet changes ontogenetically; fish <30 cm eat mainly invertebrates such as annelids and crustaceans (Cargnelli et al., 1999; Klein-MacPhee, 2002). The ratio of invertebrates to bony fish declines until halibut >80 cm eat fish almost exclusively (Cargnelli et al., 1999; Klein-MacPhee, 2002). Greenland Shark, seals, Monkfish (Goosefish), and Spiny Dogfish are common predators of halibut (Cargnelli et al., 1999; Klein-MacPhee, 2002). Atlantic Halibut was heavily fished resulting in a depleted stock prior to 1900 and have yet to recover (Cargnelli et al., 1999). There is evidence of two genetic stocks: Gulf of St. Lawrence and Scotian Shelf, and there is no longer a breeding population in the Gulf of Maine (Klein-MacPhee, 2002). Atlantic Halibut are managed under the New England Fisheries Management Council Northeast Multispecies Fishery Management Plan, and based on the most recent stock assessments, are overfished, but not undergoing overfishing (Blaylock and Legault, 2012).

Literature Cited:

Björnsson B, Tryggvadóttir SV. Effects of size on optimal temperature for growth and growth efficiency of immature Atlantic halibut (*Hippoglossus hippoglossus* L.). Aquaculture. 1999; 142(1): 33-42. DOI: 10.1016/0044-8486(95)01240-0

Northeast Fisheries Science Center (NEFSC). Assessment or Data Updates of 13 Northeast Groundfish Stocks through 2010. US Dept Commer, Northeast Fish Sci Cent Ref Doc. 2012; 12-06: 789 p. Accessed Online (August 2015): http://www.nefsc.noaa.gov/publications/crd/crd1206/

Cargnelli L, Griesbach S, Morse W. Essential Fish Habitat Source Document: Atlantic Halibut, Hippoglossus hippoglossus, Life History and Habitat Characteristics. NOAA Tech Memo. 1999; NMFS-NE-125. Accessed Online (August 2015): http://www.nefsc.noaa.gov/nefsc/publications/tm/tm125/index.htm

Gräns A, Jutfelt F, Sandblom E, Jönsson E, Wiklander K, Seth H, et al. Aerobic scope fails to explain the detrimental effects on growth resulting from warming and elevated CO2 in Atlantic halibut. J Exp Boil. 2014; 217(5): 711-717. DOI: 10.1242/jeb.096743

Klein-MacPhee G. Atlantic halibut/ *Hippoglossus hippoglossus* (Linnaeus 1758). Pages 569-572. In: BB Collete, G Klein-MacPhee (Editors). Bigelow and Schrodeder's Fishes of the Gulf of Maine. 3rd Edition. Smithsonian Institution Press, Washington, D.C. 2002; 882 p.

Nye JA, Link JS, Hare JA, Overholtz WJ. Changing spatial distribution of fish stocks in relation to climate and population size on the Northeast United States continental shelf. Mar Ecol Prog Ser. 2009; 393: 111-129. DOI: 0.3354/meps08220

Sigourney D, Ross M, Brodziak J, Burnett J. Length at age, sexual maturity and distribution of the Atlantic halibut off the northeast USA. Journal of Northwest Atlantic Fisheries Science. 2006; 36(7). Accessed Online (August 2015): http://journal.nafo.int/36/ sigourney/7-sigourney.html

Atlantic Herring – *Clupea harengus*

Overall Vulnerability Rank = Low

Biological Sensitivity = Low
Climate Exposure = High
Data Quality = 88% of scores ≥ 2

Clupea harengus	Expert Scores	Data Quality	Expert Scores Plots (Portion by Category)
Stock Status	1.4	2.8	
Other Stressors	1.7	1.9	
Population Growth Rate	1.9	2.9	
Spawning Cycle	2.4	3.0	
Complexity in Reproduction	2.2	2.4	
Early Life History Requirements	2.8	2.4	
Sensitivity to Ocean Acidification	1.5	2.4	
Prey Specialization	1.3	3.0	
Habitat Specialization	1.7	3.0	
Sensitivity to Temperature	2.0	3.0	
Adult Mobility	1.1	3.0	
Dispersal & Early Life History	1.4	3.0	
Sensitivity Score	Low		
Sea Surface Temperature	4.0	3.0	
Variability in Sea Surface Temperature	1.0	3.0	
Salinity	1.2	3.0	
Variability Salinity	1.2	3.0	
Air Temperature	1.9	3.0	
Variability Air Temperature	1.0	3.0	
Precipitation	1.0	3.0	
Variability in Precipitation	1.1	3.0	
Ocean Acidification	4.0	2.0	
Variability in Ocean Acidification	1.0	2.2	
Currents	2.1	1.0	
Sea Level Rise	1.3	1.5	
Exposure Score	High		
Overall Vulnerability Rank	Low		

Low
Moderate
High
Very High

Atlantic Herring (*Clupea harengus*)

Overall Climate Vulnerability Rank: **Low** (72% certainty from bootstrap analysis).

Climate Exposure: **High**. Two exposure factors contributed to this score: Ocean Surface Temperature (4.0) and Ocean Acidification (4.0). Exposure to ocean surface temperature and ocean acidification occurs during all life stages.

Biological Sensitivity: **Low**. The highest sensitivity attribute was Early Life History Requirements (2.8). Atlantic Herring spawn benthic eggs in distinct locations.

Distributional Vulnerability Rank: **High** (91% certainty from bootstrap analysis). Three of the attributes indicated vulnerability to distribution shift. Atlantic Herring have low Habitat Specialization. Adults are mobile and larval duration is long resulting in potentially broad larval dispersal.

Directional Effect in the Northeast U.S. Shelf: The effect of climate change on Atlantic Herring on the Northeast U.S. Shelf is estimated to be negative, but this estimate is uncertain (66-90% certainty in expert scores). Higher temperatures may decrease productivity and limit habitat availability for Atlantic Herring. But Atlantic Herring spawn in specific locations creating uncertainty as to the effect of climate change on adult distributions. Ocean acidification also may have a negative effect, but more research is needed.

Data Quality: 88% of the data quality scores were 2 or greater

Climate Effects on Abundance and Distribution: Several studies have indicated an effect of climate change on Atlantic Herring productivity. Gröger et al. (2009) linked recruitment dynamics to climate indices (NAO, AMO), but the mechanisms were not specified. Nash et al. (2005) found a negative relationship between age-0 and age-1 Atlantic Herring abundance and water temperature; they hypothesized a link through prey abundance. Other studies have suggested Atlantic Herring dynamics are linked more closely to species interactions (Möllmann et al., 2005; Richardson et al., 2008). Atlantic Herring larvae may be affected by ocean acidification; Franke and Clemmisen (2011) found evidence that the metabolism of embryos was negatively affected at elevated pCO_2 levels. Atlantic Herring distributions may be impacted by increasing temperatures (Murawski, 1993), but homing to spawning locations may limit ability to shift distributions (Wheeler and Winters, 1984; McQuinn, 1997).

Life History Synopsis**:** Atlantic Herring is a highly-mobile, marine, obligate-schooling species found on both sides of the North Atlantic, but from Labrador to Cape Hatteras, North Carolina, in the western North Atlantic (Stevenson and Scott, 2005). Atlantic Herring mature between 3 and 4 years (NEFSC, 2012). In United States waters, spawning occurs during summer-fall from inshore to mid-shelf in high-energy, saline environments with strong tidal currents (Stevenson and Scott, 2005). Major spawning grounds exist on Georges Bank, Nantucket Shoals, and the Gulf of Maine (Stevenson and Scott, 2005). Eggs are deposited on boulder, rocky, gravel, shell fragment, sand, or macrophyte substrate on the sea floor, and then are fertilized (Stevenson and Scott, 2005). Eggs are demersal and adhere to the substrate till hatching after about 2 weeks (Stevenson and Scott, 2005). Yolk-sac stage larvae stay near the bottom for several days, but become increasingly pelagic with strong diel vertical migrations (Munroe, 2002; Stevenson and Scott, 2005). Metamorphosis only occurs from April – October, so the larval period can last 4-8 months depending on when spawning occurred (Munroe, 2002; Stevenson and Scott, 2005). Metamorphosed juveniles form large schools and egress from estuaries and near-shore waters in

summer and fall to enter deep bays or offshore bottom overwintering habitat, then return to inshore areas the following spring (Stevenson and Scott, 2005; Able and Fahay, 2010). Juveniles migrate only in the inshore-offshore direction, and so experience colder temperatures than adults. To survive these cold winter temperatures, juvenile Atlantic Herring produce antifreeze blood proteins (Munroe, 2002; Stevenson and Scott, 2005). Juvenile Atlantic Herring are pelagic but perform diel vertical migrations putting them deeper in the water column during the day (Stevenson and Scott, 2005). Adult Atlantic Herring occupy the water column from inshore (estuaries and embayments) to offshore and surface to 200 m (Munroe, 2002). Seasonal migrations span summer and fall spawning grounds in the northern part of the species' range to overwintering grounds to the south (Southern New England and the Mid-Atlantic; Stevenson and Scott, 2005). Changes in temperature drive distribution patterns, but salinity also plays a roll (Munroe, 2002; Stevenson and Scott, 2005). Diel vertical migrations of adults put the fish at the surface at night (Munroe, 2002; Stevenson and Scott, 2005). During all life history stages, Atlantic Herring are opportunistic planktivores, limited by the size of the prey and mouth size (Stevenson and Scott, 2005). Adults can alternate between filter feeding and biting behavior, and sometimes consume fish eggs and larvae (including Atlantic Herring larvae; Munroe, 2002; Stevenson and Scott, 2005). Atlantic Herring are also important prey fish throughout their life history, feeding a variety of piscivorous fish, elasmobranchs, marine mammals, squid, and seabirds (Stevenson and Scott, 2005). In fact, Spiny Dogfish, alone, may consume as many Atlantic Herring as are taken in the fishery (Stevenson and Scott, 2005). The Georges Bank-Nantucket Shoals stock collapsed in the early 1970s due to exploitation by foreign fishing fleets, but has since recovered (Stevenson and Scott, 2005). In the United States, the Atlantic States Marine Fishery Commission and the New England Fishery Management Council under Amendment 2 to the Interstate Fishery Management Plan for Atlantic Herring manage Atlantic Herring as a single coastal stock complex jointly (ASMFC, 2013). Based on the most recent benchmark assessment, Atlantic Herring is not overfished and overfishing is not occurring (NEFSC, 2012).

Literature Cited:

Able KW, Fahay MP. Ecology of estuarine fishes: temperate waters of the western North Atlantic. Baltimore: The Johns Hopkins University Press; 2010. 566p.

Atlantic States Marine Fisheries Commission (ASMFC). 2013. Addendum VI to Amendment 2 to the Interstate Fishery Management Plan for Atlantic Herring. 5 p. Accessed online (May 2014): http://www.asmfc.org/uploads/file//5331ec3cAtlanticHerring_AddVItoAmd2_Aug2013.pdf

Franke A, Clemmesen C. Effect of ocean acidification on early life stages of Atlantic herring (Clupea harengus L.). Biogeosciences. 2011; (12): 3697-3707. doi:10.5194/bg-8-3697-2011

Gröger JP, Kruse GH, Rohlf N. Slave to the rhythm: how large-scale climate cycles trigger herring (Clupea harengus) regeneration in the North Sea. ICES J Mar Sci. 2010; 67 (3): 454-465. doi: 10.1093/icesjms/fsp259

McQuinn IH. Metapopulations and the Atlantic herring. Rev Fish Biol Fish. 1997; 7(3): 297-329. 10.1023/A:1018491828875

Möllmann C, Kornilovs G, Fetter M, Köster FW. Climate, zooplankton, and pelagic fish growth in the central Baltic Sea. ICES J Mar Sci. 2005; 62(7): 1270-1280. doi: 10.1016/j.icesjms.2005.04.021

Munroe TA. Atlantic herring/ Clupea harengus Linnaeus 1758. In: Collete BB, Klein-MacPhee G, editors, Fishes of the Gulf of Maine, 3rd edition. Washington: Smithsonian Institution Press; 2002. pp. 141-156.

Murawski SA. Climate change and marine fish distributions: forecasting from historical analogy. Trans Am Fish Soc. 1993; 122(5): 647-658. doi: 10.1577/1548-8659(1993)122<0647:CCAMFD>2.3.CO;2

Nash RDM, Dickey-Collas M. The influence of life history dynamics and environment on the determination of year class strength in North Sea herring (Clupea harengus L.). Fish Oceanogr. 2005; 14(4): 279-291. doi: 10.1111/j.1365-2419.2005.00336.x

Northeast Fisheries Science Center. 2012. 54th Northeast Regional Stock Assessment Workshop (54th SAW) Assessment Report. US Dept Commer, Northeast Fish Sci Cent Ref Doc. 12-18; 600 p. Available from: National Marine Fisheries Service, 166 Water Street, Woods Hole, MA 02543-1026, Accessed online (May 2014): http://nefsc.noaa.gov/publications/crd/crd1218/

Richardson DE, Hare JA, Fogarty MJ, Link JS. Role of egg predation by haddock in the decline of an Atlantic herring population. Proc Natl Acad Sci U S A. 2011; 108(33): 13606-13611. doi: 10.1073/pnas.1015400108

Stevenson DK, Scott ML. 2005. Essential fish habitat source document: Atlantic herring, Clupea harengus, life history and habitat characteristics, Second Edition. NOAA Tech Memo. NMFS-NE-192. 84p. Available: http://www.nefsc.noaa.gov/publications/tm/tm192/tm192.pdf

Wheeler JP, Winters GH. Homing of Atlantic herring (Clupea harengus harengus) in Newfoundland waters as indicated by tagging data. Can J Fish Aquat Sci. 1984; 41(1): 108-117. doi: 10.1139/f84-010

Atlantic Mackerel – *Scomber scombrus*

Overall Vulnerability Rank = Moderate

Biological Sensitivity = Moderate
Climate Exposure = High
Data Quality = 83% of scores ≥ 2

Scomber scombrus	Expert Scores	Data Quality	Expert Scores Plots (Portion by Category)
Stock Status	2.6	1.4	
Other Stressors	1.5	1.7	
Population Growth Rate	1.6	2.7	
Spawning Cycle	2.5	3.0	
Complexity in Reproduction	2.0	2.7	
Early Life History Requirements	2.6	2.5	
Sensitivity to Ocean Acidification	1.4	2.0	
Prey Specialization	1.1	3.0	
Habitat Specialization	1.5	3.0	
Sensitivity to Temperature	1.5	3.0	
Adult Mobility	1.1	2.8	
Dispersal & Early Life History	1.5	2.4	
Sensitivity Score	Moderate		
Sea Surface Temperature	4.0	3.0	
Variability in Sea Surface Temperature	1.0	3.0	
Salinity	1.4	3.0	
Variability Salinity	1.2	3.0	
Air Temperature	1.9	3.0	
Variability Air Temperature	1.0	3.0	
Precipitation	1.0	3.0	
Variability in Precipitation	1.1	3.0	
Ocean Acidification	4.0	2.0	
Variability in Ocean Acidification	1.0	2.2	
Currents	2.1	1.0	
Sea Level Rise	1.1	1.5	
Exposure Score	High		
Overall Vulnerability Rank	Moderate		

Low
Moderate
High
Very High

Atlantic Mackerel (*Scomber scombrus*)

Overall Climate Vulnerability Rank: **Moderate** (80% certainty from bootstrap analysis).

Climate Exposure: **High**. Two exposure factors contributed to this score: Ocean Surface Temperature (4.0) and Ocean Acidification (4.0). Exposure to ocean surface temperature and ocean acidification occurs during all life stages.

Biological Sensitivity: **Moderate**. The highest sensitivity attributes were Stock Status (2.6), Early Life History Requirements (2.6), and Spawning Cycle (2.5). The stock status of Atlantic Mackerel is currently unknown, but fewer older fish are present in the population compared to historical periods (TRAC, 2010). Atlantic Mackerel spawn in a distinct season and larvae develop quickly. The spawning cycle and early life stages may also be influenced by warming waters.

Distributional Vulnerability Rank: **High** (81% certainty from bootstrap analysis). Three of the attributes indicated vulnerability to distribution shift: Atlantic Mackerel have low Habitat Specialization, are highly mobile and make large seasonal migrations, and larvae are planktonic with potentially broad dispersal.

Directional Effect in the Northeast U.S. Shelf: The effect of climate change on Atlantic Mackerel on the Northeast U.S. Shelf is very likely to be negative (>95% certainty in expert scores). Dramatic changes have already been observed in Atlantic Mackerel distribution in both the Northeast and Northwest Atlantic and these changes have been linked to warming temperatures. The effect of ocean acidification over the next 30 years is likely to be minimal.

Data Quality: 83% of the data quality scores were 2 or greater.

Climate Effects on Abundance and Distribution: Few studies have examined the effect of climate on the productivity of Atlantic Mackerel. However, numerous studies have indicated that Atlantic Mackerel populations shift as a result of changing environmental conditions. In the Northeast Atlantic, Atlantic Mackerel have expanded into Icelandic waters in the past decade (Astthorsson et al., 2012). Shifts in Atlantic Mackerel distribution also have been observed in the Northwest Atlantic (Overholtz et al., 2011). The timing of spawning and migrations has been linked to temperature in the Northeast Atlantic (Jansen and Gislason, 2011; Radlinski et al., 2013).

Life History Synopsis**:** Atlantic Mackerel is a highly mobile, pelagic, schooling, marine species found from the Labrador to Cape Lookout, North Carolina. Mackerel reach sexual maturity after 2-3 years (Studholme et al., 1999). There are two spawning contingent, but both migrate to inshore spawning grounds (Studholme et al., 1999). The southern contingent aggregates off the coast of the mid-Atlantic states and western Gulf of Maine from April – June (Studholme et al., 1999). The northern contingent spawns from May to August in the southern Gulf of St. Lawrence (Studholme et al., 1999). Mackerel are batch spawners, and the onset of spawning may be triggered by warm water temperature, ensuring eggs hatch during periods of high zooplankton abundance (Studholme et al., 1999). Eggs are pelagic, near the surface, and hatch within a week of spawning (Studholme et al., 1999). Larvae occur in offshore waters from Chesapeake Bay to the southern Gulf of St. Lawrence, with light intensity, age, and the location of the thermocline effecting depth distribution (Studholme et al., 1999; MAFMC, 2011). Mackerel larvae are generalist planktivores, limited by mouth size, and may cannibalistically consume smaller mackerel (Studholme et al., 1999). Larvae transition to the juvenile stage gradually, but after approximately two months switch from planktonic to swimming and schooling behavior (Studholme et

al., 1999). While generally common offshore, Atlantic Mackerel juveniles are found farther inshore than adults and in some estuaries in the mid-Atlantic during spring (Studholme et al., 1999). The depth distribution of juvenile Atlantic Mackerel changes seasonally: mid-depths in fall, deeper in winter, more vertically dispersed in spring, and shallower in summer (Studholme et al., 1999). Juveniles are opportunistic feeders, using both filter feeding and biting behavior to capture small crustaceans, pelagic molluscs, chaetognaths and other small pelagic fish and invertebrates (Studholme et al., 1999). Squid, seabirds and larger piscivores, including Atlantic Cod, are major predators of juvenile Atlantic Mackerel (Studholme et al., 1999). Adult Atlantic Mackerel from the two spawning contingents appear to follow different seasonal migration paths: the southern contingent stays farther inshore, the northern contingent stays more offshore, but the two paths cross during spring and fall (Studholme et al., 1999). The primary fishery occurs in winter and is composed of unknown proportions of the two spawning contingents. Adult Atlantic Mackerel distribution throughout the water column also changes seasonally: mid-depths during summer and fall, shallower in winter, and dispersed throughout water column in spring, with diel vertical migrations following food (Studholme et al., 1999). The diet of adult Atlantic Mackerel includes a variety of planktonic organisms, including: euphausiids, small crustaceans, squid, and small fish (Studholme et al., 1999). Predation is a very important source of natural mortality, with a large number of predators including several large and small fish, whales, seals, seabirds, sharks, and skates (Studholme et al., 1999). Atlantic Mackerel is assessed as one unit and managed under the Mid-Atlantic Fishery Management Plan for Atlantic Mackerel, Squid, and Butterfish (MAFMC, 2011). As of the 2010 assessment, the status of the stock is unknown (TRAC, 2010).

Literature Cited:

Astthorsson OS, Valdimarsson H, Gudmundsdottir A, Óskarsson GJ. Climate-related variations in the occurrence and distribution of mackerel (Scomber scombrus) in Icelandic waters. ICES J Mar Sci. 2012; 69(7): 1289-1297. doi: 10.1093/icesjms/fss084

Jansen T, Gislason H. Temperature affects the timing of spawning and migration of North Sea mackerel. Cont Shelf Res. 2011; 31(1): 64-72. doi:10.1016/j.csr.2010.11.003

Mid-Atlantic Fishery Management Council (MAFMC). 2011. Amendment 11 to the Atlantic mackerel, squid, and butterfish (MSB) fishery management plan (FMP). MAFMC [Dover, DE]. 559 p + appendices. Accessed online (June, 2014): http://www.mafmc.org/fisheries/fmp/msb

Transboundary Assessment Committee (TRAC). Atlantic Mackerel in the Northwest Atlantic [NAFO Subareas 2 - 6]. 2010. Status Report 2010/01 . http://www2.mar.dfo-mpo.gc.ca/science/trac/TSRs/TSR_2010_01_E.pdf

Overholtz WJ, Hare JA, Keith CM. Impacts of interannual environmental forcing and climate change on the distribution of Atlantic mackerel on the US Northeast continental shelf. Mar Coast Fish. 2011; 3(1): 219-232. doi: 10.1080/19425120.2011.578485

Radlinski MK, Sundermeyer MA, Bisagni JJ, Cadrin SX. Spatial and temporal distribution of Atlantic mackerel (Scomber scombrus) along the northeast coast of the United States, 1985–1999. ICES J Mar Sci. 2013; 70(6): 1151-1161. doi: 10.1093/icesjms/fst029

Studholme AL, Packer DB, Berrien PL, Johnson DL, Zetlin CA, Morse WW. 1999. Essential Fish Habitat Source Document: Atlantic mackerel, Scomber scombrus, life history and habitat characteristics. NOAA

Tech. Memo. NMFS-NE-141. 35p. Accessed online (June 2014): http://www.nefsc.noaa.gov/nefsc/publications/tm/tm141/tm141.pdf

Atlantic Menhaden – *Brevoortia tyrannus*

Overall Vulnerability Rank = Moderate

Biological Sensitivity = Low
Climate Exposure = Very High
Data Quality = 88% of scores ≥ 2

Brevoortia tyrannus	Expert Scores	Data Quality	Expert Scores Plots (Portion by Category)
Stock Status	2.4	1.4	
Other Stressors	2.1	2.4	
Population Growth Rate	1.2	2.8	
Spawning Cycle	1.8	2.8	
Complexity in Reproduction	1.9	3.0	
Early Life History Requirements	2.9	2.9	
Sensitivity to Ocean Acidification	1.8	2.4	
Prey Specialization	1.5	3.0	
Habitat Specialization	2.0	3.0	
Sensitivity to Temperature	1.5	3.0	
Adult Mobility	1.6	3.0	
Dispersal & Early Life History	1.8	3.0	
Sensitivity Score	Low		
Sea Surface Temperature	4.0	3.0	
Variability in Sea Surface Temperature	1.0	3.0	
Salinity	2.4	3.0	
Variability Salinity	1.2	3.0	
Air Temperature	4.0	3.0	
Variability Air Temperature	1.0	3.0	
Precipitation	1.2	3.0	
Variability in Precipitation	1.3	3.0	
Ocean Acidification	4.0	2.0	
Variability in Ocean Acidification	1.0	2.2	
Currents	2.0	1.0	
Sea Level Rise	1.5	1.5	
Exposure Score	Very High		
Overall Vulnerability Rank	Moderate		

Low
Moderate
High
Very High

Return to Table of Contents

Atlantic Menhaden (*Brevoortia tyrannus*)

Overall Climate Vulnerability Rank: **Moderate** (64% certainty from bootstrap analysis).

Climate Exposure: **Very High**. Three exposure factors contributed to this score: Ocean Surface Temperature (4.0), Ocean Acidification (4.0) and Air Temperature (4.0). Exposure to all three factors occur during all life stages. Atlantic Menhaden spawn on the shelf, juveniles use estuarine nurseries, and adults make seasonal migrations feeding in estuarine and coastal areas.

Biological Sensitivity: **Low**. Only one sensitivity attribute scored above 2.5: Early Life History Requirements (2.9). Atlantic Menhaden exhibit an obligate estuarine-dependent life cycle, using estuaries during the late larval and juvenile stages (Able, 2005).

Distributional Vulnerability Rank: **High** (99% certainty from bootstrap analysis). Two attributes indicated vulnerability to distribution shift. Atlantic Menhaden larvae are widely dispersed after spawning and adults make large seasonal migrations along the East Coast of the United States.

Directional Effect in the Northeast U.S. Shelf: The effect of climate change on Atlantic Menhaden on the Northeast U.S. Shelf is very likely to be positive (90-95% certainty in expert scores). Recruitment will likely increase as temperature warm and more spawning occurs in the region. Adult distribution will likely extend northwards and the species may re-occupy the Gulf of Maine during summertime. The effect of ocean acidification over the next 30 years is likely to be minimal.

Data Quality: 88% of the data quality scores were 2 or greater.

Climate Effects on Abundance and Distribution: Wood and Austin (2009) described synchrony in recruitment of three coastal spawning species in Chesapeake Bay: Atlantic Menhaden, Spot, and Summer Flounder. The generalized recruitment pattern was asynchronous with recruitment of diadromous species in the Bay and the authors suggest large-scale climate forcing is responsible for the patterns in recruitment. This study suggests that Atlantic Menhaden productivity may change with changes in precipitation and temperature. Atlantic Menhaden distribution may also change. Dow (1977) reported that Atlantic Menhaden catches in the Gulf of Maine increased in the 1950s coincident with a warm period suggesting that distribution expands northward during warmer periods. Walsh et al. (2015) documented that the time of spawning of Atlantic Menhaden in the Northeast U.S. Shelf has also changed with more spawning in the spring in recent years.

Life History Synopsis**:** Atlantic Menhaden is a schooling, migratory, pelagic, estuarine-dependent, marine species that ranges seasonally from Nova Scotia to northern Florida (Rogers and van den Avyle, 1989). Menhaden mature after 2-3 years (ASMFC, 2011). Spawning occurs year round with a latitudinal gradient in occurrence, but peaks in spring and fall in the north and mid-Atlantic regions (Rogers and van den Avyle, 1989; Able and Fahay, 2010). All ages and size classes accumulate off the Carolinas in winter near the shelf break and spawn. They then migrate inshore and to the north, continuing to spawn on the inner continental shelf and into estuaries. Spawning continues through the fall during the southward and offshore migration (Rogers and van den Avyle, 1989; Able and Fahay, 2010). Spawning north of Long Island, New York may not result in many surviving larvae (Munroe, 2002). Eggs are pelagic and hatch after 2-3 days depending on temperature (Rogers and van den Avyle, 1989; Able and Fahay, 2010). Larval menhaden spend 1-3 months in the upper water column on the continental shelf and make diel vertical migrations to the surface (Rogers and can den Avyle, 1989; Munroe, 2002; Able and Fahay,

2010). Late stage larvae ingress into estuaries from spring to fall in the North Atlantic and winter through spring off the coast of the southeastern United States (Rogers and van den Avyle, 1989). These pre-juveniles inhabit shallow, low-salinity areas of vegetated tidal marshes for 4 months or up to a year (Rogers and van den Avyle, 1989; Able and Fahay, 2010). Early juvenile survival is linked to low salinity, temperatures above 5 ^{o}C, and slow rates of cooling (Able and Fahay, 2010). As the young menhaden mature, they move from the upper estuary to more saline parts of the estuary (Able and Fahay, 2010). Young menhaden are very selective feeders on specific zooplankton, particularly copepods, but as they mature, become increasingly reliant on filter feeding of phytoplankton and detritus (Rogers and van den Avyle, 1989; Able and Fahay, 2010). While a few Atlantic Menhaden have been observed to overwinter in the estuary, most leave the shallow habitat for the open ocean in fall, when temperatures begin to fall more quickly, and migrate south and offshore with the adults (Able and Fahay, 2010). At this same time, the schooling behavior begins (Able and Fahay, 2010). Large schools of similarly sized and aged adult menhaden make long migrations both along- and cross-shelf, with larger fish migrating larger distances (Rogers and van den Avyle, 1989; Able and Fahay, 2010). Atlantic Menhaden are a very important prey species, particularly for Bluefish, Striped Bass, Bluefin Tuna, and sharks (Rogers and van den Avyle, 1989; Able and Fahay, 2010). The Atlantic States Marine Fishery Commission under Amendment 2 to the Interstate Fishery Management Plan for Atlantic Menhaden manages Atlantic Menhaden. As of the most recent assessment, menhaden are not overfished, and overfishing is not occurring (SEDAR, 2015).

Literature Cited:

Able KW. A re-examination of fish estuarine dependence: evidence for connectivity between estuarine and ocean habitats. Estuar Coast Shelf Sci. 2005; 64(1), 5-17. doi:10.1016/j.ecss.2005.02.002

Able KW, Fahay MP. Ecology of estuarine fishes: temperate waters of the western North Atlantic. Baltimore: The Johns Hopkins University Press; 2010. 566p.

Atlantic States Marine Fisheries Commission (ASMFC). 2011. Atlantic menhaden stock assessment and review panel reports. Stock Assessment Report No. 10-02. 326p. Accessed online (June 2014): http://www.asmfc.org/uploads/file/2010AtlanticMenhadenStockAssessmentAndReviewPanelReport.pdf

SEDAR. 2015. SEDAR 40 – Atlantic Menhaden Stock Assessment Report. SEDAR, North Charleston SC. 643 pp. Available: http://www.sefsc.noaa.gov/sedar/Sedar_Workshops.jsp?WorkshopNum=40.

Dow RL. Effects of climatic cycles on the relative abundance and availability of commercial marine and estuarine species. ICES J Mar Sci. 1977; 37(3): 274-280. doi: 10.1093/icesjms/37.3.274

Munroe T. 2002. Herring and herring-like fishes: Order Clupeiformes. In: B.B. Collette BB, Klein-MacPhee G, editors, Fishes of the Gulf of Maine, 3rd ed. Washington: Smithsonian Institution Press; 2002. pp. 104-158.

Rogers SG, Van Den Avyle MJ. 1989. Species profiles: life histories and environmental requirements of coastal fishes and invertebrates (Mid-Atlantic)--Atlantic menhaden. U.S. Fish Wildl. Serv. Biol. Rep.82 (11.108). U.S. Army Corps of Engineers TR EL-82-4. 23 pp. Accessed online (June 2014): http://www.nwrc.usgs.gov/wdb/pub/species_profiles/82_11-108.pdf

Return to Table of Contents

Walsh HJ, Richardson DE, Marancik KE, Hare JA. 2015. Long-term changes in the distributions of larval and adult fish in the Northeast U.S. Shelf Ecosystem. PLOS ONE. doi: 10.1371/journal.pone.0137382

Wood RJ, Austin HM. Synchronous multidecadal fish recruitment patterns in Chesapeake Bay, USA. Can J Fish Aquat Sci. 2009; 66(3): 496-508. doi: 10.1139/F09-013

[Return to Table of Contents]

Atlantic Salmon – *Salmo salar*

Overall Vulnerability Rank = Very High

Biological Sensitivity = Very High
Climate Exposure = Very High
Data Quality = 88% of scores ≥ 2

Salmo salar	Expert Scores	Data Quality	Expert Scores Plots (Portion by Category)
Stock Status	3.8	2.4	
Other Stressors	3.4	2.9	
Population Growth Rate	1.8	2.4	
Spawning Cycle	3.5	3.0	
Complexity in Reproduction	3.5	3.0	
Early Life History Requirements	3.6	3.0	
Sensitivity to Ocean Acidification	1.5	1.8	
Prey Specialization	2.0	2.8	
Habitat Specialization	2.8	3.0	
Sensitivity to Temperature	2.8	3.0	
Adult Mobility	1.5	3.0	
Dispersal & Early Life History	3.6	3.0	
Sensitivity Score	Very High		
Sea Surface Temperature	4.0	3.0	
Variability in Sea Surface Temperature	1.0	3.0	
Salinity	1.0	3.0	
Variability Salinity	1.2	3.0	
Air Temperature	4.0	3.0	
Variability Air Temperature	1.0	3.0	
Precipitation	1.3	3.0	
Variability in Precipitation	1.4	3.0	
Ocean Acidification	4.0	2.0	
Variability in Ocean Acidification	1.0	2.2	
Currents	2.2	1.0	
Sea Level Rise	2.8	1.5	
Exposure Score	Very High		
Overall Vulnerability Rank	Very High		

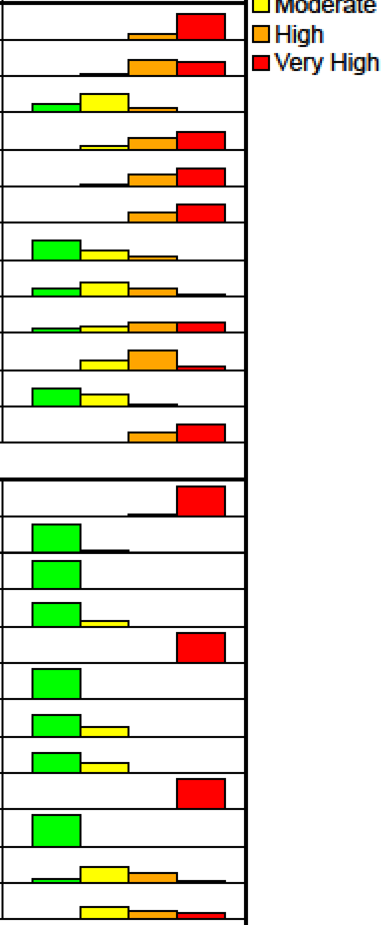

Atlantic Salmon (*Salmo salar*)

Overall Climate Vulnerability Rank: **Very High** (100% certainty from bootstrap analysis).

Climate Exposure: **Very High**. Three exposure factors contributed to this score: Ocean Surface Temperature (4.0), Ocean Acidification (4.0) and Air Temperature (4.0). Atlantic Salmon are anadromous, spawning in freshwater, developing in freshwater and estuarine habitats, feeding as adults in marine habitats.

Biological Sensitivity: **Very High**. Five sensitivity attributes scored above 3.5: Stock Status (3.8), Early Life History Requirements (3.6), Dispersal and Early Life History (3.6), Spawning Cycle (3.5), and Complexity in Reproduction (3.5). Atlantic Salmon are diadromous and iteroparous, but few fish survive to repeat spawn. The Gulf of Maine Distinct Population Segment was listed as endangered under the endangered species act in 2000 (FR, 2000). Adults return to natal rivers and spawn in gravel habitats in the fall. Benthic eggs incubate through the winter and larvae are relatively large and dispersal is limited.

Distributional Vulnerability Rank: **Moderate** (87% certainty from bootstrap analysis). Atlantic Salmon have a relatively high degree of spawning fidelity, which limits the ability of the species to shift distribution (Stabell 1984). However, a low degree of straying has been identified (Martin et al., 2012).

Directional Effect in the Northeast U.S. Shelf: The effect of climate change on Atlantic Salmon in the Northeast U.S. Shelf Ecosystem is very likely to be negative (>95% certainty in expert scores). Warming will change freshwater and marine habitats and potentially effect the phenology of Atlantic Salmon migration. Ocean acidification could also affect olfaction, which Atlantic Salmon use for natal homing.

Data Quality: 88% of the data quality scores were 2 or greater indicate that data quality is moderate.

Climate Effects on Abundance and Distribution: In a review, Jonsson and Josson (2009) concluded that the thermal niche of Atlantic Salmon will likely shift northward causing decreased production and possibly extinction at the southern end of the range. The Northeast U.S. Shelf Ecosystem represents the southern extent of the range of Atlantic Salmon in the Northwest Atlantic Ocean. In a more recent review, Friedland et al. (2014) found that declines in post-smolt survival were associated with ocean warming. Friedland et al. (2014) hypothesized that in the Northwest Atlantic, the decline in survival was a result of early ocean migration by post-smolts. Similarly, Mills et al. (2013) suggested that poor trophic conditions, likely due to climate-driven environmental factors, and warmer ocean temperatures are constraining the productivity and recovery of Atlantic Salmon in the Northwest Atlantic. Thus, there is ample evidence that climate change and long-term climate variability will reduce the productivity of Atlantic Salmon in the Northeast U.S. Shelf Ecosystem.

Life History Synopsis**:** Atlantic Salmon is an anadromous species found in rivers and along the coast of both sides of the north Atlantic. Once common from Long Island Sound to northeastern Labrador in the western Atlantic, the distribution of Atlantic Salmon on both sides of the Atlantic has been greatly reduced due to human activity and habitat degradation (Kocik and Friedland, 2002; Fay et al., 2006). Atlantic Salmon are sexually mature after 1-3 years at sea, but the spawning population is predominantly made up of fish that have experienced 2 winters at sea (Kocik and Friedland, 2002; USASAC, 2004). Starting in spring, Atlantic Salmon return to their natal river; the homing instinct is very strong with >90% site fidelity (NRC, 2003; USASAC, 2004). Spawning occurs from October – November (Fay et al., 2006). Females lay eggs in shallow gravel nests, called redds; bury the eggs under gravel after

fertilization; then return to the ocean or overwinter in the river (Fay et al., 2006). Atlantic Salmon are iteroperous, but very few survive to repeat spawn (Kocik and Friedland, 2002; USASAC, 2004). Eggs incubate in the nest for approximately 6 months before hatching in the spring (Fay et al., 2006). The newly hatched alevin, remain buried in the nest till their yolk is absorbed (3 – 6 weeks), then emerge as independently feeding fry (Fay et al., 2006). Fry prefer shallow, low velocity, gravel substrate and feed opportunistically on zooplankton (Nislow et al., 1999). Once fry develop vertical bars of pigment, they are referred to as parr, which remain in the river for 1-3 years (Kocik and Friedland, 2002; USASAC, 2004). Parr consume invertebrates, such as larval insects and molluscs, and small fish (Scott and Crossman, 1973; Baum, 1997; Nislow et al., 1999). American Eel and Brook Trout are the main predators of young Atlantic Salmon (Fay et al., 2006). Parr mature, changing physiologically, into smolts, which enter salt water in spring and continue to mature, now called postsmolts, for 1-3 years in the ocean before becoming sexually mature (Fay et al., 2006). Oceanic postsmolts are opportunistic, surface feeders consuming invertebrates, insects, amphipods, euphausiids, gammarids, and fishes (Kocik and Friedland, 2002). Opportunistic predators, such as gadids, Silver Hake, and several sea birds, include postsmolt Atlantic Salmon in their diet (Kocik and Friedland, 2002). Atlantic Salmon mature to adulthood in ocean waters north of Newfoundland (Kocik and Friedland, 2002). Fish are the primary prey of adults, including Atlantic Herring, Capelin, small Atlantic Mackerel, Haddock, and some flatfishes (Kocik and Friedland, 2002; Mills et al., 2013). As adults, the only threats come from large fish such as tuna, Swordfish, large sharks, and seals (Kocik and Friedland, 2002). The U.S. Fish and Wildlife Service and National Marine Fisheries Service list several Atlantic Salmon populations as endangered distinct population segments under the federal Endangered Species Act since 2000 (FR, 2000; 2009; NRC, 2004). Farming of Atlantic Salmon has alleviated the fishing pressure on wild Atlantic Salmon populations, but may also have negative effects on the wild population by introducing disease and through genetic interactions (Kocik and Friedland, 2002).

Literature Cited:

Baum ET. Maine Atlantic Salmon: A National Treasure, 1st Edition. Hermon: Atlantic Salmon Unlimited; 1997.

Fay, C.; M. Bartron; S. Craig; A. Hecht; J. Pruden; R. Saunders; T. Sheehan; J. Trial. 2006. Status review for anadromous Atlantic Salmon (Salmo salar) in the United States. Report to the National Marine Fisheries Service and U. S. Fish and Wildlife Service. 294 p. Accessed online (July 2014): http://www.nmfs.noaa.gov/pr/pdfs/statusreviews/atlanticsalmon.pdf

Federal Register. 2000. Endangered and Threatened Species; Final Endangered Status for a Distinct Population Segment of Anadromous Atlantic Salmon (Salmo salar) in the Gulf of Maine Admission of Refugees." 65, 223, 69459-69481. http://www.fisheries.noaa.gov/pr/pdfs/fr/fr65-69459.pdf

Federal Register. 2009. Endangered and Threatened Species; Determination of Endangered Status for the Gulf of Maine Distinct Population Segment of Atlantic Salmon; Final Rule. 74, 117, 29344- 29387. http://www.fisheries.noaa.gov/pr/pdfs/fr/fr74-29344.pdf

Friedland KD, Shank BV, Todd CD, McGinnity P, Nye JA. Differential response of continental stock complexes of Atlantic salmon (Salmo salar) to the Atlantic Multidecadal Oscillation. J Mar Syst. 2014; 133: 77-87. doi: 10.1016/j.jmarsys.2013.03.003

Jonsson B, Jonsson N. A review of the likely effects of climate change on anadromous Atlantic salmon Salmo salar and brown trout Salmo trutta, with particular reference to water temperature and flow. J Fish Biol. 2009; 75(10): 2381-2447. doi: 10.1111/j.1095-8649.2009.02380.x

Kocik JF, Friedland KD. Salmons and Trouts, Family Salmonidae. In: B.B. Collette BB, Klein-MacPhee G, editors, Fishes of the Gulf of Maine, 3rd ed. Washington: Smithsonian Institution Press; 2002. pp. 170-181.

Martin J, Bareille G, Berail S, Pécheyran C, Gueraud F, Lange F, et al. Persistence of a southern Atlantic salmon population: diversity of natal origins from otolith elemental and Sr isotopic signatures. Can J Fish Aquat Sci. 2013; 70(2): 182-197. doi: 10.1139/cjfas-2012-0284

Mills KE, Pershing AJ, Sheehan TF, Mountain D. Climate and ecosystem linkages explain widespread declines in North American Atlantic salmon populations. Glob Chang Biol. 2013; 19(10): 3046-3061. doi: 10.1111/gcb.12298

National Research Council (NRC). Atlantic Salmon in Maine. Washington: National Academy Press; 2004. 304pp. Accessed online (July 2014): http://www.nap.edu/openbook.php?record_id=10892&page=21

Nislow KH, Folt CL, Parrish DL. Favorable foraging locations for young Atlantic salmon: application to habitat and population restoration. Ecol Appl. 1999; 9(3): 1085-1099. doi: 10.1890/1051-0761(1999)009[1085:FFLFYA]2.0.CO;2

Scott, W.B. and E.J. Crossman. Freshwater Fishes of Canada. Bulletin 184. Ottawa: Fisheries Research Board of Canada; 1973.

Stabell OB. Homing and olfaction in salmonids: a critical review with special reference to the Atlantic salmon. Biolog Rev. 1984; 59(3): 333-388. doi: 10.1111/j.1469-185X.1984.tb00709.x

United States Atlantic Salmon Assessment Committee (USASAC). 2004. Annual Report of the U.S. Atlantic Salmon Assessment Committee Report No. 16 – 2003 Activities. Annual Report 2004/16. Woods Hole, MA – February 23-26, 2004. 74pp. and appendices. Available: http://www.nefsc.noaa.gov/USASAC/Reports/USASAC2004-Report%2316-2003-Activities.pdf

Atlantic Saury – *Scomberesox saurus*

Overall Vulnerability Rank = Low

Biological Sensitivity = Low
Climate Exposure = High
Data Quality = 67% of scores ≥ 2

Scomberesox saurus	Expert Scores	Data Quality	Expert Scores Plots (Portion by Category)
Stock Status	1.8	1.2	
Other Stressors	1.4	1.6	
Population Growth Rate	1.3	2.1	
Spawning Cycle	2.1	1.8	
Complexity in Reproduction	1.9	1.4	
Early Life History Requirements	2.3	1.6	
Sensitivity to Ocean Acidification	1.4	2.0	
Prey Specialization	1.2	2.4	
Habitat Specialization	1.1	2.2	
Sensitivity to Temperature	2.0	2.2	
Adult Mobility	1.5	2.2	
Dispersal & Early Life History	1.4	1.6	
Sensitivity Score	Low		
Sea Surface Temperature	4.0	3.0	
Variability in Sea Surface Temperature	1.4	3.0	
Salinity	2.4	3.0	
Variability Salinity	1.2	3.0	
Air Temperature	1.1	3.0	
Variability Air Temperature	1.0	3.0	
Precipitation	1.0	3.0	
Variability in Precipitation	1.1	3.0	
Ocean Acidification	4.0	2.0	
Variability in Ocean Acidification	1.0	2.2	
Currents	2.5	1.0	
Sea Level Rise	1.0	1.5	
Exposure Score	High		
Overall Vulnerability Rank	Low		

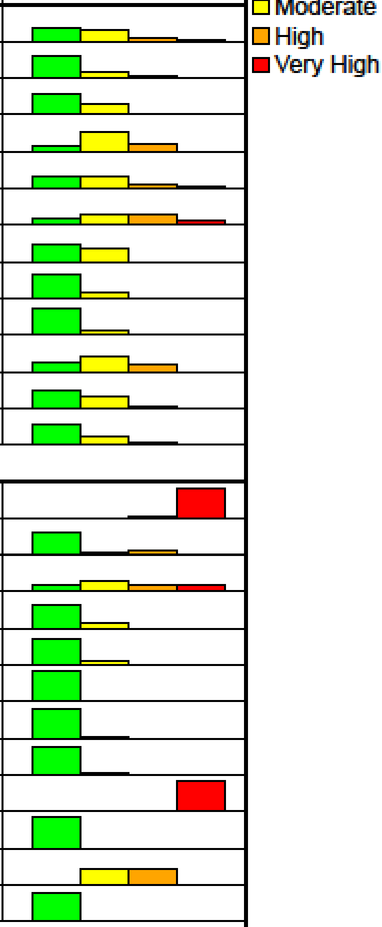

Return to Table of Contents

Atlantic Saury (*Scomberesox saurus*)

Overall Climate Vulnerability Rank: **Low** (100% certainty from bootstrap analysis).

Climate Exposure: **High**. Two exposure factors contributed to this score: Ocean Surface Temperature (4.0) and Ocean Acidification (4.0). Exposure to ocean surface temperature and ocean acidification occurs during all life stages.

Biological Sensitivity: **Low**. All sensitivity scores were below 2.5.

Distributional Vulnerability Rank: **Very High** (40% certainty from bootstrap analysis). Three of the attributes indicated vulnerability to distribution shift. Atlantic Saury are habitat generalists and occur in pelagic habitats both on and off the continental shelf. They are highly mobile and make large seasonal migrations. Spawning occurs in slope waters and larvae have the potential to be broadly dispersed.

Directional Effect in the Northeast U.S. Shelf: The effect of climate change on Atlantic Saury on the Northeast U.S. Shelf is estimated to be positive, but this estimate is highly uncertain (<66% certainty in expert scores). Warming may increase available habitat on the Northeast U.S. Shelf and a northward shift in the Gulf Stream may result in more Atlantic Saury on the shelf. However, the data quality for Atlantic Saury is low reducing confidence in understanding of distribution and abundance.

Data Quality: 67% of the data quality scores were 2 or greater.

Climate Effects on Abundance and Distribution: There is relatively little information available regarding climate effects on Atlantic Saury. The abundance of Pacific Saury (*Cololabis saira*) in the northwestern Pacific is correlated to temperature (Tian et al., 2003). A change in distribution in the East/Japan Sea in the 1970s has been linked to an increase in temperature (Zhang and Gong, 2005). Tseng et al. (2011) projected a poleward shift in Pacific Saury under different future warming scenarios based on a habitat model, and Ito et al. (2013) projected a decrease in growth under different climate scenarios using an ecosystem-based bioenergetic model. If parallels exist for Atlantic Saury, a decrease in productivity and a northward shift in distribution may occur with climate change.

Life History Synopsis**:** Atlantic Saury is an epipelagic, open-ocean, forage fish species found from offshore of Cape Hatteras, North Carolina, to Newfoundland out to 40° W (Collette, 2002). Atlantic Saury mature at age 2, and most spawning results from fish aged 2-3 years (Collette, 2002). Spawning occurs south of the Gulf Stream frontal zone in winter and spring (Collette, 2002). Saury are likely batch spawners (Dudnik et al., 1981). Eggs and larvae are epipelagic in the upper 1 m of the open ocean (Dudnik et al., 1981). Incubation of the eggs takes more than 2 weeks (Collette, 2002). Little is known about the larval stage. Adult Atlantic Saury also occur in the warm surface waters of the open sea, west of the Gulf Stream core, but may go as deep as 50 m during the day (Dudnik et al., 1981). Seasonal along- and cross-shelf migrations take Atlantic Saury from the waters well offshore of Cape Hatteras, North Carolina, in winter to continental shelf waters, such as the Gulf of Maine, Georges Bank, and Scotian Shelf, in summer and fall (Dudnik et al., 1981). Atlantic Saury is a planktivorous species, and feeds mainly on siphonophores, copepods, euphausiids, and amphipods (Collette, 2002). A variety of predators, including squids, Swordfish, marlins, sharks, tunas, hakes, Atlantic Cod, Pollock, dolphins, whales, and birds, feed on the abundant, open-ocean prey species (Collette, 2002). There is no directed fishery for the species in the western Atlantic, but Atlantic Saury is an important food fish in other parts of the world (Collette, 2002).

Return to Table of Contents

Literature Cited:

Collette BB. North Atlantic Saury/ Scomberesox saurus (Walbaum 1792). In: Collete BB, Klein-MacPhee G, editors, Fishes of the Gulf of Maine, 3rd edition. Washington: Smithsonian Institution Press; 2002. pp. 285-287.

Dudnik YI, V K Zilanov VK, Kudrin VD, Nesvetov VA, Nesterov AS. 1981. Distribution and biology of Atlantic saury, Scomberesox saurus (Walbaum), in the northwest Atlantic. NAFO Sci. Coun. Stud. No. 1:23-29. Accessed online (April 2015): http://www.nafo.int/publications/frames/studies.html

Tian Y, Akamine T, Suda M. Variations in the abundance of Pacific saury (Cololabis saira) from the northwestern Pacific in relation to oceanic-climate changes. Fish Res. 2003; 60(2), 439-454. doi: 10.1016/S0165-7836(02)00143-1

Tseng CT, Sun CL, Yeh SZ, Chen SC, Su WC, Liu DC. Influence of climate-driven sea surface temperature increase on potential habitats of the Pacific saury (Cololabis saira). ICES J Mar Sci. 2011; 68(6): 1105-1113. doi: 10.1093/icesjms/fsr070

Zhang CI, Gong Y. Effect of ocean climate changes on the Korean stock of Pacific saury, Cololabis saira (Brevoort). J Oceangr. 2005; 61(2): 313-325. doi: 10.1007/s10872-005-0042-2

Return to Table of Contents

Atlantic Sea Scallop – *Placopecten magellanicus*

Overall Vulnerability Rank = High

Biological Sensitivity = High
Climate Exposure = High
Data Quality = 88% of scores ≥ 2

Placopecten magellanicus	Expert Scores	Data Quality	Expert Scores Plots (Portion by Category)
Stock Status	1.8	3.0	
Other Stressors	1.8	1.7	
Population Growth Rate	2.0	2.8	
Spawning Cycle	2.0	3.0	
Complexity in Reproduction	1.7	3.0	
Early Life History Requirements	2.2	3.0	
Sensitivity to Ocean Acidification	4.0	2.6	
Prey Specialization	1.4	2.8	
Habitat Specialization	1.2	3.0	
Sensitivity to Temperature	2.4	2.8	
Adult Mobility	3.7	3.0	
Dispersal & Early Life History	2.2	2.8	
Sensitivity Score	High		
Sea Surface Temperature	3.9	3.0	
Variability in Sea Surface Temperature	1.0	3.0	
Salinity	1.8	3.0	
Variability Salinity	1.2	3.0	
Air Temperature	1.0	3.0	
Variability Air Temperature	1.0	3.0	
Precipitation	1.0	3.0	
Variability in Precipitation	1.0	3.0	
Ocean Acidification	4.0	2.0	
Variability in Ocean Acidification	1.0	2.2	
Currents	2.1	1.0	
Sea Level Rise	1.1	1.5	
Exposure Score	High		
Overall Vulnerability Rank	High		

Low Moderate High Very High

Return to Table of Contents

Atlantic Sea Scallop (*Placopecten magellanicus*)

Overall Climate Vulnerability Rank: **High** (100% certainty from bootstrap analysis).

Climate Exposure: **High**. Two exposure factors contributed to this score: Ocean Surface Temperature (3.9) and Ocean Acidification (4.0). All life stages of Atlantic Sea Scallop use marine habitats.

Biological Sensitivity: **High**. Two sensitivity attributes scored above 3.0: Sensitivity to Ocean Acidification (4.0) and Adult Mobility (3.7). Atlantic Sea Scallops form calcium carbonate shell and adults are sessile, but capable of small-scale movements (meters).

Distributional Vulnerability Rank: **Moderate** (83% certainty from bootstrap analysis).

Directional Effect in the Northeast U.S. Shelf: The effect of climate change on Atlantic Sea Scallop on the Northeast U.S. Shelf is very likely to be negative (>95% certainty in expert scores). Ocean acidification will likely negatively impact molluscs, including Atlantic Sea Scallop. Warming may also reduce habitat and increase vulnerability to predation which will reduce productivity and cause distributions to shift northwards and into deeper waters.

Data Quality: 88% of the data quality scores were 2 or greater indicate that data quality is moderate.

Climate Effects on Abundance and Distribution: Using a coupled biogeochemical, population, bioeconomic model, Cooley et al. (2015) indicated that yields may decrease in the Atlantic Sea Scallop fishery as adult growth slows under ocean acidification. There are no studies on the effects of ocean acidification on Atlantic Sea Scallops specifically, but work with other molluscs suggest negative effects (Ries et al., 2009; Talmage and Gobler, 2010). Predation of juvenile Atlantic Sea Scallops was higher at higher temperatures (Barbeau and Scheibling, 1994). Recruitment of Atlantic Sea Scallops in shallow water is likely decreased owing to higher temperatures and recruitment in offshore waters is likely decreased because of temperature related overlap with an important predator species *Astropecten americanus*. Increased temperatures may lead to lower recruitment and thus negatively affect population productivity.

Life History Synopsis**:** Atlantic Sea Scallop is a marine bivalve species that occurs from the Gulf of St. Lawrence to Cape Hatteras, North Carolina (Hart and Chute, 2004). Atlantic Sea Scallops are among the most fecund of all bivalves, but while most mature at 2 years, significant egg production does not occur until after 4 years of life (Hart and Chute, 2004). Atlantic Sea Scallops have separate sexes, with occasional instances of hermaphroditism. Spawning occurs between August and November, but there also is some spawning in the spring (Hart and Chute, 2004). Scallop eggs are dense and benthic (Hart and Chute, 2004). There are several stages of larval development. Eggs hatch into a trochophore stage, which is pelagic and only lasts a few days. Trochophore larvae then transform into the veliger stage, which is also pelagic and can last 1-2 months (Posgay, 1953). The pelagic larvae are generally at the mercy of currents, but can make small corrections to their distribution (Hart and Chute, 2004). These early pelagic stages are eaten by filter feeders and planktivores (Hart and Chute, 2004). At a shell height of about 0.25 mm, usually in late fall or early winter, larvae transform to pediveligers, then settle to the benthos, changing their diet, morphology, and locomotory ability (Hart and Chute, 2004). At this stage, young Atlantic Sea Scallops attach to bottom substrate such as shell fragments, gravel, buoys, or filamentous plants and animals (e.g., algae, hydroids) using byssal threads and are referred to as spat (Hart and Chute, 2004). Juvenile Atlantic Sea Scallops are free swimming, but only for short distances

when disturbed (Hart and Chute, 2004). Adult Atlantic Sea Scallops prefer firm sand, gravel, shell, or rock habitat, in cool water, with high salinity, and require a current for feeding and waste removal (Hart and Chute, 2004). Throughout most of their lives, Atlantic Sea Scallops are suspension filter feeders on phytoplankton, microzooplankton, and detritus (Hart and Chute, 2004). There are many fish and invertebrate predators of Atlantic Sea Scallops, namely: Atlantic Cod, Atlantic Wolfish, Ocean Pout, sculpins, American Plaice, Winter Flounder, Yellowtail Flounder, cancer crabs, American Lobsters, and several sea star species (Hart and Chute, 2004). The New England Fishery Management Council's Sea Scallop Management Plan manages Atlantic Sea Scallops, one of the most valuable fisheries in the United States (Hart and Chute, 2004). Based on the most recent stock assessment, Atlantic Sea Scallops are neither overfished nor is overfishing occurring (NEFSC, 2007).

Literature Cited:

Barbeau MA, Scheibling RE. Temperature effects on predation of juvenile sea scallops [*Placopecten magellanicus* (Gmelin)] by sea stars (*Asterias vulgaris* Verrill) and crabs (*Cancer irroratus* Say). J Exper Mar Bio Ecol. 1994; 182(1): 27-47. DOI: 10.1016/0022-0981(94)90209-7

Hart DR. Effects of sea stars and crabs on sea scallop *Placopecten magellanicus* recruitment in the Mid-Atlantic Bight (USA). Mar Ecol Prog Ser. 2006; 306: 209-221. DOI: 10.3354/meps306209

Hart DR, Chute AS. Essential Fish Habitat Source Document: Sea scallop, *Placopecten magellanicus*, life history and habitat characteristics, Second Edition. NOAA Tech. Memo. 2004; NMFS-NE-189: 21 p. Accessed online (August 2014): http://www.nefsc.noaa.gov/nefsc/publications/tm/tm189/tm189.pdf

Northeast Fisheries Science Center (NEFSC). 45th Northeast Regional Stock Assessment Workshop (45th SAW): 45th SAW assessment report. US Dep Commer, Northeast Fish Sci Cent Ref Doc 2007; 07-16: 370 p. Accessed online (August 2015): http://www.nefsc.noaa.gov/publications/crd/crd0716/

Ries JB, Cohen AL, McCorkle DC. Marine calcifiers exhibit mixed responses to CO2-induced ocean acidification. Geol. 2009; 37(12), 1131-1134. doi: 10.1130/G30210A.1

Talmage SC, Gobler CJ. Effects of past, present, and future ocean carbon dioxide concentrations on the growth and survival of larval shellfish. Proc Nat Acad Sci. 2010; 107(40): 17246-17251. DOI: 10.1073/pnas.0913804107

Return to Table of Contents

Atlantic Sturgeon – *Acipenser oxyrinchus*

Overall Vulnerability Rank = Very High

Biological Sensitivity = High
Climate Exposure = Very High
Data Quality = 88% of scores ≥ 2

Acipenser oxyrinchus	Expert Scores	Data Quality	Expert Scores Plots (Portion by Category)
Stock Status	3.5	1.8	
Other Stressors	2.9	2.6	
Population Growth Rate	3.9	2.4	
Spawning Cycle	2.6	2.8	
Complexity in Reproduction	2.8	2.4	
Early Life History Requirements	2.2	2.2	
Sensitivity to Ocean Acidification	1.6	2.0	
Prey Specialization	1.4	3.0	
Habitat Specialization	3.1	3.0	
Sensitivity to Temperature	1.7	2.8	
Adult Mobility	1.2	2.9	
Dispersal & Early Life History	3.0	3.0	
Sensitivity Score	High		
Sea Surface Temperature	4.0	3.0	
Variability in Sea Surface Temperature	1.0	3.0	
Salinity	2.1	3.0	
Variability Salinity	1.2	3.0	
Air Temperature	4.0	3.0	
Variability Air Temperature	1.0	3.0	
Precipitation	1.3	3.0	
Variability in Precipitation	1.4	3.0	
Ocean Acidification	4.0	2.0	
Variability in Ocean Acidification	1.0	2.2	
Currents	2.0	1.0	
Sea Level Rise	2.7	1.5	
Exposure Score	Very High		
Overall Vulnerability Rank	Very High		

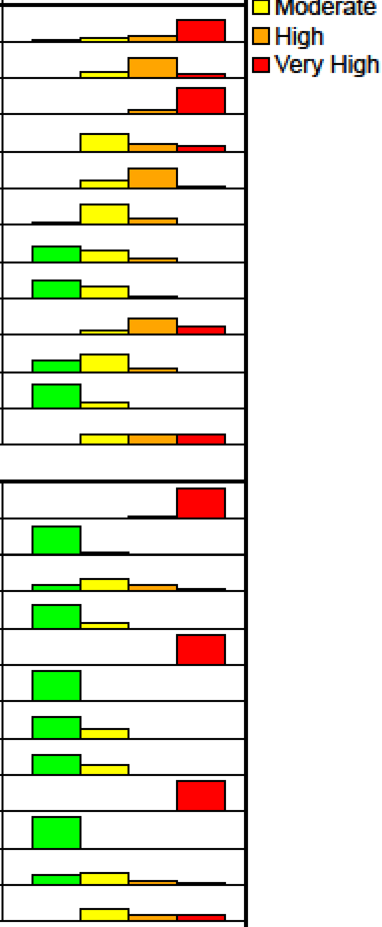

Atlantic Sturgeon (*Acipenser oxyrhynchus*)

Overall Climate Vulnerability Rank: **Very High** (99% certainty from bootstrap analysis).

Climate Exposure: **Very High**. Three exposure factors contributed to this score: Ocean Surface Temperature (4.0), Ocean Acidification (4.0) and Air Temperature (4.0). Atlantic Sturgeon are anadromous, spawning in freshwater, developing in freshwater and estuarine habitats, and feeding as adults in freshwater, estuarine, and marine habitats.

Biological Sensitivity: **High**. Four sensitivity attributes scored above 3.0: Population Growth Rate (3.9), Stock Status (3.5), Habitat Specialization (3.1), and Dispersal and Early Life History (3.0). Shortnose Sturgeon was listed as Endangered under the Endangered Species Act in 2009 (ASSRT, 2010) and are long-lived and slow growing (Musick, 2002). Spawning occurs in specific habitats in the spring and individuals spawn every 1-5 years. Eggs are benthic and relatively large, hatched larvae are relatively well-developed, and larval dispersal is minimal.

Distributional Vulnerability Rank: **Low** (100% certainty from bootstrap analysis). Atlantic Sturgeon are relatively invulnerable to distribution shifts. Spawning occurs in freshwater (Able and Fahay, 2010) and genetic studies indicate a high level of separation between river system (Grunwald et al., 2008). Adults do move into marine habitats (10-50 m) for feeding creating the possibility for movement among river systems (Stein et al., 2004). Climate projections based on a habitat model of a con-specific European Atlantic Sturgeon (*Acipenser sturio).*

Directional Effect in the Northeast U.S. Shelf: The effect of climate change on Atlantic Sturgeon is estimated to be negative, but this estimate has a high degree of uncertainty (<66% certainty in expert scores). Most climate factors have the potential to decrease productivity (sea level rise; reduced dissolved oxygen, increased temperatures). However, understanding the magnitude and interaction of different effects is difficult. The effect of ocean acidification over the next 30 years is likely to be minimal.

Data Quality: 88% of the data quality scores were 2 or greater indicate that data quality is moderate.

Climate Effects on Abundance and Distribution: Numerous studies indicate that Atlantic Sturgeon will be impacted by climate change. Secor and Gunderson (1998) found that juvenile metabolism and survival were impacted by increasing hypoxia in combination with increasing temperature. Niklitschek and Secor (2005) used a multivariable bioenergetics and survival model to generate spatially explicit maps of potential production in the Chesapeake Bay; a 1°C temperature increase reduced productivity by 65% (Niklitschek and Secor, 2005). Habitat models coupled with global climate models for the cogener, European Atlantic Sturgeon (*Acipenser sturio*) indicate strong climate effects throughout the range, especially in the southern portions (Lassalle et al., 2010).

Life History Synopsis**:** Atlantic Sturgeon is a long-lived, anadromous species found from Labrador to northern Florida associated with most large river estuarine systems and surrounding coastal waters (Able and Fahay, 2010). Males reach maturity at 9-24 years, and females mature at 10-30 years, maturing at younger ages in the warm south than the cool north (Musick, 2002; Able and Fahay, 2010). Older, larger females produce substantially more eggs than younger females (Able and Fahay, 2010). Individuals do not spawn every year, with 1-5 years between spawning events (Able and Fahay, 2010). Spawning occurs during winter-spring in the south and spring-summer in northern areas after returning

to natal rivers (Able and Fahay, 2010). Spawning occurs in freshwater with a strong current, at least 3 m deep, and over rubble bottom (Able and Fahay, 2010). Eggs are darkly pigmented, demersal, and adhere to structure and vegetation (Able and Fahay, 2010). Eggs hatch within a week of spawning, and early larvae are large, photonegative, and hide in crevices of nearby structure (Musick, 2002; Able and Fahay, 2010). Larvae rely on yolk for 6-12 days, and remain in freshwater near the spawning site for at least a few months; larvae do not tolerate even low salinities (Able and Fahay, 2010; ASMFC, 2012). At sizes between 31.5 mm and 136 mm, late larvae and early juveniles become photopositive and migrate downstream to nursery areas, slowly becoming more tolerant of saline water (Musick, 2002; Able and Fahay, 2010; ASMFC, 2012). Juveniles remain in freshwater and estuaries for several years before heading to sea (Able and Fahay, 2010; ASMFC, 2012). Once juveniles have left the estuary, they mature to adulthood in coastal waters and may make seasonal along-shelf migrations south in fall and winter, then north in spring and summer (Musick, 2002). Juvenile Atlantic Sturgeon consume aquatic insects, amphipods, isopods, molluscs, polychaete and oligochaete worms in fresh and brackish waters and may cease feeding during summer (Able and Fahay, 2010). Adult Atlantic Sturgeon are found in coastal and estuarine waters during fall and winter (Able and Fahay, 2010). Spawning adults migrate to freshwater in spring when water temperatures rise; however, a small portion of the population may migrate to spawning areas during the previous fall (Able and Fahay, 2010). Males arrive at spawning sites and stay in the river or lower estuary till fall before migrating, but females migrate out of spawning areas after 4-6 weeks (Able and Fahay, 2010). Atlantic Sturgeon are opportunistic benthivores, consuming insect larvae, polychaetes, isopods, decapod crustaceans, amphipods, gastropods, bivalves, and small fishes (Musick, 2002; Able and Fahay, 2010). Little is know about predators of the Atlantic Sturgeon. The Atlantic States Marine Fisheries Commission manages the species and has placed a moratorium on fishing until 2038 (ASMFC, 2006). In 2012 the Gulf of Maine population was listed as a threatened species and the remaining four Atlantic populations were listed as endangered under the Endangered Species Act (NMFS, 2012a, b).

Literature Cited:

Able KW, Fahay MP. 2010. Ecology of estuarine fishes: temperate waters of the western North Atlantic. Baltimore: The Johns Hopkins University Press; 2010. 566p.

Atlantic States Marine Fisheries Commission (ASMFC). 2006. Addendum III to Amendment 1 of the interstate fishery management plan for Atlantic Sturgeon. 10p. Accessed online (August 2015): http://www.asmfc.org/uploads/file/sturgeonAddendumIII.pdf

Atlantic States Marine Fisheries Commission (ASMFC). 2012. Habitat Addendum IV to Amendment 1 of the interstate fishery management plan for Atlantic Sturgeon. 16p. Accessed online (August 2015): http://www.asmfc.org/uploads/file/sturgeonHabitatAddendumIV_Sept2012.pdf

Atlantic Sturgeon Status Review Team (ASSRT) 2007. Status Review of Atlantic sturgeon (Acipenser oxyrinchus oxyrinchus). Report to National Marine Fisheries Service, Northeast Regional Office. February 23, 2007. 174 pp. http://www.nmfs.noaa.gov/pr/pdfs/statusreviews/atlanticsturgeon2007.pdf

Grunwald C, Maceda L, Waldman J, Stabile J, Wirgin I. Conservation of Atlantic sturgeon Acipenser oxyrinchus oxyrinchus: delineation of stock structure and distinct population segments. Conserv Genet. 2008; 9(5): 1111-1124. doi: 10.1007/s10592-007-9420-1

Lassalle G, Crouzet P, Gessner J, Rochard E. Global warming impacts and conservation responses for the critically endangered European Atlantic sturgeon. Biolog Conserv. 2010; 143(11): 2441-2452. Available: http://dx.doi.org/10.1016/j.biocon.2010.06.008

Musick JA. Atlantic Sturgeon/ Acipenser oxyrinchus Mitchill 1815. In: B.B. Collette BB, Klein-MacPhee G, editors, Fishes of the Gulf of Maine, 3rd ed. Washington: Smithsonian Institution Press; 2002. pp. 85-88.

National Marine Fisheries Service (NMFS). 2012. Endangered and Threatened Wildlife and Plants; Threatened and Endangered Status for Distinct Population Segments of Atlantic Sturgeon in the Northeast Region. Federal Register. 77: 5880-5912. Available: http://www.nmfs.noaa.gov/pr/pdfs/fr/fr77-5880.pdf

National Marine Fisheries Service (NMFS). 2012. Endangered and Threatened Wildlife and Plants; Final Listing Determinations for Two Distinct Population Segments of Atlantic Sturgeon (Acipenser oxyrinchus oxyrinchus) in the Southeast. Federal Register 77: 5914-5982. Available: http://www.nmfs.noaa.gov/pr/pdfs/fr/fr77-5914.pdf

Niklitschek EJ, Secor DH. Modeling spatial and temporal variation of suitable nursery habitats for Atlantic sturgeon in the Chesapeake Bay. Estuar Coast Shelf Sci, 2005; 64(1): 135-148. doi: 10.1016/j.ecss.2005.02.012

Secor DH, Gunderson TE. Effects of hypoxia and temperature on survival, growth, and respiration of juvenile Atlantic sturgeon, Acipenser oxyrinchus. Fish Bull. 1998; 96(3): 603-613.

Stein AB, Friedland KD, Sutherland M. Atlantic sturgeon marine distribution and habitat use along the northeastern coast of the United States. Trans Am Fish Soc. 2004; 133(3): 527-537. doi: 10.1577/T02-151.1

Return to Table of Contents

Atlantic Surfclam – *Spisula solidissima*

Overall Vulnerability Rank = High

Biological Sensitivity = High
Climate Exposure = High
Data Quality = 92% of scores ≥ 2

Spisula solidissima	Expert Scores	Data Quality	Expert Scores Plots (Portion by Category)
Stock Status	2.0	3.0	
Other Stressors	2.0	2.4	
Population Growth Rate	2.3	2.2	
Spawning Cycle	2.3	2.8	
Complexity in Reproduction	1.5	2.8	
Early Life History Requirements	1.8	2.2	
Sensitivity to Ocean Acidification	3.7	2.4	
Prey Specialization	1.8	3.0	
Habitat Specialization	1.2	3.0	
Sensitivity to Temperature	1.9	3.0	
Adult Mobility	3.8	3.0	
Dispersal & Early Life History	2.0	2.8	
Sensitivity Score	High		
Sea Surface Temperature	3.9	3.0	
Variability in Sea Surface Temperature	1.0	3.0	
Salinity	2.1	3.0	
Variability Salinity	1.2	3.0	
Air Temperature	1.9	3.0	
Variability Air Temperature	1.0	3.0	
Precipitation	1.1	3.0	
Variability in Precipitation	1.1	3.0	
Ocean Acidification	4.0	2.0	
Variability in Ocean Acidification	1.0	2.2	
Currents	2.1	1.0	
Sea Level Rise	1.1	1.5	
Exposure Score	High		
Overall Vulnerability Rank	High		

Low
Moderate
High
Very High

Return to Table of Contents

Atlantic Surfclam (*Spisula solidissima*)

Overall Climate Vulnerability Rank: **High** (100% certainty from bootstrap analysis).

Climate Exposure: Two exposure factors contributed to this score: Ocean Surface Temperature (3.9) and Ocean Acidification (4.0). All life stages of Atlantic Surfclam use marine habitats.

Biological Sensitivity: **High**. Two sensitivity attributes scored above 3.0: Sensitivity to Ocean Acidification (3.7) and Adult Mobility (3.8). Atlantic Surfclams form calcium carbonate shell and adults are sessile.

Distributional Vulnerability Rank: **High** (67% certainty from bootstrap analysis).

Directional Effect in the Northeast U.S. Shelf: The effect of climate change on Atlantic Surfclam on the Northeast U.S. Shelf is very likely to be negative (>95% certainty in expert scores). Ocean acidification will likely negatively impact molluscs, including Atlantic Surfclam. Warming may also further reduce habitat, which will reduce productivity and cause distributions to shift northwards and into deeper waters.

Data Quality: 92% of the data quality scores were 2 or greater indicate that data quality is moderate.

Climate Effects on Abundance and Distribution: Distribution of Atlantic Surfclams are affected by increasing temperature; mortality is higher at higher temperatures (Naráez et al., 2015) resulting in a shift in the distribution of the population (Weinberg, 2005). A southern congener (Raveneli's Surfclam) has been identified genetically from the Northeast U.S. Shelf suggesting a range expansion in recent decades. Fertilization success was not affected by a wide-range of temperature nor pH conditions (Clotteau and Dubé, 1993). While fertilization may not be affected, embryonic and larval development of molluscs in general are negatively impacted by ocean acidification (Gazeau et al., 2013).

Life History Synopsis**:** Atlantic Surfclam is a marine bivalve species that occurs from the Gulf of St. Lawrence to Cape Hatteras, North Carolina (Cargnelli et al., 1999). Size and age at maturity varies by region and ranges from 3 months after settlement and 5 mm off the coast of New Jersey to 4 years and 80-95 mm off Prince Edward Island, Canada (Cargnelli et al., 1999). Spawning occurs in summer and early fall in warm water, starting earlier inshore than offshore (Cargnelli et al., 1999). Gametes are broadcast into the water column. Surfclam eggs hatch into trochophore larvae within 1-2 days of fertilization (Cargnelli et al., 1999). The shell first appears on veliger larvae in 1-3 days, and after approximately 18 days, larvae develop into the pediveliger stage, which have a foot and can swim and burrow (Cargnelli et al., 1999). Larvae cannot survive high temperature and prefer higher salinities (Cargnelli et al., 1999). Settlement occurs approximately 3-4 weeks after fertilization. Juveniles and adults occur in coastal waters up to 66 m and do not tolerate low DO (Cargnelli et al., 1999). Offshore surfclams grow larger than individuals inshore (Cargnelli et al., 1999). Atlantic Surfclam is susceptible to a variety of parasites and predators. Parasites include *Sphenophyra dosinae*, *Myocheres major*, *Echeneribothrium* spp., *Paranisakiopsis pectinis*, *Urosporidium spisuli*. A number of species prey on recently settled Atlantic Surfclam including naticid snails, sea stars, Lady Crab, Jonah Crab, Horseshoe Crab, Haddock, Atlantic Cod, and Sevenspine Bay Shrimp (Cargnelli et al., 1999). Surfclams are planktivorous siphon feeders (Cargnelli et al., 1999). The Mid-Atlantic Fishery Management Council manages Atlantic Surfclams through the Atlantic Surfclam and Ocean Quahog Fishery Management Plan and the stock is neither overfished nor is overfishing occurring (NEFSC, 2013).

Literature Cited:

Cargnelli LM, Griesbach SJ, Packer DB, Weissberger E. Essential fish habitat source document: Atlantic Surfclam, *Spisula solidissima*, life history and habitat characteristics. NOAA Tech Memo NMFS NE 1999; 142: 13 p. Accessed Online (August 2015): http://www.nefsc.noaa.gov/nefsc/publications/tm/tm142/

Clotteau G, Dubé F. Optimization of fertilization parameters for rearing surf clams (*Spisula solidissima*). Aquaculture. 1993; 114(3): 339-353. DOI: 10.1016/0044-8486(93)90308-L

Gazeau F, Parker LM, Comeau S, Gattuso JP, O'Connor WA, Martin S, et al. Impacts of ocean acidification on marine shelled molluscs. Mar Biol. 2013; 160(8): 2207-2245. DOI: 10.1007/s00227-013-2219-3

Hare MP, Weinberg J, Peterfalvy O, Davidson M. The "southern" surfclam (Spisula solidissima similis) found north of its reported range: a commercially harvested population in Long Island Sound, New York. J Shell Res. 2010; 29(4): 799-807. DOI: http://dx.doi.org/10.2983/035.029.0413

Narváez DA, Munroe DM, Hofmann EE, Klinck JM, Powell EN, Mann R, et al. Long-term dynamics in Atlantic Surfclam (*Spisula solidissima*) populations: The role of bottom water temperature. J Mar Sys. 2015; 141: 136-148. DOI:10.1016/j.jmarsys.2014.08.007

Northeast Fisheries Science Center (NEFSC). 56th Northeast Regional Stock Assessment Workshop (56th SAW) Assessment Report. US Dept Commer, Northeast Fish Sci Cent Ref Doc. 2013; 13-10: 868 p. Accessed Online (August 2015): http://nefsc.noaa.gov/publications/crd/crd1310/

Weinberg JR. Bathymetric shift in the distribution of Atlantic Surfclams: response to warmer ocean temperature. ICES J Mar Sci. 2005; 62(7): 1444-1453. DOI: 10.1016/j.icesjms.2005.04.020

Return to Table of Contents

Atlantic Wolffish – *Anarhichas lupus*

Overall Vulnerability Rank = High

Biological Sensitivity = High
Climate Exposure = High
Data Quality = 83% of scores ≥ 2

Anarhichas lupus	Expert Scores	Data Quality	Expert Scores Plots (Portion by Category)
Stock Status	3.6	1.4	
Other Stressors	1.4	1.8	
Population Growth Rate	3.6	2.6	
Spawning Cycle	3.0	2.2	
Complexity in Reproduction	1.8	2.8	
Early Life History Requirements	1.9	2.1	
Sensitivity to Ocean Acidification	2.5	2.1	
Prey Specialization	1.1	3.0	
Habitat Specialization	1.1	2.8	
Sensitivity to Temperature	2.0	2.8	
Adult Mobility	2.6	2.7	
Dispersal & Early Life History	2.2	2.5	
Sensitivity Score	High		
Sea Surface Temperature	3.9	3.0	
Variability in Sea Surface Temperature	1.0	3.0	
Salinity	1.1	3.0	
Variability Salinity	1.2	3.0	
Air Temperature	1.0	3.0	
Variability Air Temperature	1.0	3.0	
Precipitation	1.0	3.0	
Variability in Precipitation	1.0	3.0	
Ocean Acidification	4.0	2.0	
Variability in Ocean Acidification	1.0	2.2	
Currents	2.1	1.0	
Sea Level Rise	1.1	1.5	
Exposure Score	High		
Overall Vulnerability Rank	High		

Low
Moderate
High
Very High

Atlantic Wolffish (*Anarhichas lupus*)

Overall Climate Vulnerability Rank: **High** (100% certainty from bootstrap analysis).

Climate Exposure: **High**. Two exposure factors contributed to this score: Ocean Surface Temperature (3.9) and Ocean Acidification (4.0). All life stages of Atlantic Wolffish use marine habitats.

Biological Sensitivity: **High**. Three sensitivity attributes scored above 3.0: Stock Status (3.6), Population Growth Rate (3.6), and Spawning Cycle (3.0). Atlantic Wolffish is on the NMFS Species of Concern List, but a petition to list Atlantic Wolffish under the Endangered Species Act was denied (Atlantic Wolffish Biological Review Team, 2009; NMFS, 2009). Atlantic Wolffish are late maturing (5-6 years) and slow growing (Atlantic Wolffish Biological Review Team, 2009). Male Atlantic Wolffish provide parental care for eggs during the spring reproduction season (Keats et al. 1985) and thus, reproductive output is relatively low and the spawning occurs in a particular season.

Distributional Vulnerability Rank: **Moderate** (74% certainty from bootstrap analysis). Atlantic Wolffish are relatively sedentary and prefer complex habitats. In addition, dispersal of early life stages is limited.

Directional Effect in the Northeast U.S. Shelf: The effect of climate change on Atlantic Wolffish on the Northeast U.S. Shelf is very likely to be negative (>95% certainty in expert scores). Atlantic Wolffish is a cold-temperate species and warming could limit habitat; shifts into deeper water have been observed. Ocean acidification may also negatively affect molluscan prey and thereby indirectly affect Atlantic Wolffish.

Data Quality: 83% of the data quality scores were 2 or greater indicate that data quality is moderate.

Climate Effects on Abundance and Distribution: Atlantic Wolffish is adapted for cold-water environments, producing antifreeze proteins (Desjardins et al., 2012). The optimal temperature for juvenile growth is 9-11°C (McCarthy et al., 1999). The effect of warming on population productivity has not been explicitly addressed, but given the temperature preferences for the species, it is likely that productivity will decrease in the Northeast U.S. Shelf Ecosystem. In addition, distributions are deepening both in the Northeast U.S. Shelf and the North Sea (Dulvy et al., 2008; Nye et al., 2009).

Life History Synopsis**:** Atlantic Wolffish is a benthic marine species found along both coasts of the northern North Atlantic, but particularly from Davis Strait to southern New England in the western North Atlantic (Rountree, 2002). Atlantic Wolffish reach maturity in 5-6 years (Atlantic Wolffish Biological Review Team, 2009). Adults make spawning migrations to colder waters and rocky substrate (Rountree, 2002; Atlantic Wolffish Biological Review Team, 2009). Males and females form bonded pairs during spring or summer, and mate, through internal fertilization, during summer and autumn (Rountree, 2002; Atlantic Wolffish Biological Review Team, 2009). Eggs are hidden in nests under rocks and boulders and guarded by males for several months (Rountree, 2002; Atlantic Wolffish Biological Review Team, 2009). Eggs remain benthic and hatch after 3-9 months partially influenced by water temperature (Rountree, 2002; Atlantic Wolffish Biological Review Team, 2009). Early larvae remain on the bottom near the hatch site from several hours to up to a week; then enter the water column and remain pelagic from 2 weeks to up to 2 months, in colder climates, before settling to the benthos (Rountree, 2002; Atlantic Wolffish Biological Review Team, 2009). While pelagic, larvae consume other fish larvae and small crustaceans such as amphipods and decapod larvae (Rountree, 2002). Juveniles can be found throughout a range of substrates and depths on the continental shelf and slope, but show

a slight preference for the shelter of rocks, large stones and sandy feeding habitats (Rountree, 2002; Atlantic Wolffish Biological Review Team, 2009). Juvenile diet includes echinoderms and molluscs (Rountree, 2002; Atlantic Wolffish Biological Review Team, 2009). Spiny Dogfish, Sea Raven, and Atlantic Cod are the primary predators of juveniles (Rountree, 2002; Atlantic Wolffish Biological Review Team, 2009). Adult Atlantic Wolfish make seasonal migrations from shallow to deep waters in the autumn and deep to shallow in spring (Rountree, 2002; Atlantic Wolffish Biological Review Team, 2009). Adults use their formidable teeth to consume hard-shelled invertebrates such as molluscs, crustaceans, echinoderms, and occasionally fish (Rountree, 2002; Atlantic Wolffish Biological Review Team, 2009). Adult wolffish act as apex predators in some habitats, but have been collected from the stomachs of Greenland Shark, Atlantic Cod, Haddock, gray seal, and Sea Raven (Rountree, 2002; Atlantic Wolffish Biological Review Team, 2009). Atlantic Wolffish are rarely collected and the population is believed to be in decline; however a review committee determined listing the species as Endangered or Threatened was unwarranted. The western Atlantic population of Atlantic Wolffish remains on the Species of Concern List (Atlantic Wolffish Biological Review Team, 2009; NMFS, 2009).

Literature Cited:

Atlantic Wolffish Biological Review Team. 2009. Status review of the Atlantic wolffish (Anarhichas lupus). Report to National Marine Fisheries Service, Northeast Regional Office. September 30, 2009. 149 pp. Accessed online (September 2014): http://www.nmfs.noaa.gov/pr/species/Status%20Reviews/atlantic_wolffish_sr_2009.pdf

Desjardins M, Graham LA, Davies PL, Fletcher GL. Antifreeze protein gene amplification facilitated niche exploitation and speciation in wolffish. FEBS Journal, 2012; 279(12): 2215-2230. DOI: 10.1111/j.1742-4658.2012.08605.x

Dulvy NK, Rogers SI, Jennings S, Stelzenmüller V, Dye SR, Skjoldal HR. Climate change and deepening of the North Sea fish assemblage: a biotic indicator of warming seas. J Appl Ecol. 2008; 45(4): 1029-1039. DOI: 10.1111/j.1365-2664.2008.01488.x

Keats DW, South GR, Steele DH. Reproduction and egg guarding by Atlantic wolffish (*Anarhichas lupus*: Anarhichidae) and ocean pout (*Macrozoarces americanus*: Zoarcidae) in Newfoundland waters. Can J Zoo. 1985; 63(11): 2565-2568. DOI: 10.1139/z85-382

McCarthy ID, Moksness E, Pavlov DA, Houlihan DF. Effects of water temperature on protein synthesis and protein growth in juvenile Atlantic wolffish (*Anarhichas lupus*). Can J Fish Aquat Sci. 1999; 56(2): 231-241. DOI: 10.1139/f98-171

National Marine Fisheries Service (NMFS). Endangered and Treatened Wildlife and Plants; Endangered Species Act Listing Determination for Atlantic Wolffish. Federal Register. 2009; 74(214): 57436-57446. Accessed online (September 2014): http://www.nmfs.noaa.gov/pr/species/fish/atlanticwolffish.htm

Nye JA, Link JS, Hare JA, Overholtz WJ. Changing spatial distribution of fish stocks in relation to climate and population size on the Northeast United States continental shelf. Mar Ecol Prog Ser. 2009; 393: 111-129. DOI: 0.3354/meps08220

Return to Table of Contents

Rountree RA. Atlantic Wolffish/ *Anarhichas lupus* Linnaeus 1758. Pages 485-494. In: BB Collette, G Klein-MacPhee (editors), Fishes of the Gulf of Maine, 3rd edition. Smithsonian Institution Press, Washington D.C. 2002; 882 p.

Barndoor Skate – *Dipturus laevis*

Overall Vulnerability Rank = Moderate

Biological Sensitivity = Moderate
Climate Exposure = High
Data Quality = 83% of scores ≥ 2

Dipturus laevis	Expert Scores	Data Quality	Expert Scores Plots (Portion by Category)
Stock Status	2.8	2.4	
Other Stressors	1.4	1.4	
Population Growth Rate	3.0	2.8	
Spawning Cycle	2.2	0.4	
Complexity in Reproduction	1.3	2.2	
Early Life History Requirements	1.0	3.0	
Sensitivity to Ocean Acidification	1.4	2.8	
Prey Specialization	1.2	3.0	
Habitat Specialization	1.2	3.0	
Sensitivity to Temperature	2.2	3.0	
Adult Mobility	1.9	2.4	
Dispersal & Early Life History	1.7	2.8	
Sensitivity Score	Moderate		
Sea Surface Temperature	3.9	3.0	
Variability in Sea Surface Temperature	1.0	3.0	
Salinity	1.6	3.0	
Variability Salinity	1.2	3.0	
Air Temperature	1.0	3.0	
Variability Air Temperature	1.0	3.0	
Precipitation	1.0	3.0	
Variability in Precipitation	1.0	3.0	
Ocean Acidification	4.0	2.0	
Variability in Ocean Acidification	1.0	2.2	
Currents	2.1	1.0	
Sea Level Rise	1.1	1.5	
Exposure Score	High		
Overall Vulnerability Rank	Moderate		

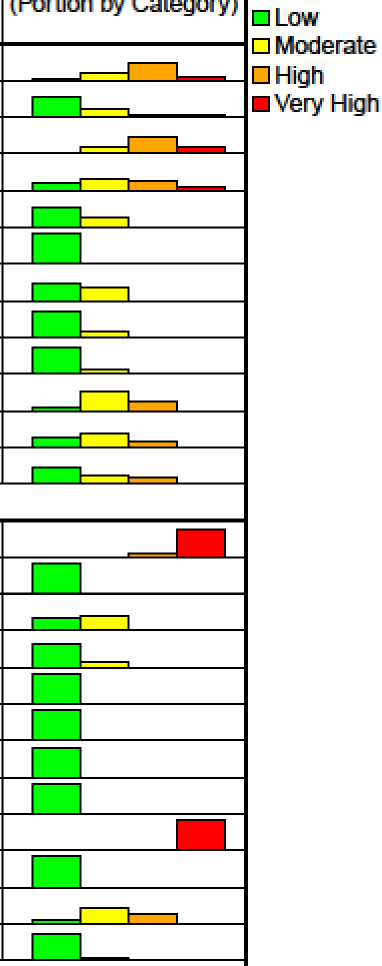

Barndoor Skate (*Dipturus laevis*)

Overall Climate Vulnerability Rank: **Moderate** (94% certainty from bootstrap analysis).

Climate Exposure: **High**. Two exposure factors contributed to this score: Ocean Surface Temperature (3.9) and Ocean Acidification (4.0). Barndoor Skate are demersal and complete their life cycle in marine habitats.

Biological Sensitivity: **Moderate**. Two attributes scored above 2.5: Population Growth Rate (3.0) and Stock Status (2.8). In general, skates have a low population growth rate (higher sensitivity to climate change) (Frisk, 2010). Barndoor Skate is below the biomass target, but above the biomass threshold and abundances have increased since 2000 (NEFSC, 2013).

Distributional Vulnerability Rank: **High** (100% certainty from bootstrap analysis). Barndoor Skates are habitat generalists and mobile as adults, making seasonal movements. In addition, skate egg cases are subject to movement by currents and juveniles may move on scales of 1-10 km.

Directional Effect in the Northeast U.S. Shelf: The effect of climate change on Barndoor Skate is estimated to be negative, but this estimate has a high degree of uncertainty (<66% certainty in expert scores). Barndoor Skate are a cold water species and reductions in productivity may occur because of warming and ocean acidification. However, distributions have not shifted in recent years, contrary to the expectation of a northward shift with warming.

Data Quality: 83% of the data quality scores were 2 or greater indicate that data quality is moderate.

Climate Effects on Abundance and Distribution: Little specific information exists on the effect of climate on Barndoor Skate. Di Santo (2015) found that increased warming and acidification reduce body condition of newly hatched Little Skate. These reductions in size could result in reduced juvenile survival and thus recruitment if similar affects occur in Barndoor Skate. In regional studies of distribution, Barndoor Skate was not included (Murawski, 1993; Nye et al., 2009) but examination of NEFSC trawl survey suggests no change in the center of the distribution over the last 30 years (http://oceanadapt.rutgers.edu/, website last checked 13 June 2015). Working in the Northeast Atlantic, Jones et al. (2013) found that the habitat distribution of *Dipturus batis* may change substantially and an important question identified was the ability of *D. batis* to disperse to the new habitat areas.

Life History Synopsis**:** Barndoor Skate is a large marine skate species found from Newfoundland to North Carolina (Packer et al., 2003). Individuals of the species reach maturity after 8-11 years and are estimated to produce approximately 47 eggs per year (McEachran, 2002; Packer et al., 2003). Spawning is believed to occur in winter, but the actual spawning season may be longer (McEachran, 2002; Packer et al., 2003). Eggs are encapsulated in a horned pouch known as a mermaid's purse and likely hatch in spring or early summer (Packer et al., 2003). Young Barndoor Skate are rarely collected, but are thought to be 180-190 mm total length at hatching (Packer et al., 2003). Adults and juveniles migrate in along- and cross-shelf directions, and are generally farther offshore during winter and are occasionally in high abundances around Georges Bank and the Gulf of Maine (Packer et al., 2003). Barndoor Skate are often associated with Little Skate and Winter Skate in soft muddy, sandy, and gravelly substrate in a range of temperatures and depths (McEachran, 2002; Packer et al., 2003). Barndoor Skate may occur in very deep offshore waters and in estuaries, but are most often collected at depths <150 m in high salinity water (McEachran, 2002). Smaller individuals consume benthic invertebrates such as polychaetes,

copepods, amphipods, isopods, Crangon shrimp, and euphausiids (McEachran, 2002). As the Barndoor Skates increase in size, they include more active prey in the diet, including polychaetes, gastropods, bivalve molluscs, squids, crustaceans, hydroids, and fishes (Spiny Dogfish, Alewife, Atlantic Herring, Atlantic Menhaden, hakes, sculpins, Cunner, Tautog, Sand Lance, Butterfish, and various flounders; McEachran, 2002; Packer et al., 2003). Little is known about predators of the species, but Barndoor Skate are likely consumed by large sharks and maybe whales (McEachran, 2003). Barndoor Skate is managed within a complex with six other species of skate, by the New England Fishery Management Council and are neither overfished nor is overfishing occurring (NEFSC, 2007).

Literature Cited:

Di Santo V. Ocean acidification exacerbates the impacts of global warming on embryonic little skate, *Leucoraja erinacea* (Mitchill). J Exp Mar Bio Ecol. 2015; 463: 72-78. doi:10.1016/j.jembe.2014.11.006

Frisk MG. Life history strategies of batoids. In: Carrier, JC, Musick, JA, Heithaus, MR, editors, Sharks and Their Relatives II: Biodiversity, Adaptive Physiology, and Conservation. Boca Raton: CRC Press; 2010. 283-318.

Jones MC, Dye SR, Fernandes JA, Frölicher TL, Pinnegar JK, Warren R, et al. Predicting the impact of climate change on threatened species in UK waters. PLOS ONE, 2013; 8(1): e54216. doi: 10.1371/journal.pone.0054216

McEachran JD. Barndoor Skate/ Dipturus laevis (Mitchill 1818). In: B.B. Collette BB, Klein-MacPhee G, editors, Fishes of the Gulf of Maine, 3rd ed. Washington: Smithsonian Institution Press; 2002. p. 65-67.

Murawski SA. Climate change and marine fish distributions: forecasting from historical analogy. Trans Am Fish Soc. 1993; 122(5): 647-658. doi: 10.1577/1548-8659(1993)122<0647:CCAMFD>2.3.CO;2

Northeast Fisheries Science Center (NEFSC). 2007. 44th Northeast Regional Stock Assessment Workshop (44th SAW): 44th SAW assessment report. US Dep Commer, Northeast Fish Sci Cent Ref Doc 07-10; 661 p. Accessed online (August 2015): http://www.nefsc.noaa.gov/publications/crd/crd0710/

Northeast Fisheries Science Center (NEFSC). 2013. Update of Skate Stock Status Based on NEFSC Bottom Trawl Survey Data through Autumn 2012 and Spring 2013. Available: http://www.nefsc.noaa.gov/program_review/background2014/TOR3TercerioSkate%20StocksStatusUpdate%202013.pdf

Nye JA, Link JS, Hare JA, Overholtz WJ. Changing spatial distribution of fish stocks in relation to climate and population size on the Northeast United States continental shelf. Mar Ecol Prog Ser. 2009; 393: 111-129. doi: 10.3354/meps08220

Packer DB, Zetlin CA, Vitaliano JJ. 2003. Essential fish habitat source document: Barndoor skate, Dipturus laevis, life history and habitat characteristics. NOAA Tech Memo NMFS NE 173; 23 p. Accessed online (August 2015): http://www.nefsc.noaa.gov/nefsc/publications/tm/tm173/

Return to Table of Contents

Bay Scallop – *Argopecten irradians*

Overall Vulnerability Rank = Very High

Biological Sensitivity = Very High
Climate Exposure = Very High
Data Quality = 88% of scores ≥ 2

Argopecten irradians	Expert Scores	Data Quality	Expert Scores Plots (Portion by Category)
Stock Status	2.6	1.6	
Other Stressors	3.6	3.0	
Population Growth Rate	1.5	2.0	
Spawning Cycle	2.8	3.0	
Complexity in Reproduction	2.4	2.8	
Early Life History Requirements	3.7	3.0	
Sensitivity to Ocean Acidification	3.9	2.2	
Prey Specialization	1.3	2.6	
Habitat Specialization	3.4	3.0	
Sensitivity to Temperature	2.4	3.0	
Adult Mobility	3.9	2.8	
Dispersal & Early Life History	2.6	2.8	
Sensitivity Score	Very High		
Sea Surface Temperature	3.9	3.0	
Variability in Sea Surface Temperature	1.0	3.0	
Salinity	2.0	3.0	
Variability Salinity	1.2	3.0	
Air Temperature	3.9	3.0	
Variability Air Temperature	1.0	3.0	
Precipitation	1.3	3.0	
Variability in Precipitation	1.4	3.0	
Ocean Acidification	4.0	2.0	
Variability in Ocean Acidification	1.0	2.2	
Currents	2.0	1.0	
Sea Level Rise	3.9	1.5	
Exposure Score	Very High		
Overall Vulnerability Rank	Very High		

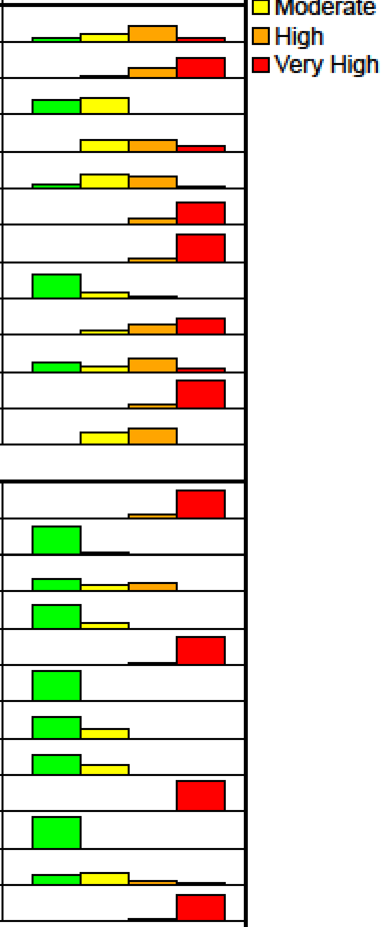

Return to Table of Contents

Bay Scallop (*Argopecten irradians*)

Overall Climate Vulnerability Rank: **Very High** (100% certainty from bootstrap analysis).

Climate Exposure: **Very High**. Three exposure factors contributed to this score: Ocean Surface Temperature (3.9), Air Temperature (3.9), Sea-Level Rise, and Ocean Acidification (4.0). Bay Scallops used both marine and estuarine habitats through their life cycle.

Biological Sensitivity: **Very High**. Four sensitivity attributes scored above 3.5: Other Stressors (3.6), Early Life History Requirements (3.7), Sensitivity to Ocean Acidification (3.9), and Adult Mobility (3.9). Bay are found in estuarine waters and are exposed other stressors including habitat loss and contaminants. Spawning occurs in the winter and larvae settle to eel grass and other biological structure. Adults have a calcium carbonate shell and very limited mobility.

Distributional Vulnerability Rank: **Low** (97% certainty from bootstrap analysis).

Directional Effect in the Northeast U.S. Shelf: The effect of climate change on Bay Scallop on the Northeast U.S. Shelf is very likely to be negative (>95% certainty in expert scores). Ocean acidification will likely negatively impact molluscs, including Bay Scallop. Warming may reduce habitat and increase vulnerability to predation which will reduce productivity. Sea-level rise also has the potential to negatively impact coastal habitats where Bay Scallop live.

Data Quality: 88% of the data quality scores were 2 or greater indicate that data quality is moderate.

Climate Effects on Abundance and Distribution: Shell growth in larval and juvenile Bay Scallops is reduced under ocean acidification (Ries et al., 2009; White et al., 2013; Talmage and Gobler, 2010). Increases in temperature and CO_2 resulted in decreased survival, development, growth, and lipid synthesis (Talmage and Gobler, 2011). These studies indicate that population productivity will likely decrease under climate change. Bay Scallop fisheries are already at very low levels as a result of fishing and harmful algal blooms (MacKenzie, 2008); climate change will add to these negative pressures.

Life History Synopsis**:** Bay Scallop is a short-lived, estuarine bivalve species that occurs from the north shore of Cape Cod, Massachusetts to Laguna Madre, Texas, as three subspecies: *Argopecten irradians irradians* (Lamark 1819) ranging from Cape Cod, Massachusetts, to New Jersey; *A. irradians concentricus* (Say 1882) ranging from New Jersey to Chandeleur Islands in the Gulf of Mexico, and *A. irradians amplicostatus* (Dall 1898) ranging from Galveston, Texas, to Laguna Madre, Texas (NCDMF, 2015). Bay Scallop mature during their first year, but because they only live 1-2 years and most die during their second winter, generally only spawn once (NCDMF, 2015). Spawning season varies with latitude, but ranges from spring to fall (Fay et al., 1983). Spawning occurs later in the south, where spawning is cued to falling temperatures, and earlier in the north, where spawning is cued to rising temperatures (Fay et al., 1983). These broadcast spawning hermaphrodites release only the male or female gametes into the water column at a time to prevent self-fertilization (NCDMF, 2015). Bay Scallop develop through several larval stages before transforming into juveniles. The short-lived trocophore larvae stage quickly develops into the veliger stage within ~2 days post fertilization (NCDMF, 2015). Over the next 10 days, the organs, gills, foot, and first shell develop, and this pediveliger larvae then alternates between swimming and resting on the bottom (NCDMF, 2015). Larval Bay Scallop settle after 2-3 weeks, attaching to submerged aquatic vegetation and other suspended substrate (e.g., rope, oyster shell, filamentous algae) with byssal threads secreted from the foot (Fay et al., 1983; NCDMF, 2015). Survival

is adversely affected by settlement directly to soft sediments, but once the early juveniles reach 20-30 mm they drop to the sediment, preferably in an area of slow currents (NCDMF, 2015). Once settled, juveniles and adults do not travel far, but while still able to make byssal threads, rarely attach to substrate, and often swim away from unfavorable conditions by pulsing water through the mantle cavity (Fay et al., 1983; NCDMF, 2015). Adults are almost exclusively estuarine in shallow flats of mud, hard sand, and submerged aquatic vegetation with slow currents (Fay et al., 1983; NCDMF, 2015). Bay Scallops can tolerate low salinity water for short time periods and cool water during winter, but prefer higher salinities and require warm water for growth (Fay et al., 1983; NCDMF, 2015). Bay Scallops are filter feeders, consuming benthic diatoms as well as planktonic algae and bacteria (Fay et al., 1983; NCDMF, 2015). Pea crabs are a common parasite, and gulls, Blue and Green Crabs, Knobbed Whelks, starfish, and Cownose Rays are common predators (Fay et al., 1983; NCDMF, 2015). Bay Scallop are managed state-by-state. Red tides, high fishing pressure, and increased rates of predation have led to massive population declines and slow recovery (NCDMF, 2015).

Literature Cited:

Fay CW, Neves RJ, Pardue GB. Species profiles: life histories and environmental requirements of coastal fishes and invertebrates (Mid-Atlantic) – bay scallop. U.S. Fish and Wildlife Service, Division of Biological Services, 1983. FWS/OBS-82/11.12. U.S. Army Corps of Engineers, TR EL-82-4. 17 pp. Accessed online (August 2015): http://www.nwrc.usgs.gov/publications/specprof.htm

MacKenzie Jr CL. The bay scallop, *Argopecten irradians*, Massachusetts through North Carolina: its biology and the history of its habitats and fisheries. Mar Fisher Rev. 2008; 70(3-4): 5-79. Accessed Online (August 2015): http://spo.nmfs.noaa.gov/mfr703-4/mfr703-42.pdf

North Carolina Division of Marine Fisheries (NCDMF). North Carolina Bay Scallop Fishery Management Plan Amendment 2. 2015. Accessed Online (May 2015): http://portal.ncdenr.org/c/document_library/get_file?uuid=1f18562d-281f-45c5-928c-f81a6301648c&groupId=38337

Ries JB, Cohen AL, McCorkle DC. Marine calcifiers exhibit mixed responses to CO2-induced ocean acidification. Geol. 2009; 37(12), 1131-1134. doi: 10.1130/G30210A.1

Talmage SC, Gobler CJ. Effects of past, present, and future ocean carbon dioxide concentrations on the growth and survival of larval shellfish. Proc Nat Acad Sci. 2010; 107(40): 17246-17251. DOI: 10.1073/pnas.0913804107

Talmage SC, Gobler CJ. Effects of elevated temperature and carbon dioxide on the growth and survival of larvae and juveniles of three species of Northwest Atlantic bivalves. PLOS ONE, 2011; 6(10): e26941. DOI: 10.1371/journal.pone.0026941

White MM, McCorkle DC, Mullineaux LS, Cohen AL. Early exposure of bay scallops (*Argopecten irradians*) to high CO2 causes a decrease in larval shell growth. PLOS ONE, 2013; 8(4): e61065. DOI: 10.1371/journal.pone.0061065

Return to Table of Contents

Black Sea Bass – *Centropristis striata*

Overall Vulnerability Rank = High

Biological Sensitivity = Moderate
Climate Exposure = Very High
Data Quality = 92% of scores ≥ 2

Centropristis striata	Expert Scores	Data Quality	Expert Scores Plots (Portion by Category)
Stock Status	2.0	3.0	
Other Stressors	1.6	2.2	
Population Growth Rate	1.6	2.6	
Spawning Cycle	2.0	3.0	
Complexity in Reproduction	2.7	2.8	
Early Life History Requirements	2.6	2.2	
Sensitivity to Ocean Acidification	1.8	2.9	
Prey Specialization	1.2	3.0	
Habitat Specialization	2.3	3.0	
Sensitivity to Temperature	1.4	3.0	
Adult Mobility	1.8	2.7	
Dispersal & Early Life History	1.8	2.5	
Sensitivity Score	Moderate		
Sea Surface Temperature	4.0	3.0	
Variability in Sea Surface Temperature	1.0	3.0	
Salinity	2.7	3.0	
Variability Salinity	1.2	3.0	
Air Temperature	4.0	3.0	
Variability Air Temperature	1.0	3.0	
Precipitation	1.2	3.0	
Variability in Precipitation	1.3	3.0	
Ocean Acidification	4.0	2.0	
Variability in Ocean Acidification	1.0	2.2	
Currents	2.0	1.0	
Sea Level Rise	1.6	1.5	
Exposure Score	Very High		
Overall Vulnerability Rank	High		

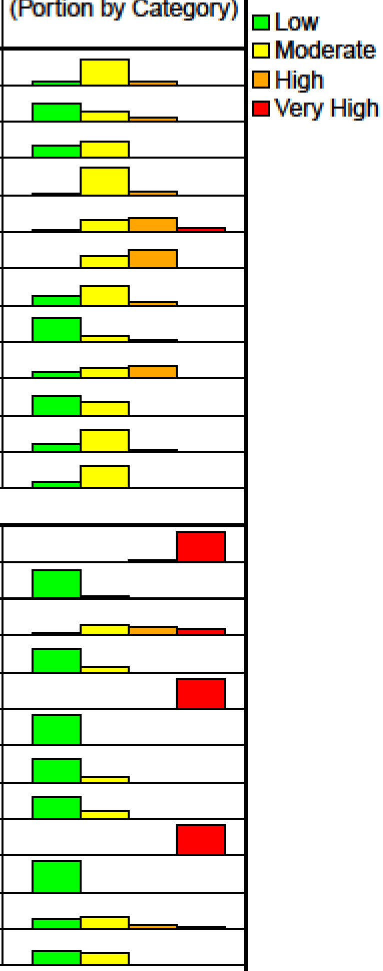

Black Sea Bass (*Centropristis striata*)

Overall Climate Vulnerability Rank: **High** (77% certainty from bootstrap analysis).

Climate Exposure: **Very High**. Three exposure factors contributed to this score: Ocean Surface Temperature (4.0), Ocean Acidification (4.0) and Air Temperature (4.0). Exposure to all three factors occur during all life stages. Black Sea Bass occur in coastal areas during warm months and migrate offshore in cold months and thus are exposed to changes occurring both in offshore and inshore waters.

Biological Sensitivity: **Moderate**. Two sensitivity attributes scored above 2.5: Complexity in Reproduction (2.7) and Early Life History Requirements (2.6). Black Sea Bass are protogynous hermaphrodites and males are territorial (Lavenda, 1949). Eggs and larvae are pelagic and juveniles settle to nearshore and estuaries areas (Able et al., 2006).

Distributional Vulnerability Rank: **High** (98% certainty from bootstrap analysis). Two of the attributes indicated vulnerability to distribution shift. Black Sea Bass are mobile and make inshore-offshore migrations. Eggs and larvae are pelagic and are likely broadly dispersed.

Directional Effect in the Northeast U.S. Shelf: The effect of climate change on Black Sea Bass on the Northeast U.S. Shelf is very likely to be positive (>95% certainty in expert scores). Recruitment will likely increase as temperatures warm and more spawning occurs in the region. Adult distribution will likely extend northwards and the species may move into the Gulf of Maine permanently. The effect of ocean acidification over the next 30 years is likely to be minimal.

Data Quality: 92% of the data quality scores were 2 or greater.

Climate Effects on Abundance and Distribution: Few studies examined the effect of climate factors on the population productivity of Black Sea Bass. Changes in distribution, however, have been linked to warming in the Northeast U.S. Shelf. Howell and Auster (2012) included Black Sea Bass in a warm-adapted assemblages and found increases in abundance in Long Island Sound over the past several decades. Bell et al. (2014) documented northward shift in spring distributions of adults on the Northeast U.S. Shelf and linked this shift to warming temperature.

Life History Synopsis**:** Black Sea Bass is a warm temperate marine species that occurs from the Gulf of Maine to southern Florida and into the Gulf of Mexico (Drohan et al., 2007; Able and Fahay, 2010). The population is managed as three stocks: a Mid-Atlantic stock (north of Cape Hatteras), a South Atlantic Bight stock (south of Cape Hatteras to Florida), and a Gulf of Mexico stock (Drohan et al., 2007; Able and Fahay, 2010). Individuals reach sexual maturity by 2-3 years, but Black Sea Bass are protogynous hermaphrodites, so most individuals <4 years are female and most individuals >5 years are males (Drohan et al., 2007). Black Sea Bass spawn in nearshore areas of the continental shelf beginning in December in the Gulf of Mexico and April through October in the Mid-Atlantic region (Drohan et al., 2007). Eggs are pelagic with temperature-dependent incubation durations ranging from a couple days to a week (Drohan et al., 2007). Larvae are also pelagic, consuming microalgae and zooplankton while being consumed by gelatinous zooplankton (Drohan et al., 2007). The pelagic stage lasts 2-4 weeks before settlement to shell beds or substrate with shell microstructure (Drohan et al., 2007). There is some evidence that settlement can be delayed, but juveniles usually settle in spring and summer to both near-shore shelf and estuarine habitats (Drohan et al., 2007; Able and Fahay, 2010). Juveniles migrate offshore in autumn to overwinter in deeper waters, and then move back inshore in spring (Able and

Fahay, 2010). The primary prey of juveniles is decapods, benthic and epibenthic crustaceans, and small fish (Drohan et al., 2007). Adult Black Sea Bass associate with structurally complex habitat (Drohan et al., 2007). The northern stock migrates offshore and south in the autumn to overwinter as far south as the outer shelf off the coast of Virginia (Drohan et al., 2007). The southern stocks show more site fidelity, but occasionally travel great distances (Drohan et al., 2007). Adult Black Sea Bass are generalist carnivores that feed on infaunal and epibenthic invertebrates such as crustaceans, squid, and small fish (Drohan et al., 2007). The Atlantic States Marine Fisheries Commission and the Mid-Atlantic Fishery Management Council jointly manage the Mid-Atlantic stock through Amendment 13 to the Summer Flounder, Scup, and Black Sea Bass Fishery Management Plan (ASMFC, 2014). The stock is not overfished or experiencing overfishing (NEFSC, 2012).

Literature Cited:

Able KW, Fahay MP. 2010. Ecology of estuarine fishes: temperate waters of the western North Atlantic. Baltimore: The Johns Hopkins University Press; 2010. 566p.

Able KW, Fahay MP, Witting DA, McBride RS, Hagan SM. Fish settlement in the ocean vs. estuary: Comparison of pelagic larval and settled juvenile composition and abundance from southern New Jersey, USA. Estuar Coast Shelf Sci. 2006; 66(1): 280-290. doi:10.1016/j.ecss.2005.09.003

Atlantic States Marine Fisheries Commission (ASMFC). 2014. Addendum XXV to the summer flounder, scup, black sea bass fishery management plan: summer flounder and black sea bass recreational management in 2014. Atlantic States Marine Fisheries Commission, 18p. Accessed online (September 2014): http://www.asmfc.org/uploads/file//53079e93SF_BSB_AddendumXXV_Feb2014.pdf

Bell RJ, Richardson DE, Hare JA, Lynch PD, Fratantoni PS. Disentangling the effects of climate, abundance, and size on the distribution of marine fish: an example based on four stocks from the Northeast US shelf. ICES J Mar Sci. 2014; fsu217. doi: 10.1093/icesjms/fsu217

Drohan AF, Manderson JP, Packer DB. 2007. Essential fish habitat source document: Black sea bass, Centropristis striata, life history and habitat characteristics, 2nd edition. NOAA Tech Memo NMFS NE 200; 68 p. Accessed online (September 2014): http://www.nefsc.noaa.gov/nefsc/publications/tm/tm200/

Howell P, Auster PJ. Phase shift in an estuarine finfish community associated with warming temperatures. Mar Coast Fish. 2012; 4(1): 481-495. doi: 10.1080/19425120.2012.685144

Lavenda N. Sexual differences and normal protogynous hermaphroditism in the Atlantic sea bass, Centropristes striatus. Copeia, 1949; 1949(3): 185-194. http://www.jstor.org/stable/1438985

Northeast Fisheries Science Center. 2012. 53rd Northeast Regional Stock Assessment Workshop (53rd SAW) Assessment Report. US Dept Commer, Northeast Fish Sci Cent Ref Doc. 12-05; 559 p. Available from: National Marine Fisheries Service, 166 Water Street, Woods Hole, MA 02543-1026. Accessed online (September 2014): http://www.nefsc.noaa.gov/publications/crd/crd1205/

Return to Table of Contents

Bloodworm – *Glycera dibranchiata*

Overall Vulnerability Rank = Very High

Biological Sensitivity = High
Climate Exposure = Very High
Data Quality = 71% of scores ≥ 2

Glycera dibranchiata	Expert Scores	Data Quality	Expert Scores Plots (Portion by Category)
Stock Status	2.5	0.6	
Other Stressors	2.4	1.8	
Population Growth Rate	1.7	1.6	
Spawning Cycle	3.0	2.8	
Complexity in Reproduction	2.3	3.0	
Early Life History Requirements	2.4	0.8	
Sensitivity to Ocean Acidification	1.2	1.8	
Prey Specialization	1.3	2.4	
Habitat Specialization	1.6	2.6	
Sensitivity to Temperature	1.2	2.4	
Adult Mobility	3.2	3.0	
Dispersal & Early Life History	3.4	2.4	
Sensitivity Score	High		
Sea Surface Temperature	4.0	3.0	
Variability in Sea Surface Temperature	1.0	3.0	
Salinity	1.7	3.0	
Variability Salinity	1.2	3.0	
Air Temperature	3.6	3.0	
Variability Air Temperature	1.0	3.0	
Precipitation	1.3	3.0	
Variability in Precipitation	1.4	3.0	
Ocean Acidification	4.0	2.0	
Variability in Ocean Acidification	1.0	2.2	
Currents	2.0	1.0	
Sea Level Rise	2.7	1.5	
Exposure Score	Very High		
Overall Vulnerability Rank	Very High		

Low
Moderate
High
Very High

Return to Table of Contents

Bloodworm (*Glycera dibranchiata*)

Overall Climate Vulnerability Rank: **Very High** (95% certainty from bootstrap analysis).

Climate Exposure: **Very High**. Three exposure factors contributed to this score: Ocean Surface Temperature (3.9), Air Temperature (3.6), and Ocean Acidification (4.0). Adult Bloodworm live in intertidal mud habitats and early life stages inhabit in coastal waters.

Biological Sensitivity: **High**. Three sensitivity attributes scored above 3.0: Spawning Complexity (3.0), Adult Mobility (3.2), and Dispersal and Early Life History (3.4). Bloodworm are semelparous and spawning occurs in swarms. Adults have limited mobility and larvae are likely demersal and dispersal is limited.

Distributional Vulnerability Rank: **Low** (100% certainty from bootstrap analysis).

Directional Effect in the Northeast U.S. Shelf: The effect of climate change on Bloodworm is estimated to be negative, but this estimate has a high degree of uncertainty (<66% certainty in expert scores). There is very little information regarding climate effects on Bloodworm. However, warming temperatures may begin restrict the distribution of Bloodworm in the region.

Data Quality: 71% of the data quality scores were 2 or greater indicate that data quality is moderate.

Climate Effects on Abundance and Distribution: There is very little known regarding the long-term population dynamics of Bloodworm. Environmental effects on recruitment, growth, survival and largely unstudied.

Life History Synopsis**:** Bloodworm is an estuarine and marine, semelparous, baitworm species found from the Gulf of St Lawrence to Florida and recorded in the Gulf of Mexico and eastern Pacific Ocean (Wilson and Ruff, 1988). Most Bloodworms mature, spawn, and die after 3 years, but some postpone until 4 or 5 years (Wilson and Ruff, 1988). Bloodworms undergo distinct physical changes to take on the sexual form or epitokes (Wilson and Ruff, 1988). Spawning occurs in swarms during afternoon high tides and only lasts a few days from May-June in Maine and southeastern Nova Scotia and in fall and possibly late spring in Maryland (Wilson and Ruff, 1988; DFO, 2009). Females rupture to release 10 million eggs, and while less dramatic, males also die after spawning (Wilson and Ruff, 1988). Eggs and early larvae occur on the sediment surface, but develop into the swimming stage after 14-20 hours postfertilization (Wilson and Ruff, 1988). The larval stage is either very short or demersal, because larvae are not found in plankton tows, and there is little evidence of larval exchange across mud flats (Wilson and Ruff, 1988; DFO, 2009). Larvae are planktotrophic (Wilson and Ruff, 1988). Adults occur mostly in the fine organic-rich mud of intertidal mud flats, but can be found in a variety of substrates and from the low water mark to 400 m (Wilson and Ruff, 1988; DFO, 2009). The species is quite adaptive to changes in salinity and oxygen content (Wilson and Ruff, 1988). During spring and summer, Bloodworms are generally found buried in the sediment, but in fall and winter occur in the water column (Wilson and Ruff, 1988). The most rapid growth occurs during the second and third years of their 5-year life (Wilson and Ruff, 1988). Bloodworms are probably predators on small invertebrates encountered while burrowing through the mud (such as amphipods and polychaetes), but may also consume detritus when needed (Wilson and Ruff, 1988; DFO, 2009). Black-bellied plovers are known predators of Bloodworms, and Striped Bass and sand shrimp consume the dead, spent Bloodworm bodies (Wilson and Ruff, 1988). Bloodworms are sold as live bait to marine sport fishermen (DFO, 2009). Commercial diggers need a license, but there is very

little regulation in United States waters (Sypitkowski et al., 2009). Bloodworms are harvested primarily in Maine where they seem to be sustainably harvested (Sypitkowski et al., 2009) and southeastern Nova Scotia where they are actively managed to maintain sustainable harvests (DFO, 2009).

Literature Cited:

Department of Fisheries and Oceans (DFO). Nova Scotia Bloodworm (*Glycera dibranchiata*) Assessment: A review of methods and harvest advice. DFO Can. Sci. Advis. Sec. Sci. Advis. Rep. 2009/037. 11pp. Accessed Online (August 2015): http://www.dfo-mpo.gc.ca/CSAS/Csas/Publications/SAR-AS/2009/2009_037_e.pdf

Sypitkowski E, Ambrose Jr WG, Bohlen C, Warren J. Catch statistics in the bloodworm fishery in Maine. Fisher Res. 2009; 96: 303-307. DOI: 10.1016/j.fishres.2008.09.018

Wilson WH, Ruff RE. Species profiles: life histories and environmental requirements of coastal fishes and invertebrates (North Atlantic) – sandworm and bloodworm. U S Fish. Wildl. Serv. Biol. Rep. 82(11.80). U S Army Corps of Engineers, 1988; TR EL-82-4. 23 pp. Accessed Online (August 2015): http://www.nwrc.usgs.gov/publications/specprof.htm

Return to Table of Contents

Blue Crab – *Callinectes sapidus*

Overall Vulnerability Rank = Very High

Biological Sensitivity = High
Climate Exposure = Very High
Data Quality = 92% of scores ≥ 2

Callinectes sapidus	Expert Scores	Data Quality	Expert Scores Plots (Portion by Category)
Stock Status	2.8	2.0	
Other Stressors	3.0	2.4	
Population Growth Rate	1.2	3.0	
Spawning Cycle	2.2	3.0	
Complexity in Reproduction	3.0	2.8	
Early Life History Requirements	2.8	2.8	
Sensitivity to Ocean Acidification	1.6	2.6	
Prey Specialization	1.1	2.8	
Habitat Specialization	2.4	3.0	
Sensitivity to Temperature	1.6	3.0	
Adult Mobility	1.7	2.8	
Dispersal & Early Life History	1.6	3.0	
Sensitivity Score	High		
Sea Surface Temperature	4.0	3.0	
Variability in Sea Surface Temperature	1.0	3.0	
Salinity	2.8	3.0	
Variability Salinity	1.2	3.0	
Air Temperature	4.0	3.0	
Variability Air Temperature	1.0	3.0	
Precipitation	1.3	3.0	
Variability in Precipitation	1.4	3.0	
Ocean Acidification	4.0	2.0	
Variability in Ocean Acidification	1.0	2.2	
Currents	2.0	1.0	
Sea Level Rise	2.7	1.5	
Exposure Score	Very High		
Overall Vulnerability Rank	Very High		

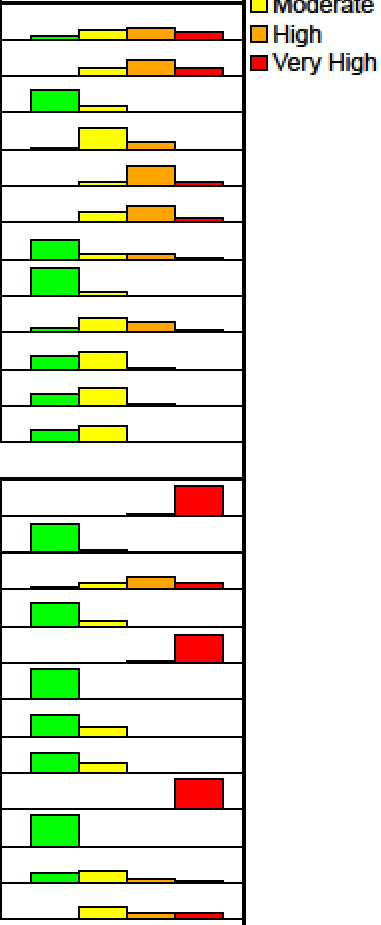

Blue Crab (*Callinectes sapidus*)

Overall Climate Vulnerability Rank: **Very High** (42% certainty from bootstrap analysis).

Climate Exposure: **Very High**. Three exposure factors contributed to this score: Ocean Surface Temperature (3.9), Air Temperature (4.0) and Ocean Acidification (4.0). Blue Crab use estuarine and ocean habitats during different portions of the life cycle.

Biological Sensitivity: **High**. Two sensitivity attributes scored above 3.0: Other Stressors (3.0) and Complexity in Reproduction (3.0). Blue Crab adults are mostly estuarine and exposed to numerous other stressors including contaminants and habitat loss. Fertilization occurs in estuarine waters and adults migrate to estuarine mouths to release eggs. Early life stages are marine and return to estuaries prior to settlement.

Distributional Vulnerability Rank: **High** (99% certainty from bootstrap analysis).

Directional Effect in the Northeast U.S. Shelf: The effect of climate change on Blue Crab on the Northeast U.S. Shelf is estimated to be neutral, but with a moderate degree of uncertainty (66-90% certainty in expert scores). Research suggests that crustaceans are not negatively impacted by ocean acidification. Warming may lead to increased productivity and northward shifts in the region, both of which would represent positive effects of climate change, but more research is needed to confirm these effects.

Data Quality: 92% of the data quality scores were 2 or greater indicate that data quality is moderate.

Climate Effects on Abundance and Distribution: Blue Crab survival in Chesapeake Bay is higher during mild winters (Rome et al., 2005; Bauer and Miller, 2010) and thus, warmer winters should lead to higher survival and increases in population productivity. Blue Crab also are moving into the Gulf of Maine and this has been linked to increasing temperatures (Johnson, 2014). Calcification rate was greater under lower aragonite saturation states, a pattern found in several crustacean species (Ries et al., 2009). Therefore, ocean acidification may not be as detrimental for crustaceans as it is for many molluscs.

Life History Synopsis**:** Blue Crab is an estuarine and coastal shellfish species found from the Gulf of Maine to the eastern coast of South America, including the Gulf of Mexico (Hill et al., 1989). Sexual maturity is reached after 12-30 months, with some evidence that crabs that hatch in spring mature after approximately 15 months, while those that spawn later in the summer may delay maturity until the following spring (approximately 21 months; UMCES, 2011). Blue Crabs are sexually dimorphic, and females likely have a terminal molt (only molt once during adulthood; Hill et al., 1989). Males may maintain the ability to molt after maturity, although seem to do so infrequently (UMCES, 2011). Females can only be mated with during their intermolt phase, and so, only mate once in their lives (Hill et al., 1989). Mating occurs in low-salinity waters in the upper estuary or lower parts of rivers during May – October, immediately after the female molts (Hill et al., 1989). Males carry females from molt to shell hardening, and females store the sperm for use over several spawning events over 1-2 years (Hill et al., 1989). After mating, females migrate to high-salinity water of estuaries, sounds, and near-shore spawning areas and burrow in the mud to wait 2-9 months before spawning (Hill et al., 1989). Some females release eggs (700,000 – 2 million) into a mass attached to the abdomen during the same season as mating, but most appear to wait until the following summer (Hill et al., 1989; UMCES, 2011). Females in the Gulf of Mexico may spawn year round (Vanderkooy, 2013). In Chesapeake Bay, ovigerous females

migrate to the mouth of the bay from where eggs and larvae are carried seaward on the ebb tide (UMCES, 2011). Eggs incubate 1-2 weeks, and hatch in salinities of 23-33 and temperatures of 19-29°C during the nocturnal high tide (Hill et al., 1989; UMCES, 2011). Many fishes and a parasitic worm (*Carcinonemertes carcinophila*) consume eggs, and on average, 1 per million eggs survives to adulthood (Hill et al., 1989). Larvae go through a series of molts and two larval stages before transforming into juveniles. The first larval stage, the zoeae, looks nothing like a grown crab, and individuals drift out to the continental shelf to feed and grow (Hill et al., 1989; UMCES, 2011). Zoeae are planktonic filter feeders on phytoplankton and molt 7-8 times over 1-2 months before reaching the second stage, the megalops (Hill et al., 1989). Megalopae look more crab-like, are 2.5 mm carapace width, and can swim freely, but stay close to the bottom (Hill et al., 1989). The megalop stage lasts 1-3 weeks, during which time the larvae reinvade the lower estuary through vertical migrations (Hill et al., 1989; UMCES, 2011). Megalopae consume fish larvae, small shellfish, and aquatic plants, and cannibalism is common during all life stages (Hill et al., 1989). Fish, shellfish, jellyfish, ctenophores, and many planktivores consume larval Blue Crabs (Hill et al., 1989). First crab, or juvenile-stage individuals look like the adult Blue Crab, and will molt and grow 9 or 10 times by winter, when molting and growth stop (Hill et al., 1989). Over the next year, juveniles gradually migrate to shallow, less-saline water in the upper estuaries and rivers to grow and mature (Hill et al., 1989). Seagrass serves as nursery habitat where the juveniles are omnivorous benthic-carnivores and scavengers on dead and live fish, crabs, organic debris, shrimp, molluscs, and aquatic plants (Hill et al., 1989; UMCES, 2011). Juvenile crabs are prey to many fish, such as Striped Bass, Spotted Sea Trout, Red Drum, Black Drum, and Sheepshead, as well as shorebirds, wading birds, and mammals (Hill et al., 1989; UMCES, 2011). Adult Blue Crabs are coastal, primarily in bays and brackish estuaries (Hill et al., 1989). Males migrate to low-salinity creeks, rivers, and upper estuaries in warm months; females may make short migrations to sea, but generally once females reach the spawning grounds they stay (Hill et al., 1989). Both males and females enter deeper water to bury in mud in winter and emerge in spring to return to rivers, tidal creeks, salt marshes, and sounds (Hill et al., 1989). Temperature and salinity effect growth, so while the number of molts is consistent among crabs, growth rates are not (Hill et al., 1989). Blue Crab eat molluscs, including many commercially valuable clams and oysters, and other invertebrates that bury shallowly, but are also detritivores and scavengers (Hill et al., 1989; UMCES, 2011). Cannibalism is also common (UMCES, 2011). Many estuarine and marine fish consume Blue Crab, including Striped Bass, American Eel, Sandbar Shark, birds, and mammals (Hill et al., 1989). The Blue Crab fisheries are managed by the states, mostly through controls on the harvest of females. Based on a recent assessment, the Chesapeake Bay fishery is not overfished or experiencing overfishing (UMCES, 2011). However, while not overfished or experiencing overfishing, the Gulf of Mexico stock is depleted (Vanderkooy, 2013).

Literature Cited:

Bauer LJ, Miller TJ. Spatial and interannual variability in winter mortality of the blue crab (*Callinectes sapidus*) in the Chesapeake Bay. Estuar Coasts. 2010; 33(3): 678-687. DOI: 10.1007/s12237-009-9237-x

Hill J, Fowler DL, Van Den Avyle MJ. Species profiles: life histories and environmental requirements of coastal fishes and invertebrates (mid-Atlantic)—Blue crab. U S Fish Wildl. Serv. Biol. Rep. 1989; 82(11.100). U. S. Army Corps of Engineers, TR EL-82-4. 18 pp. Accessed online (August 2015): http://www.nwrc.usgs.gov/publications/specprof.htm

Johnson DS. The savory swimmer swims north: a northern range extension of the blue crab *Callinectes sapidus*?. J Crust Biol. 2015; 35(1): 105-110. DOI: 10.1163/1937240X-00002293

Ries JB, Cohen AL, McCorkle DC. Marine calcifiers exhibit mixed responses to CO2-induced ocean acidification. Geol. 2009; 37(12), 1131-1134. doi: 10.1130/G30210A.1

Rome MS, Young-Williams AC, Davis GR, Hines AH. Linking temperature and salinity tolerance to winter mortality of Chesapeake Bay blue crabs (*Callinectes sapidus*). J Exp Mar Biol Ecol. 2005; 319(1): 129-145. DOI: 10.1016/j.jembe.2004.06.014

University of Maryland Center for Environmental Science (UMCES). Stock Assessment of Blue Crab in Chesapeake Bay 2011. Final Assessment Report. Technical Report Series 2011; TS-614-11: 192 pp. Accessed Online (August 2015): http://hjort.cbl.umces.edu/crabs/Assessment.html

VanderKooy SJ. GDAR01 Stock Assessment Report Gulf of Mexico Blue Crab. Gulf States Marine Fisheries Commission. Ocean Springs, MS. 2013. Accessed online (August 2015): http://www.gsmfc.org/publications/GSMFC%20Number%20215.pdf

Return to Table of Contents

Blue Mussel – *Mytilus edulis*

Overall Vulnerability Rank = Very High

Biological Sensitivity = High
Climate Exposure = Very High
Data Quality = 88% of scores ≥ 2

Mytilus edulis	Expert Scores	Data Quality	Expert Scores Plots (Portion by Category)
Stock Status	2.1	0.8	
Other Stressors	2.3	2.0	
Population Growth Rate	1.6	2.1	
Spawning Cycle	1.9	2.7	
Complexity in Reproduction	1.6	2.8	
Early Life History Requirements	2.0	2.8	
Sensitivity to Ocean Acidification	3.6	2.2	
Prey Specialization	1.7	3.0	
Habitat Specialization	2.3	3.0	
Sensitivity to Temperature	1.4	2.9	
Adult Mobility	3.8	3.0	
Dispersal & Early Life History	2.4	2.8	
Sensitivity Score	High		
Sea Surface Temperature	4.0	3.0	
Variability in Sea Surface Temperature	1.0	3.0	
Salinity	1.4	3.0	
Variability Salinity	1.2	3.0	
Air Temperature	3.6	3.0	
Variability Air Temperature	1.0	3.0	
Precipitation	1.3	3.0	
Variability in Precipitation	1.4	3.0	
Ocean Acidification	4.0	2.0	
Variability in Ocean Acidification	1.0	2.2	
Currents	2.0	1.0	
Sea Level Rise	2.1	1.5	
Exposure Score	Very High		
Overall Vulnerability Rank	Very High		

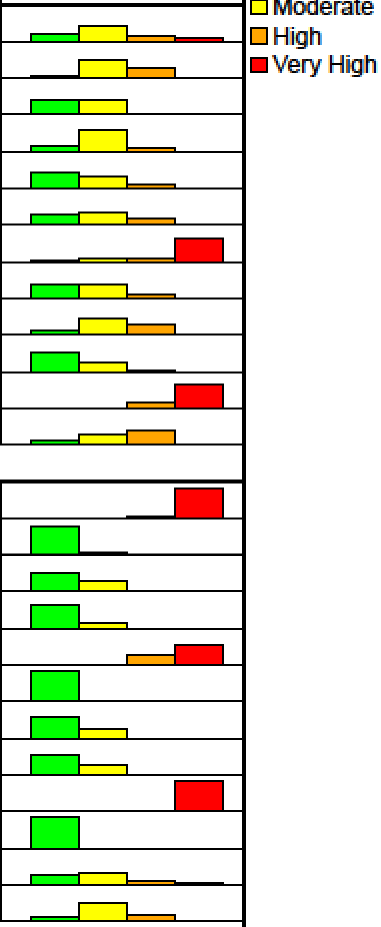

Blue Mussel (*Mytilus edulis*)

Overall Climate Vulnerability Rank: **Very High** (95% certainty from bootstrap analysis).

Climate Exposure: **Very High**. Three exposure factors contributed to this score: Ocean Surface Temperature (4.0), Air Temperature (3.6) and Ocean Acidification (4.0). Blue Mussels use intertidal, nearshore and marine habitats through their life cycle.

Biological Sensitivity: **High**. Two sensitivity attributes scored above 3.0: Sensitivity to Ocean Acidification (3.6) and Adult Mobility (3.8). Adults have a calcium carbonate shell and are sessile.

Distributional Vulnerability Rank: **Moderate** (61% certainty from bootstrap analysis).

Directional Effect in the Northeast U.S. Shelf: The effect of climate change on Blue Mussel on the Northeast U.S. Shelf is very likely to be negative (>95% certainty in expert scores). Ocean acidification will likely negatively impact molluscs, including Blue Mussel. Warming may cause continued shifts northward and may reduce productivity in the southern part of the ecosystem.

Data Quality: 88% of the data quality scores were 2 or greater indicate that data quality is moderate.

Climate Effects on Abundance and Distribution: Blue Mussel are sensitive to heat shock, and along the East Coast of the U.S. large mortality events are associated with high summer temperatures; as a result, distributions have contracted ~350 km to the north since 1960 (Jones et al., 2010). Blue Mussel have also been found recently in the Arctic and this northward range extension was associated with warming temperature and change in circulation patterns (Berge et al., 2005). Blue Mussel adults exhibited no trend in calcification rate in response to changing aragonite saturation state (Ries et al., 2009) and in naturally low pH systems, adult shell growth is similar to control sites. However, shell growth of Blue Mussel larvae is lower at lower pHs (Gazeau et al., 2010). These results point to the complexity of understanding the effect of ocean acidification on marine species.

Life History Synopsis**:** Blue Mussel is a boreo-temperate, semi-sessile, epibenthic bivalve species found in the Arctic, North Pacific, and North Atlantic Oceans. In the western North Atlantic, Blue Mussels are found from Labrador to Cape Hatteras, North Carolina (Newell, 1989). Generally dioecious, although there have been examples of hermaphrodites, Blue Mussel mature at age 1 but can delay maturity if growing conditions have been poor (Newell, 1989). The timing of the reproductive cycle depends on food availability and environmental conditions, so spawning usually occurs in spring and summer, but is actually quite variable in areas where phytoplankton availability is less predictable (Newell, 1989). Spawning can be postponed at any point during gametogenesis if conditions are not right for survival of the larvae or the adult is under stress (Newell, 1989). Fertilization occurs in the water column where males release gametes first, triggering egg release (Newell, 1989). Spawning occurs in either two large batches or a slow dribble, and hundreds of thousands of eggs are produced (Newell, 1989). Eggs hatch in 5 hours post fertilization in 18°C water (Newell, 1989). A ciliated embryo emerges from the egg and develops into the trochophore larvae within 24 hours (Newell, 1989). Trochophore larvae are ciliated and motile, but non-feeding (Newell, 1989). The veliger larval stage lasts up to 35 days and is characterized by the use of a ciliated velum to feed and for locomotion (Newell, 1989). This stage is characterized by the development of the shell, pigmented eyespots, and the foot (Newell, 1989). The veliger larva can move vertically in the water column in response to salinity and may use vertical distribution and currents to move horizontally (Newell, 1989). During these pelagic stages, grazing

predators such as jellyfish and larval and adult fishes regularly consume larval Blue Mussels (Newell, 1989). The final larval stage, the pediveliger, uses the foot to explore for filamentous substrate (such as algae or hydroids) suitable for attachment, then hangs on and transforms into the juvenile form; this early attached life stage is known as plantigrade (Newell, 1989). Juvenile Blue Mussels remain attached to filamentous substrate until 1-1.5 mm shell length is reached (Newell, 1989). After detachment, the Blue Mussel drifts near the bottom until it finds other Blue Mussels, and then reattaches with byssal threads to the substrate or other Blue Mussel shells (Newell, 1989). Only strong predators able to break hard shells consume Blue Mussels, including large starfish, crustaceans, and some birds (Newell, 1989). Adult Blue Mussels occur from the coastal ocean to polyhaline and mesohaline estuarine environments, but can also colonize deeper and cooler waters as far south as Charleston, South Carolina (Newell, 1989). Blue Mussels anchor to the substrate or to other Blue Mussel shells with byssal thread secreted from the foot, but can adjust their position by shortening, lengthening, or replacing byssal threads (Newell, 1989). This species can tolerate a range of temperature, salinity, wave action, and air exposure, the upper and lower limits of which are influenced by previous exposure (e.g., estuarine populations can tolerate lower salinity water than oceanic populations) and have been shown capable of adapting phenotypically to changes in predation pressure (Newell, 1989; Leonard et al., 1999). Mussels are suspension feeders, primarily of phytoplankton, but detritus and attached bacteria may also be digested (Newell, 1989). Birds, crustaceans, starfish, and various fish including Tautog and Cunner are common predators (Newell, 1989). Since most Blue Mussel predators are marine species, the estuaries may provide a refuge (Newell, 1989). Blue Mussels are particularly well suited for mariculture, and have been farmed for decades in Europe, and are increasingly farmed in Canada and the United States (FAO, 2015; GARFO, 2014).

Literature Cited:

Abdelrhman MA, Bergen BJ, Nelson WG. Modeling of PCB concentrations in water and biota (*Mytilus edulis*) in New Bedford Harbor, Massachusetts. Estuar. 1998; 21: 435-448. DOI: 10.2307/1352842

Berge J, Johnsen G, Nilsen F, Gulliksen B, Slagstad D. Ocean temperature oscillations enable reappearance of blue mussels *Mytilus edulis* in Svalbard after a 1000 year absence. Mar Ecol Prog Ser. 2005; 303: 167-175. DOI: 10.3354/meps303167

Food and Agriculture Organization (FAO). Cultured Aquatic Species Information Programme. Mytilus edulis (Linnaeus, 1758). Food and Agriculture Organization of the United Nations. 2015. Accessed online (August 2015): http://www.fao.org/fishery/culturedspecies/Mytilus_edulis/en#tcNA00D6

Greater Atlantic Regional Fisheries Office (GARFO). First federally permitted offshore mussel aquaculture project on east coast soon to get underway. 2014. Accessed online (August 2015): http://www.greateratlantic.fisheries.noaa.gov/stories/2014/first_offshore_mussel_aquaculture_project_on_east_coast_soon_to_get_underway.html

Gazeau FPH, Gattuso JA, Dawber C, Pronker AE, Peene F, Peene J, et al. Effect of ocean acidification on the early life stages of the blue mussel *Mytilus edulis*. Biogeosc. 2010; 7: 2051-2060. DOI:10.5194/bg-7-2051-2010

Jones SJ, Lima FP, Wethey DS. Rising environmental temperatures and biogeography: poleward range contraction of the blue mussel, Mytilus edulis L., in the western Atlantic. J Biogeogr. 2010; 37(12): 2243-2259. DOI: 10.1111/j.1365-2699.2010.02386.x

Leonard GH, Bertness MD, Yund PO. Crab predation, waterborne cues, and inducible defenses in the blue mussel, *Mytilus edulis*. Ecol. 1990; 80(1): 1-14. http://dx.doi.org/10.1890/0012-9658(1999)080[0001:CPWCAI]2.0.CO;2

Newell RIE. Species profiles: life histories and environmental requirements of coastal fishes and invertebrates (North and Mid-Atlantic) – blue mussel. U.S. Fish. Wildl. Serv. Biol. Rep. 1989; 82(11.102). U S Army Corps of Engineers, TR El-82-4. 25 pp. Accessed online (August 2015): http://www.nwrc.usgs.gov/publications/specprof.htm

Ries JB, Cohen AL, McCorkle DC. Marine calcifiers exhibit mixed responses to CO2-induced ocean acidification. Geol. 2009; 37(12), 1131-1134. doi: 10.1130/G30210A.1

Return to Table of Contents

Blueback Herring – *Alosa aestivalis*

Overall Vulnerability Rank = Very High

Biological Sensitivity = High
Climate Exposure = Very High
Data Quality = 88% of scores ≥ 2

Alosa aestivalis	Expert Scores	Data Quality	Expert Scores Plots (Portion by Category)
Stock Status	2.7	1.2	
Other Stressors	2.7	2.0	
Population Growth Rate	2.3	2.0	
Spawning Cycle	3.5	2.8	
Complexity in Reproduction	3.2	3.0	
Early Life History Requirements	3.3	2.5	
Sensitivity to Ocean Acidification	1.5	2.2	
Prey Specialization	1.8	3.0	
Habitat Specialization	2.3	3.0	
Sensitivity to Temperature	1.8	3.0	
Adult Mobility	1.3	2.8	
Dispersal & Early Life History	3.2	2.6	
**Sensitivity Score**	High		
Sea Surface Temperature	4.0	3.0	
Variability in Sea Surface Temperature	1.0	3.0	
Salinity	1.8	3.0	
Variability Salinity	1.2	3.0	
Air Temperature	4.0	3.0	
Variability Air Temperature	1.0	3.0	
Precipitation	1.3	3.0	
Variability in Precipitation	1.4	3.0	
Ocean Acidification	4.0	2.0	
Variability in Ocean Acidification	1.0	2.2	
Currents	2.0	1.0	
Sea Level Rise	2.6	1.5	
**Exposure Score**	Very High		
**Overall Vulnerability Rank**	Very High		

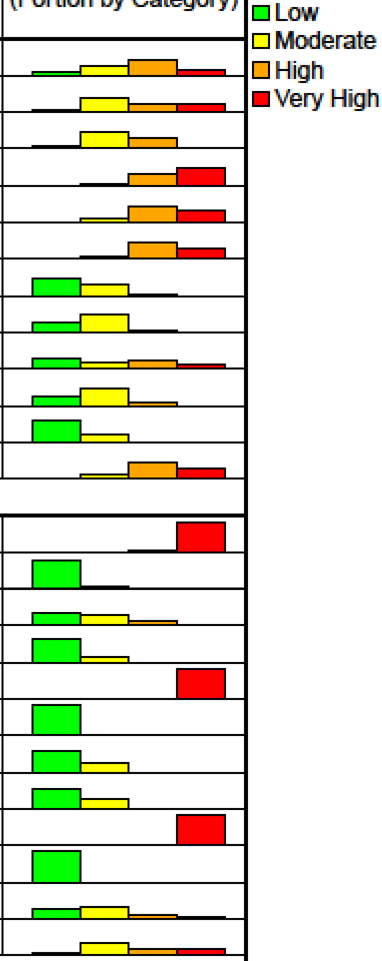

Return to Table of Contents

Blueback Herring (*Alosa aestivalis*)

Overall Climate Vulnerability Rank: **Very High** (100% certainty from bootstrap analysis).

Climate Exposure: **Very High**. Three exposure factors contributed to this score: Ocean Surface Temperature (4.0), Ocean Acidification (4.0) and Air Temperature (4.0). Blueback Herring are anadromous, spawning in freshwater, developing in freshwater and estuarine habitats, feeding as adults in marine habitats.

Biological Sensitivity: **High**. Four sensitivity attributes scored above 3.0: Spawning Cycle (3.5), Early Life History Requirements (3.3), Complexity in Reproduction (3.2), and Dispersal and Early Life History. Blueback Herring spawning time varies latitudinally and is linked to spring warming. Eggs and larvae inhabit freshwaters and then juveniles move to estuarine and ocean waters. Dispersal of eggs and larvae is limited.

Distributional Vulnerability Rank: **Medium** (73% certainty from bootstrap analysis). Blueback Herring have a relatively high degree of spawning site fidelity, limiting the ability of the species to shift distribution. However, migrations occur over a wide range (from Northeast U.S. Shelf to Florida) providing more opportunity for straying from natal spawning sites.

Directional Effect in the Northeast U.S. Shelf: The effect of climate change on Blueback Herring is estimated to be neutral, but this estimate has a high degree of uncertainty (<66% certainty in expert scores). Blueback Herring is distributed to Florida, so northward range shifts will likely move Blueback Herring into the Northeast U.S. Shelf. However, changes in rivers from increased precipitation and warming may cause decreases in productivity particularly in the southern portion of the Northeast U.S. shelf. The magnitude of these contrasting effects is unknown. The effect of ocean acidification over the next 30 years is likely to be minimal.

Data Quality: 88% of the data quality scores were 2 or greater indicate that data quality is moderate.

Climate Effects on Abundance and Distribution: Blueback Herring productivity and distribution are susceptible to climate change. Tommasi et al. (2015) indicated that recruitment was affected by stream temperatures and river flow, both of which will be impacted by climate change. However, the effects of the environment on recruitment were less for Blueback Herring compared to Alewife. A number of other components of Alewife physiology and ecology are affected by temperature (Loesch and Lund, 1977) and other climate factors such as changes in streamflow and sea-level rise may also affect Alewife (NMFS, 2012). Lynch et al. (2014) developed projections of the change in the distribution of thermal habitat. However, natal homing is an important element in Blueback Herring life history, thus the marine distribution may be changing faster than the spawning distribution.

Life History Synopsis**:** Blueback Herring is an anadromous, schooling, coastal pelagic species found in the northwest Atlantic from Cape Breton, Nova Scotia, to the St. Johns River, Florida (Munroe, 2002). Adults reach maturity between ages 3-6 years and females live longer than males (Munroe, 2002). The timing of the spawning season is determined by temperature (peaking when temperatures are 21-25°C), but generally occurs in spring through summer (Munroe, 2002). Blueback Herring make extensive migrations to return to natal streams and prefer areas with swiftly moving fresh or brackish water for spawning (Munroe, 2002). Eggs and sperm are broadcast over the substrate (Munroe, 2002). Eggs scatter to the bottom and adhere to twigs, gravel, or aquatic vegetation till they harden (Munroe, 2002). Spent adults

leave shortly after spawning to return to the sea, but approximately a third of the population is repeat spawners (Munroe, 2002). This proportion of repeat spawners may be decreasing in recent years for many rivers (NMFS, 2013). Once the eggs harden, they become pelagic or semi-pelagic (Munroe, 2002). Incubation takes 3-4 days and yolk absorption takes another 3-4 days post hatch (Munroe, 2002). During this first week, the eggs and early larvae are passively transported downstream to nursery areas with slower moving water (Munroe, 2002). First feeding larvae consumer rotifers with increasing consumption of cladocerans as they grow (Munroe, 2002). Transformation to the juvenile stage is usually complete within a month (Munroe, 2002). Eggs, larvae, and juveniles can tolerate salinities of 28, so juvenile nursery habitat occurs in both freshwater and semi-brackish waters (Munroe, 2002; NMFS, 2013). The diet of juveniles mostly contains copepods and cladocerans from the water column, but occasional benthic prey is also consumed (Munroe, 2002). Many predators consume juvenile Blueback Herring, including: American Eel, Yellow Perch, White Perch, turtles, snakes, birds, and mink (Munroe, 2002). Adult herring occur north of 40 degrees in water <13 ^{o}C during summer and fall and between 40-43 degrees during winter (Munroe, 2002). During spring, the distribution expands to include the continental shelf from Cape Hatteras, North Carolina, to Nova Scotia as the Blueback Herring return to natal streams for spawning (Munroe, 2002). Adult and juvenile Blueback Herring make diel migrations to the upper water column at night and down in the water column during the day (Munroe, 2002). There are also landlocked populations in freshwater lakes that are likely the result of stocking and dams blocking the juvenile egress. These populations are genetically distinct from the anadromous population (NMFS, 2013). Adult herring are planktivorous and primarily consume ctenophores, calanoid copepods, amphipods, mysids, pelagic shrimps, and small fishes at sea (Munroe, 2002). During the spawning migration up river, herring consume planktonic organisms and terrestrial insects (Munroe, 2002). Blueback Herring are preyed upon by a variety of predators such as Spiny Dogfish, American Eel, Atlantic Cod, Silver Hake, White Hake, Atlantic Halibut, Bluefish, Weakfish, Striped Bass, seals, gulls, and terns (Munroe, 2002). River herring, which includes Alewife, was once an important fishery, but most stocks are either depleted or of unknown status due to a lack of time-series data (ASMFC, 2012). A 2011 petition to have river herring listed as endangered was determined to be unwarranted at the time of review (NMFS, 2013).

Literature Cited:

Atlantic States Marine Fisheries Commission (ASMFC). 2012. River Herring Benchmark Stock Assessment, Volumes I. Stock Assessment Report No. 12-02 of the Atlantic States Marine Fisheries Commission. Accessed online (October 2014): http://www.asmfc.org/species/shad-river-herring

Loesch JG, Lund JR WA. A contribution to the life history of the blueback herring, Alosa aestivalis. Trans Am Fish Soc. 1977; 106(6): 583-589. doi: 10.1577/1548-8659(1977)106<583:ACTTLH>2.0.CO;2

Lynch PD, Nye JA, Hare JA, Stock CA, Alexander MA, Scott JD, et al. Projected ocean warming creates a conservation challenge for river herring populations. ICES J Mar Sci. 2015; 72(2): 374-387. doi: 10.1093/icesjms/fsu134

Munroe T. Herring and herring-like fishes: Order Clupeiformes. In: B.B. Collette BB, Klein-MacPhee G, editors, Fishes of the Gulf of Maine, 3rd ed. Washington: Smithsonian Institution Press; 2002.pp. 104–158.

National Marine Fisheries Service (NMFS). 2012. River Herring Climate Change Workshop Report. Greater Atlantic Fishery Regional Office, Gloucester, Massachusetts, 60p. http://www.greateratlantic.fisheries.noaa.gov/prot_res/CandidateSpeciesProgram/sswpdocs/RIVER%20HERRING%20CLIMATE%20CHANGE%20WORKSHOP%20REPORT_122712.pdf

National Marine Fisheries Service (NMFS). 2013.Endangered and Threatened Wildlife and Plants; Endangered Species Act Listing Determination for Alewife and Blueback Herring. Federal Register Notice. 78: 48944. 52pp. Accessed Online (October 2014): http://www.greateratlantic.fisheries.noaa.gov/regs/2013/august/13riverherringlistingesanoticefr.pdf

Tommasi D, Nye J, Stock C, Hare JA, Alexander M, Drew K. Effect of Environmental Conditions on Juvenile Recruitment of Alewife (Alosa pseudoharengus) and Blueback Herring (A. aestivalis) in fresh water: A Coastwide Perspective. Can J Fish Aquat Sci. 2015; 72(7): 1037-1047. doi: 10.1139/cjfas-2014-0259

Return to Table of Contents

Bluefish – *Pomatomus saltatrix*

Overall Vulnerability Rank = Low

Biological Sensitivity = Low
Climate Exposure = High
Data Quality = 92% of scores ≥ 2

Pomatomus saltatrix	Expert Scores	Data Quality	Expert Scores Plots (Portion by Category)
Stock Status	2.0	3.0	
Other Stressors	2.2	2.4	
Population Growth Rate	1.9	2.8	
Spawning Cycle	2.0	3.0	
Complexity in Reproduction	1.7	2.6	
Early Life History Requirements	2.7	2.7	
Sensitivity to Ocean Acidification	1.2	2.4	
Prey Specialization	1.1	3.0	
Habitat Specialization	1.9	3.0	
Sensitivity to Temperature	1.1	3.0	
Adult Mobility	1.1	2.8	
Dispersal & Early Life History	1.4	2.7	
Sensitivity Score	Low		
Sea Surface Temperature	4.0	3.0	
Variability in Sea Surface Temperature	1.0	3.0	
Salinity	2.5	3.0	
Variability Salinity	1.2	3.0	
Air Temperature	2.1	3.0	
Variability Air Temperature	1.0	3.0	
Precipitation	1.1	3.0	
Variability in Precipitation	1.1	3.0	
Ocean Acidification	4.0	2.0	
Variability in Ocean Acidification	1.0	2.2	
Currents	2.1	1.0	
Sea Level Rise	1.5	1.5	
Exposure Score	High		
Overall Vulnerability Rank	Low		

Low
Moderate
High
Very High

Return to Table of Contents

Bluefish (*Pomatomus saltatrix*)

Overall Climate Vulnerability Rank: **Low** (100% certainty from bootstrap analysis).

Climate Exposure: **High**. Two exposure factors contributed to this score: Ocean Surface Temperature (4.0) and Ocean Acidification (4.0). Exposure to ocean surface temperature and ocean acidification occurs during all life stages.

Biological Sensitivity: **Low**. Only one sensitivity attribute scored above 2.5: Early Life History Requirements (2.7). Bluefish spawn in waters of defined temperatures and spawning shifts from south to north to south through the seasonal cycle (Wuenschel et al., 2012).

Distributional Vulnerability Rank: **High** (81% certainty from bootstrap analysis). Three of the attributes indicated vulnerability to distribution shift. Bluefish are pelagic and habitat generalists. They are highly mobile and make large seasonal migrations, and larvae disperse over large areas.

Directional Effect in the Northeast U.S. Shelf: The effect of climate change on Bluefish on the Northeast U.S. Shelf is very likely to be positive (>95% certainty in expert scores). As warming continues more habitat in the Northeast U.S. is expected to become available and seasonal movements and spawning may extend into the Gulf of Maine. The effect of ocean acidification over the next 30 years is likely to be minimal.

Data Quality: 92% of the data quality scores were 2 or greater.

Climate Effects on Abundance and Distribution: Few studies examined the effect of climate factors on the population productivity of Bluefish. Changes in distribution, however, have been documented. In the Mediterranean, changes in Bluefish distribution have been linked to warming waters, and changes in spawning locations have been identified (Sabatés et al., 2012). Along the Northeast U.S. Shelf, changes in distribution have not be documented, but an extension of the spawning season into the early fall has been observed (Walsh et al., 2015).

Life History Synopsis**:** Bluefish is a migratory, coastal, warm-temperate species found from Nova Scotia to Argentina, but rare from southern Florida to northern South America (Shepherd and Packer, 2006). Adults reach maturity by age 2 (Shepherd and Packer, 2006). There is most likely one spawning stock with two or three peaks in spawning. Spawning begins in late spring south of Cape Hatteras, North Carolina, with a peak in March-May near the continental shelf edge (Hare and Cowen, 1996). A second peak on the continental shelf of the Mid-Atlantic region occurs from June - August (Shepherd and Packer, 2006). There is also evidence of a small, third peak in fall off the southeast US coast in September to January (McBride et al., 1993). Pelagic eggs hatch within 2 days at 18-22 ^{o}C (Shepherd and Packer, 2006). Larvae are pelagic and associated with the upper 15 m of the water column and concentrated just below the surface (Shepherd and Packer, 2006). The spring-spawned cohort occurs near the Gulf Stream and is probably advected north in the current (Hare and Cowen, 1996); the summer-spawned cohort occurs in the mid-Atlantic region (Shepherd and Packer, 2006). Larvae prey on copepods (Shepherd and Packer, 2006). After 2-4 weeks, larvae transition into pelagic juveniles, remain associated with the surface waters, and spend spring and summer offshore (Shepherd and Packer, 2006). Pelagic juveniles begin including fish in their diet at 30 mm and are almost exclusively piscivorous by 40 mm (Shepherd and Packer, 2006). When the water temperature rises above 13-15 ^{o}C, pelagic juveniles actively move into estuaries and coastal nurseries (Shepherd and Packer, 2006). The northern

transport of eggs and larvae and the cross-shelf migration of juveniles are aided by oceanographic features such as the Gulf Stream, wind-driven surface flow, warm-core ring streamers, and Gulf Stream filaments (Shepherd and Packer, 2006). Juvenile Bluefish use soft substrates and occasionally grass, algae, or oyster beds in estuaries, bays, and coastal areas as nurseries. The spring-spawned cohort recruits to estuarine habitats in the mid-Atlantic in late May to mid-June or 2-3 months after spawning (Shepherd and Packer, 2006). Individuals from the summer-spawned cohort enter estuaries or stay in coastal nursery areas in mid to late August, or 1-2 months after spawning (Shepherd and Packer, 2006). Young Bluefish are prey to oceanic birds and rarely cannibalism (Shepherd and Packer, 2006). Both cohorts leave mid-Atlantic estuaries and coastal areas in October to overwinter in warmer waters south of Cape Hatteras, North Carolina (Shepherd and Packer, 2006). Adult Bluefish move north into the mid-Atlantic region during spring and summer (Shepherd and Packer, 2006). Bluefish prefer oceanic salinities and warmer temperatures of the open-ocean, large-embayment, and estuarine systems, and travel in schools of similarly sized individuals (Shepherd and Packer, 2006). Late juveniles and adults eat locally abundant fish and benthic invertebrates, targeting schooling species such as squids, clupeids, and Butterfish while offshore (Shepherd and Packer, 2006). Sharks, tunas, and billfishes are the only predators large and fast enough to prey on adult Bluefish (Shepherd and Packer, 2006). Bluefish is jointly managed by the Atlantic States Marine Fisheries Commission and the Mid-Atlantic Fishery Management Council as a single stock (ASMFC, 2012). The stock has rebuilt over the last 20 years and is neither overfished nor experiencing overfishing (ASMFC, 2012; NEFSC, 2012).

Literature Cited:

Atlantic States Marine Fisheries Commission (ASMFC). 2012. Addendum I to Amendment 1 to the bluefish fishery management plan. 3p. Accessed online (October 2014): http://www.asmfc.org/uploads/file/bluefishAddendumI.pdf

Hare JA., Cowen RK. Transport mechanisms of larval and pelagic juvenile bluefish (Pomatomus saltatrix) from South Atlantic Bight spawning grounds to Middle Atlantic Bight nursery habitats. Limnol Oceanogr. 1996; 41(6) 1264–1280. doi: 10.4319/lo.1996.41.6.1264

McBride, RS, JL Ross, DO Conover. Recruitment of bluefish Pomatomus saltatrix to estuaries of the U.S. South Atlantic Bight. Fish Bull. 1993; 91: 389-395.

Northeast Fisheries Science Center (NEFSC). 2012. Bluefish 2012 Stock Assessment Update. Coastal/Pelagic Working Group, Northeast Fisheries Science Center, National Marine Fisheries Service. Woods Hole, MA. 36p. Accessed Online (October 2014): http://www.asmfc.org/uploads/file/bluefishStockAssmtUpdate_July2012.pdf

Sabatés A, Martín P, Raya V. Changes in life-history traits in relation to climate change: bluefish (Pomatomus saltatrix) in the northwestern Mediterranean. ICES J Mar Sci. 2012; 69(6): 1000-1009. doi: 10.1093/icesjms/fss053

Shepherd GR, Packer DB. 2006. Essential fish habitat source document: Bluefish, Pomatomus saltatrix, life history and habitat characteristics (2nd Edition). NOAA Tech Memo NMFS NE 198; 89 p. Available: http://www.nefsc.noaa.gov/publications/tm/tm198/

Walsh HJ, Richardson DE, Marancik KE, Hare JA. 2015. Long-term changes in the distributions of larval and adult fish in the Northeast U.S. Shelf Ecosystem. PLOS ONE. doi: 10.1371/journal.pone.0137382

Return to Table of Contents

Wuenschel MJ, Able KW, Buckel JA, Morley JW, Lankford T, Branson AC, et al. Recruitment patterns and habitat use of young-of-the-year bluefish along the United States east coast: insights from coordinated coastwide sampling. Rev Fish Sci. 2012; 20(2): 80-102. DOI: 10.1080/10641262.2012.660999

Return to Table of Contents

Butterfish – *Peprilus triacanthus*

Overall Vulnerability Rank = Low

Biological Sensitivity = Low
Climate Exposure = High
Data Quality = 88% of scores ≥ 2

Peprilus triacanthus	Expert Scores	Data Quality	Expert Scores Plots (Portion by Category)
Stock Status	1.2	2.2	
Other Stressors	1.4	1.8	
Population Growth Rate	1.1	2.5	
Spawning Cycle	2.2	2.8	
Complexity in Reproduction	1.5	2.4	
Early Life History Requirements	2.1	2.2	
Sensitivity to Ocean Acidification	1.4	2.6	
Prey Specialization	1.6	2.6	
Habitat Specialization	1.5	2.9	
Sensitivity to Temperature	1.8	2.8	
Adult Mobility	1.1	2.6	
Dispersal & Early Life History	1.4	2.5	
Sensitivity Score	Low		
Sea Surface Temperature	4.0	3.0	
Variability in Sea Surface Temperature	1.0	3.0	
Salinity	2.1	3.0	
Variability Salinity	1.2	3.0	
Air Temperature	2.1	3.0	
Variability Air Temperature	1.0	3.0	
Precipitation	1.1	3.0	
Variability in Precipitation	1.1	3.0	
Ocean Acidification	4.0	2.0	
Variability in Ocean Acidification	1.0	2.2	
Currents	2.1	1.0	
Sea Level Rise	1.5	1.5	
Exposure Score	High		
Overall Vulnerability Rank	Low		

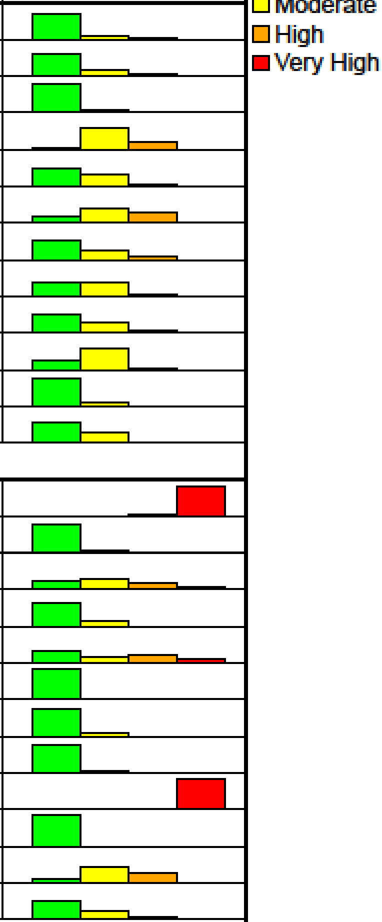

Return to Table of Contents

Butterfish (*Peprilus traicanthus*)

Overall Climate Vulnerability Rank: **Low** (100% certainty from bootstrap analysis).

Climate Exposure: **High**. Two exposure factors contributed to this score: Ocean Surface Temperature (4.0) and Ocean Acidification (4.0). Exposure to ocean surface temperature and ocean acidification occurs during all life stages.

Biological Sensitivity: **Low**. All sensitivity scores were below 2.5.

Distributional Vulnerability Rank: **Very High** (52% certainty from bootstrap analysis). Three of the attributes indicated vulnerability to distribution shift: Butterfish are habitat generalists. They are highly mobile and make large seasonal migrations. Spawning occurs across the shelf and larvae are broadly dispersed.

Directional Effect in the Northeast U.S. Shelf: The effect of climate change on Butterfish on the Northeast U.S. Shelf is very likely to be positive (>95% certainty in expert scores). As warming continues more habitat in the Northeast U.S. is expected to become available. Recent excursions into the Gulf of Maine have occurred in recent years and this is likely to continue as the ecosystem warms. The effect of ocean acidification over the next 30 years is likely to be minimal.

Data Quality: 88% of the data quality scores were 2 or greater.

Climate Effects on Abundance and Distribution: Several studies indicate that Butterfish distribution is vulnerable to changes in climate. Colton (1972) showed that Butterfish distribution shifted southwards during an extended period of cooling on the Northeast U.S. Shelf (1953-1967) and Collie et al. (2008) found that Butterfish abundance increased in Narragansett Bay during the more recent warming trend (1970-2005). Manderson et al. (2011) developed a habitat model for Butterfish on the Northeast U.S. Shelf, which suggests that, as the Northeast U.S. Shelf warms, more pelagic habitat will become available for Butterfish.

Life History Synopsis**:** Butterfish is a short-lived, pelagic species that occurs from the Gulf of St Lawrence to southern Florida, but is primarily found from Maine to Cape Hatteras, North Carolina (Able and Fahay, 2010). Butterfish mature in 1-2 years (Cross et al., 1999). Spawning begins in late winter (or year round) south of Cape Hatteras, and continues to the north and inshore as water temperatures increase, peaking in spring and summer (Able and Fahay, 2010). Spawning is primarily on the continental shelf, but some spawning in coastal areas and in large estuaries has also been documented (Able and Fahay, 2010). Butterfish are broadcast spawners, and the resultant pelagic eggs incubate for 2-3 days at approximate 18-20°C before hatching (Cross et al., 1999). Larvae are pelagic, become increasingly nektonic with growth and development, and often associate with jellyfish, Sargassum, and floating debris during the late-larval stage (Cross et al., 1999). Larvae develop gradually into pelagic juveniles and then adults (Cross et al., 1999; Able and Fahay, 2010). Juvenile Butterfish are pelagic or nektonic and while often associated with large jellyfish, are also frequently seen swimming freely in loose schools (Cross et al., 1999; Able and Fahay, 2010). The juvenile Butterfish diet consists of abundant plankton including copepods and ctenophores (Able and Fahay, 2010). Juveniles can reach half their adult size in the first year and follow similar migration patterns to adult Butterfish (Cross et al., 1999; Able and Fahay, 2010). Adult and juvenile Butterfish form loose schools and migrate in response to changing temperatures. Butterfish move to offshore areas along the continental shelf edge south of Georges Bank in winter,

then migrate inshore and to the north as temperatures rise above 10 ^{o}C (Cross et al., 1999; Able and Fahay, 2010). During spring and summer, Butterfish can be found across the shelf and into large estuaries (Cross et al., 1999). These cross-shelf migrations are less pronounced south of Cape Hatteras where the temperatures stay mild (Cross et al., 1999; Able and Fahay, 2010). While generally associated with the upper water column, Butterfish can also be found near the bottom, particularly during winter, and are frequently collected over sand, mud, or rock substrates (Cross et al., 1999). Adult Butterfish are planktivores consuming urochordates, mulluscs, crustaceans, jellyfish, polychaetes, small fishes, and ctenophores (Cross et al., 1999; NEFSC, 2014). The predators of Butterfish include Silver Hake, Monkfish (Goosefish), Weakfish, Bluefish, Swordfish, and Hammerhead Sharks (NEFSC, 2014). Butterfish are managed by the Mid-Atlantic Fishery Management Council and are neither overfished nor is overfishing occurring (NEFSC, 2014).

Literature Cited:

Able KW, Fahay MP. Ecology of estuarine fishes: temperate waters of the western North Atlantic. Baltimore: The Johns Hopkins University Press; 2010. 566p.

Collie JS, Wood AD, Jeffries HP. Long-term shifts in the species composition of a coastal fish community. Can J Fish Aquat Sci. 2008; 65(7): 1352-1365. doi: 10.1139/F08-048

Colton Jr. JB. Temperature trends and the distribution of groundfish in continental shelf waters, Nova Scotia to Long Island. Fish Bull. 1972; 70: 637-658.

Cross JN, Zetlin CA, Berrien PL, Johnson DL, McBride C. 1999. Essential fish habitat source document: Butterfish, Peprilus triacanthus, life history and habitat characteristics. NOAA Tech Memo NMFS NE 145; 42 p. Accessed online (October 2014): http://www.nefsc.noaa.gov/nefsc/habitat/efh/. Available: http://www.nefsc.noaa.gov/publications/tm/tm145/

Manderson J, Palamara L, Kohut J, Oliver MJ. Ocean observatory data are useful for regional-habitat modeling of species with different vertical habitat preferences. Mar Ecol Prog Ser. 2011; 438: 1-17. doi:10.3354/meps09308

Northeast Fisheries Science Center (NEFSC). 2014. 58th Northeast Regional Stock Assessment Workshop (58th SAW) Assessment Report. US Dept Commer, Northeast Fish Sci Cent Ref Doc. 14-0. Accessed online (October 2014): http://nefsc.noaa.gov/publications/crd/crd1404/

Return to Table of Contents

Cancer Crabs – *Cancer borealis/C. irroratus*

Overall Vulnerability Rank = Moderate

Biological Sensitivity = Moderate
Climate Exposure = High
Data Quality = 75% of scores ≥ 2

Cancer borealis/C. irroratus	Expert Scores	Data Quality	Expert Scores Plots (Portion by Category)
Stock Status	2.0	0.8	
Other Stressors	2.0	1.6	
Population Growth Rate	1.3	1.6	
Spawning Cycle	2.6	2.0	
Complexity in Reproduction	1.2	2.0	
Early Life History Requirements	2.0	2.2	
Sensitivity to Ocean Acidification	2.2	2.6	
Prey Specialization	1.1	2.4	
Habitat Specialization	1.2	2.2	
Sensitivity to Temperature	1.8	3.0	
Adult Mobility	2.7	1.9	
Dispersal & Early Life History	1.7	3.0	
Sensitivity Score	Moderate		
Sea Surface Temperature	4.0	3.0	
Variability in Sea Surface Temperature	1.0	3.0	
Salinity	1.9	3.0	
Variability Salinity	1.2	3.0	
Air Temperature	2.8	3.0	
Variability Air Temperature	1.0	3.0	
Precipitation	1.1	3.0	
Variability in Precipitation	1.1	3.0	
Ocean Acidification	4.0	2.0	
Variability in Ocean Acidification	1.0	2.2	
Currents	2.1	1.0	
Sea Level Rise	1.8	1.5	
Exposure Score	High		
Overall Vulnerability Rank	Moderate		

Low
Moderate
High
Very High

Return to Table of Contents

Cancer Crabs (*Cancer borealis / Cancer irroratus*)

Overall Climate Vulnerability Rank: **Moderate** (75% certainty from bootstrap analysis).

Climate Exposure: **High**. Two exposure factors contributed to this score: Ocean Surface Temperature (3.9) and Ocean Acidification (4.0). All life stages of Cancer Crabs use marine habitats.

Biological Sensitivity: **Moderate**. Two sensitivity attributes scored above 2.5: Spawning Cycle (2.6) and Adult Mobility (2.7). Spawning occurs in warmer months after molting and adults have low mobility.

Distributional Vulnerability Rank: **High** (100% certainty from bootstrap analysis).

Directional Effect in the Northeast U.S. Shelf: The effect of climate change on Cancer Crabs on the Northeast U.S. Shelf is estimated to be neutral, but with a moderate degree of uncertainty (66-90% certainty in expert scores). Research suggests that crustaceans are not negatively impacted by ocean acidification. Cancer Crabs are temperate and cold-water species, so warming may reduce the availabile habitat in the region, but specific studies have not yet been completed.

Data Quality: 75% of the data quality scores were 2 or greater indicate that data quality is moderate.

Climate Effects on Abundance and Distribution: Relatively little is known regarding the dynamics of Cancer Crabs in the Northeast U.S. Shelf. Abundance of Cancer Crabs have been increasing in Narragansett Bay, but multiple causes have been proposed (Collie et al., 2008). Larval growth and metamorphosis of *C. irroratus* is temperature dependent with maximum rates observed at 15-18°C (Johns, 1981). *C. irroratus* is more common in temperate waters, whereas *C. borealis* is more common in boreal waters suggesting the response to climate change may differ between species (Jeffries, 1966).

Life History Synopsis**:** Cancer Crabs are benthic marine species found in intertidal to shelf slope habitats from Nova Scotia, Canada, to Florida (Robichaud and Frail, 2006). In the region, two species are harvesterd as Cancer Crab: Rock Crab (*Cancer irroratus*) and Jonah Crab (*Cancer borealis*). Rock Crabs reach maturity between 1 and 2 years (DFA, 1999). Females spawn while their shell is still soft after molting between April and October, producing one brood per year (Bigford, 1979; DFA, 1999; ISFMP, 2013). Eggs and the first 6 stages of post-hatch development occur in the neuston and are transported via wind and tide toward shore (DFA, 1999). These early life history stages are omnivorous planktivores and preyed upon by larger zooplankton and ichthyoplankton (DFA, 1999). Cancer Crabs settle to the benthos when megalopae molt to the first crab instar (Bigford, 1979). Cancer Crabs occur in shallow waters in the northern part of their range, and are more likely to be offshore in the southern part (Bigford, 1979; DFA, 1999; ISFMP, 2013). Jonah Crabs are generally more abundant in deeper, more thermally stable, habitat with rocky, silt, or clay substrates than Rock Crab which can tolerate a larger temperature range and prefer sandy substrates (Bigford, 1979; ISFMP, 2013). Rock Crab appear to migrate between inshore and offshore habitat seasonally (Bigford, 1979). Adult Cancer Crabs are generalist predators on benthic organisms such as mussels, polychaetes and sea urchins, as well as scavenging on crab and fish remains (DFA, 1999). Small, inshore crabs are common prey for Cunner, sculpins, and American Lobster, while Atlantic Cod and other large fish are common predators of offshore populations (ISFMP, 2013). Cancer Crabs will be managed by the Atlantic States Marine Fisheries Commission starting in 2016 (ASMFC 2015), owing to a growing targeted fishery and high bycatch levels in the American Lobster fishery (ISFMP, 2013).

Literature Cited:

Atlantic States Marine Fisheries Commission (ASMFC). Interstate Fishery Management Plan for Jonah Crab. Accessed Online (August 2015): http://www.asmfc.org/uploads/file/55d7720eJonahCrabInterstateFMP_Aug2015.pdf

Bigford TE. Synopsis of biological data on the rock crab, *Cancer irroratus* Say. NOAA Technical Report NMFS Circular 1979; 426: 26 pp. Accessed online (August 2015): http://spo.nmfs.noaa.gov/Circulars/CIRC426.pdf

Collie JS, Wood AD, Jeffries HP. Long-term shifts in the species composition of a coastal fish community. Can J Fish Aquat Sci. 2008; 65(7): 1352-1365. DOI: 10.1139/F08-048

Department of Fisheries and Aquaculture. Emerging Species Profile Sheets: Rock Crab (Cancer irroratus). 1999. Accessed online (August 2015): http://www.fishaq.gov.nl.ca/research_development/fdp/rock_crab.pdf

Interstate Fisheries Management Plan Policy Board (IFMPB). Cancer Crab Fishery Overview. 2013. Accessed online (August 2015): http://www.asmfc.org/files/Meetings/Annual2013/ISFMP_PolicyBoard_Supplemental.pdf

Jeffries HP. Partitioning of the estuarine environment by two species of *Cancer*. Ecology, 1966; 47: 477-481. DOI: http://dx.doi.org/10.2307/1932987

Johns DM. Physiological studies on *Cancer irroratus* larvae. I. Effects of temperature and salinity on survival, development rate and size. Mar Ecol Prog Ser. 1991; 5: 75-83. Accessed Online (August 2015): http://www.int-res.com/articles/meps/5/m005p075.pdf

Robichaud DA, Frail C. Development of Jonah crab, *Cancer borealis*, and rock crab, *Cancer irroratus*, fisheries in the Bay of Fundy (LFAs 35-38) and off southwest Nova Scotia (LFA 34): from exploratory to commercial status (1995-2004). Can Manuscr Rep Fish Aquat Sci. 2006; 2775: 48 pp. Accessed online (August 2015): http://www.dfo-mpo.gc.ca/Library/326113.pdf

Return to Table of Contents

Channeled Whelk – *Busycotypus canaliculatus*

Overall Vulnerability Rank = Very High

Biological Sensitivity = High
Climate Exposure = Very High
Data Quality = 75% of scores ≥ 2

Busycotypus canaliculatus	Expert Scores	Data Quality	Expert Scores Plots (Portion by Category)
Stock Status	2.3	0.4	
Other Stressors	1.8	1.1	
Population Growth Rate	2.9	2.3	
Spawning Cycle	3.0	2.4	
Complexity in Reproduction	1.6	2.1	
Early Life History Requirements	2.1	1.8	
Sensitivity to Ocean Acidification	3.9	2.6	
Prey Specialization	1.4	2.1	
Habitat Specialization	1.4	2.1	
Sensitivity to Temperature	1.8	2.6	
Adult Mobility	2.8	1.8	
Dispersal & Early Life History	3.5	2.5	
Sensitivity Score	High		
Sea Surface Temperature	3.9	3.0	
Variability in Sea Surface Temperature	1.0	3.0	
Salinity	1.6	3.0	
Variability Salinity	1.2	3.0	
Air Temperature	3.5	3.0	
Variability Air Temperature	1.0	3.0	
Precipitation	1.3	3.0	
Variability in Precipitation	1.3	3.0	
Ocean Acidification	4.0	2.0	
Variability in Ocean Acidification	1.0	2.2	
Currents	2.0	1.0	
Sea Level Rise	1.6	1.5	
Exposure Score	Very High		
Overall Vulnerability Rank	Very High		

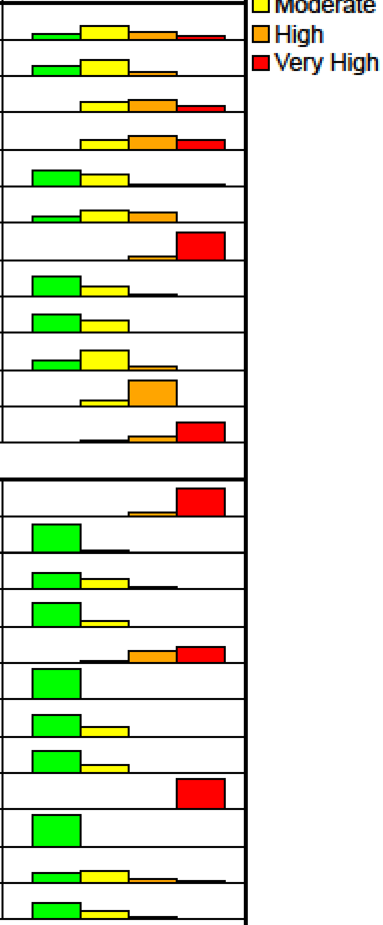

Return to Table of Contents

Channeled Whelk (*Busycotypus canaliculatus*)

Overall Climate Vulnerability Rank: **Very High** (58% certainty from bootstrap analysis).

Climate Exposure: **Very High**. Three exposure factors contributed to this score: Ocean Surface Temperature (3.9), Air Temperature (3.5) and Ocean Acidification (4.0). Channeled Whelk are found in inner shelf and nearshore waters.

Biological Sensitivity: **High**. Three sensitivity attributes scored above 3.0: Sensitivity to Ocean Acidification (3.9), Spawning Cycle (3.0), and Dispersal and Early Life History (3.5). Adults have a calcium carbonate shell. Eggs are attached to the bottom in a gelatinous egg sack in the fall. Eggs hatch the following spring and dispersal of larvae is believed to be limited.

Distributional Vulnerability Rank: **Low** (100% certainty from bootstrap analysis).

Directional Effect in the Northeast U.S. Shelf: The effect of climate change on Channeled Whelk on the Northeast U.S. Shelf is very likely to be negative (>95% certainty in expert scores). Ocean acidification will likely negatively impact molluscs, including Channeled Whelk. Although there is relatively little information, warming has the potential to cause northward distribution shifts and may reduce productivity in the southern part of the ecosystem.

Data Quality: 75% of the data quality scores were 2 or greater indicate that data quality is moderate.

Climate Effects on Abundance and Distribution: There is relatively little information regarding the population dynamics and climate effects on productivity and distribution of Channeled Whelk. Ries et al. (2009) found that calcification rate of other gastropods decreased with decreasing aragonite saturation state. Thus ocean acidification may reduce population productivity, but specific experiments on Channeled Whelk are needed.

Life History Synopsis**:** Channeled Whelk is a coastal shellfish species found from Cape Cod, Massachusetts to Cape Canaveral, Florida (Peemoeller and Stevens, 2013). Female Whelks grow larger and tend to be older than males (Peemoeller and Stevens, 2013). Sexual maturity is reached around 6.9 years for males and 8.6 years for females (Peemoeller and Stevens, 2013). Eggs are fertilized internally, and then the females produce a string of egg capsules attached to the sand on intertidal and shallow subtidal mudflats during fall (Harding, 2011). Eggs hatch the following spring (Harding, 2011). Hatchlings look like small, immature adults and likely remain in nearshore, infaunal nursery habitats before moving to deeper, nearshore and coastal waters, but this stage of development is rarely observed in the wild (Harding, 2011). Juvenile Channeled Whelks move by crawling with the foot or inflating the foot and floating at the surface (Harding, 2011). Channeled Whelks are known to eat bivalves, but the true extent of their diet is unknown. Channeled Whelks likely enter the fishery before (females) or just after (males) adults have a chance to reproduce and the fishery selects for the larger females (Peemoeller and Stevens, 2013), so overfishing is a concern. Whelks are fished and managed on a state-by-state basis throughout their range.

Literature Cited:

Harding JM. Observations on the early life history and growth rates of juvenile channel whelks *Busycotypus canaliculatus* (Linnaeus, 1758). J Shell Res. 2011; 30(3): 901–903. DOI: http://dx.doi.org/10.2983/035.030.0331

Return to Table of Contents

Peemoeller B-J. Stevens B. Age, size, and sexual maturity of *Busycotypus canaliculatus* in Buzzards Bay, Massachusetts. Fish Bull. 2013; 11(3): 265-278. Accessed online: http://fishbull.noaa.gov/1113/peemoeller.pdf

Return to Table of Contents

Clearnose Skate – *Raja eglanteria*

Overall Vulnerability Rank = Low

Biological Sensitivity = Low
Climate Exposure = High
Data Quality = 83% of scores ≥ 2

Raja eglanteria	Expert Scores	Data Quality	Expert Scores Plots (Portion by Category)
Stock Status	2.1	2.4	
Other Stressors	1.2	1.2	
Population Growth Rate	2.8	1.6	
Spawning Cycle	1.9	2.4	
Complexity in Reproduction	1.2	2.2	
Early Life History Requirements	1.0	3.0	
Sensitivity to Ocean Acidification	1.4	2.2	
Prey Specialization	1.2	3.0	
Habitat Specialization	1.2	3.0	
Sensitivity to Temperature	2.3	2.7	
Adult Mobility	1.8	2.2	
Dispersal & Early Life History	2.1	3.0	
Sensitivity Score	Low		
Sea Surface Temperature	3.9	3.0	
Variability in Sea Surface Temperature	1.0	3.0	
Salinity	2.6	3.0	
Variability Salinity	1.2	3.0	
Air Temperature	1.0	3.0	
Variability Air Temperature	1.0	3.0	
Precipitation	1.0	3.0	
Variability in Precipitation	1.0	3.0	
Ocean Acidification	4.0	2.0	
Variability in Ocean Acidification	1.0	2.2	
Currents	2.1	1.0	
Sea Level Rise	1.1	1.5	
Exposure Score	High		
Overall Vulnerability Rank	Low		

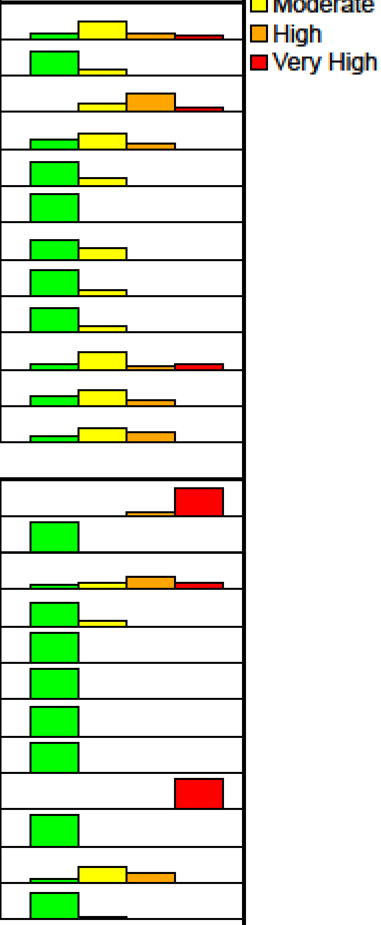

Return to Table of Contents

Clearnose Skate (*Raja eglanteria*)

Overall Climate Vulnerability Rank: **Low** (87% certainty from bootstrap analysis).

Climate Exposure: **High**. Two exposure factors contributed to this score: Ocean Surface Temperature (3.9) and Ocean Acidification (4.0). Clearnose Skate are demersal and complete their life cycle in marine habitats.

Biological Sensitivity: **Low**. One attribute scored above 2.5: Population Growth Rate (2.8). In general, skates have a low population growth rate (higher sensitivity to climate change) (Frisk 2010).

Distributional Vulnerability Rank: **High** (94% certainty from bootstrap analysis). Clearnose Skate are habitat generalists and moderately mobile as adults, making seasonal movements. In addition, skate egg cases are subject to movement by currents and juveniles may move on scales of 1-10 km.

Directional Effect in the Northeast U.S. Shelf: The effect of climate change on Clearnose Skate is likely to be neutral (90-95% certainty in expert scores). Clearnose Skate inhabits temperate waters and may benefit from warming on the Northeast U.S. Shelf. Ocean acidification may reduce productivity and no changes in distribution have been observed over the past 30 years despite significant warming.

Data Quality: 83% of the data quality scores were 2 or greater indicate that data quality is moderate.

Climate Effects on Abundance and Distribution: Little specific information exists on the effect of climate on Clearnose Skate. Di Santo (2015) found that increased warming and acidification reduce body condition of newly-hatched Little Skate. These reductions in size could result in reduced juvenile survival and thus recruitment if similar effects occur in Clearnose Skate. In regional studies of distribution, Clearnose Skate was not included (Murawski, 1993; Nye et al., 2009) but examination of NEFSC trawl survey suggests no change in the center of the distribution over the last 30 years (http://oceanadapt.rutgers.edu/, website last checked 13 June 2015).

Life History Synopsis**:** Clearnose Skate is a benthic marine skate species found from the Nova Scotian shelf to northeastern Florida and the northern Gulf of Mexico from northwestern Florida to Texas, but primarily occurs south of Cape Cod, Massachusetts (Packer et al., 2003). Clearnose Skate reach maturity in 5-6 years (Packer et al., 2003). Spawning occurs in spring and summer north of Cape Hatteras, and eggs are deposited on the benthos in rectangular, horned egg cases (Packer et al., 2003). Eggs incubate for approximately 3 months, and then a small juvenile emerges (Packer et al., 2003). There is no larval stage and juveniles behave similarly to adults (Packer et al., 2003). Clearnose Skate occur year round south of Cape Hatteras, North Carolina (Packer et al., 2003). North of Cape Hatteras, Clearnose Skate are found across the shelf in the southern portion of the Mid-Atlantic region during winter and spring, and then occupy inshore areas south of Cape Cod in summer and fall (NEFSC, 2007). Clearnose Skates are found over soft, rocky, or gravelly bottom (Packer et al., 2003). The diet of Clearnose Skate includes: polychaetes, amphipods, several shrimp and crab species, bivalves, squid, and small fish such as soles, Weakfish, Butterfish, and Scup (Packer et al., 2003). Boring snails may be predators of eggs (Packer et al., 2003). Adults have been found in the stomachs of Sand Tiger and Greater Amberjack (Packer et al., 2003). Clearnose Skate is managed as part of the skate complex by the New England Fishery Management Council and is neither overfished nor is overfishing occurring (NEFSC, 2007).

Return to Table of Contents

Literature Cited:

Di Santo V. Ocean acidification exacerbates the impacts of global warming on embryonic little skate, *Leucoraja erinacea* (Mitchill). J Exp Mar Bio Ecol. 2015; 463: 72-78. doi:10.1016/j.jembe.2014.11.006

Frisk MG. Life history strategies of batoids. In: Carrier, JC, Musick, JA, Heithaus, MR, editors, Sharks and Their Relatives II: Biodiversity, Adaptive Physiology, and Conservation. Boca Raton: CRC Press; 2010. 283-318.

Murawski SA. Climate change and marine fish distributions: forecasting from historical analogy. Trans Am Fish Soc. 1993; 122(5): 647-658. doi: 10.1577/1548-8659(1993)122<0647:CCAMFD>2.3.CO;2

Northeast Fisheries Science Center (NEFSC). 2007. 44th Northeast Regional Stock Assessment Workshop (44th SAW): 44th SAW assessment report. US Dep Commer, Northeast Fish Sci Cent Ref Doc 07-10; 661 p. Accessed online (August 2015): http://www.nefsc.noaa.gov/publications/crd/crd0710/

Nye JA, Link JS, Hare JA, Overholtz WJ. Changing spatial distribution of fish stocks in relation to climate and population size on the Northeast United States continental shelf. Mar Ecol Prog Ser. 2009; 393: 111-129. doi: 10.3354/meps08220

Packer DB, Zetlin CA, Vitaliano JJ. 2003. Essential fish habitat source document: Clearnose skate, Raja eglanteria, life history and habitat characteristics. NOAA Tech Memo NMFS NE 174; 50 p. Accessed online (August 2015): http://www.nefsc.noaa.gov/nefsc/publications/tm/tm174/

Return to Table of Contents

Conger Eel – *Conger oceanicus*

Overall Vulnerability Rank = High

Biological Sensitivity = Moderate
Climate Exposure = Very High
Data Quality = 62% of scores ≥ 2

Conger oceanicus	Expert Scores	Data Quality	Expert Scores Plots (Portion by Category)
Stock Status	2.4	0.5	
Other Stressors	2.5	1.2	
Population Growth Rate	2.1	0.8	
Spawning Cycle	2.9	2.4	
Complexity in Reproduction	2.4	1.9	
Early Life History Requirements	2.5	1.8	
Sensitivity to Ocean Acidification	1.2	1.3	
Prey Specialization	1.6	2.1	
Habitat Specialization	2.4	3.0	
Sensitivity to Temperature	1.6	2.8	
Adult Mobility	1.5	1.8	
Dispersal & Early Life History	1.3	2.8	
Sensitivity Score	Moderate		
Sea Surface Temperature	4.0	3.0	
Variability in Sea Surface Temperature	1.0	3.0	
Salinity	2.4	3.0	
Variability Salinity	1.2	3.0	
Air Temperature	4.0	3.0	
Variability Air Temperature	1.0	3.0	
Precipitation	1.3	3.0	
Variability in Precipitation	1.4	3.0	
Ocean Acidification	4.0	2.0	
Variability in Ocean Acidification	1.0	2.2	
Currents	2.2	1.0	
Sea Level Rise	2.4	1.5	
Exposure Score	Very High		
Overall Vulnerability Rank	High		

Low
Moderate
High
Very High

Return to Table of Contents

Conger Eel (*Anguilla oceanica*)

Overall Climate Vulnerability Rank: **High** (93% certainty from bootstrap analysis).

Climate Exposure: **Very High**. Three exposure factors contributed to this score: Ocean Surface Temperature (4.0), Ocean Acidification (4.0) and Air Temperature (4.0). Conger Eel are semelparous: spawning in the ocean, developing in marine and estuarine habitats, then feeding growing, and maturing in marine and estuarine habitats.

Biological Sensitivity: **Moderate**. Three sensitivity attributes scored above 2.5: Spawning Cycle (2.9), Other Stressors (2.5), and Early Life History Requirements (2.5). Conger Eel found in coastal waters and are exposed to a number of other stressors including habitat destruction, blockage to spawning habitats, and contaminants (Limburg and Waldman, 2009). Conger Eel migrate to specific areas in the Sargasso Sea to spawn, specialized larval stages – leptocephali – disperse and enter shelf and coastal areas throughout North American while undergoing metamorphosis.

Distributional Vulnerability Rank: **High** (100% certainty from bootstrap analysis). Two attributes indicated vulnerability to distribution shift. Conger Eel larvae are widely dispersed over much of the North Atlantic and adults are highly mobile returning to the Sargasso Sea from shelf and coastal systems along most of the North American coast.

Directional Effect in the Northeast U.S. Shelf: The effect of climate change on Conger Eel is estimated to be neutral, but this estimate has uncertainty (66-90% certainty in expert scores). Conger Eel is found in a range of habitats and it is unclear the effect of climate change on population productivity and distribution. However, Conger Eel spawn in the Sargasso Sea, so shifts in the Gulf Stream have the potential to effect larval dispersal, survival, and potentially recruitment, but the direction and magnitude of these effects are unclear. The effect of ocean acidification over the next 30 years is likely to be minimal.

Data Quality: 62% of the data quality scores were 2 or greater indicate that data quality is moderate.

Climate Effects on Abundance and Distribution: The ecology of Conger Eel is not well understood and little work has been done examining links between climate factors and Conger Eel abundances and distribution. Conger Eel occur across a wide range of habitats from estuaries to the shelf break (Hood et al., 1988) so climate effects are likely to be complicated.

Life History Synopsis**:** The Conger Eel is a marine species which spawns in the Sargasso Sea and uses the continental shelf, from estuaries to the shelf break, for nursery and adult habitat (McCleave and Miller, 1994). Adult eels range from Massachusettes to Florida and the eastern Gulf of Mexico (Smith and Tighe, 2002). In late October to mid-December, congers migrate from the shelf to the Sargasso Sea between the subtropical convergence zone in the north and the Bahamas and Antilles in the south seeking the sharp change in temperature of the oceanic front to trigger spawning (McCleave and Miller, 1994). Like many other eel species, Conger Eels are mostly likely semelparous and die after spawning (Able and Fahay, 2010). Eggs hatch into ribbon-like, pelagic leptocephalus larvae (Able and Fahay, 2010). During the 2-4 months between hatching and the onset of metamorphosis (Correia et al., 2004), Conger Eel leptocephali are capable swimmers, able to swim from the Gulf Stream to estuarine and near-shore habitat (Smith and Tighe, 2002; Wuenschel and Able, 2008). Most Conger Eels reach estuarine and coastal areas as 5-6 month old metamorphosing leptocephali and become bentho-pelagic

(Correia et al., 2004; Wuenschel and Able, 2008; Able and Fahay, 2010). Congers remain in nursery habitat until winter, then migrate to the continental shelf adult feeding habitat (Able and Fahay, 2010). Adult Conger Eels are associated with structure such as holes and crevices in piers, wrecks, and rocky reefs or share Tilefish burrows (Able and Fahay, 2010). Congers have been observed in shallow coastal waters out to the shelf edge and may also make seasonal inshore-offshore migrations (Able and Fahay, 2010). Adults are piscivorous on gadids, Atlantic herring, American Eel, and Butterfish, but occasionally consume crustaceans (shrimps) and small molluscs (Bowman et al., 2000; Smith and Tighe, 2002). Spiny Dogfish are the main predator of Conger Eel, though other large piscivores like Cobia, Atlantic Cod, and Dusky Shark are also known to prey on Conger Eel (Smith and Tighe, 2002). There are minor commercial and recreational fisheries for Conger Eel, but they are not currently managed in the United States.

Literature Cited:

Able KW, Fahay MP. 2010. Ecology of estuarine fishes: temperate waters of the western North Atlantic. Baltimore: The Johns Hopkins University Press; 2010. 566p.

Bowman RE, Stillwill CE, Michaels WL, Grosslein MD. 2000. Food of Northwest Atlantic Fishes and Two Common Species of Squid. US Dep Commer, NOAA Tech Memo NMFS NE 155. 137 p. Available: http://www.nefsc.noaa.gov/publications/tm/tm155/

Correia AT. Able KW, Antunes C, Coimbra J. Early life history of the Conger Eel eel (Conger oceanicus) as revealed by otolith microstructure and microchemistry of metamorphosing leptocephali. Mar Biol. 2004; 145: 477-488. doi: 10.1007/s00227-004-1349-z

Hood PB, Able KW, Grimes CB. Biology of the conger eel Conger oceanicus in the Mid-Atlantic Bight. Mar Biol. 1988; 98(4): 587-596. doi: 10.1007/BF00391549

McCleave JD, Miller MJ. Spawning of Conger oceanicus and Conger triporiceps (Congridae) in the Sargasso Sea and subsequent distribution of leptocephali. Environ Biol Fishes. 1994; 39: 339-355. doi: 10.1007/BF00004803

Smith DG, Tighe KA. Conger Eel/ Conger oceanicus (Mitchill 1818). In: B.B. Collette BB, Klein-MacPhee G, editors, Fishes of the Gulf of Maine, 3rd ed. Washington: Smithsonian Institution Press; 2002. p. 101-102

Wuenschel MJ, Able KW. Swimming ability of eels (Anguilla rostrata, Conger oceanicus) at estuarine ingress: contrasting patterns of cross-shelf transport? Mar Biol. 2008; 154: 775-786. doi: 10.1007/s00227-008-0970-7

Return to Table of Contents

Cusk – *Brosme brosme*

Overall Vulnerability Rank = High

Biological Sensitivity = High
Climate Exposure = High
Data Quality = 67% of scores ≥ 2

Brosme brosme	Expert Scores	Data Quality	Expert Scores Plots (Portion by Category)
Stock Status	3.0	0.4	
Other Stressors	1.5	0.8	
Population Growth Rate	3.1	1.6	
Spawning Cycle	3.0	2.4	
Complexity in Reproduction	1.3	1.5	
Early Life History Requirements	2.6	0.2	
Sensitivity to Ocean Acidification	1.2	1.6	
Prey Specialization	1.1	2.4	
Habitat Specialization	2.7	2.4	
Sensitivity to Temperature	2.0	2.4	
Adult Mobility	2.7	2.4	
Dispersal & Early Life History	1.8	2.2	
Sensitivity Score	High		
Sea Surface Temperature	3.9	3.0	
Variability in Sea Surface Temperature	1.0	3.0	
Salinity	1.1	3.0	
Variability Salinity	1.2	3.0	
Air Temperature	1.0	3.0	
Variability Air Temperature	1.0	3.0	
Precipitation	1.0	3.0	
Variability in Precipitation	1.0	3.0	
Ocean Acidification	4.0	2.0	
Variability in Ocean Acidification	1.0	2.2	
Currents	2.1	1.0	
Sea Level Rise	1.1	1.5	
Exposure Score	High		
Overall Vulnerability Rank	High		

Low Moderate High Very High

Return to Table of Contents

Cusk (*Brosme brosme*)

Overall Climate Vulnerability Rank: **High** (69% certainty from bootstrap analysis).

Climate Exposure: **High**. Two exposure factors contributed to this score: Ocean Surface Temperature (3.9) and Ocean Acidification (4.0). All life stages of Cusk use marine habitats.

Biological Sensitivity: **High**. Three sensitivity attributes scored above 3.0: Stock Status (3.0), Population Growth Rate (3.1), and Spawning Cycle (3.0). Cusk is a NMFS Species of Concern owing to apparent low population sizes (Hare et al., 2012). Peak spawning occurs during summer and adults are late maturing and slow growing (Klein-MacPhee, 2002).

Distributional Vulnerability Rank: **Low** (96% certainty from bootstrap analysis).

Directional Effect in the Northeast U.S. Shelf: The effect of climate change on Cusk on the Northeast U.S. Shelf is very likely to be negative (>95% certainty in expert scores). Cusk is a cold-temperate species and warming will likely reduce habitat and increase population fragmentation.

Data Quality: 67% of the data quality scores were 2 or greater indicate that data quality is moderate.

Climate Effects on Abundance and Distribution: Relatively little is known regarding the dynamics of Cusk. Hare et al. (2012) projected that the amount of habitat for Cusk in the Gulf of Maine will decrease with climate change and that the available habitat will become more fragmented. Both could potentially result in lower population productivity. Nye et al. (2009) reported that Cusk have shifted deeper in recent years consistent. Thus both productivity and distribution will likely be impacted.

Life History Synopsis**:** Cusk is a marine, sedentary, solitary, benthic species found on both sides of the Atlantic, ranging from the Straits of Belle Isle and the Grand Banks of Newfoundland to New Jersey in the western Atlantic Ocean (Klein-MacPhee, 2002). Males begin to mature at age 5, females at age 6, and complete maturity for both genders occurs by age 10 (Klein-MacPhee, 2002). Spawning occurs between March and November with peak spawning in spring and summer (Klein-MacPhee, 2002). Cusk are highly fecund and produce pelagic eggs (Klein-MacPhee, 2002). Larval Cusk are pelagic, float near the surface, and are often associated with jellyfish (Klein-MacPhee, 2002). Little is known about the juvenile stage, but settlement to benthic habitats occurs around 50 mm, at which time they are believed to adopt the solitary and sedentary habits of the adults (Klein-MacPhee, 2002). Cusk <51 cm consume a diet of polychaetes, Crangon shrimp, and hermit crabs (Klein-MacPhee, 2002). Adult Cusk prefer hard bottom in moderately deep areas of the shelf and slope and also associate with deepwater coral (Klein-MacPhee, 2002). Cusk is a cool-water species that is entirely benthic and rarely moves (Klein-MacPhee, 2002). The diet of adult Cusk includes: crustaceans, fish, and echinoderms (Klein-MacPhee, 2002). The primary predator of Cusk is Spiny Dogfish, but Cusk have also been found in the stomachs of Winter Skate, Atlantic Cod, Atlantic Halibut, White Hake, Monkfish (Goosefish), Fawn Cusk-eel, Sea Raven, Summer Flounder and Windowpane, hooded seal, and gray seal (Klein-MacPhee, 2002; Harris and Hanke, 2010). Cusk are not managed in United States waters, but are being considered for listing as endangered or threatened after drastic reductions in abundance due to fishing pressure and climate-change induced habitat loss (Hare et al., 2012).

Return to Table of Contents

Literature Cited:

Hare JA, Manderson JP, Nye JA, Alexander MA, Auster PJ, Borggaard DL, et al. Cusk (*Brosme brosme*) and climate change: assessing the threat to a candidate marine fish species under the US Endangered Species Act. ICES J Mar Sci. 2012; 69: 1753–1768. DOI: 10.1093/icesjms/fss160

Harris LE, Hanke AR. Assessment of the Status, Threats and Recovery Potential of Cusk (*Brosme brosme*). DFO Can. Sci. Advis. Sec. Res. Doc. 2010/004. vi + 23 p. Accessed online (August 2015): http://www.dfo-mpo.gc.ca/CSAS/Csas/publications/resdocs-docrech/2010/2010_004_e.pdf

Klein-MacPhee G. Cusk/ *Brosme brosme* (Ascanius 1772). Pages 223-225. In: BB Collette, G Klein-MacPhee (editors), Fishes of the Gulf of Maine, 3rd edition. Smithsonian Institution Press, Washington D.C. 2002; 882 p.

Return to Table of Contents

Deep–sea Red Crab – *Chaceon quinquedens*

Overall Vulnerability Rank = Low

Biological Sensitivity = Low
Climate Exposure = High
Data Quality = 79% of scores ≥ 2

Chaceon quinquedens	Expert Scores	Data Quality	Expert Scores Plots (Portion by Category)
Stock Status	2.3	0.8	
Other Stressors	1.5	2.0	
Population Growth Rate	2.1	1.4	
Spawning Cycle	2.1	2.4	
Complexity in Reproduction	1.4	2.5	
Early Life History Requirements	1.8	1.2	
Sensitivity to Ocean Acidification	2.1	2.6	
Prey Specialization	1.2	2.8	
Habitat Specialization	1.3	3.0	
Sensitivity to Temperature	2.2	2.4	
Adult Mobility	2.1	2.6	
Dispersal & Early Life History	1.5	2.4	
Sensitivity Score	Low		
Sea Surface Temperature	3.9	3.0	
Variability in Sea Surface Temperature	1.0	3.0	
Salinity	1.8	3.0	
Variability Salinity	1.2	3.0	
Air Temperature	1.0	3.0	
Variability Air Temperature	1.0	3.0	
Precipitation	1.0	3.0	
Variability in Precipitation	1.0	3.0	
Ocean Acidification	4.0	2.0	
Variability in Ocean Acidification	1.0	2.2	
Currents	2.1	1.0	
Sea Level Rise	1.1	1.5	
Exposure Score	High		
Overall Vulnerability Rank	Low		

Low
Moderate
High
Very High

Deep-Sea Red Crab (*Chaceon quinquedens*)

Overall Climate Vulnerability Rank: **Low** (98% certainty from bootstrap analysis).

Climate Exposure: **High**. Two exposure factors contributed to this score: Ocean Surface Temperature (3.9) and Ocean Acidification (4.0). All life stages of Deep-Sea Red Crab use marine habitats.

Biological Sensitivity: **Low**. No sensitivity attributes scored above 2.5.

Distributional Vulnerability Rank: **High** (100% certainty from bootstrap analysis).

Directional Effect in the Northeast U.S. Shelf: The effect of climate change on Deep-sea Red Crab on the Northeast U.S. Shelf is estimated to be neutral, but with a moderate degree of uncertainty (66-90% certainty in expert scores). Research suggests that crustaceans are not negatively impacted by ocean acidification. Warming may negatively impact Deep-sea Red Crab and reduce habitat availability, but there is a lot of uncertainty.

Data Quality: 79% of the data quality scores were 2 or greater indicate that data quality is moderate.

Climate Effects on Abundance and Distribution: There is very little information on the effect of climate forcing on Deep-Sea Red Crabs. Most studies indicate that acidification has neutral or positive effects on crustacean in general (Kroeker et al., 2013), but no specific studies have been done. Temperature effects on Deep-Sea Red Crab are also little known, but their continental slope habitat is warming (Forsyth et al., 2015).

Life History Synopsis**:** Deep-sea Red Crab is a long-lived crustacean that occurs along the outer continental shelf and slope from the Gulf of Saint Lawrence to possibly the western South Atlantic including the Gulf of Maine and Gulf of Mexico (Steimle et al., 2001). Males mature at about 75 mm carapace width; females likely mature at smaller sizes (NMFS, 2006). Females occur in shallower waters of the upper slope than males, so males may migrate from deeper, mid-slope waters for spawning (Steimle et al., 2001). Males form a protective cage around females, carrying them for up to three weeks, before the female molts and copulation occurs (Steimle et al., 2001). Males may need to be larger than females for mating to occur (NMFS, 2006). Females are believed to go 5-7 years between molts, meaning spawning may not occur annually for each individual, but females may be able to store sperm to fertilize eggs between molts (Steimle et al., 2001; NMFS, 2006). Eggs are attached to the underside of females for up to 9 months before hatching (Steimle et al., 2001). Hatching occurs between January and June (NMFS, 2006). Larvae are released into the water column and may actively swim to the surface where they exist for 23-125 days (based on lab-reared larvae) and transition through four zoeal and one megalops stages before settling to the bottom (Steimle et al., 2001). Larvae are rarely collected, but are likely zooplanktivorous (Steimle et al., 2001). Settlement to the middle or base of the slope occurs at 4 mm carapace width, which is fairly large for brachyuran crabs (Steimle et al., 2001). Juveniles prefer smooth or dimpled silt-clay sediments and occur deeper than the adult population (Steimle et al., 2001). Young red crabs move upslope to shallower waters as they grow and become available to the fishery at about 100 mm carapace width and age 4-6 years (Steimle et al., 2001). Juvenile red crabs are opportunistic foragers on infaunal and epifaunal benthic inverts and small fish including: sponges, hydroids, gastropods, scaphopods, small polychaetes, crustaceans, tunicates, and bivalves (Steimle et al., 2001). Adult Deep-sea Red Crabs occur on the slope and outer continental shelf and travel short distances except for a possible spawning migration up and down the slope (Steimle et

al., 2001). The diet of adults includes similar benthic fauna to juvenile red crab and larger prey such as demersal and mid-water fish, squid, Quill Worm and scavenging deadfalls of fish and squid (Steimle et al., 2001). Red crab is not a major prey species for any predator, but has been found in Longfin Hake, Atlantic Cod, and Red Hake stomachs (Steimle et al., 2001). Deep-sea Red Crab is managed under the New England Fishery Management Council's Deep-Sea Red Crab Fishery Management Plan and is probably not being overfished, but poor estimates of biomass and productivity prevent a designation (NMFS, 2006).

Literature Cited:

Forsyth JST, Andres M, Gawarkiewicz GG. Recent accelerated warming of the continental shelf off New Jersey: Observations from the CMV Oleander expendable bathythermograph line. J Geophys Res Oceans. 2015; 120(3): 2370-2384. DOI: 10.1002/2014JC010516

Kroeker KJ, Kordas RL, Crim R, Hendriks IE, Ramajo L, Singh GS, et al. Impacts of ocean acidification on marine organisms: quantifying sensitivities and interaction with warming. Global Change Biol. 2013; 19(6): 1884-1896. DOI: 10.1111/gcb.12179

Northeast Fisheries Science Center (NEFSC). 43rd Northeast Regional Stock Assessment Workshop (43rd SAW): 43rd SAW assessment report. US Dep Commer, Northeast Fish Sci Cent Ref Doc 2006; 06-25: 400 p. Accessed Online (August 2015): http://www.nefsc.noaa.gov/publications/crd/crd0625/

Steimle FW, Zetlin CA, Chang S. Essential Fish Habitat Source Document: Red Deepsea Crab, Chaceon (Geryon) quinquedens, Life History and Habitat Characteristics. US Dep Commer, NOAA Tech Memo NMFS NE 2001; 163: 27 p. Accessed online (October 2014): http://www.nefsc.noaa.gov/nefsc/publications/tm/tm163/

[Return to Table of Contents](#)

Dusky Shark – *Carcharhinus obscurus*

Overall Vulnerability Rank = High

Biological Sensitivity = High
Climate Exposure = High
Data Quality = 88% of scores ≥ 2

Carcharhinus obscurus	Expert Scores	Data Quality	Expert Scores Plots (Portion by Category)
Stock Status	3.9	2.4	
Other Stressors	1.8	2.2	
Population Growth Rate	4.0	3.0	
Spawning Cycle	2.2	2.2	
Complexity in Reproduction	1.5	1.2	
Early Life History Requirements	1.0	2.6	
Sensitivity to Ocean Acidification	1.0	2.6	
Prey Specialization	1.1	2.8	
Habitat Specialization	1.1	3.0	
Sensitivity to Temperature	1.1	3.0	
Adult Mobility	1.0	3.0	
Dispersal & Early Life History	1.2	3.0	
Sensitivity Score	High		
Sea Surface Temperature	3.9	3.0	
Variability in Sea Surface Temperature	1.0	3.0	
Salinity	2.8	3.0	
Variability Salinity	1.2	3.0	
Air Temperature	1.0	3.0	
Variability Air Temperature	1.0	3.0	
Precipitation	1.0	3.0	
Variability in Precipitation	1.0	3.0	
Ocean Acidification	4.0	2.0	
Variability in Ocean Acidification	1.0	2.2	
Currents	2.1	1.0	
Sea Level Rise	1.1	1.5	
Exposure Score	High		
Overall Vulnerability Rank	High		

Low
Moderate
High
Very High

Dusky Shark (*Carcharhinus obscurus*)

Overall Climate Vulnerability Rank: **High** (100% certainty from bootstrap analysis).

Climate Exposure: **High**. Two exposure factors contributed to this score: Ocean Surface Temperature (3.9) and Ocean Acidification (4.0). Dusky Shark are pelagic and complete their life cycle in marine habitats.

Biological Sensitivity: **High**. Two attributes scored above 3.0: Population Growth Rate (4.0) and Stock Status (3.9). Dusky Shark have low population growth rates (Cortés 1998). Dusky Shark is listed as vulnerable by the IUCN owing to low population abundance (http://www.iucnredlist.org/details/3852/0). Dusky Shark is identified as a Species of Concern in the Western Atlantic by the U.S. (http://www.nmfs.noaa.gov/pr/pdfs/species/duskyshark_detailed.pdf).

Distributional Vulnerability Rank: **Very High** (99% certainty from bootstrap analysis). Dusky Shark are habitat generalists and highly mobile. In addition, Dusky Shark are a placental, viviparous species and do not have a larval stage.

Directional Effect in the Northeast U.S. Shelf: The effect of climate change on Dusky Shark is very likely to be neutral (>95% certainty in expert scores). Dusky Shark is a highly mobile temperate shark. There is very little information available that suggests negative or positive effects of climate change.

Data Quality: 88% of the data quality scores were 2 or greater indicate that data quality is moderate.

Climate Effects on Abundance and Distribution: There is very little information on the effect of climate change on Dusky Shark. Chin et al. (2010) conducted a vulnerability assessment of sharks and rays on Australia's Great Barrier Reef (GBR) identifying similar factors for use in their vulnerability assessment, and ranked the level of exposure and sensitivity to these factors using current knowledge and expert opinion based on a 3 point scale (low, moderate, and high). Dusky shark exposure rankings were highly influenced by water temperature but sensitivity to this factor was ranked low for GBR sharks. Although the population growth rate was taken into account in the GBR study, little is known about the population status of sharks in this area (Chin et al. 2010, McAuley et al., 2012). GBR Dusky Sharks were assessed a low vulnerability ranking with respect to climate change.

Life History Synopsis**:** Dusky Shark is a large, coastal, migratory, warm-temperate shark species found from southern New England to the Caribbean and the Gulf of Mexico to southern Brazil (SEDAR, 2011). Males of the species reach 50% maturity at 231 cm fork length (FL) (17.4 years); females are slightly larger (234 cm FL) and 17.6 years at 50% maturity (Natanson et al.; 1995, Natanson et al., 2013). Dusky Sharks are viviparous with an 18-month gestation period and around 7 pups per litter (Castro 2009; Romine et al. 2009). Young Dusky Sharks are large at birth, 90-100 cm total length, and occur in warm-temperate, nearshore areas with sand or rocky bottoms (Branstetter, 2002; McCandless et al., 2007). Rarely, juveniles may enter estuarine habitats, but generally avoid areas of low salinity (McCandless et al., 2007). Adult Dusky Sharks seasonally migrate great distances between New England and the Gulf of Mexico (Branstetter, 2002; Kohler et al. 1998). The diet of Dusky Sharks includes several species of teleosts, elasmobranchs, crustaceans, and squid (Branstetter, 2002). The Atlantic States Marine Fisheries Commission manages Dusky Sharks through an interstate fishery management plan and NMFS manages them as part of the Consolidated Atlantic Highly Migratory Species Fishery Management Plan (ASMFC 2008; NMFS 2006). Based on the last northwest Atlantic stock assessment, Dusky Sharks are

considered to be overfished and experiencing overfishing (SEDAR, 2011) and landing of the species is prohibited (ASMFC, 2008; NMFS, 2006).

Literature Cited:

Atlantic States Marine Fisheries Commission (ASMFC). 2008. Management Report No. 46 of the Atlantic States Marine Fisheries Commission. 172p. Accessed online (August 2015): http://www.asmfc.org/uploads/file/interstateFMPforAtlanticCoastalSharks.pdf

Branstetter S. Dusky Shark/ Carcharhinus obscurus (LeSueur 1818). In: B.B. Collette BB, Klein-MacPhee G, editors, Fishes of the Gulf of Maine, 3rd ed. Washington: Smithsonian Institution Press; 2002. p. 42-44.

Castro JI. Observations on the reproductive cycles of some viviparous North American sharks. Aqua. 2009; 15(4): 205-222. Available: http://www.aqua-aquapress.com/observations-on-the-reproductive-cycles-of-some-viviparous-north-american-sharks-2/

Chin A, Kyne PM, Walker TI, McAuley RB. An integrated risk assessment for climate chang: analyzing the vulnerability of sharks and rays on Australia's Great Barrier Reef. Glob Chang Biol. 2010; 16(7): 1936–1953. doi: 10.1111/j.1365-2486.2009.02128.x

Cortés E. Demographic analysis as an aid in shark stock assessment and management. Fish Res. 1998; 39(2): 199-208. doi: 10.1016/S0165-7836(98)00183-0

Kohler NE, Casey JG, Turner PA. NMFS Cooperative Shark Tagging Program, 1962-93: An Atlas of Shark Tag and Recapture Data. Mar Fish Rev. 1998; 60(2): 1- 87. Available: http://spo.nwr.noaa.gov/mfr6021.pdf

McAuley R, Fowler A, Peddemors V. 2012. Status of key Australian fish stocks 2012, dusky shark *Carcharhinus obscurus*. Fisheries Research and Development Corporation. Available: http://www.fish.gov.au/reports/sharks/Pages/dusky_shark.aspx

McCandless CT, Kohler NE, Pratt Jr. HL, editors. Shark nursery grounds of the Gulf of Mexico and the East Coast waters of the United States. Bethesda: American Fisheries Society, Symposium 50; 2007. 402 p.

Natanson LJ, Casey JG, Kohler NE. Age and growth estimates for the dusky shark, Carcharhinus obscurus, in the western North Atlantic Ocean. Fishery Bulletin 1995; 93: 116-126. Available: http://fishbull.noaa.gov/931/natanson.pdf

NMFS. 2006. Final Consolidated Atlantic Highly Migratory Species Fishery Management Plan. National Oceanic and Atmospheric Administration, National Marine Fisheries Service, Office of Sustainable Fisheries, Highly Migratory Species Management Division, Silver Spring, MD. Available: http://www.fisheries.noaa.gov/sfa/hms/documents/fmp/index.html

Romine JG, Musick JA, Burgess GH. Demographic analyses of the dusky shark, *Carcharhinus obscurus*, in the Northwest Atlantic incorporating hooking mortality estimates and revised reproductive parameters. Environ Biol Fishes. 2009; 84(3): 277-289. doi: 10.1007/s10641-008-9435-6

Return to Table of Contents

SEDAR. 2011. Southeast Data, Assessment, and Review (SEDAR) 21. 2011. Sandbar, Dusky, and Blacknose sharks. NMFS/HMS/SEFSC. Accessed online (August 2015): http://sedarweb.org/docs/sar/Dusky_SAR.pdf

Return to Table of Contents

Eastern Oyster – *Crassostrea virginica*

Overall Vulnerability Rank = Very High

Biological Sensitivity = High
Climate Exposure = Very High
Data Quality = 88% of scores ≥ 2

Crassostrea virginica	Expert Scores	Data Quality	Expert Scores Plots (Portion by Category)
Stock Status	2.6	1.4	
Other Stressors	3.4	3.0	
Population Growth Rate	2.5	2.0	
Spawning Cycle	1.8	3.0	
Complexity in Reproduction	2.3	2.6	
Early Life History Requirements	2.6	3.0	
Sensitivity to Ocean Acidification	3.9	2.2	
Prey Specialization	2.1	2.6	
Habitat Specialization	1.8	2.6	
Sensitivity to Temperature	1.4	3.0	
Adult Mobility	4.0	3.0	
Dispersal & Early Life History	2.3	3.0	
Sensitivity Score	High		
Sea Surface Temperature	4.0	3.0	
Variability in Sea Surface Temperature	1.0	3.0	
Salinity	2.0	3.0	
Variability Salinity	1.2	3.0	
Air Temperature	4.0	3.0	
Variability Air Temperature	1.0	3.0	
Precipitation	1.3	3.0	
Variability in Precipitation	1.4	3.0	
Ocean Acidification	4.0	2.0	
Variability in Ocean Acidification	1.0	2.2	
Currents	2.0	1.0	
Sea Level Rise	2.6	1.5	
Exposure Score	Very High		
Overall Vulnerability Rank	Very High		

Low
Moderate
High
Very High

Return to Table of Contents

Eastern Oyster (*Crassostrea virginica*)

Overall Climate Vulnerability Rank: **Very High** (100% certainty from bootstrap analysis).

Climate Exposure: **Vey High**. Three exposure factors contributed to this score: Ocean Surface Temperature (4.0), Air Temperature (4.0) and Ocean Acidification (4.0). Eastern Oyster utilize near coastal habitats and have a calcium carbonate shell.

Biological Sensitivity: **High**. Three sensitivity attributes scored above 3.0: Sensitivity to Ocean Acidification (3.9), Adult Mobility (4.0) and Other Stressors (3.4). Eastern Oyster are sessile and have a calcium carbonate shell. Adults are reef building and found in estuarine habitats. They are subject to a number of other stressors including contaminants, invasive species, and habitat loss.

Distributional Vulnerability Rank: **Moderate** (93% certainty from bootstrap analysis).

Directional Effect in the Northeast U.S. Shelf: The effect of climate change on Eastern Oyster on the Northeast U.S. Shelf is very likely to be negative (>95% certainty in expert scores). Ocean acidification will likely negatively impact molluscs, including Eastern Oyster. Increased precipitation may also reduce available habitat in estuarine areas as upper estuaries become fresher.

Data Quality: 88% of the data quality scores were 2 or greater indicate that data quality is moderate.

Climate Effects on Abundance and Distribution: Shell growth of juvenile Eastern Oysters is lower under lower aragonite saturation states (Ries et al., 2009) and lower pH (Waldbusser et al., 2011). Larvae experienced lowered growth and delayed metamorphosis at higher CO_2 concentrations (Talmage and Goblet, 2009). Kimmel and Newell (2007) indicated that reduced recruitment in Chesapeake Bay was due to decreased spawning stock biomass and climate-driven changes in environmental conditions, mostly changes in precipitation and freshwater input into the Bay (Kimmel et al., 2012).

Life History Synopsis**:** Eastern Oyster is an estuarine keystone species found from the Gulf of St. Lawrence, Canada, to Key Biscayne, Florida, the Gulf of Mexico to the Yucatan Peninsula, and the West Indies to Venezuela (Sellers and Stanley, 1984). Eastern Oyster has also been introduced to Japan, Australia, Great Britain, Hawaii, and the west coast of North America (Sellers and Stanley, 1984). The species is dioecious, but most young individuals are males while older individuals are females, and there is evidence that individuals can change gender back and forth annually if needed (Sellers and Stanley, 1984; EOBRT, 2007). Spawning is stimulated by temperature, but the spawning temperature is stock-specific (Sellers and Stanley, 1984). In northern climates, spawning occurs in the summer, and can occur year round in southern climates if temperatures remain above 20°C and salinities above 10 (EOBRT, 2007). Males release gametes into the water column first, inducing females to release eggs (Sellers and Stanley, 1984). Female oysters are highly fecund, producing tens of millions of eggs, and can spawn multiple times per season (Sellers and Stanley, 1984). Eggs incubate for 4-6 hours, and larvae progress through a series of planktonic stages over a period of 2-3 weeks post-fertilization, but can delay the settlement process up to 2 months to search for suitable attachment sites (Sellers and Stanley, 1984; EOBRT, 2007). The initial larval stage, the trochophore stage, lasts 1-2 days and is non-feeding (EOBRT, 2007). The veliger stages are marked by the secretion of a shell, development of a ciliated velum for swimming and feeding on planktonic organisms (Sellers and Stanley, 1984; EOBRT, 2007). Larvae do not tolerate high temperatures and have a narrower salinity tolerance range than adults (Sellers and Stanley, 1984; EOBRT, 2007). Larvae can be quite abundant in the plankton and are food for a variety of

filter feeders (Sellers and Stanley, 1984). By the end of the veliger stage, the larvae have a foot with a byssal gland and react to changes in salinity by changing depth distribution, aiding retention within the estuary (Sellers and Stanley, 1984). At the end of the larval stage, and likely triggered by rising temperatures, young oysters drop to the bottom and begin crawling in a circular search area, seeking substrate (Sellers and Stanley, 1984). Shells seem to be the preferred substrate, but other hard surfaces or mud that can support the weight of the reef are also used (Sellers and Stanley, 1984). A current strong enough to supply food but not re-suspend sediment is necessary for survival (Sellers and Stanley, 1984; EOBRT, 2007). Once, a suitable attachment site is found, the oysters reabsorb the foot and velum and become sedentary spat (Sellers and Stanley, 1984). Settlement can be repeated until suitable habitat is found, but once metamorphosis is complete, the oyster is permanently sessile (EOBRT, 2007). Therefore, adult distribution is determined by the factors effecting larval dispersal, settlement, and survival. Oyster Drills, Channeled Whelk, starfish, crabs, and the Flat Worm, *Stylochus ellipticus*, are predators of small oysters (Sellers and Stanley, 1984). Adult oysters are sessile and usually occur in groups called beds or reefs in a variety of habitats including: oceanic bays, estuaries, drowned river mouths, and behind barrier beaches with wide ranges in temperature, salinity, and water clarity (Sellers and Stanley, 1984). The extent of Eastern Oyster distribution to the north and inland in the northern extent of their range are temperature limited, and predation and food availability also play a role in distribution and survival (EOBRT, 2007). Eastern Oysters are filter feeders of phytoplankton and suspended detritus (Sellers and Stanley, 1984). Oyster Drills and Channeled Whelks are common predators of adults (Sellers and Stanley, 1984). Easter Oyster is a keystone species in most estuaries throughout their range, providing habitat for many managed and unmanaged species and promoting species diversity, effecting water circulation patterns, and improving water quality (EOBRT, 2007). Harvesting of most natural beds in the northeastern United States is no longer cost-effective (Sellers and Stanley, 1984; EOBRT, 2007). Aquaculture is a major source of commercial oysters (EOBRT, 2007). Eastern Oysters are managed by each state in the United States and by Canadian and Caribbean governing bodies. A petition to list Eastern Oyster as endangered or threatened was denied because the long-term persistence of oyster throughout its range is not believed to be at risk in the foreseeable future (EOBRT, 2007).

Literature Cited:

Eastern Oyster Biological Review Team (EOBRT). Status review of the eastern oyster (Crassostrea virginica). Report to the National Marine Fisheries Service, Northeast Regional Office. 2007. NOAA Tech. Memo. NMFS F/SPO-88, 105 p. Available: http://www.nmfs.noaa.gov/pr/species/Status%20Reviews/eastern_oyster_sr_2007.pdf

Kimmel DG, Newell RI. The influence of climate variation on eastern oyster (*Crassostrea virginica*) juvenile abundance in Chesapeake Bay. Limnol Oceanogr. 2007; 52(3): 959-965. doi: 10.4319/lo.2007.52.3.0959

Kimmel DG, Tarnowski M, Newell RI. Long-term (1939 to 2008) spatial patterns in juvenile eastern oyster (*Crassostrea virginica*, Gmelin 1791) abundance in the Maryland portion of Chesapeake Bay. J Shell Res. 2012; 31(4), 1023-1031. doi: http://dx.doi.org/10.2983/035.031.0414

Ries JB, Cohen AL, McCorkle DC. Marine calcifiers exhibit mixed responses to CO2-induced ocean acidification. Geol. 2009; 37(12), 1131-1134. doi: 10.1130/G30210A.1

Sellers MA, Stanley JG. Species profiles: life histories and environmental requirements of coastal fishes and invertebrates (North Atlantic) – American oyster. 1984. U.S. Fish Wildl. Serv. FWS/OBS-82/11.23. U.S. Army Corps of Engineers, TR EL-82-4. 15 pp. Available: http://www.nwrc.usgs.gov/publications/specprof.htm

Talmage SC, Gobler CJ. The effects of elevated carbon dioxide concentrations on the metamorphosis, size, and survival of larval hard clams (*Mercenaria mercenaria*), bay scallops (*Argopecten irradians*), and Eastern oysters (*Crassostrea virginica*). Limnol Oceanogr. 2009; 54(6): 2072-2080. doi: 10.4319/lo.2009.54.6.2072

Waldbusser GG, Voigt EP, Bergschneider H, Green MA, Newell RI. Biocalcification in the eastern oyster (*Crassostrea virginica*) in relation to long-term trends in Chesapeake Bay pH. Estuar Coasts. 2011; 34(2): 221-231. 10.1007/s12237-010-9307-0

Return to Table of Contents

Green Sea Urchin – *Strongylocentrotus droebachiensis*

Overall Vulnerability Rank = High

Biological Sensitivity = High
Climate Exposure = High
Data Quality = 83% of scores ≥ 2

Strongylocentrotus droebachiensis	Expert Scores	Data Quality	Expert Scores Plots (Portion by Category)
Stock Status	2.5	1.2	
Other Stressors	1.8	2.1	
Population Growth Rate	2.5	1.6	
Spawning Cycle	3.4	3.0	
Complexity in Reproduction	1.4	2.5	
Early Life History Requirements	2.7	2.6	
Sensitivity to Ocean Acidification	3.3	2.6	
Prey Specialization	1.9	2.6	
Habitat Specialization	2.4	2.7	
Sensitivity to Temperature	1.7	2.8	
Adult Mobility	3.2	3.0	
Dispersal & Early Life History	1.9	2.4	
Sensitivity Score	High		
Sea Surface Temperature	3.9	3.0	
Variability in Sea Surface Temperature	1.0	3.0	
Salinity	1.1	3.0	
Variability Salinity	1.2	3.0	
Air Temperature	3.0	3.0	
Variability Air Temperature	1.0	3.0	
Precipitation	1.2	3.0	
Variability in Precipitation	1.2	3.0	
Ocean Acidification	4.0	2.0	
Variability in Ocean Acidification	1.0	2.2	
Currents	2.2	1.0	
Sea Level Rise	1.1	1.5	
Exposure Score	High		
Overall Vulnerability Rank	High		

Low
Moderate
High
Very High

Return to Table of Contents

Green Sea Urchin (*Strongylocentrotus droebachiensis*)

Overall Climate Vulnerability Rank: **High** (94% certainty from bootstrap analysis).

Climate Exposure: **High**. Three exposure factors contributed to this score: Ocean Surface Temperature (3.9), Air Temperature (3.0), and Ocean Acidification (4.0). All life stages of Green Sea Urchin use marine habitats and adults can be found in shallow waters.

Biological Sensitivity: **High**. Three sensitivity attributes scored above 3.0: Spawning Cycle (3.4), Sensitivity to Ocean Acidification (3.3), and Adult Mobility (3.2). Spawning occurs in a discrete season: early spring. Tests are made of calcium carbonate and adult mobility is limited.

Distributional Vulnerability Rank: **Moderate** (86% certainty from bootstrap analysis).

Directional Effect in the Northeast U.S. Shelf: The effect of climate change on Green Sea Urchin on the Northeast U.S. Shelf is very likely to be negative (>95% certainty in expert scores). Ocean acidification will likely negatively impact test forming echinoderms, including Green Sea Urchins. Warming will also likely reduce population productivity and may cause shifts out of the region.

Data Quality: 83% of the data quality scores were 2 or greater indicate that data quality is moderate.

Climate Effects on Abundance and Distribution: Juvenile growth and survivorship is related to temperature with maximum rates observed at 9-13°C (Pearce et al., 2005). Warming above these temperatures would result in decreased growth and survivorship thereby reducing productivity. In addition, decreasing pH (i.e., ocean acidification) negatively affects many aspects of the life cycle including larval survival and settlement success (Dupont et al., 2013). However, there is evidence of plasticity and some ability to acclimate to lower levels of pH (Dupont and Thorndyke, 2013).

Life History Synopsis**:** Green Sea Urchin is a cold water echinoderm species found in the northern Pacific, western Baltic Sea, Korean coast, and from the Canadian Arctic to New Jersey in the western north Atlantic (CCIAP, 2007). Spawning is broadcast and external and predominantly occurs in spring with a smaller season in fall in Newfoundland (Meidel and Scheibling, 1998; CCIAP, 2007). Adults in barren grounds likely contribute fewer recruits than adults from kelp beds (Meidel and Scheibling, 1998; Meidel et al., 1999). Larvae are planktonic for 4-21 weeks (CCIAP, 2007). Settlement occurs in late spring and summer when larvae attach to suitable substrate and metamorphose into benthic juveniles (Lambert and Harris, 2000; CCIAP, 2007). Metamorphosis can be delayed until suitable substrate is found, usually calcareous algae although non-calcareous algae and microbial and algal films are also used (CCIAP, 2007). Juveniles are solitary, dispersed, and not associated with adults (Nishizaki and Ackerman, 2007). Adults are the dominant herbivore in shallow rocky subtidal zones of the northwest Atlantic, and also occur in sheltered and exposed kelp beds out to 1200 m (Meidel et al., 1999; CCIAP, 2007). Green Sea Urchins are cryptic and usually sparsely distributed in crevices and under boulders (Meidel and Scheibling, 1998). Green Sea Urchin consume detritus and drift algae when sparse, but when numbers increase, adults aggregate into fronts along the edge of kelp beds (Meidel et al., 1999). Population outbreaks lead to destruction of kelp beds and formation of sea urchin-dominated barren grounds (Meidel and Scheibling, 1998). Green Sea Urchin predominately consume perennial phaeophytes, but coralline algae and animals such as barnacles, gastropods, small shrimps, and occasionally dead fish are consumed in smaller amounts (Himmelman and Steele, 1971). American Lobsters, Rock Crabs, Purple Sea Stars, other urchins, and a variety of fishes and birds are common predators of the Green Sea Urchin

(Himmelman and Steele, 1971). Green Sea Urchins are prized for their roe, particularly in Japanese markets. The U.S. fishery, which predominantly occurs in Maine and Massachusetts, is managed on a state-by-state basis.

Literature Cited:

Climate Change Impacts and Adaptation Program (CCIAP). Climate Change and Thermal Sensitivity of Canadian Atlantic Commercial Marine Species. 2007. Natural Resources Canada Project A515. Available: http://www.geog.mcgill.ca/climatechange/ReportsMap/sea_urchinRpt.pdf

Dupont S. Thorndyke M. Direct impacts of near-future ocean acidification on sea urchins. In Fernández-Palacios J. M., de Nascimento L., Hernández J. C., Clemente S., González A. & Díaz-González J. P., Climate change perspective from the Atlantic: past, present and future. 2013. Servicio de Publicaciones, Universidad de La Laguna. pp. 461-485.

Dupont S, Dorey N, Stumpp M, Melzner F, Thorndyke M. Long-term and trans-life-cycle effects of exposure to ocean acidification in the green sea urchin *Strongylocentrotus droebachiensis*. Mar. Bio. 2013; 160(8): 1835-1843. doi: 10.1007/s00227-012-1921-x

Himmelman JH, Steele DH. Foods and predators of the green sea urchin *Strongylocentroltus droebachiensis* in Newfoundland waters. Mar Biol. 1971; 9: 315-322. doi: 10.1007/BF00372825

Lambert DM, Harris LG. Larval settlement of the green sea urchin, Strongylocentrotus droebachiensis, in the southern Gulf of Maine. Invertebr Biol 2000; 119: 403-409. doi: 10.1111/j.1744-7410.2000.tb00110.x

Mann KH. Destruction of kelp beds by sea urchin: a cyclical phenomenon or irreversible degradation? Helgolander wiss Meeresunters. 1977; 30: 455-467.

Meidel SK, Scheibling RE, Metaxas A. Relative importance of parental and larval nutrition on larval development and metamorphosis of the sea urchin *Strongylocentrotus droebachiensis.* J Exper Mar Bio Ecol. 1999; 240: 161-178. doi:10.1016/S0022-0981(99)00046-5

Nishizaki MT, Ackerman JD. Juvenile-adults associations in sea urchins (*Strongylocentrotus franciscanus* and *S. droebachiensis*): protection from predation and hydrodynamics in *S. franciscanus*. Mar Biol. 2007; 151: 135-145.doi: 10.1007/s00227-006-0462-6

Pearce CM, Williams SW, Yuan F, Castell JD, Robinson S. Effect of temperature on somatic growth and survivorship of early post-settled green sea urchins, *Strongylocentrotus droebachiensis* (Müller). Aquacult Res. 2005; 36(6): 600-609. doi: 10.1111/j.1365-2109.2005.01264.x

Wharton WG, Mann KH. Relationship between destructive grazing by the sea urchin, *Strongylocentrotus droebachiensis,* and the abundance of American Lobster in the Atlantic coast of Nova Scotia. Can J Fish Aquat Sci. 1981; 38: 1339-1349. doi: 10.1139/f81-180

XChen Y, Hunter M. Assessing the green sea urchin (Strongylocentrotus drobachiensis) stock in Maine, USA. Fish Res 2003; 60: 527-537

Return to Table of Contents

Haddock – *Melanogrammus aeglefinus*

Overall Vulnerability Rank = Low

Biological Sensitivity = Low
Climate Exposure = High
Data Quality = 83% of scores ≥ 2

Melanogrammus aeglefinus	Expert Scores	Data Quality	Expert Scores Plots (Portion by Category)
Stock Status	2.3	2.8	
Other Stressors	1.3	1.6	
Population Growth Rate	1.8	2.6	
Spawning Cycle	2.4	3.0	
Complexity in Reproduction	1.9	2.8	
Early Life History Requirements	2.5	2.8	
Sensitivity to Ocean Acidification	1.1	1.4	
Prey Specialization	1.4	3.0	
Habitat Specialization	2.0	3.0	
Sensitivity to Temperature	2.4	3.0	
Adult Mobility	2.1	2.8	
Dispersal & Early Life History	1.6	3.0	
Sensitivity Score	Low		
Sea Surface Temperature	3.9	3.0	
Variability in Sea Surface Temperature	1.0	3.0	
Salinity	1.4	3.0	
Variability Salinity	1.2	3.0	
Air Temperature	1.0	3.0	
Variability Air Temperature	1.0	3.0	
Precipitation	1.0	3.0	
Variability in Precipitation	1.0	3.0	
Ocean Acidification	4.0	2.0	
Variability in Ocean Acidification	1.0	2.2	
Currents	2.1	1.0	
Sea Level Rise	1.1	1.5	
Exposure Score	High		
Overall Vulnerability Rank	Low		

Low, Moderate, High, Very High

Haddock (*Melanogrammus aeglefinus*)

Overall Climate Vulnerability Rank: **Low** (73% certainty from bootstrap analysis).

Climate Exposure: **High**. Two exposure factors contributed to this score: Ocean Surface Temperature (3.9) and Ocean Acidification (4.0). All life stages of Haddock use marine habitats.

Biological Sensitivity: **Low**. No sensitivity attributes scored above 2.5.

Distributional Vulnerability Rank: **High** (73% certainty from bootstrap analysis). Haddock are habitat generalists, with moderate mobility, and dispersive early life history stages.

Directional Effect in the Northeast U.S. Shelf: The effect of climate change on Haddock on the Northeast U.S. Shelf is estimated to be negative, but this estimate is uncertain (66-90% certainty in expert scores). Haddock is a cold-temperate species and warming will likely cause reductions in available habitat. However, in recent years Haddock recruitment has been very high despite warming. Further, Haddock distribution has remained relatively constant even with temperature increases.

Data Quality: 83% of the data quality scores were 2 or greater indicate that data quality is moderate.

Climate Effects on Abundance and Distribution: Haddock productivity has been linked to a number of environmental factors including larval retention, larval feeding conditions, and adult conditions (Lough et al., 2006; Platt et al., 2003; Friedland et al., 2008). However, these environmental factors have not be directly linked with climate factors. Haddock distribution has remained relatively stable over the past several decades (Nye et al., 2007) and Murawski (1993) did not find a link between Haddock distribution and temperature. Thus, climate effects of Haddock productivity and distribution are not as clear as they are for other groundfish species in the Northeast U.S. Shelf Ecosystem.

Life History Synopsis**:** Haddock are a typical marine species living their entire life cycle in the ocean between the Strait of Belle Isle to Cape May, New Jersey, along the east coast of North America, along both coasts of southern Greenland, and from the Barents Sea and Iceland to the Bay of Biscay along the west coast of Europe (Brodziak, 2005; Fahay, 2007). Haddock mature at around 3 years (NEFSC, 2012). The location of spawning is limited by bottom type and primarily occurs on Georges Bank and the Gulf of Maine in United States waters (Cargnelli et al., 1999; Brodziak, 2005). Spawning occurs from January to July varying latitudinally and controlled by temperature (later in colder autumn/winter; Page and Frank, 1989; Cargnelli et al., 1999; Klein-MacPhee, 2002; Brodziak, 2005). Eggs hatch within a month (about 15 days at typical Georges Bank temperatures; Page and Frank, 1989; Klein-MacPhee, 2002). Larvae are pelagic (usually found within the thermocline) and forage on less motile prey such as invertebrate eggs, copepods, and phytoplankton (Klein-MacPhee, 2002; Brodziak, 2005). Metamorphosis occurs approximately a month after hatching, but juveniles remain in the epipelagic zone for 3-5 months before settling to the bottom (Cargnelli et al., 1999; Brodziak, 2005; Fahay, 2007). Juveniles prey on abundant organisms within a specific size range: copepods and pteropods while pelagic, but switching to ophiuroids and polychaetes during the transition to demersal habitat (Cargnelli et al., 1999; Brodziak, 2005). Once demersal, juveniles and adults occupy similar habitat: primarily cool, shelf water with gravel, pebble, clay, and smooth hard sand substrate, particularly smooth areas between rocky patches (Klein-MacPhee, 2002). Benthic juveniles primarily feed on benthic prey such as crustaceans, polychaetes, and small fish (Klein-MacPhee, 2002; Brodziak, 2005). Adults are indiscriminate consumers of benthic organisms such as echinoderms, polychaetes, Atlantic Herring eggs, small fish, and

crustaceans (Klein-MacPhee, 2002; Brodziak, 2005). Haddock are occasional prey for several species of fish, including larger Haddock, and gray seals (Klein-MacPhee, 2002). Haddock populations had declined and growth and maturation had shifted since the 1960s through the early 2000s (Klein-MacPhee, 2002; NEFSC, 2012). However, Haddock appear to be responding to reduced fishing mortality and recent management strategies, both United States stocks are no longer experiencing overfishing nor are they overfished. Haddock are managed under the Northeast Multispecies Fishery Management Plan (NEFSC, 2012).

Literature Cited:

Brodziak J. Essential Fish Habitat Source Document: Haddock, Melanogrammus aeglefinus, life history and habitat characteristics. NOAA Tech. Memo. 2005; NMFS-NE-196: 64p. Accessed online (August 2014): http://www.nefsc.noaa.gov/nefsc/publications/tm/tm196/tm196.pdf

Cargnelli LM, Griesbach SJ, Berrien PL, Morse WW, Johnson DL. Essential Fish Habitat Source Document: Haddock, *Melanogrammus aeglefinus*, life history and habitat characteristics. NOAA Tech. Memo. 1999; NMFS-NE-128: 40p. Accessed online (August 2015): http://www.nefsc.noaa.gov/nefsc/publications/tm/tm128/tm128.pdf

Fahay MP. 2007. Early Stages of Fishes in the western North Atlantic Ocean (Davis Strait, Southern Greenland and Flemish Cap to Cape Hatteras). Accessed Online (August 2015): http://www.nafo.int/publications/fahay/pdfs.html

Friedland KD, Hare JA, Wood GB, Col LA, Buckley LJ, Mountain DG, et al. Does the fall phytoplankton bloom control recruitment of Georges Bank haddock, Melanogrammus aeglefinus, through parental condition? Can J Fish Aquat Sci. 2008; 65(6): 1076-1086. DOI: 10.1139/F08-040

Lough RG, Hannah CG, Berrien P, Brickman D, Loder JW, Quinlan JA. Spawning pattern variability and its effect on retention, larval growth and recruitment in Georges Bank cod and haddock. Mar Ecol Prog Ser. 2006; 310: 193-212. DOI: 10.3354/meps310193

Klein-MacPhee G. Haddock/ *Melanogrammus aeglefinus* Linnaeus 1758. In: Collette BB, Klein-MacPhee G, editors. Bigelow and Schroeder's fishes of the Gulf of Maine. 3rd Edition. Washington, DC: Smithsonian Institution Press. 2002; p.235-242.

Northeast Fisheries Science Center (NEFSC). 2012. Assessment or Data Updates of 13 Northeast Groundfish Stocks through 2010. US Dept Commer, Northeast Fish Sci Cent Ref Doc. 2012; 12-06: 789 p. Accessed online (May 2014): http://nefsc.noaa.gov/publications/crd/crd1206/

Page FH., Frank KT. Spawning time and egg stage duration in northwest Atlantic haddock (*Melanogrammus aeglefinus*) stocks with emphasis on Georges and Browns Bank. Can J Fish Aquat Sci. 1989; 46 (Suppl. No. 1): 68-81. DOI: 10.1139/f89-279

Platt T, Fuentes-Yaco C, Frank KT. Marine ecology: spring algal bloom and larval fish survival. Nature, 2003; 423(6938): 398-399. DOI: 10.1038/423398b

Nye JA, Link JS, Hare JA, Overholtz WJ. Changing spatial distribution of fish stocks in relation to climate and population size on the Northeast United States continental shelf. Mar Ecol Prog Ser. 2009; 393: 111-129. DOI: 0.3354/meps08220

Return to Table of Contents

Hickory Shad – *Alosa mediocris*

Overall Vulnerability Rank = Very High

Biological Sensitivity = High
Climate Exposure = Very High
Data Quality = 62% of scores ≥ 2

Alosa mediocris	Expert Scores	Data Quality	Expert Scores Plots (Portion by Category)
Stock Status	2.9	0.4	
Other Stressors	3.6	1.6	
Population Growth Rate	2.3	1.0	
Spawning Cycle	3.7	2.4	
Complexity in Reproduction	3.4	1.8	
Early Life History Requirements	3.4	2.0	
Sensitivity to Ocean Acidification	1.3	1.8	
Prey Specialization	1.7	1.8	
Habitat Specialization	2.8	2.2	
Sensitivity to Temperature	2.5	2.0	
Adult Mobility	1.3	2.8	
Dispersal & Early Life History	3.0	1.4	
Sensitivity Score	High		
Sea Surface Temperature	4.0	3.0	
Variability in Sea Surface Temperature	1.0	3.0	
Salinity	1.9	3.0	
Variability Salinity	1.2	3.0	
Air Temperature	4.0	3.0	
Variability Air Temperature	1.0	3.0	
Precipitation	1.3	3.0	
Variability in Precipitation	1.4	3.0	
Ocean Acidification	4.0	2.0	
Variability in Ocean Acidification	1.0	2.2	
Currents	2.0	1.0	
Sea Level Rise	2.4	1.5	
Exposure Score	Very High		
Overall Vulnerability Rank	Very High		

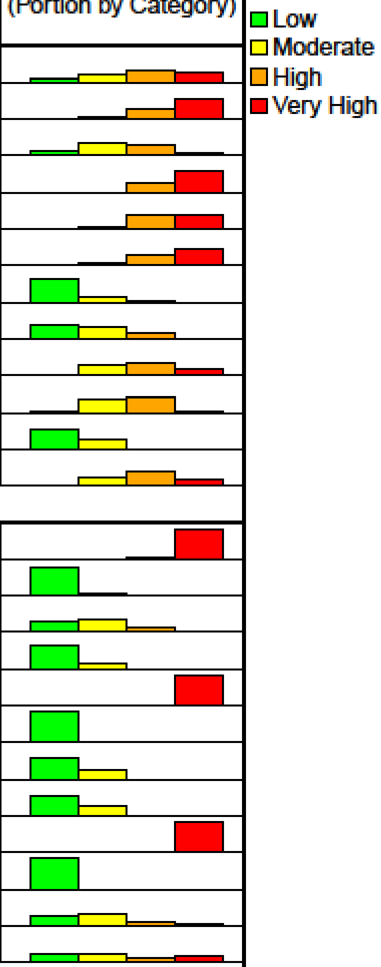

Return to Table of Contents

Hickory Shad (*Alosa mediocris*)

Overall Climate Vulnerability Rank: **Moderate** (100% certainty from bootstrap analysis).

Climate Exposure: **Very High**. Three exposure factors contributed to this score: Ocean Surface Temperature (4.0), Ocean Acidification (4.0) and Air Temperature (4.0). Hickory Shad are anadromous, spawning in freshwater, developing in freshwater and estuarine habitats, feeding as adults in marine habitats.

Biological Sensitivity: **High**. Five sensitivity attributes scored above 3.0: Spawning Cycle (3.7), Other Stressors (3.6), Complexity in Reproduction (3.4), Early Life Stage Requirements (3.4), and Dispersal and Early Life History (3.0). Hickory Shad are diadromous and exposed to a number of other stressors including habitat destruction, blockage to spawning habitats, and contaminants (Limburg and Waldman, 2009). Spawning time varies latitudinally and is linked to spring warming (Monroe, 2002). Eggs and larvae inhabit freshwaters and then juveniles move to estuarine and ocean waters. Dispersal of eggs and larvae is limited.

Distributional Vulnerability Rank: **Moderate** (56% certainty from bootstrap analysis). Hickory Shad have a relatively high degree of spawning site fidelity, limiting the ability of the species to shift distribution.

Directional Effect in the Northeast U.S. Shelf: The effect of climate change on Blueback Herring is estimated to be neutral, but this estimate has a high degree of uncertainty (<66% certainty in expert scores). Of the alosiids species along the U.S. East Coast, Hickory Shad is the least well known. Warming temperatures may impact various aspects of the life cycle, but the direction and magnitude of these impacts is difficult to determine. The effect of ocean acidification over the next 30 years is likely to be minimal.

Data Quality: 62% of the data quality scores were 2 or greater indicate that data quality is moderate.

Climate Effects on Abundance and Distribution: Relative to other alosiids, little is known regarding the effects of climate change and variability on Hickory Shad. Spawning migration timing has been linked to water temperature as well as size, age, and energy reserves (Murauskas and Rulifson, 2011). Egress to saltwater in the fall is also linked temperature (Able et al., 2014). Studies on other alosiids in the region (Alewife, Blueback Herring) indicate that climate will impact Hickory Shad productivity. Distribution will be affected less, owing to spawning site fidelity (NMFS, 2012; Lynch et al., 2014; Tommasi et al., 2015).

Life History Synopsis**:** Hickory Shad is an anadromous, iteroperous, coastal species found from the Gulf of Maine to St. John's River, Florida (Munroe, 2002). Most Hickory Shad are mature by age 3-5 years (Munroe, 2002). Mature adults migrate to freshwater around Chesapeake Bay and to the south in spring to spawn (Munroe, 2002). Little is know about the specific location of spawning, but Hickory Shad seem to prefer freshwater streams with some current (Munroe, 2002; Able and Fahay, 2010). Spent adults return to sea (Able and Fahay, 2010). Eggs are slightly adhesive and semi-demersal (Munroe, 2002). Larvae and small juveniles leave the freshwater spawning areas for tidal-freshwater and estuarine nursery areas (Able and Fahay, 2010). Young shad migrate from the nursery areas to the ocean in early summer (Able and Fahay, 2010). Little is known about the oceanic part of the Hickory Shad life cycle. Adult shad occur in estuarine and coastal areas, occasionally ranging as far north as the Gulf of Maine in fall, but probably overwinter in North and South Carolina (Munroe, 2002; Able and Fahay, 2010). Hickory Shad are more piscivorous than their congeners and in addition to squid, fish eggs,

and small crabs, have been know to consume Sand Lance, anchovies, Cunner, Atlantic Herring, Scup, and Atlantic Silversides (Munroe, 2002). The Atlantic States Marine Fisheries Commission manages Hickory Shad through the Interstate Fishery Management Plan for Shad and River Herring (ASMFC, 1999).

Literature Cited:

Able KW, Grothues TM, Turnure JT, Malone MA, Henkes GA. Dynamics of residency and egress in selected estuarine fishes: evidence from acoustic telemetry. Environ Biol Fishes. 2014; 97(1): 91-102. doi: 10.1007/s10641-013-0126-6

Able KW, Fahay MP. 2010. Ecology of estuarine fishes: temperate waters of the western North Atlantic. Baltimore: The Johns Hopkins University Press; 2010. 566p.

Atlantic States Marine Fisheries Commission (ASMFC). 1999. Amendment 1 to the Interstate Fishery Management Plan for Shad and River Herring. Fishery Management Report No. 35. 77 p. Accessed online (October 2014): http://www.asmfc.org/uploads/file/shadam1.pdf

Limburg KE, Waldman JR. Dramatic declines in North Atlantic diadromous fishes. BioScience. 2009; 59(11): 955-965. doi: 10.1525/bio.2009.59.11.7

Lynch, P. D., Nye, J. A., Hare, J. A., Stock, C. A., Alexander, M. A., Scott, J. D., et al. Projected ocean warming creates a conservation challenge for river herring populations. ICES J Mar Sci. 2015; 72(2): 374-387. doi: 10.1093/icesjms/fsu134

Munroe T. Hickory Shad/ Alosa mediocris (Mitchill 1814). In: B.B. Collette BB, Klein-MacPhee G, editors, Fishes of the Gulf of Maine, 3rd ed. Washington: Smithsonian Institution Press; 2002. pp. 116-118.

Murauskas JG, Rulifson RA. Reproductive development and related observations during the spawning migration of Hickory Shad. Trans Am Fish Soc. 2011; 140(4), 1035-1048. doi: 10.1080/00028487.2011.607036

National Marine Fisheries Service (NMFS). 2012. River Herring Climate Change Workshop Report. Greater Atlantic Fishery Regional Office, Gloucester, Massachusetts, 60p. http://www.greateratlantic.fisheries.noaa.gov/prot_res/CandidateSpeciesProgram/sswpdocs/RIVER%20HERRING%20CLIMATE%20CHANGE%20WORKSHOP%20REPORT_122712.pdf

Tommasi D, Nye J, Stock C, Hare JA, Alexander M, Drew K. Effect of Environmental Conditions on Juvenile Recruitment of Alewife (Alosa pseudoharengus) and Blueback Herring (A. aestivalis) in fresh water: A Coastwide Perspective. Can J Fish Aquat Sci. 2015; 72(7): 1037-1047. doi: 10.1139/cjfas-2014-0259

[Return to Table of Contents]

Horseshoe Crab – *Limulus polyphemus*

Overall Vulnerability Rank = Very High

Biological Sensitivity = High
Climate Exposure = Very High
Data Quality = 92% of scores ≥ 2

Limulus polyphemus	Expert Scores	Data Quality	Expert Scores Plots (Portion by Category)
Stock Status	2.0	2.8	
Other Stressors	2.4	2.2	
Population Growth Rate	3.4	2.6	
Spawning Cycle	2.8	3.0	
Complexity in Reproduction	3.1	2.8	
Early Life History Requirements	2.0	3.0	
Sensitivity to Ocean Acidification	2.1	2.6	
Prey Specialization	2.2	2.4	
Habitat Specialization	2.3	2.8	
Sensitivity to Temperature	2.1	3.0	
Adult Mobility	2.6	2.8	
Dispersal & Early Life History	2.9	3.0	
Sensitivity Score	High		
Sea Surface Temperature	3.9	3.0	
Variability in Sea Surface Temperature	1.0	3.0	
Salinity	2.4	3.0	
Variability Salinity	1.2	3.0	
Air Temperature	3.1	3.0	
Variability Air Temperature	1.0	3.0	
Precipitation	1.3	3.0	
Variability in Precipitation	1.4	3.0	
Ocean Acidification	4.0	2.0	
Variability in Ocean Acidification	1.0	2.2	
Currents	2.0	1.0	
Sea Level Rise	3.6	1.5	
Exposure Score	Very High		
Overall Vulnerability Rank	Very High		

Low
Moderate
High
Very High

Horseshoe Crab (*Limulus polyphemus*)

Overall Climate Vulnerability Rank: **Very High** (83% certainty from bootstrap analysis).

Climate Exposure: **Very High**. Three exposure factors contributed to this score: Ocean Surface Temperature (3.9), Ocean Acidification (4.0) and Sea Level Rise (3.6). Horseshoe Crabs use marine habitats, but spawn in intertidal areas that could be impacted by sea-level rise.

Biological Sensitivity: **High**. Two sensitivity attributes scored above 3.0: Population Growth Rate (3.4) and Complexity in Reproduction (3.1). Horseshoe Crabs are slow growing and long-lived. Their life cycle includes spawning on specific beaches on a lunar cycle.

Distributional Vulnerability Rank: **Low** (83% certainty from bootstrap analysis).

Directional Effect in the Northeast U.S. Shelf: The effect of climate change on Horseshoe Crab is likely to be negative (90-95% certainty in expert scores). Sea-level rise will reduce spawning habitat. Warming will negatively impact egg and larval survival and thereby reduce productivity. There is little information available regarding the effect of ocean acidification.

Data Quality: 92% of the data quality scores were 2 or greater indicate that data quality is moderate.

Climate Effects on Abundance and Distribution: Growth and larval development of larval Horseshoe Crab is temperature dependent, with maximum growth and development occurring at 30°C (Laughlin, 1983). Vasquez et al. (2015) showed that the interaction of multiple stressors was important in determining the embryo development rate. Changes in temperature and salinity in the nearshore habitats could affect larval survival and ultimately recruitment and population productivity. Sea-level rise also poses a threat, and Galbraith et al. (2002) projected losses of intertidal habitat in Delaware Bay, which represents an important spawning area for Horseshoe Crabs. The effects of beach erosion and loss of spawning habitat may be more important to Horseshoe Crab dynamics than climate-related changes in temperature and salinity (Botton and Itow, 2009).

Life History Synopsis**:** Horseshoe Crab is a marine arthropod more closely related to spiders than to true crabs. Horseshoe Crabs can be found from northern Maine to the Yucatan Peninsula (ASMFC, 1998). Maturity is reached in 9-11 years (ASMFC, 1998). Adults migrate to protected, low-energy, intertidal sandy beaches between March and August to spawn (ASMFC, 1998). Multiple batch spawns occur during the season with peaks surrounding the full and new moons (ASMFC, 1998). Horseshoe Crabs are highly fecund with high egg and larval mortality (ASMFC, 1998). Eggs are deposited about 10 cm deep in moist sand nests at or near the water line (Laughlin, 1983; ASMFC, 1998), but waves or burrowing activity can wash eggs to the surface leaving them vulnerable to predation (ASMFC, 1998). Eggs hatch 2 -4 weeks post-fertilization depending on temperature, moisture, and oxygen content of the nest environment, but larvae can delay emergence to overwinter in the beach sediments until spring (ASMFC, 1998). Eggs are an important food source for shorebirds (ASMFC, 1998). The first larval stage is called the trilobite and is non-feeding and yolk-dependent until first molt into the first tailed stage (Laughlin, 1983). Trilobites remain close to shore and settle in shallow water to molt (ASMFC, 1998). Larvae eat polychaetes and nematodes and are prey to shorebirds and a variety of invertebrates and finfish including sharks (ASMFC, 1998). Juveniles spend the first two years on sand flats and in shallow waters of bays near the spawning beaches (ASMFC, 1998). Older juveniles either stay in coastal bays year-round or migrate to subtidal areas a few kilometers offshore with the adults of the population

(ASMFC, 1998). Horseshoe Crabs must molt to grow, and molt 16-17 times over the 9-11 years spent as a juvenile, molting more frequently during the early years (ASMFC, 1998). Adult Horseshoe Crabs are able to survive a wide range of conditions, but require salinities >7 and are generally found in 20 m or less water depths (although they have been found well offshore; ASMFC, 1998). Juveniles and adults consume a variety of molluscs, mainly bivalves, and benthic invertebrates such as arthropods, annelids, nemertean worms, and some vascular plants (ASMFC, 1998). Predation on adults of the species is likely low, but sea turtles and marine mammals may prey on Horseshoe Crabs, and the crabs are the preferred bait for American Eel, Channeled Whelk, and Knobbed Whelk fisheries (ASMFC, 1998). Adults are found in bay areas near spawning beaches during most of the year and can either remain there all winter or migrate in the fall to the continental shelf (ASMFC, 1998). Horseshoe Crabs are the primary bait for several commercial fisheries, and an important component of the biomedical industry, thus overfishing is a concern (ASMFC, 2013). Management of the Atlantic stock, from Maine to eastern Florida (with many subpopulations) is controlled by the Atlantic States Marine Fisheries Commission, but administered on state-by-state, multistate, and embayment levels (ASMFC, 2013). The Delaware Bay and southeast populations are stabilized and possibly rebuilding, but the New York and New England populations have declined (ASMFC, 2013). A ban on the import of Asian Horseshoe Crabs has been recommended to reduce importation of parasites and diseases (ASMFC, 2013).

Literature Cited:

Atlantic States Marine Fisheries Commission (ASMFC). Interstate Fishery Management Plan for Horseshoe Crab. Fishery Management Report No. 32 of the Atlantic States Marine Fisheries Commission. 1998. Accessed Online (August 2015): http://www.asmfc.org/uploads/file/hscFMP.pdf

Atlantic States Marine Fisheries Commission (ASMFC). 2013 Horseshoe Crab stock assessment update. 2013. Accessed online (August 2015): http://www.asmfc.org/uploads/file//52a88db82013HSC_StockAssessmentUpdate.pdf

Botton ML, Itow T. The effects of water quality on Horseshoe Crab embryos and larvae. In Biology and Conservation of Horseshoe Crabs 2009; 439-454. Springer US.

Galbraith H, Jones R, Park R, Clough J, Herrod-Julius S, Harrington B, Page G. Global climate change and sea level rise: potential losses of intertidal habitat for shorebirds. Waterbirds. 2002; 25(2): 173-183. DOI: http://dx.doi.org/10.1675/1524-4695(2002)025[0173:GCCASL]2.0.CO;2

Laughlin R. The effects of temperature and salinity on larval growth of the Horseshoe Crab *Limulus polyphemus*. Biol Bull. 1983; 164(1): 93-103. Accessed Online (August 2015): http://www.biolbull.org/content/164/1/93.abstract?sid=18594fa0-13a0-4c3a-8459-be9ccd368aa4

Vasquez MC, Murillo A, Brockmann HJ, Julian D. Multiple stressor interactions influence embryo development rate in the American Horseshoe Crab, *Limulus polyphemus*. J Exp Biol. 2015; jeb-117184. DOI: 10.1242/jeb.117184

Return to Table of Contents

Knobbed Whelk – *Busycon carica*

Overall Vulnerability Rank = Very High

Biological Sensitivity = High
Climate Exposure = Very High
Data Quality = 79% of scores ≥ 2

Busycon carica	Expert Scores	Data Quality	Expert Scores Plots (Portion by Category)
Stock Status	2.4	0.2	
Other Stressors	2.3	1.5	
Population Growth Rate	2.2	1.6	
Spawning Cycle	2.2	2.9	
Complexity in Reproduction	1.9	2.3	
Early Life History Requirements	1.5	2.8	
Sensitivity to Ocean Acidification	3.9	2.6	
Prey Specialization	2.4	2.7	
Habitat Specialization	1.9	2.5	
Sensitivity to Temperature	1.7	2.8	
Adult Mobility	3.0	2.8	
Dispersal & Early Life History	3.6	3.0	
Sensitivity Score	High		
Sea Surface Temperature	3.9	3.0	
Variability in Sea Surface Temperature	1.0	3.0	
Salinity	1.6	3.0	
Variability Salinity	1.2	3.0	
Air Temperature	3.5	3.0	
Variability Air Temperature	1.0	3.0	
Precipitation	1.3	3.0	
Variability in Precipitation	1.3	3.0	
Ocean Acidification	4.0	2.0	
Variability in Ocean Acidification	1.0	2.2	
Currents	2.0	1.0	
Sea Level Rise	1.6	1.5	
Exposure Score	Very High		
Overall Vulnerability Rank	Very High		

Low
Moderate
High
Very High

Knobbed Whelk (*Busycon carica*)

Overall Climate Vulnerability Rank: **Very High** (58% certainty from bootstrap analysis).

Climate Exposure: **Very High**. Three exposure factors contributed to this score: Ocean Surface Temperature (3.9), Air Temperature (3.5) and Ocean Acidification (4.0). Knobbed Whelk are found in inner shelf and nearshore waters.

Biological Sensitivity: **High**. Three sensitivity attributes scored above 3.0: Sensitivity to Ocean Acidification (3.9), Adult Mobility (3.0), and Dispersal and Early Life History (3.6). Adults have a calcium carbonate shell and low mobility. Eggs are attached to the bottom in a gelatinous egg sack and dispersal of larvae is believed to be limited.

Distributional Vulnerability Rank: **High** (100% certainty from bootstrap analysis).

Directional Effect in the Northeast U.S. Shelf: The effect of climate change on Knobbed Whelk on the Northeast U.S. Shelf is very likely to be negative (>95% certainty in expert scores). Ocean acidification will likely negatively impact molluscs, including Knobbed Whelk. Although there is relatively little information, warming has the potential to cause northward distribution shifts and may reduce productivity in the southern part of the ecosystem.

Data Quality: 79% of the data quality scores were 2 or greater indicate that data quality is moderate.

Climate Effects on Abundance and Distribution: There is relatively little information regarding the population dynamics and climate effects on productivity and distribution of Knobbed Whelk. Ries et al. (2009) found that calcification rate of other gastropods decreased with decreasing aragonite saturation state. Thus ocean acidification may reduce population productivity, but specific experiments on knobbed whelk are needed.

Life History Synopsis**:** Knobbed Whelk is a slow-growing, estuarine and marine shellfish that occurs from Cape Cod, Massachusetts, to Cape Canaveral, Florida (Eversole et al., 2008). The species was once believed to be hermaphroditic, but recent work and genetics have confirmed that Knobbed Whelks are dioecious with larger and later maturing females (6 years) and smaller, earlier maturing males (4 years) (Avise et al., 2004; Power et al., 2009). Maturity may occur later in northern parts of their range (Castagna and Kraeuter, 1994; Power et al., 2009). Spawning occurs in spring and fall, but it is uncertain whether an individual female can spawn twice a year, and fall is the dominant spawning season (Magalhaes, 1948). Knobbed Whelk form small spawning aggregations and fertilization occurs internally (Magalhaes, 1948). A female produces thousands of eggs that are encased in capsules and laid in a long string with one end buried in the mud or sand as an anchor (Magalhaes, 1948). Eggs usually incubate over the winter and hatch in the spring, but incubation can be as short as 6 weeks (Power et al., 2009) or as long as 13 months (Anderson, 2005). Young Knobbed Whelks emerge from the eggs as small juveniles (Magalhaes, 1948). Knobbed Whelk are an abundant species of whelk throughout most of their range, particularly in inshore and shelf mud and sand bottom habitat (Magalhaes, 1948). During late fall and winter, Knobbed Whelks partially submerge themselves in the sand in deeper waters (Magalhaes, 1948; Power et al., 2009). Adults prey primarily on bivalves, but annelids are also consumed and Knobbed Whelk may scavenge on recently dead fish (Magalhaes, 1948). Crabs and gulls are common predators (Magalhaes, 1948). Knobbed Whelks are long-lived, late maturing, and slow growing (Eversole et al.,

2008). Commercial fisheries exist throughout their range and are managed on a state-by-state basis. Knobbed Whelks can be hand-picked, dredged, or occasionally caught in traps (Magalhaes, 1948).

Literature Cited:

Anderson WD. *Busycon carica*. Comprehensive Wildlife Conservation Strategy; SC Department of Natural Resources. 2005. Accessed Online (August 2015): http://www.dnr.sc.gov/cwcs/pdf/Knobbedwhelk.pdf

Avise JC, Power AJ, Walker D. Genetic sex determination, gender identification and pseudohermaphroditism in the knobbed whelk, *Busycon carica* (Mollusca: Melongenidae). Roy Soc Proc B. 2004; 271: 641-646. DOI: 10.1098/rspb.2003.2533

Castagna M, Kraeuter JN. Age, growth rate, sexual dimorphism and fecundity of Knobbed Whelk *Busycon carica* (Gmelin, 1791) in a western mid-Atlantic lagoon system, Virginia. J Shell Res. 1994; 13: 581-585.

Eversole AG, Anderson WD, Isely JJ. Age and growth of the knobbed whelk *Busycon carica* (Gmelin 1791) in South Carolina subtidal waters. J Shell Res. 2008; 27(2): 423-426. DOI: http://dx.doi.org/10.2983/0730-8000(2008)27[423:AAGOTK]2.0.CO;2

Magalhaes H. An ecological study of snails of the genus *Busycon* at Beaufort, North Carolina. Ecol Monogr. 1948; 18: 379-409. DOI: http://dx.doi.org/10.2307/1948577

Power AJ, Sellers CJ, Walker RL. Growth and sexual maturity of the Knobbed Whelk, *Busycon carica* (Gmelin, 1791), from a commercially harvested population in coastal Georgia. Occasional Papers of the University of Georgia Marine Extension Service, 2009; 4: 1-29. Accessed online (August 2015): http://georgiaseagrant.uga.edu/images/uploads/media/Knobbed_Whelk.pdf

[Return to Table of Contents](#)

Little Skate – *Leucoraja erinacea*

Overall Vulnerability Rank = Low

Biological Sensitivity = Low
Climate Exposure = High
Data Quality = 88% of scores ≥ 2

Leucoraja erinacea	Expert Scores	Data Quality	Expert Scores Plots (Portion by Category)
Stock Status	2.0	2.8	
Other Stressors	1.5	1.4	
Population Growth Rate	2.9	2.4	
Spawning Cycle	1.2	3.0	
Complexity in Reproduction	1.3	2.2	
Early Life History Requirements	1.1	3.0	
Sensitivity to Ocean Acidification	1.5	2.8	
Prey Specialization	1.2	3.0	
Habitat Specialization	1.2	3.0	
Sensitivity to Temperature	2.1	3.0	
Adult Mobility	2.3	2.2	
Dispersal & Early Life History	1.9	2.8	
Sensitivity Score	Low		
Sea Surface Temperature	3.9	3.0	
Variability in Sea Surface Temperature	1.0	3.0	
Salinity	2.0	3.0	
Variability Salinity	1.2	3.0	
Air Temperature	1.0	3.0	
Variability Air Temperature	1.0	3.0	
Precipitation	1.0	3.0	
Variability in Precipitation	1.0	3.0	
Ocean Acidification	4.0	2.0	
Variability in Ocean Acidification	1.0	2.2	
Currents	2.1	1.0	
Sea Level Rise	1.1	1.5	
Exposure Score	High		
Overall Vulnerability Rank	Low		

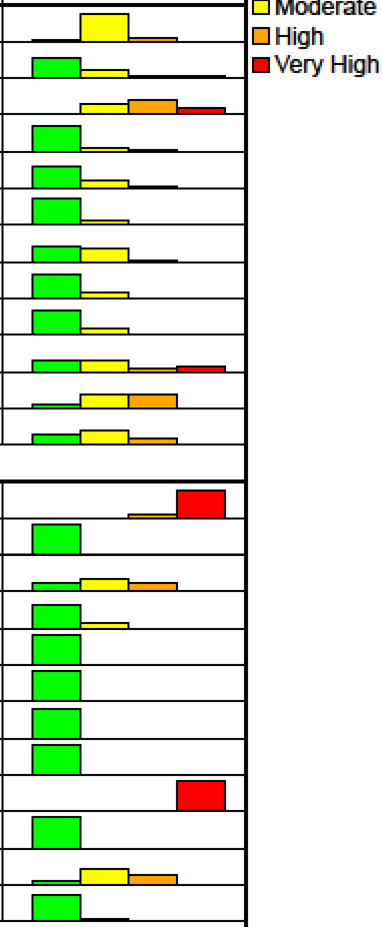

Little Skate (*Leucoraja erinacea*)

Overall Climate Vulnerability Rank: **Low** (88% certainty from bootstrap analysis).

Climate Exposure: **High**. Two exposure factors contributed to this score: Ocean Surface Temperature (3.9) and Ocean Acidification (4.0). Little Skate are demersal and complete their life cycle in marine habitats.

Biological Sensitivity: **Low**. Only one attribute scored above 2.5: Population Growth Rate (3.4). In general, skates have a low population growth rate (higher sensitivity to climate change) (Frisk 2010).

Distributional Vulnerability Rank: **High** (76% certainty from bootstrap analysis). Little Skate are habitat generalists and moderately mobile as adults, making seasonal movements. In addition, skate egg cases are subject to movement by currents and juveniles may move on scales of 1-10 km.

Directional Effect in the Northeast U.S. Shelf: The effect of climate change on Little Skate is estimated to be negative, but this estimate has a high degree of uncertainty (<66% certainty in expert scores). Little Skate are a cold water species and reductions in productivity may occur because of warming and ocean acidification. However, distributions have shifted southwards in recent years, contrary to the expectation of a northward shift with warming.

Data Quality: 88% of the data quality scores were 2 or greater indicate that data quality is moderate.

Climate Effects on Abundance and Distribution: Di Santo (2015) found that increased warming and acidification reduce body condition of newly hatched Little Skate. These reductions in size could result in reduced juvenile survival and thus recruitment. Nye et al. (2009) found that Little Skate moved southwards while a majority of species moved northwards. In addition, Collie et al. (2008) found Little Skate increasing in Narragansett Bay. The causes for this distribution shift and change in abundance remain unclear, but Frisk et al. (2008) suggested a connection between skate populations on the Northeast U.S. Shelf and the Scotian Shelf. Such a connection could play a role in the southward movement of Little Skate (e.g., increasing population size).

Life History Synopsis**:** Little Skate is a coastal, benthic elasmobranch that occurs from southeastern Newfoundland to Cape Hatteras, North Carolina (McEachran, 2002). Little Skate reach sexual maturity at about 4 years and mating occurs through internal fertilization (Packer et al., 2003). Spawning occurs year round with peaks in spring and fall (Packer et al., 2003). An individual may spawn twice annually and produce approximately 30 eggs per year (Packer et al., 2003). Eggs are encapsulated in a greenish-brown case and laid in pairs on the sea floor (Packer et al., 2003). The egg case adheres to the bottom substrates with sticky filaments, but frequently wash up on beaches (Packer et al., 2003). Eggs incubate for 6-12 months; duration is partially determined by temperature (Packer et al., 2003). Sea urchins and whelks prey on skate eggs (Packer et al., 2003). Juveniles hatch looking just like adults (Packer et al., 2003). Juveniles and adults occur on sand, gravel, and mud substrates and range from estuaries to 300 m, but usually occur at depths <111 m (McEachran, 2002; Packer et al., 2003). The inshore portion of the population migrates seasonally inshore and offshore regulated by temperature, as well as small migrations to the north and south (Packer et al., 2003). Juveniles and adults are generally found farther inshore and in estuaries during spring and summer, and overwinter in deeper water (McEachran, 2002; Packer et al., 2003). Juveniles consume crustaceans, particularly amphipods and decapods (Packer et al., 2003). The reliance on amphipods decreases with size, and the consumption of fish and polychaetes

increases (Packer et al., 2003). Adult skates consume primarily decapod crustaceans, amphipods, polychaetes and to a lesser degree isopods, bivalves, and small fish (Packer et al., 2003). The dominant predators of juvenile and adult Little Skates are sharks, other skates, teleost fishes, gray seals, and Rock Crabs (Packer et al., 2003). Little Skate is managed as part of the skate complex by the New England Fishery Management Council and are neither overfished nor is overfishing occurring (NEFSC, 2007).

Literature Cited:

Collie JS, Wood AD, Jeffries HP. Long-term shifts in the species composition of a coastal fish community. Can J Fish Aquat Sci. 2008; 65(7): 1352-1365. doi: 10.1139/F08-048

Di Santo V. Ocean acidification exacerbates the impacts of global warming on embryonic little skate, *Leucoraja erinacea* (Mitchill). J Exp Mar Bio Ecol. 2015; 463: 72-78. doi:10.1016/j.jembe.2014.11.006

Frisk MG. Life history strategies of batoids. In: Carrier, JC, Musick, JA, Heithaus, MR, editors, Sharks and Their Relatives II: Biodiversity, Adaptive Physiology, and Conservation. Boca Raton: CRC Press; 2010. 283-318.

Frisk MG, Miller TJ, Martell SJD, Sosebee K. New hypothesis helps explain elasmobranch "outburst" on Georges Bank in the 1980s. Ecol Appl. 2008; 18(1): 234-245. doi: 10.1890/06-1392.1

McEachran JD. Little Skate/ Leucoraja erinacea (Mitchill 1825). In: B.B. Collette BB, Klein-MacPhee G, editors, Fishes of the Gulf of Maine, 3rd ed. Washington: Smithsonian Institution Press; 2002. p. 67-69.

Northeast Fisheries Science Center (NEFSC). 2007. 44th Northeast Regional Stock Assessment Workshop (44th SAW): 44th SAW assessment report. US Dep Commer, Northeast Fish Sci Cent Ref Doc 07-10; 661 p. Accessed online (August 2015): http://www.nefsc.noaa.gov/publications/crd/crd0710/

Nye JA, Link JS, Hare JA, Overholtz WJ. Changing spatial distribution of fish stocks in relation to climate and population size on the Northeast United States continental shelf. Mar Ecol Prog Ser. 2009; 393: 111-129. doi: 10.3354/meps08220

Packer DB, Zetlin CA, Vitaliano JJ. 2003. Essential fish habitat source document: Little skate, Leucoraja erinacea, life history and habitat characteristics. NOAA Tech Memo NMFS NE 175; 66 p. Accessed online (August 2015): http://www.nefsc.noaa.gov/nefsc/publications/tm/tm175/

Return to Table of Contents

Longfin Inshore Squid - *Doryteuthis pealeii*

Overall Vulnerability Rank = Low

Biological Sensitivity = Low
Climate Exposure = High
Data Quality = 88% of scores ≥ 2

Doryteuthis pealeii	Expert Scores	Data Quality	Expert Scores Plots (Portion by Category)
Stock Status	1.8	2.6	
Other Stressors	1.4	2.0	
Population Growth Rate	1.2	2.6	
Spawning Cycle	1.4	2.5	
Complexity in Reproduction	1.5	2.4	
Early Life History Requirements	2.6	1.8	
Sensitivity to Ocean Acidification	1.4	2.4	
Prey Specialization	1.4	2.8	
Habitat Specialization	1.1	2.8	
Sensitivity to Temperature	1.5	3.0	
Adult Mobility	1.2	3.0	
Dispersal & Early Life History	1.8	2.1	
Sensitivity Score	Low		
Sea Surface Temperature	4.0	3.0	
Variability in Sea Surface Temperature	1.0	3.0	
Salinity	2.4	3.0	
Variability Salinity	1.2	3.0	
Air Temperature	1.7	3.0	
Variability Air Temperature	1.0	3.0	
Precipitation	1.0	3.0	
Variability in Precipitation	1.1	3.0	
Ocean Acidification	4.0	2.0	
Variability in Ocean Acidification	1.0	2.2	
Currents	2.1	1.0	
Sea Level Rise	1.3	1.5	
Exposure Score	High		
Overall Vulnerability Rank	Low		

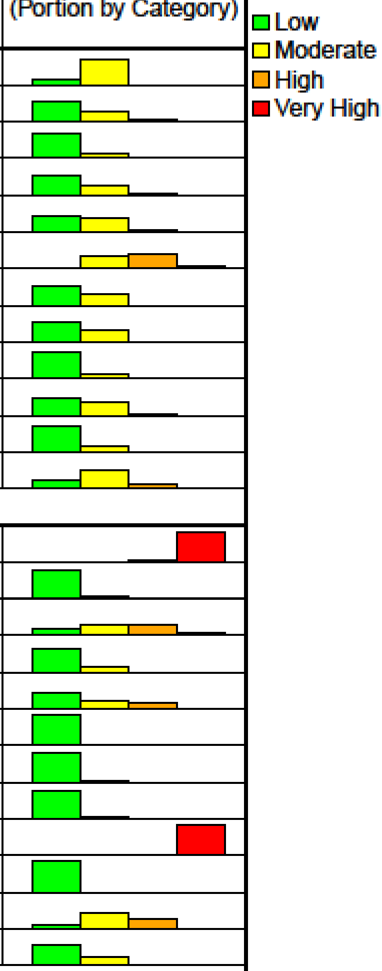

Longfin Inshore Squid (*Doryteuthis pealeii*)

Overall Climate Vulnerability Rank: **Low** (100% certainty from bootstrap analysis).

Climate Exposure: **High**. Two exposure factors contributed to this score: Ocean Surface Temperature (4.0) and Ocean Acidification (4.0). Exposure to ocean surface temperature and ocean acidification occurs during all life stages.

Biological Sensitivity: **Low**. Only one sensitivity score was above 2.5: Early Life History Requirements (2.6). Longfin Inshore Squid have benthic egg masses, which are predominantly found in coastal waters.

Distributional Vulnerability Rank: **High** (100% certainty from bootstrap analysis). Three of the attributes indicated vulnerability to distribution shift. Longfin Inshore Squid are habitat generalists occurring pelagic habitats from the coast to at least the shelf edge. They are mobile and make onshore-offshore seasonal migrations. Spawning occurs in coastal waters and paralarvae have the potential to be broadly dispersed.

Directional Effect in the Northeast U.S. Shelf: The effect of climate change on Longfin Inshore Squid on the Northeast U.S. Shelf is estimated to be positive, but this estimate is uncertain (66-90% certainty in expert scores). More habitat is likely to become available, particularly in the Gulf of Maine. However, productivity may decrease as ocean acidification continues.

Data Quality: 88% of the data quality scores were 2 or greater.

Climate Effects on Abundance and Distribution: Little is known about how climate will impact Longfin Inshore Squid; however, research on similar species suggests there may be some impact. For example, in the Northeast Atlantic, survival of embryos of the European Squid (*Loligo vulgaris*) decreased with increasing temperature and decreasing pH (Rosa et al., 2014). Timing of the inshore migration of Veined Squid (*Loligo forbesi*) is linked to temperature and in warmer years, this migration occurs earlier (Sims et al., 2001). Warming and ocean acidification will also likely impact Longfin Inshore Squid. Individuals raised under elevated pCO_2 demonstrated increased time to hatching and shorter mantle lengths; aragonite statoliths were also smaller, abnormally shaped and had an altered crystal structure (Kaplan et al., 2013). Manderson et al. (2011) found that temperature was an important component of Longfin Inshore Squid habitat and as temperature increases, the amount of available habitat should increase.

Life History Synopsis**:** Longfin Inshore Squid is a short-lived, coastal, schooling, pelagic cephalopod species that occurs from Newfoundland to the Gulf of Venezuela (NEFSC, 2011). This semelparous species most likely has a lifespan of less than one year (Cargnelli et al., 1999; NEFSC, 2011). Males grow faster and get larger than females, but most individuals mature around 16 cm mantle length (Cargnelli et al., 1999; NEFSC, 2011). Spawning occurs in bays and shallow coastal areas and on Georges Bank, probably year round, but with a peak in spring – summer (Cargnelli et al., 1999). Egg masses are laid in clusters on sand or mud bottom and attach to fixed objects like rocks, small boulders, and aquatic vegetation (Cargnelli et al., 1999; NEFSC, 2011). Eggs hatch after 10-27 days depending on temperature. Larvae are called paralarvae and are pelagic in near surface waters, but go deeper as they mature (Cargnelli et al., 1999). Paralarvae consume copepods primarily (Cargnelli et al., 1999). Paralarvae mature into juveniles, a stage that lasts approximately one month during which the squid remain in the near surface waters, but undergo a shift in diet from copepods to euphausiids and arrow worms (Cargnelli et al., 1999). At approximately 45 mm, the juvenile squid transition from a surface to a

demersal lifestyle and begin to look and migrate like the adults (Cargnelli et al., 1999). Subadults occur year-round, migrating offshore in fall, overwintering along the continental shelf edge, returning inshore in spring, and mingling with adults in the summer (Cargnelli et al., 1999). The diet of subadults transitions from planktonic organisms to benthic crustaceans, polychaetes, shrimp, and small squid (Cargnelli et al., 1999). Adult Longfin Inshore Squid form large schools based on size and migrate offshore to the shelf edge and slope for the winter and inshore for the summer (Cargnelli et al., 1999). Longfin Inshore Squid also make diel vertical migrations into the water column at night (Cargnelli et al., 1999; NEFSC, 2011). Temperature effects migrations, distribution, growth, and spawning of the species (NEFSC, 2011). Adult squid consume crustaceans, polychaetes, small fish, and other squid, with an increasing reliance on fish and squid with size and while onshore, and more crustaceans consumed while offshore (Cargnelli et al., 1999). Longfin Inshore Squid are prey to marine mammals (longfin pilot whale and common dolphin), finfish (Bluefish, Black Sea Bass, Atlantic Mackerel, Atlantic Cod, Haddock, Pollock, Silver Hake, Red Hake, Sea Raven, Angel Shark, Monkfish (Goosefish), Spiny Dogfish, and Summer Flounder), and diving birds (Cargnelli et al., 1999). The species is most abundant between Georges Bank and Cape Hatteras, North Carolina, and this region is managed as a single stock (Cargnelli et al., 1999). The Mid-Atlantic Fishery Management Council manages Longfin Inshore Squid under the Atlantic Mackerel, Squid, and Butterfish Fishery Management Plan. In 2010, Longfin Inshore Squid was not overfished, but it was unknown whether overfishing was occurring (NEFSC, 2011).

Literature Cited:

Cargnelli LM, Griesbach SJ, McBride C, Zetlin CA, Morse WW. 1999. Essential fish habitat source document: Longfin inshore squid, Loligo pealeii, life history and habitat characteristics. NOAA Tech Memo NMFS NE 146; 27 p. Accessed Online (October 2014): http://www.nefsc.noaa.gov/nefsc/publications/tm/tm146/

Kaplan MB, Mooney TA, McCorkle DC, Cohen AL. Adverse effects of ocean acidification on early development of squid (Doryteuthis pealeii). PLOS ONE, 2013; 8(5): e63714. doi: 10.1371/journal.pone.0063714

Manderson J, Palamara L, Kohut J, Oliver MJ. Ocean observatory data are useful for regional-habitat modeling of species with different vertical habitat preferences. Mar Ecol Prog Ser. 2011; 438: 1-17. doi:10.3354/meps09308

Mid-Atlantic Fishery Management Council (MAFMC). 2011. Amendment 11 to the Atlantic mackerel, squid, and butterfish (MSB) fishery management plan (FMP). MAFMC [Dover, DE]. 559 p + appendices. Accessed online (June, 2014): http://www.mafmc.org/fisheries/fmp/msb

Northeast Fisheries Science Center. 2011. 51st Northeast Regional Stock Assessment Workshop (51st SAW) Assessment Report. US Dept Commer, Northeast Fish Sci Cent Ref Doc. 11-02; 856 p. Available from: National Marine Fisheries Service, 166 Water Street, Woods Hole, MA 02543-1026. Accessed online (October 2014): http://www.nefsc.noaa.gov/publications/crd/crd1102/

Rosa R, Trübenbach K, Pimentel MS, Boavida-Portugal J, Faleiro F, Baptista M, et al. Differential impacts of ocean acidification and warming on winter and summer progeny of a coastal squid (Loligo vulgaris). J Exp Biol 2014; 217(4): 518-525. doi: 10.1242/jeb.096081

Return to Table of Contents

Sims DW, Genner MJ, Southward AJ, Hawkins SJ. Timing of squid migration reflects North Atlantic climate variability. Proc Biol Sci. 2001; 268(1485): 2607-2611. doi: 10.1098/rspb.2001.1847

Return to Table of Contents

Monkfish – *Lophius americanus*

Overall Vulnerability Rank = Low

Biological Sensitivity = Low
Climate Exposure = High
Data Quality = 79% of scores ≥ 2

Lophius americanus	Expert Scores	Data Quality	Expert Scores Plots (Portion by Category)
Stock Status	2.0	2.6	
Other Stressors	1.5	2.0	
Population Growth Rate	3.0	2.0	
Spawning Cycle	2.4	2.8	
Complexity in Reproduction	1.7	1.6	
Early Life History Requirements	1.9	1.2	
Sensitivity to Ocean Acidification	1.1	1.4	
Prey Specialization	1.3	3.0	
Habitat Specialization	1.5	2.8	
Sensitivity to Temperature	2.0	2.8	
Adult Mobility	2.3	2.6	
Dispersal & Early Life History	2.0	2.4	
Sensitivity Score	Low		
Sea Surface Temperature	3.9	3.0	
Variability in Sea Surface Temperature	1.0	3.0	
Salinity	1.5	3.0	
Variability Salinity	1.2	3.0	
Air Temperature	1.0	3.0	
Variability Air Temperature	1.0	3.0	
Precipitation	1.0	3.0	
Variability in Precipitation	1.0	3.0	
Ocean Acidification	4.0	2.0	
Variability in Ocean Acidification	1.0	2.2	
Currents	2.1	1.0	
Sea Level Rise	1.1	1.5	
Exposure Score	High		
Overall Vulnerability Rank	Low		

Low
Moderate
High
Very High

Monkfish (Goosefish) (*Lophius americanus*)

Overall Climate Vulnerability Rank: **Low** (76% certainty from bootstrap analysis).

Climate Exposure: **High**. Two exposure factors contributed to this score: Ocean Surface Temperature (3.9) and Ocean Acidification (4.0). All life stages of Monkfish (Goosefish) use marine habitats.

Biological Sensitivity: **Low**. Only one sensitivity attributes scored above 2.5: Population Growth Rate (3.0). Monkfish (Goosefish) are relatively slow growing and late maturing (Steimle et al., 1999).

Distributional Vulnerability Rank: **High** (72% certainty from bootstrap analysis). Monkfish (Goosefish) are habitat generalists that make seasonal movements and have dispersive early life history stages.

Directional Effect in the Northeast U.S. Shelf: The effect of climate change on Monkfish (Goosefish) on the Northeast U.S. Shelf is estimated to be neutral, but with a moderate degree of uncertainty (66-90% certainty in expert scores). Monkfish (Goosefish) is a temperate water fish and the effect of warming on habitat availability in the Northeast U.S. is unclear. There is not much information regarding productivity, thereby creating uncertainty as to the directional effect of climate.

Data Quality: 79% of the data quality scores were 2 or greater indicate that data quality is moderate.

Climate Effects on Abundance and Distribution: Relatively little is known regarding climate effects on Monkfish (Goosefish). The abundance of a congener, *Lophius piscatorius*, has increased in Iceland in recent years, likely as a result of warming temperatures (Solmundsson et al., 2010). They may suggest that productivity of Monkfish (Goosefish), which are at the southern extent of their range in the Northeast U.S. Shelf, will decrease as warming continues. Monkfish (Goosefish) have shifted northwards (Nye et al., 2009) and their distribution is related to temperature (Murawski, 1993).

Life History Synopsis**:** Monkfish (Goosefish), or Monkfish, is a benthic, marine, anglerfish species that occurs from southern and eastern Grand Banks and northern Gulf of St. Lawrence to eastern Florida, but is only common north of Cape Hatteras, North Carolina (Steimle et al., 1999). Males mature after about 4 years, but females mature after about 5 years and live longer than males (Steimle et al., 1999; NEFSC, 2010). Spawning occurs in spring through autumn starting in the south, and eggs are released in long, floating, mucus veils (Steimle et al., 1999). Egg veils, and individual eggs, are buoyant on the surface and incubation lasts 1 week to 4.5 months depending on temperature (Steimle et al., 1999). Newly hatched larvae remain in the veil for 2-3 days after hatching, and are pelagic after emerging (Steimle et al., 1999). Larvae are zooplanktivores, consuming copepods, crustacean larvae, and chaetognaths (Steimle et al., 1999). After several weeks to a few months in the plankton, young juveniles slowly transition into benthic juveniles that resemble the adult (Steimle et al., 1999). Small juveniles consume fish (Sand Lance), crustaceans, and squid, but the consumption of inverts decreases with growth (Steimle et al., 1999). Swordfish, Spiny Dogfish, Thorny Skate, Smooth Dogfish, Atlantic Cod, Sandbar Shark, and Dusky Shark consume Monkfish (Goosefish) juveniles, and cannibalism by larger individuals may be a substantial source of mortality (Steimle et al., 1999). Juveniles and adults tolerate a wide range of temperatures and salinities, but avoid low salinity and temperature extremes (Caruso, 2002). In the Gulf of Maine, Monkfish (Goosefish) migrate offshore in winter – spring to avoid cold temperatures and find food, then return to coastal waters in summer – autumn (Steimle et al., 1999). In the mid-Atlantic region, Monkfish (Goosefish) move offshore in summer – autumn to avoid warm temperatures and find food (Steimle et al., 1999). Adult Monkfish (Goosefish) are found over hard sand, pebbly-gravel, mixed

sand and shell, mud, and clay substrate and rest for long periods in depressions on the bottom or partially covered in sediment (Steimle et al., 1999). The first dorsal spine is adapted to act as a lure to coax small fish to within range (Caruso, 2002). The large wide mouth opens quickly creating suction, and prey fishes are swallowed whole (Caruso, 2002). Adults are opportunistic ambush predators of invertebrates and fish (primarily squid, clupeids, hakes, American Plaice, Little Skate, Monkfish (Goosefish), and Sand Lances), but also eat sea birds and diving ducks (Steimle et al., 1999; Caruso, 2002). Monkfish (Goosefish) can consume large quantities of prey including animals almost as large as they are, but then refuse food for several days (Caruso, 2002). Large Monkfish (Goosefish) have few predators (Steimle et al., 1999). The New England and Mid-Atlantic Fishery Management Councils jointly manage Monkfish (Goosefish) through the Monkfish (Goosefish) Fishery Management Plan (NEFSC, 2010). The Monkfish (Goosefish) fishery is divided into two regions: north of Georges Bank and south of Georges Bank (NEFSC, 2010). Neither stock is overfished nor experiencing overfishing, but the northern stock was identified as most vulnerable (NEFSC, 2010).

Literature Cited:

Caruso JH. Monkfish (Goosefish)/ *Lophius americanus* Valenciennes 1837. Pages 264-270. In: BB Collete,G Klein-MacPhee (editors), Fishes of the Gulf of Maine, 3rd edition. Smithsonian Institution Press, Washington D.C. 2002; 882 p.

Murawski SA. Climate change and marine fish distributions: forecasting from historical analogy. Trans Amer Fish Soc. 1993; 122(5): 647-658. DOI: 0.1577/1548-8659(1993)122<0647:CCAMFD>2.3.CO;2

Northeast Fisheries Science Center (NEFSC). 50th Northeast Regional Stock Assessment Workshop (50th SAW) Assessment Report. US Dept Commer, Northeast Fish Sci Cent Ref Doc. 2010; 10-17: 844 p. Accessed online (August 2015): http://www.nefsc.noaa.gov/publications/crd/crd1017/

Nye JA, Link JS, Hare JA, Overholtz WJ. Changing spatial distribution of fish stocks in relation to climate and population size on the Northeast United States continental shelf. Mar Ecol Prog Ser. 2009; 393: 111-129. DOI: 0.3354/meps08220

Steimle FW, Morse WW, Johnson DL. Essential fish habitat source document: Monkfish (Goosefish), *Lophius americanus*, life history and habitat characteristics. NOAA Tech Memo. 1999; NMFS NE 127: 31 p. Accessed online (August 2015): http://www.nefsc.noaa.gov/nefsc/publications/tm/tm127/

Solmundsson J, Jonsson E, Bjornsson H. Phase transition in recruitment and distribution of Monkfish (Goosefish) (*Lophius piscatorius*) in Icelandic waters. Mar Biol. 2010; 157(2): 295-305. DOI: 10.1007/s00227-009-1317-8

[Return to Table of Contents]

Northern Kingfish – *Menticirrhus saxatilis*

Overall Vulnerability Rank = Moderate

Biological Sensitivity = Low
Climate Exposure = Very High
Data Quality = 75% of scores ≥ 2

Menticirrhus saxatilis	Expert Scores	Data Quality	Expert Scores Plots (Portion by Category)
Stock Status	2.2	0.2	
Other Stressors	2.1	1.6	
Population Growth Rate	1.2	2.2	
Spawning Cycle	2.1	3.0	
Complexity in Reproduction	2.1	2.4	
Early Life History Requirements	2.5	0.8	
Sensitivity to Ocean Acidification	1.6	2.4	
Prey Specialization	1.6	2.6	
Habitat Specialization	1.4	3.0	
Sensitivity to Temperature	1.2	2.6	
Adult Mobility	1.4	2.8	
Dispersal & Early Life History	2.4	1.0	
Sensitivity Score	Low		
Sea Surface Temperature	4.0	3.0	
Variability in Sea Surface Temperature	1.0	3.0	
Salinity	3.0	3.0	
Variability Salinity	1.2	3.0	
Air Temperature	4.0	3.0	
Variability Air Temperature	1.0	3.0	
Precipitation	1.2	3.0	
Variability in Precipitation	1.3	3.0	
Ocean Acidification	4.0	2.0	
Variability in Ocean Acidification	1.0	2.2	
Currents	2.0	1.0	
Sea Level Rise	1.3	1.5	
Exposure Score	Very High		
Overall Vulnerability Rank	Moderate		

Low
Moderate
High
Very High

Return to Table of Contents

Northern Kingfish (*Menticirrhus saxatilis*)

Overall Climate Vulnerability Rank: **Moderate** (83% certainty from bootstrap analysis).

Climate Exposure: **Very High**. Three exposure factors contributed to this score: Ocean Surface Temperature (4.0), Ocean Acidification (4.0) and Air Temperature (4.0). Exposure to all three factors occur during all life stages. Northern Kingfish have an onshore-offshore seasonal migration and are exposed to ocean waters and nearshore waters.

Biological Sensitivity: **Low**. Only one sensitivity attribute scored above 2.5: Early Life History Requirements (2.5). Northern Kingfish spawn in coastal waters and juveniles settle in shallow water.

Distributional Vulnerability Rank: **High** (100% certainty from bootstrap analysis). Three of the attributes indicated vulnerability to distribution shift. Northern Kingfish are habitat generalists and found along sandy beaches. They are mobile as adults and larvae disperse over moderately large areas.

Directional Effect in the Northeast U.S. Shelf: The effect of climate change on Northern Kingfish on the Northeast U.S. Shelf is estimated to be positive, but this estimate has uncertainty (66-90% certainty in expert scores) owing to the relative lack of information regarding this species. This species is more common in the south and thus there is the expectation that as warming occurs more areas on the Northeast U.S. shelf will be thermally suitable. The effect of ocean acidification over the next 30 years is likely to be minimal.

Data Quality: 75% of the data quality scores were 2 or greater.

Climate Effects on Abundance and Distribution: Relatively few studies have examined the effect of climate factors on Northern Kingfish. Studies in other regions on congeners indicate that river discharge may be an important factor in the distribution and productivity of these species (Carassou et al., 2011). Increases in precipitation in the Northeast may negatively impact Northern Kingfish, but increases in temperature may allow range extension (Howell and Auster, 2012).

Life History Synopsis**:** Northern Kingfish is an estuarine and coastal finfish species that occurs from the Gulf of Maine to the Gulf of Mexico, but is most abundant between Cape Hatteras, North Carolina, and Cape Cod Massachusetts (Klein-MacPhee, 2002; Able and Fahay, 2010). Kingfish mature between 1-2 years of age and spawn in estuarine and coastal areas in spring through late summer (Klein-MacPhee, 2002; Able and Fahay, 2010). Eggs are pelagic and incubate for 46-50 hours (Klein-MacPhee, 2002). Larvae occur mostly in coastal areas and the surf zone, and some move into estuaries during summer months (Able and Fahay, 2010). Juveniles resemble adults and live on or near bottom in estuaries and coastal waters with sandy bottom where they consume crustaceans, polychaetes, and clupeid fishes (Klein-MacPhee, 2002; Able and Fahay, 2010). Juveniles leave estuaries in fall (Able and Fahay, 2010). Adult kingfish prefer hard or sandy bottom, form schools, and have no swim bladder to make the drumming sounds common to other members of the Sciaenidae family (Klein-MacPhee, 2002). Juvenile and adult kingfish make large seasonal migrations, moving south and maybe offshore in winter, occur off North Carolina in spring, and are abundant along the coast between North Carolina and Massachusetts in summer and fall (Able and Fahay, 2010). Northern Kingfish are bottom feeders that consume crustaceans, polychaetes, and fishes (Klein-MacPhee, 2002). Northern Kingfish are not managed in the northeastern US region, and there is no directed commercial fishery for the species.

Return to Table of Contents

Literature Cited:

Able KW, Fahay MP. Ecology of estuarine fishes: temperate waters of the western North Atlantic. Baltimore: The Johns Hopkins University Press; 2010. 566p.

Carassou L, Dzwonkowski B, Hernandez FJ, Powers SP, Park K, Graham WM, et al. Environmental influences on juvenile fish abundances in a river-dominated coastal system. Mar Coast Fish. 2011; 3(1): 411-427. doi: 10.1080/19425120.2011.642492

Howell P, Auster PJ. Phase shift in an estuarine finfish community associated with warming temperatures. Mar Coast Fish. 2012; 4(1): 481-495. doi: 10.1080/19425120.2012.685144

Klein-MacPhee G. Northern Kingfish/ Menticirrhus saxatilis (Bloch and Schneider 1801). In: Collete BB, Klein-MacPhee G, editors, Fishes of the Gulf of Maine, 3rd edition. Washington: Smithsonian Institution Press; 2002. pp. 442-444.

Return to Table of Contents

Northern Quahog – *Mercenaria mercenaria*

Overall Vulnerability Rank = Very High

Biological Sensitivity = Very High
Climate Exposure = High
Data Quality = 88% of scores ≥ 2

Mercenaria mercenaria	Expert Scores	Data Quality	Expert Scores Plots (Portion by Category)
Stock Status	2.3	0.6	
Other Stressors	3.6	2.8	
Population Growth Rate	2.4	2.2	
Spawning Cycle	2.2	3.0	
Complexity in Reproduction	2.2	2.8	
Early Life History Requirements	2.3	2.7	
Sensitivity to Ocean Acidification	3.8	2.6	
Prey Specialization	1.3	3.0	
Habitat Specialization	2.5	3.0	
Sensitivity to Temperature	1.7	3.0	
Adult Mobility	3.9	3.0	
Dispersal & Early Life History	2.2	2.8	
Sensitivity Score	Very High		
Sea Surface Temperature	4.0	3.0	
Variability in Sea Surface Temperature	1.0	3.0	
Salinity	1.8	3.0	
Variability Salinity	1.2	3.0	
Air Temperature	3.1	3.0	
Variability Air Temperature	1.0	3.0	
Precipitation	1.2	3.0	
Variability in Precipitation	1.2	3.0	
Ocean Acidification	4.0	2.0	
Variability in Ocean Acidification	1.0	2.2	
Currents	2.1	1.0	
Sea Level Rise	2.4	1.5	
Exposure Score	High		
Overall Vulnerability Rank	Very High		

Low
Moderate
High
Very High

Return to Table of Contents

Northern Quahog (*Mercenaria mercenaria*)

Overall Climate Vulnerability Rank: **Very High** (74% certainty from bootstrap analysis).

Climate Exposure: **High**. Two exposure factors contributed to this score: Ocean Surface Temperature (4.0) and Ocean Acidification (4.0). Exposure to Air Temperature was also high (3.1). Northern Quahog utilize near coastal and intertidal habitats and have a calcium carbonate shell.

Biological Sensitivity: **Very High**. Three sensitivity attributes scored above 3.5: Sensitivity to Ocean Acidification (3.8), Adult Mobility (3.9) and Other Stressors (3.6). Northern Quahog are sessile and have a calcium carbonate shell. Adults are intertidal / shallow water and subject to a number of other stressors including contaminants and habitat loss.

Distributional Vulnerability Rank: **High** (54% certainty from bootstrap analysis).

Directional Effect in the Northeast U.S. Shelf: The effect of climate change on Northern Quahog on the Northeast U.S. Shelf is very likely to be negative (>95% certainty in expert scores). Ocean acidification will likely negatively impact molluscs, including Northern Quahog. Larval survival and recruitment could be negatively impacted by warming, thereby decreasing productivity. Warming may also decrease available habitat resulting in shifts in distribution.

Data Quality: 88% of the data quality scores were 2 or greater indicate that data quality is moderate.

Climate Effects on Abundance and Distribution: Growth in Northern Quahog is related to temperature (Jones et al., 1989). Larval survival and growth exhibit a domed shaped response to temperature with an optimum between 25-30 ^{o}C (Davis and Calabrese, 1964). Larval development is during summer and temperatures in excess of 30 ^{o}C could be detrimental. Larval growth and survival are also related to salinity with an optimum at a salinity of 27. Under CO_2 concentrations estimated to occur later this century, Northern Quahog larvae exhibited declines in survivorship (>50%), as well as significantly delayed metamorphosis and significantly smaller sizes (Talmage and Gobler, 2009). Net calcification rate also decreased with decreasing aragonite saturation state (Reis et al., 2009). However, carbonate chemistry and ocean acidification are affected by many factors including atmospheric CO_2, making future aragonite saturation state in these systems difficult to project (Waldbusser and Salisbury, 2014).

Life History Synopsis**:** Northern Quahog, or the hard clam, is a highly fecund, estuarine and marine shellfish species found from the Gulf of St. Lawrence to the Atlantic coast of Florida and in Texas. Quahog need up to three years to reach maturity (Stanley and Dewitt, 1983). Spawning occurs from May - August beginning in the southern part of the range and continuing to the north (Stanley and Dewitt, 1983). Gamete release is triggered by rising temperatures within the range of 21-30 ^{o}C, and females release eggs several times over a 2-2.5 month period with the first release being the largest (Stanley and Dewitt, 1983). The pelagic eggs hatch after 12-14 hours and can be carried many kilometers from the spawning site (Stanley and Dewitt, 1983). The larval or veliger stage can be as short as 6-12 days depending on temperature (Stanley and Dewitt, 1983). Settlement is determined by size and can be delayed by low salinities (Stanley and Dewitt, 1983). Seed clams prefer sandy substrate with bits of shell or detritus (Stanley and Dewitt, 1983). Juveniles consume phytoplankton and small zooplankton (Stanley and Dewitt, 1983). Adult Northern Quahogs inhabit intertidal and subtidal bays and estuaries and move very little (a few cm) once settled (Stanley and Dewitt, 1983). As filter feeders, Northern Quahog strain plankton and microorganisms from the bottom water (Stanley and Dewitt, 1983). Gastropods, crabs,

shrimp, sea stars, fish, and birds consume Northern Quahog (Stanley and Dewitt, 1983; Tarnowski, 2007). There is no federal stock assessment for this species. Commercial and recreational harvests are managed on a state-by-state basis mostly through bag limits and gear limitations (e.g., NCDMF, 2001; MDDNR, 2012).

Literature Cited:

Davis HC, Calabrese A. Combined effects of temperature and salinity on development of eggs and growth of larvae of *M. mercenaria* and *C. virginica*. Fish Bull. 1964; 63(3): 643-655. Available: http://fishbull.noaa.gov/63-3/davis.pdf

Jones DS, Arthur MA, Allard DJ. Sclerochronological records of temperature and growth from shells of *Mercenaria mercenaria* from Narragansett Bay, Rhode Island. Mar Biol. 1989; 102(2): 225-234. doi: 10.1007/BF00428284

Maryland Department of Natural Resources (MDDNR). 2011 Fishery management Plan report to the legislative committees. 2012. Available: http://dlslibrary.state.md.us/publications/Exec/DNR/FS/NR4-215%28g%29_2011.pdf

North Carolina Division on Marine Fisheries (NCDMF). North Carolina Hard Clam Fishery Management Plan. Morehead City, North Carolina. 2001. 158p. Available: http://www.ncdmf.net/download/CLAMFMP.pdf

Ries JB, Cohen AL, McCorkle DC. Marine calcifiers exhibit mixed responses to CO2-induced ocean acidification. Geol. 2009; 37(12), 1131-1134. doi: 10.1130/G30210A.1

Stanley JG, DeWitt R. Species profiles: life histories and environmental requirements of coastal fishes and invertebrates (North Atlantic) - hard clam. U.S. Fish Wildl. Serv. FWS/OBS-82/11.18. U.S. Army Corps of Engineers, 1983. TR EL-82-4. 19pp. Available: http://www.nwrc.usgs.gov/publications/specprof.htm

Talmage SC, Gobler CJ. The effects of elevated carbon dioxide concentrations on the metamorphosis, size, and survival of larval hard clams (Mercenaria mercenaria), bay scallops (Argopecten irradians), and Eastern oysters (Crassostrea virginica). Limnol Oceanogr. 2009; 54(6): 2072-2080. doi: 10.4319/lo.2009.54.6.2072

Tarnowski M. Hard-Shell Clam *Mercenaria mercenaria*. 2007. Available: http://dnr2.maryland.gov/Fisheries/Pages/Fish-Facts.aspx?fishname=Shellfish%20-%20Hard%20Shell%20Clam

Waldbusser GG, Salisbury JE. Ocean acidification in the coastal zone from an organism's perspective: multiple system parameters, frequency domains, and habitats. Ann Rev Mar Sci, 2014; 6; 221-247. doi: 10.1146/annurev-marine-121211-172238

Return to Table of Contents

Northern Shortfin Squid – *Illex illecebrosus*

Overall Vulnerability Rank = Low

Biological Sensitivity = Low
Climate Exposure = High
Data Quality = 83% of scores ≥ 2

Illex illecebrosus	Expert Scores	Data Quality	Expert Scores Plots (Portion by Category)
Stock Status	2.1	2.2	
Other Stressors	1.2	1.2	
Population Growth Rate	1.1	2.6	
Spawning Cycle	1.3	2.1	
Complexity in Reproduction	1.5	1.9	
Early Life History Requirements	2.4	2.4	
Sensitivity to Ocean Acidification	1.8	2.0	
Prey Specialization	1.3	2.8	
Habitat Specialization	1.2	2.8	
Sensitivity to Temperature	1.3	2.8	
Adult Mobility	1.0	2.6	
Dispersal & Early Life History	1.2	2.7	
Sensitivity Score	Low		
Sea Surface Temperature	4.0	3.0	
Variability in Sea Surface Temperature	1.0	3.0	
Salinity	1.7	3.0	
Variability Salinity	1.2	3.0	
Air Temperature	1.7	3.0	
Variability Air Temperature	1.0	3.0	
Precipitation	1.0	3.0	
Variability in Precipitation	1.1	3.0	
Ocean Acidification	4.0	2.0	
Variability in Ocean Acidification	1.0	2.2	
Currents	2.2	1.0	
Sea Level Rise	1.1	1.5	
Exposure Score	High		
Overall Vulnerability Rank	Low		

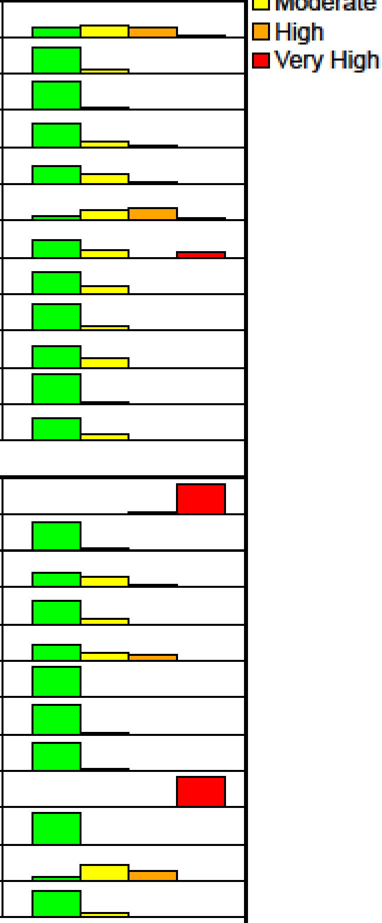

Northern Shortfin Squid (*Illex illecebrosus*)

Overall Climate Vulnerability Rank: **Low** (100% certainty from bootstrap analysis).

Climate Exposure: **High**. Two exposure factors contributed to this score: Ocean Surface Temperature (4.0) and Ocean Acidification (4.0). Exposure to ocean surface temperature and ocean acidification occurs during all life stages.

Biological Sensitivity: **Low**. All sensitivity scores were below 2.5.

Distributional Vulnerability Rank: **Very High** (100% certainty from bootstrap analysis). Four of the attributes indicated vulnerability to distribution shift. Northern Shortfin Squid are habitat generalists and occur in pelagic habitats both on and off the continental shelf. They are highly mobile and make large seasonal migrations on and off the shelf. Spawning occurs in slope waters and larvae (called paralarvae) have the potential to be broadly dispersed.

Directional Effect in the Northeast U.S. Shelf: The effect of climate change on Northern Shortfin Squid on the Northeast U.S. Shelf is estimated to be positive, but this estimate is highly uncertain (<66% certainty in expert scores). Warming may increase available habitat on the Northeast U.S. Shelf and a northward shift in the Gulf Stream may result in more squid on the shelf. However, population productivity may decrease as ocean acidification continues creating some uncertainty in the directional effect of climate change.

Data Quality: 83% of the data quality scores were 2 or greater.

Climate Effects on Abundance and Distribution: There is some evidence that Northern Shortfin Squid are affected by climate factors. Egg development is inhibited below a threshold of 12.5 °C (Coelho et al., 1994) and thus warming may open more spawning habitat off the Northeast U.S. Shelf. Abundance fluctuates in concert with the North Atlantic Oscillation (NAO; Dawe et al., 2000). Dawe et al. (2007) suggested environmentally driven alternation between Northern Shortfin Squid and Inshore Longfin Squid. This suggests that conditions that increase productivity of Northern Shortfin Squid may decrease productivity of Inshore Longfin Squid.

Life History Synopsis**:** Northern Shortfin Squid is a highly migratory, short-lived, marine species that occurs from the Sea of Labrador to the Straits of Florida (Hendrickson and Holmes, 2004). The spawning patterns of Northern Shortfin Squid are largely unknown. In captivity, females matured in 40-60 days (Hendrickson and Holmes, 2004). The lifespan of the semelparous Northern Shortfin Squid is <1 year with mortality within days of spawning (Hendrickson and Holmes, 2004). The majority of spawning occurs between October and June, but the species may spawn year round (Hendrickson and Holmes, 2004). The winter spawning habitat is unknown, but spawning has been confirmed on the Mid-Atlantic shelf and offshore areas along the Gulf Stream-Slope water frontal zone; south of Cape Hatteras may also be important based on observations of paralarvae and hatchlings in these areas (Hendrickson and Holmes, 2004). Females can produce multiple, neutrally buoyant, egg masses housed in a gelatinous balloon and containing thousands of eggs (10,000 – 100,000; Hendrickson and Holmes, 2004). Eggs hatch after 1-2 weeks depending on temperature (Hendrickson and Holmes, 2004). Paralarvae hatch at 1.1 mm mantle length, develop into a transitional stage by 5 mm mantle length, and reach the juvenile stage at 7-10 mm mantle length (Hendrickson and Holmes, 2004). Paralarvae occur year round in the Gulf Stream, but are most common in winter-spring (Hendrickson and Holmes, 2004). Eggs and paralarvae require waters > 12.5^{o}C, and the Gulf Stream is likely the main route of transport

(Hendrickson and Holmes, 2004; Dawe et al., 2007). Hatchlings have been collected only south of Cape Hatteras, but identification to species is difficult in this area (Hendrickson and Holmes, 2004). Juveniles and adults migrate onto the Newfoundland to Cape Hatteras shelf in spring and by late autumn migrate offshore, possibly to a winter spawning site (Hendrickson and Holmes, 2004). The proportion of the population that remains offshore year round is unknown (Hendrickson and Holmes, 2004). Males are smaller than females and begin to disappear from the shelf in early autumn (Hendrickson and Holmes, 2004). The cause of this shift in sex ratio is unknown, but the males could be migrating to offshore mating grounds earlier than females or experiencing increased mortality, possibly due to cannibalism by the larger females (Hendrickson and Holmes, 2004). Fish, squid, and crustaceans make up the majority of the diet, with an ontogenetic shift from primarily crustaceans to fish (early life stages of several species and adult Capelin) and squid (including cannibalism) with increased size (Hendrickson and Holmes, 2004). Predators of Northern Shortfin Squid include several benthic and pelagic predators such as Bluefin Tuna, Silver and Red Hakes, Bluefish, Monkfish (Goosefish), Fourspot Flounder, Atlantic Cod, Sea Raven, Spiny Dogfish, Swordfish, pilot whales, common dolphin, shearwaters, gannets, and fulmars (Hendrickson and Holmes, 2004). Northern Shortfin Squid has an interesting array of defense mechanisms including: camouflage coloration, schooling behavior, jetting, and ink release (Hendrickson and Holmes, 2004). Although considered one stock commercially exploited from Newfoundland to Cape Hatteras, North Carolina, the species is managed by Northwest Atlantic Fisheries Organization in Canadian waters and by the Mid-Atlantic Fishery Management Council in United States waters. The Mid-Atlantic Council manages Northern Shortfin Squid as part of the Atlantic Mackerel, Squid, and Butterfish Fishery Management Plan. In 2005, the stock was overfished and overfishing status could not be determined due to a lack of reliable stock biomass and fishing mortality estimates (NEFSC, 2006).

Literature Cited:

Coelho ML, Stobberup KA, O'Dor R, Dawe EG. Life history strategies of squid, Illex illecebrosus, in the Northwest Atlantic. Aquat Living Resour. 1994; 7: 233–246. doi: http://dx.doi.org/10.1051/alr:1994026

Dawe EG, Colbourne EB, Drinkwater KF. Environmental effects on recruitment of short-finned squid (Illex illecebrosus). ICES J Mar Sci. 2000; 57: 1002-1013. doi: 10.1006/jmsc.2000.0585

Dawe EG, Hendrickson LC, Colburne EB, Drinkwater KF, Showell MA. Ocean climate effects on the relative abundance of short-finned (Illex illecebrosus) and long-finned (Loligo pealeii) squid in the Northwest Atlantic Ocean. Fish Oceanog. 2007; 16: 303-316. doi: 10.1111/j.1365-2419.2007.00431.x

Hendrickson LC, Holmes EM. 2004. Essential fish habitat source document: northern shortfin squid, Illex illecebrosus, life history and habitat characteristics (2nd edition). NOAA Tech Memo NMFS NE 191; 36 p. Accessed online (February 2015): http://www.nefsc.noaa.gov/nefsc/publications/tm/tm191/

Northeast Fisheries Science Center. 2006. 42nd Northeast Regional Stock Assessment Workshop (42nd SAW) stock assessment report, part A: silver hake, Atlantic mackerel, and northern shortfin squid (CRD 06-09a). U.S. Dep. Commer., Northeast Fish. Sci. Cent. Ref. Doc. 06-09a; 284 p. Accessed online (February 2015): http://www.nefsc.noaa.gov/publications/crd/crd0609/

Return to Table of Contents

Northern Shrimp – *Pandalus borealis*

Overall Vulnerability Rank = High

Biological Sensitivity = High
Climate Exposure = High
Data Quality = 83% of scores ≥ 2

Pandalus borealis	Expert Scores	Data Quality	Expert Scores Plots (Portion by Category)
Stock Status	3.6	1.4	
Other Stressors	2.3	2.0	
Population Growth Rate	1.2	2.8	
Spawning Cycle	3.1	3.0	
Complexity in Reproduction	2.8	2.2	
Early Life History Requirements	2.9	1.8	
Sensitivity to Ocean Acidification	2.2	2.6	
Prey Specialization	1.7	2.4	
Habitat Specialization	2.6	3.0	
Sensitivity to Temperature	3.2	3.0	
Adult Mobility	1.7	2.6	
Dispersal & Early Life History	2.1	2.2	
Sensitivity Score	High		
Sea Surface Temperature	4.0	3.0	
Variability in Sea Surface Temperature	1.0	3.0	
Salinity	1.0	3.0	
Variability Salinity	1.2	3.0	
Air Temperature	1.4	3.0	
Variability Air Temperature	1.0	3.0	
Precipitation	1.1	3.0	
Variability in Precipitation	1.1	3.0	
Ocean Acidification	4.0	2.0	
Variability in Ocean Acidification	1.0	2.2	
Currents	2.1	1.0	
Sea Level Rise	1.6	1.5	
Exposure Score	High		
Overall Vulnerability Rank	High		

Low
Moderate
High
Very High

Northern Shrimp (*Pandalus borealis*)

Overall Climate Vulnerability Rank: **High** (99% certainty from bootstrap analysis).

Climate Exposure: **High**. Two exposure factors contributed to this score: Ocean Surface Temperature (4.0) and Ocean Acidification (4.0). All life stages of Northern Shrimp use marine habitats.

Biological Sensitivity: **High**. Three sensitivity attributes scored above 3.0: Stock Size (3.6), Spawning Cycle (3.1), and Sensitivity to Temperature (3.2). Northern Shrimp in the Gulf of Maine is at very low population sizes. They migrate offshore to spawn in the summer, and spawning is sensitive to temperature (Koeller et al., 2009).

Distributional Vulnerability Rank: **High** (96% certainty from bootstrap analysis).

Directional Effect in the Northeast U.S. Shelf: The effect of climate change on Northern Shrimp on the Northeast U.S. Shelf is very likely to be negative (>95% certainty in expert scores). Effects of ocean acidification on crustaceans are equivocal, but warming decreases population productivity and reductions in recruitment have already been observed.

Data Quality: 83% of the data quality scores were 2 or greater indicate that data quality is moderate.

Climate Effects on Abundance and Distribution: The productivity of Northern Shrimp in the Gulf of Maine is affected by ocean temperatures. Reduced recruitment in recent years has been linked with increasing temperatures (Richards et al., 2012) and has led to closure of the fishery (ASMFC, 2015). In Greenland, warming temperatures has led to an increase in the area occupied by the species (Wieland, 2005). In experiments, larval survival was not affected by CO_2 levels expected by 2100, but development rates were lower than in a control group (Bechmann et al., 2011; Arnberg et al., 2013). However, effects of warming temperature are likely greater on survival than decreasing pH (Arnberg et al., 2013). Decreasing pH may also decrease the quality of Northern Shrimp to consumers raising the possibility of a socio-economic interaction (decreased value at lower pH) (Dupont et al., 2014).

Life History Synopsis**:** Northern Shrimp is a boreal crustacean that occurs in cold waters of the North Pacific and Arctic Oceans and from the Atlantic Ocean as far south as the Gulf of Maine (NEFSC, 2014). Northern Shrimp are hermaphroditic, maturing first as males at 2.5 years, then as females at 3.5 years, and only a small portion of the population lives to spawn more than once as a female (ASMFC, 2014). Spawning occurs in offshore waters during summer and early fall (NEFSC, 2014). Eggs are extruded onto the abdomen of the females during fall and are carried until hatching (NEFSC, 2014). During late fall, egg-bearing females migrate inshore where the eggs hatch in spring (NEFSC, 2014). On the Newfoundland-Labrador Shelf, incubation times averaged approximately 7 months (Fuentes-Yaco et al., 2007). Larvae develop through 6 larval stages over an approximately 3 month pelagic period (NEFSC, 2014). Cooler temperatures support higher recruitment (NEFSC, 2014), and the timing of hatching in relation to the onset and duration of the spring plankton bloom may be important to the growth of early life history stages (Fuentes-Yaco et al., 2007). Larvae consume phytoplankton, copepod eggs, and nauplii while pelagic, but become increasingly associated with the benthos and increasingly consume larger copepodites and detritus at later stages (Fuentes-Yaco et al., 2007). After metamorphosis, juveniles settle to the bottom and remain in inshore waters for a year or more before migrating to deeper offshore waters to complete maturation (NEFSC, 2014). Juveniles and young males are found farther inshore than older males and non-ovigerous females (NEFSC, 2014). Growth requires molting of

the exoskeleton and males undergo a series of transitional stages before maturing into females (ASMFC, 2014). Distribution is limited by temperature, salinity, depth, and substrate type (NEFSC, 2014). Organic-rich mud bottom is the preferred habitat, but rocky substrate and structure such as boulders and anemones are also used by the species (NEFSC, 2014). As the southern limit of their distribution, Northern Shrimp in the Gulf of Maine are further restricted to cooler parts of the Gulf and deep basins where temperatures remain in the 0-5 ^{o}C range (NEFSC, 2014). Adults prey on plankton and benthic invertebrates, occurring near the bottom during the day, but moving up in the water column at night to feed (NEFSC, 2014). Many commercially important species feed on Northern Shrimp, including: Atlantic Cod, Redfish, Silver Hake, and White Hake, and Pollock (NEFSC, 2014). Commercial fisheries target female Northern Shrimp (ASMFC, 2014). The Atlantic States Marine Fisheries Commission manages these fisheries through an interstate fishery management plan for Northern Shrimp including Maine, New Hampshire, and Massachusetts. The most recent assessment did not provide acceptable models for the stock, but a moratorium on fishing was enacted due to record low abundance, biomass, and recruitment indices and a record low estimate of an index of current fishable biomass (ASMFC, 2014).

Literature Cited:

Arnberg M, Calosi P, Spicer JI, Tandberg AHS, Nilsen M, Westerlund S, et al. Elevated temperature elicits greater effects than decreased pH on the development, feeding and metabolism of northern shrimp (*Pandalus borealis*) larvae. Mar Bio. 2013; 160(8): 2037-2048. doi: 10.1007/s00227-012-2072-9

Atlantic States Marine Fisheries Commission (ASMFC). Northern Shrimp. Available: http://www.asmfc.org/species/northern-shrimp

Atlantic States Marine Fisheries Commission (ASMFC). Stock status report for Gulf of Maine Northern Shrimp – 2014. Atlantic States Marine Fisheries Commission's Northern Shrimp Technical Committee. 2014. Available: http://www.asmfc.org/species/northern-shrimp

Bechmann RK, Taban IC, Westerlund S, Godal BF, Arnberg M, Vingen S, et al. Effects of ocean acidification on early life stages of shrimp (*Pandalus borealis*) and mussel (*Mytilus edulis*). J Toxicol Environ Health, Part A, 2011; 74(7-9): 424-438. doi: 10.1080/15287394.2011.550460

Dupont S, Hall E, Calosi P, Lundve B. First evidence of altered sensory quality in a shellfish exposed to decreased pH relevant to ocean acidification. J Shell Res. 2014; 33(3): 857-861. doi: http://dx.doi.org/10.2983/035.033.0320

Fuentes-Yaco C. Koeller PA, Sathyendranath S, Platt T. Shrimp (*Pandalus borealis*) growth and timing of the spring phytoplankton bloom on the Newfoundland-Labrador Shelf. Fish Oceanogr. 2007; 16: 116-129. doi: 10.1111/j.1365-2419.2006.00402.x

Koeller P, Fuentes-Yaco C, Platt T, Sathyendranath S, Richards A, Ouellet P, et al. Basin-scale coherence in phenology of shrimps and phytoplankton in the North Atlantic Ocean. Science. 2009; 324(5928): 791-793. doi:10.1126/science.1170987

Northeast Fisheries Science Center (NEFSC). 58th Northeast Regional Stock Assessment Workshop (58th SAW) Assessment Report. 2014. US Dept Commer, Northeast Fish Sci Cent Ref Doc. 14-04; 784 p. Available: http://www.nefsc.noaa.gov/publications/crd/crd1404/

Richards R, Fogarty MJ, Mountain DG, Taylor MH. Climate change and northern shrimp recruitment variability in the Gulf of Maine. Mar Ecol Prog Ser. 2012; 464: 167-178. doi:10.3354/meps09869

Wieland K. Changes in recruitment, growth, and stock size of northern shrimp (*Pandalus borealis*) at West Greenland: temperature and density-dependent effects at released predation pressure. ICES J Mar Sci. 2005; 62(7): 1454-1462. doi: 10.1016/j.icesjms.2005.02.012

Return to Table of Contents

Ocean Pout – *Zoarces americanus*

Overall Vulnerability Rank = High

Biological Sensitivity = High
Climate Exposure = High
Data Quality = 88% of scores ≥ 2

Zoarces americanus	Expert Scores	Data Quality	Expert Scores Plots (Portion by Category)
Stock Status	3.6	2.6	
Other Stressors	1.7	2.0	
Population Growth Rate	3.0	2.2	
Spawning Cycle	2.1	2.4	
Complexity in Reproduction	1.5	2.6	
Early Life History Requirements	2.4	1.2	
Sensitivity to Ocean Acidification	1.6	2.2	
Prey Specialization	1.7	3.0	
Habitat Specialization	1.7	3.0	
Sensitivity to Temperature	2.8	2.8	
Adult Mobility	2.9	2.8	
Dispersal & Early Life History	3.7	2.4	
Sensitivity Score	High		
Sea Surface Temperature	3.9	3.0	
Variability in Sea Surface Temperature	1.0	3.0	
Salinity	1.4	3.0	
Variability Salinity	1.2	3.0	
Air Temperature	1.0	3.0	
Variability Air Temperature	1.0	3.0	
Precipitation	1.0	3.0	
Variability in Precipitation	1.0	3.0	
Ocean Acidification	4.0	2.0	
Variability in Ocean Acidification	1.0	2.2	
Currents	2.1	1.0	
Sea Level Rise	1.1	1.5	
Exposure Score	High		
Overall Vulnerability Rank	High		

Low
Moderate
High
Very High

Ocean Pout (*Zoarces americanus*)

Overall Climate Vulnerability Rank: **High.** (100% certainty from bootstrap analysis).

Climate Exposure: **High**. Two exposure factors contributed to this score: Ocean Surface Temperature (3.9) and Ocean Acidification (4.0). All life stages of Ocean Pout use marine habitats.

Biological Sensitivity: **High**. Three sensitivity attributes scored above 3.0: Dispersal and Early Life History (3.7), Stock Status (3.6) and Population Growth Rate (3.0). Ocean Pout do not have dispersive early life stages; eggs are benthic and larvae at large at hatch, quickly associating with benthic habitats (Steimle et al., 1999). Based on a recent assessment ocean pout is overfished but overfishing is not occurring (NEFSC, 2012). Ocean Pout are a relatively long lived species with a slow growth rate (Hoenig, 1983).

Distributional Vulnerability Rank: **Moderate** (93% certainty from bootstrap analysis). Ocean Pout are relatively sensitive to temperature and are habitat generalists, but prefer more complex habitats. However, early life stages are non-dispersive and adults are relatively sedentary.

Directional Effect in the Northeast U.S. Shelf: The effect of climate change on Ocean Pout on the Northeast U.S. Shelf is estimated to be negative, but this estimate is uncertain (66-90% certainty in expert scores). Ocean Pout is a cold-temperate species and warming will likely cause reductions in available habitat. However, there is little direct evidence of the effect of climate change on Ocean Pout productivity and distribution, which contributes to the uncertainty.

Data Quality: 88% of the data quality scores were 2 or greater indicate that data quality is moderate.

Climate Effects on Abundance and Distribution: Little work has been conducted examining the effect of climate on Ocean Pout productivity. In terms of distribution, Nye et al. (2009) found that the distribution of Ocean Pout shifted poleward and Murawski (1993) linked distribution to temperature: increased temperatures were related with more northward distributions.

Life History Synopsis**:** Ocean Pout is a cool-temperate, marine groundfish that occurs from Labrador and the southern Grand Banks to Virginia (Steimle et al., 1999). Adults reach maturity between 26 – 31 cm or 2-9 years (Steimle et al., 1999; Klein-MacPhee and Collette, 2002). Spawning occurs from late summer to early winter on hard-bottom areas with sheltered crevices in water <10 °C (Steimle et al., 1999). Eggs are fertilized internally then laid in nests in masses encased in a gelatinous substance (Klein-MacPhee and Collette, 2002). The female guards the nest until hatching, approximately 2-3 months, during which time she does not feed (Steimle et al., 1999; Klein-MacPhee and Collette, 2002). The larval stage is short or possibly non-existent. At hatching, Ocean Pout are large, well developed, demersal, and probably stay near the hatch site (Steimle et al., 1999). At this smallest size, Ocean Pout consume small benthic crustaceans, particularly harpacticoid copepods (Steimle et al., 1999). Juveniles inhabit shallow coastal waters around rocks, algae, and shell beds on the shelf and in saline estuarine waters (Steimle et al., 1999). Juvenile Ocean Pout filter sediment for prey and primarily consume gammarid amphipods and polychaetes (Steimle et al., 1999). Squid, Spiny Dogfish, Sea Raven, Atlantic Cod, skates, harbor seals, and cormorants are the primary predators of juveniles (Steimle et al., 1999). Adults are demersal and inhabit most sediment types across the shelf and into the saline parts of estuaries. They are more common at depths <100 m (Steimle et al., 1999). Ocean Pout make localized seasonal migrations from diverse areas of the shelf and estuaries in mid-winter through spring to cooler, rockier areas in summer where they remain through the spawning season to early winter (Klein-MacPhee and

Collette, 2002). Like juveniles, adults filter sediment for prey including polychaetes, molluscs, crustaceans, and echinoderms (Steimle et al., 1999). A variety of fish consume Ocean Pout including: Sandbar Shark, Spiny Dogfish, skates, Atlantic Cod, hakes, Sea Raven, and Bluefish (Steimle et al., 1999; Klein-MacPhee and Collette, 2002). Egg cannibalism may occur during periods of high stress (Steimle et al., 1999). The New England Fishery Management Council manages the Ocean Pout fishery through the Northeast Multispecies Fishery Management Plan. The population ranging from Cape Cod Bay and Georges Bank to Delaware is overfished, but overfishing is not occurring (NEFSC, 2012).

Literature Cited:

Klein-MacPhee G, Collette BB. Ocean Pout/ *Zoarces americanus* (Bloch and Schneider 1801). Pages 470-474. In: BB Collete G Klein-MacPhee (editors), Fishes of the Gulf of Maine, 3rd edition. Smithsonian Institution Press, Washington D.C. 2002; 882 p.

Murawski SA. Climate change and marine fish distributions: forecasting from historical analogy. Trans Amer Fish Soc. 1993; 122(5): 647-658. DOI: 0.1577/1548-8659(1993)122<0647:CCAMFD>2.3.CO;2

Northeast Fisheries Science Center (NEFSC). Assessment or Data Updates of 13 Northeast Groundfish Stocks through 2010. US Dept Commer, Northeast Fish Sci Cent Ref Doc. 2012; 12-06: 789 p. Accessed Online (August 2015): http://www.nefsc.noaa.gov/publications/crd/crd1206/

Nye JA, Link JS, Hare JA, Overholtz WJ. Changing spatial distribution of fish stocks in relation to climate and population size on the Northeast United States continental shelf. Mar Ecol Prog Ser. 2009; 393: 111-129. DOI: 0.3354/meps08220

Steimle FW, Morse WW, Berrien PL, Johnson DL, Zetlin CA. Essential fish habitat source document: Ocean Pout, *Macrozoarces americanus*, life history and habitat characteristics. NOAA Tech Memo 1999; NMFS NE 129: 26 p. Accessed online (August 2015): http://www.nefsc.noaa.gov/nefsc/publications/tm/tm129/

Return to Table of Contents

Ocean Quahog – *Arctica islandica*

Overall Vulnerability Rank = Very High

Biological Sensitivity = Very High
Climate Exposure = High
Data Quality = 92% of scores ≥ 2

Arctica islandica	Expert Scores	Data Quality	Expert Scores Plots (Portion by Category)
Stock Status	1.2	2.4	
Other Stressors	1.6	2.6	
Population Growth Rate	3.9	2.8	
Spawning Cycle	1.8	2.8	
Complexity in Reproduction	1.5	2.4	
Early Life History Requirements	2.0	2.2	
Sensitivity to Ocean Acidification	3.9	2.6	
Prey Specialization	1.4	3.0	
Habitat Specialization	1.8	3.0	
Sensitivity to Temperature	2.4	2.8	
Adult Mobility	3.9	2.6	
Dispersal & Early Life History	1.9	2.8	
Sensitivity Score	Very High		
Sea Surface Temperature	3.9	3.0	
Variability in Sea Surface Temperature	1.0	3.0	
Salinity	1.9	3.0	
Variability Salinity	1.2	3.0	
Air Temperature	2.0	3.0	
Variability Air Temperature	1.0	3.0	
Precipitation	1.1	3.0	
Variability in Precipitation	1.1	3.0	
Ocean Acidification	4.0	2.0	
Variability in Ocean Acidification	1.0	2.2	
Currents	2.1	1.0	
Sea Level Rise	1.8	1.5	
Exposure Score	High		
Overall Vulnerability Rank	Very High		

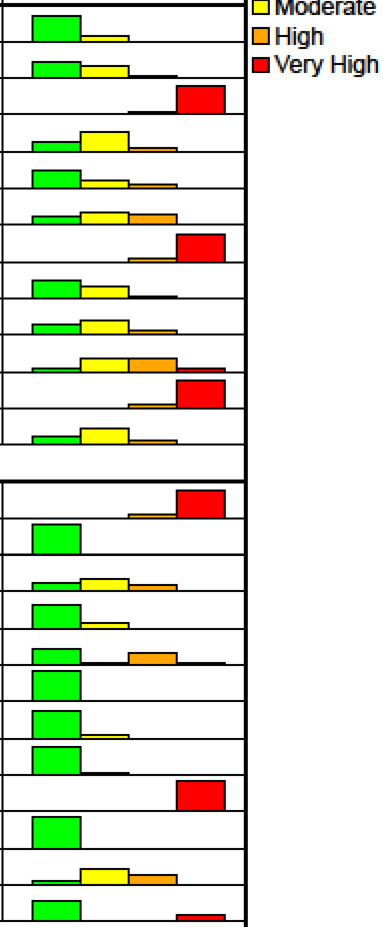

Return to Table of Contents

Return to Table of Contents

Ocean Quahog (*Arctica islandica*)

Overall Climate Vulnerability Rank: **Very High** (100% certainty from bootstrap analysis).

Climate Exposure: **High**. Two exposure factors contributed to this score: Ocean Surface Temperature (3.9) and Ocean Acidification (4.0). All life stages of Ocean Quahog use marine habitats.

Biological Sensitivity: **Very High**. Three sensitivity attributes scored above 3.5: Population Growth Rate (3.9), Sensitivity to Ocean Acidification (3.9), and Adult Mobility (3.9). Ocean Quahog are slow growing, sessile, and have a calcium carbonate shell.

Distributional Vulnerability Rank: **High** (71% certainty from bootstrap analysis).

Directional Effect in the Northeast U.S. Shelf: The effect of climate change on Ocean Quahog on the Northeast U.S. Shelf is very likely to be negative (>95% certainty in expert scores). Ocean acidification will likely negatively impact molluscs, including Ocean Quahog. Warming will likely reduce growth, which will affect productivity. Warming may also decrease the available habitat resulting in shifts in distribution.

Data Quality: 92% of the data quality scores were 2 or greater indicate that data quality is moderate.

Climate Effects on Abundance and Distribution: Growth of Ocean Quahog slows at higher temperatures (Hiebenthal et al., 2013) and since the species is long-lived, shell growth increments are used as a proxy for ocean temperatures (e.g., Wanamaler et al., 2009). Experiments suggest minimal effect of decreasing aragonite saturation (Hiebenthal et al., 2013) and increasing CO_2 levels (Stemmer et al., 2013) on growth. Thus increased warming, not ocean acidification, is likely to have the greatest impact on Ocean Quahog resulting from climate change.

Life History Synopsis**:** Ocean Quahog is a cold-water, long-lived, bivalve found on the continental shelf from Newfoundland to Cape Hatteras, North Carolina (Cargnelli et al., 1999). The mean age of maturity for this slow-growing species is 9-19 years, and they can live for over 200 years (Cargnelli et al., 1999; NEFSC, 2009). Ocean Quahog broadcast spawn over a protracted season from spring to fall (Cargnelli et al., 1999). Planktonic eggs mature into free-swimming trochophore larvae, which mature into shelled and swimming veliger larvae (Cargnelli et al., 1999). The final larval stage, the pediveliger stage, swims, but also has a foot for burrowing (Cargnelli et al., 1999). Temperature influences growth rates, with an optimal range from 13-15 ^oC (Cargnelli et al., 1999). Larvae consume phytoplankton (Cargnelli et al., 1999). Metamorphosis to the benthic juvenile stage occurs between 175-200 µm, and juvenile clams grow relatively quickly (Cargnelli et al., 1999). Juveniles occur in offshore sandy substrates, typically between 45-75 m depths in saline waters (Cargnelli et al., 1999). Adult quahogs occur in dense beds over level bottom just below the surface sediments in medium to fine grain sand (Cargnelli et al., 1999). Temperature regulates the cross-shelf distribution of the clam, with 6-16° C optimal, and temperatures above 16° C limit distribution (Cargnelli et al., 1999). The clams usually occur at depths between 25-61 m, and can survive anoxic conditions (Cargnelli et al., 1999). Adults and juveniles pump water from just above the substrate surface through their siphon and filter suspended phytoplankton, alternating between feeding and digestion periods (Cargnelli et al., 1999). Rock Crabs, sea stars, crustaceans, Longhorn Sculpin, Ocean Pout, Haddock, and Atlantic Cod are common predators (Cargnelli et al., 1999). The Mid-Atlantic Fishery Management Council manages Ocean Quahog through the Ocean Quahog and

Atlantic Surfclam Fishery Management Plan. The species is not overfished and overfishing is not occurring, but the population is in decline (NEFSC, 2009).

Literature Cited:

Cargnelli LM, Griesbach SJ. Packer DB, Weissberger E. Essential Fish Habitat Source Document: Ocean Quahog, Arctica islandica, Life History and Habitat Characteristics. 1999. NOAA Tech Memo NMFS-NE-148. Accessed Online (August 2015): http://www.nefsc.noaa.gov/nefsc/publications/tm/tm148/

Hiebenthal C, Philipp EE, Eisenhauer A, Wahl M. Effects of seawater pCO_2 and temperature on shell growth, shell stability, condition and cellular stress of Western Baltic Sea *Mytilus edulis* (L.) and *Arctica islandica* (L.). Mar Bio. 2013; 160(8): 2073-2087. DOI: 10.1007/s00227-012-2080-9

Northeast Fisheries Science Center (NEFSC). 48th Northeast Regional Stock Assessment Workshop (48th SAW) Assessment Report. 2009. Northeast Fish Sci Cent Ref Doc. 09-15; 834 p. Accessed Online (August 2015): http://www.nefsc.noaa.gov/publications/crd/crd0915/

Stemmer K, Nehrke G, Brey T. Elevated CO2 levels do not affect the shell structure of the bivalve *Arctica islandica* from the Western Baltic. PLOS ONE. 2013; 8(7): e70106. DOI: 10.1371/journal.pone.0070106

Wanamaker AD, Kreutz KJ, Schöne BR, Maasch KA, Pershing AJ, Borns HW. A late Holocene paleo-productivity record in the western Gulf of Maine, USA, inferred from growth histories of the long-lived Ocean Quahog (*Arctica islandica*). Inter J Earth Sci. 2009; 98(1): 19-29. DOI 10.1007/s00531-008-0318-z

Return to Table of Contents

Offshore Hake – *Merluccius albidus*

Overall Vulnerability Rank = Low

Biological Sensitivity = Low
Climate Exposure = High
Data Quality = 58% of scores ≥ 2

Merluccius albidus	Expert Scores	Data Quality	Expert Scores Plots (Portion by Category)
Stock Status	2.4	0.2	
Other Stressors	1.7	0.6	
Population Growth Rate	2.3	1.4	
Spawning Cycle	1.4	2.7	
Complexity in Reproduction	2.0	0.9	
Early Life History Requirements	2.4	1.2	
Sensitivity to Ocean Acidification	1.4	2.0	
Prey Specialization	1.4	3.0	
Habitat Specialization	1.5	1.9	
Sensitivity to Temperature	1.7	2.2	
Adult Mobility	1.6	1.2	
Dispersal & Early Life History	1.9	1.3	
Sensitivity Score	Low		
Sea Surface Temperature	3.9	3.0	
Variability in Sea Surface Temperature	1.0	3.0	
Salinity	1.6	3.0	
Variability Salinity	1.2	3.0	
Air Temperature	1.0	3.0	
Variability Air Temperature	1.0	3.0	
Precipitation	1.0	3.0	
Variability in Precipitation	1.0	3.0	
Ocean Acidification	4.0	2.0	
Variability in Ocean Acidification	1.0	2.2	
Currents	2.1	1.0	
Sea Level Rise	1.1	1.5	
Exposure Score	High		
Overall Vulnerability Rank	Low		

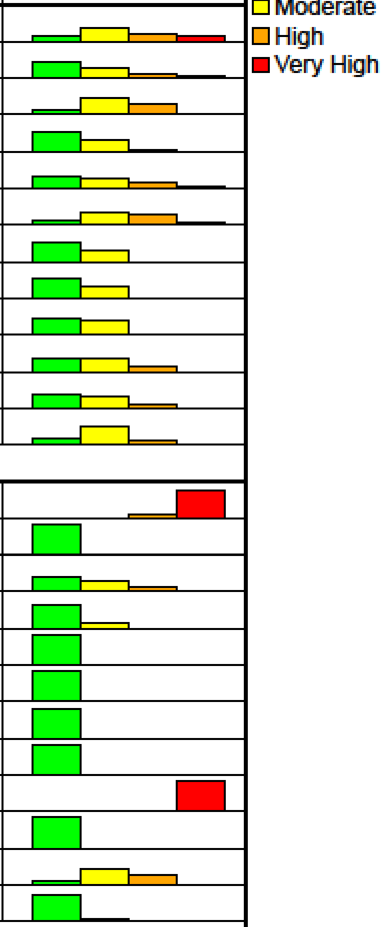

Return to Table of Contents

Offshore Hake (*Merluccius albidus*)

Overall Climate Vulnerability Rank: **Low** (92% certainty from bootstrap analysis).

Climate Exposure: **High**. Two exposure factors contributed to this score: Ocean Surface Temperature (3.9) and Ocean Acidification (4.0). All life stages of Offshore Hake use marine habitats.

Biological Sensitivity: **Low**. No sensitivity attributes scored above 2.5.

Distributional Vulnerability Rank: **High** (100% certainty from bootstrap analysis). Offshore Hake are habitat generalists, with moderate mobility, and dispersive early life history stages (Chang et al., 1999).

Directional Effect in the Northeast U.S. Shelf: The effect of climate change on Offshore Hake on the Northeast U.S. Shelf is estimated to be negative, but this estimate is highly uncertain (<66% certainty in expert scores). Offshore Hake is a cold-temperate species and warming will likely cause reductions in available habitat. However, there is little direct evidence of the effect of climate change on Offshore Hake productivity and distribution, which contributes to the uncertainty.

Data Quality: 58% of the data quality scores were 2 or greater indicate that data quality is moderate.

Climate Effects on Abundance and Distribution: There is relatively little information as to the effect of climate factors on Offshore Hake. Factors effecting variability in population are relatively unknown (Chang et al., 1999) and Offshore Hake were not included in regional studies of population distribution (Murawski, 1993; Nye et al., 2009).

Life History Synopsis**:** Offshore Hake is a marine species found along the outer continental shelf and upper slope from the southern edge of the Grand Banks to the Caribbean and Gulf of Mexico (Klein-MacPhee, 2002). The mean length at maturity for this sexually dimorphic species is 23 cm for males and 28 cm for females, who tend to grow faster and live longer than males (NEFSC, 2011). The spawning season is long, peaking between April and July, but may continue year round (Chang et al., 1999; NEFSC, 2011). Spawning occurs on the outer continental shelf and presumably also on the slope at or near the sea floor, but produces pelagic eggs (Chang et al., 1999). After 6-8 days, pelagic larvae hatch out of the eggs (Chang et al., 1999). Larvae transform at approximately 20 mm total length, but juveniles may not settle to benthic habitats until 30 mm TL (Chang et al., 1999). Juveniles and adults are demersal, occurring between 80 – 1170 m, but primarily occur around 200 m (Chang et al., 1999). Juveniles consume small fish, shrimp, and crustaceans (Chang et al., 1999). Monkfish (Goosefish), larger hakes, and likely other fishes prey on juvenile Offshore Hake (Chang et al., 1999). Adult Offshore Hake may make vertical migrations at night and mature females may congregate on deeper parts of the slope than the males and juveniles (Chang et al., 1999; Klein-MacPhee, 2002). Adult hakes consume mostly fish, such as Lanternfish, sardines, anchovies, and juvenile conspecifics, but occasionally also include crustaceans and squids in their diet (Chang et al., 1999). The only documented predator of adult Offshore Hake is Monkfish (Goosefish); however other predators likely consume the species but are not identified due to difficulty separating Offshore Hake from Silver Hake (Klein-MacPhee, 2002). Offshore Hake is treated as one stock in the northwest Atlantic and is managed through the New England Fishery Management Council's small mesh multi-species fishery management plan (NEFSC, 2011). The species is not overfished, but no determination could be made on the overfishing status for this data-poor stock (NEFSC, 2011).

Return to Table of Contents

Literature Cited:

Chang S, Berrien PL, Johnson DL, Zetlin CA. Essential fish habitat source document: Offshore Hake, *Merluccius albidus*, life history and habitat characteristics. NOAA Tech Memo 1999; NMFS NE 130: 24 p. Accessed Online (November 2014): http://www.nefsc.noaa.gov/nefsc/publications/tm/tm130/

Klein-MacPhee G. Offshore Hake/ *Merluccius albidus* (Mitchill 1818). Pages 217-218. In: BB Collete, G Klein-MacPhee (editors), Fishes of the Gulf of Maine, 3rd edition. Smithsonian InstitutionPress, Washington D.C. 2002; 882 p.

Murawski SA. Climate change and marine fish distributions: forecasting from historical analogy. Trans Amer Fish Soc. 1993; 122(5): 647-658. DOI: 0.1577/1548-8659(1993)122<0647:CCAMFD>2.3.CO;2

Northeast Fisheries Science Center (NEFSC). 51st Northeast Regional Stock Assessment Workshop (51st SAW) Assessment Report. US Dept Commer, Northeast Fish Sci Cent Ref Doc. 2011; 11-02: 856 p. Accessed Online (August 2015): http://www.nefsc.noaa.gov/publications/crd/crd1102/

Nye JA, Link JS, Hare JA, Overholtz WJ. Changing spatial distribution of fish stocks in relation to climate and population size on the Northeast United States continental shelf. Mar Ecol Prog Ser. 2009; 393: 111-129. DOI: 0.3354/meps08220

[Return to Table of Contents](#)

Pollock – *Pollachius virens*

Overall Vulnerability Rank = Moderate

Biological Sensitivity = Moderate
Climate Exposure = High
Data Quality = 88% of scores ≥ 2

Pollachius virens	Expert Scores	Data Quality	Expert Scores Plots (Portion by Category)
Stock Status	1.1	2.8	
Other Stressors	1.4	2.2	
Population Growth Rate	3.0	2.2	
Spawning Cycle	2.2	3.0	
Complexity in Reproduction	1.6	2.5	
Early Life History Requirements	2.6	1.2	
Sensitivity to Ocean Acidification	1.6	2.0	
Prey Specialization	1.5	3.0	
Habitat Specialization	1.3	3.0	
Sensitivity to Temperature	1.9	2.4	
Adult Mobility	1.7	2.5	
Dispersal & Early Life History	1.1	3.0	
Sensitivity Score	Moderate		
Sea Surface Temperature	3.9	3.0	
Variability in Sea Surface Temperature	1.0	3.0	
Salinity	1.2	3.0	
Variability Salinity	1.2	3.0	
Air Temperature	1.0	3.0	
Variability Air Temperature	1.0	3.0	
Precipitation	1.0	3.0	
Variability in Precipitation	1.0	3.0	
Ocean Acidification	4.0	2.0	
Variability in Ocean Acidification	1.0	2.2	
Currents	2.1	1.0	
Sea Level Rise	1.1	1.5	
Exposure Score	High		
Overall Vulnerability Rank	Moderate		

Low
Moderate
High
Very High

Pollock (*Pollachius virens*)

Overall Climate Vulnerability Rank: **Moderate** (72% certainty from bootstrap analysis).

Climate Exposure: **High**. Two exposure factors contributed to this score: Ocean Surface Temperature (3.9) and Ocean Acidification (4.0). All life stages of Pollock use marine habitats.

Biological Sensitivity: **Moderate**. Two sensitivity attributes scored above 2.5: Population Growth Rate (3.0) and Early Life History Requirements (2.6). Pollock are relatively late maturing (4-6 years) and long-lived (25 years) (NEFSC, 2012). Pollock are winter spawners and have a long egg and larval period. Juveniles use inshore habitats before moving offshore and joining adult populations (Cargnelli et al., 1999).

Distributional Vulnerability Rank: **High** (98% certainty from bootstrap analysis). Pollock are pelagic and highly mobile. Early life stages are dispersive.

Directional Effect in the Northeast U.S. Shelf: The effect of climate change on Pollock on the Northeast U.S. Shelf is very likely to be negative (>95% certainty in expert scores). Pollock is a cold-temperate species and warming could limit habitat; shifts into deeper water have been observed. There is also evidence for a congener that productivity will decrease as temperatures warm.

Data Quality: 88% of the data quality scores were 2 or greater indicate that data quality is moderate.

Climate Effects on Abundance and Distribution: Spawning activity and success is negatively impacted by higher temperatures in the congener *P. pollachius* (Suguet et al., 2005) suggesting that warming could decrease productivity of Pollock in the Northeast U.S. Shelf ecosystem. Although latitudinal movements in the distribution of Pollock have not been documented (Perry et al., 2005; Nye et al., 2009), Pollock have moved into deeper waters in recent years on both sides of the Atlantic (Dulvy et al., 2008; Nye et al., 2009).

Life History Synopsis**:** Pollock is a boreal, marine species found in the eastern and western Atlantic. In the western Atlantic, Pollock occur from western Greenland, Hudson Strait, and Labrador to Cape Hatteras, North Carolina, but are uncommon south of New Jersey (Able and Fahay, 2010). Pollock reach maturity between ages 3 and 6 (NEFSC, 2010). Spawning peaks from November to February as water temperature drops (peaking in 4.5-6°C water; Cargnelli et al., 1999). Most spawning occurs in the northern part of the range (Georges Bank, western Gulf of Maine, Massachusetts Bay, and Scotian Shelf) over hard, stony, or rocky bottom, but eggs and small larvae collected in the southern portion indicates limited spawning in areas near Delaware Bay and the Virginia Capes (Cargnelli et al., 1999; Able and Fahay, 2010). Some large adults form huge spawning aggregations in winter and spring (Klein-MacPhee, 2002; Able and Fahay, 2010). Eggs are pelagic and incubate for 1-2 weeks (Klein-MacPhee, 2002). Larvae are also pelagic on the continental shelf in 50-250 m cool water during February – May (Cargnelli et al., 1999; Able and Fahay, 2010). After 3-4 months, late larvae migrate inshore and by 25-30 mm have most of the adult characters (Cargnelli et al., 1999; Klein-MacPhee, 2002). Larvae consume phytoplankton and copepod nauplii when small then switch to copepods, amphipods, cumaceans, isopods, and larval fishes at later stages (Able and Fahay, 2010). Larvae transform to a pelagic juvenile stage at sizes >25 mm and move into estuaries during late winter or after approximately 6 months (Cargnelli et al., 1999; Able and Fahay, 2010). Juveniles remain in rocky coastal and estuarine areas for 1-2 years before moving offshore (Cargnelli et al., 1999; Able and Fahay, 2010). Juveniles feed near

vegetated areas and consume mostly euphausiids, but some amphipods, decapod larvae, isopods, copepods, polychaetes, and small fishes have also been observed in stomachs (Able and Fahay, 2010). Juvenile and adult Pollock distribution is highly influenced by temperature; avoiding habitat with temperature >15°C with seasonal migrations (Klein-MacPhee, 2002; Able and Fahay, 2010). Juveniles make several inshore-offshore migrations to avoid extreme temperature for up to two years before joining the adult populations and occur over sand, mud, rock, and vegetated bottom in a wide range of temperatures (Cargnelli et al., 1999). Juveniles join the adult population at approximately 20 cm during autumn and are found across the shelf in spring and autumn, but offshore and to the south (as far as the mid-Atlantic region) in the winter (Able and Fahay, 2010). Adults are found throughout the water column and make short inshore-offshore migrations to avoid temperature extremes and north-south migrations for spawning (Cargnelli et al., 1999; Klein-MacPhee, 2002). Pollock are deeper in the water column during spring and summer than during winter and adults move father offshore than juveniles (Cargnelli et al., 1999). Spawning adults move into shallower waters during the spawning season and the northern populations may migrate south to the Gulf of Maine and Georges Bank to spawn (Klein-MacPhee, 2002). Adult Pollock consume pelagic crustaceans (particularly euphausiids), small fishes, and squid (Able and Fahay, 2010). Spiny Dogfish, Monkfish (Goosefish), American lobster, minke whale, gray seal, harbor seal, and larger Pollock are the primary predators of the species (Klein-MacPhee, 2002). The New England Fishery Management Council manages Pollock as part of the Northeast Multispecies Fishery Management Plan. Pollock are not overfished, and overfishing is not occurring (NEFSC, 2010).

<u>Literature Cited</u>:

Able KW, Fahay MP. Ecology of estuarine fishes: temperate waters of the western North Atlantic. The Johns Hopkins University Press. Baltimore, MD. 2010; 566p.

Cargnelli LM, Griesbach SJ, Packer DB, Berrien PL, Johnson DL, Morse WW. Essential fish habitat source document: Pollock, Pollachius virens, life history and habitat characteristics. NOAA Tech Memo 1999; 131: 30 p. Accessed Online (November 2014):
http://www.nefsc.noaa.gov/nefsc/publications/tm/tm131/

Dulvy NK, Rogers SI, Jennings S, Stelzenmüller V, Dye SR, Skjoldal HR. Climate change and deepening of the North Sea fish assemblage: a biotic indicator of warming seas. J Appl Ecol. 2008; 45(4): 1029-1039. DOI: 10.1111/j.1365-2664.2008.01488.x

Klein-MacPhee G. Pollock/ *Pollachius virens* (Linnaeus 1758). Pages 247-252. In: BB Collette, G Klein-MacPhee (editors), Fishes of the Gulf of Maine, 3rd edition. Smithsonian InstitutionPress, Washington D.C. 2002; 882 p.

Northeast Fisheries Science Center (NEFSC). 50th Northeast Regional Stock Assessment Workshop (50th SAW) Assessment Report. US Dept Commer, Northeast Fish Sci Cent Ref Doc. 2010; 10-17: 844 p. Accessed Online (August 2015): http://www.nefsc.noaa.gov/publications/crd/crd1017/

Nye JA, Link JS, Hare JA, Overholtz WJ. Changing spatial distribution of fish stocks in relation to climate and population size on the Northeast United States continental shelf. Mar Ecol Prog Ser. 2009; 393: 111-129. DOI: 0.3354/meps08220

Perry AL, Low PJ, Ellis JR, Reynolds JD. Climate change and distribution shifts in marine fishes. Science, 2005; 308(5730): 1912-1915. DOI:10.1126/science.1111322

Return to Table of Contents

Suquet M, Normant Y, Gaignon JL, Quemener L, Fauvel C. Effect of water temperature on individual reproductive activity of pollack (*Pollachius pollachius*). Aquaculture. 2005; 243(1): 113-120. DOI: 10.1016/j.aquaculture.2004.09.021

[Return to Table of Contents](#)

Porbeagle – *Lamna nasus*

Overall Vulnerability Rank = High

Biological Sensitivity = High
Climate Exposure = High
Data Quality = 83% of scores ≥ 2

Lamna nasus	Expert Scores	Data Quality	Expert Scores Plots (Portion by Category)
Stock Status	3.8	2.6	
Other Stressors	1.4	1.8	
Population Growth Rate	4.0	2.8	
Spawning Cycle	2.2	2.8	
Complexity in Reproduction	1.4	1.8	
Early Life History Requirements	1.0	3.0	
Sensitivity to Ocean Acidification	1.1	3.0	
Prey Specialization	1.2	2.6	
Habitat Specialization	1.3	2.4	
Sensitivity to Temperature	2.0	2.8	
Adult Mobility	1.0	3.0	
Dispersal & Early Life History	1.0	3.0	
Sensitivity Score	High		
Sea Surface Temperature	3.9	3.0	
Variability in Sea Surface Temperature	1.0	3.0	
Salinity	2.0	3.0	
Variability Salinity	1.2	3.0	
Air Temperature	1.0	3.0	
Variability Air Temperature	1.0	3.0	
Precipitation	1.0	3.0	
Variability in Precipitation	1.0	3.0	
Ocean Acidification	4.0	2.0	
Variability in Ocean Acidification	1.0	2.2	
Currents	2.1	1.0	
Sea Level Rise	1.1	1.5	
Exposure Score	High		
Overall Vulnerability Rank	High		

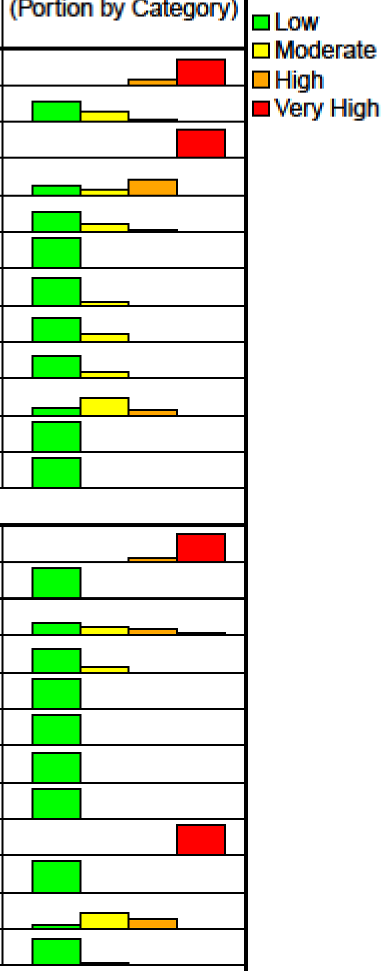

Porbeagle (*Lamna nasus*)

Overall Climate Vulnerability Rank: **High** (100% certainty from bootstrap analysis).

Climate Exposure: **High**. Two exposure factors contributed to this score: Ocean Surface Temperature (3.9) and Ocean Acidification (4.0). Porbeagle are pelagic and complete their life cycle in marine habitats.

Biological Sensitivity: **High**. Two attributes scored above 3.0: Population Growth Rate (3.8) and Stock Status (3.1). Porbeagle have low population growth rates (Cortés et al., 2010). Porbeagle is listed as vulnerable by the IUCN owing to low population abundance (http://www.iucnredlist.org/details/11200/0). Porbeagle is identified as a Species of Concern in the Western Atlantic by the U.S. (http://www.nmfs.noaa.gov/pr/pdfs/species/porbeagleshark_detailed.pdf).

Distributional Vulnerability Rank: **Very High** (100% certainty from bootstrap analysis). Porbeagle are habitat generalists and highly mobile. In addition, Porbeagle do not have larval early life stages.

Directional Effect in the Northeast U.S. Shelf: The effect of climate change on Porbeagle is very likely to be neutral (>95% certainty in expert scores). Porbeagle is a highly mobile cold-temperate shark. There is very little information available that suggests negative or positive effects of climate change.

Data Quality: 83% of the data quality scores were 2 or greater indicate that data quality is moderate.

Climate Effects on Abundance and Distribution: There is very little information on the effect of climate change on Porbeagle. Drinkwater et al. (2003) noted decreases in the catch of Porbeagle on the Scotian Shelf when colder Labrador Slope Water moved into the region.

Life History Synopsis**:** The Porbeagle is a migratory-pelagic, cold-temperate elasmobranch species found throughout the north Atlantic, south Atlantic, and south Pacific Oceans, but from Newfoundland to New Jersey and the Sargasso Sea in the western Atlantic (Campana et al., 2010a; Campana et al., 2010b). Maturity is reached at a later age and larger size for females than males: 50% maturity at 174 cm fork length (FL) and 8 years for males, 217 cm FL and 13 years for females (Jensen et al., 2002; Natanson et al., 2002). Research indicates that mating likely occurs off southern Newfoundland and the entrance to the Gulf of St Lawrence and on Georges Bank in fall (Jensen et al. 2002; Campana et al., 2010b). Porbeagles reproduce annually and are ovoviviparous (Jensen et al., 2002). Embryos are oophagous, but are not known to consume siblings (Jensen et al., 2002). After an 8-9 month gestation period, litters of 1-4 large individuals (60-70 cm) are born in late spring (Branstetter, 2002; Jensen et al. 2002). The locations of pupping grounds are unknown, but satellite tagging suggests females travel over 2000 km to the Sargasso Sea during the pupping season (Campana et al., 2010a). Porbeagles migrate seasonally from winter habitats in the southern portion of their range (namely Gulf of Maine, Georges Bank, and the southern Scotian Shelf), along the Scotian Shelf in spring, to their summer and fall habitat along the southern coast of Newfoundland and in the Gulf of St. Lawrence with little mixing with the eastern Atlantic population (Campana et al., 2010b). Porbeagles probably adjust their distribution in the water column to maintain their preferred temperature range, 5-10°C; but are able to maintain muscle and stomach temperatures greater than the ambient water temperature (Branstetter, 2002; Campana et al., 2010b). Adults consume Atlantic mackerel, herrings, other sharks, other small fishes, and squids (Branstetter, 2002). Porbeagle is listed as a NMFS Species of Concern. Population size in the western Atlantic dropped in the 1960s due to fishing pressure, but is on a long road to recovery now that fishing

pressure has been reduced (Campana et al., 2010b). The species is federally managed through the Consolidated Atlantic Highly Migratory Species fishery management plan (NMFS 2006) and is also managed in state waters through the Interstate Fishery Managemement Plan for Atlantic coastal sharks (ASMFC 2008) . Assessments are conducted by the International Commission for the Conservation of Atlantic Tunas (ICCAT) and Porbeagle are considered overfished, but overfishing is not occurring (ICCAT, 2009).

Literature Cited:

Branstetter S. Porbeagle/ Lamna nasus (Bonnaterre 1788). In: B.B. Collette BB, Klein-MacPhee G, editors, Fishes of the Gulf of Maine, 3rd ed. Washington: Smithsonian Institution Press; 2002. p. 30-32.

Campana, SE, Joyce W, Fowler M. Subtropical pupping ground for a cold-water shark. Can J Fish Aquat Sci. 2010a; 67: 769-773. doi: 10.1139/F10-020

Campana, SE; JF Gibson, M Fowler, A Dorey, and W Joyce. Population dynamics of porbeagle in the northwest Atlantic, with an assessment of status to 2009 and projections for recovery. Collect. Vol. Sci. Pap. ICCAT. 2010b; 65:2109-2182. Accessed online (August 2015): https://www.iccat.int/Documents/CVSP/CV065_2010/no_6/CV065062109.pdf

Cortes E, Arocha F, Beerkircher L, Carvalho F, Domingo A, Heupel M, et al. 2010. Ecological risk assessment of pelagic sharks caught in Atlantic pelagic longline fisheries. Aquat Living Resour. 2010; 23: 25-34. doi: 10.1051/alr/2009044

Drinkwater KF, Petrie B, Smith PC. (2003). Climate variability on the Scotian Shelf during the 1990s. In ICES Mar. Sci. Symp. 2003; 219: 40-49.

Jensen CF, Natanson LJ, PrattJr. HL, Kohler NE, Campana SE. The reproductive biology of the porbeagle shark (*Lamna nasus*) in the western North Atlantic Ocean. Fish Bull. 2002; 100(4): 727–738. Available: http://aquaticcommons.org/15246/1/08jensen.pdf

Natanson LJ, Mello JJ, Campana SE. Validated age and growth of the porbeagle shark (*Lamna nasus*) in the western North Atlantic Ocean. Fish Bull. 2002; 100(2): 266–278. Available: http://fishbull.noaa.gov/1002/10natans.pdf

ICCAT. 2009. Report of the 2009 Porbeagle stock assessments meeting. SCRS/2009/014 – Shark Stock Assessment. SCI-032/2009. Copenhagen, Denmark, June 22-27, 2009. Accessed online (August 2015): http://www.iccat.int/Documents/Meetings/Docs/2009_POR_ASSESS_ENG.pdf

Return to Table of Contents

Rainbow Smelt – *Osmerus mordax*

Overall Vulnerability Rank = Very High

Biological Sensitivity = High
Climate Exposure = Very High
Data Quality = 83% of scores ≥ 2

Osmerus mordax	Expert Scores	Data Quality	Expert Scores Plots (Portion by Category)
Stock Status	2.9	1.2	
Other Stressors	3.3	2.1	
Population Growth Rate	1.4	1.8	
Spawning Cycle	3.3	2.6	
Complexity in Reproduction	2.8	2.8	
Early Life History Requirements	3.2	2.2	
Sensitivity to Ocean Acidification	1.6	2.2	
Prey Specialization	1.5	3.0	
Habitat Specialization	2.8	3.0	
Sensitivity to Temperature	2.7	3.0	
Adult Mobility	1.9	2.9	
Dispersal & Early Life History	2.9	2.6	
Sensitivity Score	High		
Sea Surface Temperature	4.0	3.0	
Variability in Sea Surface Temperature	1.0	3.0	
Salinity	1.1	3.0	
Variability Salinity	1.2	3.0	
Air Temperature	4.0	3.0	
Variability Air Temperature	1.0	3.0	
Precipitation	1.3	3.0	
Variability in Precipitation	1.4	3.0	
Ocean Acidification	4.0	2.0	
Variability in Ocean Acidification	1.0	2.2	
Currents	2.0	1.0	
Sea Level Rise	3.1	1.5	
Exposure Score	Very High		
Overall Vulnerability Rank	Very High		

Low
Moderate
High
Very High

Return to Table of Contents

Rainbow Smelt (*Osmerus mordax*)

Overall Climate Vulnerability Rank: **High** (100% certainty from bootstrap analysis).

Climate Exposure: **Very High**. Three exposure factors contributed to this score: Ocean Surface Temperature (4.0), Ocean Acidification (4.0) and Air Temperature (4.0). Rainbow Smelt are anadromous, spawning in freshwater, developing in freshwater and estuarine habitats, feeding as adults in estuarine, coastal, and marine habitats.

Biological Sensitivity: **High**. Three sensitivity attributes scored above 3.0: Other Stressors (3.3), Spawning Cycle (3.3), and Early Life History Requirements (3.2). Rainbow Smelt are diadromous and exposed to a number of other stressors including habitat destruction, blockage to spawning habitats, and contaminants (Limburg and Waldman, 2009). Spawning is in the spring as temperatures warm (Murawski et al., 1980) and eggs and larvae develop in fresh and brackish water (Laprise and Dodson, 1989).

Distributional Vulnerability Rank: **Moderate** (89% certainty from bootstrap analysis). Several attributes indicate limited vulnerability to distribution shifts. Adults are small and not highly mobile. Eggs and larvae remain near spawning sites and use selective tidal-stream transport to enhance retention (Laprise and Dodson, 1989). Spawning sites are relatively specific and the proportion of natal homing is high (Bradbury et al., 2008).

Directional Effect in the Northeast U.S. Shelf: The effect of climate change on Rainbow Smelt is very likely to be negative (>95% certainty in expert scores). Rainbow Smelt are found in the colder coastal habitats in the region. As these habitats warm, productivity may decrease and distributions may shift northwards. Increased precipitation and sea-level rise may also negatively impact Rainbow Smelt through changes in their coastal habitats. The effect of ocean acidification over the next 30 years is likely to be minimal.

Data Quality: 83% of the data quality scores were 2 or greater indicate that data quality is moderate.

Climate Effects on Abundance and Distribution: There is relatively little information on the effect of climate on anadromous Rainbow Smelt. In one study, Mingelbier et al. (2001) found that catch of Rainbow Smelt in the St. Lawrence estuary was linked to air temperature and water level. Population-scale differences in the effect of climate variables were explained by the interaction between climate factors and adult habitat – shallow shoals and deep channels.

Life History Synopsis**:** Rainbow Smelt is a pelagic, anadromous species from the Arctic Ocean, the northern Pacific Ocean, and from southern Labrador to New Jersey in the North Atlantic Ocean (Able and Fahay, 2010). While generally an anadromous species, Rainbow Smelt also occur as several land-locked populations. Most of the population reaches maturing at age 2, but maturity is related to size and may occur earlier in the faster growing southern portion (Klein-MacPhee, 2002). Adults move from near-shore and estuarine waters to freshwater streams to spawn in late winter through spring (Klein-MacPhee, 2002). Males arrive at spawning sites first and remain there longer than females (Klein-MacPhee, 2002). Spawning occurs at night over rock, rubble, sand, or gravel in fast flowing freshwater streams and individuals may spawn in several streams during the season (Klein-MacPhee, 2002). The semi-adhesive eggs are deposited on the bottom and form clusters, sticking to anything they touch (Klein-MacPhee, 2002; Able and Fahay, 2010). Adults return to saltwater by mid to late spring (Klein-

MacPhee, 2002). Eggs require a fairly long incubation time of 2 weeks to 2 months, determined by temperature, and exposure to even low-salinity water can be fatal (Klein-MacPhee, 2002). Larvae drift passively downstream to brackish water or larger lakes and maintain their position in more turbid parts of the estuary using tidal currents and vertical migrations (Able and Fahay, 2010). Larvae are planktivorous, consuming rotifers, copepods, and other planktonic invertebrates (Klein-MacPhee, 2002). The dominant predators of eggs and early larvae are sticklebacks and trout (Able and Fahay, 2010). Juveniles are found on sea grass beds in shallow near-shore areas (Able and Fahay, 2010). By their first winter, juveniles mix with the estuarine adult population and move to salt water during their first year of life (Klein-MacPhee, 2002). Adults occur in bays, estuaries, coastal waters, and freshwater lakes (Able and Fahay, 2010). Juveniles and adults can produce antifreeze chemicals to withstand freezing water temperatures without migrations, but seek deeper, cooler waters in summer (Able and Fahay, 2010). Adults also aggregate in estuaries in autumn in preparation for the spawning run to freshwater (Klein-MacPhee, 2002). Rainbow Smelt consume many invertebrates (e.g., amphipods, euphausiids, mysids, shrimps, and marine worms; Klein-MacPhee, 2002) and small fish (e.g., Atlantic Herring, Bay Anchovy, Cunner, Sand Lance, Atlantic Silversides, Three-spine Sticklebacks, and river herring; Able and Fahay, 2010). Atlantic Cod, Atlantic Salmon, Lake Trout, Striped Bass, Bluefish, Burbot, Walleye, Yellow Perch, mergansers, cormorants, gulls, and terns are common predators of the species (Klein-MacPhee, 2002; Able and Fahay, 2010). Rainbow Smelt were made a federal species of concern after construction of dams, blocking entrance to suitable spawning habitat, and habitat degradation, mostly due to development, severely reduced anadromous population abundances (Klein-MacPhee, 2002). Smelt are managed on a state-by-state basis to rebuild the anadromous population (Enterline et al., 2012) and as an invasive species in several mid-western United States systems (USGS, 2014).

Literature Cited:

Able KW, Fahay MP. 2010. Ecology of estuarine fishes: temperate waters of the western North Atlantic. Baltimore: The Johns Hopkins University Press; 2010. 566p.

Bradbury IR, Campana SE, Bentzen P. Otolith elemental composition and adult tagging reveal spawning site fidelity and estuarine dependency in rainbow smelt. Mar Ecol Prog Ser. 2008; 368: 255-268. doi:10.3354/meps07583

Enterline CL, Chase BC, Carloni JM, Mills KE. 2012. A Regional Conservation Plan for anadomous rainbow smelt in the U. S. Gulf of Maine. Maine Dept of Marine Resources. Accessed Online (December 2014): http://restorerainbowsmelt.com/wp-content/uploads/2013/01/Smelt-Conservation-Plan-final.pdf

Klein-MacPhee G. Rainbow Smelt/ Osmerus mordax (Mitchill 1814). In: B.B. Collette BB, Klein-MacPhee G, editors, Fishes of the Gulf of Maine, 3rd ed. Washington: Smithsonian Institution Press; 2002. pp. 166-170.

Laprise R, Dodson JJ. Ontogeny and importance of tidal vertical migrations in the retention of larval smelt Osmerus mordax in a well-mixed estuary. Mar Ecol Prog Ser. 1989; 55(2): 101-111. Available: http://www.int-res.com/articles/meps/55/m055p101.pdf

Murawski SA, Clayton GR, Reed RJ, Cole CF. Movements of spawning rainbow smelt, Osmerus mordax, in a Massachusetts estuary. Estuaries, 1980; 3(4): 308-314. doi: 10.2307/1352086

U.S. Geological Survey (USGS). 2014. Nonindigenous Aquatic Species Database. Gainesville, Florida. Accessed online (December 2014): http://nas.er.usgs.gov/queries/factsheet.aspx?SpeciesID=796

[Return to Table of Contents]

Red Drum – *Sciaenops ocellatus*

Overall Vulnerability Rank = High

Biological Sensitivity = Moderate
Climate Exposure = Very High
Data Quality = 88% of scores ≥ 2

Sciaenops ocellatus	Expert Scores	Data Quality	Expert Scores Plots (Portion by Category)
Stock Status	2.1	1.6	
Other Stressors	2.4	2.8	
Population Growth Rate	2.9	3.0	
Spawning Cycle	2.6	3.0	
Complexity in Reproduction	1.5	2.8	
Early Life History Requirements	2.8	2.5	
Sensitivity to Ocean Acidification	1.8	3.0	
Prey Specialization	1.4	3.0	
Habitat Specialization	2.0	3.0	
Sensitivity to Temperature	1.5	2.8	
Adult Mobility	1.6	2.6	
Dispersal & Early Life History	2.2	2.6	
Sensitivity Score	Moderate		
Sea Surface Temperature	4.0	3.0	
Variability in Sea Surface Temperature	1.0	3.0	
Salinity	2.8	3.0	
Variability Salinity	1.2	3.0	
Air Temperature	4.0	3.0	
Variability Air Temperature	1.0	3.0	
Precipitation	1.2	3.0	
Variability in Precipitation	1.3	3.0	
Ocean Acidification	4.0	2.0	
Variability in Ocean Acidification	1.0	2.2	
Currents	2.0	1.0	
Sea Level Rise	3.3	1.5	
Exposure Score	Very High		
Overall Vulnerability Rank	High		

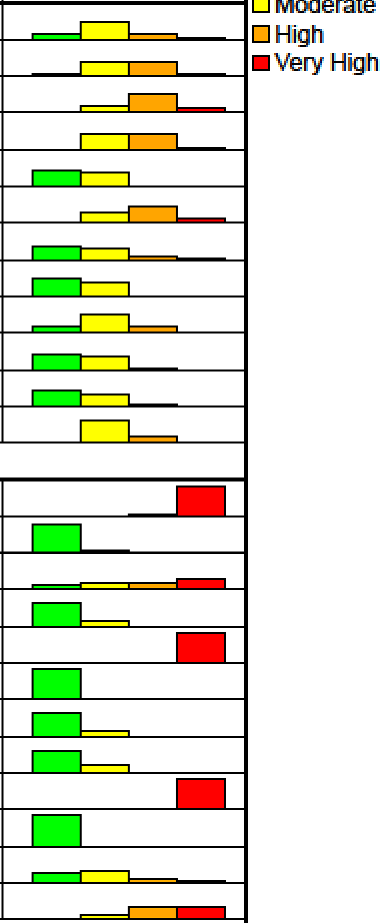

Return to Table of Contents

Red Drum (*Sciaenops ocellatus*)

Overall Climate Vulnerability Rank: **High** (48% certainty from bootstrap analysis).

Climate Exposure: **Very High**. Three exposure factors contributed to this score: Ocean Surface Temperature (4.0), Ocean Acidification (4.0) and Air Temperature (4.0). Exposure to all three factors occur during all life stages. Red Drum spend most of their life cycle in coastal waters.

Biological Sensitivity: **Moderate**. Three sensitivity attributes scored above 2.5: Population Growth Rate (2.9), Spawning Cycle (2.6), and Early Life History Requirements (2.8). Red Drum are long-lived and relatively slow growing for a sciaenid. They spawn in late-summer and early-autumn in coastal areas including inlets. Early Life Stages remain in coastal areas and are rarely observed in shelf waters. Juveniles use structured habitats in coastal and estuarine waters.

Distributional Vulnerability Rank: **Moderate** (42% certainty from bootstrap analysis). Only one attribute indicated vulnerability to distribution shift. Red Drum are capable of moving long distances and make seasonal migrations in colder portions of their range.

Directional Effect in the Northeast U.S. Shelf: The effect of climate change on Red Drum on the Northeast U.S. Shelf is estimated to be positive, but this estimate is uncertain (<66% certainty in expert scores). Overwinter mortality may be an important component of the population dynamics and warming winters would increase recruitment. The species is more common in the south and thus there is the expectation that as warming occurs more areas on the Northeast U.S. shelf will be thermally suitable. The effect of ocean acidification over the next 30 years is likely to be minimal.

Data Quality: 88% of the data quality scores were 2 or greater.

Climate Effects on Abundance and Distribution: The productivity of Red Drum is likely related to climate. In laboratory experiments, Anderson and Scharf (2012) indicated that severe winters may cause high mortality of young-of-year Red Drum independent of body size, whereas smaller young-of-the-year are more susceptible to mortality during moderate winters. The over-winter mortality hypothesis for Red Drum is very similar to that for Atlantic Croaker (Hare and Able, 2007). Rooker et al. (1998) found that predation on young-of-the-year Red Drum was lower in vegetated compared to non-vegetated habitats suggesting potential susceptibility to sea-level rise and loss of vegetated habitats in estuaries and coastal areas. Little work has been done examining potential climate effects on Red Drum distribution.

Life History Synopsis**:** Red Drum is a large, long-lived, oligohaline, coastal and estuarine fish species found historically from Long Island, New York to the western Gulf of Mexico, but rare north of Chesapeake Bay in recent years (Able and Fahay, 2010). Males mature earlier, at 1-3 years, than females, at 3-6 years (ASMFC, 2013). Spawning occurs in near-shore and high-salinity estuarine areas such as along beaches, near the mouths of estuaries, and at the mouth of large embayments during summer-fall as temperatures drop (Able and Fahay, 2010; ASMFC, 2013). Eggs and larvae are pelagic in near-shore and bay-mouth areas, but rarely collected in shelf waters (Able and Fahay, 2010). After about 3 weeks, larvae settle to lower salinity nursery areas of the upper estuary where they transform into the juvenile stage around 10 mm standard length (Able and Fahay, 2010; ASMFC, 2013). Juveniles become increasingly tolerant of freshwater as they grow and can occur in a wide range of salinities from tidal freshwater to coastal marine water. They generally reside in estuarine water over mud, sand, and oyster bars (Able and Fahay, 2010; ASMFC, 2013). Juveniles in the northern population may migrate to

deeper water during winter and move to coastal water incrementally from age 1-3 years (ASMFC, 2013). Juveniles are predators of zooplankton, particularly amphipods and mysids (Able and Fahay, 2010). Adult Red Drum can tolerate a wide range of salinities from freshwater to the high salinities of the Gulf of Mexico, but generally occur in high-salinity surf zones and on natural and artificial reefs and structure (ASMFC, 2013). The northern population (North Carolina to New Jersey) makes seasonal migrations from the North Carolina shelf in winter to estuaries from North Carolina to Chesapeake Bay in late spring to early fall (historically going as far north as Massachusetts; Able and Fahay, 2010; ASMFC, 2013). The southern (South Carolina to Florida) population makes cross-shelf seasonal migrations to deeper shelf water in the winter and estuaries in the summer (ASMFC, 2013). The adult diet becomes increasingly dependent on large crustaceans and small fishes with growth, and the largest Red Drum rely almost exclusively on crustaceans (Able and Fahay, 2010). There are few known predators on adults, but the bottlenose dolphin has been known to eat Red Drum (Able and Fahay, 2010). The population dynamics and habitat use of adults has been poorly studied, making management difficult. The Atlantic States Marine Fisheries Commission manages Red Drum as two Atlantic stocks (northern: New Jersey to North Carolina; southern: South Carolina to Florida). Based on the 2009 stock assessment, neither population is believed to be undergoing overfishing, but stock status could not be reliably determined due to the high degree of uncertainty in the assessment (SEDAR, 2009).

Literature Cited:

Able KW, Fahay MP. Ecology of estuarine fishes: temperate waters of the western North Atlantic. Baltimore: The Johns Hopkins University Press; 2010. 566p.

Anderson DA, Scharf FS. The effect of variable winter severity on size-dependent overwinter mortality caused by acute thermal stress in juvenile red drum (Sciaenops ocellatus). ICES J Mar Sci. 2013; fst041. doi: 10.1093/icesjms/fst041

Atlantic States Marine Fisheries Commission (ASMFC). 2013. Addendum I to Amendment 2 to the Red Drum fishery management plan: habitat needs and concerns. Accessed online (May 2015): http://www.asmfc.org/species/red-drum

Hare JA, Able KW. Mechanistic links between climate and fisheries along the east coast of the United States: explaining population outbursts of Atlantic croaker (Micropogonias undulatus). Fisheries Oceanography, 2007; 16(1): 31-45. doi: 10.1111/j.1365-2419.2006.00407.x

Rooker JR, Holt GJ, Holt SA. Vulnerability of newly settled red drum (Sciaenops ocellatus) to predatory fish: is early-life survival enhanced by seagrass meadows? Mar Biol. 1998; 131(1): 145-151. doi: 10.1007/s002270050305

Southeast Data, Assessment, and Review (SEDAR). 2009. SEDAR 18 Atlantic Red Drum Stock Assessment Report. Accessed online (May 2015): http://sedarweb.org/sedar-18

Return to Table of Contents

Red Hake – *Urophycis chuss*

Overall Vulnerability Rank = Low

Biological Sensitivity = Low
Climate Exposure = High
Data Quality = 88% of scores ≥ 2

Urophycis chuss	Expert Scores	Data Quality	Expert Scores Plots (Portion by Category)
Stock Status	1.1	3.0	
Other Stressors	1.9	2.6	
Population Growth Rate	1.7	2.4	
Spawning Cycle	2.0	3.0	
Complexity in Reproduction	1.7	2.8	
Early Life History Requirements	2.3	2.0	
Sensitivity to Ocean Acidification	1.8	2.4	
Prey Specialization	1.2	3.0	
Habitat Specialization	1.8	3.0	
Sensitivity to Temperature	1.8	3.0	
Adult Mobility	1.4	2.8	
Dispersal & Early Life History	2.5	1.6	
Sensitivity Score	Low		
Sea Surface Temperature	3.9	3.0	
Variability in Sea Surface Temperature	1.0	3.0	
Salinity	1.7	3.0	
Variability Salinity	1.2	3.0	
Air Temperature	1.0	3.0	
Variability Air Temperature	1.0	3.0	
Precipitation	1.0	3.0	
Variability in Precipitation	1.0	3.0	
Ocean Acidification	4.0	2.0	
Variability in Ocean Acidification	1.0	2.2	
Currents	2.1	1.0	
Sea Level Rise	1.1	1.5	
Exposure Score	High		
Overall Vulnerability Rank	Low		

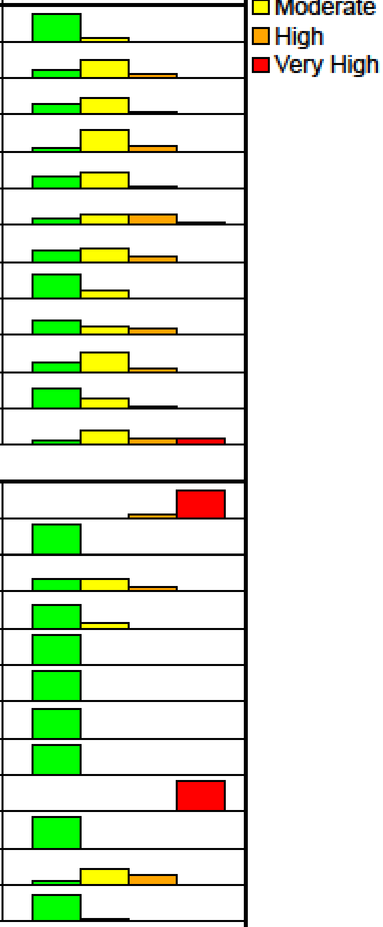

Return to Table of Contents

Red Hake (*Urophycis chuss*)

Overall Climate Vulnerability Rank: **Low** (94% certainty from bootstrap analysis).

Climate Exposure: **High**. Two exposure factors contributed to this score: Ocean Surface Temperature (3.9) and Ocean Acidification (4.0). All life stages of Red Hake use marine habitats.

Biological Sensitivity: **Low**. Only one sensitivity attributes scored above 2.5. Dispersal of early life stages may be partially limited as juveniles are commensal with Atlantic Sea Scallops (Steiner et al., 1982). This relationship with Atlantic Sea Scallops is likely facultative.

Distributional Vulnerability Rank: **High** (94% certainty from bootstrap analysis). Adults are habitat generalists and mobile. Eggs and larvae are planktonic.

Directional Effect in the Northeast U.S. Shelf: The effect of climate change on Red Hake on the Northeast U.S. Shelf is estimated to be neutral, but with a moderate degree of uncertainty (66-90% certainty in expert scores). Red Hake is a temperate fish and warming in the Northeast U.S. seems to have made more of the Gulf of Maine available to the species. Abundance in the southern portions of the system may be decreasing, but the region-wide affects are unclear.

Data Quality: 88% of the data quality scores were 2 or greater indicate that data quality is moderate.

Climate Effects on Abundance and Distribution: There is little information regarding the effect of climate on productivity, but the abundance of Red Hake has decreased in Narragansett Bay with increasing temperatures (Collie et al., 2008). Distribution has also changed dramatically in recent years with the population shifting northwards (Nye et al., 2009). Further, Murawski (1993) indicated that Red Hake distribution changed in response to temperature.

Life History Synopsis**:** Red hake is a marine, demersal species found from the Gulf of St. Lawrence to North Carolina, but is most abundant from the western Gulf of Maine through southern New England (NEFSC, 2011). Red Hake reach maturity around 1.4 (males) and 1.8 years (females; NEFSC, 2011), with females generally older and larger than males (Steimle et al., 1999). Spawning occurs from April through November (July to November in the Gulf of Maine) on the continental shelf and in coastal embayments (Steimle et al., 1999). Spawning in the Mid-Atlantic Bight may produce the majority of recruits (Steimle et al., 1999). Within a week from spawning, buoyant eggs hatch into small pelagic larvae that prey on copepods and other small planktonic crustaceans (Steimle et al., 1999). Larvae transition into pelagic juveniles at approximately 20-30 mm standard length (Fahay, 2007) and remain pelagic for approximately 2 months relying on floating debris, sargassum, and jellyfish tentacles for shelter (Steimle et al., 1999). By 35-40 mm total length, Red Hake begin a gradual descent to the benthos. They settle on fine-sand sediment on the shelf, and in larger estuaries in areas such as Sea Scallop beds, depressions in open seabeds, Atlantic surfclam shells, Moon Snail egg-case collars, anemone and polychaete tubes, debris, and artificial reefs (Steimle et al., 1999). Settlement occurs in September to December, but a strong thermocline may delay descent (Steimle et al., 1999). Throughout the juvenile stage, Red Hake prey on small crustaceans including larval and small decapod shrimp and crabs, mysids, euphausiids, and amphipods (Steimle et al., 1999). Red Hake are mostly demersal, but can be found in the water column. They tolerate a large range of temperatures, but may be sensitive to low dissolved oxygen levels (Steimle et al., 1999). Like juveniles, adult hake prefer soft sediments and use depressions in the sediment, shell beds, and inshore reefs (natural and artificial) for shelter, and are rarely found in open

sandy bottom (Steimle et al., 1999). Red Hake make seasonal migrations influenced by temperature, preferring inshore habitat during warm months, and offshore habitats during colder months (Steimle et al., 1999). Adult hake prey upon crustaceans, demersal and pelagic fish, and squid (Steimle et al., 1999). Predators on adult and juvenile hake include many large piscivores such as Striped Bass, Spiny Dogfish, Monkfish (Goosefish), other hake species, Sea Raven, harbor porpoise, and larger Red Hake (Steimle et al., 1999). Red Hake are managed as two stocks: northern Georges – Gulf of Maine and southern Georges Bank – Mid-Atlantic Bight region as part of the Northeast Fishery Management Council's Northeast Small Mesh Multispecies Fishery Management Plan (NEFSC, 2011; NEFMC, 2012). Based on the 2010 stock assessment, neither stock is overfished nor undergoing overfishing (NEFSC, 2011).

Literature Cited:

Collie JS, Wood AD, Jeffries HP. Long-term shifts in the species composition of a coastal fish community. Canadian J Fish Aquat Sci. 2008; 65(7): 1352-1365. DOI: 10.1139/F08-048

Fahay MP. 2007. Early Stages of Fishes in the western North Atlantic Ocean (Davis Strait, Southern Greenland and Flemish Cap to Cape Hatteras). Accessed Online (August 2015): http://www.nafo.int/publications/fahay/pdfs.html

Murawski SA. Climate change and marine fish distributions: forecasting from historical analogy. Trans Amer Fish Soc. 1993; 122(5): 647-658. DOI: 0.1577/1548-8659(1993)122<0647:CCAMFD>2.3.CO;2

New England Fishery Management Council (NEFMC). Final amendment 19 to the Northeast Multispecies FMP (Small-mesh multi species) Environmental Assessment regulatory impact review and initial regulatory flexibility analysis. Newburyport, MA. 2012; Accessed online (August 2015): http://archive.nefmc.org/mesh/planamen/Amend%2019/Final%20Amendment%2019%20revised.pdf

Northeast Fisheries Science Center (NEFSC). 51st Northeast Regional Stock Assessment Workshop (51st SAW) Assessment Report. US Dept Commer, Northeast Fish Sci Cent Ref Doc. 2011; 11-02: 856 p. Accessed Online (August 2014): http://www.nefsc.noaa.gov/publications/crd/crd1102/

Nye JA, Link JS, Hare JA, Overholtz WJ. Changing spatial distribution of fish stocks in relation to climate and population size on the Northeast United States continental shelf. Mar Ecol Prog Ser. 2009; 393: 111-129. DOI: 0.3354/meps08220

Steimle FW, Morse WW, Berrien PL, Johson DL. 1999. Essential fish habitat source document: Red Hake, *Urophycis chuss*, life history and habitat characteristics. NOAA Tech Memo NMFS NE 1999; 133: 34 p. Accessed Online (August 2015): http://www.nefsc.noaa.gov/nefsc/publications/tm/tm133/

Steiner WW, Luczkovich JJ, Olla BL. Activity, shelter usage, growth and recruitment of juvenile Red Hake *Urophycis chuss*. Mar Ecol Prog Ser. 1982; 7(2): 125-135. Accessed Online (August 2015): http://www.int-res.com/articles/meps/7/m007p125.pdf

Return to Table of Contents

Rosette Skate – *Leucoraja garmani*

Overall Vulnerability Rank = Moderate

Biological Sensitivity = Moderate
Climate Exposure = High
Data Quality = 75% of scores ≥ 2

Leucoraja garmani	Expert Scores	Data Quality	Expert Scores Plots (Portion by Category)
Stock Status	3.0	2.8	
Other Stressors	1.4	1.0	
Population Growth Rate	2.9	0.6	
Spawning Cycle	1.3	2.2	
Complexity in Reproduction	1.5	1.6	
Early Life History Requirements	1.0	2.8	
Sensitivity to Ocean Acidification	1.4	2.2	
Prey Specialization	1.1	2.8	
Habitat Specialization	1.2	2.8	
Sensitivity to Temperature	1.2	2.8	
Adult Mobility	2.4	1.4	
Dispersal & Early Life History	1.9	3.0	
Sensitivity Score	Moderate		
Sea Surface Temperature	3.9	3.0	
Variability in Sea Surface Temperature	1.0	3.0	
Salinity	2.7	3.0	
Variability Salinity	1.2	3.0	
Air Temperature	1.0	3.0	
Variability Air Temperature	1.0	3.0	
Precipitation	1.0	3.0	
Variability in Precipitation	1.0	3.0	
Ocean Acidification	4.0	2.0	
Variability in Ocean Acidification	1.0	2.2	
Currents	2.1	1.0	
Sea Level Rise	1.1	1.5	
Exposure Score	High		
Overall Vulnerability Rank	Moderate		

Low
Moderate
High
Very High

Return to Table of Contents

Rosette Skate (*Leucoraja garmani*)

Overall Climate Vulnerability Rank: **Moderate** (78% certainty from bootstrap analysis).

Climate Exposure: **High**. Two exposure factors contributed to this score: Ocean Surface Temperature (4.0) and Ocean Acidification. Rosette Skate are demersal and complete their life cycle in marine habitats.

Biological Sensitivity: **Moderate**. Two sensitivity attributes scored above 2.5: Stock Status (3.0) and Population Growth Rate (2.9). In 2013, based on trawl survey indices Rosette Skate was above the biomass threshold but below the biomass target. Further, the index has been declining since the early 2000s (NEFSC, 2013). There is a high degree of uncertainty in Population Growth, but skates in general have a low population growth rate (Frisk, 2010).

Distributional Vulnerability Rank: **High** (83% certainty from bootstrap analysis). Rosette Skate are habitat generalists and moderately mobile as adults, making seasonal movements. In addition, skate egg cases are subject to movement by currents and juveniles may move on scales of 1-10 km.

Directional Effect in the Northeast U.S. Shelf: The effect of climate change on Rosette Skate is estimated to be neutral, but this estimate has high uncertainty (<66% certainty in expert scores). Rosette Skate inhabits temperate waters and may benefit from warming on the Northeast U.S. Shelf. But ocean acidification may reduce productivity and no changes in distribution have been observed over the past 30 years despite significant warming.

Data Quality: 75% of the data quality scores were 2 or greater indicate that data quality is moderate.

Climate Effects on Abundance and Distribution: Little specific information exists on the effect of climate on Rosette Skate. Di Santo (2015) found that increased warming and acidification reduce body condition of newly hatched Little Skate – a congener. These reductions in size could result in reduced juvenile survival and thus recruitment. In regional studies of distribution, Rosette Skate was not included (Murawski, 1993; Nye et al., 2009) but examination of NEFSC trawl survey data suggests no change in the center of the distribution over the last 30 years (http://oceanadapt.rutgers.edu/, website last checked 13 June 2015).

Life History Synopsis**:** Rosette skate is a benthic, marine elasmobranch found from Nantucket Shoals to the Dry Tortugas, Florida, but the population south of Cape Hatteras, North Carolina, may be a separate species (Packer et al., 2003). North of Cape Hatteras, Rosette Skates mature between 33-43 cm total length, which is estimated to be about 4 years of age and mate using internal fertilization (Packer et al., 2003; Sosebee, 2005). Like most skates, single eggs are encased in a horned capsule; females produce egg capsules year-round, but peak production occurs during summer (Packer et al., 2003). There is no larval stage. Juvenile Rosette Skates inhabit the outer continental shelf and mostly occur in the mid-Atlantic region, but are occasionally recorded in the Gulf of Maine (Packer et al., 2003). Adults are found on soft-bottom habitat on the outer shelf and upper slope and prefer moderate water temperatures (Packer et al., 2003). Rosette Skates prey upon Crangon Shrimp, Cancer and galatheoid crabs, amphipods, polychaetes, copepods, cumaceans, squids, octopods, and small fishes (Packer et al., 2003). The New England Fishery Management Council manages the species as part of a Northeast Skate Complex. Rosette Skates are not overfished and overfishing is not occurring (NEFSC, 2007).

Literature Cited:

Di Santo V. Ocean acidification exacerbates the impacts of global warming on embryonic little skate, *Leucoraja erinacea* (Mitchill). J Exp Mar Bio Ecol. 2015; 463: 72-78. doi:10.1016/j.jembe.2014.11.006

Frisk MG. Life history strategies of batoids. In: Carrier, JC, Musick, JA, Heithaus, MR, editors, Sharks and Their Relatives II: Biodiversity, Adaptive Physiology, and Conservation. Boca Raton: CRC Press; 2010. 283-318.

Murawski SA. Climate change and marine fish distributions: forecasting from historical analogy. Trans Am Fish Soc. 1993; 122(5): 647-658. doi: 10.1577/1548-8659(1993)122<0647:CCAMFD>2.3.CO;2

Northeast Fisheries Science Center (NEFSC). 2013. Update of Skate Stock Status Based on NEFSC Bottom Trawl Survey Data through Autumn 2012 and Spring 2013. Available: http://www.nefsc.noaa.gov/program_review/background2014/TOR3TercerioSkate%20StocksStatusUpdate%202013.pdf

Northeast Fisheries Science Center (NEFSC). 2007. 44th Northeast Regional Stock Assessment Workshop (44th SAW): 44th SAW assessment report. US Dep Commer, Northeast Fish Sci Cent Ref Doc 07-10; 661 p. Accessed online (August 2015): http://www.nefsc.noaa.gov/publications/crd/crd0710/

Nye JA, Link JS, Hare JA, Overholtz WJ. Changing spatial distribution of fish stocks in relation to climate and population size on the Northeast United States continental shelf. Mar Ecol Prog Ser. 2009; 393: 111-129. doi: 10.3354/meps08220

Packer DB, Zetlin CA, Vitaliano JJ. 2003. Essential fish habitat source document: Rosette skate, Leucoraja garmani virginica, life history and habitat characteristics. NOAA Tech Memo NMFS NE 176; 17 p. Accessed online (August 2015): http://www.nefsc.noaa.gov/nefsc/publications/tm/tm176/

Sosebee KA. Maturity of Skates in Northeast United States Waters. J Northw Atl Fish Sci. 2205; 35: 141-153. doi: doi:10.2960/J.v35.m499

Sand Lances – *Ammodytes spp.*

Overall Vulnerability Rank = Moderate

Biological Sensitivity = Moderate
Climate Exposure = High
Data Quality = 88% of scores ≥ 2

Ammodytes spp.	Expert Scores	Data Quality	Expert Scores Plots (Portion by Category)
Stock Status	1.7	1.2	
Other Stressors	2.0	2.0	
Population Growth Rate	1.4	2.8	
Spawning Cycle	2.7	2.4	
Complexity in Reproduction	1.7	2.2	
Early Life History Requirements	2.3	2.0	
Sensitivity to Ocean Acidification	1.4	2.4	
Prey Specialization	1.3	2.5	
Habitat Specialization	2.0	2.6	
Sensitivity to Temperature	2.5	2.4	
Adult Mobility	2.8	2.4	
Dispersal & Early Life History	1.6	2.6	
Sensitivity Score	Moderate		
Sea Surface Temperature	4.0	3.0	
Variability in Sea Surface Temperature	1.0	3.0	
Salinity	1.8	3.0	
Variability Salinity	1.2	3.0	
Air Temperature	2.7	3.0	
Variability Air Temperature	1.0	3.0	
Precipitation	1.1	3.0	
Variability in Precipitation	1.1	3.0	
Ocean Acidification	4.0	2.0	
Variability in Ocean Acidification	1.0	2.2	
Currents	2.2	1.0	
Sea Level Rise	2.7	1.5	
Exposure Score	High		
Overall Vulnerability Rank	Moderate		

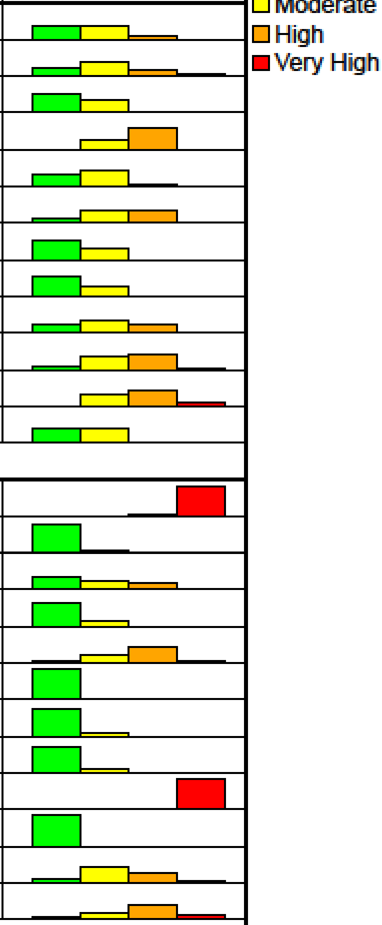

Sand Lances (*Ammodytes americanus / Ammodytes dubius*)

Overall Climate Vulnerability Rank: **Moderate** (97% certainty from bootstrap analysis).

Climate Exposure: **High**. Two exposure factors contributed to this score: Ocean Surface Temperature (4.0) and Ocean Acidification (4.0). Exposure to ocean surface temperature and ocean acidification occurs during all life stages.

Biological Sensitivity: **Moderate**. The highest sensitivity attributes were Adult Mobility (2.8), Spawning Cycle (2.7), and Sensitivity to Temperature (2.5). Sand Lances are associated with sandy habitats where they seek shelter and overwinter in the sand (aestivation) (Wright et al., 2000). This association with sandy habitats limits adult mobility. Sand Lance spawn in a distinct season: late winter/early spring and the cycle of feeding, aestivation, and spawning is related to temperature (Tomiyama and Yanagibahi, 2004).

Distributional Vulnerability Rank: **Moderate** (45% certainty from bootstrap analysis). Only one attribute indicated vulnerability to distribution shift: Sand Lance have dispersive early life history stages. A shift in distribution is limited by restricted adult mobility, specific requirements of adults for sand habitats of specific grain sizes, and a moderate sensitivity in the timing of the life cycle to temperature.

Directional Effect in the Northeast U.S. Shelf: The effect of climate change on Sand Lance on the Northeast U.S. Shelf is estimated to be negative, but this estimate is uncertain (66-90% certainty in expert scores). Higher temperatures may decrease productivity and limit habitat availability for Sand Lance. But Sand Lance require specific habitat types defined by grained size creating uncertainty as to the effect of climate change on adult distributions.

Data Quality: 88% of the data quality scores were 2 or greater.

Climate Effects on Abundance and Distribution: In the Northeast Atlantic, Sand Lances are identified as particularly at risk from climate change owing to the strict association with coarse sandy sediments; which in some regions represents limited habitat (Heath et al., 2012). Further, Arnott and Ruxton (2002) found a negative correlation between warmer sea temperatures and recruitment. Wanless et al. (2000) suggested that changing environmental conditions may have contributed to the long-term decline in size of age-0 in the North Sea. They hypothesize that changes in spawning time and or growth could have resulted in the decrease in size. These studies suggest a potential effect of climate change on population productivity. In the Northwest Atlantic, Richardson et al. (2014) documents an alternation between Atlantic Herring and Sand Lances; the cause of this alternation is uncertain.

Life History Synopsis**:** Sand Lance are schooling and burrowing fish species found along the Atlantic continental shelf. There are two species of Sand Lance in the northwest Atlantic from Greenland to Cape Hatteras, North Carolina, that are so morphologically similar that they are rarely identified to species (Nizinski, 2002). Sand Lance mature at 2 years of age, and spawn in late fall through early spring over sandy bottom across the shelf (Nizinski, 2002). The cross-shelf range of spawning habitat is likely species specific (an inshore species and an offshore species), but the exact spawning locations of either species are poorly known (Able and Fahay, 2010). Eggs are demersal and adhesive and probably have a long incubation period of 1-2 months (Nizinski, 2002). Inter-annual abundance of larvae entering New Jersey estuaries varied little over almost 2 decades of sampling (Able and Fahay, 2010). Larvae are widespread during winter and spring on the shelf and into estuaries consuming mostly phytoplankton

and copepods (Nizinski, 2002; Able and Fahay, 2010). Larvae begin schooling 3 months after hatching and are fully metamorphosed and begin burrowing after 4-5 months (Able and Fahay, 2010). As larvae mature they include a more diverse group of invertebrates in their diet, including copepods, crabs, cirripedes, bivalves, gastropods, cnidarians, mysid shrimp, decapod shrimp, cladocerans, phytoplankton, and occasional fish (Able and Fahay, 2010). Juvenile and adult Sand Lance burrow into sandy substrates of very shallow coastal to deep shelf waters (Able and Fahay, 2010). *Ammodytes americanus* usually occurs farther inshore and into estuaries than *A. dubius*, but there is considerable overlap and an obligatory association with sand habitat (Nizinski, 2002; Richardson et al., 2014). The primary predators of Sand Lance are terns, cormorant, and a variety of fishes, including: Little Skate, Monkfish (Goosefish), Atlantic Cod, Haddock, Atlantic Halibut, Silver Hake, Red Hake, Atlantic Salmon, Atlantic Mackerel, Striped Bass, Bluefish, Pollock, American Plaice, Yellowtail Flounder, and Gulf Stream Flounder (Nizinski, 2002). Sand Lance are not currently managed in United States waters, but oscillations between Sand Lance and Atlantic Herring abundance and their link to Atlantic Cod distributions may make them an important part of an ecosystem-based management plan (Richardson et al., 2014). Sand Lance are heavily exploited in other regions of the world (Furness 2002) but are not currently exploited in the Northeast U.S. Shelf.

Literature Cited:

Able KW, Fahay MP. Ecology of estuarine fishes: temperate waters of the western North Atlantic. Baltimore: The Johns Hopkins University Press; 2010. 566p.

Arnott SA, Ruxton GD. Sandeel recruitment in the North Sea: demographic, climatic and trophic effects. Mar Ecol Prog Ser. 2002; 238: 199-210. doi: 10.3354/meps238199

Furness RW. Management implications of interactions between fisheries and sandeel-dependent seabirds and seals in the North Sea. ICES J Mar Sci. 2002; 59(2): 261-269. doi: 10.1006/jmsc.2001.1155

Heath MR, Neat FC, Pinnegar JK, Reid DG, Sims DW, Wright PJ. Review of climate change impacts on marine fish and shellfish around the UK and Ireland. Aquatic Conservation: Marine and Freshwater Ecosystems, 2012; 22(3): 337-367. doi: 10.1002/aqc.2244

Nizinski MS. Sand Lances. Family Ammodytidae. In: B.B. Collette BB, Klein-MacPhee G, editors, Fishes of the Gulf of Maine, 3rd ed. Washington: Smithsonian Institution Press; 2002. pp. 496-505.

Richardson, DE, Palmer MC, Smith BE. The influence of forage fish abundance on the aggregation of Gulf of Maine Atlantic cod (*Gadus morhua*) and their catchability in the fishery. Can J Fish Aquat Sci. 2014; 71: 1349-1362. doi: 10.1139/cjfas-2013-0489

Tomiyama M, Yanagibashi S. Effect of temperature, age class, and growth on induction of aestivation in Japanese sandeel (*Ammodytes personatus*) in Ise Bay, central Japan. Fish Oceanogr. 2004; 13(2): 81-90. doi: 10.1046/j.1365-2419.2003.00272.x

Wanless S, Wright PJ, Harris MP, Elston DA. Evidence for decrease in size of lesser sandeels *Ammodytes marinus* in a North Sea aggregation over a 30-yr period. Mar Ecol Prog Ser. 2004; 279: 237-246. doi: 10.3354/meps279237

Wright PJ, Jensen H, Tuck I. The influence of sediment type on the distribution of the lesser sandeel, *Ammodytes marinus*. J Sea Res. 2000; 44(3): 243-256. doi: 10.1016/S1385-1101(00)00050-2

Return to Table of Contents

Sand Tiger – *Carcharias taurus*

Overall Vulnerability Rank = High

Biological Sensitivity = High
Climate Exposure = High
Data Quality = 79% of scores ≥ 2

Carcharias taurus	Expert Scores	Data Quality	Expert Scores Plots (Portion by Category)
Stock Status	3.1	1.8	
Other Stressors	1.8	1.8	
Population Growth Rate	3.8	2.6	
Spawning Cycle	1.8	2.4	
Complexity in Reproduction	1.8	1.8	
Early Life History Requirements	1.0	3.0	
Sensitivity to Ocean Acidification	1.0	2.4	
Prey Specialization	1.3	2.8	
Habitat Specialization	1.3	3.0	
Sensitivity to Temperature	1.3	3.0	
Adult Mobility	1.0	3.0	
Dispersal & Early Life History	1.0	3.0	
Sensitivity Score	High		
Sea Surface Temperature	3.9	3.0	
Variability in Sea Surface Temperature	1.0	3.0	
Salinity	2.8	3.0	
Variability Salinity	1.2	3.0	
Air Temperature	1.0	3.0	
Variability Air Temperature	1.0	3.0	
Precipitation	1.0	3.0	
Variability in Precipitation	1.0	3.0	
Ocean Acidification	4.0	2.0	
Variability in Ocean Acidification	1.0	2.2	
Currents	2.1	1.0	
Sea Level Rise	1.2	1.5	
Exposure Score	High		
Overall Vulnerability Rank	High		

- Low
- Moderate
- High
- Very High

Return to Table of Contents

Sand Tiger (*Carcharias taurus*)

Overall Climate Vulnerability Rank: **High** (76% certainty from bootstrap analysis).

Climate Exposure: **High**. Two exposure factors contributed to this score: Ocean Surface Temperature (3.9) and Ocean Acidification (4.0). Sand Tiger Shark are pelagic but associate with benthic habitats.

Biological Sensitivity: **High**. Two attributes scored above 3.0: Population Growth Rate (3.8) and Stock Status (3.1). Sand Tigers have a moderate population growth rate for a shark, but this is still low compared to many other marine species (Smith et al., 1998). Sand Tiger were identified as a Species of Concern by the U.S. in 2004 owing to historic declines in abundance and low productivity. Even though a status review indicated that declines in abundance were not as severe as originally reported, the Sand Tiger remains on the Species of Concern list due to the uncertainty in abundance estimates and their exceptionally low productivity (Carlson et al. 2009; http://www.nmfs.noaa.gov/pr/pdfs/species/sandtigershark_detailed.pdf)

Distributional Vulnerability Rank: **Very High** (94% certainty from bootstrap analysis). Sand Tigers are habitat generalists and highly mobile. In addition, Sand Tigers are ovoviviparous and do not have planktonic eggs and larval stages.

Directional Effect in the Northeast U.S. Shelf: The effect of climate change on Sand Tiger is very likely to be neutral (>95% certainty in expert scores). Sand Tiger is a highly mobile temperate shark. There is very little information available that suggests negative or positive effects of climate change.

Data Quality: 79% of the data quality scores were 2 or greater indicate that data quality is moderate.

Climate Effects on Abundance and Distribution: There is very little information on the effect of climate change on Sand Tigers.

Life History Synopsis**:** The Sand Tiger is a highly mobile, coastal shark species that occurs in United States waters from the Gulf of Maine to Florida and in the northern Gulf of Mexico (Carlson et al., 2009). Males reach maturity at a length of 190-195 cm TL between 6-7 years of age; females mature later (9-10 years) at 220-230 cm TL (Gilmore et al., 1983; Goldman et al., 2006; Carlson et al., 2009). Evidence suggests a 2-year reproductive cycle for Sand Tigers in the western North Atlantic, with gestation likely taking a year followed by a year resting period (Branstetter, 2002; Carlson et al., 2009). Multiple batches of eggs are produced and encapsulated, but typically only one capsule is fertilized (Branstetter, 2002). The additional eggs and eventually smaller siblings provide nourishment for the largest pups, resulting in one or two pups born each year (Branstetter, 2002; Carlson et al., 2009). Juveniles occupy shallow estuarine and coastal nursery habitats between Massachusetts and North Carolina during late spring and summer (McCandless et al., 2007). Adults occur off Florida and in the Gulf of Mexico year round, and venture as far north as the Gulf of Maine during summer and fall months (Branstetter, 2002; Carlson et al., 2009). Both Juveniles and adults are generalist predators that consume lobsters, crabs, and squids, but prey primarily on fishes such as Summer Flounder, skates, Monkfish (Goosefish), sea robins, Scup, Spot, Bluefish, Butterfish, and Tautog (Branstetter, 2002). There are not many predators of this large, demersal shark, but there is evidence of cannibalism (Branstetter, 2002). Sand Tigers were targeted and a frequent bycatch species until regulations prohibited landing the species from federal and state waters ((NMFS 1999; ASMFC 2008; Carlson et al., 2009). The Atlantic States Marine Fisheries Commission manages Sand Tigers through an interstate fishery management plan and NMFS manages

them through the Consolidated Atlantic Highly Migratory Species Fishery Management Plan (ASMFC 2008; NMFS 2006)

Literature Cited:

ASMFC. 2008. Interstate fishery management plan for Atlantic coastal sharks. Fishery Management Report No. 46. Accessed online (August 2015): http://www.asmfc.org/uploads/file/interstateFMPforAtlanticCoastalSharks.pdf

Branstetter S. Sand Tiger/ Carcharias taurus. In: B.B. Collette BB, Klein-MacPhee G, editors, Fishes of the Gulf of Maine, 3rd ed. Washington: Smithsonian Institution Press; 2002. p. 25-27

Carlson JK, McCandless CT, Cortes E, Grubbs RD, Andrews KI, MacNeil MA, Musick JA. 2009. An update on the status of the sand tiger shark, Carcharias Taurus in the northwest Atlantic Ocean. NOAA Tech Memo NMFS-SEFSC-585. 23 p. Accessed online (August 2015): http://www.sefsc.noaa.gov/labs/panama/library/TM_SEFSC_585.pdf

Gilmore RG, Dodrill JW, Linley PA. Reproduction and embryonic development of the sand tiger shark, *Odontaspis taurus* (Rafinesque). Fish Bull. 1983; 81: 201-225. Available: http://fishbull.noaa.gov/81-2/gilmore.pdf

Goldman KJ, Branstetter S, Musick JA. A re-examination of the age and growth of sand tiger sharks, Carcharias taurus , in the western North Atlantic: the importance of ageing protocols and use of multiple back-calculation techniques. Environ Biol Fishes. 2006; 77: 241-252. doi: 10.1007/978-1-4020-5570-6_4

McCandless CT, Kohler NE, Pratt Jr. HL, editors. Shark nursery grounds of the Gulf of Mexico and the east coast waters of the United States. Bethesda: AFS Symposium 50; 2007. 402p.

NMFS. 1999. Final Fisheries Management Plan for Atlantic Tunas, Swordfish, and Sharks. National Oceanic and Atmospheric Administration, National Marine Fisheries Service, Office of Sustainable Fisheries, Highly Migratory Species Management Division, Silver Spring, MD. Available: http://www.nmfs.noaa.gov/sfa/hms/documents/fmp/tss_fmp/index.html

NMFS. 2006. Final Consolidated Atlantic Highly Migratory Species Fishery Management Plan. National Oceanic and Atmospheric Administration, National Marine Fisheries Service, Office of Sustainable Fisheries, Highly Migratory Species Management Division, Silver Spring, MD. Available: http://www.nmfs.noaa.gov/sfa/hms/documents/fmp/consolidated/index.html

Smith SE, Au DW, Show C. Intrinsic rebound potentials of 26 species of Pacific sharks. Mar Freshwater Res. 1998; 49(7): 663-678. doi: 10.1071/MF97135

Return to Table of Contents

Scup – *Stenotomus chrysops*

Overall Vulnerability Rank = Moderate

Biological Sensitivity = Low
Climate Exposure = Very High
Data Quality = 88% of scores ≥ 2

Stenotomus chrysops	Expert Scores	Data Quality	Expert Scores Plots (Portion by Category)
Stock Status	1.0	3.0	
Other Stressors	1.8	2.4	
Population Growth Rate	2.5	2.6	
Spawning Cycle	3.2	3.0	
Complexity in Reproduction	1.3	3.0	
Early Life History Requirements	2.5	1.8	
Sensitivity to Ocean Acidification	1.3	3.0	
Prey Specialization	1.3	3.0	
Habitat Specialization	1.4	3.0	
Sensitivity to Temperature	1.6	3.0	
Adult Mobility	1.5	3.0	
Dispersal & Early Life History	2.3	2.0	
Sensitivity Score	Low		
Sea Surface Temperature	4.0	3.0	
Variability in Sea Surface Temperature	1.0	3.0	
Salinity	2.5	3.0	
Variability Salinity	1.2	3.0	
Air Temperature	4.0	3.0	
Variability Air Temperature	1.0	3.0	
Precipitation	1.2	3.0	
Variability in Precipitation	1.3	3.0	
Ocean Acidification	4.0	2.0	
Variability in Ocean Acidification	1.0	2.2	
Currents	2.0	1.0	
Sea Level Rise	1.5	1.5	
Exposure Score	Very High		
Overall Vulnerability Rank	Moderate		

Low
Moderate
High
Very High

Scup (*Stenotomus chrysops*)

Overall Climate Vulnerability Rank: **Moderate** (30% certainty from bootstrap analysis).

Climate Exposure: **Very High**. Three exposure factors contributed to this score: Ocean Surface Temperature (4.0), Ocean Acidification (4.0) and Air Temperature (4.0). Exposure to all three factors occur during all life stages. Scup spawn in coastal waters and early life stages are typically found in nearshore waters. Adults make seasonal onshore-offshore migrations.

Biological Sensitivity: **Low**. Three sensitivity attributes scored at or above 2.5: Population Growth Rate (2.5), Spawning Cycle (3.2), and Early Life History Requirements (2.5). Scup are a temperate sparid and have relatively long life spans. Spawning occurs in late spring and early summer in nearshore areas. Early life stages occur in coastal waters and are noticeable rare in plankton collections suggesting early settlement or epi-benthic larvae.

Distributional Vulnerability Rank: **High** (100% certainty from bootstrap analysis). Two attributes indicated vulnerability to distribution shift. As adults, Scup are mobile and make seasonal onshore-offshore migrations. Scup are also habitat generalists, but are commonly found around structured habitats.

Directional Effect in the Northeast U.S. Shelf: The effect of climate change on Scup on the Northeast U.S. Shelf is estimated to be positive, but this estimate is uncertain (<66% certainty in expert scores). Scup overwinter offshore and to the south and move northwards and inshore in the summer. As warming continues, the availability of winter and summer habitat may increases. The effect of ocean acidification over the next 30 years is likely to be minimal.

Data Quality: 88% of the data quality scores were 2 or greater.

Climate Effects on Abundance and Distribution: Long-term surveys indicate an effect of climate on Scup in the Northeast U.S. Shelf Ecosystem. Howell and Auster (2012) and Collie et al. (2008) classified Scup as part of the warm-temperate fauna in Long Island Sound and Narragansett Bay; both studies found adult Scup abundance increasing in recent years. Bell et al. (2014) found that Scup distribution shifted northward in the spring on the Northeast U.S. shelf and that the shift was related to temperature, not population abundance.

Life History Synopsis**:** Scup is a migratory, schooling, coastal fish species that occurs from Nova Scotia to South Carolina, but is most common between Cape Cod, Massachusetts, and Cape Hatteras, North Carolina (Able and Fahay, 2010). Scup mature at age 2-3 years (Able and Fahay, 2010). Spawning occurs annually from May to August with a peak in June in deep parts of large bays and coastal areas between New Jersey and Massachusetts (Klein-MacPhee, 2002; Able and Fahay, 2010). Eggs are pelagic, small, and hatch after 2-4 days (Klein-MacPhee, 2002; Able and Fahay, 2010). Larvae are pelagic in coastal waters and begin active feeding on small zooplankton 2-3 days after hatching (Steimle et al., 1999; Klein-MacPhee, 2002; Able and Fahay, 2010). Planktivores such as medusa, crustaceans, and fish consume larval Scup (Steimle et al., 1999). Scup settle to inshore bottom habitat during the late larval stage (15-30 mm) starting in early July (Steimle et al., 1999; Able and Fahay, 2010). Transition to the juvenile stage occurs at 40-60 mm total length in estuaries and coastal areas, where the juveniles reside in high salinity waters until egress in the early fall (Able and Fahay, 2010). Juveniles consume polychaetes, small benthic crustaceans, molluscs, small fishes, fish eggs, and larvae (Klein-MacPhee, 2002; Able and

Fahay, 2010). Growth is slow during the juvenile stage (Steimle et al., 1999). Juveniles and adults over-winter on the mid- and outer shelf between New Jersey and Cape Hatteras during which time, little is known about habitat preferences (Able and Fahay, 2010). During spring, juveniles and adults migrate north and inshore to coastal and estuarine areas where they use a variety of bottom types from open sandy areas to structured rocky or reef areas (Steimle et al., 1999). Scup school by size, and larger fish tend to stay outside of estuaries while younger fish move into estuaries (Klein-MacPhee, 2002; Able and Fahay, 2010). Adults are bottom feeders that consume cnidarians, squid, polychaetes, crustaceans, and small fishes (Klein-MacPhee, 2002; Able and Fahay, 2010). Several species of bony fish and elasmobranchs prey on Scup, but the dominant predators are Spiny Dogfish, Smooth Dogfish, and Bluefish (Klein-MacPhee, 2002; Able and Fahay, 2010). The Atlantic States Marine Fisheries Commission and the Mid-Atlantic Fishery Management Council jointly manage Scup under Amendments 8, 12, and 14 of the Summer Flounder, Scup, and Black Sea Bass Fishery management Plan (Terceiro, 2012). Scup are neither overfished nor experiencing overfishing (Terceiro, 2012).

Literature Cited:

Able KW, Fahay MP. Ecology of estuarine fishes: temperate waters of the western North Atlantic. Baltimore: The Johns Hopkins University Press; 2010. 566p.

Bell RJ, Richardson DE, Hare JA, Lynch PD, Fratantoni PS. Disentangling the effects of climate, abundance, and size on the distribution of marine fish: an example based on four stocks from the Northeast US shelf. ICES J Mar Sci. 2014; fsu217. doi: 10.1093/icesjms/fsu217

Collie JS, Wood AD, Jeffries HP. Long-term shifts in the species composition of a coastal fish community. Can J Fish Aquat Sci. 2008; 65(7): 1352-1365. doi: 10.1139/F08-048

Howell P, Auster PJ. Phase shift in an estuarine finfish community associated with warming temperatures. Mar Coast Fish. 2012; 4(1): 481-495. doi: 10.1080/19425120.2012.685144

Klein-MacPhee G. Scup/ Stenotomus chrysops (Linnaeus 1766). In: Collete BB, Klein-MacPhee G, editors, Fishes of the Gulf of Maine, 3rd edition. Washington: Smithsonian Institution Press; 2002. pp. 431-435.

Steimle FW, Zetlin CA, Berrien PL, Johnson DL, Chang S. 1999. Essential fish habitat source document: Scup, Stenotomus chrysops, life history and habitat characteristics. NOAA Tech Memo NMFS NE 149; 39 p. Accessed online (February 2015): http://www.nefsc.noaa.gov/nefsc/publications/tm/tm149/

Terceiro M. 2012. Stock Assessment of Scup (Stenotomus chrysops) for 2012. US Dept Commer, Northeast Fish Sci Cent Ref Doc. 12-25; 104 p. Available from: National Marine Fisheries Service, 166 Water Street, Woods Hole, MA 02543-1026. Accessed online (February 2015): http://nefsc.noaa.gov/publications/crd/crd1225/

Return to Table of Contents

Shortnose Sturgeon – *Acipenser brevirostrum*

Overall Vulnerability Rank = Very High

Biological Sensitivity = High
Climate Exposure = Very High
Data Quality = 83% of scores ≥ 2

Acipenser brevirostrum	Expert Scores	Data Quality	Expert Scores Plots (Portion by Category)
Stock Status	3.6	1.6	
Other Stressors	2.5	2.2	
Population Growth Rate	3.8	2.3	
Spawning Cycle	3.4	2.9	
Complexity in Reproduction	2.5	2.6	
Early Life History Requirements	3.0	2.3	
Sensitivity to Ocean Acidification	1.6	1.8	
Prey Specialization	1.3	3.0	
Habitat Specialization	2.5	3.0	
Sensitivity to Temperature	2.0	2.9	
Adult Mobility	2.7	3.0	
Dispersal & Early Life History	3.0	2.6	
Sensitivity Score	High		
Sea Surface Temperature	4.0	3.0	
Variability in Sea Surface Temperature	1.0	3.0	
Salinity	1.3	3.0	
Variability Salinity	1.2	3.0	
Air Temperature	4.0	3.0	
Variability Air Temperature	1.0	3.0	
Precipitation	1.3	3.0	
Variability in Precipitation	1.4	3.0	
Ocean Acidification	4.0	2.0	
Variability in Ocean Acidification	1.0	2.2	
Currents	2.0	1.0	
Sea Level Rise	2.6	1.5	
Exposure Score	Very High		
Overall Vulnerability Rank	Very High		

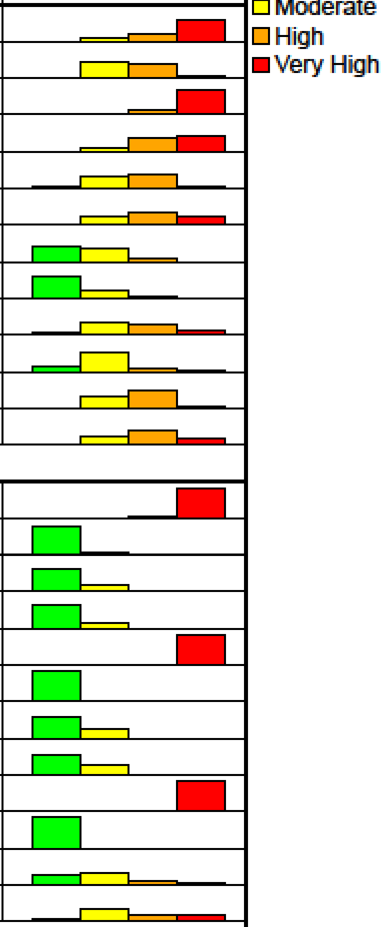

Shortnose Sturgeon (*Acipenser brevirostrum*)

Overall Climate Vulnerability Rank: **Very High** (98% certainty from bootstrap analysis).

Climate Exposure: **Very High**. Three exposure factors contributed to this score: Ocean Surface Temperature (4.0), Ocean Acidification (4.0) and Air Temperature (4.0). Shortnose Sturgeon are anadromous, spawning in freshwater, developing in freshwater and estuarine habitats, and feeding as adults in marine habitats.

Biological Sensitivity: **High**. Four sensitivity attributes scored above 3.0: Population Growth Rate (3.8), Stock Status (3.6), Spawning Cycle (3.4), Early Life History Requirements (3.0), and Dispersal and Early Life History. Shortnose Sturgeon was listed as Endangered under the Endangered Species Act in 1967 (SSSRT, 2010) and are long-lived and slow growing (Musick 2002). Spawning occurs in the spring and individuals spawn every 1-3 years. Eggs are benthic and relatively large and hatched larvae are relatively well-developed. Eggs and larvae inhabit fresh and brackish waters.

Distributional Vulnerability Rank: **Low** (98% certainty from bootstrap analysis). Shortnose Sturgeon are relatively invulnerable to distribution shifts. Spawning occurs in freshwater and adults are primarily resident to individual river systems (Able and Fahay 2010). However, recent genetic and acoustic tagging indicate that individuals do move between near-by river systems providing a basis for the species to shift distribution (Wirgin et al., 2010, Dionne et al., 2013).

Directional Effect in the Northeast U.S. Shelf: The effect of climate change on Shortnose Sturgeon is estimated to be neutral, but this estimate has a high degree of uncertainty (<66% certainty in expert scores). Climate factors have the potential to decrease (sea level rise; reduced dissolved oxygen) or increase (temperature) productivity of Shortnose Sturgeon. Understanding the magnitude and interaction of different effects is difficult at the point. The effect of ocean acidification over the next 30 years is likely to be minimal.

Data Quality: 83% of the data quality scores were 2 or greater indicate that data quality is moderate.

Climate Effects on Abundance and Distribution: The effect of climate on Shortnose Sturgeon populations is not well understood. A population viability analysis for Shortnose Sturgeon at the southern end of their range found that salt-water intrusion and decreases in summer dissolved oxygen could reduce population productivity (Jager et al., 2013). In the Hudson River, Woodland and Secor (2007) found that flow volume and water temperature in the fall months preceding spawning were significantly correlated with subsequent year-class strength. Numerous aspects of Shortnose Sturgeon life history and ecology are linked to temperature, river flow, dissolved oxygen, salinity, but the effect of change in these environmental variables on Shortnose Sturgeon is unclear (Cech and Doroshov, 2005; Ziegeweid et al., 2008a, 2008b). Habitat models coupled with global climate models for the cogener, European Atlantic Sturgeon (*Acipenser sturio*) indicate strong climate effects throughout the range, especially in the southern portions (Lassalle et al. 2010).

Life History Synopsis**:** Shortnose Sturgeon is an anadromous and land-locked fish species that occurs in rivers and estuaries from the St. John River, New Brunswick, to the St. Johns River, Florida (Able and Fahay, 2010). In northeastern United States waters, males of the species reach maturity in 3-5 years, females in 6-7 years; however the age at maturity is younger in the southeastern United States and older in Canadian waters (Musick, 2002). Spawning occurs from February in the southern areas to mid-

May in the northern areas in deep, swiftly moving, 9-12°C freshwater, over rocky substrate, in upstream areas of the main branch of large rivers (Musick, 2002; Able and Fahay, 2010). Benthic, adhesive eggs are deposited on the rocky bottom, then adults head back downstream as far as low-salinity estuarine water shortly after spawning (Musick, 2002; Able and Fahay, 2010). The large eggs hatch after 2 weeks and the demersal larvae remain hidden on the bottom for approximately 9 days before becoming active, photopositive swimmers and begin heading downstream (Musick, 2002). Larvae transform into juveniles at 57-67 mm (Able and Fahay, 2010). Larvae and juveniles occur in deep (>9 m) channels of the river with strong currents and sand or gravel substrates and remain in freshwater for at least 2 years (Able and Fahay, 2010). Juveniles are benthic foragers on crustaceans and insects and are consumed by perch (Able and Fahay, 2010). Adults spend most of their lives in rivers and low salinity areas of estuaries, but occasionally venture into near-coastal areas (Able and Fahay, 2010). Shortnose Sturgeons make seasonal migrations between spawning, feeding, and overwintering grounds. Summers are spent in areas with little or no current and cooler waters (Able and Fahay, 2010). Part of the population migrates upstream and overwinters near the spawning grounds, while the rest of the population remains in deep, higher-salinity areas until an upstream migration in spring just before spawning (Able and Fahay, 2010). Spent adults return to shallow, downstream, foraging habitat shortly after spawning (Musick, 2002). Adults prey on benthic crustaceans, insects, molluscs, small flounders, and polychaete worms (Musick, 2002; Able and Fahay, 2010). A long history of overfishing (for meat and caviar) and habitat destruction decreased the populations so much that Shortnose Sturgeon is listed as an endangered species in United States waters (NMFS, 1998) and a Species of Special Concern in Canada (COSEWIC, 2005).

Literature Cited:

Able KW, Fahay MP. 2010. Ecology of estuarine fishes: temperate waters of the western North Atlantic. Baltimore: The Johns Hopkins University Press; 2010. 566p.

Cech Jr JJ, Doroshov SI. Environmental requirements, preferences, and tolerance limits of North American sturgeons. In Sturgeons and paddlefish of North America. Netherlands: Springer; 2005. pp. 73-86.

Committee on the Status of Endangered Wildlife in Canada (COSEWIC). 2005. Assessment Summary – Shortnose Sturgeon. Accessed online (February 2015): http://www.registrelep-sararegistry.gc.ca/virtual_sara/files/cosewic/as_shortnose_sturgeon_e.pdf

Dionne PE, Zydlewski GB, Kinnison MT, Zydlewski J, Wippelhauser GS. Reconsidering residency: characterization and conservation implications of complex migratory patterns of shortnose sturgeon (Acispenser brevirostrum). Can J Fish Aquat Sci. 2013; 70(1): 119-127. doi: 10.1139/cjfas-2012-0196

Jager HI, Peterson DL, Farrae D, Bevelhimer MS. A population model to assess influences on the viability of the Shortnose sturgeon population in the Ogeechee River, Georgia. Trans Am Fish Soc. 2013; 142(3): 731-746. doi: 10.1080/00028487.2013.763853

Lassalle G, Crouzet P, Gessner J, Rochard E. Global warming impacts and conservation responses for the critically endangered European Atlantic sturgeon. Biolog Conserv. 2010; 143(11): 2441-2452. doi:10.1016/j.biocon.2010.06.008

Musick JA. Shortnose Sturgeon/ Acipenser brevirostrum LeSueur 1818. In: B.B. Collette BB, Klein-MacPhee G, editors, Fishes of the Gulf of Maine, 3rd ed. Washington: Smithsonian Institution Press; 2002. pp. 83-85.

National Marine Fisheries Service. 1998. Recovery Plan for the Shortnose Sturgeon (*Acipenser brevirostrum*). Prepared by the Shortnose Sturgeon Recovery Team for the National Marine Fisheries Service, Silver Spring, Maryland. 104 pages. Accessed online (February 2015): http://www.nmfs.noaa.gov/pr/pdfs/recovery/sturgeon_shortnose.pdf

Shortnose Sturgeon Status Review Team (SSSRT). 2010. A Biological Assessment of shortnose sturgeon (Acipenser brevirostrum). Report to National Marine Fisheries Service, Northeast Regional Office. November 1, 2010. 417 p. Available: http://www.nmfs.noaa.gov/pr/pdfs/species/shortnosesturgeon_biological_assessment2010.pdf

Wirgin I, Grunwald C, Stabile J, & Waldman JR. Delineation of discrete population segments of shortnose sturgeon Acipenser brevirostrum based on mitochondrial DNA control region sequence analysis. Conserv Genet. 2010; 11(3): 689-708. doi: 10.1007/s10592-009-9840-1

Woodland RJ, Secor DH. Year-class strength and recovery of endangered shortnose sturgeon in the Hudson River, New York. Trans Am Fish Soc. 2007; 136(1): 72-81. doi: DOI:10.1577/T06-015.1

Ziegeweid JR, Jennings CA, Peterson DL. Thermal maxima for juvenile shortnose sturgeon acclimated to different temperatures. Environ Biol Fishes. 2008; 82(3): 299-307. doi: 10.1007/s10641-007-9292-8

Ziegeweid JR, Jennings CA, Peterson DL, Black MC. Effects of salinity, temperature, and weight on the survival of young-of-year shortnose sturgeon. Trans Am Fish Soc. 2008; 137(5): 1490-1499. doi: 10.1577/T07-046.1

Return to Table of Contents

Silver Hake – *Merluccius bilinearis*

Overall Vulnerability Rank = Low

Biological Sensitivity = Low
Climate Exposure = High
Data Quality = 88% of scores ≥ 2

Merluccius bilinearis	Expert Scores	Data Quality	Expert Scores Plots (Portion by Category)
Stock Status	2.2	2.7	
Other Stressors	1.4	1.0	
Population Growth Rate	1.7	2.2	
Spawning Cycle	1.4	3.0	
Complexity in Reproduction	1.8	2.8	
Early Life History Requirements	2.1	2.3	
Sensitivity to Ocean Acidification	1.2	2.0	
Prey Specialization	1.5	3.0	
Habitat Specialization	1.2	2.8	
Sensitivity to Temperature	1.6	3.0	
Adult Mobility	1.3	2.8	
Dispersal & Early Life History	1.8	2.6	
Sensitivity Score	Low		
Sea Surface Temperature	3.9	3.0	
Variability in Sea Surface Temperature	1.0	3.0	
Salinity	1.9	3.0	
Variability Salinity	1.2	3.0	
Air Temperature	1.0	3.0	
Variability Air Temperature	1.0	3.0	
Precipitation	1.0	3.0	
Variability in Precipitation	1.0	3.0	
Ocean Acidification	4.0	2.0	
Variability in Ocean Acidification	1.0	2.2	
Currents	2.1	1.0	
Sea Level Rise	1.1	1.5	
Exposure Score	High		
Overall Vulnerability Rank	Low		

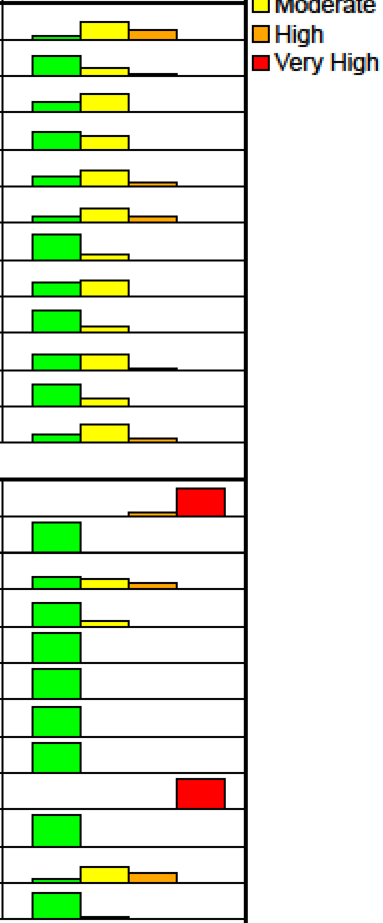

Return to Table of Contents

Silver Hake (*Merluccius bilinearis*)

Overall Climate Vulnerability Rank: **Low** (100% certainty from bootstrap analysis).

Climate Exposure: **High**. Two exposure factors contributed to this score: Ocean Surface Temperature (3.9) and Ocean Acidification (4.0). All life stages of Silver Hake use marine habitats.

Biological Sensitivity: **Low**. No sensitivity attributes scored above 2.5.

Distributional Vulnerability Rank: **High** (100% certainty from bootstrap analysis). Silver Hake are habitat generalists that are moderately mobile and have dispersive early life stages (Lock and Packer, 2004).

Directional Effect in the Northeast U.S. Shelf: The effect of climate change on Silver Hake on the Northeast U.S. Shelf is very likely to be negative (>95% certainty in expert scores). Decreases in recruitment related to warming have been observed on the Scotian Shelf and the distribution has shifted northward with warming on the Northeast U.S. Shelf. Continued warming will likely cause continued decreases in recruitment and northward shifts in distribution.

Data Quality: 88% of the data quality scores were 2 or greater indicate that data quality is moderate.

Climate Effects on Abundance and Distribution: On the Scotian Shelf, Silver Hake recruitment has been linked to temperature: lower recruitment in years of higher temperature (Sigaev, 1992). Bartolino et al. (2008) found a similar negative relationship between temperature and recruitment in a congener in the Mediterranean Sea. Silver Hake distribution also changes with temperature (Murawski, 1993) and has shifted northwards in recent years (Nye et al., 2009). In subsequent work, Nye et al. (2011) reported that Silver Hake distribution was correlated to the position of the Gulf Stream and hypothesized large-scale forcing on shelf dynamics as the causal link. Distribution of Pacific Hake, a congener, is also affected by local oceanographic conditions (Agostini et al., 2006).

Life History Synopsis**:** Silver Hake is a fast swimming, mostly benthic, marine finfish species that occurs from the Gulf of St. Lawrence to South Carolina, but is most abundant from Nova Scotia to New Jersey (Lock and Packer, 2004). The species reaches maturity between 2 and 3 years of age (NEFSC, 2011). Spawning occurs in inshore areas of the Gulf of Maine, southern Georges Bank, Nantucket Shoals, and south of Martha's Vineyard to Cape Hatteras (Klein-MacPhee, 2002). Spawning begins in January in the southern portion of the range with a peak in spring, and continues to the north with a northern US peak in summer and a Canadian peak in late summer (Lock and Packer, 2004). Silver Hake are serial spawners with up to three spawning events per season (Klein-MacPhee, 2002). Eggs are pelagic and hatch after about 2 days (Klein-MacPhee, 2002). Larvae are pelagic in the upper 40 m of water for approximately 1 month in the southern part of their range to up to 5 months in Canadian waters (Klein-MacPhee, 2002; Lock and Packer, 2004). Calanoid copepods are the main prey of larval Silver Hake (Klein-MacPhee, 2002). Larvae first mature into pelagic juveniles that associate with jellyfish, then settle to the benthos at 12-20mm fork length (Klein-MacPhee, 2002; Lock and Packer, 2004). Benthic juveniles prefer silt or sand bottom with amphipod tubes for cover (Klein-MacPhee, 2002). Copepods, amphipods, mysids, euphausiids, and small decapod shrimp are the main prey of juveniles (Klein-MacPhee, 2002). Adult Silver Hake prefer cool waters (3-17°C) at a variety of depths over sand or silt bottom from shallow inshore areas out to 400 m and possibly deeper (Klein-MacPhee, 2002). Silver Hake are more active and hunt at night for crustaceans, a large variety of small fish, and squid (Klein-MacPhee, 2002). An ontogenetic shift from mostly crustaceans to mostly fish and squid prey occurs at 20-25cm, and

cannibalism is also quite common in the species (Klein-MacPhee, 2002). Some of the many predators of Silver Hake include: Spiny Dogfish, Little Skate, Monkfish (Goosefish), Pollock, Atlantic Cod, Haddock, hakes, Acadian Redfish, Sea Raven, Bluefish, Atlantic Mackerel, Swordfish, flounders, Silver Hake, and harbor porpoise (Klein-MacPhee, 2002). Seasonal migrations from inshore summer and autumn habitat to offshore winter and spring habitat are influenced by temperature (Klein-MacPhee, 2002). Silver Hake also undergo along-shore migrations and the northern and southern stocks mix on Georges Bank in summer (Lock and Packer, 2004). Silver Hake is managed by the New England Fishery Management Council's small mesh multispecies plan as two stocks: northern Georges Bank and the Gulf of Maine to the north and southern Georges Bank to Cape Hatteras to the south (NEFSC, 2011). Based on a variety of metrics, the Gulf of Maine and Mid-Atlantic stocks are distinct, but the degree of mixing and the location of the boundary between stocks are not well understood (Lock and Packer, 2004). Based on the most recent assessment, neither stock is overfished, nor is overfishing occurring (NEFSC, 2011).

Literature Cited:

Agostini VN, Francis RC, Hollowed AB, Pierce SD, Wilson C, Hendrix AN. The relationship between Pacific hake (*Merluccius productus*) distribution and poleward subsurface flow in the California Current System. Can J Fish Aquat Sci. 2006; 63(12): 2648-2659. DOI: 10.1139/f06-139

Bartolino V, Colloca F, Sartor P, Ardizzone G. Modelling recruitment dynamics of hake, *Merluccius merluccius*, in the central Mediterranean in relation to key environmental variables. Fisher Res. 2008; 92(2): 277-288. DOI: 10.1016/j.fishres.2008.01.007

Klein-MacPhee G. Silver Hake/ *Merluccius bilinearis* (Mitchill 1814). Pages 219-222. In: BB Collette, G Klein-MacPhee (editors), Fishes of the Gulf of Maine, 3rd edition. Smithsonian Institution Press, Washington D.C. 2002; 882 p.

Lock MC, Packer PB. Essential fish habitat source document: Silver hake, *Merluccius bilinearis*, life history and habitat characteristiscs, 2nd edition. NOAA Tech Memo NMFS NE 2004; 186: 68 p. Accessed online (February 2015): http://www.nefsc.noaa.gov/nefsc/publications/tm/tm186/

Murawski SA. Climate change and marine fish distributions: forecasting from historical analogy. Trans Amer Fish Soc. 1993; 122(5): 647-658. DOI: 0.1577/1548-8659(1993)122<0647:CCAMFD>2.3.CO;2

Northeast Fisheries Science Center. 51st Northeast Regional Stock Assessment Workshop (51st SAW) Assessment Report. US Dept Commer, Northeast Fish Sci Cent Ref Doc. 2011; 11-02: 856 p. Accessed Online (August 2015): http://www.nefsc.noaa.gov/publications/crd/crd1102/

Nye JA, Joyce TM, Kwon YO, Link JS. Silver hake tracks changes in Northwest Atlantic circulation. Nature Comm. 2011: 2; 412. DOI: 10.1038/ncomms1420

Nye JA, Link JS, Hare JA, Overholtz WJ. Changing spatial distribution of fish stocks in relation to climate and population size on the Northeast United States continental shelf. Mar Ecol Prog Ser. 2009; 393: 111-129. DOI: 0.3354/meps08220

Sigaev IK. On possible causes of year-class variability of Scotian Shelf Silver Hake (*Merluccius bilinearis*). J Northw Atlant Fish Sci. 1992; 14: 103-105. Accessed Online (August 2015): http://journal.nafo.int/J14/sigaev.pdf

Return to Table of Contents

Smooth Dogfish – *Mustelus canis*

Overall Vulnerability Rank = Low

Biological Sensitivity = Low
Climate Exposure = High
Data Quality = 88% of scores ≥ 2

Mustelus canis	Expert Scores	Data Quality	Expert Scores Plots (Portion by Category)
Stock Status	2.3	0.8	
Other Stressors	1.8	2.3	
Population Growth Rate	2.7	2.8	
Spawning Cycle	2.1	2.8	
Complexity in Reproduction	1.5	2.0	
Early Life History Requirements	1.0	3.0	
Sensitivity to Ocean Acidification	1.3	2.6	
Prey Specialization	1.2	3.0	
Habitat Specialization	1.4	3.0	
Sensitivity to Temperature	1.9	3.0	
Adult Mobility	1.1	2.8	
Dispersal & Early Life History	1.0	3.0	
Sensitivity Score	Low		
Sea Surface Temperature	3.9	3.0	
Variability in Sea Surface Temperature	1.0	3.0	
Salinity	2.4	3.0	
Variability Salinity	1.2	3.0	
Air Temperature	1.0	3.0	
Variability Air Temperature	1.0	3.0	
Precipitation	1.0	3.0	
Variability in Precipitation	1.0	3.0	
Ocean Acidification	4.0	2.0	
Variability in Ocean Acidification	1.0	2.2	
Currents	2.1	1.0	
Sea Level Rise	1.1	1.5	
Exposure Score	High		
Overall Vulnerability Rank	Low		

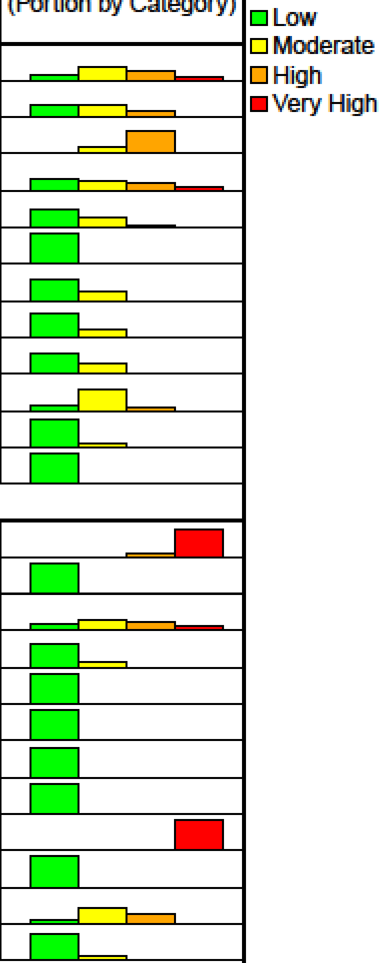

Smooth Dogfish (*Mustelus canis*)

Overall Climate Vulnerability Rank: **Low** (84% certainty from bootstrap analysis).

Climate Exposure: **High**. Two exposure factors contributed to this score: Ocean Surface Temperature (3.9) and Ocean Acidification (4.0). Smooth Dogfish are demersal / semi-pelagic and complete their life cycle in marine habitats.

Biological Sensitivity: **Low**. One attribute scored above 2.5: Population Growth Rate (2.5). Dogfish (*Mustelus sp.)* have low population growth rates compared to many teleost species, but have relatively high productivity when compared to other shark species (Smith et al., 2008).

Distributional Vulnerability Rank: **Very High** (100% certainty from bootstrap analysis). Smooth Dogfish are habitat generalists and highly mobile as adults, making seasonal migrations. In addition, Smooth Dogfish are viviparous (Conrath and Musick, 2002).

Directional Effect in the Northeast U.S. Shelf: The effect of climate change on Smooth Dogfish is likely to be neutral (90-95% certainty in expert scores). Smooth Dogfish inhabits temperate waters and may benefit from warming on the Northeast U.S. Shelf. But ocean acidification may reduce productivity and no changes in distribution have been observed over the past 30 years despite significant warming.

Data Quality: 88% of the data quality scores were 2 or greater indicate that data quality is moderate.

Climate Effects on Abundance and Distribution: Little specific information exists on the effect of climate on Smooth Dogfish. Dixson et al. (2015) suggested that Smooth Dogfish feeding could be affected by ocean acidification by the end of this century. In regional studies of distribution, Smooth Dogfish was not included (Nye et al., 2009) but examination of NEFSC trawl survey suggests no change in the center of the distribution over the last 30 years (http://oceanadapt.rutgers.edu/, website last checked 13 June 2015).

Life History Synopsis**:** Smooth Dogfish is a viviparous, demersal, marine elasmobranch species that occurs from the Bay of Fundy, Canada, to Argentina, but is rare north of Cape Cod and absent along the central American coast (Able and Fahay, 2010). Females reach 50% maturity at 102.3 cm fork length (4.41 years of age)and males at 85.4 cm fork length (2.46 years of age) (Conrath and Musick, 2002; Conrath et al., 2002). Mating occurs from May to September followed by a 10-12 month gestation (Branstetter, 2002; Conrath and Musick, 2002; Able and Fahay, 2010). Pupping occurs in estuarine waters from Virginia to Massachusetts (McCandless et al. 2007). Litters of 3-18 pups are born in spring and summer (older, larger females produce larger litters), and juveniles remain in the estuarine nursery areas until fall (Able and Fahay, 2010). Larger juveniles occur in deeper waters of bays before joining adults on the continental shelf (Able and Fahay, 2010). During their first year, Smooth Dogfish prey on polychaetes, decapod shrimp, crabs, bivalves, and small estuarine fish (Able and Fahay, 2010). Adult Smooth Dogfish occur from estuaries to the continental shelf edge with seasonal migrations from the inshore pupping and nursery grounds in spring to early fall to the winter grounds on the continental shelf edge off North Carolina to New Jersey (Branstetter, 2002; Able and Fahay, 2010). Decapod crustaceans (crabs, lobsters, shrimp) are the dominant prey of adult Smooth Dogfish, but squid, bivalves, gastropods, and several species of teleosts are also frequently consumed (Gelsleichter et al., 1999; Able and Fahay, 2010). Smooth Dogfish are preyed on by larger sharks and teleosts (Smith 1995, Gelsleichter et al., 1999, Able and Fahay, 2010). The Smooth Dogfish is managed in state waters under the by

Atlantic States Marine Fishery Commission's Interstate Fishery Management Plan for Atlantic Coastal Sharks (ASMFC, 2013). Federal management for Smooth Dogfish falls under the Consolidated Atlantic Highly Migratory Species Fishery Management Plan per Amendment 3 (NMFS 2010). The Atlantic smooth dogfish stock was recently assessed and is not considered overfished nor is overfishing occurring (SEDAR 2015).

Literature Cited:

Able KW, Fahay MP. 2010. Ecology of estuarine fishes: temperate waters of the western North Atlantic. Baltimore: The Johns Hopkins University Press; 2010. 566p.

Atlantic States Marine Fisheries Commission (ASMFC). 2013. Addendum II to the interstate fishery management plan for Atlantic coastal sharks: Smoothhound shark state shares. Accessed Online (August 2015): http://www.asmfc.org/uploads/file/smoothDogfishAddendumII_May2013.pdf

Branstetter S. Smooth Dogfish/ Mustelus canis canis (Mitchill 1815). In: B.B. Collette BB, Klein-MacPhee G, editors, Fishes of the Gulf of Maine, 3rd ed. Washington: Smithsonian Institution Press; 2002. P 37-38.

Conrath CL, Gelsleichter J, Musick JA. 2002. Age and growth of the smooth dogfish (*Mustelus canis*), in the northwest Atlantic Ocean. Fish Bull. 2002; 100: 674-682. Available: http://aquaticcommons.org/15241/1/03conrat.pdf

Conrath CL, Musick JA. Reproductive biology of the smooth dogfish, Mustelus canis, in the northwest Atlantic Ocean. Environ Biol Fishes. 2002; 64(4): 367-377. doi: 10.1023/A:1016117415855

Dixson DL, Jennings AR, Atema J, Munday PL. Odor tracking in sharks is reduced under future ocean acidification conditions. Glob Chang Biol. 2015; 21(4): 1454-1462. doi: 10.1111/gcb.12678

Gelsleichter J, Musick JA, Nichols S. Food habits of the smooth dogfish, *Mustelus canis*, dusky shark, *Carcharhinus obscurus*, Atlantic sharpnose shark, *Rhizoprionodon terraenovae*, and the sand tiger, *Carcharias taurus*, from the northwest Atlantic Ocean. Environ Biol Fishes, 1999; 54(2): 205-217. doi: 10.1023/A:1007527111292

McCandless CT, Kohler NE, Pratt Jr. HL, editors. Shark nursery grounds of the Gulf of Mexico and the East Coast waters of the United States. Bethesda: American Fisheries Society, Symposium 50; 2007. 402 p.

NMFS. 2014. Final Amendment 3 to the Consolidated Atlantic Highly Migratory Species Fishery Management Plan. Available: http://www.fisheries.noaa.gov/sfa/hms/documents/fmp/am3/index.html

Nye JA, Link JS, Hare JA, Overholtz WJ. Changing spatial distribution of fish stocks in relation to climate and population size on the Northeast United States continental shelf. Mar Ecol Prog Ser. 2009; 393: 111-129. doi: 10.3354/meps08220

SEDAR. 2015. Southeast Data, Assessment, and Review (SEDAR) 39 Stock Assessment Report. Highly Migratory Species Atlantic Smooth Dogfish Shark. Available: http://sedarweb.org/docs/sar/S39_Atl_smooth_dog_SAR.pdf

Smith JW. Life history of cobia, *Rachycentron canadum* (Osteichtyes: Rachycentridae), in North Carolina waters. Brimleyana. 1995; 23: 1-23. Available: http://sedarweb.org/docs/wsupp/SEDAR28-RD08%20Smith%201995.pdf

Smith SE, Au DW, Show C. Intrinsic rates of increase in pelagic elasmobranchs. In: Camhi MD, Pikitch EK, Babcock EA, editors, Sharks of the Open Ocean: Biology, Fisheries and Conservation. Hoboken: Wiley-Blackwell; 2008. p. 288-297.

Return to Table of Contents

Smooth Skate – *Malacoraja senta*

Overall Vulnerability Rank = Moderate

Biological Sensitivity = Moderate
Climate Exposure = High
Data Quality = 83% of scores ≥ 2

Malacoraja senta	Expert Scores	Data Quality	Expert Scores Plots (Portion by Category)
Stock Status	2.6	2.5	
Other Stressors	1.2	1.4	
Population Growth Rate	3.0	2.0	
Spawning Cycle	1.2	3.0	
Complexity in Reproduction	1.3	1.8	
Early Life History Requirements	1.1	3.0	
Sensitivity to Ocean Acidification	1.6	2.1	
Prey Specialization	1.2	3.0	
Habitat Specialization	1.2	3.0	
Sensitivity to Temperature	2.1	2.8	
Adult Mobility	2.0	2.1	
Dispersal & Early Life History	2.5	3.0	
Sensitivity Score	Moderate		
Sea Surface Temperature	3.9	3.0	
Variability in Sea Surface Temperature	1.0	3.0	
Salinity	1.4	3.0	
Variability Salinity	1.2	3.0	
Air Temperature	1.0	3.0	
Variability Air Temperature	1.0	3.0	
Precipitation	1.0	3.0	
Variability in Precipitation	1.0	3.0	
Ocean Acidification	4.0	2.0	
Variability in Ocean Acidification	1.0	2.2	
Currents	2.1	1.0	
Sea Level Rise	1.1	1.5	
Exposure Score	High		
Overall Vulnerability Rank	Moderate		

Low
Moderate
High
Very High

Smooth Skate (*Malacoraja senta*)

Overall Climate Vulnerability Rank: **Moderate** (93% certainty from bootstrap analysis).

Climate Exposure: **High**. Two exposure factors contributed to this score: Ocean Surface Temperature (3.9) and Ocean Acidification (4.0). Smooth Skate are demersal and complete their life cycle in marine habitats.

Biological Sensitivity: **Moderate**. Two sensitivity attributes scored above 2.5: Stock Status (2.6) and Population Growth Rate (3.0). In 2013, based on trawl survey indices Smooth Skate was above the biomass threshold but below the biomass target. Further, the index has been low in the 1980s, 1990s and 2000s relative to higher indices in the 1960s and 1970s. In general, skates have a low population growth rate (higher sensitivity to climate change) (Frisk, 2010).

Distributional Vulnerability Rank: **Moderate** (44% certainty from bootstrap analysis). Smooth Skate are habitat generalists and moderately mobile as adults, making seasonal migrations.

Directional Effect in the Northeast U.S. Shelf: The effect of climate change on Smooth Skate is very likely to be negative (>95% certainty in expert scores). Smooth Skate is a cold-water species and warming and acidification may reduce productivity. In addition, abundance indices are low relative to the 1960s and 1970s.

Data Quality: 83% of the data quality scores were 2 or greater indicate that data quality is moderate.

Climate Effects on Abundance and Distribution: Little specific information exists on the effect of climate on Smooth Skate. Di Santo (2015) found that increased warming and acidification reduce body condition of newly hatched Little Skate. If similar effects occur on Smooth Skate, reductions in size could result in reduced juvenile survival and thus recruitment. In regional studies of distribution, Smooth Skate was not included (Murawski, 1993; Nye et al., 2009) but examination of NEFSC trawl survey data suggests a northward then southward shift in distribution over the last 30 years (http://oceanadapt.rutgers.edu/, website last checked 13 June 2015).

Life History Synopsis**:** Smooth Skate is a boreal, marine elasmobranch species found from the Gulf of St. Lawrence and Labrador Shelf to South Carolina, with the center of abundance in the Gulf of Maine (Packer et al., 2003). Smooth Skate reach maturity at 5 years, mate using internal fertilization, and females likely spawn in summer and winter, based on the occurrence of females with fully formed egg capsules (Packer et al., 2003), or year round based on hormone levels (Kneebone et al., 2007). Horned egg cases each contain a single egg and house the embryo throughout early development (Packer et al., 2003). Juveniles hatch out of the egg case as small immature adults (Packer et al., 2003). Juveniles and adults range from 31-874 m depths, but are most common at depths 110-500 m, in cold water, primarily over mud or silt, but also use broken shell, sand, or gravel bottom (McEachran, 2002). Smooth Skate specialize on epifaunal crustaceans, but their diet includes: cephalopods, polychaetes, copepods, larval stomatopods, isopods, amphipods, mysids, euphausiids, decapod shrimps and crabs, and fishes (McEachran, 2002). Decapods and euphausiids are the primary prey throughout the life of the Smooth Skate, but an ontogenetic shift from amphipods, mysids, and euphausiids to crustaceans occurs at about 30cm (McEachran, 2002). Not much is known about predators of the species, but other skates likely prey on Smooth Skates, especially as embryos (Packer et al., 2003). The New England Fishery Management Council manages Smooth Skate as part of a seven species skate complex under the Skate

Fishery Management Plan. Based on the most recent assessment, Smooth Skate are not overfished nor is overfishing occurring (NEFSC, 2007).

Literature Cited:

Di Santo V. Ocean acidification exacerbates the impacts of global warming on embryonic little skate, *Leucoraja erinacea* (Mitchill). J Exp Mar Bio Ecol. 2015; 463: 72-78. doi:10.1016/j.jembe.2014.11.006

Frisk MG. Life history strategies of batoids. In: Carrier, JC, Musick, JA, Heithaus, MR, editors, Sharks and Their Relatives II: Biodiversity, Adaptive Physiology, and Conservation. Boca Raton: CRC Press; 2010. 283-318.

Kneebone J, Ferguson DE, Slikowski JA,Tsang PCW. Endocrinological investigation into the reproductive cycles of two sympatric skate species, Malacoraja senta and Amblyraja radiata, in the western Gulf of Maine. Environ Biol Fishes. 2007; 80: 257-265. doi: 10.1007/978-1-4020-9703-4_10

McEachran JD. Smooth Skate/ Malacoraja senta (Garman 1885). In: B.B. Collette BB, Klein-MacPhee G, editors, Fishes of the Gulf of Maine, 3rd ed. Washington: Smithsonian Institution Press; 2002. p. 72-74.

Murawski SA. Climate change and marine fish distributions: forecasting from historical analogy. Trans Am Fish Soc. 1993; 122(5): 647-658. doi: 10.1577/1548-8659(1993)122<0647:CCAMFD>2.3.CO;2

Northeast Fisheries Science Center (NEFSC). 2007. 44th Northeast Regional Stock Assessment Workshop (44th SAW): 44th SAW assessment report. US Dep Commer, Northeast Fish Sci Cent Ref Doc 07-10; 661 p. Accessed online (August 2015): http://www.nefsc.noaa.gov/publications/crd/crd0710/

Nye JA, Link JS, Hare JA, Overholtz WJ. Changing spatial distribution of fish stocks in relation to climate and population size on the Northeast United States continental shelf. Mar Ecol Prog Ser. 2009; 393: 111-129. doi: 10.3354/meps08220

Packer DB, Zetlin CA, Vitaliano JJ. 2003. Essential fish habitat source document: Smooth skate, Malacoraja senta, life history and habitat characteristics. NOAA Tech Memo NMFS NE 177; 26 p. Accessed Online (August 2015): http://www.nefsc.noaa.gov/nefsc/publications/tm/tm177/

Return to Table of Contents

Softshell Clam – *Mya arenaria*

Overall Vulnerability Rank = Very High

Biological Sensitivity = High
Climate Exposure = Very High
Data Quality = 83% of scores ≥ 2

Mya arenaria	Expert Scores	Data Quality	Expert Scores Plots (Portion by Category)
Stock Status	2.3	0.4	
Other Stressors	2.6	2.6	
Population Growth Rate	1.9	2.8	
Spawning Cycle	2.4	3.0	
Complexity in Reproduction	2.3	2.6	
Early Life History Requirements	2.4	2.6	
Sensitivity to Ocean Acidification	3.5	1.8	
Prey Specialization	1.7	2.8	
Habitat Specialization	1.8	2.8	
Sensitivity to Temperature	1.8	3.0	
Adult Mobility	4.0	2.8	
Dispersal & Early Life History	1.9	2.8	
Sensitivity Score	High		
Sea Surface Temperature	4.0	3.0	
Variability in Sea Surface Temperature	1.0	3.0	
Salinity	1.4	3.0	
Variability Salinity	1.2	3.0	
Air Temperature	3.5	3.0	
Variability Air Temperature	1.0	3.0	
Precipitation	1.2	3.0	
Variability in Precipitation	1.3	3.0	
Ocean Acidification	4.0	2.0	
Variability in Ocean Acidification	1.0	2.2	
Currents	2.0	1.0	
Sea Level Rise	1.6	1.5	
Exposure Score	Very High		
Overall Vulnerability Rank	Very High		

Low
Moderate
High
Very High

Softshell Clam (*Mya arenaria*)

Overall Climate Vulnerability Rank: **Very High** (58% certainty from bootstrap analysis).

Climate Exposure: **Very High**. Three exposure factors contributed to this score: Ocean Surface Temperature (4.0), Air Temperature (3.5), and Ocean Acidification (4.0). Softshell Clam utilize near coastal and intertidal habitats.

Biological Sensitivity: **High**. Two sensitivity attributes scored above 3.0: Sensitivity to Ocean Acidification (3.5) and Adult Mobility (4.0). Softshell Clams are sessile and have a calcium carbonate shell.

Distributional Vulnerability Rank: **High** (82% certainty from bootstrap analysis).

Directional Effect in the Northeast U.S. Shelf: The effect of climate change on Softshell Clam is likely to be negative (90-95% certainty in expert scores). Ocean acidification is likely to negatively impact Softshell Clams, but carbonate chemistry is complicated in coastal and intertidal systems. Warming temperatures may also lead to decreases in available habitat and may decrease productivity.

Data Quality: 83% of the data quality scores were 2 or greater indicate that data quality is moderate.

Climate Effects on Abundance and Distribution: Long-term decreases in recruitment of Softshell Clam in the Wadden Sea has been linked to an increase in predation pressure, with predators increases linked to climate change (Beukema and Dekker, 2005, Freitas et al., 2007). Laboratory studies found that calcification rates of Softshell Clam decreased as aragonite saturation state decreased from 2.5 to 1(ocean acidification) (Reis 2009). However, carbonate chemistry and ocean acidification are affected by many factors including atmospheric CO_2, making future aragonite saturation state in these systems difficult to project (Waldbusser and Salisbury, 2014). However, these studies suggest that productivity of Softshell Clam in the Northeast U.S. Shelf Ecosystem is likely to decrease as a result of direct (ocean acidification) and indirect (increases in predation) effects of climate change.

Life History Synopsis**:** Softshell Clam, or softshell clam, is a coastal bivalve species found from Labrador to South Carolina, extending in low abundance south to Florida, throughout western Europe, and introduced to the eastern Pacific from Alaska to California (Newell and Hidu, 1986). Softshell Clams are typically dioecious (sepearte sexes) but can be hermaphroditic. They reach maturity within 5 years, at sizes typically >20 mm (Abraham and Dillon, 1986; Newell and Hidu, 1986). Spawning season is determined by temperature and food availability, occurs from late-spring through fall, and may occur as two spawning events (spring and fall) south of Cape Cod, Massachusetts, or a single event north of Cape Cod (Newell and Hidu, 1986). Males spawn first, inducing egg release in females, and fertilization is external (Newell and Hidu, 1986). Individual females are capable of producing millions of eggs each year, with larger females producing more eggs than smaller individuals (Newell and Hidu, 1986). The pelagic eggs hatch after 9-12 hours (Abraham and Dillon, 1986; Newell and Hidu, 1986). The ciliated embryo quickly develops into the planktonic trochophore larva, which is also ciliated and feeds on suspended particles (Abraham and Dillon, 1986). After 1-1.5 days, the first shell and swimming organ develop and the veliger stage begins (Abraham and Dillon, 1986; Newell and Hidu, 1986). The veliger larva uses the ciliated velum to remain suspended in the water column and drifts in estuarine and ocean currents (Abraham and Dillon, 1986; Newell and Hidu, 1986). Larval Softshell Clams feed on phytoplankton and are preyed on by fish larvae, jellyfish, and ctenophores (Abraham and Dillon, 1986; Newell and Hidu, 1986). This stage is often the most abundant plankton during late summer in inshore,

subsurface waters, but is also characterized by very high mortality rates (Newell and Hidu, 1986). After 2-6 weeks (duration depends on water temperature) and at approximately 200 μm, the shell thickens, a foot replaces the velum, the byssal gland develops, and the larva settles to the substrate, and becomes a bottom-dwelling spat (Abraham and Dillon, 1986; Newell and Hidu, 1986). The late-stage larva can delay metamorphosis until a suitable attachment site is found, but once found the spat anchors to the substrate with the byssal threads and can remain attached until 7 mm long (Abraham and Dillon, 1986; Newell and Hidu, 1986). The juvenile spat is generally attached to the substrate, but can crawl to a more favorable location with the foot (Abraham and Dillon, 1986). The floating and crawling stage can last 2-5 weeks, but the spat eventually burrow into the sediment, becoming sedentary (Abraham and Dillon, 1986; Newell and Hidu, 1986). The juvenile is shallowly buried in sandy substrate, so predation rates are high and movement due to hydrographic condition such as storms or eddies causes irregular settlement patterns (Abraham and Dillon, 1986; Newell and Hidu, 1986). Predators of juvenile Softshell Clam include: oyster drills, flatworms, blue, green, and mud crabs, Mummichog, Spot, Cownose Ray, American Eel, Winter Flounder, polychaetes, snails, and shrimp (Abraham and Dillon, 1986; Homer et al., 2011). Like juveniles, adults inhabit sand bottom, or a mix of sand and mud or clay, of intertidal bays and inlets out to 199m (Abraham and Dillon, 1986; Newell and Hidu, 1986). Burrowing depth increases with time, so adults can be as deep as 30 cm in the sediment with the siphons extended to the sediment surface (Abraham and Dillon, 1986). Softshell Clams can tolerate a wide range of temperature and salinities, but adults experience and tolerate a narrower range than juveniles due to their deep burrows (Abraham and Dillon, 1986; Newell and Hidu, 1986). Additionally, southern populations can tolerate lower salinities than northern populations (Newell and Hidu, 1986). Softshell Clams are filter feeders of microscopic particles of organic materials, especially planktonic flagellates and diatoms, but also organic detritus and bacteria (Newell and Hidu, 1986). Crabs, Spot, Atlantic Croaker, Cownose Ray, American Eel, Winter and Summer Flounder, moon snails, gulls, diving ducks, tundra swans, and raccoons are predators of the species, but the depth of burrows helps Softshell Clams avoid most predators (Abraham and Dillon, 1986; Newell and Hidu, 1986; Homer et al., 2011). Softshell Clams support important commercial and recreational fisheries throughout most of their range and are managed by each state. The Chesapeake Bay fishery collapsed and is slow to recover due to a combination of overharvesting, disease, and predation (Homer et al., 2011). Red tides do not harm the clams, but bioaccumulation of toxins can cause paralytic shellfish poisoning in humans (Abraham and Dillon, 1986).

Literature Cited:

Abraham BJ, Dillon PL. Species Profiles: life histories and environmental requirements of coastal fishes and invertebrates (mid-Atlantic) – softshell clam. U.S. Fish Wildl. Serv. Biol. Rep. 82(11.68). U. S. Army Corps of Engineers, 1986. TR EL-82-4. 18 pp. Accessed Online (August 2015): http://www.nwrc.usgs.gov/publications/specprof.htm

Beukema JJ, Dekker R. Decline of recruitment success in cockles and other bivalves in the Wadden Sea: possible role of climate change, predation on postlarvae and fisheries. Mar Ecol Prog Ser. 2005; 287: 149-167. DOI: 10.3354/meps287149

Freitas V, Campos J, Fonds M, Van der Veer HW. Potential impact of temperature change on epibenthic predator–bivalve prey interactions in temperate estuaries. J Therm Bio. 2007; 32(6): 328-340. DOI: 10.1016/j.jtherbio.2007.04.004

Homer ML, Dungan CF, Tarnowski ML. Assessment of Chesapeake Bay commercial softshell clams *Mya arenaria* and *Tagelus plebeius* with emphasis on abundance and disease status. NOAA Chesapeake Bay Office Fisheries Science Program. 2011 Accessed online (August 2015): http://dnr2.maryland.gov/fisheries/Documents/NOAA_Final_report_softshell_clam.pdf

Newell CR, Hidu H. Species profiles: life histories and environmental requirements of coastal fishes and invertebrates (North Atlantic) – softshell clam. U.W. Fish Wildl. Serv. Biol. Rep. 82(11.53). U. S. Army Corps of Engineers, 1986. TR EL-82-4. 17pp. Accessed Online (August 2015): http://www.nwrc.usgs.gov/publications/specprof.htm

Ries JB, Cohen AL, McCorkle DC. Marine calcifiers exhibit mixed responses to CO2-induced ocean acidification. Geol. 2009; 37(12), 1131-1134. doi: 10.1130/G30210A.1

Waldbusser GG, Salisbury JE. Ocean acidification in the coastal zone from an organism's perspective: multiple system parameters, frequency domains, and habitats. Ann Rev Mar Sci. 2014; 6: 221-247. DOI: 10.1146/annurev-marine-121211-172238

Return to Table of Contents

Spanish Mackerel – *Scomberomorus maculatus*

Overall Vulnerability Rank = Moderate

Biological Sensitivity = Low
Climate Exposure = Very High
Data Quality = 83% of scores ≥ 2

Scomberomorus maculatus	Expert Scores	Data Quality	Expert Scores Plots (Portion by Category)
Stock Status	1.9	2.2	
Other Stressors	2.1	1.8	
Population Growth Rate	1.7	2.6	
Spawning Cycle	2.4	2.8	
Complexity in Reproduction	2.1	2.6	
Early Life History Requirements	2.3	1.2	
Sensitivity to Ocean Acidification	1.1	2.2	
Prey Specialization	1.3	2.8	
Habitat Specialization	1.6	3.0	
Sensitivity to Temperature	1.3	3.0	
Adult Mobility	1.3	2.4	
Dispersal & Early Life History	2.0	2.6	
Sensitivity Score	Low		
Sea Surface Temperature	4.0	3.0	
Variability in Sea Surface Temperature	1.0	3.0	
Salinity	3.2	3.0	
Variability Salinity	1.2	3.0	
Air Temperature	4.0	3.0	
Variability Air Temperature	1.0	3.0	
Precipitation	1.2	3.0	
Variability in Precipitation	1.3	3.0	
Ocean Acidification	4.0	2.0	
Variability in Ocean Acidification	1.0	2.2	
Currents	2.0	1.0	
Sea Level Rise	1.2	1.5	
Exposure Score	Very High		
Overall Vulnerability Rank	Moderate		

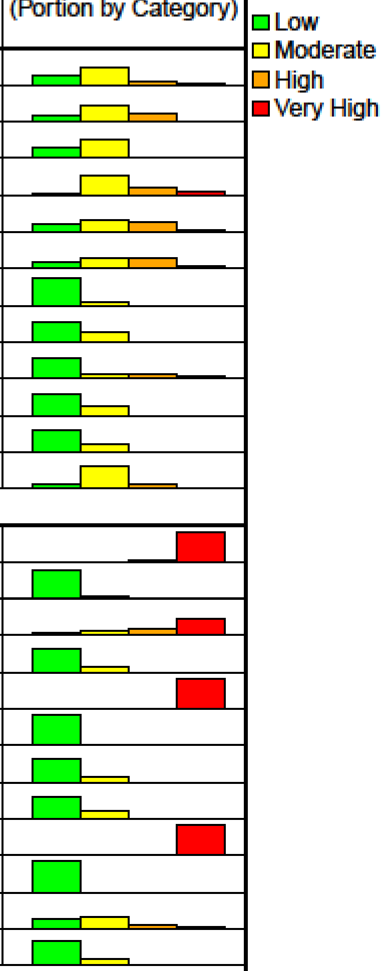

Return to Table of Contents

Spanish Mackerel (*Scomberomorus maculatus*)

Overall Climate Vulnerability Rank: **Moderate** (97% certainty from bootstrap analysis).

Climate Exposure: **Very High**. Three exposure factors contributed to this score: Ocean Surface Temperature (4.0), Ocean Acidification (4.0) and Air Temperature (4.0). Spanish Mackerel spend most of their life cycle in inner-shelf and coastal waters.

Biological Sensitivity: **Low**. No sensitivity attributes scored above 2.5.

Distributional Vulnerability Rank: **High** (99% certainty from bootstrap analysis). Three attributes indicated vulnerability to distribution shift. Spanish Mackerel are coastal and pelagic with minimal habitat requirements and make seasonal north-south migrations. Eggs and larvae are planktonic and susceptible to broad dispersal.

Directional Effect in the Northeast U.S. Shelf: The effect of climate change on Spanish Mackerel on the Northeast U.S. Shelf is very likely to be positive (>95% certainty in expert scores). Spanish Mackerel abundance in the Northeast U.S. Shelf Ecosystem increases in warm-years so the expectation is that abundance will continue to increase as warming continues. The effect of ocean acidification over the next 30 years is likely to be minimal.

Data Quality: 83% of the data quality scores were 2 or greater indicate that data quality is moderate.

Climate Effects on Abundance and Distribution: There is very little work on the effect of climate on the abundance and distribution of Spanish Mackerel in the Northeast U.S. Shelf Ecosystem. Chittenden et al (1993) reported that the abundance of Spanish Mackerel in the Chesapeake Bay region is quite variable but indicated that the causes of this variability were unknown. Gilmore et al. (1977) reported that Spanish Mackerel were killed during cold events in Florida indicating thermal sensitivity that could affect abundance and distribution.

Life History Synopsis**:** Spanish Mackerel is an epipelagic, neritic, migratory, marine finfish that occurs in coastal waters of the western Atlantic and Gulf of Mexico, but is rarely found north of New York (Collette, 2002). Adults reach sexual maturity at age 2-3 years (Collette, 2002). Spawning occurs in waters <50 m depth all along the United States east coast beginning in April off Florida to North Carolina and throughout the summer off Chesapeake Bay to New York (Buonaccorsi et al., 2001). Eggs are pelagic and hatch after 25 hours (Buonaccorsi et al., 2001). Larvae are pelagic and transform within 1-2 weeks (Buonaccorsi et al., 2001). Juveniles occur in coastal waters and estuaries (ASMFC, 2011). A warm water species, adult Spanish Mackerel over winter off Florida and move north as water temperatures rise in spring to spend summers north of Cape Hatteras, North Carolina; occasionally venturing as far as the Gulf of Maine (Collette, 2002; ASMFC, 2011). Spanish Mackerel primarily consume small fishes, but also prey on pandalid and penaeoid shrimps and squid (Collette, 2002). The Gulf of Mexico and Atlantic populations may not be genetically distinct (Buonaccorsi et al., 2001), but are managed separately (ASMFC, 2011). The Atlantic States Marine Fisheries Commission jointly manages the Atlantic population with the South Atlantic Fishery Management Council. Based on the most recent assessment, Spanish Mackerel are not overfished nor is overfishing occurring (SEDAR, 2012).

Return to Table of Contents

Literature Cited:

Atlantic States Marine Fisheries Commission (ASMFC). 2011. Omnibus amendment to the interstate fishery management plans for Spanish Mackerel, Spot, and Spotted Seatrout. 161 p. Accessed online (March 2015): http://www.asmfc.org/species/spanish-mackerel#stock

Atlantic States Marine Fisheries Commission: http://www.asmfc.org/species/shad-river-herring

Buonaccorsi VP; Starkey E; Graves JE. Mitochondrial and nuclear DNA analysis of population subdivision among young-of-the-year Spanish mackerel (Scomberomorus maculatus) from the western Atlantic and Gulf of Mexico. Mar Biol. 2001; 138: 37-45. doi: 10.1007/s002270000439

Chittenden Jr. ME, Barbieri LR, Jones CM. Fluctuations in abundance of Spanish mackerel in Chesapeake Bay and the mid-Atlantic region. North American Journal of Fisheries Management, 1993; 13(3), 450-458. doi: DOI:10.1577/1548-8675(1993)013<0450:FIAOSM>2.3.CO;2

Collette BB. Spanish Mackerel/ Scomberomorus maculatus (Mitchill 1815). In: Collete BB, Klein-MacPhee G, editors, Fishes of the Gulf of Maine, 3rd edition. Washington: Smithsonian Institution Press; 2002. pp. 530-531.

Gilmore RG, Bullock LH, Berry FH. Hypothermal mortality in marine fishes of south-central Florida January 1977. Northeast Gulf Science, 1978; 2(2): 77-97.

SouthEast Data, Assessment and Review (SEDAR). 2012. SEDAR 28- South Atlantic Spanish Mackerel stock assessment report. SEDAR, North Charleston SC. 444 p. Accessed Online (March 2015): http://sedarweb.org/sedar-28-stock-assessment-report-south-atlantic-spanish-mackerel

Return to Table of Contents

Spiny Dogfish – *Squalus acanthias*

Overall Vulnerability Rank = Low

Biological Sensitivity = Low
Climate Exposure = High
Data Quality = 92% of scores ≥ 2

Squalus acanthias	Expert Scores	Data Quality	Expert Scores Plots (Portion by Category)
Stock Status	1.9	3.0	
Other Stressors	1.2	2.4	
Population Growth Rate	3.8	3.0	
Spawning Cycle	2.0	2.4	
Complexity in Reproduction	1.4	2.0	
Early Life History Requirements	1.0	3.0	
Sensitivity to Ocean Acidification	1.1	3.0	
Prey Specialization	1.0	3.0	
Habitat Specialization	1.1	3.0	
Sensitivity to Temperature	1.4	2.8	
Adult Mobility	1.0	3.0	
Dispersal & Early Life History	1.0	3.0	
Sensitivity Score	Low		
Sea Surface Temperature	3.9	3.0	
Variability in Sea Surface Temperature	1.0	3.0	
Salinity	1.8	3.0	
Variability Salinity	1.2	3.0	
Air Temperature	1.0	3.0	
Variability Air Temperature	1.0	3.0	
Precipitation	1.0	3.0	
Variability in Precipitation	1.0	3.0	
Ocean Acidification	4.0	2.0	
Variability in Ocean Acidification	1.0	2.2	
Currents	2.1	1.0	
Sea Level Rise	1.2	1.5	
Exposure Score	High		
Overall Vulnerability Rank	Low		

Low
Moderate
High
Very High

Spiny Dogfish (*Squalus acanthias*)

Overall Climate Vulnerability Rank: **Low** (100% certainty from bootstrap analysis).

Climate Exposure: **High**. Two exposure factors contributed to this score: Ocean Surface Temperature (3.9) and Ocean Acidification (4.0). Spiny Dogfish are demersal / semi-pelagic and complete their life cycle in marine habitats.

Biological Sensitivity: **Low**. One attribute scored above 2.5: Population Growth Rate (2.8). Spiny Dogfish have low population growth rates (higher sensitivity to climate change) (Smith et al. 2008).

Distributional Vulnerability Rank: **Very High** (100% certainty from bootstrap analysis). Spiny Dogfish are habitat generalists and highly mobile as adults, making seasonal migrations. In addition, Spiny Dogfish are ovoviviparous and young are 20-33 cm at birth (Stehlik, 2007).

Directional Effect in the Northeast U.S. Shelf: The effect of climate change on Spiny Dogfish is very likely to be neutral (>95% certainty in expert scores). Spiny Dogfish inhabit cold-temperate waters and will likely not be affected by the magnitude of warming projected for the region. No changes in distribution have been observed over the past 30 years despite significant warming. Changes in productivity have not been documented.

Data Quality: 92% of the data quality scores were 2 or greater indicate that data quality is moderate.

Climate Effects on Abundance and Distribution: Little specific information exists on the effect of climate on Spiny Dogfish. In both the Northeast and Northwest Atlantic, no changes in distribution have been detected (Perry et al., 2005; Nye et al., 2009).

Life History Synopsis**:** Spiny Dogfish is a slow-growing, long-lived, elasmobranch species that occurs in temperate and boreal zones of the northern and southern hemisphere, and specifically from Greenland to northeastern Florida in the western Atlantic (Burgess, 2002). Spiny Dogfish females grow larger and older than males. Based on a recent study in the Gulf of Maine, 50% maturity for females is at 9.1years of age and 76.9 cm stretch total length), while males reach 50%maturity at 7.5 years and 63.1 cm stretch total length (Bubley et al., 2013). Mating involves internal fertilization, as is the case with all elasmobranchs (sharks, skates, and rays), and occurs in offshore areas during fall and winter (Burgess, 2002; Stehlik, 2007). This ovoviviparous species bears 1-15 pups after an 18-22 month gestation period (Burgess, 2002; NEFSC, 2006). The next batch of eggs begins forming while the female is still pregnant, so she can become pregnant again shortly after giving birth (Burgess, 2002). Parturition occurs on offshore wintering grounds from November to January, but can stretch into summer (Stehlik, 2007). Newborns range in size from 20-33 cm, and the sex ratio of the litter is usually 1:1 (Stehlik, 2007). Adults and Juveniles are found from inshore to offshore shelf waters, in a variety of temperatures and substrates, and can tolerate brackish water, but prefer full salinity water (Burgess, 2002; Stehlik, 2007). Spiny Dogfish form schools based on size with large mature females more common inshore, schools of mixed gender juveniles more common offshore, and schools of medium-sized immature females and mature males more common in the middle (Burgess, 2002). Seasonal migrations governed by the movement of prey bring Spiny Dogfish to the Gulf of Maine and areas north during summer and fall, and to offshore areas of the Mid-Atlantic and Southeastern United States Shelf to overwinter (Burgess, 2002). Juveniles prey on ctenophores, squid, and euphausiids, and begin substituting bivalves, decapods, and shrimp for plankton as they mature (Stehlik, 2007). Adults prey mostly on small

schooling fish such as Atlantic Herring and Atlantic Mackerel, but also consume molluscs, crustaceans, jellyfish, and solitary fish such as flatfish and Haddock (Burgess, 2002). Predators include: larger sharks, including conspecifics, Atlantic Cod, hakes, Monkfish (Goosefish), Striped Bass, and possibly whales, seals, and dolphins (Burgess, 2002; Stehlik, 2007). In state waters, Spiny Dogfish are managed under the Atlantic States Marine Fisheries Commission's Interstate fishery management plan (ASMFC, 2008). The Mid-Atlantic and New England Fishery Management Councils jointly manage the species in federal waters. Based on the most recent assessment, Spiny Dogfish were not overfished nor was overfishing occurring (NEFSC, 2006). A Transboundary Resources Assessment Committee assessment (a joint stock assessment with Canada) was completed in 2010, but it was not accepted (TRAC, 2010).

Literature Cited:

Atlantic States Marine Fisheries Commission (ASMFC). 2008. Management Report No. 46 of the Atlantic States Marine Fisheries Commission. 172p. Accessed online (August 2015): http://www.asmfc.org/uploads/file/interstateFMPforAtlanticCoastalSharks.pdf

Bubley WJ, Sulikowski JA, Koester DM, Tsang PCW. Using a multi-parameter approach to reassess maturity of spiny dogfish, *Squalus acanthias*, following increased fishing pressure in the Western North Atlantic. Fish Res. 2013; 147: 202-212. doi: 10.1016/j.fishres.2013.06.004

Burgess GH. Spiny Dogfish/ Squalus acanthias Linnaeus 1758. In: B.B. Collette BB, Klein-MacPhee G, editors, Fishes of the Gulf of Maine, 3rd ed. Washington: Smithsonian Institution Press; 2002. p. 54-57.

Northeast Fisheries Science Center (NEFSC). 2006. 43rd Northeast Regional Stock Assessment Workshop (43rd SAW): 43rd SAW assessment report. US Dep Commer, Northeast Fish Sci Cent Ref Doc 06-25; 400 p. Accessed online (August 2015): http://www.nefsc.noaa.gov/publications/crd/crd0625/

Nye JA, Link JS, Hare JA, Overholtz WJ. Changing spatial distribution of fish stocks in relation to climate and population size on the Northeast United States continental shelf. Mar Ecol Prog Ser. 2009; 393: 111-129. doi:10.3354/meps08220

Smith, S. E., Au, D. W., & Show, C. Intrinsic rates of increase in pelagic elasmobranchs. In: Camhi MD, Pikitch EK, Babcock EA, Editors, Sharks of the Open Ocean: Biology, Fisheries and Conservation. Hoboken: Wiley-Blackwell; 2008. p. 288-297.

Stehlik LL. 2007. Essential fish habitat source document: Spiny dogfish, Squalus acanthias, life history and habitat characteristics, 2nd edition. NOAA Tech Memo NMFS NE 203; 44 p. Accessed online (August 2015): http://www.nefsc.noaa.gov/nefsc/publications/tm/tm203/

Transboundary Resources Assessment Committee (TRAC). 2010. Northwest Atlantic spiny dogfish. DFO/NOAA Status Report 2010/02. Accessed online (August 2015): http://www2.mar.dfo-mpo.gc.ca/science/trac/TSRs/TSR_2010_02_E.pdf

Return to Table of Contents

Spot – *Leiostomus xanthurus*

Overall Vulnerability Rank = Moderate

Biological Sensitivity = Low
Climate Exposure = Very High
Data Quality = 92% of scores ≥ 2

Leiostomus xanthurus	Expert Scores	Data Quality	Expert Scores Plots (Portion by Category)
Stock Status	2.0	2.0	
Other Stressors	2.4	2.4	
Population Growth Rate	1.2	2.8	
Spawning Cycle	2.0	3.0	
Complexity in Reproduction	2.0	3.0	
Early Life History Requirements	2.5	2.9	
Sensitivity to Ocean Acidification	1.7	2.8	
Prey Specialization	1.2	3.0	
Habitat Specialization	1.8	3.0	
Sensitivity to Temperature	1.4	3.0	
Adult Mobility	1.4	3.0	
Dispersal & Early Life History	2.0	2.4	
Sensitivity Score	Low		
Sea Surface Temperature	4.0	3.0	
Variability in Sea Surface Temperature	1.0	3.0	
Salinity	3.0	3.0	
Variability Salinity	1.2	3.0	
Air Temperature	4.0	3.0	
Variability Air Temperature	1.0	3.0	
Precipitation	1.2	3.0	
Variability in Precipitation	1.3	3.0	
Ocean Acidification	4.0	2.0	
Variability in Ocean Acidification	1.0	2.2	
Currents	2.0	1.0	
Sea Level Rise	3.2	1.5	
Exposure Score	Very High		
Overall Vulnerability Rank	Moderate		

Low
Moderate
High
Very High

Spot (*Leiostomus xanthurus*)

Overall Climate Vulnerability Rank: **Moderate** (90% certainty from bootstrap analysis).

Climate Exposure: **Very High**. Three exposure factors contributed to this score: Ocean Surface Temperature (4.0), Ocean Acidification (4.0) and Air Temperature (4.0). Exposure to all three factors occur during all life stages. Spot spawn on the shelf, juveniles use estuarine nurseries, and adults make seasonal migrations from winter grounds offshore to feeding grounds in estuarine and coastal areas.

Biological Sensitivity: **Low**. Only one sensitivity attribute scored above 2.5: Early Life History Requirements (2.5). Spot exhibit an obligate estuarine-dependent life cycle, using estuaries during the late larval and juvenile stages (Able 2005).

Distributional Vulnerability Rank: **High** (96% certainty from bootstrap analysis). Three attributes indicated vulnerability to distribution shift. Spot larvae are widely dispersed after spawning, adults make seasonal north-south migrations along the East Coast of the United States, and adults are habitat generalists preferring coastal, nearshore and estuarine waters.

Directional Effect in the Northeast U.S. Shelf: The effect of climate change on Spot on the Northeast U.S. Shelf is very likely to be positive (>95% certainty in expert scores). Adult distribution will likely extend northwards as warming continues. The effect of ocean acidification over the next 30 years is likely to be minimal.

Data Quality: 92% of the data quality scores were 2 or greater.

Climate Effects on Abundance and Distribution: Wood and Austin (2009) described synchrony in the recruitment of three coastal spawning species in Chesapeake Bay: Atlantic Menhaden, Spot, and Summer Flounder. The generalized recruitment pattern was asynchronous with the recruitment of diadromous species in the Bay and the authors suggest large-scale climate forcing is responsible for the patterns in recruitment. Wingate and Secor (2008) associated high abundances of Spot with low winter river flows and high winter temperatures. These studies suggests that Spot productivity may change with changing climate. Distribution may also change with climate change. Murawski (1993) found that the maximum latitude of the occurrence of Spot in the region was related to temperature. Howell and Auster (2012) included Spot in the warm-temperate fauna of Long Island Sound, but found no trend in abundance over time. Walsh et al. (2015) indicated the presence of Spot larvae on the shelf in the fall suggesting that spawning is shifting northwards.

Life History Synopsis**:** Spot is an estuarine-dependent, coastal fish species found in inshore waters from southern New England to Mexico (Klein-MacPhee, 2002). Adults reach maturity at age 2-3 years (Able and Fahay, 2010). Spawning occurs in offshore waters during late fall to early spring primarily near the Gulf Stream Front off the coast of North Carolina and continental waters to the south (Klein-MacPhee, 2002; Able and Fahay, 2010). Eggs are pelagic and hatch after about 48 hours (Klein-MacPhee, 2002). Larvae are also pelagic and, over a period of about 2 months, transport from offshore spawning grounds to estuarine nursery grounds (Klein-MacPhee, 2002). Ingress into estuaries of the Mid-Atlantic states occurs during late winter – early spring, and larvae are sporadically abundant indicating that many mid-Atlantic recruits were likely larvae spawned south of Cape Hatteras (Able and Fahay, 2010). Late larvae enter bays and subtidal creeks, but move into intertidal and subtidal marshes as they grow (Able and Fahay, 2010). Spot can tolerate freshwater and full saline water, but usually occur in brackish waters

over a variety of substrates (Able and Fahay, 2010). Although some individuals overwinter in the estuary, most young Spot egress into coastal waters in fall and stay closer to shore than older juveniles and adults (Able and Fahay, 2010). Larvae consume tintinnids, pteropods, pelecypods, ostracods, and all stages of copepods (Klein-MacPhee, 2002). An ontogenetic shift to more benthic invertebrate prey occurs at approximately 30 mm, including: harpacticoids, maldanid and nereid polychaetes, nematodes, oligochaetes, clam siphons, and ostracods (Klein-MacPhee, 2002). Juveniles and adults occur over mud and sand bottom in inshore and estuarine waters out to 132 m water depth and can tolerate a wide range of temperatures and salinities (Klein-MacPhee, 2002). During spring and summer, Spot are found in estuaries and inshore waters, then migrate in schools to offshore spawning grounds during fall, and by late winter, are mostly found offshore south of Cape Hatteras (Klein-MacPhee, 2002). Like many species in the sciaenid family, Spot produce sounds when spawning, feeding, or alarmed (Klein-MacPhee, 2002). Adult Spot are benthic carnivores consuming polychaetes, crustaceans, bivalves, and occasionally fish (Klein-MacPhee, 2002). Several species of sharks, Monkfish (Goosefish), Bluefish, Weakfish, and Summer Flounder are common predators (Able and Fahay, 2010). There is no coastwide assessment for Spot currently. In state waters, the Atlantic States Marine Fisheries Commission coordinates management among states through an interstate fishery management plan (ASMFC, 2014).

Literature Cited:

Able KW. A re-examination of fish estuarine dependence: evidence for connectivity between estuarine and ocean habitats. Estuar Coast Shelf Sci. 2005; 64(1): 5-17. doi: doi:10.1016/j.ecss.2005.02.002

Able KW, Fahay MP. Ecology of estuarine fishes: temperate waters of the western North Atlantic. Baltimore: The Johns Hopkins University Press; 2010. 566p.

Atlantic States Marine Fisheries Commission (ASMFC). 2014. Addendum II to the omnibus amendment to the interstate fishery management plans for Spanish mackerel, spot, and spotted seatrout: Management of the Spot Fishery using the Traffic Light Approach. Accessed online (March 2015): http://www.asmfc.org/species/spot

Howell P, Auster PJ. Phase shift in an estuarine finfish community associated with warming temperatures. Mar Coast Fish. 2012; 4(1): 481-495. doi: 10.1080/19425120.2012.685144

Klein-MacPhee G. Spot/ Leiostomus xanthurus Lacepede 1802. In: Collete BB, Klein-MacPhee G, editors, Fishes of the Gulf of Maine, 3rd edition. Washington: Smithsonian Institution Press; 2002. pp. 440-442.

Murawski SA. Climate change and marine fish distributions: forecasting from historical analogy. Trans Am Fish Soc. 1993; 122(5): 647-658. doi: 10.1577/1548-8659(1993)122<0647:CCAMFD>2.3.CO;2

Walsh HJ, Richardson DE, Marancik KE, Hare JA. 2015. Long-term changes in the distributions of larval and adult fish in the Northeast U.S. Shelf Ecosystem. PLOS ONE. doi: 10.1371/journal.pone.0137382

Wingate RL, Secor DH. Effects of winter temperature and flow on a summer-fall nursery fish assemblage in the Chesapeake Bay, Maryland. Trans Am Fish Soc. 2008; 137(4): 1147-1156. doi: 10.1577/T07-098.1

[Return to Table of Contents](#)

Spotted Seatrout – *Cynoscion nebulosus*

Overall Vulnerability Rank = High

Biological Sensitivity = Moderate
Climate Exposure = Very High
Data Quality = 88% of scores ≥ 2

Cynoscion nebulosus	Expert Scores	Data Quality	Expert Scores Plots (Portion by Category)
Stock Status	2.1	1.6	
Other Stressors	2.5	2.6	
Population Growth Rate	1.7	2.7	
Spawning Cycle	2.2	2.6	
Complexity in Reproduction	2.4	2.5	
Early Life History Requirements	2.8	2.5	
Sensitivity to Ocean Acidification	1.4	2.8	
Prey Specialization	1.2	3.0	
Habitat Specialization	2.0	3.0	
Sensitivity to Temperature	1.4	3.0	
Adult Mobility	1.9	2.7	
Dispersal & Early Life History	2.4	2.9	
Sensitivity Score	Moderate		
Sea Surface Temperature	4.0	3.0	
Variability in Sea Surface Temperature	1.0	3.0	
Salinity	2.8	3.0	
Variability Salinity	1.2	3.0	
Air Temperature	4.0	3.0	
Variability Air Temperature	1.0	3.0	
Precipitation	1.2	3.0	
Variability in Precipitation	1.3	3.0	
Ocean Acidification	4.0	2.0	
Variability in Ocean Acidification	1.0	2.2	
Currents	2.0	1.0	
Sea Level Rise	3.4	1.5	
Exposure Score	Very High		
Overall Vulnerability Rank	High		

- Low
- Moderate
- High
- Very High

Spotted Seatrout (*Cynoscion nebulosus*)

Overall Climate Vulnerability Rank: **High** (68% certainty from bootstrap analysis).

Climate Exposure: **Very High**. Three exposure factors contributed to this score: Ocean Surface Temperature (4.0), Ocean Acidification (4.0) and Air Temperature (4.0). Exposure to all three factors occurs during all life stages. In most areas, Spotted Seatrout complete their entire life history within estuaries, with some movements onto the shelf in colder areas.

Biological Sensitivity: **Moderate**. Two sensitivity attributes scored above 2.5: Other Stressors and Early Life History Requirements (2.5). Deterioration of coastal waters due to urbanization, dredging, and other human activities has contributed to a decline in Spotted Seatrout abundance in some areas. Spotted Seatrout are estuarine and early life stages develop in estuaries (Bortone 2003) making them susceptible to changes in air and nearshore temperature changes.

Distributional Vulnerability Rank: **High** (54% certainty from bootstrap analysis). Two attributes indicated vulnerability to distribution shift. Spotted Seatrout have the potential to be dispersed during egg and larval stages although most development occurs within estuaries. Adults are mobile and make seasonal migrations in colder areas including the Northeast U.S. Shelf.

Directional Effect in the Northeast U.S. Shelf: The effect of climate change on Spotted Seatrout on the Northeast U.S. Shelf is estimated to be neutral, but with a high degree of uncertainty (<66% certainty in expert scores). The uncertainty likely stems from the general lack of data on the species in the region. Adult distribution may extend northwards as warming continues, but the magnitude of this extension could be minimal over the next 30 years. The effect of ocean acidification over the next 30 years is likely to be minimal.

Data Quality: 88% of the data quality scores were 2 or greater indicate that data quality is moderate.

Climate Effects on Abundance and Distribution: Relatively little information exists regarding the role of climate in affecting Spotted Seatrout productivity or distribution. Working in the Gulf of Mexico, Froeschke and Froeschke (2011) found that distribution of juvenile Spotted Seatrout was strongly associated with temperature and salinity. Working in Florida Bay, Kearney et al. (2014) found minimal decreases in Spotted Seatrout habitat availability under several climate change scenarios. These studies suggest that as the Northeast U.S. Shelf warms, areas may become more favorable to Spotted Seatrout.

Life History Synopsis**:** Spotted Seatrout is an estuarine-dependent fish species of the western north Atlantic that occurs from Cape Cod, Massachusetts, to Florida and the Gulf of Mexico, but is rare north of Delaware Bay (Able and Fahay, 2010). Adults are mature by age 2, and spawning occurs from May to August with a peak from late May – early June (Able and Fahay, 2010). Spotted Seatrout are highly fecund and spawn repeatedly during their relatively long spawning season (ASMFC, 2011). Spawning occurs in a variety of estuarine habitats including seagrass beds, sandy banks, shell reefs, and inlets, but seagrass beds in warm water with salinities > 15 seem to be the most used (ASMFC, 2011). Eggs and larvae are pelagic and remain in the estuaries or bays where they were spawned (Able and Fahay, 2010). Seagrass beds are important habitat throughout the life of Spotted Seatrout, and while juveniles settle into a variety of estuarine habitats, seagrass beds and marsh edge habitat are the most used (Able and Fahay, 2010; ASMFC, 2011). Juveniles travel in schools for several years and inhabit larger tributaries and lower estuarine habitat (ASMFC, 2011). Juveniles prey on benthic invertebrates, especially

copepods, mysid shrimp, and as they get larger, penaeid and palaemonid shrimp (ASMFC, 2011). Many larger estuarine fish species are predators of juvenile Spotted Seatrout (Able and Fahay, 2010). Juveniles and adults are, in general, non-migratory, staying within 10-15 km of their natal estuary (ASMFC, 2011). However, movement to deeper warmer water during cold weather has been observed, and the population north of Cape Hatteras may migrate to coastal ocean waters off North Carolina during late fall and winter and back into estuaries and bays in spring and summer (Able and Fahay, 2010). Adult Spotted Seatrout are piscivorous, but also feed on decapod crustaceans such as penaeid and palaemonid shrimp (Able and Fahay, 2010; ASMFC, 2011). Striped Bass, Croaker, Barracuda, and Tarpon are common predators of the species (Able and Fahay, 2010). There is no coast-wide assessment of Spotted Seatrout. The Atlantic States Marine Fisheries Commission coordinates management, but assessments are conducted on a state-by-state basis (ASMFC, 2011). Stocks in the northern part of the range are experiencing overfishing, but the southern stocks appear to be sustainably harvested (ASMFC, 2011).

Literature Cited:

Able KW, Fahay MP. Ecology of estuarine fishes: temperate waters of the western North Atlantic. Baltimore: The Johns Hopkins University Press; 2010. 566p.

Atlantic States Marine Fisheries Commission (ASMFC). 2011. Omnibus Amendment to the Interstate Fishery Management Plans for Spanish Mackerel, Spot, and Spotted Seatrout. Fishery Management Report. 143p. Accessed online (April 2015): http://www.asmfc.org/species/spotted-seatrout

Bortone SA. Biology of the Spotted Seatrout. Boca Raton: CRC Press; 2003.

Froeschke JT, Froeschke BF. Spatio-temporal predictive model based on environmental factors for juvenile spotted seatrout in Texas estuaries using boosted regression trees. Fish Res. 2011; 111(3): 131-138. doi:10.1016/j.fishres.2011.07.008

Kearney KA, Butler M, Glazer R, Kelble CR, Serafy JE, Stabenau E. Quantifying Florida Bay Habitat Suitability for Fishes and Invertebrates Under Climate Change Scenarios. Environ Manag. 2015; 55(4): 836-856. doi: 10.1007/s00267-014-0336-5

Return to Table of Contents

Striped Bass – *Morone saxatilis*

Overall Vulnerability Rank = Very High

Biological Sensitivity = High
Climate Exposure = Very High
Data Quality = 88% of scores ≥ 2

Morone saxatilis	Expert Scores	Data Quality	Expert Scores Plots (Portion by Category)
Stock Status	2.0	1.4	
Other Stressors	2.6	2.6	
Population Growth Rate	2.6	2.8	
Spawning Cycle	3.3	3.0	
Complexity in Reproduction	2.3	2.8	
Early Life History Requirements	3.0	3.0	
Sensitivity to Ocean Acidification	1.2	3.0	
Prey Specialization	1.2	3.0	
Habitat Specialization	1.9	3.0	
Sensitivity to Temperature	1.7	3.0	
Adult Mobility	1.6	3.0	
Dispersal & Early Life History	2.4	3.0	
Sensitivity Score	High		
Sea Surface Temperature	4.0	3.0	
Variability in Sea Surface Temperature	1.0	3.0	
Salinity	1.8	3.0	
Variability Salinity	1.2	3.0	
Air Temperature	4.0	3.0	
Variability Air Temperature	1.0	3.0	
Precipitation	1.2	3.0	
Variability in Precipitation	1.3	3.0	
Ocean Acidification	4.0	2.0	
Variability in Ocean Acidification	1.0	2.2	
Currents	2.0	1.0	
Sea Level Rise	2.5	1.5	
Exposure Score	Very High		
Overall Vulnerability Rank	Very High		

Low
Moderate
High
Very High

Return to Table of Contents

Striped Bass (*Morone saxatilis*)

Overall Climate Vulnerability Rank: **Very High** (69% certainty from bootstrap analysis).

Climate Exposure: **Very High**. Three exposure factors contributed to this score: Ocean Surface Temperature (4.0), Ocean Acidification (4.0) and Air Temperature (4.0). Exposure to all three factors occurs during all life stages. Striped Bass spawn and develop in freshwater and low salinity habitats, while adults make seasonal north-south migrations in the coastal ocean.

Biological Sensitivity: **High**. Two sensitivity attributes scored above 3: Spawning Cycle (3.3) and Early Life History Requirements. Spawning occurs in the spring on the freshwater side of the salt-wedge. Eggs and larvae develop in brackish waters. Striped Bass are obligate estuarine dependent (Able 2005).

Distributional Vulnerability Rank: **High** (81% certainty from bootstrap analysis). Two attributes indicated vulnerability to distribution shift. Striped Bass are highly mobile making large seasonal north-south migrations. They also use a variety of estuarine, nearshore, and coastal habitats.

Directional Effect in the Northeast U.S. Shelf: The effect of climate change on Striped Bass on the Northeast U.S. Shelf is estimated to be neutral, but with a moderate degree of uncertainty (66-90% certainty in expert scores). The uncertainty likely stems from the complex life history and the potential for different aspects of climate change to effect the species differently. Increasing temperatures could reduce habitat in the southern part of the Northeast U.S. Shelf while increasing habitat in the northern portions. Higher precipitation may increase recruitment, but combined with sea-level rise may decrease the salt-wedge are where Striped Bass spawn. The effect of ocean acidification over the next 30 years is likely to be minimal.

Data Quality: 88% of the data quality scores were 2 or greater.

Climate Effects on Abundance and Distribution: A number of studies indicated that Striped Bass productivity can be influenced by climate change. Hurst and Conover (1998) showed that temperature-induced overwinter mortality of juveniles was important to recruitment in northern portions of the range. Further, Coutant and Benson (1990) indicated that increasing summer temperatures resulted in a reduction of habitat in Chesapeake Bay. Egg and larval distribution relative to the position of the salt-wedge and estuarine turbidity maximum also are important to recruitment (North and Houde 2003). O'Connor et al. (2012) found that larval abundance was greater in the Hudson River in years with higher freshwater inputs. Thus, changes in temperature, precipitation and sea-level rise have the potential to affect population productivity.

Life History Synopsis**:** Striped Bass is a freshwater, estuarine, and coastal-anadromous species found from the lower St Lawrence and southern Gulf of St. Lawrence to Florida and the northern shore of the Gulf of Mexico as well as being introduced to rivers, reservoirs, and estuaries throughout the United States and several places in Europe (Klein-MacPhee, 2002). Females reach sexual maturity between 4-6 years, and males at 2-3 years or possibly later (Klein-MacPhee, 2002). Spawning occurs from February to July in freshwater usually within 40 km of the salt-wedge as temperatures rise above 11-14°C (Klein-MacPhee, 2002; Able and Fahay, 2010). Striped Bass return to natal rivers throughout their eastern United States range, but the majority of spawning in the northeastern US occurs in the Chesapeake Bay, the Delaware Bay, and the Hudson River in areas with a strong current (Klein-MacPhee, 2002; Able and Fahay, 2010). Striped Bass are highly fecund broadcast spawners producing large, semi-buoyant eggs

that require a strong current to stay afloat (Klein-MacPhee, 2002). Incubation takes about 2 days, or up to 5 days in cooler water, with survival dependent on low salinity and temperatures <27°C, although several water quality parameters can mean the difference between survival of the egg or not (Klein-MacPhee, 2002). Eggs and larvae follow currents downstream to the lower tidal reaches, but survival rates are higher in low salinity water (Able and Fahay, 2010). Larvae occur in 13-22C water with larval durations of about 1 month on average, but up to 2 months in cooler water (Klein-MacPhee, 2002). Larvae consume copepods and cladocerans as big as they can catch (Klein-MacPhee, 2002). Many larger fish likely prey on larval Striped Bass, but they are rarely seen in the stomachs of wild-caught fish (Klein-MacPhee, 2002). Juvenile habitat includes shallow waters with sluggish currents, sand or gravel bottom, and dissolved oxygen levels >3mg l^{-1} (Klein-MacPhee, 2002; Able and Fahay, 2010). Early juveniles consume a variety of prey including insect larvae, polychaete worms, larval fishes, mysids, amphipods, and Crangon shrimps, but begin consuming a higher proportion of small fishes as they grow (Klein-MacPhee, 2002; Able and Fahay, 2010). Schooling behavior is common among juveniles and small adults (up to 4.5 kg) (Klein-MacPhee, 2002). Most adults larger than 4.5 kg are found singly (Klein-MacPhee, 2002). There is a large degree of variability in the range of migrations and the use of oceanic habitat. In general, there are resident populations throughout the range of the species that remain in freshwater or river/estuarine systems for the duration of their life (Klein-MacPhee, 2002). Between Cape Hatteras and New England, there are also migratory anadromous populations whose members stay within natal estuaries for 1-3 years before leaving the estuary for coastal summer habitat (Klein-MacPhee, 2002; Able and Fahay, 2010; NEFSC, 2013). The amount and distance of migrations increases with fish size, so large females are the most likely to migrate and to migrate the farthest in a season (Klein-MacPhee, 2002; Able and Fahay, 2010). Oceanic migrations tend to be to the north and east during spring and to the south and west, returning to estuaries, during fall, rarely venturing farther than 6-8 km from shore, and often observed feeding near beaches (Klein-MacPhee, 2002; Able and Fahay, 2010; NEFSC, 2013). Adults are voracious predators and feed on a variety of seasonally available freshwater and marine fish and invertebrates, including crustaceans, worms, squid, soft clams, mussels, and small fish (Klein-MacPhee, 2002). Large Bluefish, Weakfish, and possibly Atlantic Cod, Silver Hake, and larger Striped Bass feed on Striped Bass, but humans are the major predator of the species (Klein-MacPhee, 2002). Striped Bass are managed as a single stock from North Carolina to Maine; however, the Albemarle Sound/Roanoke River population is managed separately due to the perception of low contribution rates to the northern populations (NEFSC, 2013). The National Marine Fisheries Service conducts a coast-wide assessment, but management is coordinated among states by the Atlantic States Marine Fisheries Commission (NEFSC, 2013). Commercial fishing is prohibited in New Jersey, Pennsylvania, Connecticut, New Hampshire, Maine, and the District of Columbia, but recreational fisheries exist throughout the stock region (NEFSC, 2013). The stock is not overfished or experiencing overfishing as of 2012 (NEFSC, 2013).

Literature Cited:

Able KW. A re-examination of fish estuarine dependence: evidence for connectivity between estuarine and ocean habitats. Estuar Coast Shelf Sci. 2005; 64(1): 5-17. doi: doi:10.1016/j.ecss.2005.02.002

Able KW, Fahay MP. Ecology of estuarine fishes: temperate waters of the western North Atlantic. Baltimore: The Johns Hopkins University Press; 2010. 566p.

Coutant CC, Benson DL. Summer habitat suitability for striped bass in Chesapeake Bay: reflections on a population decline. Trans Am Fish Soc. 1990; 119(4): 757-778. doi: 10.1577/1548-8659(1990)119<0757:SHSFSB>2.3.CO;2

Hurst TP, Conover DO. Winter mortality of young-of-the-year Hudson River striped bass (Morone saxatilis): size-dependent patterns and effects on recruitment. Can J Fish Aquat Sci. 1998; 55(5): 1122-1130. doi: 10.1139/cjfas-55-5-1122

Klein-MacPhee G. Striped Bass/ Morone saxatilis (Walbaum 1792). In: Collete BB, Klein-MacPhee G, editors, Fishes of the Gulf of Maine, 3rd edition. Washington: Smithsonian Institution Press; 2002. pp. 377-389.

North EW, Houde ED. Linking ETM physics, zooplankton prey, and fish early-life histories to white perch (Morone americana) and striped bass (M. saxatilis) recruitment success. Mar Ecol Prog Ser. 2003; 260: 219-236. doi:10.3354/meps260219

Northeast Fisheries Science Center (NEFSC). 2013. 57th Northeast Regional Stock Assessment Workshop (57th SAW) Assessment Report. US Dept Commer, Northeast Fish Sci Cent Ref Doc. 13-16; 967 p. Available from: National Marine Fisheries Service, 166 Water Street, Woods Hole, MA 02543-1026. Accessed online (April 2015): http://www.nefsc.noaa.gov/publications/crd/crd1316/

O'Connor MP, Juanes F, McGarigal K, Gaurin S. Findings on American Shad and striped bass in the Hudson River Estuary: a fish community study of the long-term effects of local hydrology and regional climate change. Mar Coast Fish. 2012; 4(1): 327-336. doi: 10.1080/19425120.2012.675970

Return to Table of Contents

Summer Flounder – *Paralichthys dentatus*

Overall Vulnerability Rank = Moderate

Biological Sensitivity = Low
Climate Exposure = Very High
Data Quality = 92% of scores ≥ 2

Paralichthys dentatus	Expert Scores	Data Quality	Expert Scores Plots (Portion by Category)
Stock Status	2.0	3.0	
Other Stressors	2.3	2.6	
Population Growth Rate	2.0	2.6	
Spawning Cycle	2.0	3.0	
Complexity in Reproduction	1.3	2.8	
Early Life History Requirements	2.2	2.7	
Sensitivity to Ocean Acidification	1.3	2.4	
Prey Specialization	1.2	3.0	
Habitat Specialization	1.4	3.0	
Sensitivity to Temperature	1.2	3.0	
Adult Mobility	1.5	3.0	
Dispersal & Early Life History	1.9	2.8	
Sensitivity Score	Low		
Sea Surface Temperature	4.0	3.0	
Variability in Sea Surface Temperature	1.0	3.0	
Salinity	2.6	3.0	
Variability Salinity	1.2	3.0	
Air Temperature	4.0	3.0	
Variability Air Temperature	1.0	3.0	
Precipitation	1.2	3.0	
Variability in Precipitation	1.3	3.0	
Ocean Acidification	4.0	2.0	
Variability in Ocean Acidification	1.0	2.2	
Currents	2.0	1.0	
Sea Level Rise	2.4	1.5	
Exposure Score	Very High		
Overall Vulnerability Rank	Moderate		

Low
Moderate
High
Very High

Summer Flounder (*Paralichthys dentatus*)

Overall Climate Vulnerability Rank: **Moderate** (100% certainty from bootstrap analysis).

Climate Exposure: **Very High**. Three exposure factors contributed to this score: Ocean Surface Temperature (4.0), Ocean Acidification (4.0) and Air Temperature (4.0). Summer Flounder is an obligate estuarine-dependent species (Able, 2005), spawns on the shelf and juveniles develop in estuaries. Adults make seasonal north-south migrations exposing them to changing condition inshore and offshore.

Biological Sensitivity: **Low**. No sensitivity attributes scored above 2.5.

Distributional Vulnerability Rank: **High** (100% certainty from bootstrap analysis). Three attributes indicated vulnerability to distribution shift. Summer flounder spawning in shelf waters and eggs and larvae are broadly dispersed. Adults make regional-scale north-south migrations seasonally. Adults use a range of habitats including estuarine, coastal, and shelf. The life history of the species has a strong potential to enable shifts in distribution.

Directional Effect in the Northeast U.S. Shelf: The effect of climate change on Summer Flounder on the Northeast U.S. Shelf is estimated to be neutral but with high uncertainty (<66% certainty in expert scores). Adult distribution have shifted northward, but this is linked to changes in fishing. Also, productivity of the stock has remained fairly constant over the past 3 decades, during which temperatures in the ecosystem have increased. The effect of ocean acidification over the next 30 years is likely to be minimal. However, there are many aspects of the life history which could result in a positive effect resulting from climate change and these lead to the expert uncertainty.

Data Quality: 92% of the data quality scores were 2 or greater.

Climate Effects on Abundance and Distribution: Wood and Austin (2009) described synchrony in the recruitment of three coastal spawning species in Chesapeake Bay: Atlantic Menhaden, Spot, and Summer Flounder. The generalized recruitment pattern was asynchronous with recruitment of diadromous species in the Bay and the authors suggest large-scale climate forcing is responsible for the patterns in recruitment. This study suggests that Summer Flounder productivity may change with changing climate. Recent changes in Summer Flounder distribution also have been identified and linked to climate (Pinsky et al 2013), but Bell et al. (2014) presented evidence that changes in Summer Flounder distribution were linked to reductions in fishing and expanding population rather than changes in temperature. Murawski (1993) also documented changes in Summer Flounder distribution related to abundance and not temperature. These results do not mean that climate does not affect distribution, but rather changes in abundance have a much greater effect, pointing to the important role of fishing in determining fish abundance and distribution. Chambers et al. (2014) found that survival of Summer Flounder eggs and larvae decreased with increasing CO_2 concentrations. However, the experimental conditions were extreme relative to the expected changes in CO_2 concentrations in the next several decades, thus the effects of ocean acidification in the near-term remain unclear.

Life History Synopsis**:** Summer Flounder is an estuarine-dependent, marine, benthic flatfish species that is found from Nova Scotia to Florida, but mostly occurs from Cape Cod, Massachusetts, to Cape Hatteras, North Carolina (Packer et al., 1999). Most Summer Flounder are sexually mature by age 2, and females reach larger sizes than males (Packer et al., 1999). North of Cape Hatteras, spawning occurs from September through January or as late as early March (peaking October to November) beginning

inshore and continuing as the adults migrate to offshore winter habitats (Packer et al., 1999; Able and Fahay, 2010). Summer Flounder are highly fecund serial spawners producing thousands to millions of pelagic eggs (Packer et al., 1999). Eggs hatch after 2-9 days depending on water temperature (Packer et al., 1999; Able and Fahay, 2010). Larvae are planktonic and are transported inshore eventually reaching estuaries (Packer et al., 1999; Able and Fahay, 2010). While offshore, survival is influenced by temperature, salinity, and the availability of prey as the yolk runs out (Packer et al., 1999). Larvae feed during the day on copepod nauplii, copepodites, and tintinnids, changing to larger prey including calanoid copepods, and appendicularians as they approach the coast (Klein-MacPhee, 2002; Able and Fahay, 2010). Beginning while still at sea, Summer Flounder larvae undergo a significant metamorphosis where the right eye migrates over the head to the left side (Packer et al., 1999). The duration of this stage depends on temperature and can last 3 weeks to 3 months (Klein-MacPhee, 2002; Able and Fahay, 2010). Larvae ingress to estuaries during late fall to early spring at approximately 13 mm standard length (Able and Fahay, 2010). The diet of post-larvae in estuaries relies heavily on the calanoid copepod, *Temora longicornis* (Able and Fahay, 2010). After completing metamorphosis, juveniles settle on estuarine soft sediments and begin exhibiting burying behavior (Packer et al., 1999). Recently settled juveniles occur in a variety of estuarine habitats including: marsh creeks, seagrass beds, mud flats, open bays, and shallow coves with mud, sand, and shell hash substrates, and remain in the same area for several months (Packer et al., 1999; Able and Fahay, 2010). Juveniles are most abundant in estuaries from Virginia to South Carolina (Able and Fahay, 2010). Many juveniles from southern estuaries remain in the estuary for up to 20 months, while northern estuary inhabitants often move to just outside the estuary in winter and return to the same estuary the next spring (Packer et al., 1999; Klein-MacPhee, 2002). The juvenile diet shifts from invertebrates, such as mysids, shrimp, polychaetes, copepods, and crabs, to a diet dominated by blue crabs and small fishes such as juvenile Winter Flounder, silversides, mummichogs, and Spot (Klein-MacPhee, 2002; Able and Fahay, 2010). Crangon shrimp and blue crabs are also common predators of juvenile flounder (Able and Fahay, 2010). Adults are common in estuarine and coastal waters from spring through fall near eelgrass beds and salt marshes in muddy or silty sediments (Klein-MacPhee, 2002; Able and Fahay, 2010). In fall, starting with the oldest and largest individuals, adults and large juveniles migrate offshore to sand or mud bottom on the outer continental shelf to overwinter (Klein-MacPhee, 2002; Able and Fahay, 2010). By late spring the adults migrate back to coastal waters, often to the same estuaries year after year; although, some large individuals stay offshore even during summer (Able and Fahay, 2010). Bony fish, such as sand lance, anchovies, hakes, and other flatfish are the dominant prey of these active hunters (Klein-MacPhee, 2002; Able and Fahay, 2010). Squid, decapod shrimp, and small crustaceans are also important prey (NEFSC, 2013). Spiny Dogfish, Blue Shark, Little Skate, Winter Skate, Atlantic Cod, Silver Hake, Monkfish (Goosefish), Northern Sea Robin, Spot, Bluefish, and Winter Flounder are all predators of Summer Flounder (Klein-MacPhee, 2002; NEFSC, 2013). The Atlantic States Marine Fisheries Commission (state waters) and Mid-Atlantic Fishery Management Council (federal waters) cooperatively manage Summer Flounder as a single stock from the Canadian border to the southern border of North Carolina (NEFSC, 2013). Summer Flounder are not overfished nor is overfishing occurring and the stock was declared rebuilt in 2010 (NEFSC, 2013).

Literature Cited:

Able KW. A re-examination of fish estuarine dependence: evidence for connectivity between estuarine and ocean habitats. Estuar Coast Shelf Sci. 2005; 64(1): 5-17. doi: doi:10.1016/j.ecss.2005.02.002

Able KW, Fahay MP. Ecology of estuarine fishes: temperate waters of the western North Atlantic. Baltimore: The Johns Hopkins University Press; 2010. 566p.

Bell RJ, Richardson DE, Hare JA, Lynch PD, Fratantoni PS. Disentangling the effects of climate, abundance, and size on the distribution of marine fish: an example based on four stocks from the Northeast US shelf. ICES J Mar Sci. 2014; fsu217. doi: 10.1093/icesjms/fsu217

Chambers RC, Candelmo AC, Habeck EA, Poach ME, Wieczorek D, Cooper KR, et al. Effects of elevated CO 2 in the early life stages of summer flounder, Paralichthys dentatus, and potential consequences of ocean acidification. Biogeosciences, 2014; 11(6): 1613-1626. doi:10.5194/bg-11-1613-2014

Klein-MacPhee G. Summer Flounder/ Paralichthys dentatus (Linnaeus 1766). In: Collete BB, Klein-MacPhee G, editors, Fishes of the Gulf of Maine, 3rd edition. Washington: Smithsonian Institution Press; 2002. pp. 554-559.

Murawski SA. Climate change and marine fish distributions: forecasting from historical analogy. Trans Am Fish Soc. 1993; 122(5): 647-658. doi: 10.1577/1548-8659(1993)122<0647:CCAMFD>2.3.CO;2

Northeast Fisheries Science Center (NEFSC). 2013. 57th Northeast Regional Stock Assessment Workshop (57th SAW) Assessment Report. US Dept Commer, Northeast Fish Sci Cent Ref Doc. 13-16; 967 p. Available from: National Marine Fisheries Service, 166 Water Street, Woods Hole, MA 02543-1026. Accessed online (March 2015): http://www.nefsc.noaa.gov/publications/crd/crd1316/

Nye JA, Link JS, Hare JA, Overholtz WJ. Changing spatial distribution of fish stocks in relation to climate and population size on the Northeast United States continental shelf. Mar Ecol Prog Ser. 2009; 393: 111-129. doi:10.3354/meps08220

Packer DB, Griesbach SJ, Berrien PL, Zetlin CA, Johnson DL, Morse WW. 1999. Essential fish habitat source document: Summer flounder, Paralichthys dentatus, life history and habitat characteristics. NOAA Tech Memo NMFS NE 151; 88 p. Accessed online (March 2015): http://www.nefsc.noaa.gov/nefsc/publications/tm/tm151/

Return to Table of Contents

Tautog – *Tautoga onitis*

Overall Vulnerability Rank = Very High

Biological Sensitivity = High
Climate Exposure = Very High
Data Quality = 83% of scores ≥ 2

Tautoga onitis	Expert Scores	Data Quality	Expert Scores Plots (Portion by Category)
Stock Status	2.1	0.8	
Other Stressors	2.6	2.8	
Population Growth Rate	3.3	2.6	
Spawning Cycle	2.5	3.0	
Complexity in Reproduction	1.6	2.6	
Early Life History Requirements	2.4	1.4	
Sensitivity to Ocean Acidification	2.2	2.8	
Prey Specialization	1.9	3.0	
Habitat Specialization	2.4	3.0	
Sensitivity to Temperature	2.4	3.0	
Adult Mobility	3.1	2.8	
Dispersal & Early Life History	2.2	2.6	
Sensitivity Score	High		
Sea Surface Temperature	4.0	3.0	
Variability in Sea Surface Temperature	1.0	3.0	
Salinity	2.1	3.0	
Variability Salinity	1.2	3.0	
Air Temperature	4.0	3.0	
Variability Air Temperature	1.0	3.0	
Precipitation	1.2	3.0	
Variability in Precipitation	1.3	3.0	
Ocean Acidification	4.0	2.0	
Variability in Ocean Acidification	1.0	2.2	
Currents	2.0	1.0	
Sea Level Rise	1.7	1.5	
Exposure Score	Very High		
Overall Vulnerability Rank	Very High		

Low
Moderate
High
Very High

Tautog (*Tautoga onitis*)

Overall Climate Vulnerability Rank: **Very High** (79% certainty from bootstrap analysis).

Climate Exposure: **Very High**. Three exposure factors contributed to this score: Ocean Surface Temperature (4.0), Ocean Acidification (4.0) and Air Temperature (4.0). Exposure to all three factors occur during the life stages. Tautog uses coastal and nearshore habitats during all life stages, and utilize offshore habitat in the winter.

Biological Sensitivity: **High**. Two sensitivity attributes scored above 3.0: Adult Mobility (3.1) and Population Growth Rate (3.3); and was at or above Tautog generally remain within the nearshore environment during all seasons with some offshore movement, particularly in the northern part of their range (Arendt et al. 2001). They are a relatively long-lived fish with low population growth rates potentially making them vulnerable to climate change (Steimle and Shaheen, 1999). However, in the southern part of the ecosystem

Distributional Vulnerability Rank: **Moderate** (82% certainty from bootstrap analysis). Three attributes indicated limited vulnerability to distribution shift: limited adult mobility, limited early life stage dispersal, and relatively high habitat specialization.

Directional Effect in the Northeast U.S. Shelf: The effect of climate change on Tautog on the Northeast U.S. Shelf is estimated to be neutral but with a moderate degree of uncertainty (66-90% certainty in expert scores). The range of Tautog extend just south of the Northeast U.S. Shelf and a northward shift would negatively affect Tautog in the region. However, no northward shift has been documented despite decades of warming. In addition Tautog primarily feed on molluscs and crustaceans and ocean acidification may weaken shells and thereby make prey more vulnerable to predation. Again, there is no evidence to support these suppositions and thus, the neutral effect of climate change and moderate uncertainty.

Data Quality: 88% of the data quality scores were 2 or greater indicate that data quality is moderate.

Climate Effects on Abundance and Distribution: There is relatively little information regarding the effect of climate on Tautog productivity. Temperature is important in triggering migratory behavior in the fall (Olla et al. 1980) and spawning in the late-spring and summer (Gauthier et al. 2008). Thus, changes in temperature have the potential to effect life history phenology, but whether these potential effects will impact productivity in unknown. Similarly, climate effects on Tautog distribution are unclear. Tautog were grouped with the cold-temperature species in Long Island Sound, New York and Narragansett Bay, Rhode Island and abundances have decreased over time (Howell and Auster, 2012; Collie et al., 2008), but there are no large-scale studies examining regional scale distribution. The low mobility of the different life stages suggest that the species would have limited ability to shift their range in the face of climate change.

Life History Synopsis**:** Tautog is a slow-growing, long-lived, coastal and estuarine species that occurs from Nova Scotia to South Carolina, but is primarily found from Cape Cod to Delaware Bay (Able and Fahay, 2010). Tautog are gonochoristic, and while males may begin maturing earlier than females, most fish reach maturity at 3-4 years (ASMFC, 2015). Spawning occurs between April and September, beginning in the southern portion of their range, at or near the mouth of estuaries and to a limited degree on wrecks and reefs on the inner shelf, with individuals generally returning to the same area

each year (Munroe, 2002; Able and Fahay, 2010; ASMFC, 2015). The females of the species increase fecundity, number of batches per season, and egg quality with size (Able and Fahay, 2010; ASMFC, 2015). Gametes are released in near-surface waters, and eggs are usually found in estuaries and sporadically on the inner-shelf and near beaches (Munroe, 2002; ASMFC, 2015). Incubation takes 2-7 days depending on temperature, and temperature >22°C may impede embryonic and larval development (Munroe, 2002). Copepods are known to eat Tautog eggs (Able and Fahay, 2010). Larvae are planktonic for approximately 2-3 weeks and occur along the estuary-ocean gradient and near beaches, but move deeper and farther from shore later in the larval stage (Able and Fahay, 2010). Tautog settle to shallow vegetated areas of the estuary, particularly where sea lettuce or other macroalgae are present, then move to deeper nearshore areas with eelgrass or structure, such as rock, jetties, or shipwrecks, as they mature (Munroe, 2002). Most juveniles remain in estuaries year round, but by spring, some juveniles are associated with structure on the inner shelf (Munroe, 2002). Young Tautog consume copepods, amphipods, and some isopods and decapods, but rely more heavily on mussels as they grow (Munroe, 2002; Able and Fahay, 2010). Adult Tautog are sometimes found feeding on sandy bottom, but generally require structure such as rocky reefs, pilings, jetties, boulders, rubble, or mussel beds (Munroe, 2002; Able and Fahay, 2010). All feeding and activity occurs during the day, with larger fish moving farther away from the home site than smaller fish (Munroe, 2002; ASMFC, 2015). Tautog consume a variety of invertebrates, especially molluscs, barnacles, small crustaceans, echinoderms, and some small fishes (Munroe, 2002). All fish return to their shelter at night where they are inactive and possibly sleep (Munroe, 2002; ASMFC, 2015). The southern portion of the population remains in the estuaries year round, and a portion of the northern population remains on the inner shelf year round (Munroe, 2002; Able and Fahay, 2010). The majority of adult Tautog from the northern part of the population make seasonal migrations from the estuary in fall to areas approximately 3 km from shore with rugged terrain (Munroe, 2002). During cold winters, Tautog are sluggish, may hibernate in shelters, and are susceptible to cold-shock mortality events (Munroe, 2002; ASMFC, 2015). In spring, the fish return to the estuary to spawn and feed (Munroe, 2002). There is very little along-shelf movement during these migrations (Munroe, 2002). Many larger fishes and piscivorous birds consume Tautog, including Spiny Dogfish, skates, Red Hake, Monkfish (Goosefish), Striped Bass, Sea Raven, and cormorants (Munroe, 2002; Able and Fahay, 2010). The Atlantic States Marine Fisheries Commission manages the species as three stocks. As of 2013, the Southern New England stock is overfished and experiencing overfishing; the Connecticut-New York-New Jersey and the Delaware-Maryland-Virginia stocks are overfished, but not experiencing overfishing (ASMFC, 2015).

Literature Cited:

Able KW, Fahay MP. Ecology of estuarine fishes: temperate waters of the western North Atlantic. Baltimore: The Johns Hopkins University Press; 2010. 566p.

Arendt MD, Lucy JA, Evans DA. Diel and seasonal activity patterns of adult tautog, Tautoga onitis, in lower Chesapeake Bay, inferred from ultrasonic telemetry. Environ Biol Fishes. 2001; 62(4): 379-391. doi: 10.1023/A:1012266214143

Atlantic States Marine Fisheries Commission (ASMFC). 2015. Tautog stock assessment for peer review. Accessed online (April 2015): http://www.asmfc.org/species/tautog

Collie JS, Wood AD, Jeffries HP. Long-term shifts in the species composition of a coastal fish community. Can J Fish Aquat Sci. 2008; 65(7): 1352-1365. doi: 10.1139/F08-048

Gauthier DA, Lincoln JA, Turner JT. Distribution, abundance, and growth of larval tautog, Tautoga onitis, in Buzzards Bay, Massachusetts, USA. Mar Ecol. 2008; 29(1): 76-88. doi: 10.1111/j.1439-0485.2007.00212.x

Howell P, Auster PJ. Phase shift in an estuarine finfish community associated with warming temperatures. Mar Coast Fish. 2012; 4(1): 481-495. doi: 10.1080/19425120.2012.685144

Munroe, TA. Tautog/ Tautoga onitus (Linnaeus 1758). In: Collete BB, Klein-MacPhee G, editors, Fishes of the Gulf of Maine, 3rd edition. Washington: Smithsonian Institution Press; 2002. pp. 449-457.

Olla BL, Studholme AL, Bejda AJ, Samet C. Role of temperature in triggering migratory behavior of the adult tautog Tautoga onitis under laboratory conditions. Mar Biol. 1980; 59(1): 23-30. doi: 10.1007/BF00396979

Steimle FW, Shaheen PA. (1999). Tautog (Tautoga onitis) Life History and Habitat Requirements. US Dep Commer. NOAA Tech Memo NMFS NE, 118, 23. Available: http://www.nefsc.noaa.gov/publications/tm/tm118/tm118.pdf

Return to Table of Contents

Thorny Skate – *Amblyraja radiata*

Overall Vulnerability Rank = High

Biological Sensitivity = High
Climate Exposure = High
Data Quality = 88% of scores ≥ 2

Amblyraja radiata	Expert Scores	Data Quality	Expert Scores Plots (Portion by Category)
Stock Status	3.8	2.8	
Other Stressors	1.5	1.8	
Population Growth Rate	3.6	2.6	
Spawning Cycle	1.4	2.8	
Complexity in Reproduction	1.5	2.0	
Early Life History Requirements	1.0	3.0	
Sensitivity to Ocean Acidification	1.4	3.0	
Prey Specialization	1.2	3.0	
Habitat Specialization	1.2	3.0	
Sensitivity to Temperature	2.2	2.8	
Adult Mobility	1.8	2.4	
Dispersal & Early Life History	1.7	2.8	
Sensitivity Score	High		
Sea Surface Temperature	3.9	3.0	
Variability in Sea Surface Temperature	1.0	3.0	
Salinity	1.2	3.0	
Variability Salinity	1.2	3.0	
Air Temperature	1.0	3.0	
Variability Air Temperature	1.0	3.0	
Precipitation	1.0	3.0	
Variability in Precipitation	1.0	3.0	
Ocean Acidification	4.0	2.0	
Variability in Ocean Acidification	1.0	2.2	
Currents	2.1	1.0	
Sea Level Rise	1.1	1.5	
Exposure Score	High		
Overall Vulnerability Rank	High		

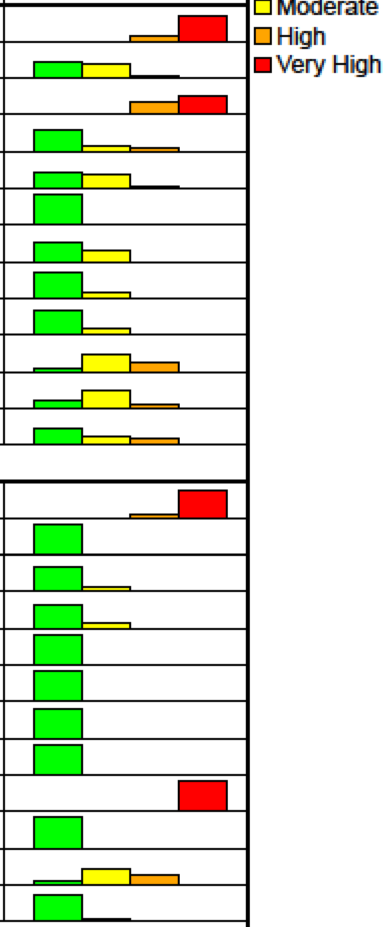

Return to Table of Contents

Thorny Skate (*Amblyraja radiata*)

Overall Climate Vulnerability Rank: **High** (100% certainty from bootstrap analysis).

Climate Exposure: **High**. Two exposure factors contributed to this score: Ocean Surface Temperature (3.9) and Ocean Acidification (4.0). Thorny Skate are demersal and complete their life cycle in marine habitats.

Biological Sensitivity: **High**. Two attributes scored above 3.0: Stock Status (3.8) and Population Growth Rate (3.6). The Thorny Skate index is below the biomass target and biomass threshold and has been declining since the 1960s (NEFSC 2013). In general, skates have a low population growth rate (higher sensitivity to climate change) (Frisk 2010).

Distributional Vulnerability Rank: **High** (100% certainty from bootstrap analysis). Thorny Skate are habitat generalists and moderately mobile as adults. In addition, skate egg cases are subject to movement by currents and juveniles may move on scales of 1-10 km's.

Directional Effect in the Northeast U.S. Shelf: The effect of climate change on Thorny Skate is very likely to be negative (>95% certainty in expert scores). Thorny Skate is a cold-water species that has shown declines in abundance in recent years. Warming and acidification may reduce productivity further.

Data Quality: 88% of the data quality scores were 2 or greater indicate that data quality is moderate.

Climate Effects on Abundance and Distribution: Little specific information exists on the effect of climate on Thorny Skate. Di Santo (2015) found that increased warming and acidification reduce body condition of newly hatched Little Skate. These reductions in size could result in reduced juvenile survival and thus recruitment if similar effects occur in Thorny Skate. Nye et al. (2009) found a small shift southward accompanied by a shift into deeper water. Swain and Benoit (2006) documented a shift into deeper water in the Gulf of Saint Lawrence and argued that this resulted from density-dependent habitat selection not a climatic shift in preferred habitat.

Life History Synopsis**:** Thorny Skate is a boreal to arctic, large-bodied skate species found on both sides of the Atlantic Ocean. In the western North Atlantic, Thorny Skate occurs from western Greenland to South Carolina, but is uncommon south of Georges Bank (Packer et al., 2003). Thorny Skate is a late maturing species that begins to mature between 5-11 years and mates using internal fertilization (Packer et al., 2003; Sulikowski et al., 2006). Eggs are encapsulated singly in a rectangular, horned egg case (Packer et al., 2003). Females with fully formed egg capsules are found year round, but the proportion of females with eggs is highest during summer (Packer et al., 2003). Later maturing females produce larger eggs (Packer et al., 2003). Embryonic development in the egg case may take 2-3 years in cold water (Packer et al., 2003). Halibut, Monkfish (Goosefish), Greeland Sharks, and predatory gastropods eat embryonic skates (Packer et al., 2003). After hatching, the juvenile skate consumes yolk for 2-4 months before beginning active feeding (Packer et al., 2003). Juveniles are common in the Gulf of Maine and northern Georges Bank over sand, gravel, broken shell, pebbles, or soft mud of the mid- and outer-shelf, in areas with 4-9°C bottom water (Packer et al., 2003). In the southern part of their range, Thorny Skate are only found on the continental slope (McEachran, 2002). Adults occur in similar conditions, but tend not to occur as shallow as younger skates (Packer et al., 2003). Thorny Skate may make small seasonal movements, but in tagging studies, are rarely collected far from the tagging site, and appear to be nearly sedentary (McEachran, 2002; Packer et al., 2003). Thorny Skate prey

opportunistically on the most abundant benthic prey species in the area (Packer et al., 2003). Polychaetes, decapods, and cephalopods are major prey species, but fishes are occasionally dominant (Packer et al., 2003). An ontogenetic shift in the importance of prey from crustaceans to polychaetes to fish and squid has been observed, but regional differences in prey abundance complicate the pattern (Packer et al., 2003). Seals, sharks, and Halibut are common predators of the species (Packer et al., 2003). The New England Fishery Management Council manages Thorny Skate as part of a skate complex. Steady declines in abundance since the 1970s led the Council to prohibit its possession since 2003 (NEFSC, 2007). In the most recent assessment update, Thorny Skate was overfished, and also experiencing overfishing (NEFSC, 2014).

Literature Cited:

Di Santo V. Ocean acidification exacerbates the impacts of global warming on embryonic little skate, *Leucoraja erinacea* (Mitchill). J Exp Mar Bio Ecol. 2015; 463: 72-78. doi:10.1016/j.jembe.2014.11.006

McEachran JD. Thorny Skate/ Amblyraja radiata. In: B.B. Collette BB, Klein-MacPhee G, editors, Fishes of the Gulf of Maine, 3rd ed. Washington: Smithsonian Institution Press; 2002. p. 62-64.

Packer DB, Zetlin CA, Vitaliano JJ. 2003. Essential fish habitat source document: Thorny skate, Amblyraja radiata, life history and habitat characteristics. NOAA Tech Memo NMFS NE 178; 39 p. Accessed online (August 2015): http://www.nefsc.noaa.gov/nefsc/publications/tm/tm178/

Northeast Fisheries Science Center (NEFSC). 2007. 44th Northeast Regional Stock Assessment Workshop (44th SAW): 44th SAW assessment report. US Dep Commer, Northeast Fish Sci Cent Ref Doc 07-10; 661 p. Accessed online (August 2015): http://www.nefsc.noaa.gov/publications/crd/crd0710/

Northeast Fisheries Science Center (NEFSC). 2014. Update of Skate Stock Status Based on NEFSC Bottom Trawl Survey Data through Autumn 2013/Spring 2014. Available: http://s3.amazonaws.com/nefmc.org/2_NEFSC_UpdateSkateStocks2014.pdf

Sulikowski J A, Kneebone J, Elzey S, Jurek J, Howell WH, Tsang PCW. Using the composite variables of reproductive morphology, histology and steroid hormones to determine age and size at sexual maturity for the thorny skate Amblyraja radiata in the western Gulf of Maine. J Fish Biol. 2006; 69(5): 1449-1465. doi: 10.1111/j.1095-8649.2006.01207.x

Swain DP, Benoit HP. Change in habitat associations and geographic distribution of thorny skate (Amblyraja radiata) in the southern Gulf of St Lawrence: density-dependent habitat selection or response to environmental change? Fish Oceanogr. 2006; 15(2): 166-182. doi: 10.1111/j.1365-2419.2006.00357.x

Nye JA, Link JS, Hare JA, Overholtz WJ. Changing spatial distribution of fish stocks in relation to climate and population size on the Northeast United States continental shelf. Mar Ecol Prog Ser. 2009; 393: 111-129. doi: 10.3354/meps08220

Return to Table of Contents

Tilefish – *Lopholatilus chamaeleonticeps*

Overall Vulnerability Rank = High

Biological Sensitivity = High
Climate Exposure = High
Data Quality = 75% of scores ≥ 2

Lopholatilus chamaeleonticeps	Expert Scores	Data Quality	Expert Scores Plots (Portion by Category)
Stock Status	2.2	2.8	
Other Stressors	2.1	2.6	
Population Growth Rate	3.8	2.6	
Spawning Cycle	2.1	2.8	
Complexity in Reproduction	2.3	0.8	
Early Life History Requirements	2.0	0.8	
Sensitivity to Ocean Acidification	1.4	1.8	
Prey Specialization	1.2	3.0	
Habitat Specialization	2.9	3.0	
Sensitivity to Temperature	2.1	2.6	
Adult Mobility	3.1	2.4	
Dispersal & Early Life History	2.0	1.4	
Sensitivity Score	High		
Sea Surface Temperature	3.9	3.0	
Variability in Sea Surface Temperature	1.0	3.0	
Salinity	2.2	3.0	
Variability Salinity	1.2	3.0	
Air Temperature	1.0	3.0	
Variability Air Temperature	1.0	3.0	
Precipitation	1.0	3.0	
Variability in Precipitation	1.0	3.0	
Ocean Acidification	4.0	2.0	
Variability in Ocean Acidification	1.0	2.2	
Currents	2.1	1.0	
Sea Level Rise	1.1	1.5	
Exposure Score	High		
Overall Vulnerability Rank	High		

Low
Moderate
High
Very High

Tilefish (*Lopholatilus chamaeleonticeps*)

Overall Climate Vulnerability Rank: **High** (100% certainty from bootstrap analysis).

Climate Exposure: **High**. Two exposure factors contributed to this score: Ocean Surface Temperature (3.9) and Ocean Acidification (4.0). All life stages of Tilefish use marine habitats.

Biological Sensitivity: **High.** Two factors scored above 3.0: Population Growth Rate (3.8) and Adult Mobility (3.1). Tilefish are slow growing and long-lived (Lombardi-Carlson and Andrew 2015). In addition, Tilefish use burrows for shelter and are relatively site specific (Able et al. 1982).

Distributional Vulnerability Rank: **Low** (100% certainty from bootstrap analysis). Tilefish is site specific and inhabit specialized habitats. They do have planktonic larvae, but this stage is very rare in regional ichthyoplankton sampling suggesting limited dispersal (Steimle et al., 1999).

Directional Effect in the Northeast U.S. Shelf: The effect of climate change on Tilefish on the Northeast U.S. Shelf is estimated to be neutral, but with a moderate degree of uncertainty (66-90% certainty in expert scores). Tilefish is a warm-water fish and warming in the Northeast U.S. may result in more available habitat. However, Tilefish has high habitat specificity and the effect of warming on habitat availability in uncertain. In addition, the effect of climate on productivity is unclear.

Data Quality: 75% of the data quality scores were 2 or greater indicate that data quality is moderate.

Climate Effects on Abundance and Distribution: A Tilefish die-off in 1882 was attributed to enhanced southward transport of cold water in the Labrador Current, coincident with a minimum in the North Atlantic Oscillation (NAO) index during the early 1880s (Marsh et al., 1999). Fisher et al. (2014) hypothesized that NAO affected commercial landings throughout most of the 20th century by altering slope water temperatures and likely the Tilefish's reproductive success; warmer water increases productivity of Tilefish, while colder water decreases productivity. However, Fisher et al. (2014) proposed that the environment-landings relationship broke down as exploitation increased.

Life History Synopsis**:** Tilefish is a large, long-lived, demersal, warm-water species of fish that occurs from Nova Scotia to Suriname, but not in the Caribbean Sea, and is primarily found from the southern edge of Georges Bank to Key West, Florida, and throughout the Gulf of Mexico (Steimle, 1999). Tilefish reach maturity at 5-11 years, with males slower to mature than females (Able, 2002). Spawning occurs from March to November with a peak in May to September (Steimle, 1999). Little is known about spawning behavior, but Tilefish are highly fecund, may be pair spawners, and are likely fractional or serial spawners (Steimle, 1999; Able, 2002). Eggs are buoyant and hatch after at least 40 hours based on captive eggs held at warmer temperatures than experienced in the wild (Steimle, 1999). Larvae are planktonic from July to September in warm waters over the outer continental shelf (Steimle, 1999). Larvae are probably zooplanktivorous (Steimle, 1999). Settlement patterns are unknown, but juveniles have been found in vertical shaft burrows in semi-lithified clay and in anthropogenic structures like lobster traps and ship wrecks (Steimle, 1999). Juveniles may not be able to excavate their own burrows initially, so may use the burrows of other animals to start their own (Steimle, 1999). Juveniles consume benthic organisms such as crabs, Conger Eels, Hagfish, bivalve molluscs, polychaetes, holothurians, sea anemones, and echinoderms, but occasionally consume pelagic prey such as salps, squid, and small fish (Steimle, 1999). Adults and juveniles have been found from depths of 80-540 m, but mostly occur in a narrow band of the outer continental shelf and upper slope (100-200 m) where water temperatures stay

fairly stable (8-17°C) known as the warm belt (Steimle, 1999). Small migrations or hibernation during low temperature events are possible, but a mass mortality event in 1882 is attributed to a flood of cold water into the area (Steimle, 1999; Able, 2002). Tilefish require structure such as boulders, the scour depressions beneath them, or more commonly, vertical or horizontal burrows in semi-lithified clay that they likely inhabit and continue to excavate throughout their adult life, never migrating far (Steimle, 1999). Tilefish stay close to their burrows at night, but forage on a variety of benthic prey during the day including: shrimps, crabs, molluscs, polychaetes, sea cucumbers, brittlestars, urchins, anemones, tunicates, and occasionally fishes (Able, 2002). Molluscs and echinoderms dominate the diet of smaller fish, but their diet shifts to galatheids, spider crabs, and ophiuroids in larger fish (Able, 2002). Tilefish burrows are also subcolonized by decapod crustaceans, and for all inhabitants, are likely a way to avoid predators (Steimle, 1999; Able, 2002). Monkfish (Goosefish), Spiny Dogfish, Conger Eels, and larger Tilefish prey on juveniles while sharks are suspected to prey on adults (Steimle, 1999). The Mid Atlantic Fishery Management Council manages Tilefish through the Golden Tilefish Fishery management plan. The Mid-Atlantic stock is considered rebuilt and is not overfished or undergoing overfishing, based on the most recent assessment (NEFSC, 2014). The southeast United States and Gulf of Mexico stocks are also not overfished nor undergoing overfishing (SEDAR, 2011a, b).

Literature Cited:

Able KW. Tilefish/ *Lopholatilus chamaeleonticeps* Goode and Bean 1879. Pages 397-400. In: BB Collette, G Klein-MacPhee (editors), Fishes of the Gulf of Maine, 3rd edition. Smithsonian Institution Press, Washington D.C. 2002. 882 p.

Able KW, Grimes CB, Cooper RA, Uzmann JR. Burrow construction and behavior of Tilefish, Lopholatilus chamaeleonticeps, in Hudson Submarine Canyon. Environ Biol Fishes. 1982; 7(3): 199-205. DOI: 10.1007/BF00002496

Fisher JA, Frank KT, Petrie B, Leggett WC. Life on the edge: environmental determinants of Tilefish (*Lopholatilus chamaeleonticeps*) abundance since its virtual extinction in 1882. ICES J Mar Sci. 2014; 71(9): 2371-2378. DOI: 10.1093/icesjms/fsu053

Lombardi-Carlson LA, Andrews AH. Age estimation and lead-radium dating of golden Tilefish, *Lopholatilus chamaeleonticeps*. Environ Biol Fishes. 2015; 1-15. DOI: 10.1007/s10641-015-0398-0

Marsh R, Petrie B, Weidman CR, Dickson RR, Loder JW, Hannah CG, et al. The 1882 Tilefish kill—a cold event in shelf waters off the north-eastern United States? Fisher Oceanogr. 1999; 8(1): 39-49. DOI: DOI: 10.1046/j.1365-2419.1999.00092.x

Northeast Fisheries Science Center (NEFSC). 58th Northeast Regional Stock Assessment Workshop (58th SAW) Assessment Report. US Dept Commer, Northeast Fish Sci Cent Ref Doc. 2014; 14-04: 784 p. Accessed online (August 2015): http://www.nefsc.noaa.gov/publications/crd/crd1404/

Southeast Data, Assessment and Review (SEDAR). SEDAR 25 Stock Assessment Report: Aouth Atlantic Tilefish. 2011. Accessed online (August 2015): http://sedarweb.org/sedar-25-stock-assessment-report-south-atlantic-Tilefish

Southeast Data, Assessment and Review (SEDAR). SEDAR 22 Stock Assessment Report: Gulf of Mexico Tilefish. 2011. Accessed online (August 2015): http://sedarweb.org/sedar-22-final-stock-assessment-report-gulf-mexico-Tilefish

Steimle FW, Zetline CA, Berrien PL, Johnson DL, Chang S. Essential fish habitat source document: Tilefish, Lopholatilus chamaeleonticeps, life history and habitat characteristics. NOAA Tech Memo NMFS NE 2999; 152: 30 p. Accessed online (August 2015): http://www.nefsc.noaa.gov/nefsc/publications/tm/tm152/

[Return to Table of Contents]

Weakfish – *Cynoscion regalis*

Overall Vulnerability Rank = Moderate

Biological Sensitivity = Low
Climate Exposure = Very High
Data Quality = 88% of scores ≥ 2

Cynoscion regalis	Expert Scores	Data Quality	Expert Scores Plots (Portion by Category)
Stock Status	2.4	1.4	
Other Stressors	2.5	2.6	
Population Growth Rate	1.8	2.6	
Spawning Cycle	2.2	3.0	
Complexity in Reproduction	2.1	2.6	
Early Life History Requirements	2.4	2.5	
Sensitivity to Ocean Acidification	1.7	3.0	
Prey Specialization	1.2	3.0	
Habitat Specialization	1.6	3.0	
Sensitivity to Temperature	1.9	2.6	
Adult Mobility	1.5	2.7	
Dispersal & Early Life History	2.6	3.0	
Sensitivity Score	Low		
Sea Surface Temperature	4.0	3.0	
Variability in Sea Surface Temperature	1.0	3.0	
Salinity	2.8	3.0	
Variability Salinity	1.2	3.0	
Air Temperature	4.0	3.0	
Variability Air Temperature	1.0	3.0	
Precipitation	1.2	3.0	
Variability in Precipitation	1.3	3.0	
Ocean Acidification	4.0	2.0	
Variability in Ocean Acidification	1.0	2.2	
Currents	2.0	1.0	
Sea Level Rise	2.5	1.5	
Exposure Score	Very High		
Overall Vulnerability Rank	Moderate		

Low
Moderate
High
Very High

Weakfish (*Cynoscion regalis*)

Overall Climate Vulnerability Rank: **Moderate** (96% certainty from bootstrap analysis).

Climate Exposure: **Very High**. Three exposure factors contributed to this score: Ocean Surface Temperature (4.0), Ocean Acidification (4.0) and Air Temperature (4.0). Exposure to all three factors occur during the life stages. Weakfish occur in estuarine and coastal waters and make seasonal north-south migrations.

Biological Sensitivity: **Low**. Two sensitivity attributes scored above 2.5: Other Stressors (2.5) and Dispersal & Early Life History (2.6). Deterioration of coastal waters due to urbanization, dredging, and other human activities has contributed to a decline in Weakfish abundance in some areas. In addition, Weakfish are facultative estuarine dependent and use estuarine and coastal areas as feeding and nursery grounds (Able, 2005).

Distributional Vulnerability Rank: **High** (100% certainty from bootstrap analysis). Two attributes indicated vulnerability to distribution shift: Adult Mobility and Habitat Sensitivity. Weakfish are mobile and make seasonal north-south migrations. Adults use a variety of nearshore and coastal habitats during warmer months and inner shelf habitats in colder months.

Directional Effect in the Northeast U.S. Shelf: The effect of climate change on Weakfish on the Northeast U.S. Shelf is estimated to be neutral, but with a moderate degree of uncertainty (<66% certainty in expert scores). Adult distribution may extend northwards as warming continues, but losses of habitat due to sea-level rise may negatively impact productivity. The effect of ocean acidification over the next 30 years is likely to be minimal. Despite high data quality, there are a number of information gaps in the region, which contribute to the uncertainty as to the directional effect of climate change.

Data Quality: 88% of the data quality scores were 2 or greater indicate that data quality is moderate.

Climate Effects on Abundance and Distribution: Relatively little work has been done on the effect of climate on distribution and productivity of Weakfish. Lankford and Targett (1994) found an interactive effect of salinity and temperature on juvenile Weakfish growth suggesting changes in temperature, precipitation, and sea level could affect productivity. In terms of distribution, the abundance of Weakfish at the northern end of their range has increased, potentially suggesting a northward shift (Howell and Auster, 2012).

Life History Synopsis**:** Weakfish is an estuarine and coastal fish species that can be found from Nova Scotia to Cape Canaveral, Florida, but is most abundant between New York and North Carolina (Able and Fahay, 2010). Maturity is reached around age 1, but older fish arrive at the spawning grounds first, produce more eggs per batch, and more batches per season, so the contribution of age-1 spawners is believed to be proportionally low (Klein-MacPhee, 2002; NEFSC, 2009). Spawning occurs May – August, beginning in the south, and in near-shore marine areas, particularly near larger inlets, and in bays and estuaries (Klein-MacPhee, 2002; Able and Fahay, 2010). Weakfish return to the same spawning grounds each year (Able and Fahay, 2010). Eggs are pelagic, occur in warm water, but a range of salinities, and hatch after 2-3 days (Klein-MacPhee, 2002; Able and Fahay 2010). Eggs and larvae occur in both marine-coastal and estuarine habitats, but it is not fully known whether both are equally suitable nursery habitats (Able and Fahay, 2010). Larvae are pelagic, but become demersal early, and may use bottom currents to move into low-salinity nursery habitats (Klein-MacPhee, 2002). Larvae consume copepods,

tintinnids, and polychaete larvae (Klein-MacPhee, 2002). Larvae settle to a variety of substrates in the intertidal and subtidal marshes, as well as shallow and deep bay areas (Able and Fahay, 2010). Larger juveniles and adults prefer the deep bay areas in summer (Able and Fahay, 2010). Juveniles leave the estuaries in fall, and migrate to the outer-shelf south of Cape Hatteras, North Carolina (Able and Fahay, 2010). The diet of juveniles shifts from primarily invertebrates, especially mysid and decapod shrimp, to primarily fish, especially clupeids and engraulids (Klein-MacPhee, 2002; Able and Fahay, 2010). Adult Weakfish occur in shallow coastal waters, bays, and estuaries over sand or mud substrates, and occasionally move into freshwater (Able and Fahay, 2010). In the southern portion of their range, Weakfish are resident and do not make offshore migrations (Klein-MacPhee, 2002). In fall, the northern portion of the population moves in schools to overwintering grounds on the outer-shelf (Klein-MacPhee, 2002). Larger Weakfish do not migrate as far south as younger fish and overwinter offshore of Southern New England and the Mid-Atlantic states (Klein-MacPhee, 2002; Able and Fahay, 2010). By spring, adults begin returning to coastal spawning habitats, starting with the larger fish (Klein-MacPhee, 2002). Weakfish adults are generally piscivorous, but include a variety of invertebrate prey in their diet, and while their diet is largely opportunistic, clupeids, anchovies, and small crustaceans are the dominant prey (Klein-MacPhee, 2002; Able and Fahay, 2010). The major predators of Weakfish include: Striped Bass, Bluefish, and older Weakfish, but dogfish, skates, Angel Shark, Monkfish (Goosefish), and Summer Flounder are also known predators of the species (Klein-MacPhee, 2002; Able and Fahay, 2010). The Atlantic States Marine Fisheries Commission manages Weakfish through the Interstate Fishery Management Plan for Weakfish, but a stock-wide assessment is conducted by the Northeast Fisheries Science Center (NEFSC, 2009). Weakfish are deemed depleted, but not experiencing overfishing (ASMFC, 2009). Total mortality is increasing despite steady fishing mortality, so recovery is not expected through management of the fishery alone (NEFSC, 2009; ASMFC, 2009).

Literature Cited:

Able KW, Fahay MP. Ecology of estuarine fishes: temperate waters of the western North Atlantic. Baltimore: The Johns Hopkins University Press; 2010. 566p.

Atlantic States Marine Fisheries Commission (ASMFC). 2009. Addendum IV to Amendment 4 to the Weakfish Fishery Management Plan. Accessed online (April 2015): http://www.asmfc.org/species/weakfish

Howell P, Auster PJ. Phase shift in an estuarine finfish community associated with warming temperatures. Mar Coast Fish. 2012; 4(1): 481-495. doi: 10.1080/19425120.2012.685144

Klein-MacPhee G. Weakfish/ Cynoscion regalis (Bloch and Schneider 1801). In: Collete BB, Klein-MacPhee G, editors, Fishes of the Gulf of Maine, 3rd edition. Washington: Smithsonian Institution Press; 2002. pp. 436-440.

Lankford Jr TE, Targett TE. Suitability of estuarine nursery zones for juvenile weakfish (Cynoscion regalis): effects of temperature and salinity on feeding, growth and survival. Mar Biol. 1994; 119(4): 611-620. doi: 10.1007/BF00354325

Northeast Fisheries Science Center. 2009. 48th Northeast Regional Stock Assessment Workshop (48th SAW) Assessment Report. US Dept Commer, Northeast Fish Sci Cent Ref Doc. 09-15; 834 p. Accessed Online (April 2015): http://www.nefsc.noaa.gov/publications/crd/crd0915/

Return to Table of Contents

White Hake – *Urophycis tenuis*

Overall Vulnerability Rank = Moderate

Biological Sensitivity = Moderate
Climate Exposure = High
Data Quality = 79% of scores ≥ 2

Urophycis tenuis	Expert Scores	Data Quality	Expert Scores Plots (Portion by Category)
Stock Status	2.6	2.8	
Other Stressors	1.9	0.4	
Population Growth Rate	2.6	2.7	
Spawning Cycle	2.7	2.5	
Complexity in Reproduction	1.5	1.4	
Early Life History Requirements	2.1	1.4	
Sensitivity to Ocean Acidification	1.2	2.4	
Prey Specialization	1.2	3.0	
Habitat Specialization	1.2	3.0	
Sensitivity to Temperature	2.0	2.8	
Adult Mobility	1.4	2.7	
Dispersal & Early Life History	2.4	2.0	
Sensitivity Score	Moderate		
Sea Surface Temperature	3.9	3.0	
Variability in Sea Surface Temperature	1.0	3.0	
Salinity	1.1	3.0	
Variability Salinity	1.2	3.0	
Air Temperature	1.0	3.0	
Variability Air Temperature	1.0	3.0	
Precipitation	1.0	3.0	
Variability in Precipitation	1.0	3.0	
Ocean Acidification	4.0	2.0	
Variability in Ocean Acidification	1.0	2.2	
Currents	2.1	1.0	
Sea Level Rise	1.2	1.5	
Exposure Score	High		
Overall Vulnerability Rank	Moderate		

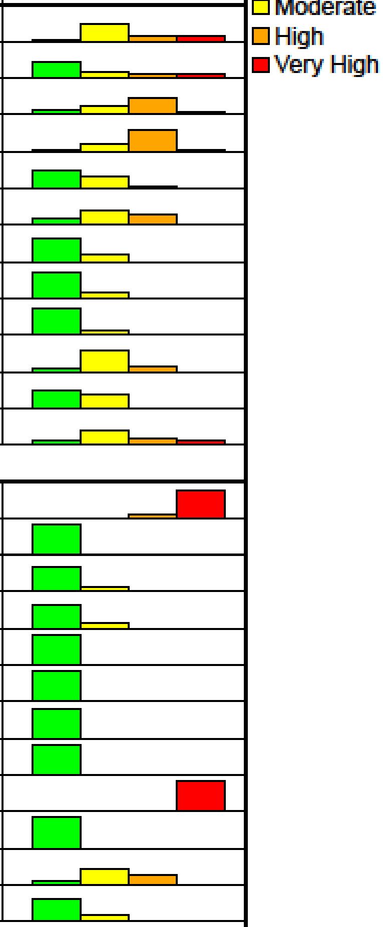

Return to Table of Contents

White Hake (*Urophycis tenuis*)

Overall Climate Vulnerability Rank: **Moderate** (90% certainty from bootstrap analysis).

Climate Exposure: **High**. Two exposure factors contributed to this score: Ocean Surface Temperature (3.9) and Ocean Acidification (4.0). All life stages of White Hake use marine habitats.

Biological Sensitivity: **Moderate**. Three sensitivity attributes scored above 2.5: Stock Status (2.6), Population Growth Rate (2.6), and Spawning Cycle (2.7). White Hake are not overfished nor is overfishing occurring, but the species is relatively rare in the Northeast U.S. Shelf. Growth of adults is relatively slow and spawning occurs in a relatively narrow time span (early spring) in deep water (Chang et al., 1999).

Distributional Vulnerability Rank: **High** (100% certainty from bootstrap analysis).

Directional Effect in the Northeast U.S. Shelf: The effect of climate change on White Hake on the Northeast U.S. Shelf is likely to be negative (90-95% certainty in expert scores). White Hake is a cold-temperate species and warming could cause distribution shifts out of the region.

Data Quality: 79% of the data quality scores were 2 or greater indicate that data quality is moderate.

Climate Effects on Abundance and Distribution: There is a lack of information regarding the potential effects of climate on White Hake productivity. In terms of distribution, Nye et al. (2009) found that White Hake distribution has shifted northwards and into deeper waters over time.

Life History Synopsis**:** White Hake is an estuarine and marine, demersal species found from Newfoundland to North Carolina (Klein-MacPhee, 2002; NEFSC, 2013). The population is divided into two stocks: a Canadian stock primarily occurring in the Gulf of St. Lawrence and Scotian Shelf, and a United States stock primarily from the Gulf of Maine and Georges Bank (NEFMC, 2014). The northern stock moves inshore to spawn in August and September; the southern stock is believed to move offshore to the continental slope to spawn in early spring (April-May; Chang et al., 1999). However, timing and location of spawning is uncertain for the United States stock (Chang et al., 1999; NEFMC, 2014). Females are larger and longer-lived than males, but both are mature around 1.5 years (Chang et al., 1999). Egg and larval distributions are poorly understood for the United States stock (Chang et al., 1999; NEFMC, 2014). Eggs are pelagic, occurring across the shelf in a wide range of temperatures, and hatch within a week of spawning (Chang et al., 1999). Larvae are pelagic and probably occur in offshore waters along the continental slope off southern Georges Bank and the mid-Atlantic (Chang et al., 1999). Early juveniles are pelagic for approximately two months before settling to muddy and fine-grained, sandy bottom or eelgrass habitat on the shelf and in estuaries (Chang et al., 1999). Juveniles prefer warmer, less saline, more turbid waters and migrate inshore when waters are warm and offshore when cool (Chang et al., 1999). The diet of demersal juveniles includes polychaetes, shrimp, and other crustaceans (Chang et al., 1999). Atlantic puffin and Arctic tern are major predators of juvenile White Hake (Chang et al., 1999). Adult White Hake are demersal occurring from estuaries to the upper continental slope and deep basins on muddy and fine-grained, sandy bottom (Chang et al., 1999). Adults also move inshore during warmer months on Georges Bank and the Gulf of Maine (Chang et al., 1999). Fish, including young White Hake, squid, shrimp, and other crustaceans are the main prey of the species (Chang et al., 1999; Klein-MacPhee, 2002). Several species of fish including Sandbar Shark, larger White Hake, and Atlantic Cod as well as Atlantic puffin and arctic tern are major predators of hake

(Klein-MacPhee, 2002). The New England Fishery Management Council manages White Hake under the Northeast Multispecies Fisheries Management Plan (NEFMC, 2014). White Hake are neither overfished nor is overfishing occurring (NEFSC, 2013).

Literature Cited:

Chang S, Morse WW, Berrien PL. Essential Fish Habitat Source Document: White Hake, Urophycis tenuis, life history and habitat characteristics. NOAA Tech. Memo. 1999; NMFS-NE-136. 23p. Accessed online (August 2015): http://www.nefsc.noaa.gov/nefsc/publications/tm/tm136/

Klein-MacPhee G. White Hake/ *Urophycis tenuis* (Mitchill 1814). Pages 258-261. In: BB Collette and G Klein-MacPhee (editors), Fishes of the Gulf of Maine, 3rd edition. Smithsonian Institution Press, Washington D.C. 2002; 882 p.

New England Fisheries Management Council (NEFMC). Framework adjustment 51 to the Northeast Multispecies FMP. 2014; 318p. Accessed online (August 2015): http://archive.nefmc.org/nemulti/frame/fw%2051/Framework%20Adjustment%2051.pdf

Northeast Fisheries Science Center (NEFSC). 56th Northeast Regional Stock Assessment Workshop (56th SAW) Assessment Summary Report. Woods Hole (MA): U.S. Department of Commerce. Northeast Fisheries Science Center Ref. Doc. 2013; 13-04: 42 p. Accessed online (August 2015): http://nefsc.noaa.gov/publications/crd/crd1304/

Nye JA, Link JS, Hare JA, Overholtz WJ. Changing spatial distribution of fish stocks in relation to climate and population size on the Northeast United States continental shelf. Mar Ecol Prog Ser. 2009; 393: 111-129. DOI: 0.3354/meps08220

Return to Table of Contents

Windowpane – *Scophthalmus aquosus*

Overall Vulnerability Rank = Low

Biological Sensitivity = Low
Climate Exposure = High
Data Quality = 79% of scores ≥ 2

Scophthalmus aquosus	Expert Scores	Data Quality	Expert Scores Plots (Portion by Category)
Stock Status	2.6	2.8	
Other Stressors	2.4	2.6	
Population Growth Rate	1.7	2.6	
Spawning Cycle	1.3	2.8	
Complexity in Reproduction	2.0	2.4	
Early Life History Requirements	2.0	1.0	
Sensitivity to Ocean Acidification	1.4	1.8	
Prey Specialization	1.9	3.0	
Habitat Specialization	1.4	3.0	
Sensitivity to Temperature	1.6	3.0	
Adult Mobility	1.8	2.4	
Dispersal & Early Life History	2.5	1.8	
Sensitivity Score	Low		
Sea Surface Temperature	3.9	3.0	
Variability in Sea Surface Temperature	1.0	3.0	
Salinity	2.1	3.0	
Variability Salinity	1.2	3.0	
Air Temperature	1.0	3.0	
Variability Air Temperature	1.0	3.0	
Precipitation	1.0	3.0	
Variability in Precipitation	1.0	3.0	
Ocean Acidification	4.0	2.0	
Variability in Ocean Acidification	1.0	2.2	
Currents	2.1	1.0	
Sea Level Rise	1.2	1.5	
Exposure Score	High		
Overall Vulnerability Rank	Low		

Low
Moderate
High
Very High

Windowpane (*Scophthalmus aquosus*)

Overall Climate Vulnerability Rank: **Low** (62% certainty from bootstrap analysis).

Climate Exposure: **High**. Two exposure factors contributed to this score: Ocean Surface Temperature (3.9) and Ocean Acidification (4.0). All life stages of Windowpane use marine habitats.

Biological Sensitivity: **Low**. Only one sensitivity attribute scored above 2.5: Stock Status (2.6). One stock in the region is overfished and overfishing is occurring, while the other stock is rebuilt (NEFSC, 2012).

Distributional Vulnerability Rank: **High** (93% certainty from bootstrap analysis). Windowpane are habitat generalists, make seasonal migrations, and have dispersive early life history stages.

Directional Effect in the Northeast U.S. Shelf: The effect of climate change on Windowpane is likely to be neutral (90-95% certainty in expert scores). Windowpane inhabits temperate waters and may benefit from warming on the Northeast U.S. Shelf. However, the abundance of Windowpane in some estuaries has decreased.

Data Quality: 79% of the data quality scores were 2 or greater indicate that data quality is moderate.

Climate Effects on Abundance and Distribution: Working in New York/New Jersey Harbor, Wilbur et al. (2015) found that Windowpane age-1 abundances were significantly reduced after several cold winters. This results suggests that Windowpane productivity may remain the same or increase with warming. Nye et al. (2009) found that Windowpane distribution has shifted northward in recent years. In Narragansett Bay and Long Island Sound, the abundance of Windowpane has decreased some (Collie et al., 2008; Howell and Auster, 2012). Thus current understanding is equivocal as to the effect of climate on Widowpane.

Life History Synopsis**:** Windowpane flounder is a shallow-water, estuarine and marine, fast-growing flatfish species found in the western Atlantic from the Gulf of St. Lawrence to Florida (Chang et al., 1999). Windowpane reach sexual maturity after 3-4 years (Chang et al., 1999). Spawning occurs from February to November, beginning in the south and expanding north (Chang et al., 1999; Klein-MacPhee, 2002). Peak spawning occurs in cool or mid-range temperature water in spring and fall (Chang et al., 1999). Eggs are pelagic and hatch in about a week (Chang et al., 1999). Larvae are pelagic and eat copepods and other zooplankton (Chang et al., 1999). Many planktivorous fish species eat egg and larval Windowpane, including conspecifics (Chang et al., 1999). Larvae settle at 10-20 mm total length in estuaries (spring-spawned larvae) and on the shelf (spring- and fall-spawned larvae; Chang et al., 1999). Juvenile Windowpane occur on the shelf year round (Chang et al., 1999). Juveniles and adults migrate seasonally from shallow or inshore water in spring - fall, to offshore or deeper water in winter, but are generally found on sand or mud substrates in mid-temperature water of <100 m depth (Chang et al., 1999). Adult Windowpane also make long distance migrations along shore; but their range is limited by high and low temperature waters (Chang et al., 1999; Klein-MacPhee, 2002). Juvenile and adult fish feed on small crustaceans (mostly mysids and decapods), polychaetes, and several small fish including smaller individuals of the species (Change et al., 1999; Klein-MacPhee, 2002). Several species of piscivorous fish, sharks, and skates consume Windowpane, but the main predators are Spiny Dogfish and Atlantic Cod (Chang et al., 1999; Klein-MacPhee, 2002). The species is managed by the New England Fishery Management Council's Northeast Multispecies Fishery Management Plan as two stocks: Gulf of Maine-Georges Bank and southern New England-middle Atlantic (NEFSC, 2012). Based on recent assessment

using data through the 2010, the northern stock was overfished and overfishing was occurring (NEFSC, 2012). In the southern stock overfishing is not occuring and was rebuilt based on the 2012 stock assessment (NEFSC, 2012).

Literature Cited:

Chang S, Berrien PL, Johnson DL, Morse WW. Essential Fish Habitat Source Document: Windowpane, *Scophthalmus aquosus*, life history and habitat characteristics. NOAA Tech. Memo. 1999; NMFS-NE-137: 32p. Accessed online (August 2015): http://www.nefsc.noaa.gov/nefsc/publications/tm/tm137/tm137.pdf

Klein-MacPhee G. Windowpane/ *Scopthalmus aquosus* (Mitchill, 1815). Pages 548-550. In: BB Collette, G Klein-MacPhee (editors), Fishes of the Gulf of Maine, 3rd edition. Smithsonian Institution Press, Washington D.C. 2002; 882 p.

Murawski SA. Climate change and marine fish distributions: forecasting from historical analogy. Trans Amer Fish Soc. 1993; 122(5): 647-658. DOI: 0.1577/1548-8659(1993)122<0647:CCAMFD>2.3.CO;2

Northeast Fisheries Science Center. Assessment or Data Updates of 13 Northeast Groundfish Stocks through 2010. US Dept Commer, Northeast Fish Sci Cent Ref Doc. 2012; 12-06: 789 p. Accessed online (August 2015): http://nefsc.noaa.gov/publications/crd/crd1206/

Nye JA, Link JS, Hare JA, Overholtz WJ. Changing spatial distribution of fish stocks in relation to climate and population size on the Northeast United States continental shelf. Mar Ecol Prog Ser. 2009; 393: 111-129. DOI: 0.3354/meps08220

Wilber DH, Clarke DG, Alcoba CM, Gallo J. Windowpane flounder (*Scophthalmus aquosus*) and winter flounder (*Pseudopleuronectes americanus*) responses to cold temperature extremes in a Northwest Atlantic estuary. J Sea Res. 2015; DOI: 10.1016/j.seares.2015.04.005

Return to Table of Contents

Winter Flounder – *Pseudopleuronectes americanus*

Overall Vulnerability Rank = Very High

Biological Sensitivity = High
Climate Exposure = Very High
Data Quality = 92% of scores ≥ 2

Pseudopleuronectes americanus	Expert Scores	Data Quality	Expert Scores Plots (Portion by Category)
Stock Status	3.4	3.0	
Other Stressors	2.4	2.6	
Population Growth Rate	2.2	2.7	
Spawning Cycle	2.6	2.7	
Complexity in Reproduction	2.4	2.8	
Early Life History Requirements	3.2	2.9	
Sensitivity to Ocean Acidification	1.4	2.4	
Prey Specialization	1.3	3.0	
Habitat Specialization	1.8	3.0	
Sensitivity to Temperature	2.1	3.0	
Adult Mobility	1.9	2.9	
Dispersal & Early Life History	2.7	2.8	
Sensitivity Score	High		
Sea Surface Temperature	4.0	3.0	
Variability in Sea Surface Temperature	1.0	3.0	
Salinity	1.4	3.0	
Variability Salinity	1.2	3.0	
Air Temperature	4.0	3.0	
Variability Air Temperature	1.0	3.0	
Precipitation	1.2	3.0	
Variability in Precipitation	1.3	3.0	
Ocean Acidification	4.0	2.0	
Variability in Ocean Acidification	1.0	2.2	
Currents	2.0	1.0	
Sea Level Rise	2.2	1.5	
Exposure Score	Very High		
Overall Vulnerability Rank	Very High		

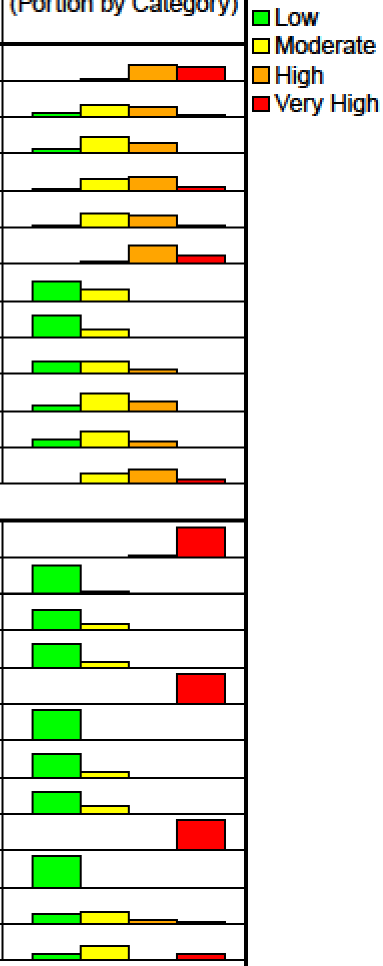

Winter Flounder (*Pseudopleuronectes americanus*)

Overall Climate Vulnerability Rank: **Very High** (64% certainty from bootstrap analysis).

Climate Exposure: **Very High**. Three exposure factors contributed to this score: Ocean Surface Temperature (4.0), Ocean Acidification (4.0) and Air Temperature (4.0). Exposure to all three factors occur during the life cycle. Two of three stocks of Winter Flounder have both obligate estuarine-dependent stocks that spawn in estuaries and a stock that spawns on the shelf. Winter flounder make seasonal onshore (winter) and offshore (summer) migrations.

Biological Sensitivity: **High**. Two sensitivity attribute scored above 3.0: Stock Status (3.4) and Early Life History Requirements (3.2). The Southern New England stock of Winter Flounder is overfished and has been so for more than a decade. Spawning occurs in the late-winter / early spring in estuaries or on Georges Bank. Eggs are benthic and larvae are planktonic.

Distributional Vulnerability Rank: **High** (74% certainty from bootstrap analysis). Two attributes indicated vulnerability to distribution shift. Adult Winter Flounder make seasonal inshore-offshore migrations and a variety of habitats are used including estuarine, coastal, and shelf.

Directional Effect in the Northeast U.S. Shelf: The effect of climate change on Winter Flounder on the Northeast U.S. Shelf is very likely to be negative (>95% certainty in expert scores). Productivity of the Southern New England stock is decreasing and this has been linked to temperature, which is expected to continue to increase in the future. The climate effect on the Georges Bank stock is more uncertain. The effect of ocean acidification over the next 30 years is likely to be minimal.

Data Quality: 92% of the data quality scores were 2 or greater.

Climate Effects on Abundance and Distribution: There is strong evidence for climate effects on Winter Flounder productivity in the southern part of its range. A number of studies have indicated that various vital rates and ecological processes are temperature dependent (see Bell 2009). Manderson (2008) hypothesized that increasing temperatures caused recruitment synchrony across multiple spawning units of winter flounder in the Mid-Atlantic region with increasing temperatures decreasing recruitment. Bell et al. (2014a) modeled the stock recruitment relationship and concluded that population productivity has been decreasing and this decrease was linked to increasing temperature. Despite climate effects on productivity, climate effects on distribution have not been identified. Bell et al (2014b) found that the distribution of the southern stock of Winter Flounder has changed little over the past decades while other species have shifted north. Spawning site fidelity is one possible explanation that keeps Winter Flounder form shifting distribution.

Life History Synopsis**:** Winter Flounder is a benthic, marine flatfish species that ranges from Labrador to Georgia (Pereira et al., 1999). Winter Flounder reach maturity after 2-5 years (NEFSC, 2011), however, females may not spawn annually, complicating age at maturity calculations (Pereira et al., 1999). Most spawning occurs in cold, shallow, estuarine waters over sand, muddy-sand, mud, and gravel substrate (Pereira et al., 1999; Klein-MacPhee, 2002), but the Georges Bank population spawns offshore and there may be limited offshore spawning in other areas (Able and Fahay, 2010). Spawning season varies latitudinally and is effected by temperature, but in general, Winter Flounder move inshore and into estuaries in fall and early winter and spawn in late winter and spring (Pereira et al., 1999; Able and Fahay, 2010). Eggs are demersal and adhesive, forming clumps (Pereira et al., 1999). Hatching occurs

after 2-3 weeks, but the incubation period is temperature dependent (Pereira et al., 1999). Larvae are briefly planktonic, but gain the ability to swim and sink vertically through the water column early and are mostly bottom oriented by metamorphosis (Pereira et al., 1999; Klein-MacPhee, 2002; Able and Fahay, 2010). Larvae are zooplanktivorous, primarily consuming invertebrate eggs and nauplii during the pelagic phase and polychaetes and copepods when larger (Pereira et al., 1999). Sarsia medusae and Atlantic Mackerel are likely predators of larval Winter Flounder (Periera et al., 1999). Metamorphosis begins after 5-6 weeks and settlement occurs approximately 8 weeks after hatch (Pereira et al., 1999). Juveniles occur in shallow waters with mud or vegetated substrates, and probably aggregate where food is abundant (Klein-MacPhee, 2002). Early juveniles primarily consume polychaetes and amphipods, but the diversity of prey increases with growth (Pereira et al., 1999). Young Bluefish, gulls, cormorants, Summer Flounder, sea robins, and Windowpane Flounder prey on juvenile Winter Flounder (Pereira et al., 1999). Emigration from estuaries to the ocean occurs in late fall or early winter of the following year (Able and Fahay, 2010). Except for the Georges Bank population, which does not migrate, adult Winter Flounder migrate back to deeper, cooler waters in summer after spawning (Pereira et al., 1999). Rising bottom water temperature and food availability drive the migration pattern (Pereira et al., 1999; Klein-MacPhee, 2002). Adult Winter Flounder are opportunistic, visual predators that eat mostly annelids, amphipods, capelin eggs, bivalves, and small fish (Pereira et al., 1999). A variety of benthic predators prey on adult Winter Flounder, including: humans, Striped Bass, Bluefish, Oyster Toadfish, cormorants, Blue Herons, seals, and Ospreys (Pereira et al., 1999). Winter Flounder are managed as three stocks: Gulf of Maine, southern New England and mid-Atlantic, and Georges Bank (Pereira et al., 1999). The Atlantic States Marine Fisheries Commission (Fishery management plan for inshore stocks of Winter Flounder) and the New England Fishery Management Council (Northeast Multispecies Fishery Management Plan) jointly manage the southern New England-mid-Atlantic and Gulf of Maine stocks. Based on the 2010 assessment, the southern New England-mid-Atlantic stock was overfished, but overfishing was not occurring; the Gulf of Maine stock was not undergoing overfishing, but the overfished status was undetermined (NEFSC, 2011). The New England Fisheries Management Council manages the Georges Bank stock, which was neither overfished nor was overfishing occurring based on the 2010 assessment (NEFSC, 2011).

Literature Cited:

Able KW, Fahay MP. Ecology of estuarine fishes: temperate waters of the western North Atlantic. Baltimore: The Johns Hopkins University Press; 2010. 566p.

Bell R J. Overwintering ecology of young-of-the-year winter flounder in Narragansett Bay, Rhode Island. PhD. Thesis, University of Rhode Island. 2009. Available: http://digitalcommons.uri.edu/dissertations/AAI3401130

Bell RJ, Hare JA, Manderson JP, Richardson DE. Externally driven changes in the abundance of summer and winter flounder. ICES J Mar Sci. 2014; 71(9): 2416-2428. doi: 10.1093/icesjms/fsu069

Bell RJ, Richardson DE, Hare JA, Lynch PD, Fratantoni PS. Disentangling the effects of climate, abundance, and size on the distribution of marine fish: an example based on four stocks from the Northeast US shelf. ICES J Mar Sci. 2014; fsu217. doi: 10.1093/icesjms/fsu217

Klein-MacPhee, G. 2002. Winter flounder/ Pseudopleuronectes americanus (Walbaum 1792). Pages 579-585. In: B.B. Collette and G. Klein-MacPhee (editors), Fishes of the Gulf of Maine, 3rd edition. Smithsonian Institution Press, Washington D.C. 882 p.

Manderson, J. P. (2008). The spatial scale of phase synchrony in winter flounder (Pseudopleuronectes americanus) production increased among southern New England nurseries in the 1990s. Canadian Journal of Fisheries and Aquatic Sciences, 65(3), 340-351.

Northeast Fisheries Science Center (NESFC). 2011. 52nd northeast regional stock assessment workshop (52nd SAW): Assessment report. US Dept. Commer, Northeast Fish. Sci. Cent. Ref. Doc. 11-17; 962 p. NMFS, 166 Water St., Woods Hole, MA 02543-1026. Accessed online (June 2014): http://www.nefsc.noaa.gov/saw/saw52/crd1117.pdf

Pereira, J. J.; R. Goldberg; J. J. Ziskowski; P. L. Berrien; W. W. Morse; D. L. Johnson. 1999. Essential Fish Habitat Source Document: Winter flounder, Pseudopleuronectes americanus, life history and habitat characteristics. NOAA Technical Memorandum NMFS-NE-138. 39 p. Accessed online (June 2014): http://www.nefsc.noaa.gov/nefsc/publications/tm/tm138/tm138.pdf

Return to Table of Contents

Winter Skate – *Leucoraja ocellata*

Overall Vulnerability Rank = Low

Biological Sensitivity = Low
Climate Exposure = High
Data Quality = 83% of scores ≥ 2

Leucoraja ocellata	Expert Scores	Data Quality	Expert Scores Plots (Portion by Category)
Stock Status	1.9	2.6	
Other Stressors	1.6	1.8	
Population Growth Rate	3.4	2.4	
Spawning Cycle	1.2	2.8	
Complexity in Reproduction	1.4	2.2	
Early Life History Requirements	1.0	3.0	
Sensitivity to Ocean Acidification	1.7	2.2	
Prey Specialization	1.1	3.0	
Habitat Specialization	1.2	2.8	
Sensitivity to Temperature	2.0	2.8	
Adult Mobility	2.0	1.8	
Dispersal & Early Life History	1.9	3.0	
Sensitivity Score	Low		
Sea Surface Temperature	3.9	3.0	
Variability in Sea Surface Temperature	1.0	3.0	
Salinity	1.6	3.0	
Variability Salinity	1.2	3.0	
Air Temperature	1.0	3.0	
Variability Air Temperature	1.0	3.0	
Precipitation	1.0	3.0	
Variability in Precipitation	1.0	3.0	
Ocean Acidification	4.0	2.0	
Variability in Ocean Acidification	1.0	2.2	
Currents	2.1	1.0	
Sea Level Rise	1.1	1.5	
Exposure Score	High		
Overall Vulnerability Rank	Low		

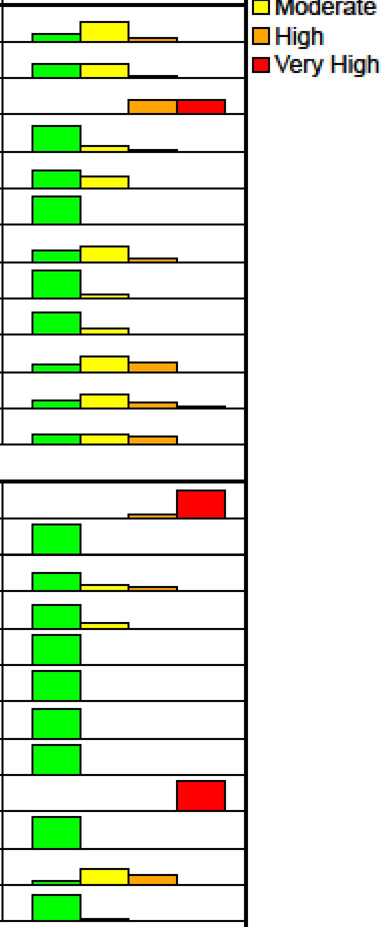

Return to Table of Contents

Winter Skate (*Leucoraja ocellata*)

Overall Climate Vulnerability Rank: **Low** (100% certainty from bootstrap analysis).

Climate Exposure: **High**. Two exposure factors contributed to this score: Ocean Surface Temperature (3.0) and Ocean Acidification (4.0). Winter Skate are demersal and complete their life cycle in marine habitats.

Biological Sensitivity: **Low**. Only one attribute scored above 2.5: Population Growth Rate (3.4). In general, skates have a low population growth rate (higher sensitivity to climate change) (Frisk, 2010).

Distributional Vulnerability Rank: **High** (88% certainty from bootstrap analysis). Winter Skate are habitat generalists and moderately mobile as adults, making seasonal movements. In addition, skate egg cases are subject to movement by currents and juveniles may move on scales of 1-10 km.

Directional Effect in the Northeast U.S. Shelf: The effect of climate change on Winter Skate is estimated to be negative, but this estimate has a high degree of uncertainty (<66% certainty in expert scores). Winter Skate are a cold water species and reductions in productivity may occur because of warming and ocean acidification. However, distributions have shifted southwards in recent years, contrary to the expectation of a northward shift with warming.

Data Quality: 83% of the data quality scores were 2 or greater indicate that data quality is moderate.

Climate Effects on Abundance and Distribution: Little specific information exists on the effect of climate on Winter Skate. Di Santo (2015) found that increased warming and acidification reduce body condition of newly-hatched Little Skate – a congener. These reductions in size could result in reduced juvenile survival and thus recruitment. Nye et al. (2009) found a shift southward accompanied by a shift into deeper water. Frisk et al. (2008) also identified a distribution shift, suggesting population connectivity between Georges Bank and the Scotian Shelf. The cause of this distribution shift remains unexplained.

Life History Synopsis**:** Winter Skate is a benthic marine species ranging from the south coast of Newfoundland and southern Gulf of St. Lawrence to Cape Hatteras, North Carolina (Packer et al., 2003). Winter Skate are sexually mature by 70-109 cm or approximately 7-11 years old and mate using internal fertilization (Packer et al., 2003; Hogan et al., 2013). Winter Skate may spawn year round, but the peak egg production occurs in summer and fall (Packer et al., 2003; Hogan et al., 2013). Eggs are encapsulated singly in an egg case with stiff pointed horns at the corners and young skates emerge from these encapsulated eggs fully developed (Packer et al., 2003). Juvenile and adult skate have similar distribution and habitat patterns. Winter Skate are found in a wide range of temperatures, salinities, and depths, but are mainly inshore (<100 m) and in estuaries in fall – spring and on Georges Bank in summer, but are absent from the Gulf of Maine in winter (Packer et al., 2003). Winter Skate are most abundant on sand, gravel, and sometimes mud substrate where they are buried during the day and emerge into the water column at night (Packer et al., 2003). Juvenile and adult skate prey on a variety of benthic infauna, including: polychaetes, amphipods, isopods, crabs, and small fish (Packer et al., 2003). The importance of fish and polychaetes to the diet increases with size while the consumption of amphipods and crustaceans decreases (Packer et al., 2003). Sharks, other skates, gray seals, and gulls are the dominant predators of winter skate (Packer et al., 2003; Swain et al., 2013). Winter Skate are managed as part of a skate complex with six other species under the New England Fishery Management

Council's Skate Fishery Management Plan. Based on the most recent assessment, Winter Skate are not overfished, but overfishing is occurring (NESFC, 2007).

Literature Cited:

Di Santo V. Ocean acidification exacerbates the impacts of global warming on embryonic little skate, *Leucoraja erinacea* (Mitchill). J Exp Mar Bio Ecol. 2015; 463: 72-78. doi:10.1016/j.jembe.2014.11.006

Frisk MG, Miller TJ, Martell SJD, Sosebee K. New hypothesis helps explain elasmobranch "outburst" on Georges Bank in the 1980s. Ecol Appl. 2008; 18(1): 234-245. doi: 10.1890/06-1392.1

Frisk MG. Life history strategies of batoids. In: Carrier, JC, Musick, JA, Heithaus, MR, editors, Sharks and Their Relatives II: Biodiversity, Adaptive Physiology, and Conservation. Boca Raton: CRC Press; 2010. 283-318.

Hogan F, Cadrin SX, Haygood A. Fishery management complexes: An impediment or aid to sustainable harvest? A discussion based on the Northeast Skate Complex. N Am J Fish Manag. 2013; 33: 406-421. doi: 10.1080/02755947.2013.763873

Northeast Fisheries Science Center (NEFSC). 2007. 44th Northeast Regional Stock Assessment Workshop (44th SAW): 44th SAW assessment report. US Dep Commer, Northeast Fish Sci Cent Ref Doc 07-10; 661 p. Accessed online (August 2015): http://www.nefsc.noaa.gov/publications/crd/crd0710/

Nye JA, Link JS, Hare JA, Overholtz WJ. Changing spatial distribution of fish stocks in relation to climate and population size on the Northeast United States continental shelf. Mar Ecol Prog Ser. 2009; 393: 111-129. doi: 10.3354/meps08220

Packer DB, Zetlin CA, Vitaliano JJ. 2003. Essential fish habitat source document: Winter Skate, Leucoraja ocellata, life history and habitat characteristics. NOAA Tech. Memo. NMFS-NE-179. 57 p. Accessed online (August 2015): http://www.nefsc.noaa.gov/nefsc/publications/tm/tm179/tm179.pdf

Return to Table of Contents

Witch Flounder – *Glyptocephalus cynoglossus*

Overall Vulnerability Rank = High

Biological Sensitivity = High
Climate Exposure = High
Data Quality = 83% of scores ≥ 2

Glyptocephalus cynoglossus	Expert Scores	Data Quality	Expert Scores Plots (Portion by Category)
Stock Status	3.9	2.8	
Other Stressors	1.5	1.8	
Population Growth Rate	3.4	2.5	
Spawning Cycle	2.1	3.0	
Complexity in Reproduction	2.0	2.0	
Early Life History Requirements	1.9	1.4	
Sensitivity to Ocean Acidification	1.4	2.2	
Prey Specialization	1.8	3.0	
Habitat Specialization	1.2	3.0	
Sensitivity to Temperature	1.7	2.8	
Adult Mobility	2.5	2.8	
Dispersal & Early Life History	1.2	2.6	
Sensitivity Score	High		
Sea Surface Temperature	3.9	3.0	
Variability in Sea Surface Temperature	1.0	3.0	
Salinity	1.4	3.0	
Variability Salinity	1.2	3.0	
Air Temperature	1.0	3.0	
Variability Air Temperature	1.0	3.0	
Precipitation	1.0	3.0	
Variability in Precipitation	1.0	3.0	
Ocean Acidification	4.0	2.0	
Variability in Ocean Acidification	1.0	2.2	
Currents	2.1	1.0	
Sea Level Rise	1.1	1.5	
Exposure Score	High		
Overall Vulnerability Rank	High		

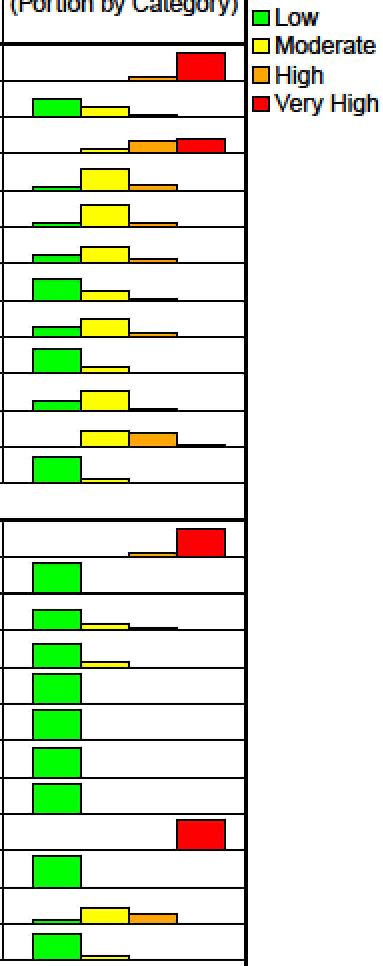

Return to Table of Contents

Witch Flounder (*Glyptocephalus cynoglossus*)

Overall Climate Vulnerability Rank: **High** (100% certainty from bootstrap analysis).

Climate Exposure: **High**. Two exposure factors contributed to this score: Ocean Surface Temperature (3.9) and Ocean Acidification (4.0). All life stages of Witch Flounder use marine habitats.

Biological Sensitivity: **High**. Two sensitivity attributes scored above 3.0: Stock Status (3.9) and Population Growth Rate (3.4). Based on the recent assessment, witch flounder are overfished and overfishing is occurring (NEFSC, 2012). Witch Flounder are relatively slow growing, late maturing (4-8 years) and long-lived (30 years) (Pentilla and Dery, 1988).

Distributional Vulnerability Rank: **High** (100% certainty from bootstrap analysis).

Directional Effect of Climate Change: The effect of climate change on Witch Flounder on the Northeast U.S. Shelf is estimated to be negative, but this estimate is uncertain (66-90% certainty in expert scores). Witch Flounder is a cold-temperate species and warming will likely cause reductions in available habitat. However, there is little direct evidence of the effect of climate change on Witch Flounder productivity and distribution, which contributes to the uncertainty.

Data Quality: 83% of the data quality scores were 2 or greater indicate that data quality is moderate.

Climate Effects on Abundance and Distribution: There is relatively little information regarding the effect of climate on Witch Flounder productivity and distribution. Regional studies in the Northeast U.S. Shelf did not include Witch Flounder (Murawski, 1993; Nye et al., 2009). Studies in the Northeast Atlantic found little change in Witch Flounder distribution (Perry et al., 2005, Dulvy et al., 2008).

Life History Synopsis**:** Witch Flounder is a benthic, marine, flatfish species found on both sides of the Atlantic Ocean, but from Labrador, Canada, to Cape Hatteras, North Carolina in the western Atlantic (Cargnelli et al., 1999). Females mature at 5-8 years old and get larger and older than males (Cargnelli et al., 1999; NEFSC, 2012). Dense spawning aggregations on or near the sea floor form in areas of cold water from March to November (Cargnelli et al., 1999). Eggs and larvae are pelagic over deep water (Cargnelli et al., 1999). Eggs hatch after a week, but larvae remain pelagic for 4 – 12 months and settlement size varies from 20-68 mm (Cargnelli et al., 1999; Klein-MacPhee, 2002). Juvenile Witch Flounder occur in deep cold water over mud, clay, silt, or muddy-sand substrate, often on the shelf slope (Cargnelli et al., 1999). The proportion of prey shifts from crustacean dominated to polychaete dominated with age (Cargnelli et al., 1999). Although their habitat overlaps, adult Witch Flounder tend to be collected on shallower portions of the shelf than juveniles (Cargnelli et al., 1999; Klein-MacPhee, 2002). Soft mud, silt, muddy-sand, and clay substrate are preferred habitat by the species, possibly due to their preference for polychaete prey (Cargnelli et al., 1999). The dominant predators of Witch Flounder include Thorny Skates and Smooth Skates, Spiny Dogfish, Monkfish (Goosefish), White Hake, Atlantic Halibut, and harp seal (Klein-MacPhee, 2002). Witch Flounder are managed under the New England Fishery Management Council's Northeast Multispecies Fishery Management Plan. Based on the recent assessment, witch flounder are overfished and overfishing is occurring (NEFSC, 2012).

Literature Cited:

Cargnelli LM, Griesbach SJ, Packer DB, Berrien PL, Morse WW, Johnson DL. Essential Fish Habitat Source Document: Witch flounder, *Glyptocephalus cynoglossus*, life history and habitat characteristics. NOAA

Tech. Memo. 1999; NMFS-NE-139: 29p. Accessed online (August 2015): http://www.nefsc.noaa.gov/nefsc/publications/tm/tm139/tm139.pdf

Dulvy NK, Rogers SI, Jennings S, Stelzenmüller V, Dye SR, Skjoldal HR. Climate change and deepening of the North Sea fish assemblage: a biotic indicator of warming seas. Journal of Applied Ecology, 2008; 45(4): 1029-1039. DOI: 10.1111/j.1365-2664.2008.01488.x

Klein-MacPhee, G. 2002. Witch flounder/ *Glyptocephalus cynoglossus* (Linnaeus 1758). In: Collette BB, Klein-MacPhee G (editors). Bigelow and Schroeder's fishes of the Gulf of Maine. 3rd Edition. Washington, DC: Smithsonian Institution Press. 2002; 334-338.

Murawski SA. Climate change and marine fish distributions: forecasting from historical analogy. Trans Amer Fish Soc. 1993; 122(5): 647-658. DOI: 0.1577/1548-8659(1993)122<0647:CCAMFD>2.3.CO;2

Northeast Fisheries Science Center (NEFSC). Assessment or Data Updates of 13 Northeast Groundfish Stocks through 2010. US Dept Commer, Northeast Fish Sci Cent Ref Doc. 2012; 12-06: 789 p. Accessed online (June 2014): http://nefsc.noaa.gov/publications/crd/crd1206/

Nye JA, Link JS, Hare JA, Overholtz WJ. Changing spatial distribution of fish stocks in relation to climate and population size on the Northeast United States continental shelf. Mar Ecol Prog Ser. 2009; 393: 111-129. DOI: 0.3354/meps08220

Pentilla J, Dery LM. Age determination methods for northwest Atlantic species. NOAA Tech. Rep. 1999; 72, 135 pp. Accessed Online (August 2015): http://www.nefsc.noaa.gov/publications/classics/penttila1988/penttila1988.pdf

Perry AL, Low PJ, Ellis JR, Reynolds JD. Climate change and distribution shifts in marine fishes. Science, 2005; 308(5730): 1912-1915. DOI:10.1126/science.1111322

Yellowtail Flounder – *Limanda ferruginea*

Overall Vulnerability Rank = Low

Biological Sensitivity = Low
Climate Exposure = High
Data Quality = 88% of scores ≥ 2

Limanda ferruginea	Expert Scores	Data Quality	Expert Scores Plots (Portion by Category)
Stock Status	3.2	2.6	
Other Stressors	1.8	1.8	
Population Growth Rate	1.8	2.8	
Spawning Cycle	2.2	3.0	
Complexity in Reproduction	1.2	2.4	
Early Life History Requirements	2.4	2.6	
Sensitivity to Ocean Acidification	1.2	2.2	
Prey Specialization	1.5	2.8	
Habitat Specialization	1.3	3.0	
Sensitivity to Temperature	2.5	2.8	
Adult Mobility	2.0	2.8	
Dispersal & Early Life History	1.2	3.0	
Sensitivity Score	Low		
Sea Surface Temperature	3.9	3.0	
Variability in Sea Surface Temperature	1.0	3.0	
Salinity	1.4	3.0	
Variability Salinity	1.2	3.0	
Air Temperature	1.0	3.0	
Variability Air Temperature	1.0	3.0	
Precipitation	1.0	3.0	
Variability in Precipitation	1.0	3.0	
Ocean Acidification	4.0	2.0	
Variability in Ocean Acidification	1.0	2.2	
Currents	2.1	1.0	
Sea Level Rise	1.1	1.5	
Exposure Score	High		
Overall Vulnerability Rank	Low		

Low
Moderate
High
Very High

Yellowtail Flounder (*Limanda ferruginea*)

Overall Climate Vulnerability Rank: **Low** (41% certainty from bootstrap analysis).

Climate Exposure: **High**. Two exposure factors contributed to this score: Ocean Surface Temperature (3.9) and Ocean Acidification (4.0). All life stages of Yellowtail Flounder use marine habitats.

Biological Sensitivity: **Low**. Only one sensitivity attributes scored above 2.5: Stock Status (3.2). One attribute above 2.5 is scored as a Low sensitivity.

Distributional Vulnerability Rank: **High** (100% certainty from bootstrap analysis). Yellowtail Flounder are habitat generalists, moderately mobile and have dispersive early life stages (Johnson et al., 1999).

Directional Effect in the Northeast U.S. Shelf: The effect of climate change on Yellowtail Flounder on the Northeast U.S. Shelf is very likely to be negative (>95% certainty in expert scores). Recruitment of the southern stock has decreased and this has been linked to warming. The species has also shifted northward in recent years as temperatures have warmed. Decreasing productivity and northward shifts will lead to negative consequences for Yellowtail Flounder in the coming years.

Data Quality: 88% of the data quality scores were 2 or greater indicate that data quality is moderate.

Climate Effects on Abundance and Distribution: Yellowtail Flounder recruitment success has been linked to ocean temperatures (Sissenwine, 1974) and more specifically to the volume of the cold pool, an area of winter water on the bottom of the Mid-Atlantic Bight during summer (Sullivan et al., 2005). Continued warming in the Mid-Atlantic will likely reduce population productivity further. The distribution of Yellowtail Flounder is related to temperature (Murawski, 1993) and the population has shifted northward in recent years (Nye et al., 2009). These study indicate a clear negative effect of climate change on Yellowtail Flounder in the Northeast U.S. Shelf ecosystem.

Life History Synopsis**:** Yellowtail Flounder are a benthic marine flatfish found from the Gulf of St. Lawrence, Labrador, and Newfoundland to the Chesapeake Bay (Johnson et al., 1999; Klein-MacPhee, 2002). Like many flatfish, yellowtail is sexually dimorphic, with larger, older females (Klein-MacPhee, 2002). Yellowtail grow faster and matures earlier than most other western Atlantic flatfish, with most females mature by their third year (Johnson et al., 1999; NEFSC, 2008). Spawning occurs from mid-February to July along the continental shelf, beginning in the southern part of the range and quickly expanding northward (Klein-MacPhee, 2002). Females batch spawn over the course of a month, depositing eggs on the bottom for fertilization (Klein-MacPhee, 2002; NESFC, 2012b). After fertilization, eggs float near the surface, and hatch into pelagic larvae after about a week (Johnson et al., 1999). Larvae occur near the surface at night and down to 20 m during the day, with larger larvae making longer migrations (Klein-MacPhee, 2002). Most larvae are collected inside the 100 m isobath in 5-17 °C water (Johnson et al., 1999). Atlantic Mackerel eat larval yellowtail, especially when copepod nauplii abundances are low (Klein-MacPhee, 2002). After approximately 2-3 months as pelagic larvae (Johnson et al., 1999; NEFSC, 2012b), 11-16 mm fish settle to mid-shelf bottom habitats, using temperature (4-8 °C) and depth (41-70 m) to determine suitable habitat (Johnson et al., 1999; Klein-MacPhee, 2002). Juvenile abundance is higher in the mid-Atlantic cold pool, initially settling in shallower water, but moving to deeper water during the months after settlement (Klein-MacPhee, 2002). Juveniles eat small benthic invertebrates such as polychaetes (Johnson et al., 1999). Adults prefer offshore sand or mixed sand and mud substrate in cool to intermediate temperature, high salinity water (Klein-MacPhee, 2002),

but are also found in some rivers, bays, and harbors (Johnson et al., 1999). There are three distinct stocks in United States waters, with little mixing among regions, except for short migrations east in spring and summer, west in fall and winter by Southern New England and Georges Bank stocks (Johnson et al., 1999; Klein-MacPhee, 2002). Amphipods and polychaetes are the main prey of Yellowtail Flounder, with occasional consumption of other benthic invertebrates and small fish (Johnson et al., 1999; Klein-MacPhee, 2002). Predators include Spiny Dogfish, Atlantic Cod, several skate species, and several other benthic piscivores (Johnson et al., 1999; Klein-MacPhee, 2002). Yellowtail Flounder has been exploited since the 1930's, with wide swings in abundance since the 1970's (Johnson et al., 1999). The New England Fishery Management Council manages the species as three stocks under the Northeast Multispecies Fishery Management Plan: Cape Cod-Gulf of Maine, Georges Bank, and Southern New England-Mid Atlantic (NESFC, 2008). The Cape Cod-Gulf of Maine and Georges Bank stocks are overfished and overfishing is occurring (NEFSC, 2008; NEFSC, 2012a). The Southern New England-Mid Atlantic stock is considered rebuilt , although biomass stock status is uncertain , but not experiencing overfishing (NEFSC, 2012b).

Literature Cited:

Johnson DL, Morse WW, Berrien PL, Vitaliano JJ. Essential Fish Habitat Source Document: Yellowtail flounder, Limanda ferruginea, life history and habitat characteristics. NOAA Tech. Memo. 1999; NMFS-NE-140: Accessed online (August 2015): http://www.nefsc.noaa.gov/nefsc/publications/tm/tm140/tm140.pdf

Klein-MacPhee G. Yellowtail flounder/ *Limanda ferruginea* (Storer 1839). Pages 572–576 In: Collette BB, Klein-MacPhee G (editors), Fishes of the Gulf of Maine, 3rd edition. Smithsonian Institution Press, Washington D.C. 2002; 882 p.

Murawski SA. Climate change and marine fish distributions: forecasting from historical analogy. Trans Amer Fish Soc. 1993; 122(5): 647-658. DOI: 0.1577/1548-8659(1993)122<0647:CCAMFD>2.3.CO;2

Northeast Fisheries Science Center (NEFSC). Assessment or Data Updates of 13 Northeast Groundfish Stocks through 2010. US Dept Commer, Northeast Fish Sci Cent Ref Doc. 2012; 12-06: 789 p. Accessed online (August 2015) http://nefsc.noaa.gov/publications/crd/crd1206/

Northeast Fisheries Science Center (NEFSC). Assessment of 19 Northeast Groundfish Stocks through 2007: Report of the 3rd Groundfish Assessment Review Meeting (GARM III), Northeast Fish Sci Cent Ref Doc. 2008; 08-15: 884 p + xvii. Accessed online (August 2015): http://nefsc.noaa.gov/publications/crd/crd0815/

Northeast Fisheries Science Center (NEFSC). 54th Northeast Regional Stock Assessment Workshop (54th SAW) Assessment Report. US Dept Commer, Northeast Fish Sci Cent Ref Doc. 2012; 12-18: 600 p. Accessed online (August 2015): http://nefsc.noaa.gov/publications/crd/crd1218/

Nye JA, Link JS, Hare JA, Overholtz WJ. Changing spatial distribution of fish stocks in relation to climate and population size on the Northeast United States continental shelf. Mar Ecol Prog Ser. 2009; 393: 111-129. DOI: 0.3354/meps08220

Sissenwine, M. P. (1974). Variability in recruitment and equilibrium catch of the Southern New England yellowtail flounder fishery. ICES J Mar Sci. 1974; 36(1): 15-26. DOI: 10.1093/icesjms/36.1.15

Return to Table of Contents

Sullivan MC, Cowen RK, Steves BP. Evidence for atmosphere–ocean forcing of yellowtail flounder (*Limanda ferruginea*) recruitment in the Middle Atlantic Bight. Fisher Oceanogr. 2005; 14(5): 386-399. DOI: 10.1111/j.1365-2419.2005.00343.x
