# Supplementary material for: A Vulnerability Assessment of Fish and Invertebrates to Climate Change on the Northeast U.S. Continental Shelf
Source: PLoS One. 2016 Feb 3;11(2):e0146756. doi: 10.1371/journal.pone.0146756 (PMC4739546; doi:10.1371/journal.pone.0146756)

S8 Supporting Information. Climate Vulnerability and Distribution Change Potential

When compared across all species, climate vulnerability was negatively correlated to potential for a distribution shift (spearman rank correlation -0.61, $p<0.001$).

Figure S8. Climate vulnerability compared to potential for a distribution change. Size of symbol represents number of species; the number of species is also provided in the center of each symbol.

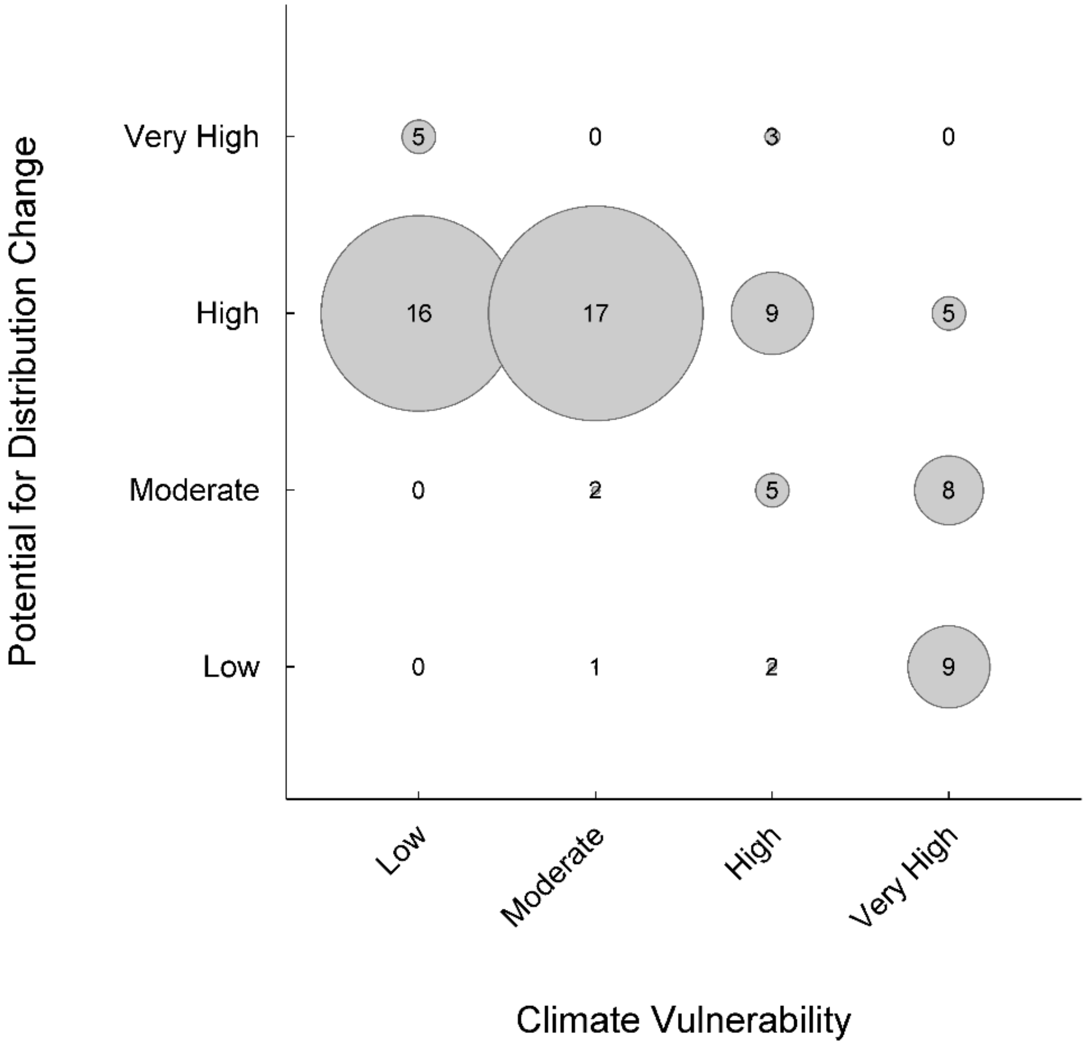

Supplement: S8 Supporting Information — (PDF) [file pone.0146756.s010.pdf]
