# Supplementary material for: A Vulnerability Assessment of Fish and Invertebrates to Climate Change on the Northeast U.S. Continental Shelf
Source: PLoS One. 2016 Feb 3;11(2):e0146756. doi: 10.1371/journal.pone.0146756 (PMC4739546; doi:10.1371/journal.pone.0146756)
Supplement: S9 Supporting Information — (PDF) [file pone.0146756.s011.pdf]

S9 Supporting Information. Climate Exposure Maps

Projected change in climate exposure factors: ocean surface temperature, surface air temperature, ocean pH, precipitation, and ocean surface salinity. Historical stdanom shows the mean difference (2006-2055 – 2005-1956) divided by the standard deviation over the historical period (1956-2005). Historical variance ratio shows the variance for the future period (2006-2055) divided by the variance of the historical period (1956-2005). These maps were compared with species distributions to determine climate exposure using the rubric presented in Table 3. Maps were obtained from NOAA's Climate Change Web Portal.

Ocean Surface Temperatures (Mean [top] and variance [bottom])

return to first page

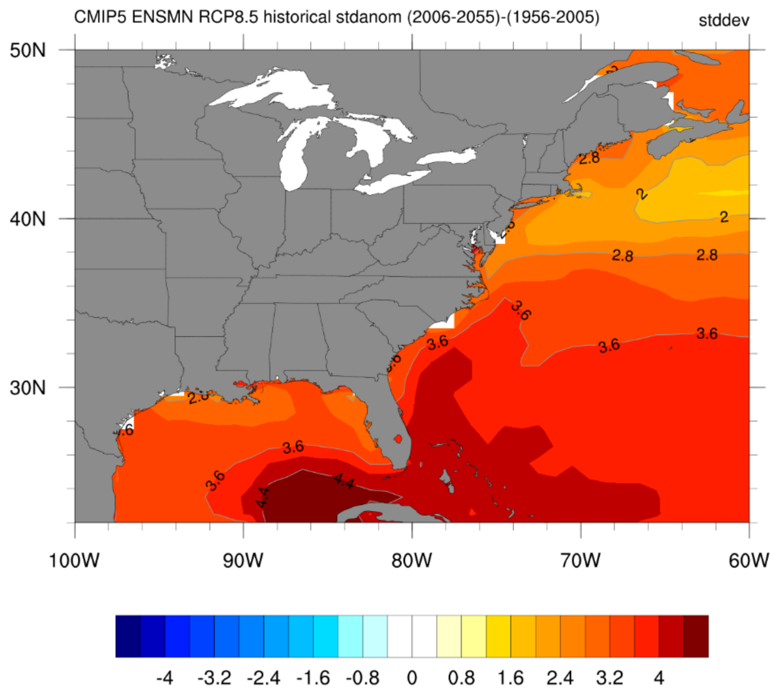

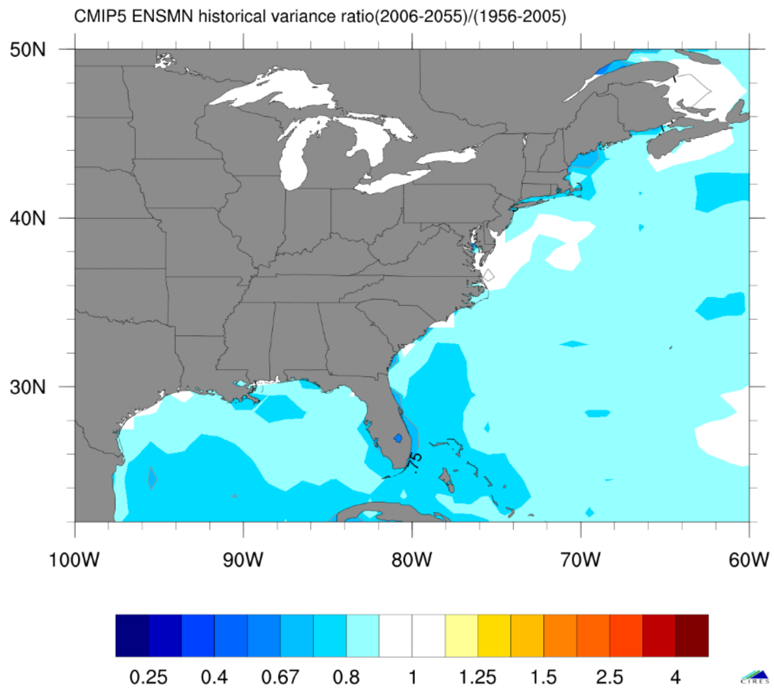

return to first page

Surface Air Temperatures (Mean [top] and variance [bottom])

return to first page

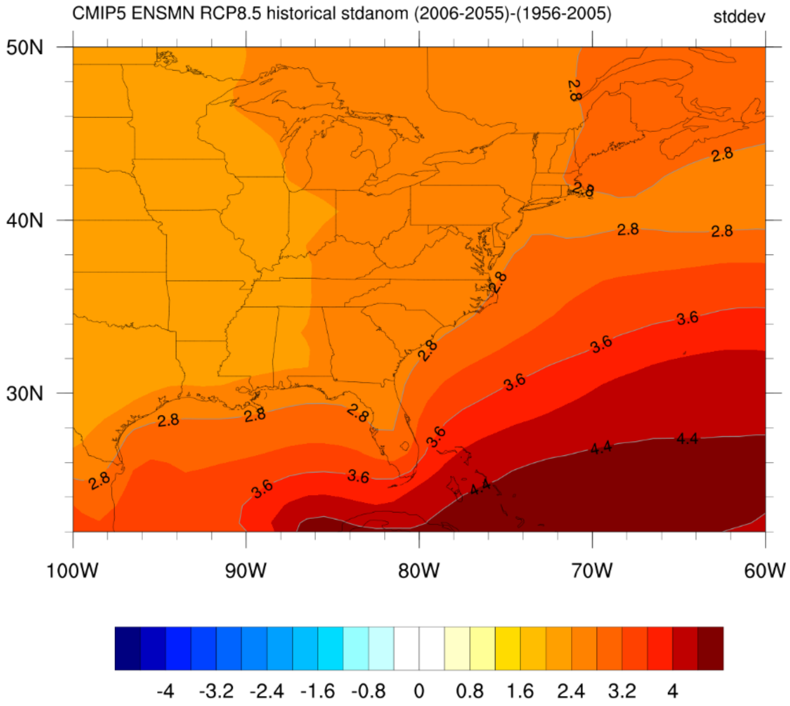

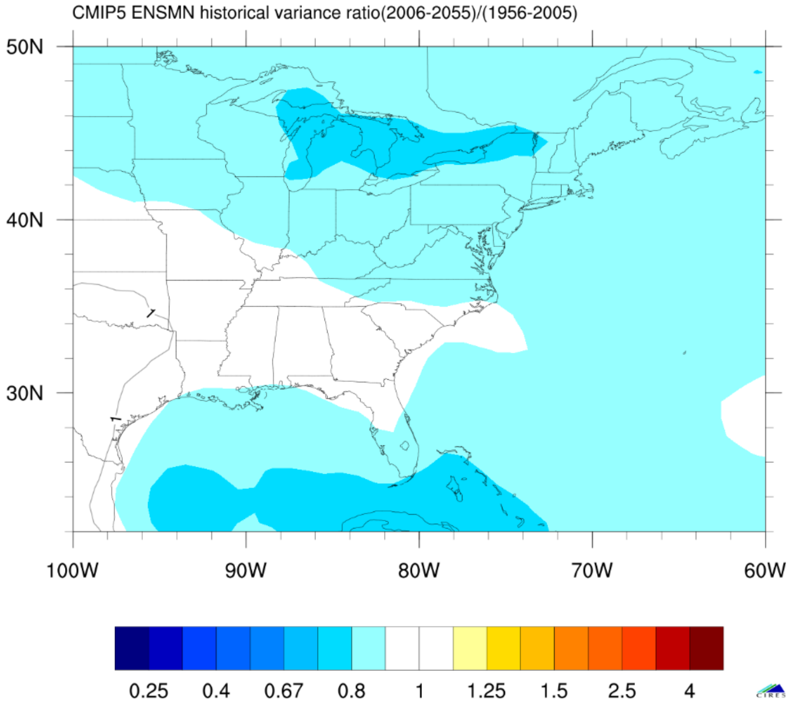

return to first page

Ocean pH (Mean [top] and variance [bottom])

return to first page

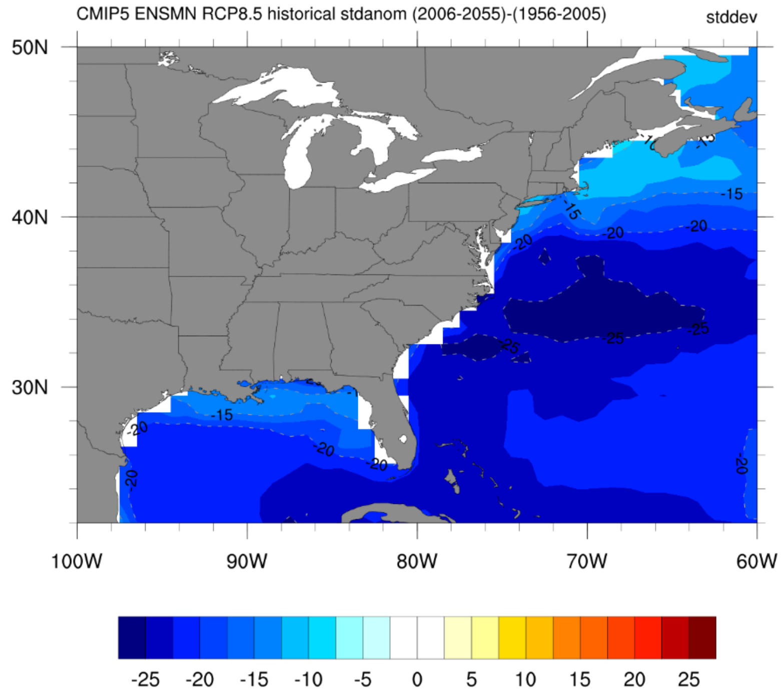

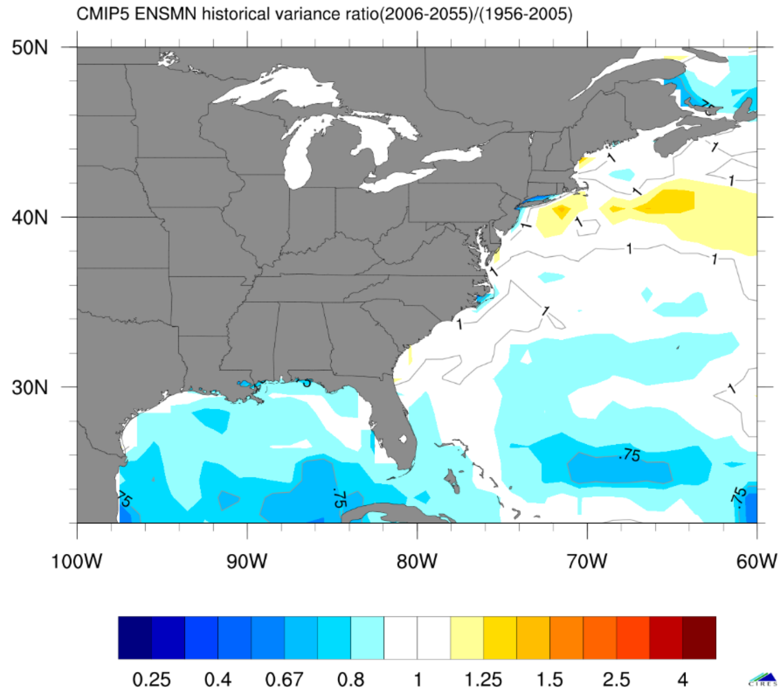

return to first page

Precipitation (Mean [top] and variance [bottom])

return to first page

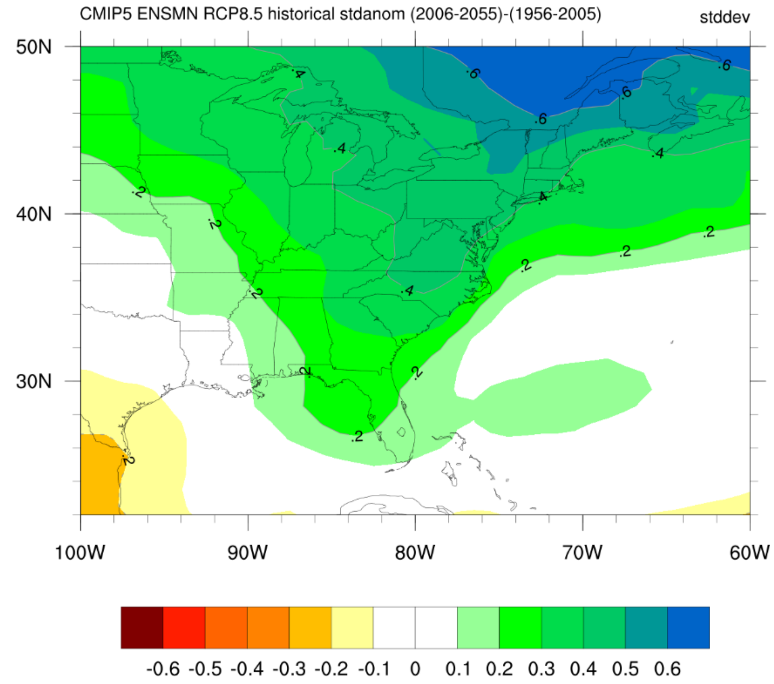

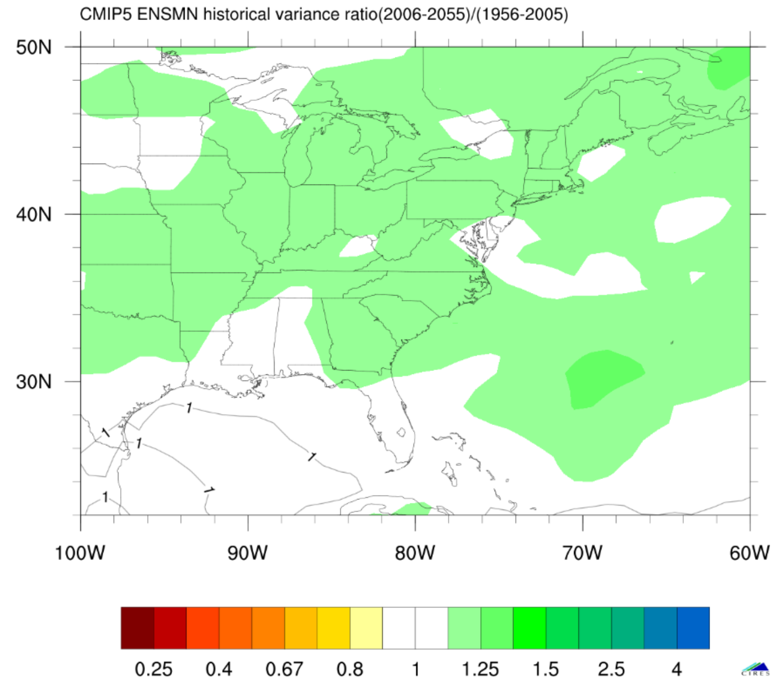

return to first page

Ocean Surface Salinity (Mean [top] and variance [bottom])

return to first page

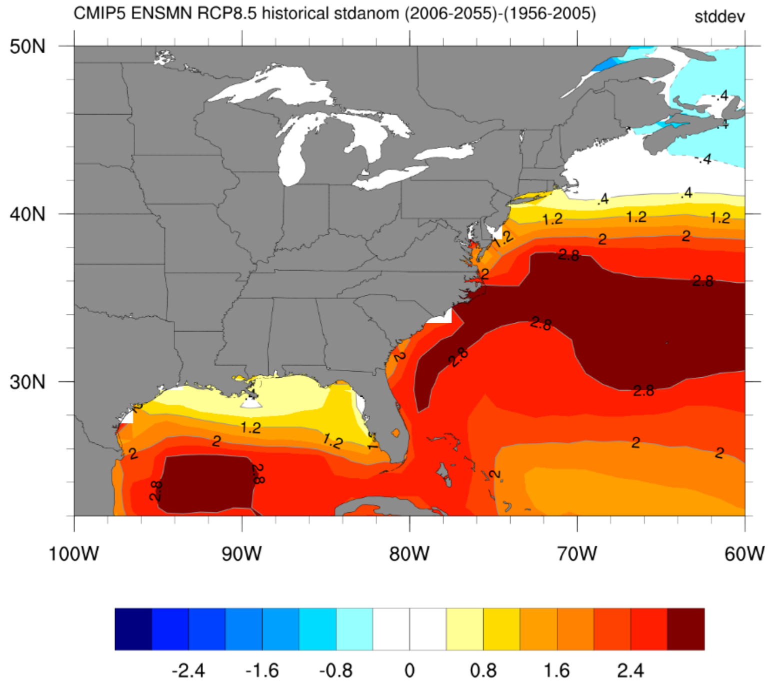

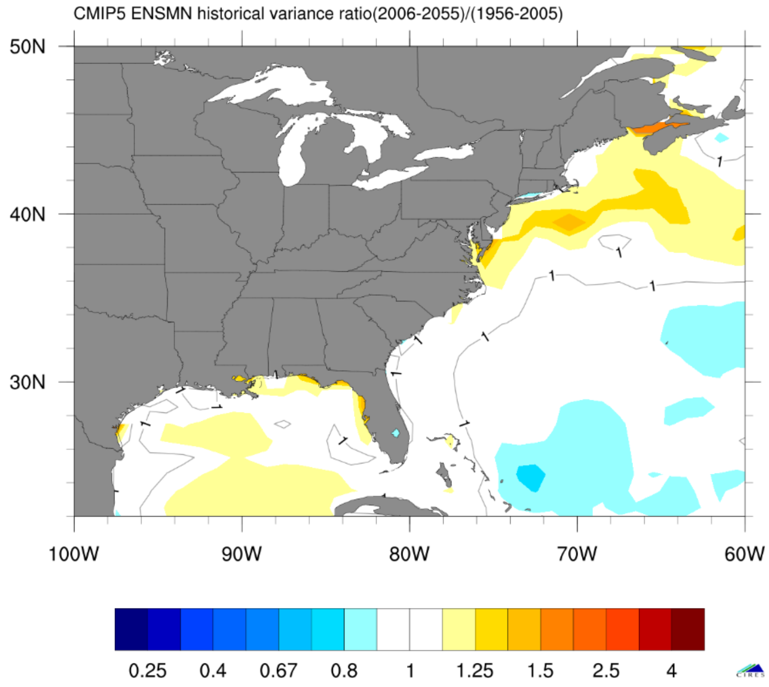

return to first page
