# Supplementary material for: A Vulnerability Assessment of Fish and Invertebrates to Climate Change on the Northeast U.S. Continental Shelf
Source: PLoS One. 2016 Feb 3;11(2):e0146756. doi: 10.1371/journal.pone.0146756 (PMC4739546; doi:10.1371/journal.pone.0146756)

S10 Supporting Information. MDS Ordination of Sensitivity Attributes

Non-metric multidimensional scaling (MDS) was used to evaluate the consistency among species within functional groups. A matrix of 82 species by 12 attribute scores was generated. A Euclidean distance matrix was then calculated between each species pair. MDS analyses were conducted in MatLab using the functions pdist and mdscale. The resulting ordination was visualized in two-dimensions for the 82 species by functional group.

Sensitivity attributes were generally consistent within functional groups, but there was overlap. Many Benthic Invertebrate species were distinct as a group in the MDS ordination. However, some Benthic Invertebrate species ordinated more closely with other groups. Most of the other groups were distinct, but the analysis indicated that many Elasmobranch and Groundfish species shared similarities and that many Pelagic Fish and Cephalopod and Coastal Fish species shared some similarities. The MDS analysis suggests that an assessment at the group-level or using select species as proxies for a group of species would yield similar results, but that in some cases, species-specific differences in attributes would be missed. For example, American Lobster (AL), Cancer crabs (CC), and Deep-Sea Red Crab (RC) have attributes more similar to Groundfish species than to other Benthic Invertebrate species (Fig. 9). Similarly, Sand Lances (SL, Pelagic Fish and Cephalopod functional group) has similarities to species to species in the Coastal Fish functional group and Tautog (Tt, Coastal functional group) has similarities to species in the Groundfish and Benthic Invertebrates functional groups.

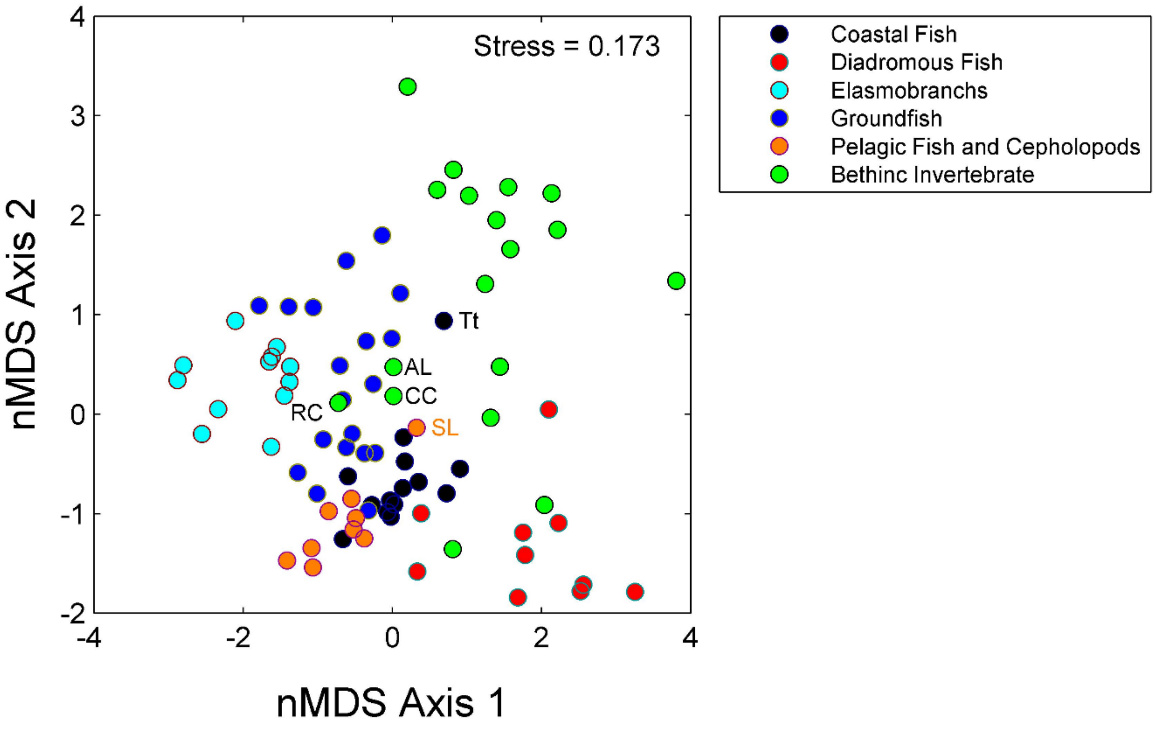

Supplement: S10 Supporting Information — (PDF) [file pone.0146756.s012.pdf]
