# Supplementary material for: A Vulnerability Assessment of Fish and Invertebrates to Climate Change on the Northeast U.S. Continental Shelf
Source: PLoS One. 2016 Feb 3;11(2):e0146756. doi: 10.1371/journal.pone.0146756 (PMC4739546; doi:10.1371/journal.pone.0146756)
Supplement: S11 Supporting Information — (PDF) [file pone.0146756.s013.pdf]

## S11 Supporting Information. Methodology Evaluation

In general the methodology developed by Morrison et al. (2015) performed well. Most exploited fish and invertebrate species in the region were included. Species were spread across the spectrum of sensitivity attributes (Low to Very High). Spread in climate exposure was limited owing to the magnitude of change projected for the region; in other regions, the exposure may exhibit more variability.

With expert opinion, expert bias is a concern. Multiple experts and a workshop for experts to discuss scoring appeared to have limited expert bias. Sensitivity analysis indicated that removing the scores of individual experts resulted in the overall climate vulnerability scores of 23% of species changing (Figure S11). In other words, the scores of 77% of species were robust to the removal of all individual experts (5 experts of sensitivity attributes and 4 experts for climate exposure factors). For those scores that did change with the removal of an expert, in all cases the climate vulnerability changed by one category (e.g., Low to Moderate). The change in scores was almost equal in terms of increasing (e.g., Low to Moderate) and decreasing (Very High to High).

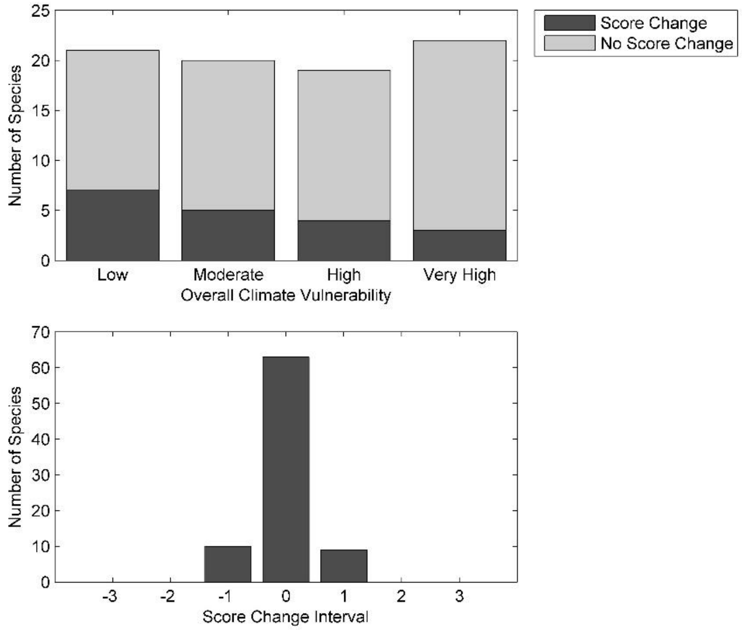


Figure S11 - Frequency distribution of score change and no score change by overall climate vulnerability and direction of change.

There were three species that scored as Very High in the assessment whose scores changed to High with the exclusion of some experts: Blue Crab, Striped Bass, and Norther Quahog. These species all had lower certainty (<90%) in the overall score as determined by the bootstrapping analysis. There were four species that scored as High in the assessment whose scores changed with the exclusion of some experts: Conger Eel, Cusk, Sand Tiger, and Spotted Seatrout. Three of these species had lower certainty (<90%) in the overall score as determined by the bootstrapping analysis. We conclude that the results of this assessment are internally robust to expert bias and that the bootstrapping analysis portrays much of the expert effect that is

present in the analysis. Future efforts could evaluate whether the entire process is robust (i.e., repeat the assessment with different experts). Another possibility would to have had each expert score every species, but this would have required an unreasonable time commitment from the experts.

The results obtained here are qualitatively similar to the climate vulnerability assessment of Gaichas et al. (2014), which was also completed in the Northeast U.S. Shelf Ecosystem. Gaichas et al. (2014) examined three functional groups (Demersal, Pelagic, and Benthic Invertebrates) in two regions of the Northeast U.S. Shelf Ecosystem. Benthic invertebrates were rated most sensitive, with Demersals intermediate and Pelagics lowest. These results agree with the functional group results presented here. In the assessment reported here, the sensitivity attributes were broadly similar within functional groups, thus similarity with the results of Gaichas et al. (2014) are expected: Benthic Invertebrates were more vulnerable than Groundfish species, which were more vulnerable than Pelagic Fish and Cephalopods. The vulnerability assessment of Gaichas et al (2014) was more rapid than the assessment reported here. The choice between less and more detailed vulnerability assessments depends on the specific objectives and the resources available to conduct the assessment. In addition, the choice would vary between regions with respect to the amount of information available, the species in a region, and the specific objectives of the assessment.

Gaichas SK, Link JS, Hare JA. A risk-based approach to evaluating northeast US fish community vulnerability to climate change. ICES J Mar Sci. 2014; 71(8): 2323-2342. doi: 10.1093/icesjms/fsu048

Morrison W, Nelson M, Howard J, Teeters E, Hare JA, Griffis R. et al. Methodology for assessing the vulnerability of fish stocks to a changing climate. NOAA Technical Memorandum. 2015; NMFS-OSF-3: 1-48.
http://www.st.nmfs.noaa.gov/Assets/ecosystems/climate/documents/TM%20OSF3.pdf
