# Supplementary material for: A Vulnerability Assessment of Fish and Invertebrates to Climate Change on the Northeast U.S. Continental Shelf
Source: PLoS One. 2016 Feb 3;11(2):e0146756. doi: 10.1371/journal.pone.0146756 (PMC4739546; doi:10.1371/journal.pone.0146756)

S12 Supporting Information. Climate Model Uncertainty

Examining among-model uncertainty showed that the results were somewhat sensitive to the climate models used. The mean expected change in ocean surface temperature was 2.5 standard deviates and the very high threshold was 2 standard deviates (Table 3). However, there was among-model variability in the projected magnitude of change and this variability had a standard deviation of approximately 1. This means that for ocean surface temperature (upper 10 m), approximately 75% of the models in the ensemble resulted in a very high climate exposure and 25% of the models results in a high exposure. These comparisons are similar for surface air temperature. For surface pH, the projected standardized magnitude of change is large (~20), so even though there is a broader spread among models (standard deviation 8), all models individually would have resulted in a Very High climate exposure score. Future iterations of the climate vulnerability assessment should incorporate ensemble member variability.

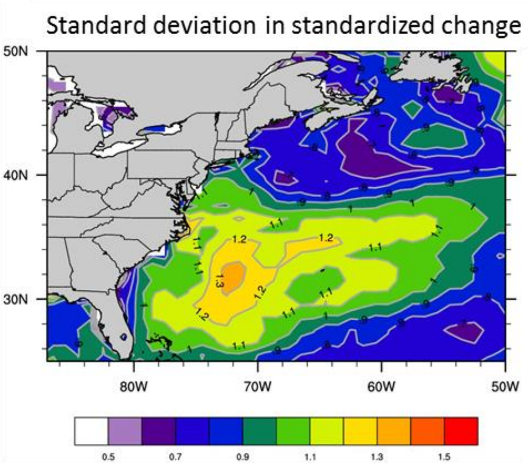

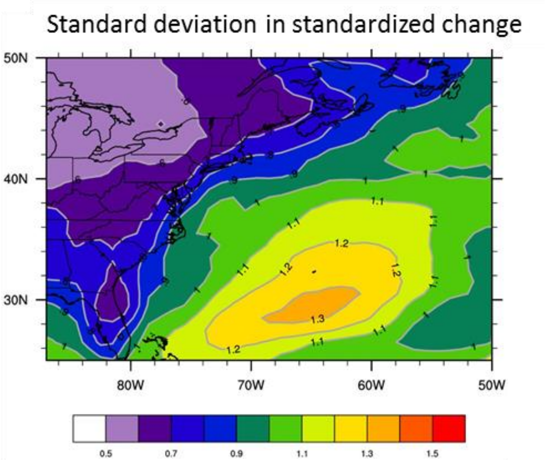

Maps were obtained from [NOAA's Climate Change Web Portal](#).

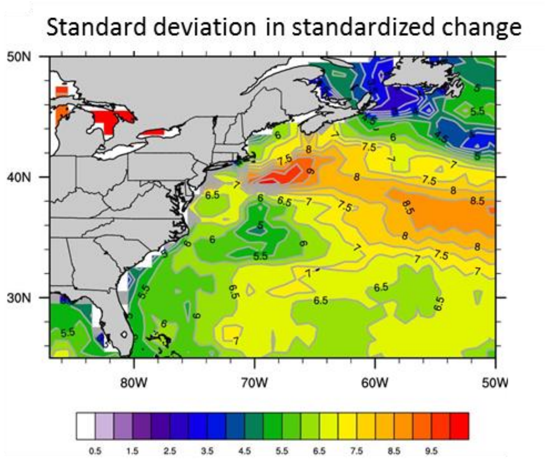

Supplement: S12 Supporting Information — (PDF) [file pone.0146756.s014.pdf]
